# Supplementary material for: High prevalence of burnout syndrome among medical and nonmedical residents during the COVID-19 pandemic
Source: PLoS One. 2022 Nov 22;17(11):e0267530. doi: 10.1371/journal.pone.0267530 (PMC9681108; doi:10.1371/journal.pone.0267530)
Supplement: S1 File — (PDF) [file pone.0267530.s002.pdf]

```
GET
  FILE='C:\Users\User\Documents\Pesquisa\Fellow\FellowGenData_V1.sav'.
DATASET NAME ConjuntodeDados1 WINDOW=FRONT.
FREQUENCIES VARIABLES=Sex Race ComorbAny HRiskFactor EBSERH FinSupp UnivHosp RsPrgType ProfCateg
    MDSpecBA
  /BARCHART PERCENT
  /ORDER=ANALYSIS.

FREQUENCIES VARIABLES=Sex Race ComorbAny HRiskFactor EBSERH FinSupp UnivHosp RsPrgType ProfCateg
    MDSpecBA
  /BARCHART PERCENT
  /ORDER=VARIABLE.
```

Frequências

Observações

Saída criada		20-SEP-2020 11:16:53
Comentários		
Entrada	Dados	C:\Users\User\Documents\Pesquisa\Fellow\FellowGenData_V1.sav
	Conjunto de dados ativo	ConjuntodeDados1
	Filtro	<none>
	Ponderação	<none>
	Arquivo Dividido	<none>
	N de linhas em arquivo de dados de trabalho	1313
Tratamento de valor omisso	Definição de omisso	Os valores omissos definidos pelo usuário são tratados como omissos.
	Casos utilizados	As estatísticas estão baseadas em todos os casos com dados válidos.
Sintaxe		FREQUENCIES VARIABLES=Sex Race ComorbAny HRiskFactor EBSERH FinSupp UnivHosp RsPrgType ProfCateg MDSpecBA /BARCHART PERCENT /ORDER=VARIABLE.
Recursos	Tempo do processador	00:00:02,95
	Tempo decorrido	00:00:01,52

Sex

Estatísticas

Sex

N	Válido	1310
	Omisso	3

Sex

		Frequência	Porcentagem	Porcentagem válida	Porcentagem acumulativa
Válido	Male	285	21,7	21,8	21,8
	Female	1025	78,1	78,2	100,0
	Total	1310	99,8	100,0	
Omisso	999	3	,2		
Total		1313	100,0		

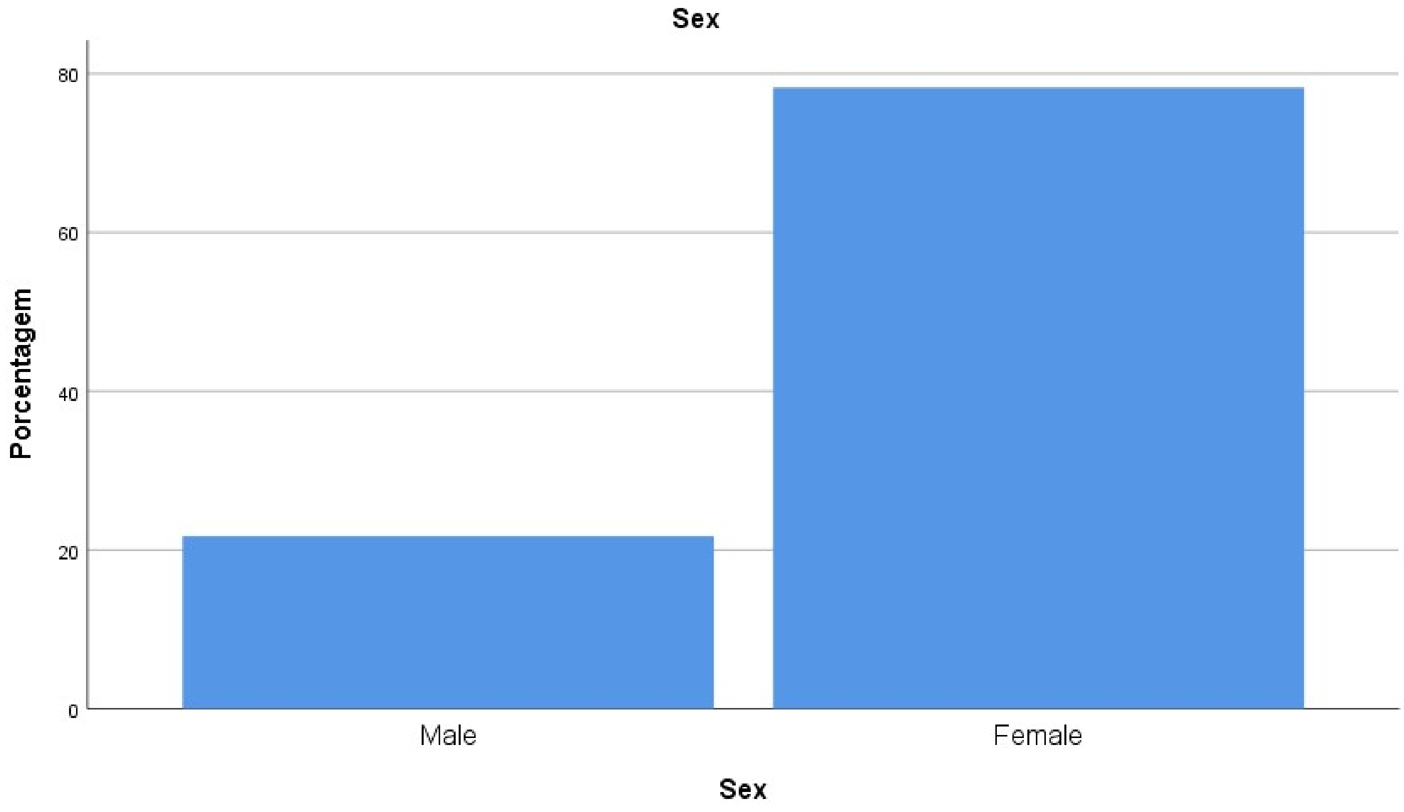

Race

Estatísticas

Race

N	Válido	1313

	Omisso	0

Race

		Frequência	Porcentagem	Porcentagem válida	Porcentagem acumulativa
Válido	White	778	59,3	59,3	59,3
	Pardo	433	33,0	33,0	92,2
	Black	81	6,2	6,2	98,4
	Other	21	1,6	1,6	100,0
	Total	1313	100,0	100,0	

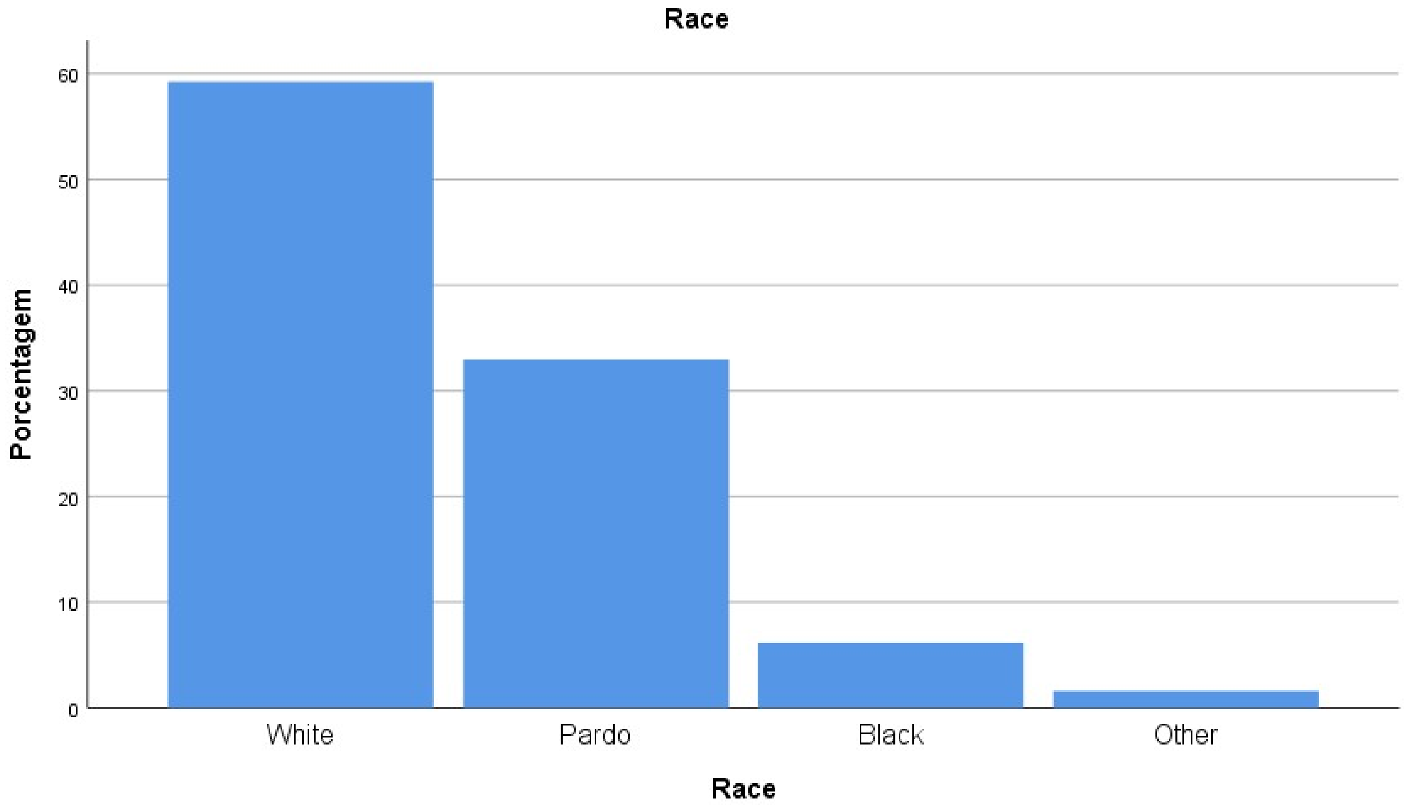


```
Any comorbidity (regardless COVID risk)
```

Estatísticas

Any comorbidity (regardless COVID risk)

N	Válido	1305
	Omisso	8

Any comorbidity (regardless COVID risk)

		Frequência	Porcentagem	Porcentagem válida	Porcentagem acumulativa
Válido	No	1071	81,6	82,1	82,1
	Yes	234	17,8	17,9	100,0
	Total	1305	99,4	100,0	
Omisso	999	8	,6		
Total		1313	100,0		

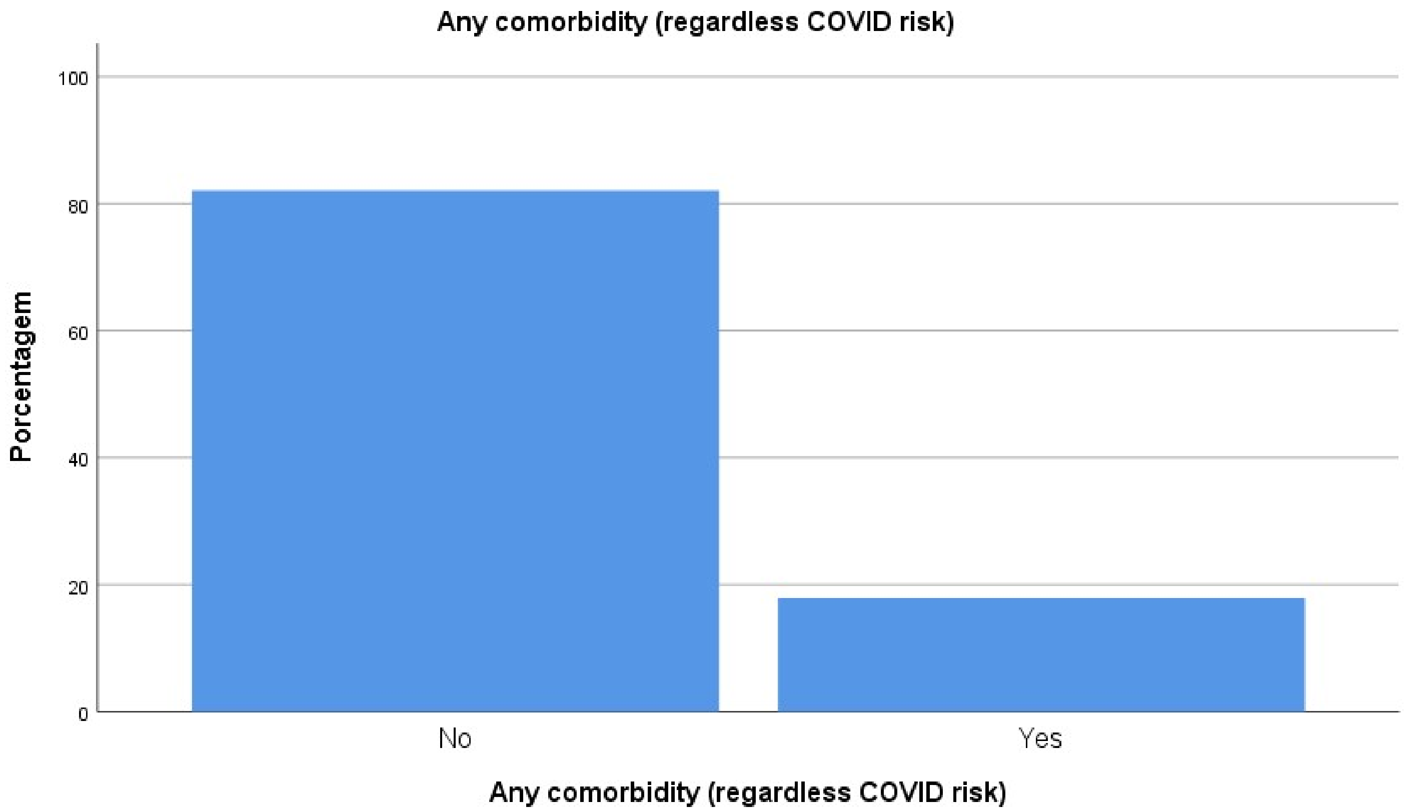

Risk factor(s) for severe COVID19

Estatísticas

Risk factor(s) for severe COVID19

N	Válido	1305
	Omisso	8

Risk factor(s) for severe COVID19

		Frequência	Porcentagem	Porcentagem válida	Porcentagem acumulativa
Válido	No	1087	82,8	83,3	83,3
	Yes	218	16,6	16,7	100,0

	Total	1305	99,4	100,0	
Omisso	999	8	,6		
Total		1313	100,0		

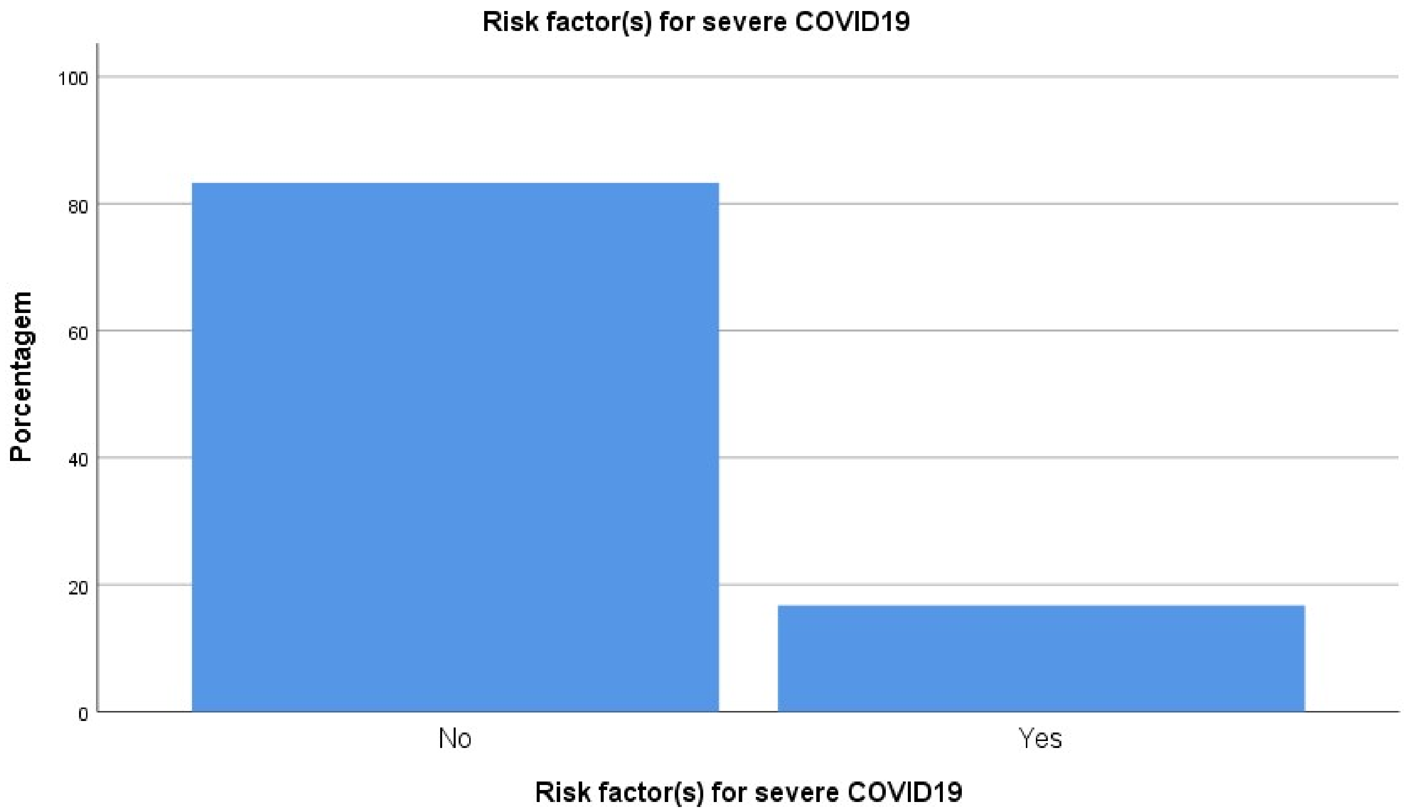


```
Institution belongs to EBSERH
```

Estatísticas

Institution belongs to EBSERH

N	Válido	1313
	Omisso	0

Institution belongs to EBSERH

		Frequência	Porcentagem	Porcentagem válida	Porcentagem acumulativa
Válido	No	206	15,7	15,7	15,7
	Yes	1107	84,3	84,3	100,0
	Total	1313	100,0	100,0	

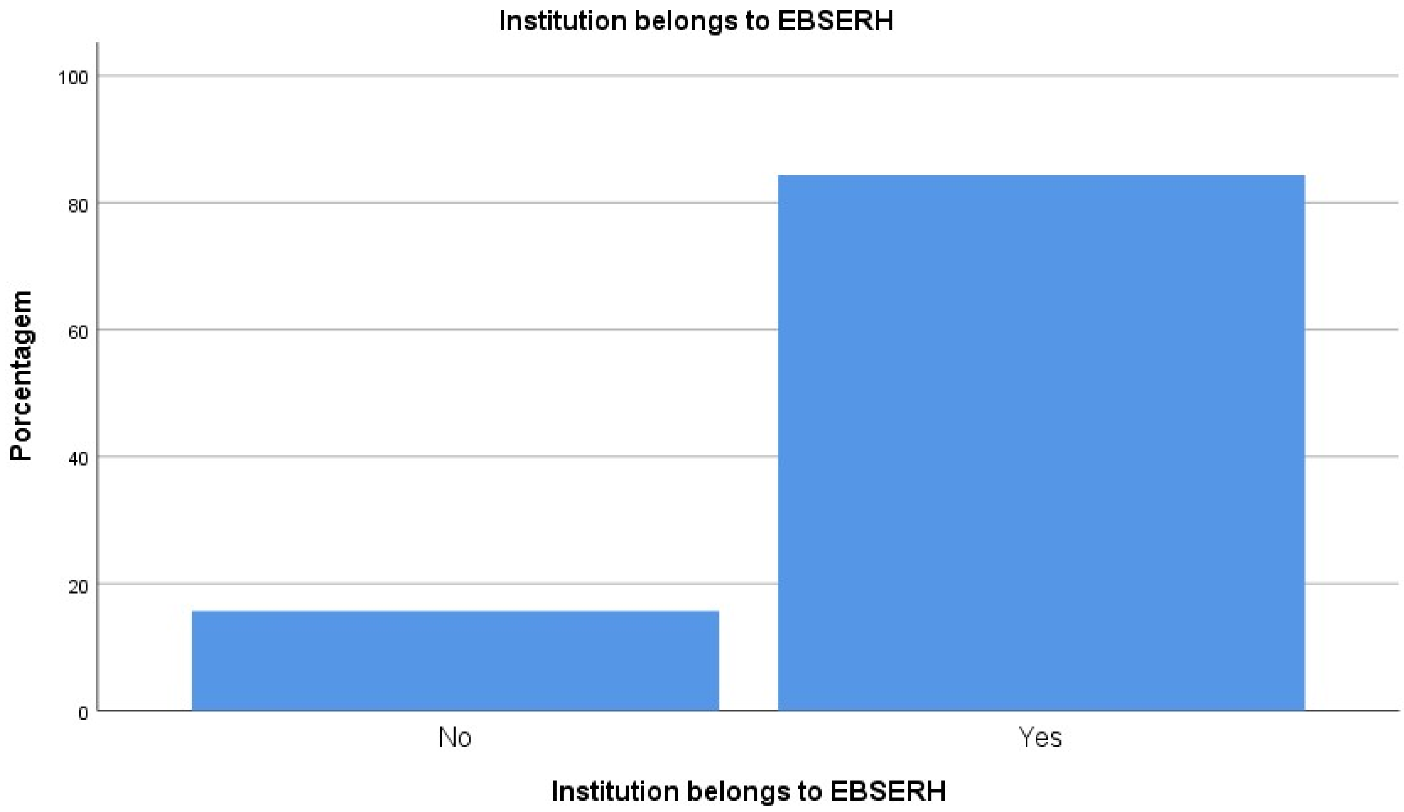

Main financial source of the institution

Estatísticas

Main financial source of the institution

N	Válido	1313
	Omisso	0

Main financial source of the institution

		Frequência	Porcentagem	Porcentagem válida	Porcentagem acumulativa
Válido	Public	1270	96,7	96,7	96,7
	Philanthropic	32	2,4	2,4	99,2
	Private	11	,8	,8	100,0
	Total	1313	100,0	100,0	

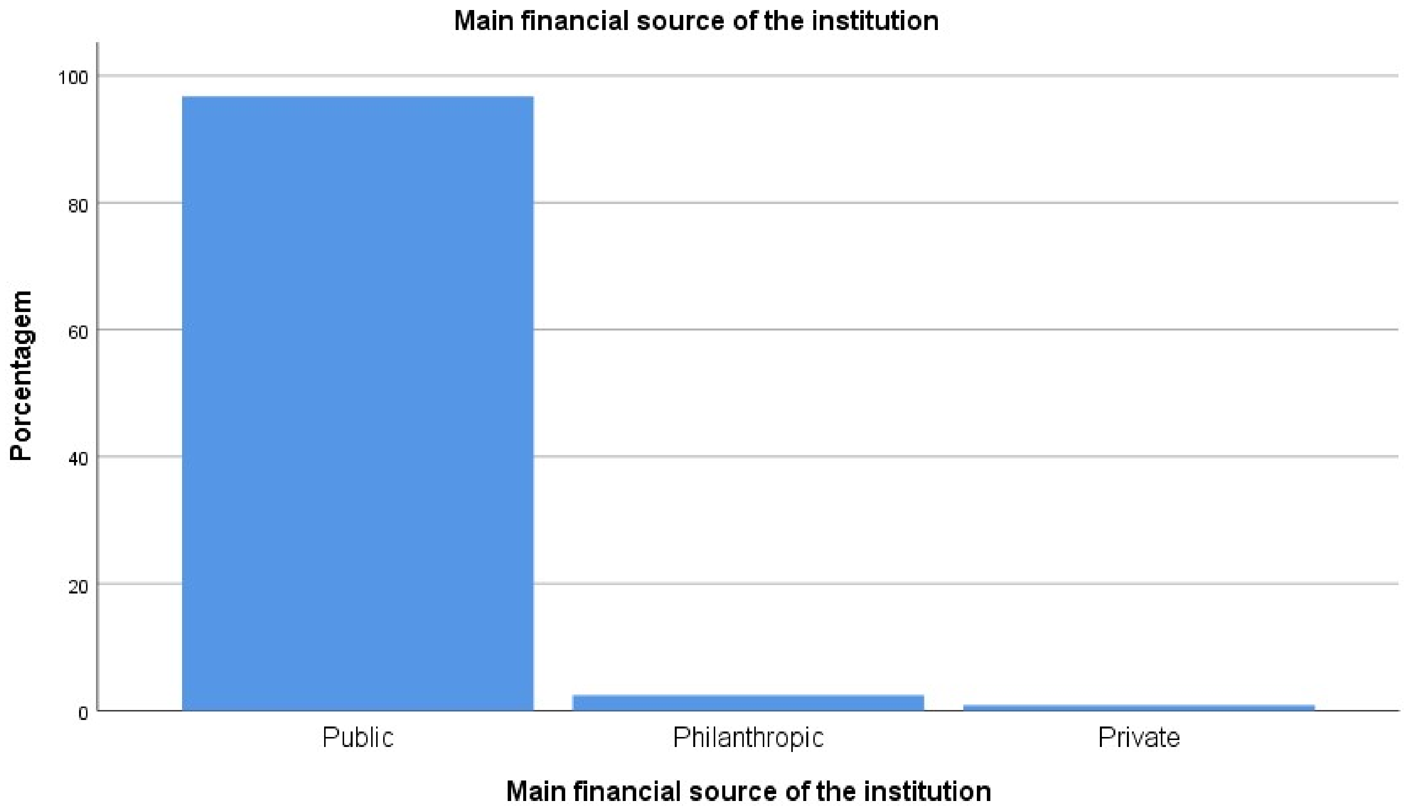

The institution is a university hospital

Estatísticas

The institution is a university hospital

N	Válido	1313
	Omisso	0

The institution is a university hospital

		Frequência	Porcentagem	Porcentagem válida	Porcentagem acumulativa
Válido	No	136	10,4	10,4	10,4
	Yes	1177	89,6	89,6	100,0
	Total	1313	100,0	100,0	

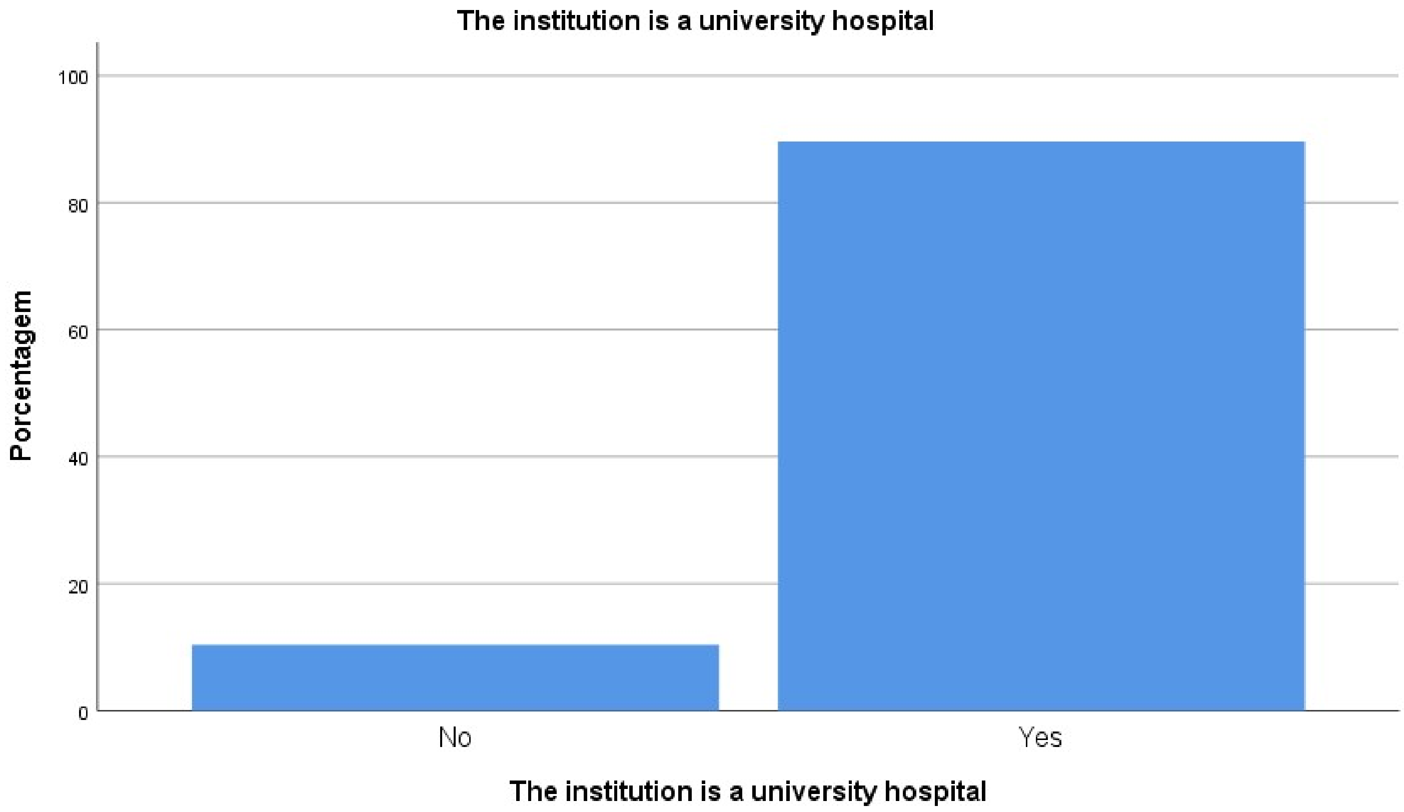

Type of residency program

Estatísticas

Type of residency program

N	Válido	1313
	Omisso	0

Type of residency program

		Frequência	Porcentagem	Porcentagem válida	Porcentagem acumulativa
Válido	Non-Medical (Other HCP)	639	48,7	48,7	48,7
	Medical	674	51,3	51,3	100,0
	Total	1313	100,0	100,0	

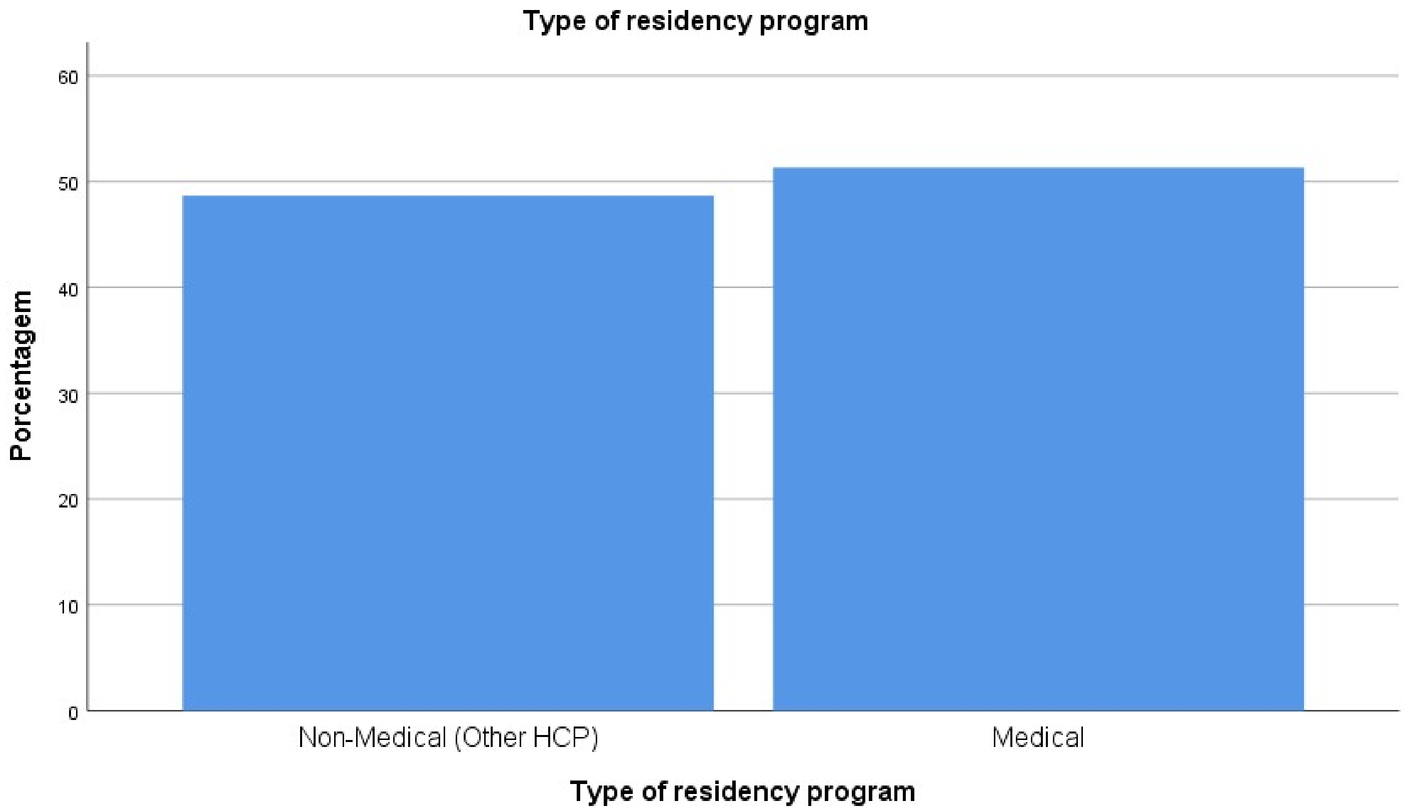

Professional category of the participant

Estatísticas

Professional category of the participant

N	Válido	1272
	Omisso	41

Professional category of the participant

		Frequência	Porcentagem	Porcentagem válida	Porcentagem acumulativa
Válido	Physicians	674	51,3	53,0	53,0
	Nurses	115	8,8	9,0	62,0
	Pharmacists	91	6,9	7,2	69,2
	Nutritionists	82	6,2	6,4	75,6
	Psychologists	82	6,2	6,4	82,1
	Physiotherapists	63	4,8	5,0	87,0
	Social Assistants	51	3,9	4,0	91,0
	Dentists	37	2,8	2,9	93,9
	Occupational therapists	22	1,7	1,7	95,7
	Others	55	4,2	4,3	100,0
	Total	1272	96,9	100,0	

Omisso	999	41	3,1		
Total		1313	100,0		

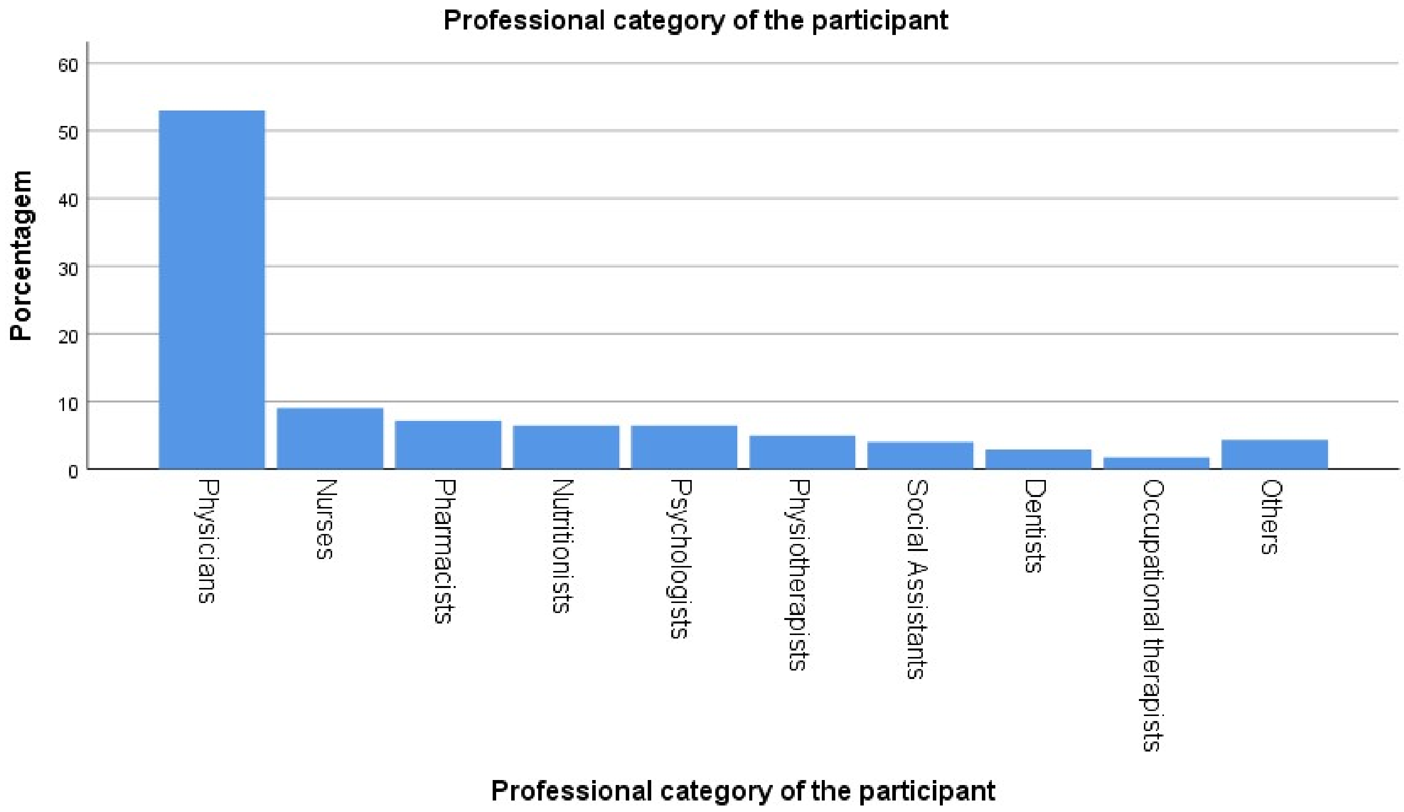

Medical specialty program - Broad Area (physicians only)

Estatísticas

Medical specialty program - Broad Area (physicians only)

N	Válido	674
	Omisso	639

Medical specialty program - Broad Area (physicians only)

		Frequência	Porcentagem	Porcentagem válida	Porcentagem acumulativa
Válido	Intensive, Emergency and General Clinical Care	138	10,5	20,5	20,5
	Clinical Subspecialties	186	14,2	27,6	48,1
	Surgical Specialties (general surgery included)	114	8,7	16,9	65,0
	Gynaecology and Obstetrics (subspecialties included)	66	5,0	9,8	74,8

	Pediatrics (subspecialties included)	124	9,4	18,4	93,2
	Diagnostic Medicine Specialties	46	3,5	6,8	100,0
	Total	674	51,3	100,0	
Omisso	999	639	48,7		
Total		1313	100,0		

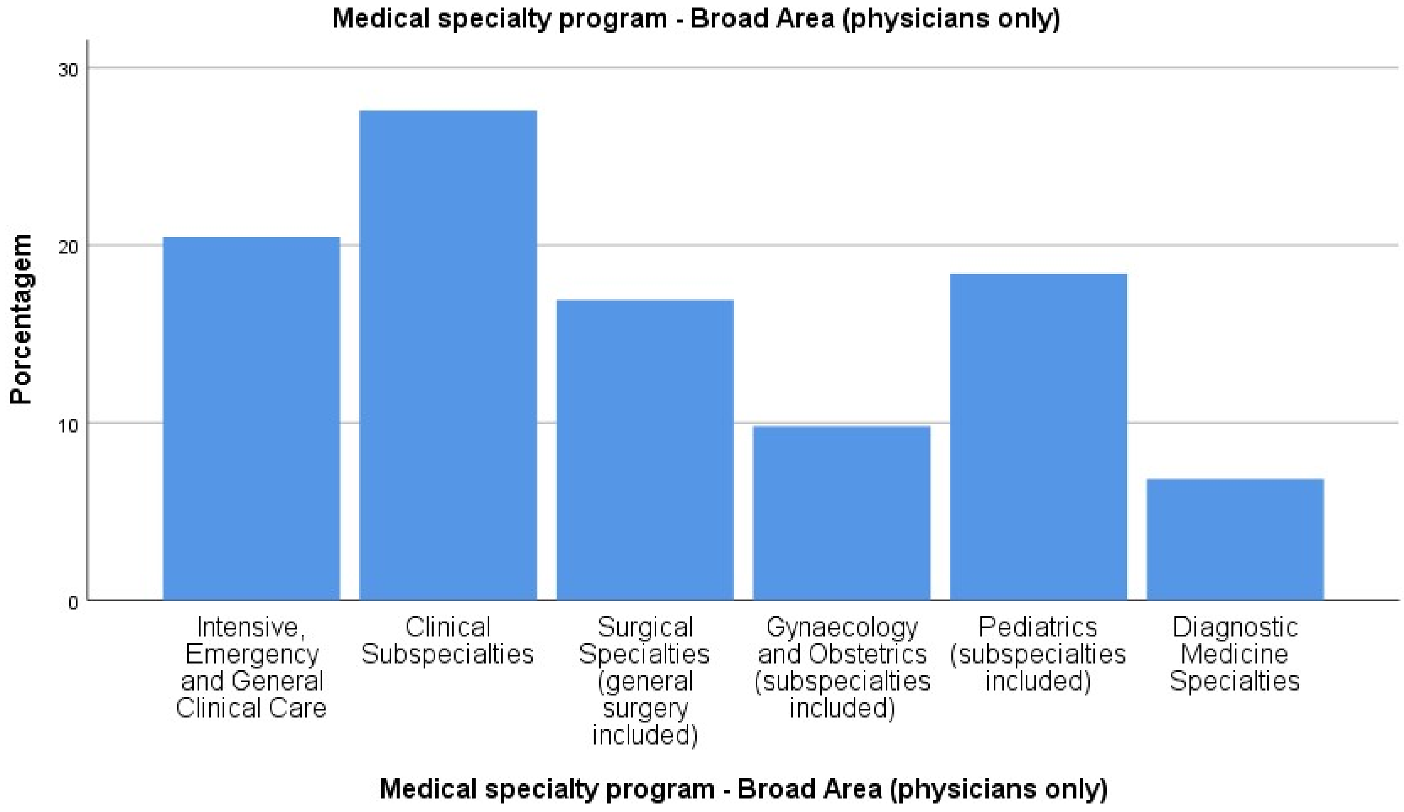


```
FREQUENCIES VARIABLES=Institution FedState ResidProgName MedSpec
  /FORMAT=DFREQ
  /ORDER=VARIABLE.
```

Frequências

Observações

Saída criada		20-SEP-2020 11:17:27
Comentários		
Entrada	Dados	C:\Users\User\Documents\Pesquisa\Fellow\FellowGenData_V1.sav
	Conjunto de dados ativo	ConjuntodeDados1
	Filtro	<none>
	Ponderação	<none>
	Arquivo Dividido	<none>

	N de linhas em arquivo de dados de trabalho	1313
Tratamento de valor omisso	Definição de omisso	Os valores omissos definidos pelo usuário são tratados como omissos.
	Casos utilizados	As estatísticas estão baseadas em todos os casos com dados válidos.
Sintaxe		FREQUENCIES VARIABLES=Institution FedState ResidProgName MedSpec<br>/FORMAT=DFREQ<br>/ORDER=VARIABLE.
Recursos	Tempo do processador	00:00:00,02
	Tempo decorrido	00:00:00,04

```
Institution of residency program
```

Estatísticas

Institution of residency program

N	Válido	1312
	Omisso	1

Institution of residency program

		Frequência	Porcentagem	Porcentagem válida	Porcentagem acumulativa
Válido	Hospital Universitário da Universidade de Brasília	116	8,8	8,8	8,8
	Complexo do Hospital de Clínicas da Universidade Federal do Paraná	113	8,6	8,6	17,5
	Hospital Universitário da Universidade Federal de Sergipe	59	4,5	4,5	22,0
	Hospital Universitário Onofre Lopes da Universidade Federal do Rio Grande do Norte	58	4,4	4,4	26,4
	Hospital Universitário da Universidade Federal do Piauí	56	4,3	4,3	30,6
	Hospital Universitário da Universidade Federal de Juiz de Fora	51	3,9	3,9	34,5
	Hospital das Clínicas da Universidade Federal de Goiás	48	3,7	3,7	38,2

	Complexo Hospitalar da Universidade Federal do Ceará	41	3,1	3,1	41,3
	Hospital de Clínicas da Universidade Federal de Uberlândia	39	3,0	3,0	44,3
	Hospital Universitário Antonio Pedro da Universidade Federal Fluminense	39	3,0	3,0	47,3
	Hospital Universitário da Universidade Federal de Santa Catarina	38	2,9	2,9	50,2
	Hospital Universitário da Universidade Federal do Maranhão	36	2,7	2,7	52,9
	Hospital Universitário de Santa Maria da Universidade Federal de Santa Maria	33	2,5	2,5	55,4
	Hospital das Clínicas da Universidade Federal de Minas Gerais	32	2,4	2,4	57,9
	Hospital das Clínicas da Universidade Federal de Pernambuco	27	2,1	2,1	59,9
	Hospital Universitário Lauro Wanderley da Universidade Federal da Paraíba	27	2,1	2,1	62,0
	Hospital Universitário Professor Edgard Santos da Universidade Federal da Bahia	27	2,1	2,1	64,0
	Hospital Universitário Júlio Müller da Universidade Federal do Mato Grosso	26	2,0	2,0	66,0
	Hospital Universitário Getúlio Vargas da Universidade Federal do Amazonas	23	1,8	1,8	67,8
	Hospital Universitário Cassiano Antonio de Moraes da Universidade Federal do Espírito Santo	21	1,6	1,6	69,4
	Hospital Universitário da Universidade Federal da Grande Dourados	21	1,6	1,6	71,0
	Complexo Hospitalar Universitário da Universidade Federal do Pará	20	1,5	1,5	72,5
	Hospital Escola da Universidade Federal de Pelotas	20	1,5	1,5	74,0

	Hospital de Clínicas da Universidade Federal do Triângulo Mineiro	19	1,4	1,4	75,5
	Hospital Universitário Ana Bezerra da Universidade Federal do Rio Grande do Norte	19	1,4	1,4	76,9
	Hospital Universitário Maria Aparecida Pedrossian da Universidade Federal de Mato Grosso do Sul	16	1,2	1,2	78,1
	Instituto Hospital de Base de Brasília	15	1,1	1,1	79,3
	Hospital Universitário Dr. Miguel Riet Corrêa Júnior da Universidade Federal do Rio Grande	14	1,1	1,1	80,3
	Hospital Universitário Professor Alberto Antunes da Universidade Federal de Alagoas	14	1,1	1,1	81,4
	Complexo Hospitalar da Universidade Federal do Ceará (Maternidade Escola Assis Chateaubriand)	12	,9	,9	82,3
	Hospital Universitário de Lagarto	11	,8	,8	83,2
	UBS/ Atenção Primária SES-DF	10	,8	,8	83,9
	Hospital da Região Leste	9	,7	,7	84,6
	Hospital de Ensino Dr. Washington Antônio de Barros da Universidade Federal do Vale do São Francisco	9	,7	,7	85,3
	Hospital Regional de Sobradinho	8	,6	,6	85,9
	Hospital Regional de Taguatinga	7	,5	,5	86,4
	Hospital Regional do Gama	7	,5	,5	87,0
	Hospital São Lucas da PUCRS	6	,5	,5	87,4
	Maternidade Escola Januário Cicco da Universidade Federal do Rio Grande do Norte	6	,5	,5	87,9
	Hospital Regional de Ceilândia	5	,4	,4	88,3
	Hospital Universitário Alcides Carneiro da Universidade Federal de Campina Grande	5	,4	,4	88,6
	Hospital Universitário de Londrina	5	,4	,4	89,0
	Hospital de Clínicas de Porto Alegre	4	,3	,3	89,3
	Hospital de Doenças Tropicais da Universidade Federal do Tocantins	4	,3	,3	89,6

	Hospital Infantil Lucidio Portela	4	,3	,3	89,9
	Hospital Regional da Asa Norte	4	,3	,3	90,2
	Hospital Regional de Santa Maria	4	,3	,3	90,5
	Hospital São Sebastião	4	,3	,3	90,9
	Secretaria de Saúde do Distrito Federal	4	,3	,3	91,2
	UBS/ Atenção Primária SES-RS	4	,3	,3	91,5
	Hospital de Clínicas de Itajubá	3	,2	,2	91,7
	Hospital Universitário Gaffrée e Guinle da Universidade Federal do Estado do Rio de Janeiro	3	,2	,2	91,9
	Hospital Universitário Pedro Ernesto	3	,2	,2	92,1
	Irmandade Nossa Senhora das Mercês Santa Casa de Montes Claros	3	,2	,2	92,4
	UBS/ Atenção Básica - SES/SC	3	,2	,2	92,6
	UBS/ Atenção Primária - SES/SE	3	,2	,2	92,8
	Centro Estadual de Reabilitação e Readaptação Dr Henrique Santillo	2	,2	,2	93,0
	Hospital Colônia Dr. João Machado	2	,2	,2	93,1
	Hospital da Região Leste (HRPa / SES-DF)	2	,2	,2	93,3
	Hospital de Apoio de Brasília	2	,2	,2	93,4
	Hospital de Base Rio Preto	2	,2	,2	93,6
	Hospital João XXIII	2	,2	,2	93,8
	Hospital Maternidade Sofia Feldman	2	,2	,2	93,9
	Hospital Materno Infantil de Brasília	2	,2	,2	94,1
	Hospital Veterinário UFU	2	,2	,2	94,2
	Instituto Ortopédico de Goiânia	2	,2	,2	94,4
	Maternidade Odete Valadares	2	,2	,2	94,5
	Santa Casa Montes Claros	2	,2	,2	94,7
	UBS/ Atenção Primária - SES/SP	2	,2	,2	94,8
	Associacao Hospitalar Santa Rosalia	1	,1	,1	94,9
	Beneficência Portuguesa de São Paulo	1	,1	,1	95,0
	CAPS SES-SE	1	,1	,1	95,0
	CAPS Taguatinga SES-DF	1	,1	,1	95,1
	Centro Avançado de Oncologia	1	,1	,1	95,2
	Centro de Referência Municipal para COVID - Santa Maria	1	,1	,1	95,3
	Clínica Odontológica Universitária	1	,1	,1	95,4

	Complexo de Saúde São João de Deus	1	,1	,1	95,4
	Departamento de Patologia Animal da UFPel	1	,1	,1	95,5
	Escola Superior de Ciências da Saúde	1	,1	,1	95,6
	Escola Superior de Ciências da Saúde SES-DF	1	,1	,1	95,7
	Hospital 9 de Julho	1	,1	,1	95,7
	Hospital Beneficente UNIMAR	1	,1	,1	95,8
	Hospital Casa de Saúde	1	,1	,1	95,9
	Hospital Cesar Leite	1	,1	,1	96,0
	Hospital da Criança de Brasília	1	,1	,1	96,0
	Hospital da Criança Santo Antônio	1	,1	,1	96,1
	Hospital da PUC-Campinas	1	,1	,1	96,2
	Hospital da Restauração Gov. Paulo Guerra	1	,1	,1	96,3
	Hospital Daher Lago Sul	1	,1	,1	96,3
	Hospital das Clínicas da Faculdade de Medicina da Universidade de São Paulo	1	,1	,1	96,4
	Hospital das Forças Armadas	1	,1	,1	96,5
	Hospital de Base de São José do Rio Preto	1	,1	,1	96,6
	Hospital de Base Dr. Ary Pinheiro	1	,1	,1	96,6
	Hospital de Clínicas de Uberlândia	1	,1	,1	96,7
	Hospital de Clínicas Veterinárias	1	,1	,1	96,8
	Hospital de Clínicas Veterinárias - UFPel	1	,1	,1	96,9
	Hospital de Urgência de Sergipe	1	,1	,1	97,0
	Hospital do Trabalhador	1	,1	,1	97,0
	Hospital e Maternidade Santa Isabel	1	,1	,1	97,1
	Hospital e Pronto Socorro 28 de Agosto	1	,1	,1	97,2
	Hospital Estadual da Criança	1	,1	,1	97,3
	Hospital Estadual Materno-Infantil Dr. Jurandir do Nascimento	1	,1	,1	97,3
	Hospital Evangélico Goiano	1	,1	,1	97,4
	Hospital Geral de Itapecerica da Serra	1	,1	,1	97,5
	Hospital Geral do Estado	1	,1	,1	97,6
	Hospital Getúlio Vargas	1	,1	,1	97,6
	Hospital HOME	1	,1	,1	97,7
	Hospital Infantil Varela Santiago	1	,1	,1	97,8
	Hospital Júlia Kubitschek	1	,1	,1	97,9

	Hospital Municipal Getúlio Vargas	1	,1	,1	97,9
	Hospital Otorrino Center	1	,1	,1	98,0
	Hospital Psiquiátrico São Pedro	1	,1	,1	98,1
	Hospital Regional Antônio Dias	1	,1	,1	98,2
	Hospital regional da asa norte	1	,1	,1	98,2
	Hospital Regional da Asa Norte SES-DF	1	,1	,1	98,3
	Hospital Regional da Samambaia	1	,1	,1	98,4
	Hospital Regional de Cacoal	1	,1	,1	98,5
	Hospital Santa Isabel Ubá	1	,1	,1	98,6
	Hospital Santa Rosália	1	,1	,1	98,6
	Hospital São Lucas - Cascavel PR	1	,1	,1	98,7
	Hospital São Vicente de Paulo	1	,1	,1	98,8
	Hospital Universitário Cajuru	1	,1	,1	98,9
	Hospital Universitário Clemente de Faria	1	,1	,1	98,9
	Hospital Universitario da Universidade Federal de São Carlos	1	,1	,1	99,0
	Hospital Universitário da Universidade Federal do Vale do São Francisco	1	,1	,1	99,1
	Hospital Universitário de Santa Maria	1	,1	,1	99,2
	Hospital Universitário Regional Norte do Paraná	1	,1	,1	99,2
	Hospital Veterinário Universitário de Santa Maria	1	,1	,1	99,3
	Hospital Veterinário Universitário de Santa Maria - UFSM	1	,1	,1	99,4
	Instituto Benjamin Constant	1	,1	,1	99,5
	Instituto de Assistência Médica ao Servidor Público Estadual	1	,1	,1	99,5
	Laboratório Regional de Diagnóstico da UFPel	1	,1	,1	99,6
	Maternidade Municipal Mãe Esperança	1	,1	,1	99,7
	Obras Sociais Irmã Dulce	1	,1	,1	99,8
	Posto de Saúde Lineu Jucá	1	,1	,1	99,8
	Santa Casa de Misericórdia de Cerquilho	1	,1	,1	99,9
	Santa Casa de Misericórdia de São Sebastião do Paraíso	1	,1	,1	100,0
	Total	1312	99,9	100,0	
Omisso	999	1	,1		
Total		1313	100,0		

Federal State

Estatísticas

Federal State

N	Válido	1313
	Omisso	0

Federal State

		Frequência	Porcentagem	Porcentagem válida	Porcentagem acumulativa
Válido	DF	205	15,6	15,6	15,6
	MG	172	13,1	13,1	28,7
	PR	124	9,4	9,4	38,2
	RS	92	7,0	7,0	45,2
	RN	87	6,6	6,6	51,8
	SE	76	5,8	5,8	57,6
	PI	61	4,6	4,6	62,2
	GO	55	4,2	4,2	66,4
	CE	54	4,1	4,1	70,5
	RJ	45	3,4	3,4	74,0
	SC	41	3,1	3,1	77,1
	PE	38	2,9	2,9	80,0
	MS	37	2,8	2,8	82,8
	MA	36	2,7	2,7	85,5
	PB	32	2,4	2,4	88,0
	BA	30	2,3	2,3	90,3
	MT	26	2,0	2,0	92,2
	AM	24	1,8	1,8	94,1
	ES	21	1,6	1,6	95,7
	PA	20	1,5	1,5	97,2
	AL	15	1,1	1,1	98,3
	SP	14	1,1	1,1	99,4
	TO	4	,3	,3	99,7
	RO	3	,2	,2	99,9
	RR	1	,1	,1	100,0
	Total	1313	100,0	100,0	

Residency program denomination

Estatísticas

Residency program denomination

N	Válido	1313
	Omisso	0

Residency program denomination

		Frequência	Porcentagem	Porcentagem válida	Porcentagem acumulativa
Válido	Medicina	671	51,1	51,1	51,1
	Enfermagem	114	8,7	8,7	59,8
	Farmácia	85	6,5	6,5	66,3
	Nutrição	82	6,2	6,2	72,5
	Psicologia	82	6,2	6,2	78,8
	Fisioterapia	63	4,8	4,8	83,5
	Serviço Social	49	3,7	3,7	87,3
	Odontologia	34	2,6	2,6	89,9
	Terapia ocupacional	22	1,7	1,7	91,5
	Multiprofissional	21	1,6	1,6	93,1
	Medicina Veterinária	19	1,4	1,4	94,6
	Fonoaudiologia	14	1,1	1,1	95,7
	Biomedicina	10	,8	,8	96,4
	Educação Física	8	,6	,6	97,0
	Farmácia-bioquímica	6	,5	,5	97,5
	Saúde da Família	5	,4	,4	97,9
	Saúde Coletiva	4	,3	,3	98,2
	Cirurgia Bucomaxilofacial	3	,2	,2	98,4
	Saúde Materno Infantil	2	,2	,2	98,6
	Saúde Mental	2	,2	,2	98,7
	Serviço Social - Saúde da Mulher	2	,2	,2	98,9
	Análises clínicas	1	,1	,1	98,9
	Cardiologia	1	,1	,1	99,0
	Cirurgia	1	,1	,1	99,1
	Cirurgia plástica	1	,1	,1	99,2
	Educação física	1	,1	,1	99,2
	Física Médica - Radiodiagnóstico	1	,1	,1	99,3
	Gestão Hospitalar	1	,1	,1	99,4
	Hematologia	1	,1	,1	99,5
	Medicina de Família e Comunidade	1	,1	,1	99,5
	Medicina do Trabalho	1	,1	,1	99,6
	MFC	1	,1	,1	99,7
	Neurointensivismotensivismo	1	,1	,1	99,8

	Neurologia	1	,1	,1	99,8
	Patologia cirúrgica	1	,1	,1	99,9
	Saúde do Adulto e do Idoso	1	,1	,1	100,0
	Total	1313	100,0	100,0	

```
Medical specialty program (physicians only)
```

Estatísticas

Medical specialty program (physicians only)

N	Válido	674
	Omisso	639

Medical specialty program (physicians only)

		Frequência	Porcentagem	Porcentagem válida	Porcentagem acumulativa
Válido	Pediatria	100	7,6	14,8	14,8
	Clínica Médica	81	6,2	12,0	26,9
	Ginecologia e Obstetrícia	64	4,9	9,5	36,4
	Cirurgia Geral	37	2,8	5,5	41,8
	Radiologia e Diagnóstico por Imagem	32	2,4	4,7	46,6
	Psiquiatria	28	2,1	4,2	50,7
	Dermatologia	27	2,1	4,0	54,7
	Anestesiologia	24	1,8	3,6	58,3
	Endocrinologia e Metabologia	21	1,6	3,1	61,4
	Neurologia	16	1,2	2,4	63,8
	Otorrinolaringologia	16	1,2	2,4	66,2
	Ortopedia e Traumatologia	14	1,1	2,1	68,2
	Patologia	14	1,1	2,1	70,3
	Infectologia	13	1,0	1,9	72,3
	Oftalmologia	13	1,0	1,9	74,2
	Gastroenterologia	12	,9	1,8	76,0
	Nefrologia	12	,9	1,8	77,7
	Reumatologia	12	,9	1,8	79,5
	Medicina de Família e Comunidade	10	,8	1,5	81,0
	Cardiologia	9	,7	1,3	82,3
	Geriatria	9	,7	1,3	83,7
	Hematologia e Hemoterapia	8	,6	1,2	84,9
	Neonatologia	7	,5	1,0	85,9

	Pneumologia	7	,5	1,0	86,9
	Cirurgia Pediátrica	6	,5	,9	87,8
	Medicina Intensiva	6	,5	,9	88,7
	Neurocirurgia	6	,5	,9	89,6
	Cirurgia do Aparelho Digestivo	5	,4	,7	90,4
	Cirurgia Plástica	5	,4	,7	91,1
	Cirurgia Vascular	5	,4	,7	91,8
	Neurologia pediátrica	5	,4	,7	92,6
	Urologia	4	,3	,6	93,2
	Infectologia pediátrica	3	,2	,4	93,6
	Alergia e Imunologia	3	,2	,4	94,1
	Medicina de Emergência	3	,2	,4	94,5
	Medicina de família e comunidade	3	,2	,4	95,0
	MFC	3	,2	,4	95,4
	Pneumologia pediátrica	3	,2	,4	95,8
	Endocrinologia pediátrica	2	,2	,3	96,1
	Endoscopia	2	,2	,3	96,4
	Medicina da família e comunidade	2	,2	,3	96,7
	Oncologia Clínica	2	,2	,3	97,0
	Medicina do Trabalho	1	,1	,1	97,2
	Cardiologia pediatrica	1	,1	,1	97,3
	Cirurgia Torácica	1	,1	,1	97,5
	Coloproctologia	1	,1	,1	97,6
	endoscopia	1	,1	,1	97,8
	Ginecologia e obstetrícia	1	,1	,1	97,9
	Hepatologia	1	,1	,1	98,1
	Homeopatia	1	,1	,1	98,2
	Intensiva pediátrica	1	,1	,1	98,4
	Mastologia	1	,1	,1	98,5
	Medicina de emergência	1	,1	,1	98,7
	MEDICINA DE EMERGENCIA	1	,1	,1	98,8
	medicina de familia e comunidade	1	,1	,1	99,0
	Medicina de familia e comunidade	1	,1	,1	99,1
	Medicina de Familia e Comunidade	1	,1	,1	99,3
	Medicina Física e Reabilitação	1	,1	,1	99,4
	medicina paliativa	1	,1	,1	99,6
	neonatologia	1	,1	,1	99,7
	Pré-requisito em Área Cirúrgica Básica	1	,1	,1	99,9
	Psiquiatria da Infância e Adolescência	1	,1	,1	100,0
	Total	674	51,3	100,0	
Omisso	999	639	48,7		
Total		1313	100,0		

```
FREQUENCIES VARIABLES=Wkload_5P ExternWork CovidCare PPEAvail PPEAvail_Classif
  /BARCHART PERCENT
  /ORDER=VARIABLE.
```

Frequências

Observações

Saída criada		20-SEP-2020 11:19:04
Comentários		
Entrada	Dados	C:\Users\User\Documents\Pesquisa\Fellow\FellowGenData_V1.sav
	Conjunto de dados ativo	ConjuntodeDados1
	Filtro	<none>
	Ponderação	<none>
	Arquivo Dividido	<none>
	N de linhas em arquivo de dados de trabalho	1313
Tratamento de valor omisso	Definição de omisso	Os valores omissos definidos pelo usuário são tratados como omissos.
	Casos utilizados	As estatísticas estão baseadas em todos os casos com dados válidos.
Sintaxe		FREQUENCIES VARIABLES=Wkload_5P ExternWork CovidCare PPEAvail PPEAvail_Classif /BARCHART PERCENT /ORDER=VARIABLE.
Recursos	Tempo do processador	00:00:01,61
	Tempo decorrido	00:00:00,81

Cumulative weekly workload (five categories)

Estatísticas

Cumulative weekly workload (five categories)

N	Válido	1313
	Omisso	0

Cumulative weekly workload (five categories)

		Frequência	Porcentagem	Porcentagem válida	Porcentagem acumulativa
Válido	|--- 24h	11	,8	,8	,8
	>24h --- 60h	530	40,4	40,4	41,2
	>60h --- 90h	682	51,9	51,9	93,1
	>90h --- 120h	81	6,2	6,2	99,3
	>120h	9	,7	,7	100,0
	Total	1313	100,0	100,0	

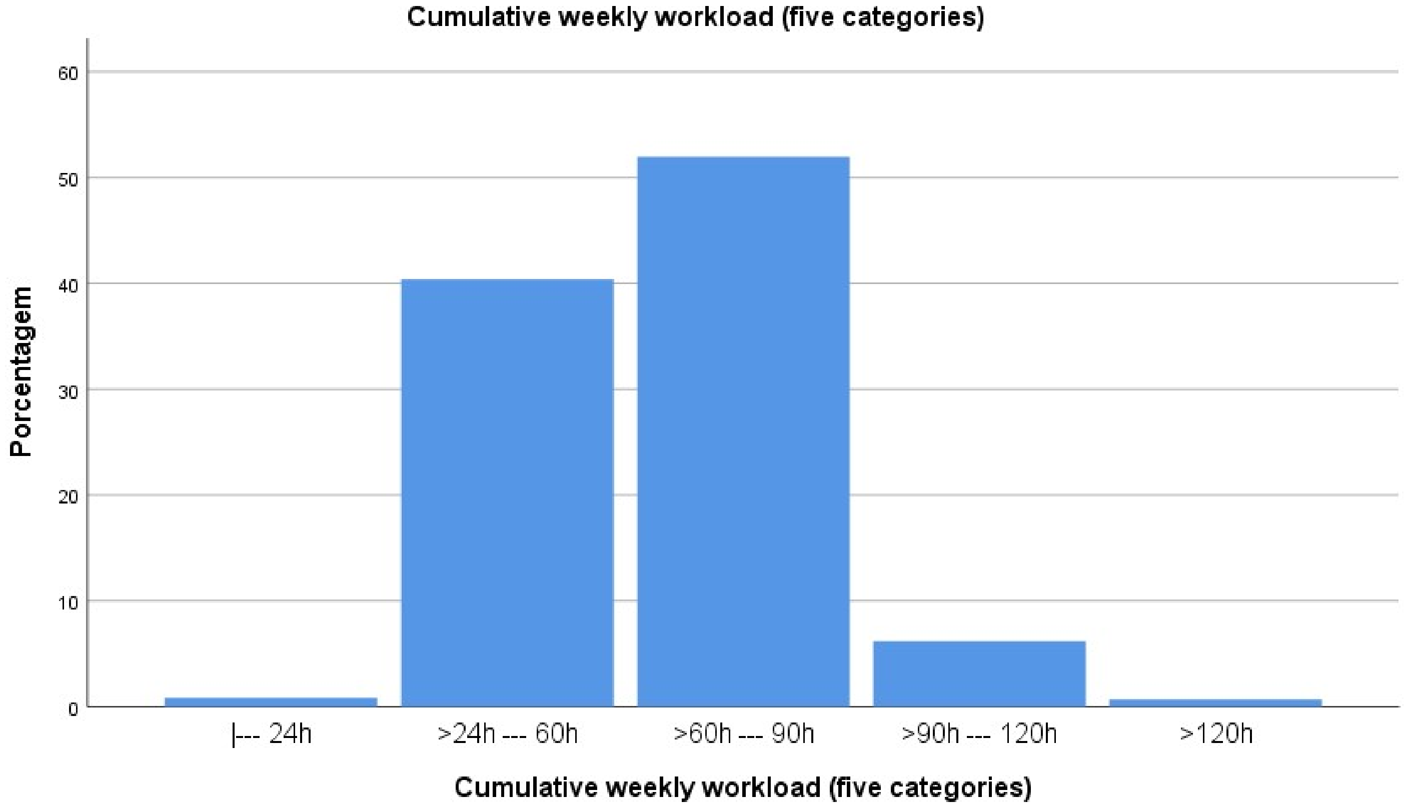


```
The participant exerts professional activity external to the residency program
```

Estatísticas

The participant exerts professional activity external to the residency program

N	Válido	1313
	Omisso	0

The participant exerts professional activity external to the residency program

		Frequência	Porcentagem	Porcentagem válida	Porcentagem acumulativa
Válido	No	889	67,7	67,7	67,7
	Yes	424	32,3	32,3	100,0
	Total	1313	100,0	100,0	

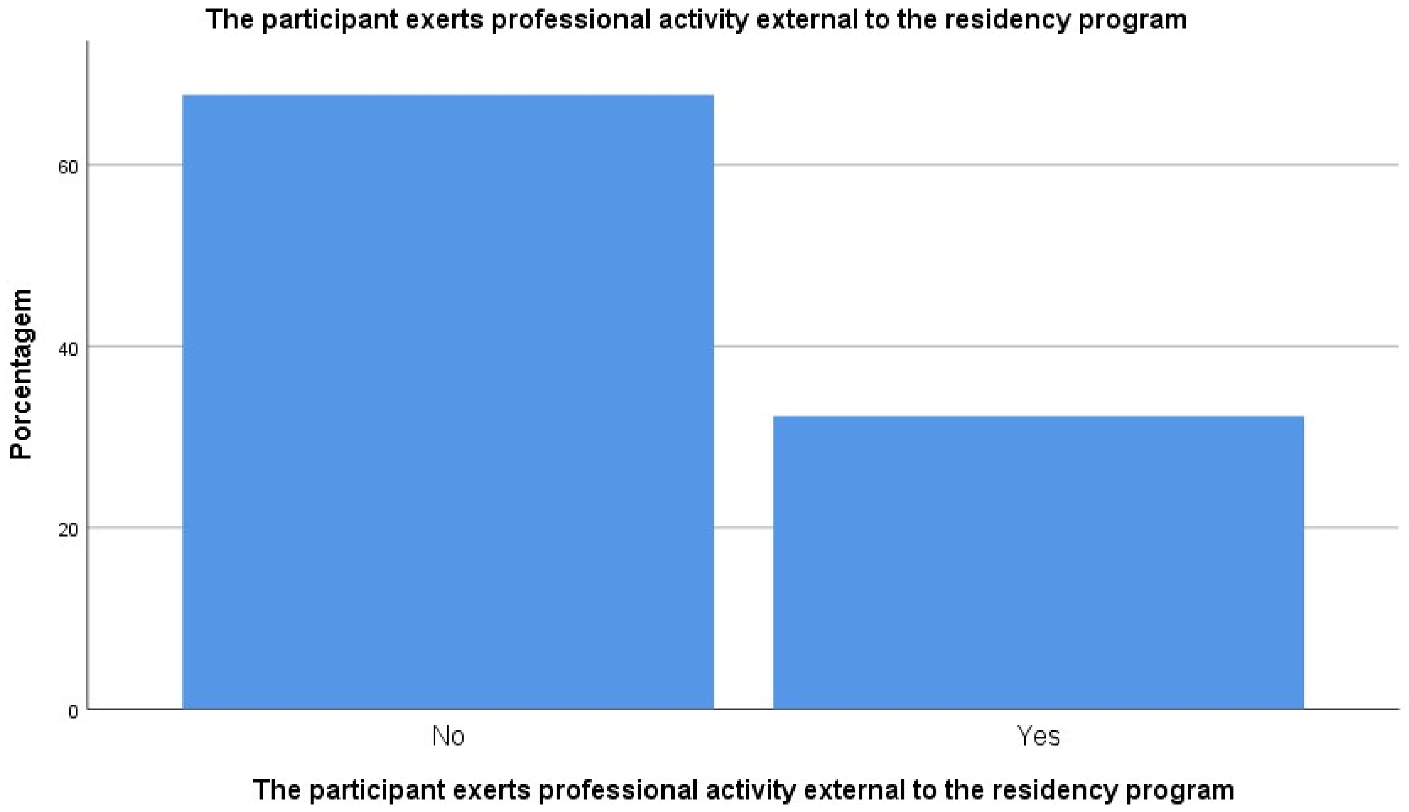

The participant provides direct health care for COVID19 patients

Estatísticas

The participant provides direct health care for COVID19 patients

N	Válido	1313
	Omisso	0

The participant provides direct health care for COVID19 patients

		Frequência	Porcentagem	Porcentagem válida	Porcentagem acumulativa
Válido	No	523	39,8	39,8	39,8
	Yes	790	60,2	60,2	100,0
	Total	1313	100,0	100,0	

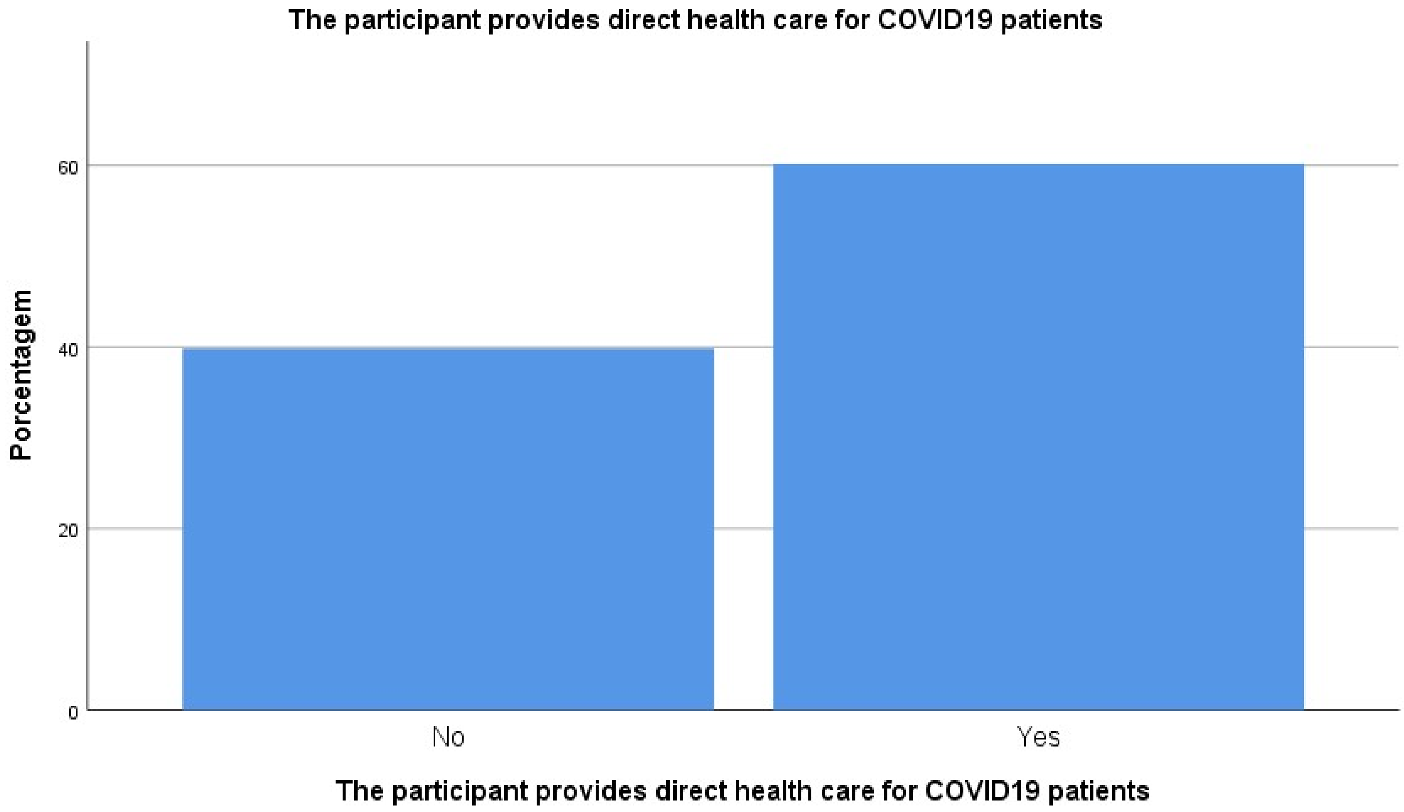


```
Na sua prática profissional, especialmente no atendimento a pacientes, por quanto tempo você tem acesso
a equipamentos de proteção individual (EPI) suficientes e adequados? (Lickert 1-5)
```

Estatísticas

Na sua prática profissional, especialmente no atendimento a pacientes, por quanto tempo você tem acesso a equipamentos de proteção individual (EPI) suficientes e adequados? (Lickert 1-5)

N	Válido	1313
	Omisso	0

Na sua prática profissional, especialmente no atendimento a pacientes, por quanto tempo você tem acesso a equipamentos de proteção individual (EPI) suficientes e adequados? (Lickert 1-5)

		Frequência	Porcentagem	Porcentagem válida	Porcentagem acumulativa
Válido	Em nenhum momento	18	1,4	1,4	1,4
	Em menos da metade do tempo	87	6,6	6,6	8,0
	Na metade do tempo	176	13,4	13,4	21,4

	Em mais da metade do tempo	483	36,8	36,8	58,2
	O tempo todo	549	41,8	41,8	100,0
	Total	1313	100,0	100,0	

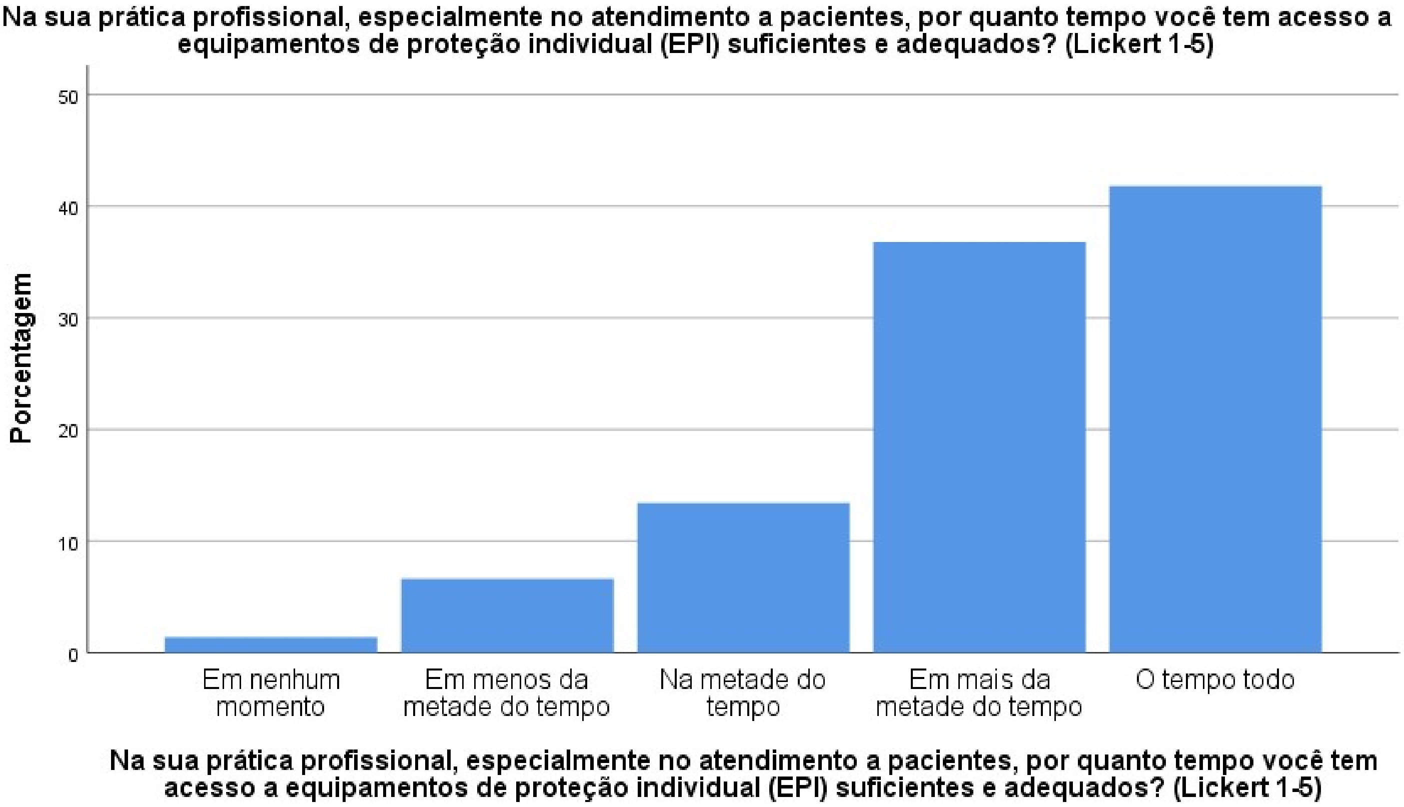


```
Perceived adequacy of the availability of personal protective equipment, when providing care for patients
in the residency program
```

Estatísticas

Perceived adequacy of the availability of personal protective equipment, when providing care for patients in the residency program

N	Válido	1313
	Omisso	0

Perceived adequacy of the availability of personal protective equipment, when providing care for patients in the residency program

		Frequência	Porcentagem	Porcentagem válida	Porcentagem acumulativa
Válido	Poor adequacy	281	21,4	21,4	21,4
	Good adequacy	1032	78,6	78,6	100,0

Total	1313	100,0	100,0	

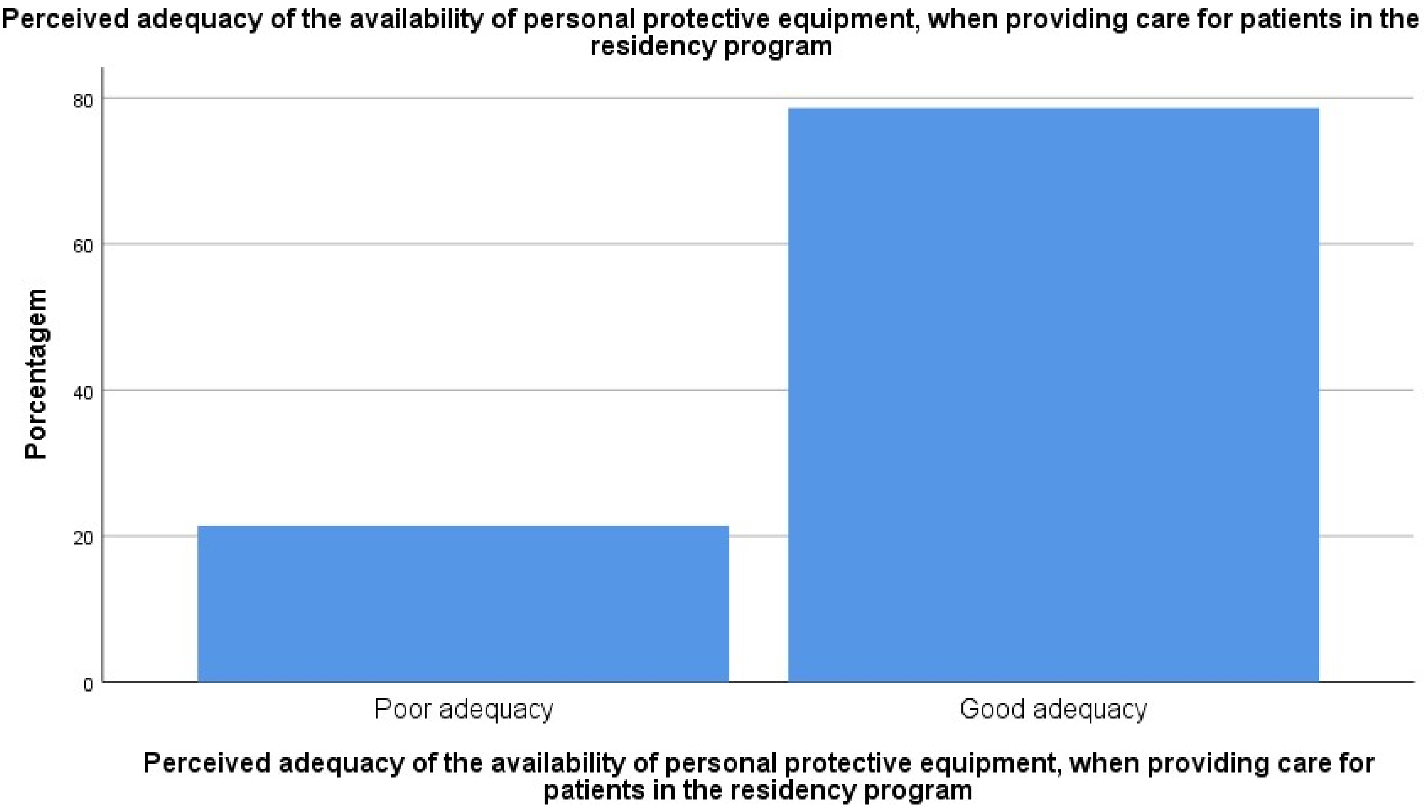


```
EXAMINE VARIABLES=Age Autonomy PedagStruct
  /PLOT HISTOGRAM NPPLOT
  /PERCENTILES(5,10,25,50,75,90,95) HAVERAGE
  /STATISTICS DESCRIPTIVES
  /CINTERVAL 95
  /MISSING PAIRWISE
  /NOTOTAL.
```

Explorar

Observações

Saída criada		20-SEP-2020 11:20:19
Comentários		
Entrada	Dados	C:\Users\User\Documents\Pesquisa\Fellow\FellowGenData_V1.sav
	Conjunto de dados ativo	ConjuntodeDados1
	Filtro	<none>
	Ponderação	<none>
	Arquivo Dividido	<none>
	N de linhas em arquivo de dados de trabalho	1313

Tratamento de valor omisso	Definição de omisso	Os valores omissos definidos pelo usuário para variáveis dependentes são tratados como omissos.
	Casos utilizados	As estatísticas são baseadas em casos sem valores omissos para a variável dependente ou fatores que estão sendo analisados.
Sintaxe		EXAMINE VARIABLES=Age Autonomy PedagStruct<br>/PLOT HISTOGRAM NPPLOT<br><br>/PERCENTILES(5,10,25,50,75,90,95) HAVERAGE<br>/STATISTICS DESCRIPTIVES<br>/CINTERVAL 95<br>/MISSING PAIRWISE<br>/NOTOTAL.
Recursos	Tempo do processador	00:00:01,78
	Tempo decorrido	00:00:01,53

Resumo de processamento do caso

	Casos					
	Válido		Omisso		Total	
	N	Porcentagem	N	Porcentagem	N	Porcentagem
Age	1230	93,7%	83	6,3%	1313	100,0%
Na sua opinião, qual o seu grau de autonomia para decidir condutas no trabalho? (EAV 1-10)	1313	100,0%	0	0,0%	1313	100,0%
Na sua opinião, qual o grau de adequação da organização pedagógica de seu programa de residência profissional? (EAV 1-10)	1313	100,0%	0	0,0%	1313	100,0%

Descritivos

			Estatística	Erro Erro
Age	Média		27,86	,126
	95% Intervalo de Confiança para Média	Limite inferior	27,61	
		Limite superior	28,11	
	5% da média aparada		27,44	
	Mediana		27,00	
	Variância		19,435	
	Erro Desvio		4,409	

	Mínimo		20	
	Máximo		61	
	Intervalo		41	
	Amplitude interquartil		5	
	Assimetria		2,146	,070
	Curtose		8,739	,139
Na sua opinião, qual o seu grau de autonomia para decidir condutas no trabalho? (EAV 1-10)	Média		6,51	,058
	95% Intervalo de Confiança para Média	Limite inferior	6,39	
		Limite superior	6,62	
	5% da média aparada		6,59	
	Mediana		7,00	
	Variância		4,433	
	Erro Desvio		2,105	
	Mínimo		1	
	Máximo		10	
	Intervalo		9	
	Amplitude interquartil		3	
	Assimetria		-,694	,068
	Curtose		-,062	,135
Na sua opinião, qual o grau de adequação da organização pedagógica de seu programa de residência profissional? (EAV 1-10)	Média		5,77	,068
	95% Intervalo de Confiança para Média	Limite inferior	5,64	
		Limite superior	5,91	
	5% da média aparada		5,81	
	Mediana		6,00	
	Variância		6,097	
	Erro Desvio		2,469	
	Mínimo		1	
	Máximo		10	
	Intervalo		9	
	Amplitude interquartil		4	
	Assimetria		-,341	,068
	Curtose		-,828	,135

Percentis

		Percentis						
		5	10	25	50	75	90	95
Média Ponderada (Definição 1)	Age	23,00	24,00	25,00	27,00	30,00	33,00	36,00
	Na sua opinião, qual o seu grau de autonomia para decidir condutas no trabalho? (EAV 1-10)	2,00	3,00	5,00	7,00	8,00	9,00	9,00

	Na sua opinião, qual o grau de adequação da organização pedagógica de seu programa de residência profissional? (EAV 1-10)	1,00	2,00	4,00	6,00	8,00	9,00	9,00
Teste de Tukey	Age			25,00	27,00	30,00		
	Na sua opinião, qual o seu grau de autonomia para decidir condutas no trabalho? (EAV 1-10)			5,00	7,00	8,00		
	Na sua opinião, qual o grau de adequação da organização pedagógica de seu programa de residência profissional? (EAV 1-10)			4,00	6,00	8,00		

Testes de Normalidade

	Kolmogorov-Smirnov[a]			Shapiro-Wilk		
	Estatística	df	Sig.	Estatística	df	Sig.
Age	,153	1230	,000	,846	1230	,000
Na sua opinião, qual o seu grau de autonomia para decidir condutas no trabalho? (EAV 1-10)	,187	1313	,000	,934	1313	,000
Na sua opinião, qual o grau de adequação da organização pedagógica de seu programa de residência profissional? (EAV 1-10)	,153	1313	,000	,948	1313	,000

a. Correlação de Significância de Lilliefors

Age

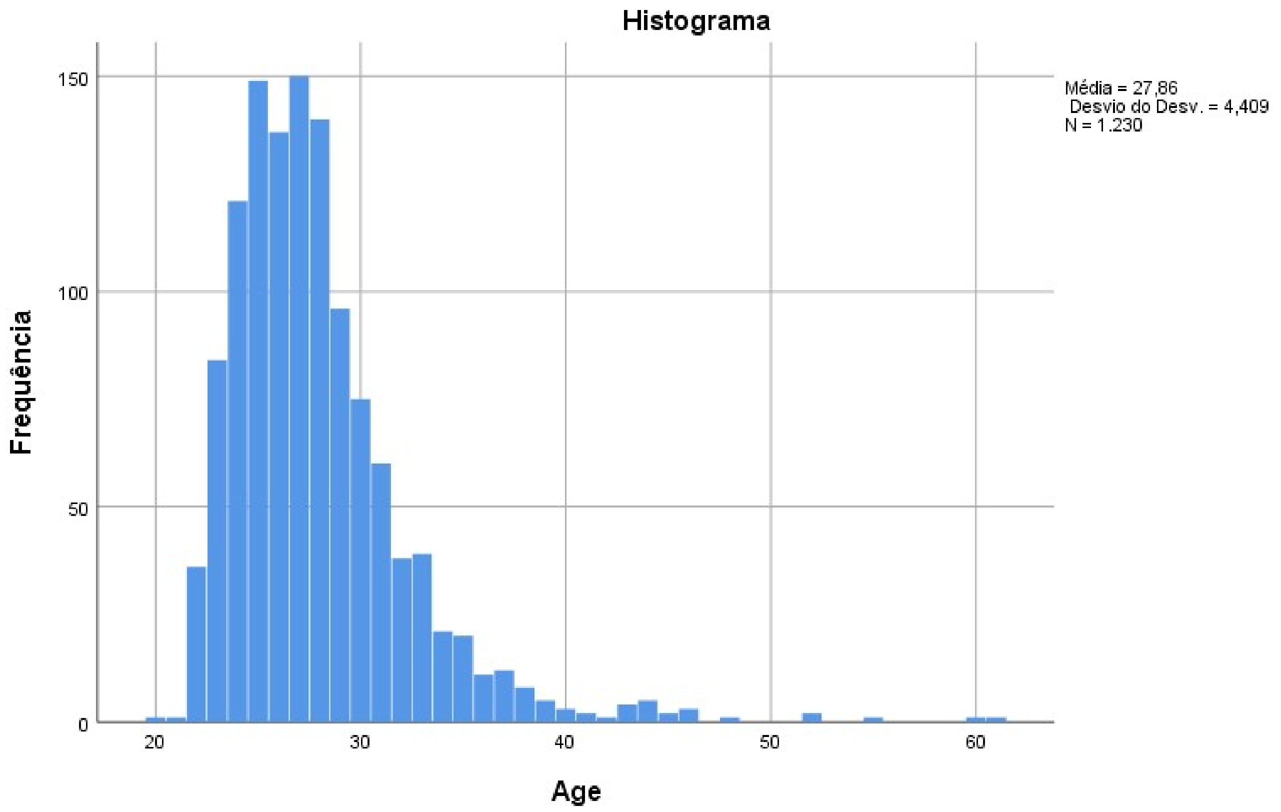
Histograma
Média = 27,86
Desvio do Desv. = 4,409
N = 1.230
Frequência
150
100
50
0
20
30
40
50
60
Age

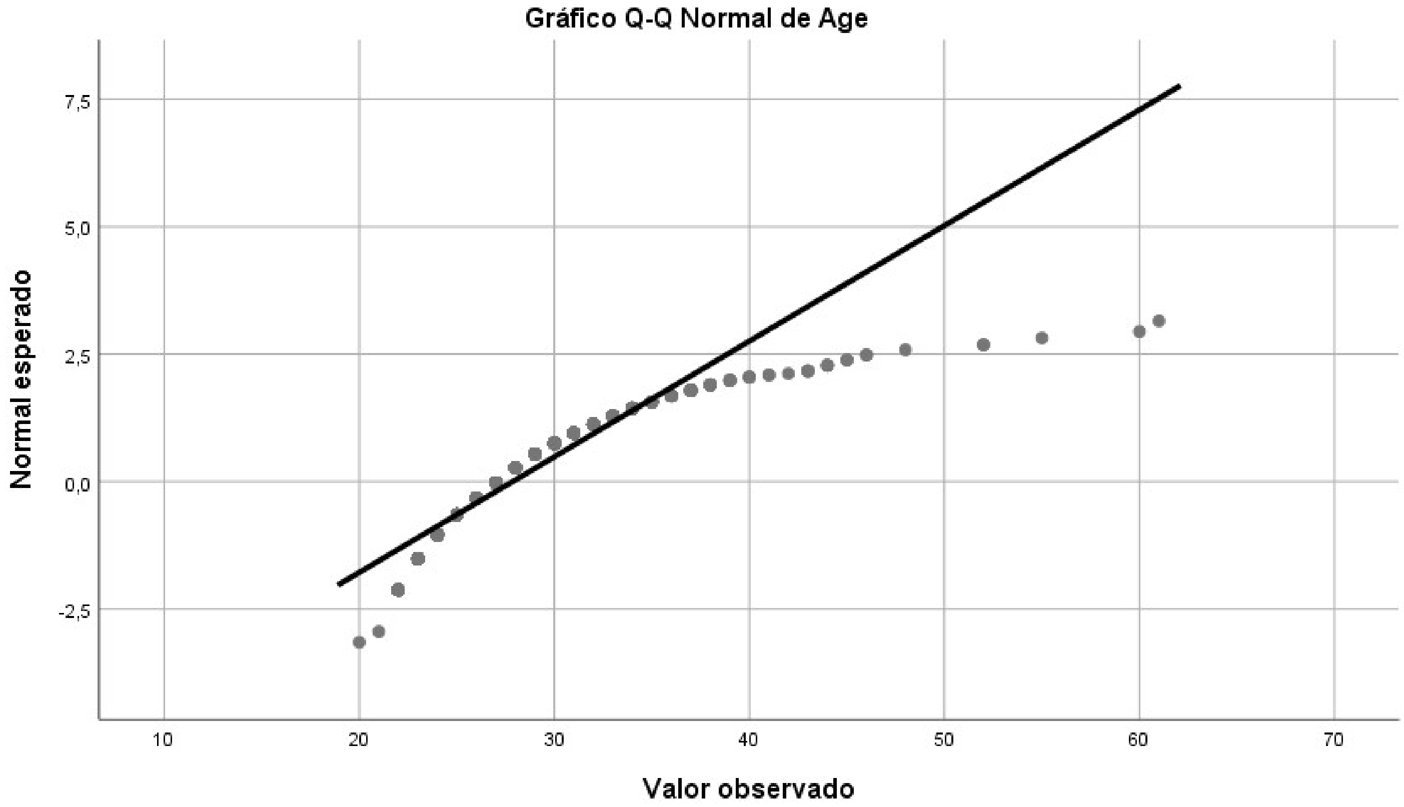
Gráfico Q-Q Normal de Age
Normal esperado
7,5
5,0
2,5
0,0
-2,5
10
20
30
40
50
60
70
Valor observado

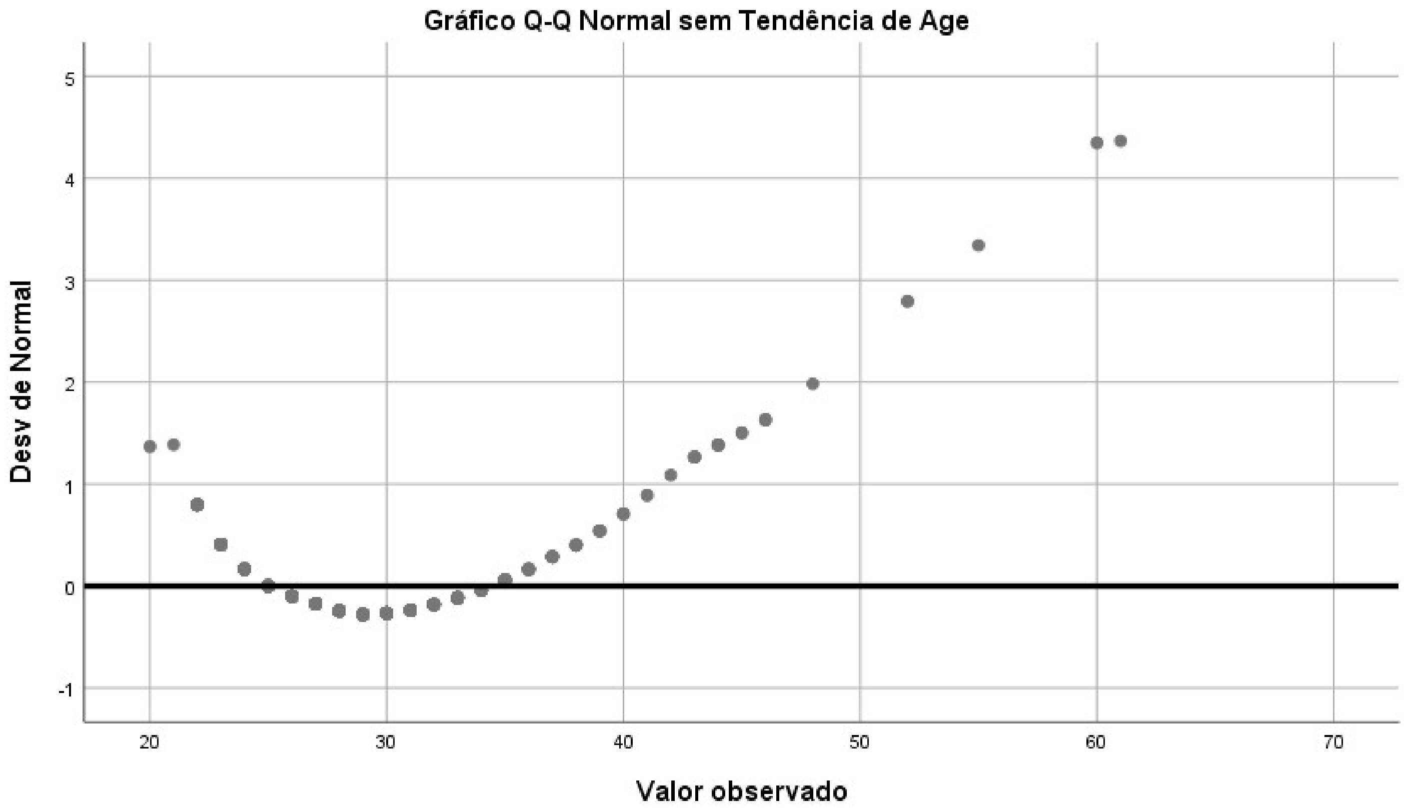

Na sua opinião, qual o seu grau de autonomia para decidir condutas no trabalho? (EAV 1-10)

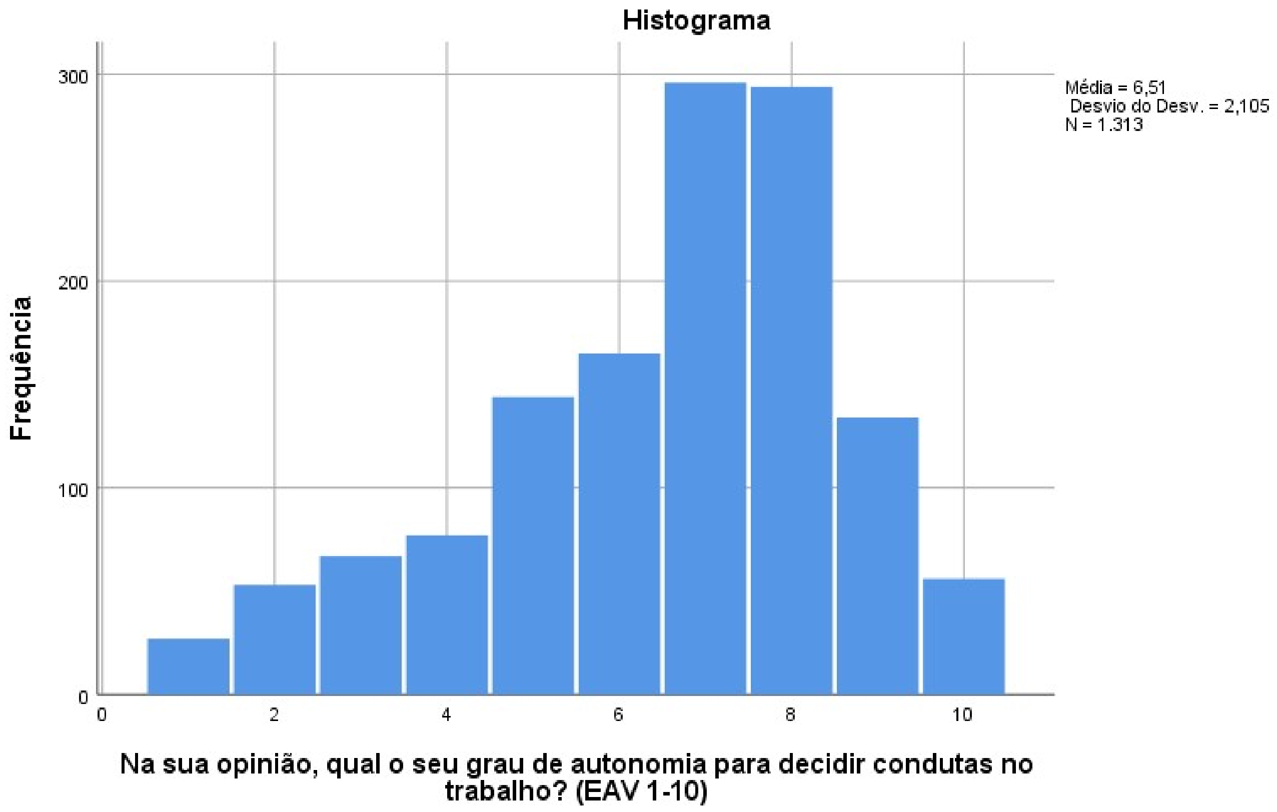

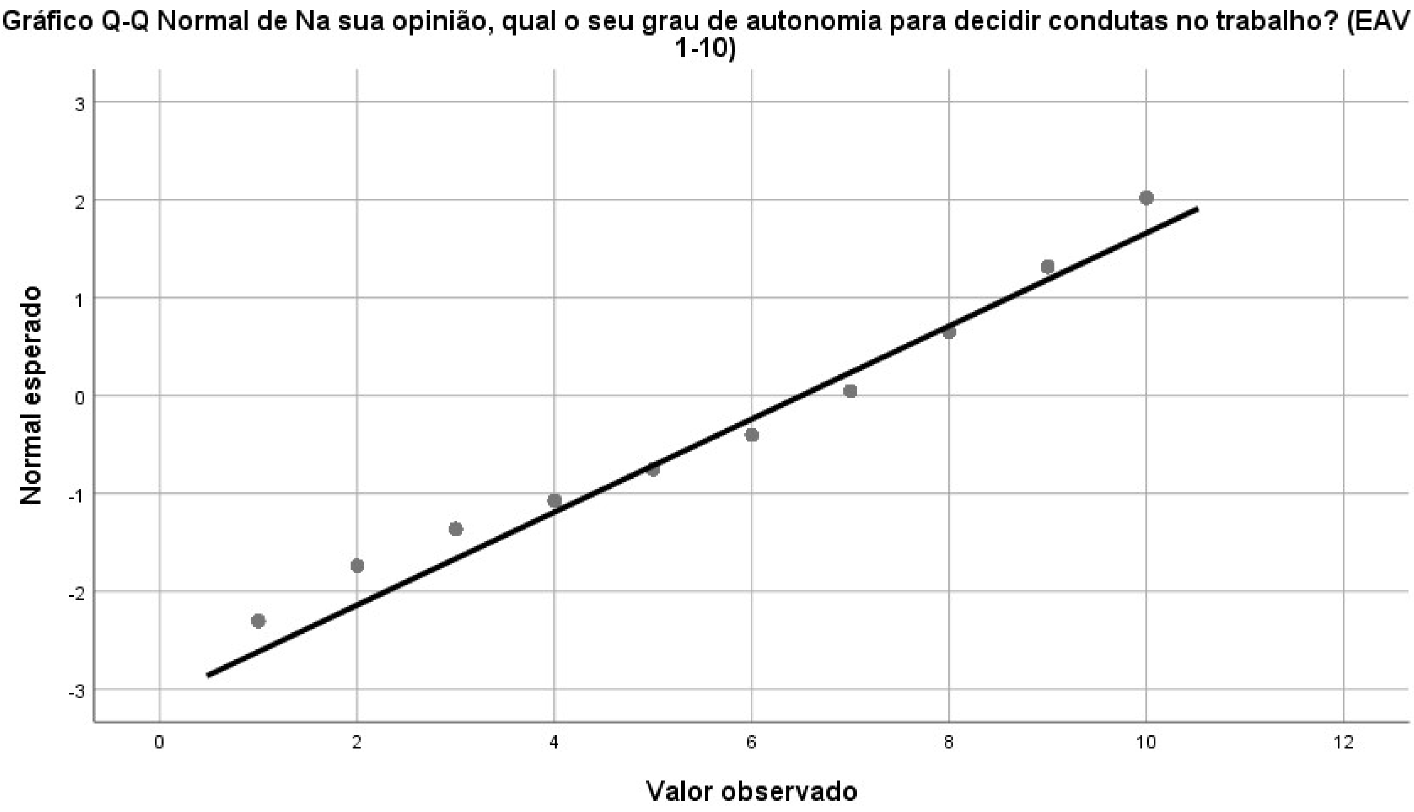

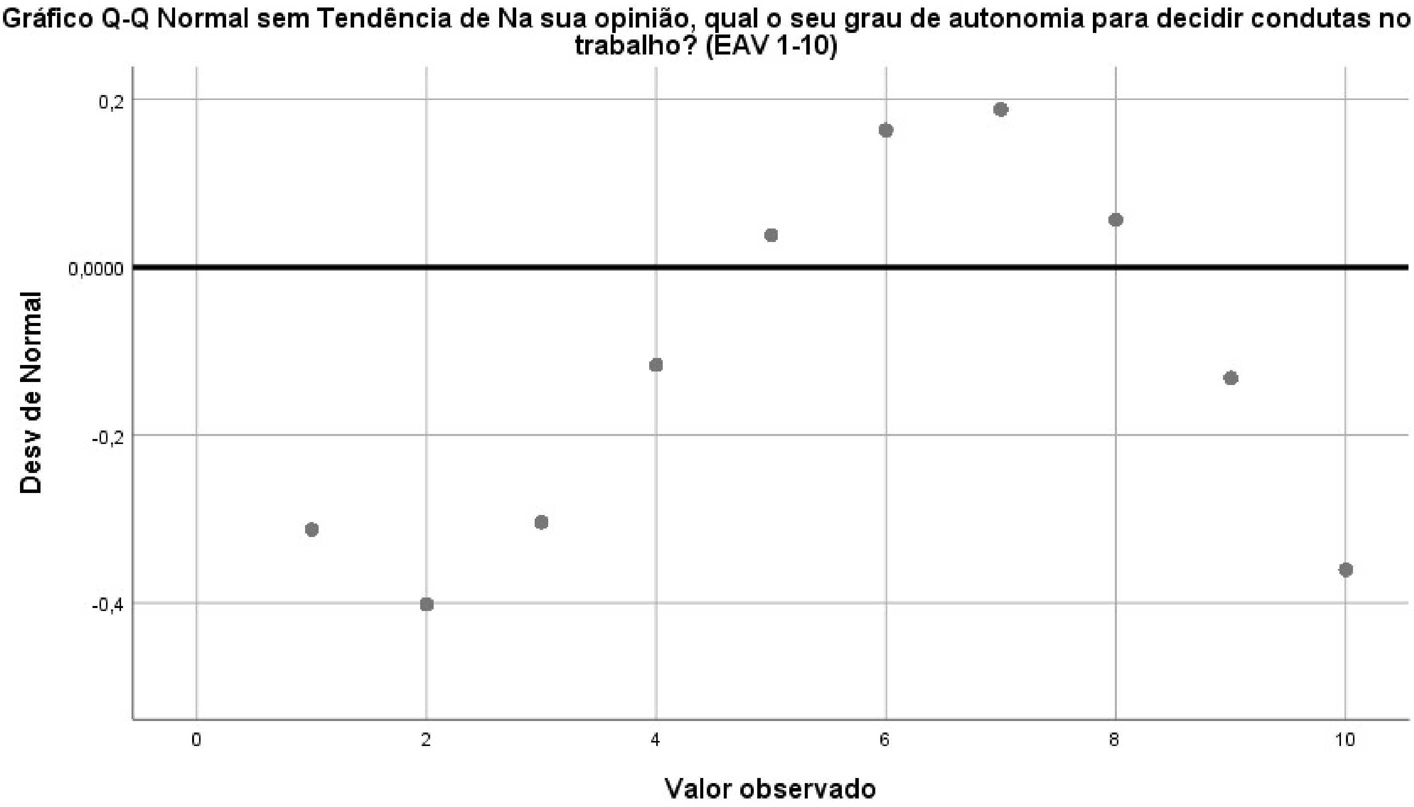

Na sua opinião, qual o grau de adequação da organização pedagógica de seu programa de residência profissional? (EAV 1-10)

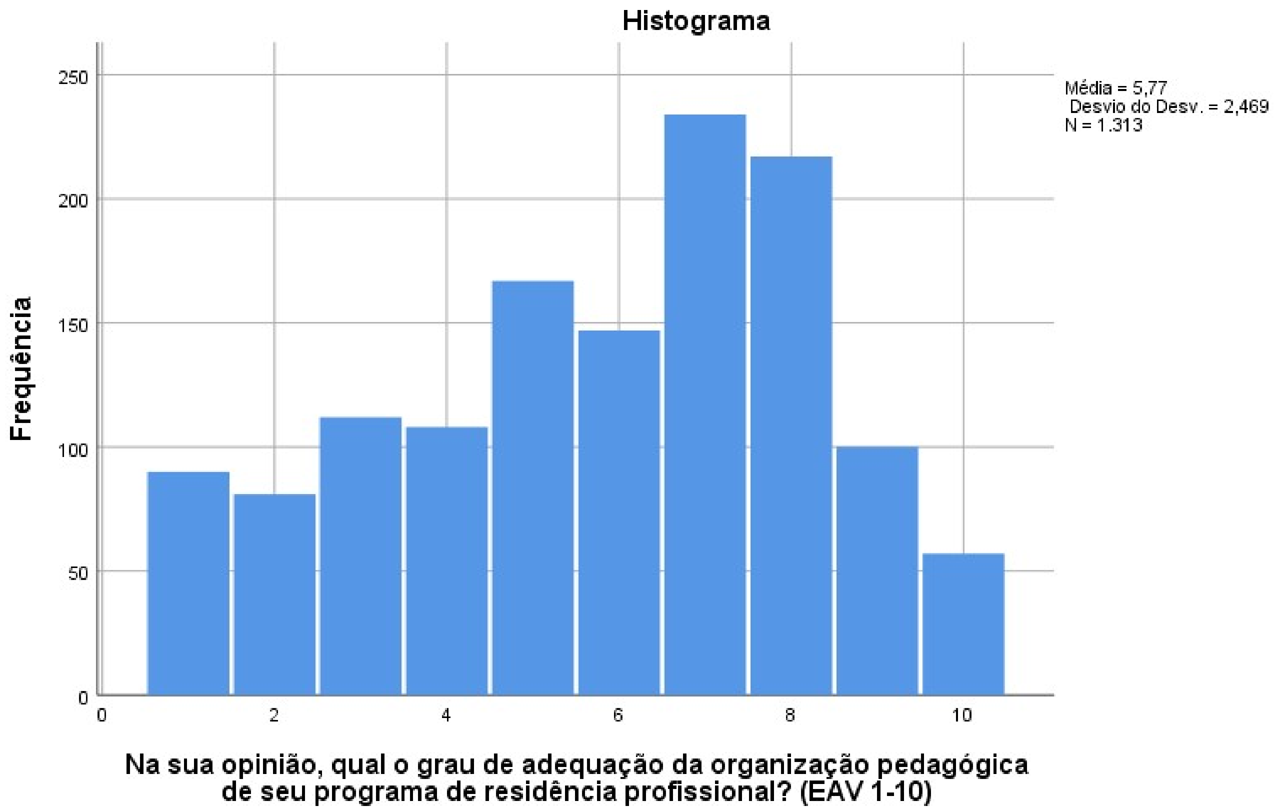
Histograma
Média = 5,77
Desvio do Desv. = 2,469
N = 1.313
Frequência
250
200
150
100
50
0
0
2
4
6
8
10
Na sua opinião, qual o grau de adequação da organização pedagógica de seu programa de residência profissional? (EAV 1-10)

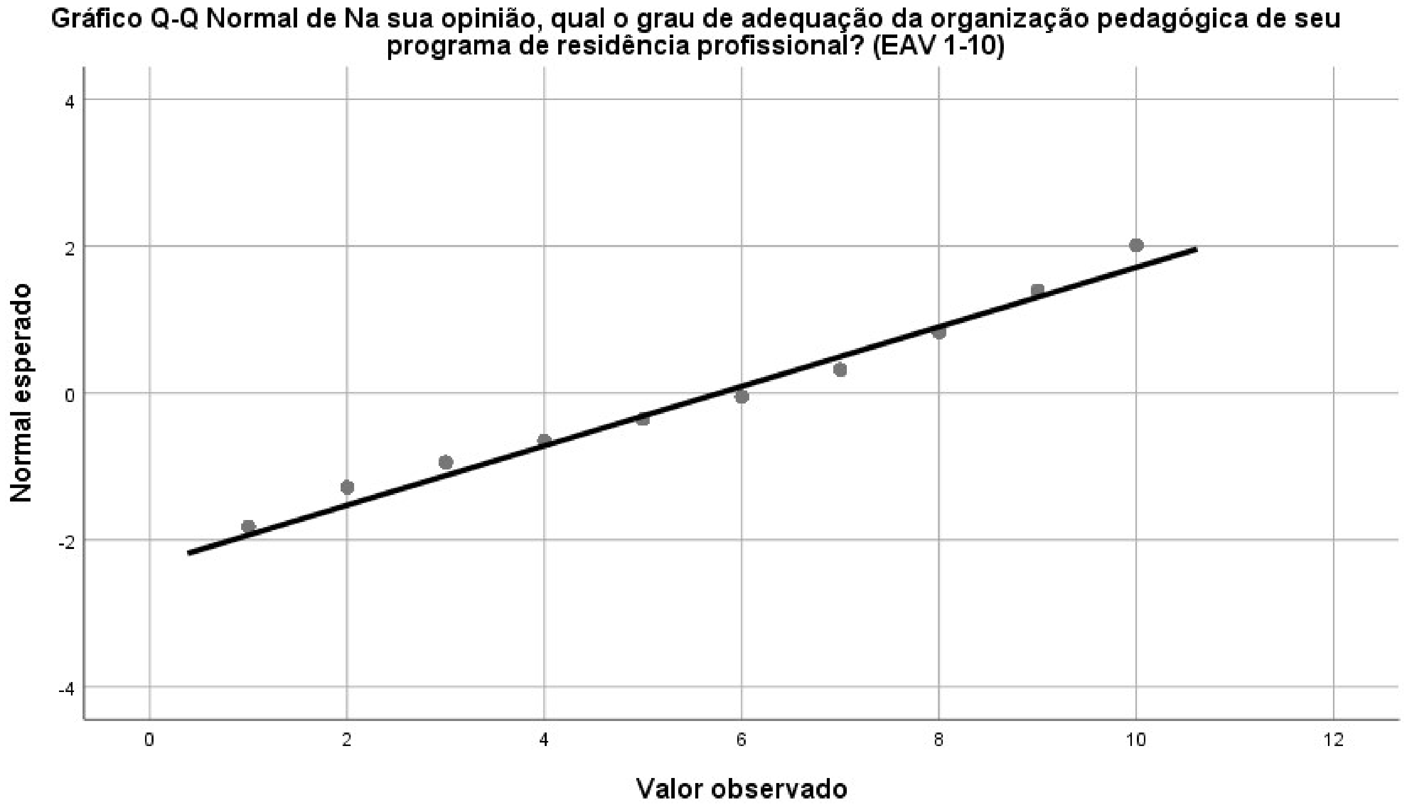
Gráfico Q-Q Normal de Na sua opinião, qual o grau de adequação da organização pedagógica de seu programa de residência profissional? (EAV 1-10)
Normal esperado
4
2
0
-2
-4
0
2
4
6
8
10
12
Valor observado

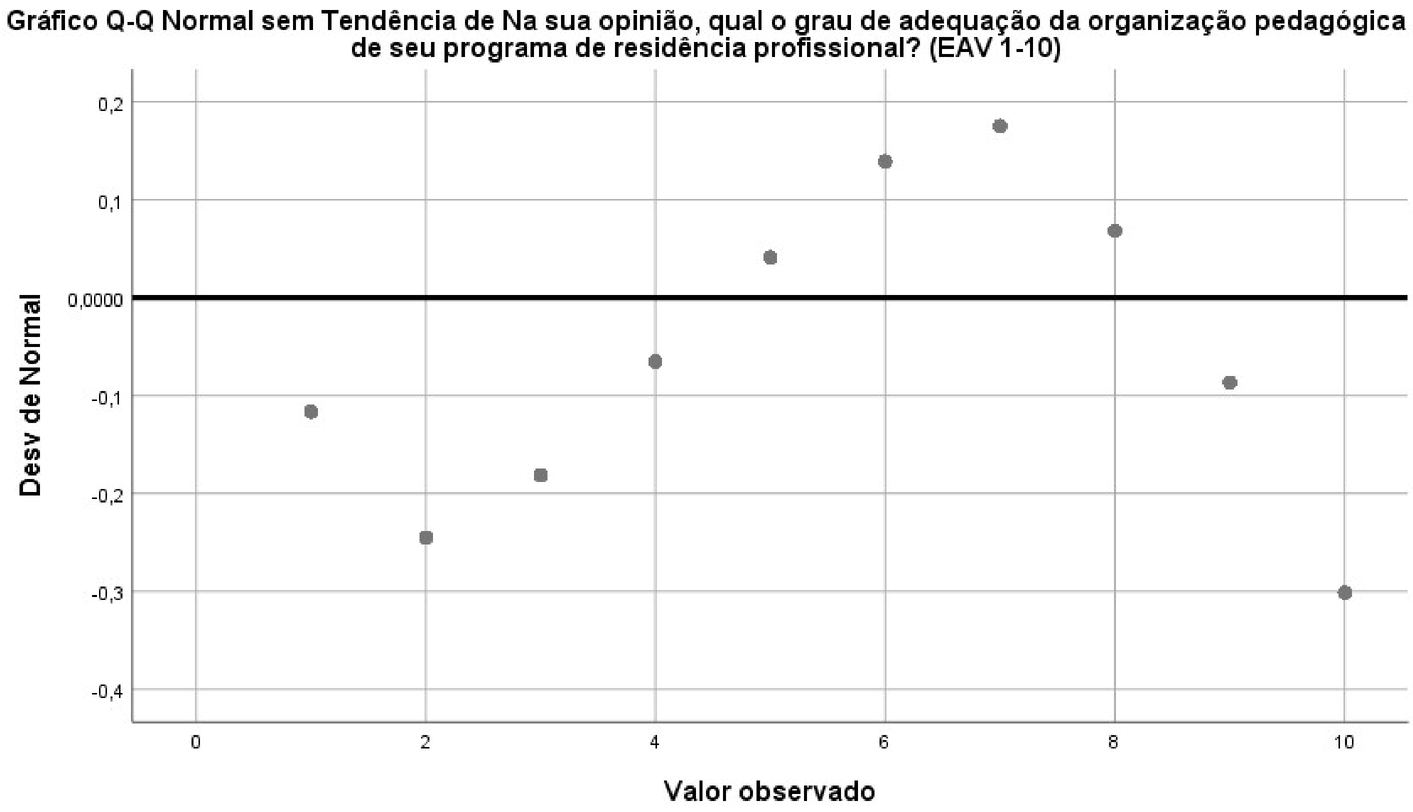


```
FREQUENCIES VARIABLES=Autonomy_Classif PedagStr_Classif
  /BARCHART PERCENT
  /ORDER=VARIABLE.
```

Frequências

Observações

Saída criada		20-SEP-2020 11:21:34
Comentários		
Entrada	Dados	C:\Users\User\Documents\Pesquisa\Fellow\FellowGenData_V1.sav
	Conjunto de dados ativo	ConjuntodeDados1
	Filtro	<none>
	Ponderação	<none>
	Arquivo Dividido	<none>
	N de linhas em arquivo de dados de trabalho	1313
Tratamento de valor omisso	Definição de omisso	Os valores omissos definidos pelo usuário são tratados como omissos.
	Casos utilizados	As estatísticas estão baseadas em todos os casos com dados válidos.

Sintaxe		FREQUENCIES VARIABLES=Autonomy_Classif PedagStr_Classif<br>/BARCHART PERCENT<br>/ORDER=VARIABLE.
Recursos	Tempo do processador	00:00:00,36
	Tempo decorrido	00:00:00,26

```
Classification of the percieved autonomy to self-conduct in the residency program
```

Estatísticas

Classification of the percieved autonomy to self-conduct in the residency program

N	Válido	1313
	Omisso	0

Classification of the percieved autonomy to self-conduct in the residency program

		Frequência	Porcentagem	Porcentagem válida	Porcentagem acumulativa
Válido	Low autonomy	224	17,1	17,1	17,1
	Moderate to high autonomy	1089	82,9	82,9	100,0
	Total	1313	100,0	100,0	

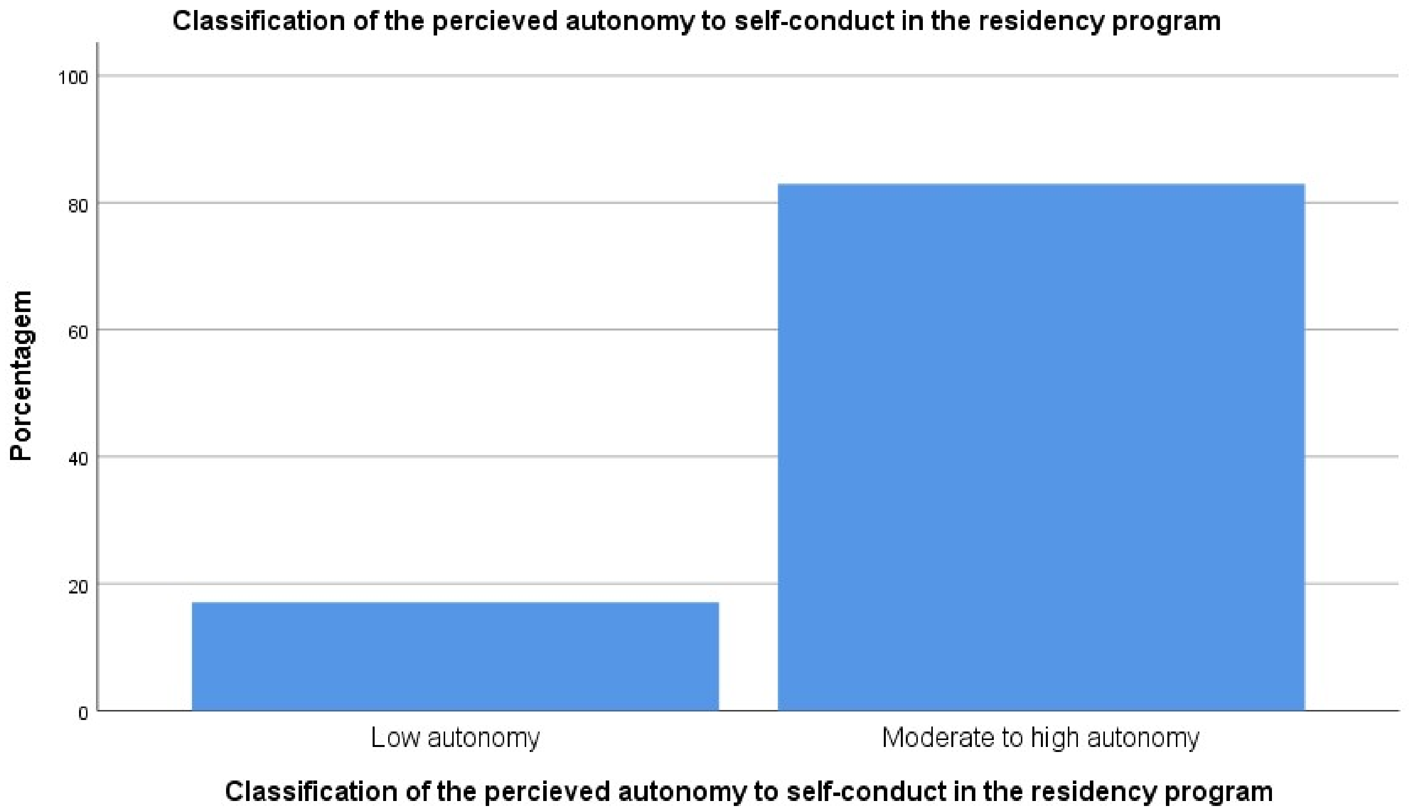

Percieved adequacy of the pedagogic structure and availability of resouces in the residency program

Estatísticas

Percieved adequacy of the pedagogic structure and availability of resouces in the residency program

N	Válido	1313
	Omisso	0

Percieved adequacy of the pedagogic structure and availability of resouces in the residency program

		Frequência	Porcentagem	Porcentagem válida	Porcentagem acumulativa
Válido	Poor adequacy	558	42,5	42,5	42,5
	Moderate to good adequacy	755	57,5	57,5	100,0
	Total	1313	100,0	100,0	

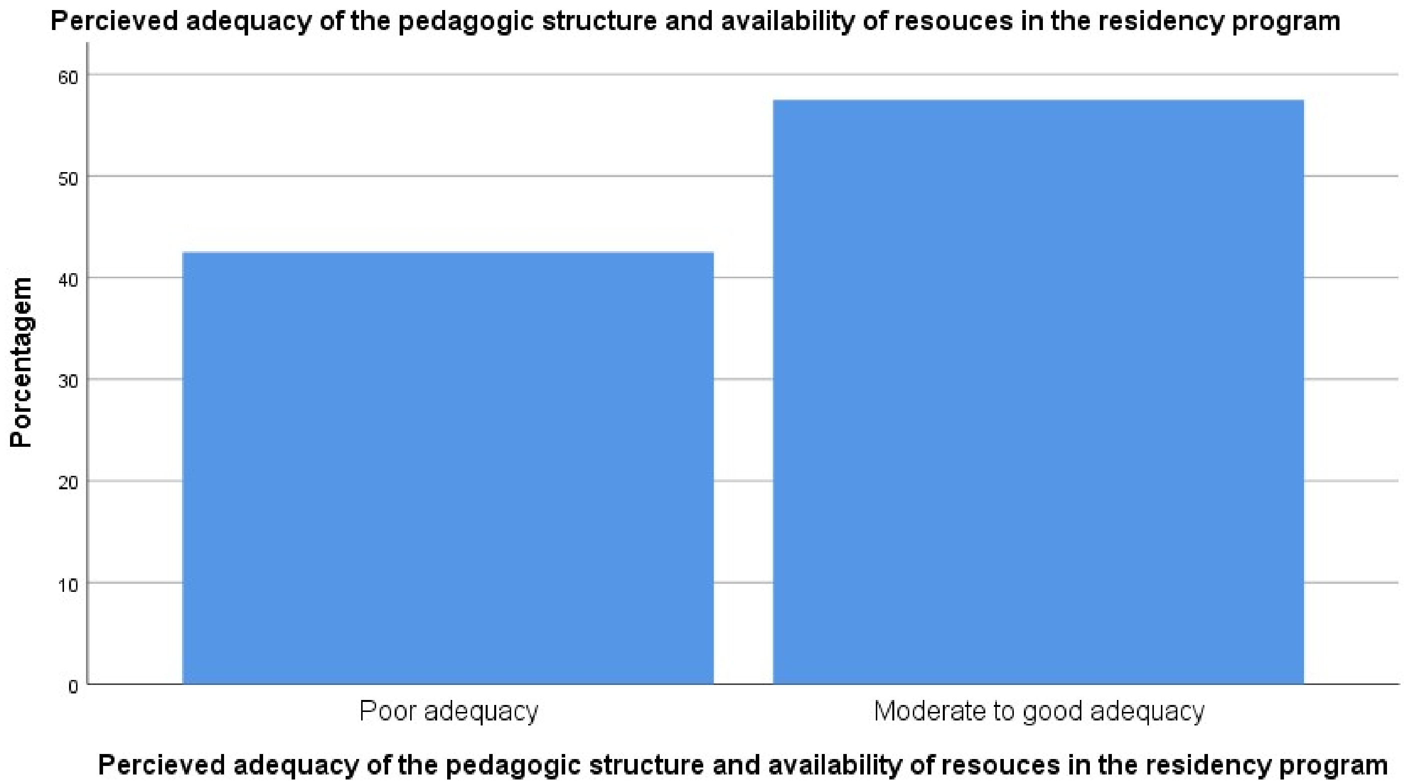


```
EXAMINE VARIABLES=DASS21_D DASS21_A DASS21_S PHQ9_Score BRCS_Score OLBI_D OLBI_E OLBI_Total
  /PLOT HISTOGRAM NPPLOT
  /STATISTICS DESCRIPTIVES
  /CINTERVAL 95
  /MISSING PAIRWISE
  /NOTOTAL.
```

Explorar

Observações

Saída criada		20-SEP-2020 11:23:59
Comentários		
Entrada	Dados	C:\Users\User\Documents\Pesquisa\Fellow\FellowGenData_V1.sav
	Conjunto de dados ativo	ConjuntodeDados1
	Filtro	<none>
	Ponderação	<none>
	Arquivo Dividido	<none>
	N de linhas em arquivo de dados de trabalho	1313

Tratamento de valor omisso	Definição de omisso	Os valores omissos definidos pelo usuário para variáveis dependentes são tratados como omissos.
	Casos utilizados	As estatísticas são baseadas em casos sem valores omissos para a variável dependente ou fatores que estão sendo analisados.
Sintaxe		EXAMINE VARIABLES=DASS21_D DASS21_A DASS21_S PHQ9_Score BRCS_Score OLBI_D OLBI_E OLBI_Total /PLOT HISTOGRAM NPPLOT /STATISTICS DESCRIPTIVES /CINTERVAL 95 /MISSING PAIRWISE /NOTOTAL.
Recursos	Tempo do processador	00:00:04,08
	Tempo decorrido	00:00:03,87

Resumo de processamento do caso

	Casos					
	Válido		Omisso		Total	
	N	Porcentagem	N	Porcentagem	N	Porcentagem
DASS21 Depression Score	1313	100,0%	0	0,0%	1313	100,0%
DASS21 Anxietty Score	1313	100,0%	0	0,0%	1313	100,0%
DASS21 Stress Score	1313	100,0%	0	0,0%	1313	100,0%
PHQ9 Depression Score	1313	100,0%	0	0,0%	1313	100,0%
BRCS Score	1313	100,0%	0	0,0%	1313	100,0%
OLBI Disengagement Score	1313	100,0%	0	0,0%	1313	100,0%
OLBI Exhaustion Score	1313	100,0%	0	0,0%	1313	100,0%
OLBI Total Score	1313	100,0%	0	0,0%	1313	100,0%

Descritivos

			Estatística	Erro Erro
DASS21 Depression Score	Média		7,64	,156
	95% Intervalo de Confiança para Média	Limite inferior	7,34	
		Limite superior	7,95	
	5% da média aparada		7,38	
	Mediana		7,00	
	Variância		31,985	
	Erro Desvio		5,656	
	Mínimo		0	

	Máximo		21	
	Intervalo		21	
	Amplitude interquartil		8	
	Assimetria		,611	,068
	Curtose		-,520	,135
DASS21 Anxietty Score	Média		6,03	,142
	95% Intervalo de Confiança para Média	Limite inferior	5,75	
		Limite superior	6,31	
	5% da média aparada		5,66	
	Mediana		5,00	
	Variância		26,570	
	Erro Desvio		5,155	
	Mínimo		0	
	Máximo		21	
	Intervalo		21	
	Amplitude interquartil		7	
	Assimetria		,829	,068
	Curtose		,022	,135
DASS21 Stress Score	Média		10,15	,148
	95% Intervalo de Confiança para Média	Limite inferior	9,86	
		Limite superior	10,44	
	5% da média aparada		10,12	
	Mediana		10,00	
	Variância		28,883	
	Erro Desvio		5,374	
	Mínimo		0	
	Máximo		21	
	Intervalo		21	
	Amplitude interquartil		8	
	Assimetria		,107	,068
	Curtose		-,771	,135
PHQ9 Depression Score	Média		12,00	,178
	95% Intervalo de Confiança para Média	Limite inferior	11,65	
		Limite superior	12,35	
	5% da média aparada		11,91	
	Mediana		12,00	
	Variância		41,628	
	Erro Desvio		6,452	
	Mínimo		0	
	Máximo		27	
	Intervalo		27	
	Amplitude interquartil		10	
	Assimetria		,188	,068
	Curtose		-,753	,135
BRCS Score	Média		12,41	,104
	95% Intervalo de Confiança para	Limite inferior	12,21	

	Média	Limite superior	12,62	
	5% da média aparada		12,42	
	Mediana		12,00	
	Variância		14,244	
	Erro Desvio		3,774	
	Mínimo		4	
	Máximo		20	
	Intervalo		16	
	Amplitude interquartil		5	
	Assimetria		,036	,068
	Curtose		-,613	,135
OLBI Disengagement Score	Média		2,7740	,02317
	95% Intervalo de Confiança para Média	Limite inferior	2,7285	
		Limite superior	2,8194	
	5% da média aparada		2,7632	
	Mediana		2,7500	
	Variância		,705	
	Erro Desvio		,83949	
	Mínimo		1,00	
	Máximo		5,00	
	Intervalo		4,00	
	Amplitude interquartil		1,25	
	Assimetria		,186	,068
	Curtose		-,428	,135
OLBI Exhaustion Score	Média		3,5546	,02032
	95% Intervalo de Confiança para Média	Limite inferior	3,5147	
		Limite superior	3,5945	
	5% da média aparada		3,5786	
	Mediana		3,6300	
	Variância		,542	
	Erro Desvio		,73625	
	Mínimo		1,00	
	Máximo		5,00	
	Intervalo		4,00	
	Amplitude interquartil		,87	
	Assimetria		-,525	,068
	Curtose		,223	,135
OLBI Total Score	Média		3,1644	,01931
	95% Intervalo de Confiança para Média	Limite inferior	3,1265	
		Limite superior	3,2022	
	5% da média aparada		3,1721	
	Mediana		3,1900	
	Variância		,490	
	Erro Desvio		,69979	
	Mínimo		1,00	
	Máximo		5,00	

	Intervalo	4,00	
	Amplitude interquartil	,94	
	Assimetria	-,135	,068
	Curtose	-,231	,135

Testes de Normalidade

	Kolmogorov-Smirnov[a]			Shapiro-Wilk		
	Estatística	df	Sig.	Estatística	df	Sig.
DASS21 Depression Score	,109	1313	,000	,939	1313	,000
DASS21 Anxietty Score	,122	1313	,000	,917	1313	,000
DASS21 Stress Score	,075	1313	,000	,979	1313	,000
PHQ9 Depression Score	,070	1313	,000	,981	1313	,000
BRCS Score	,065	1313	,000	,983	1313	,000
OLBI Disengagement Score	,056	1313	,000	,990	1313	,000
OLBI Exhaustion Score	,067	1313	,000	,980	1313	,000
OLBI Total Score	,026	1313	,032	,996	1313	,003

a. Correlação de Significância de Lilliefors

DASS21 Depression Score

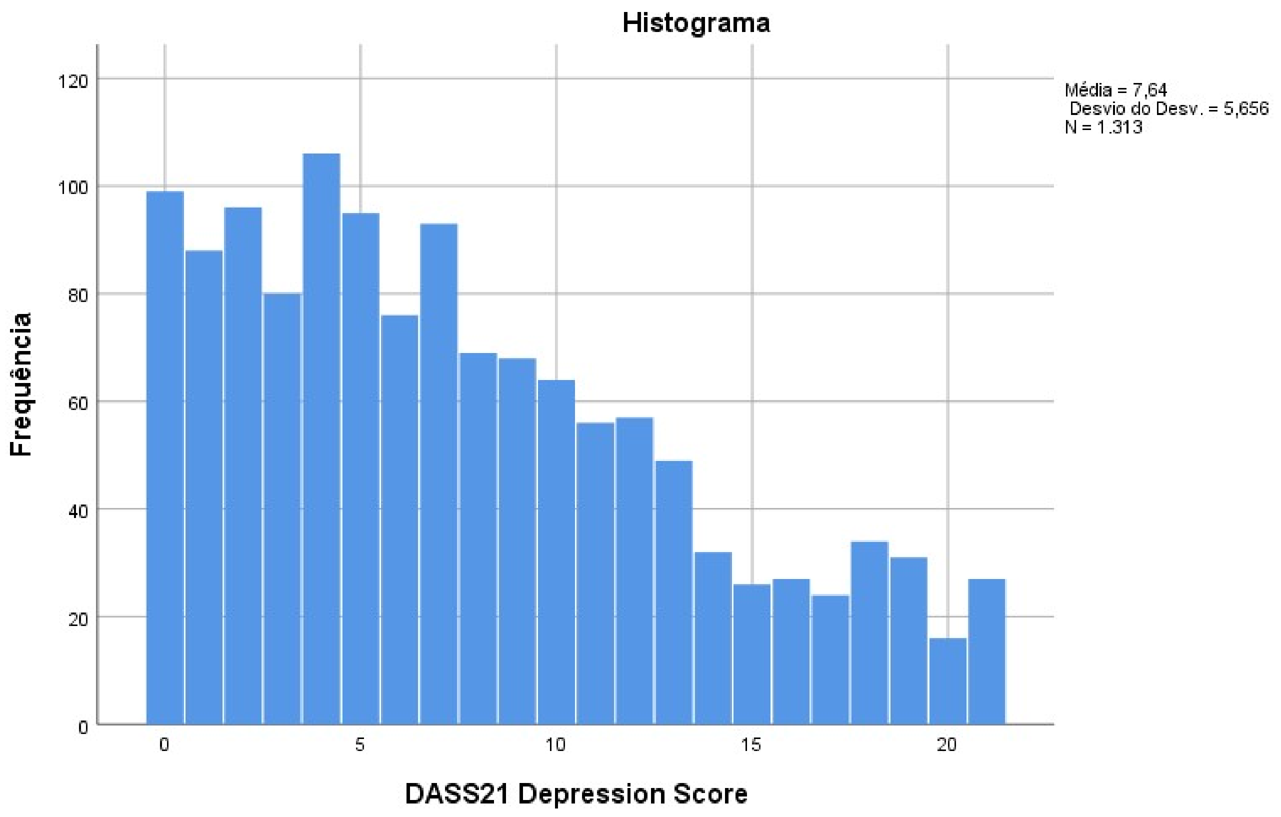

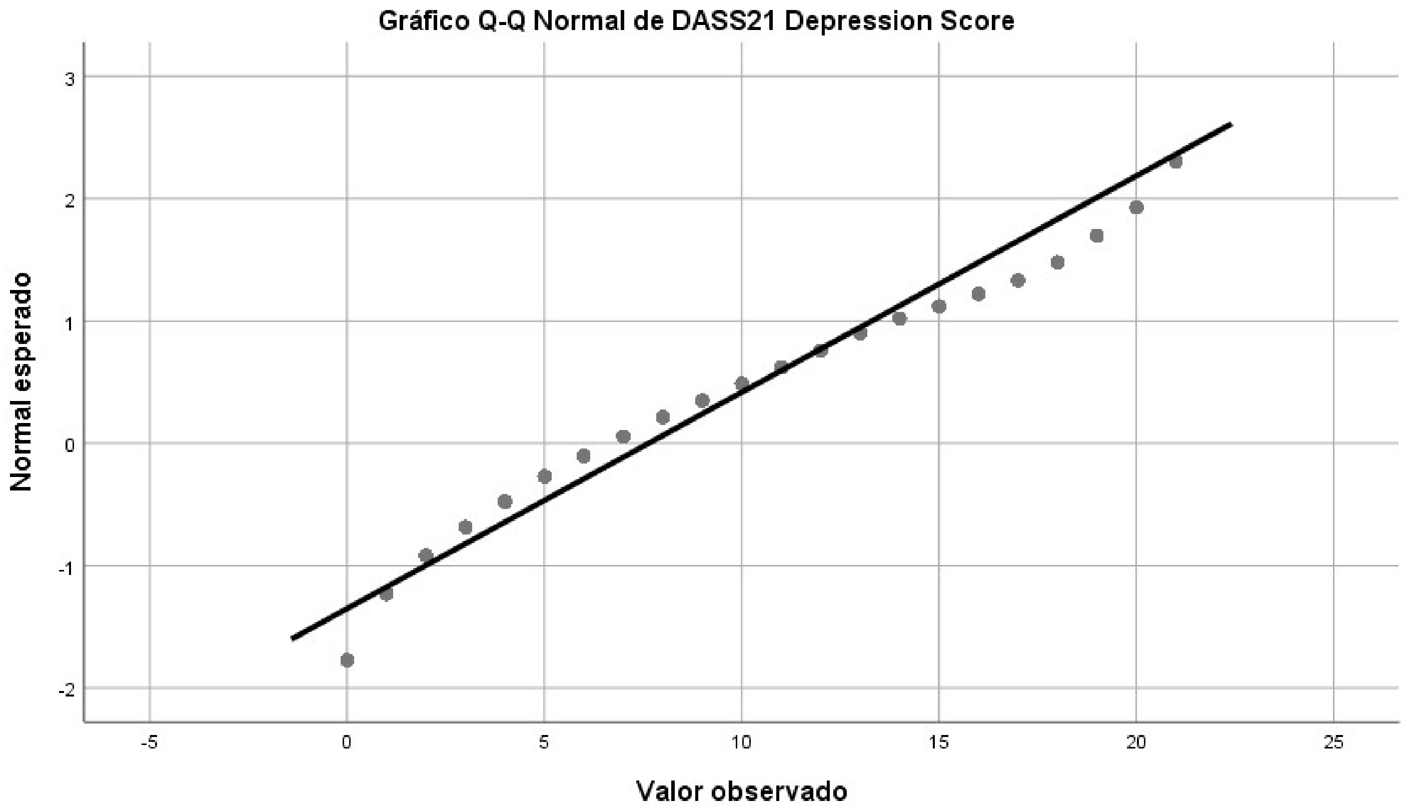

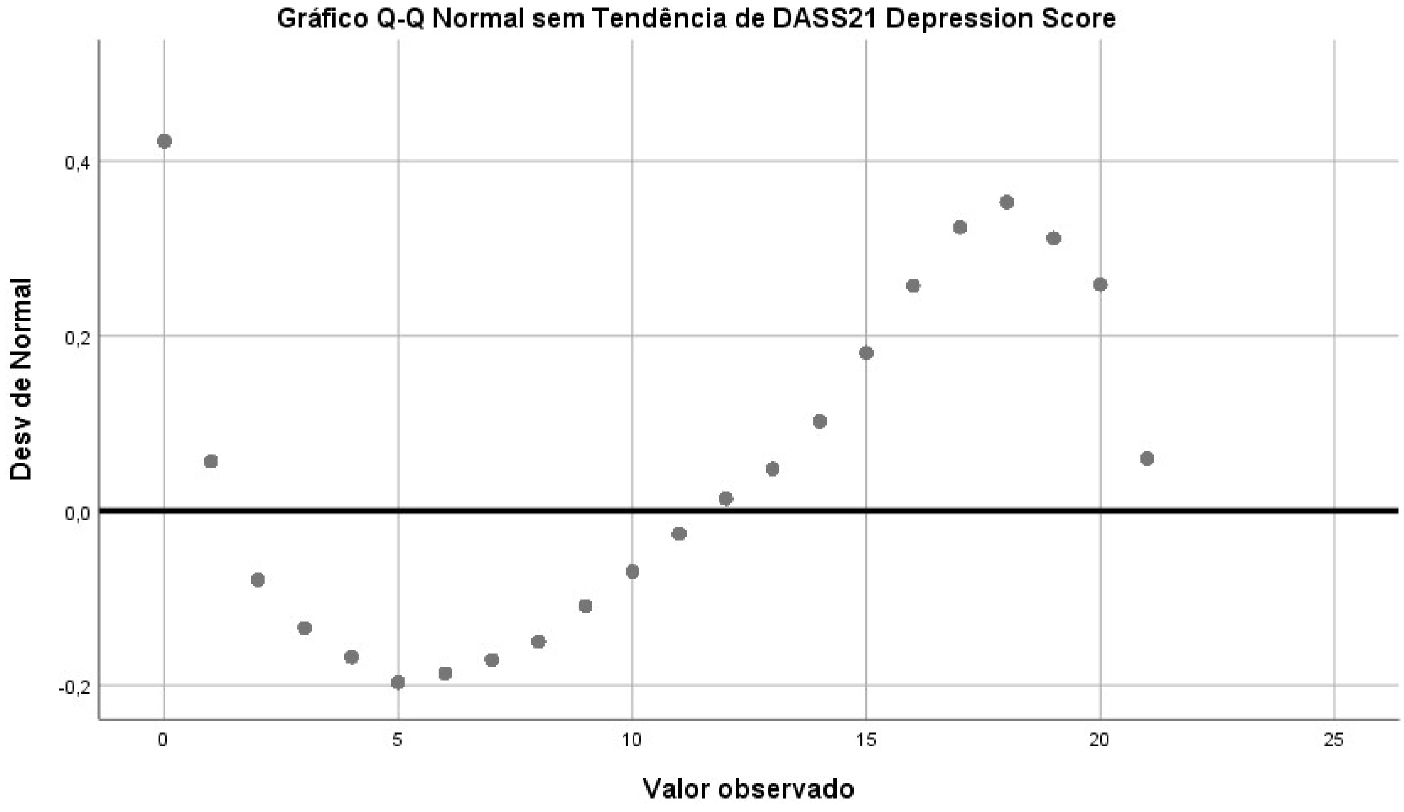

DASS21 Anxietty Score

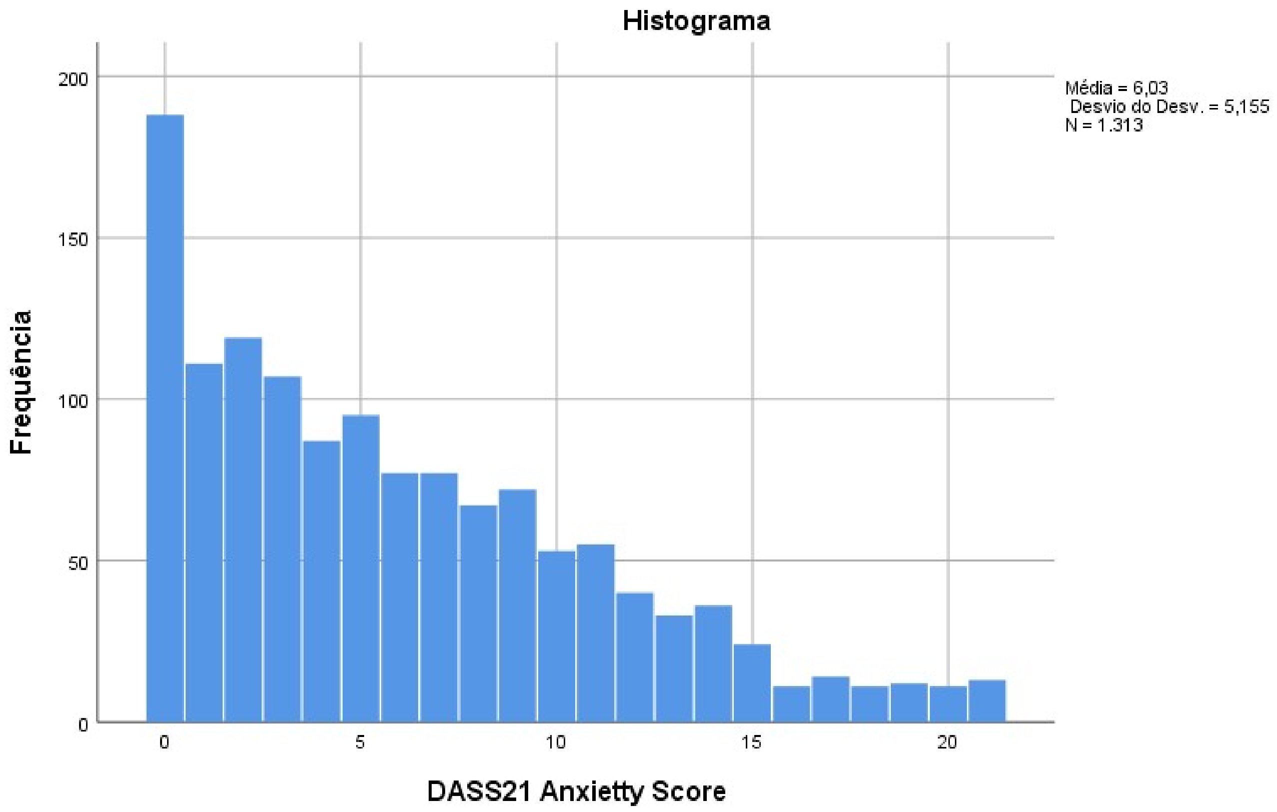

Histograma
Média = 6,03
Desvio do Desv. = 5,155
N = 1.313
Frequência
0
50
100
150
200
0
5
10
15
20
DASS21 Anxietty Score

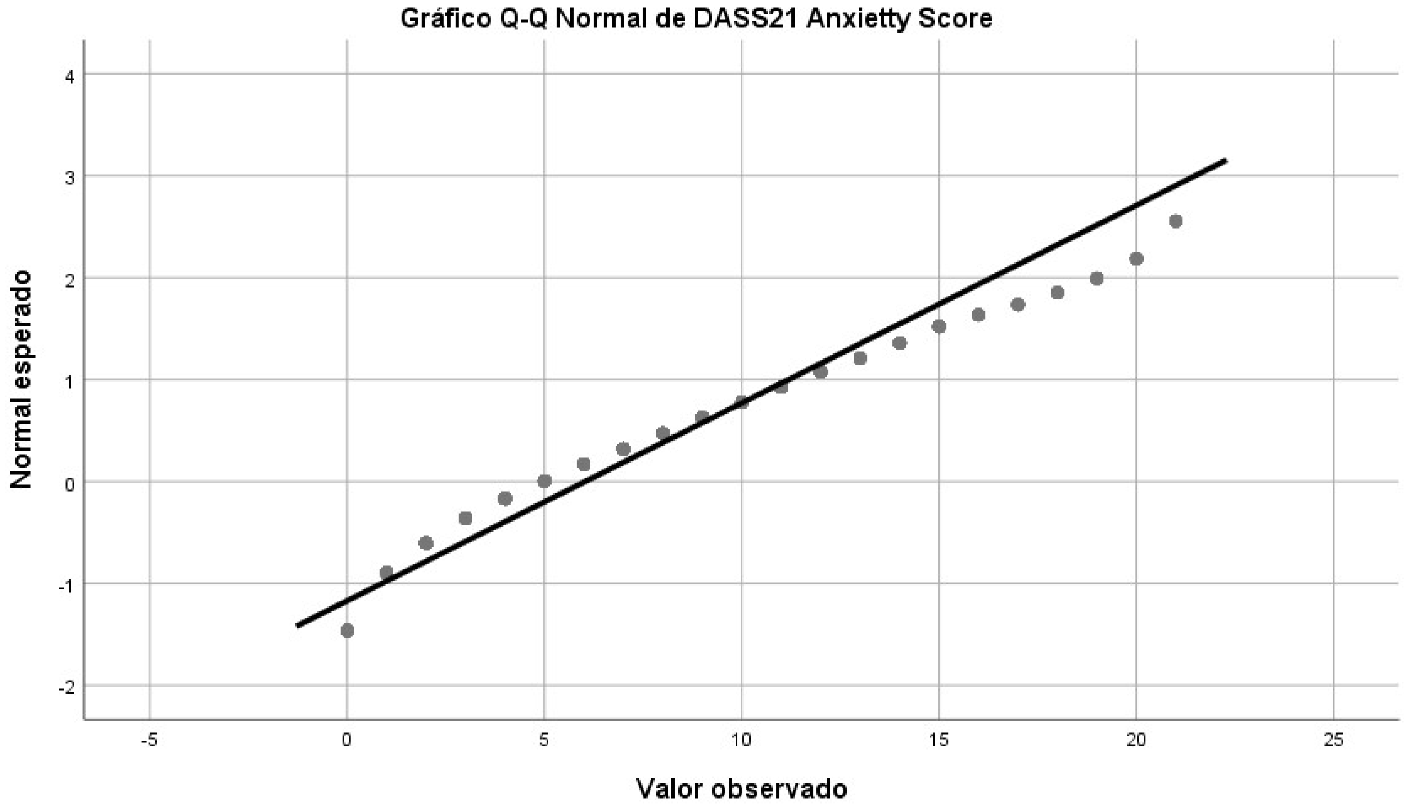

Gráfico Q-Q Normal de DASS21 Anxietty Score
Normal esperado
-2
-1
0
1
2
3
4
-5
0
5
10
15
20
25
Valor observado

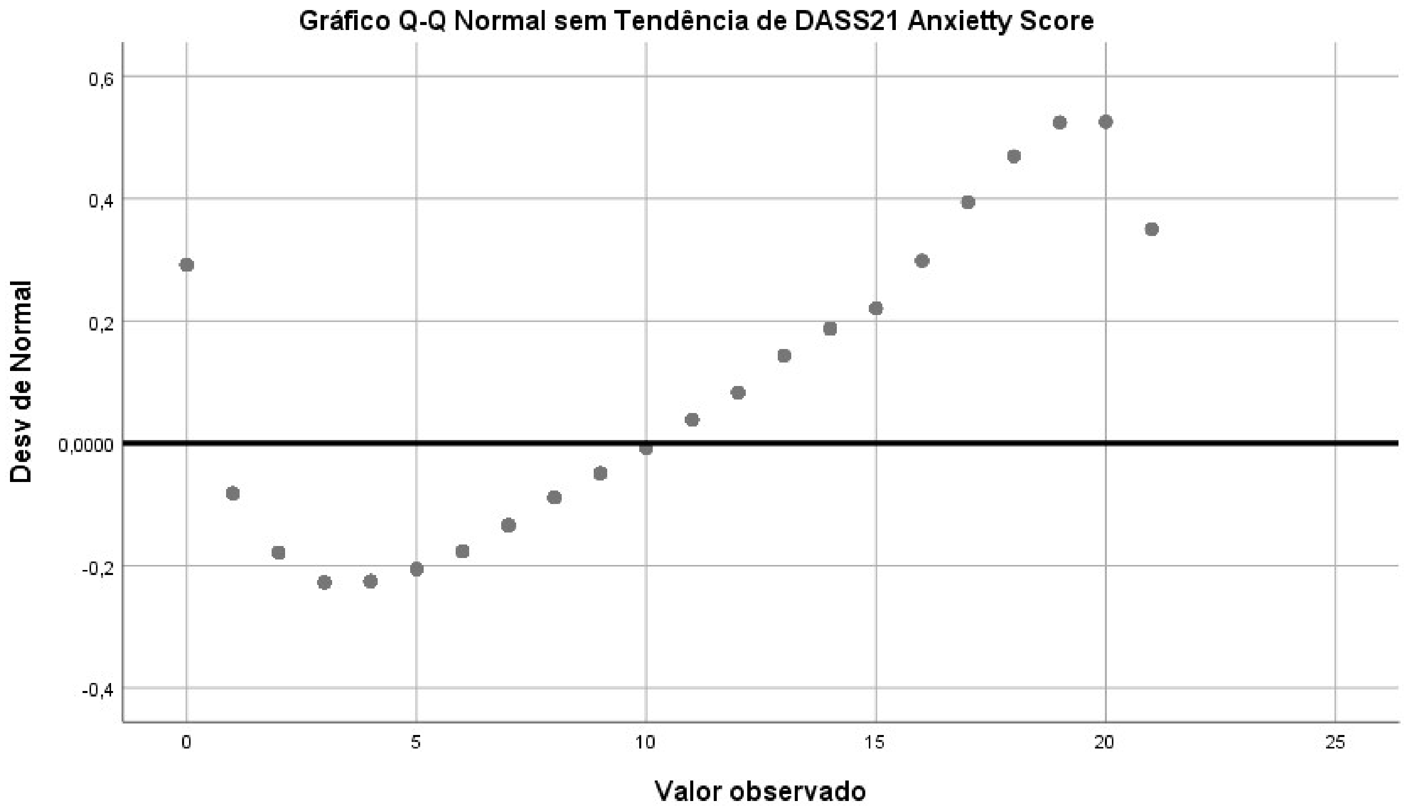

DASS21 Stress Score

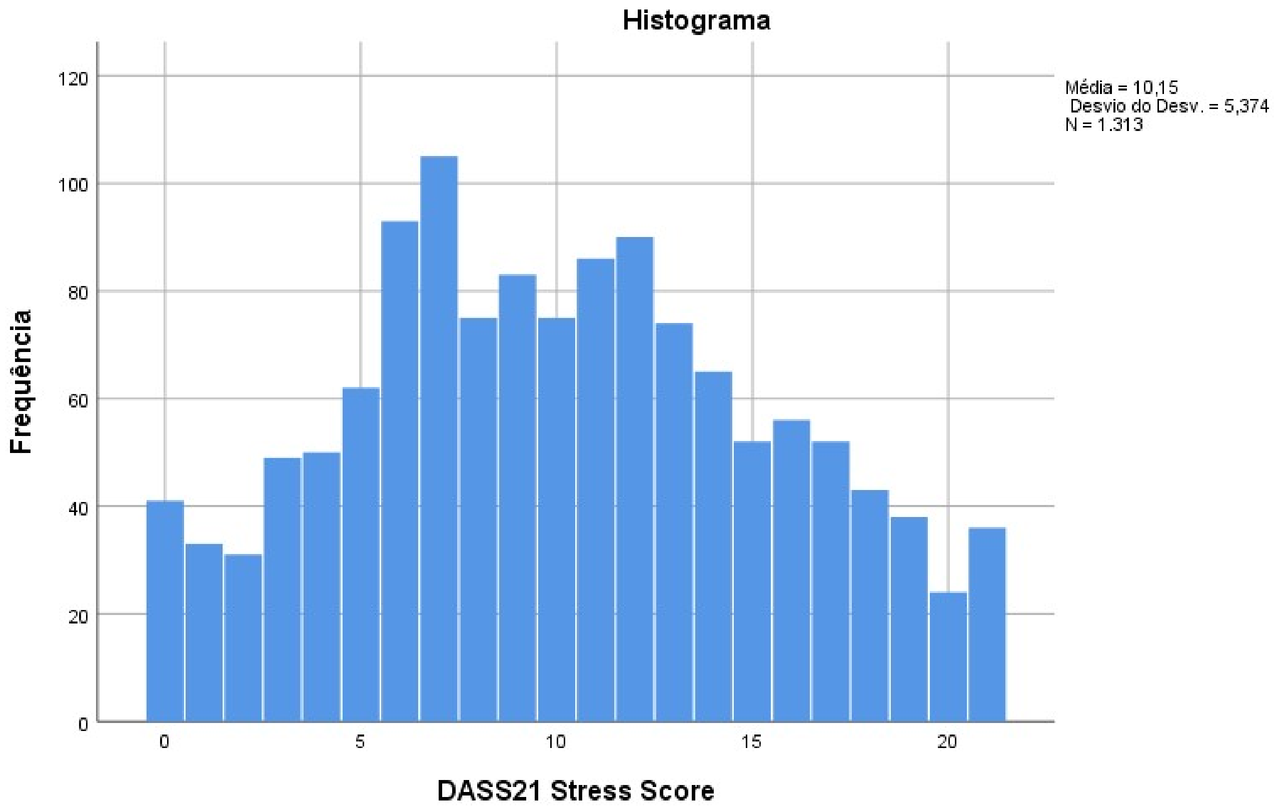

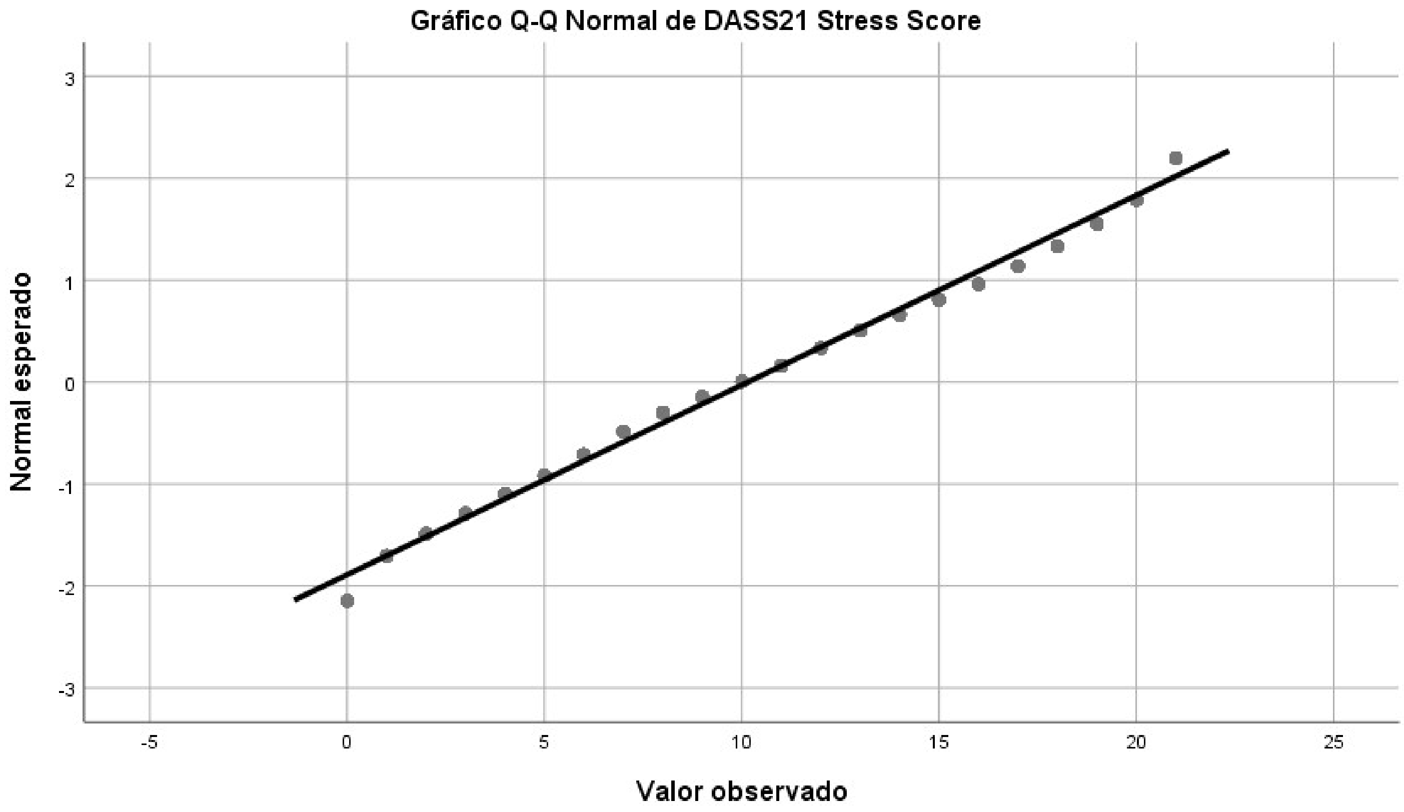

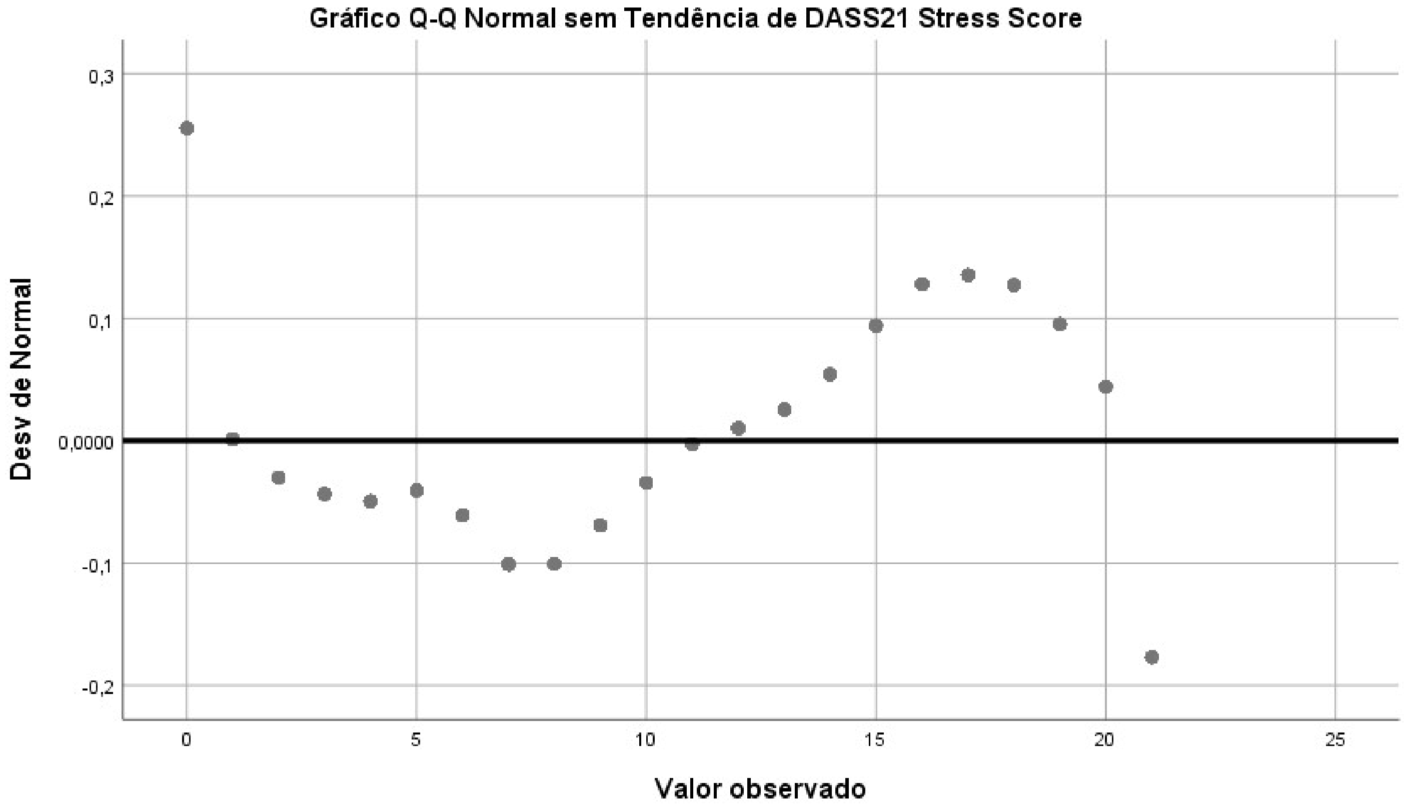

PHQ9 Depression Score

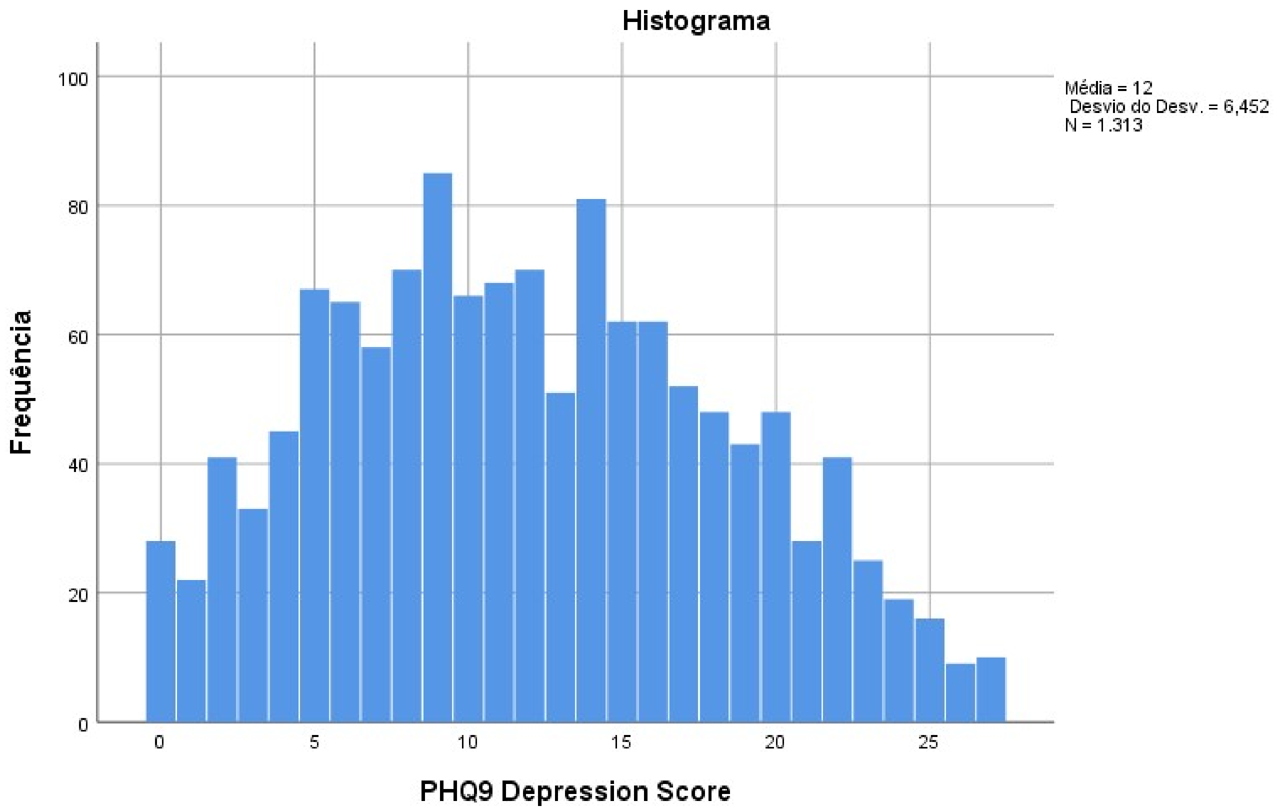
Histograma
Média = 12
Desvio do Desv. = 6,452
N = 1.313
Frequência
100
80
60
40
20
0
0
5
10
15
20
25
PHQ9 Depression Score

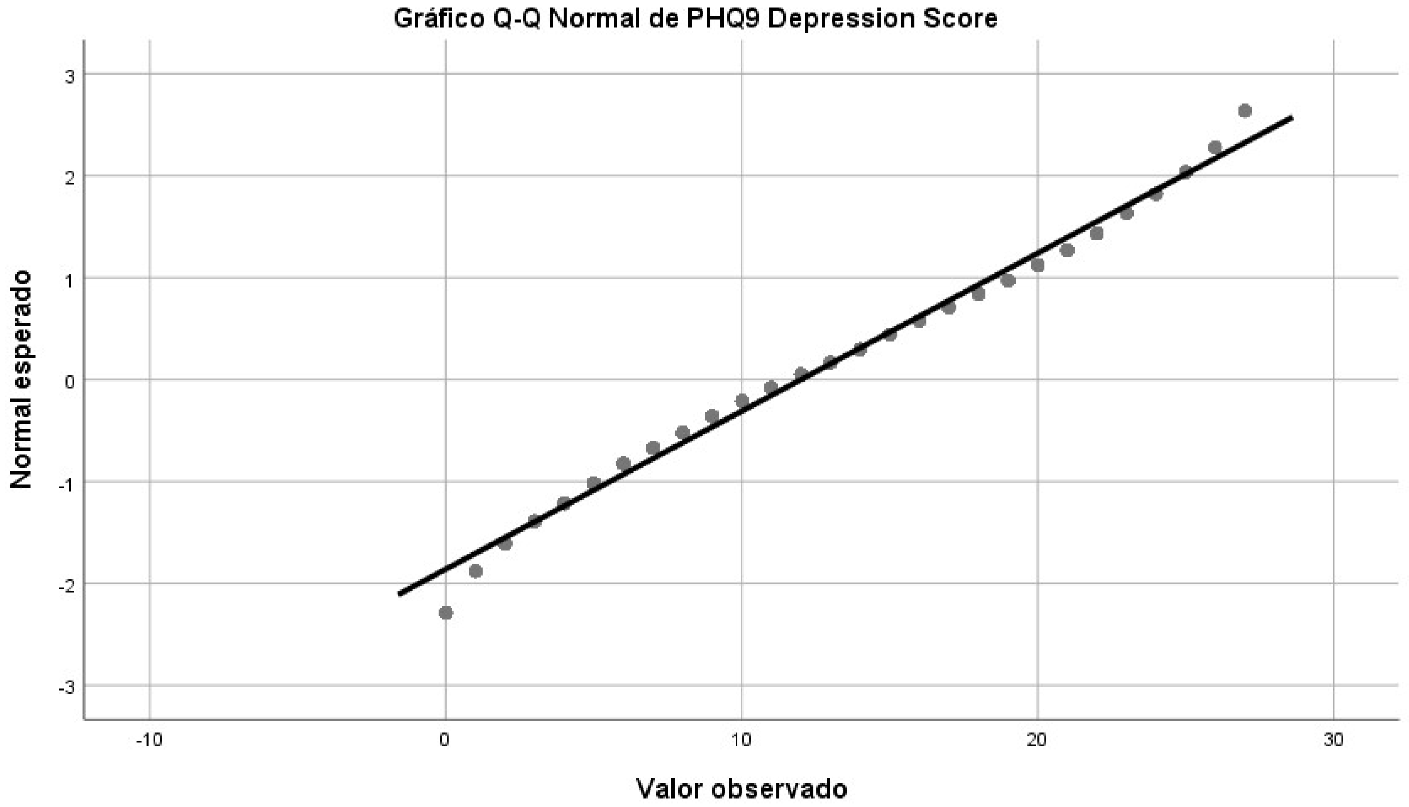
Gráfico Q-Q Normal de PHQ9 Depression Score
Normal esperado
3
2
1
0
-1
-2
-3
-10
0
10
20
30
Valor observado

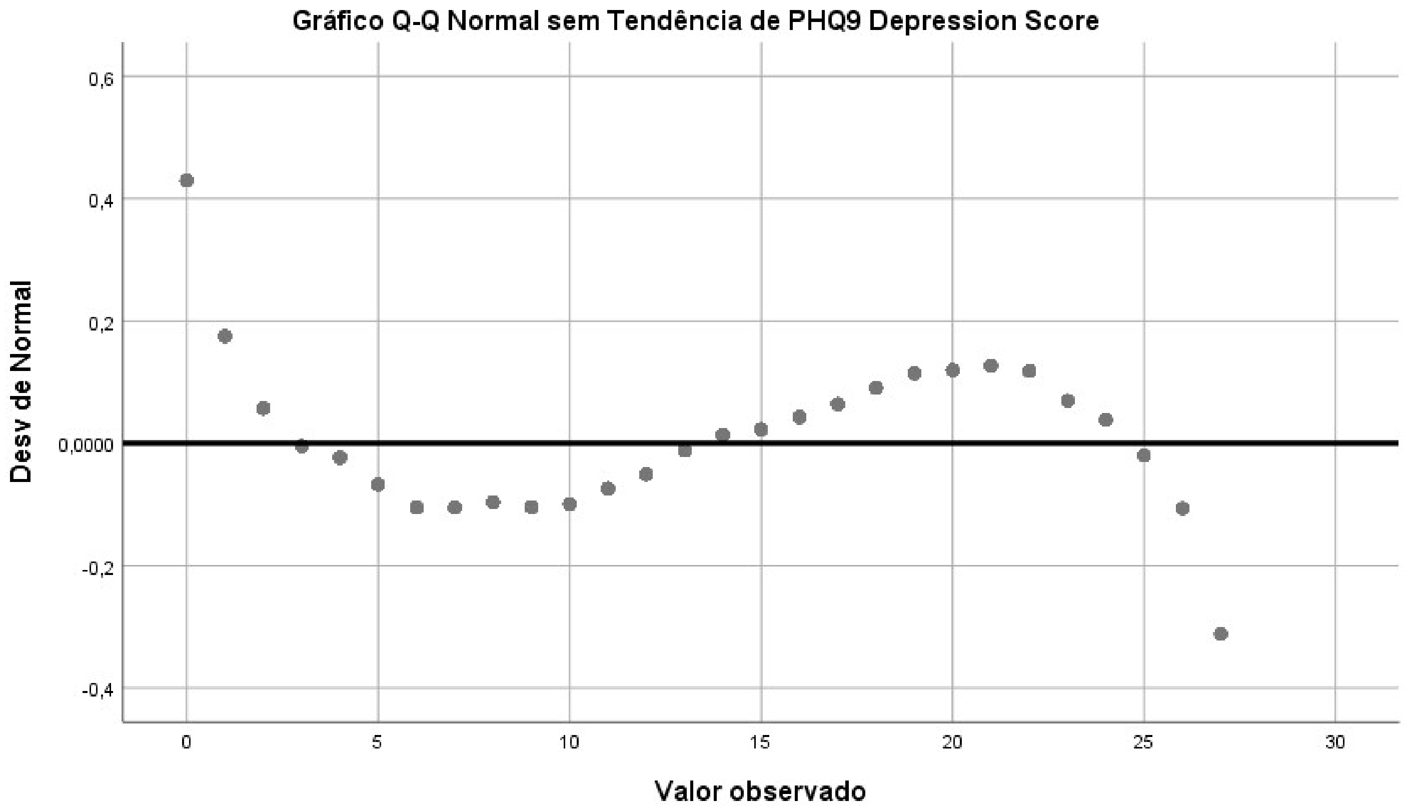
Gráfico Q-Q Normal sem Tendência de PHQ9 Depression Score
0,6
0,4
0,2
0,0000
-0,2
-0,4
Desv de Normal
0
5
10
15
20
25
30
Valor observado

BRCS Score

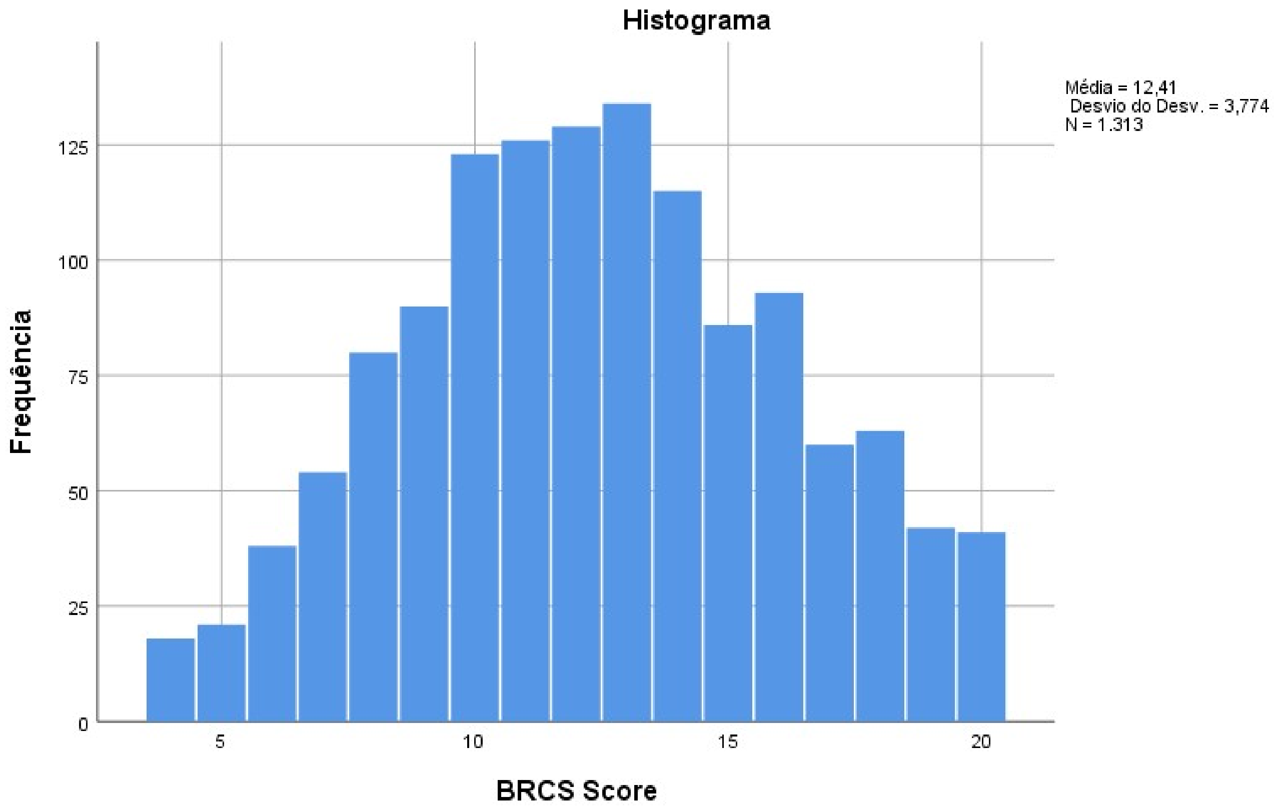
Histograma
Média = 12,41
Desvio do Desv. = 3,774
N = 1.313
125
100
75
50
25
0
Frequência
5
10
15
20
BRCS Score

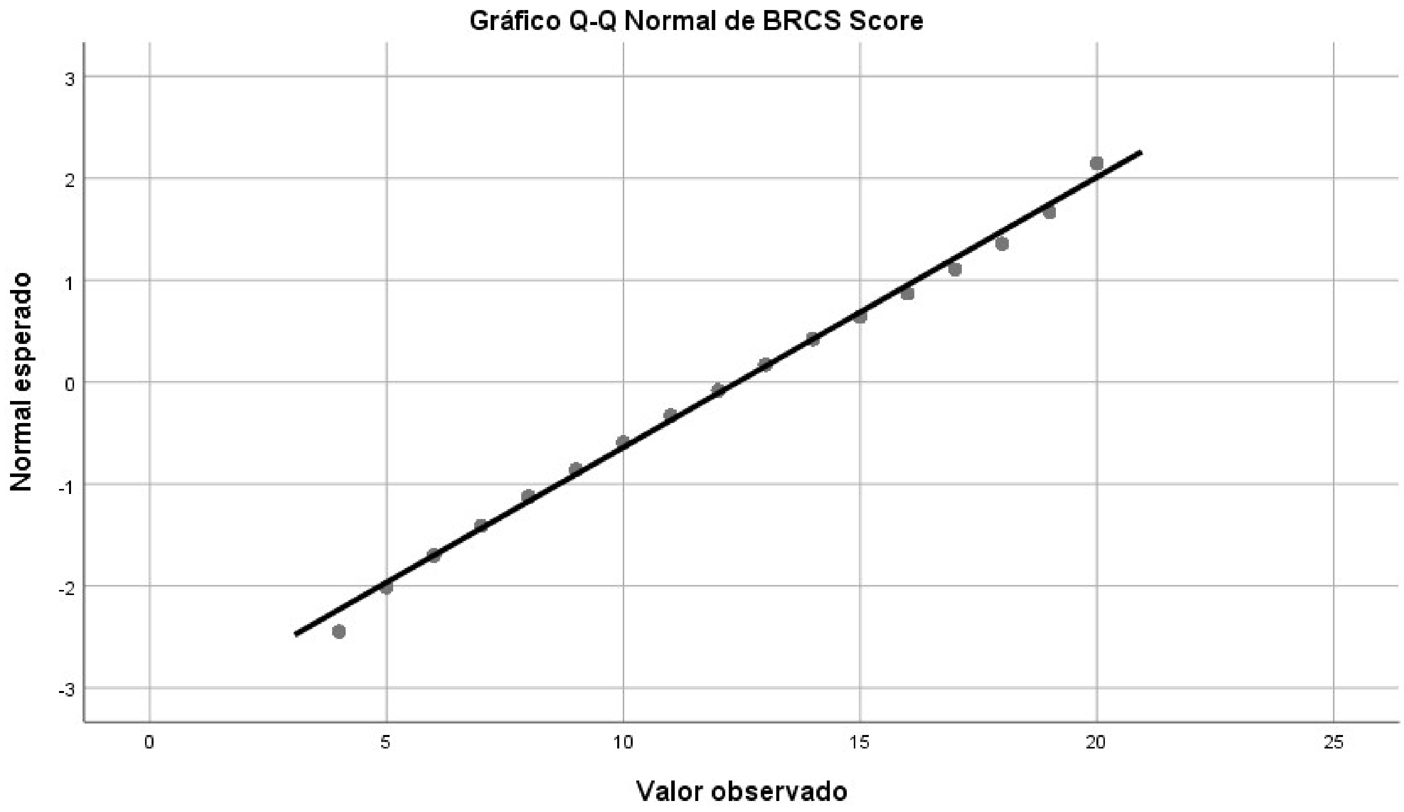

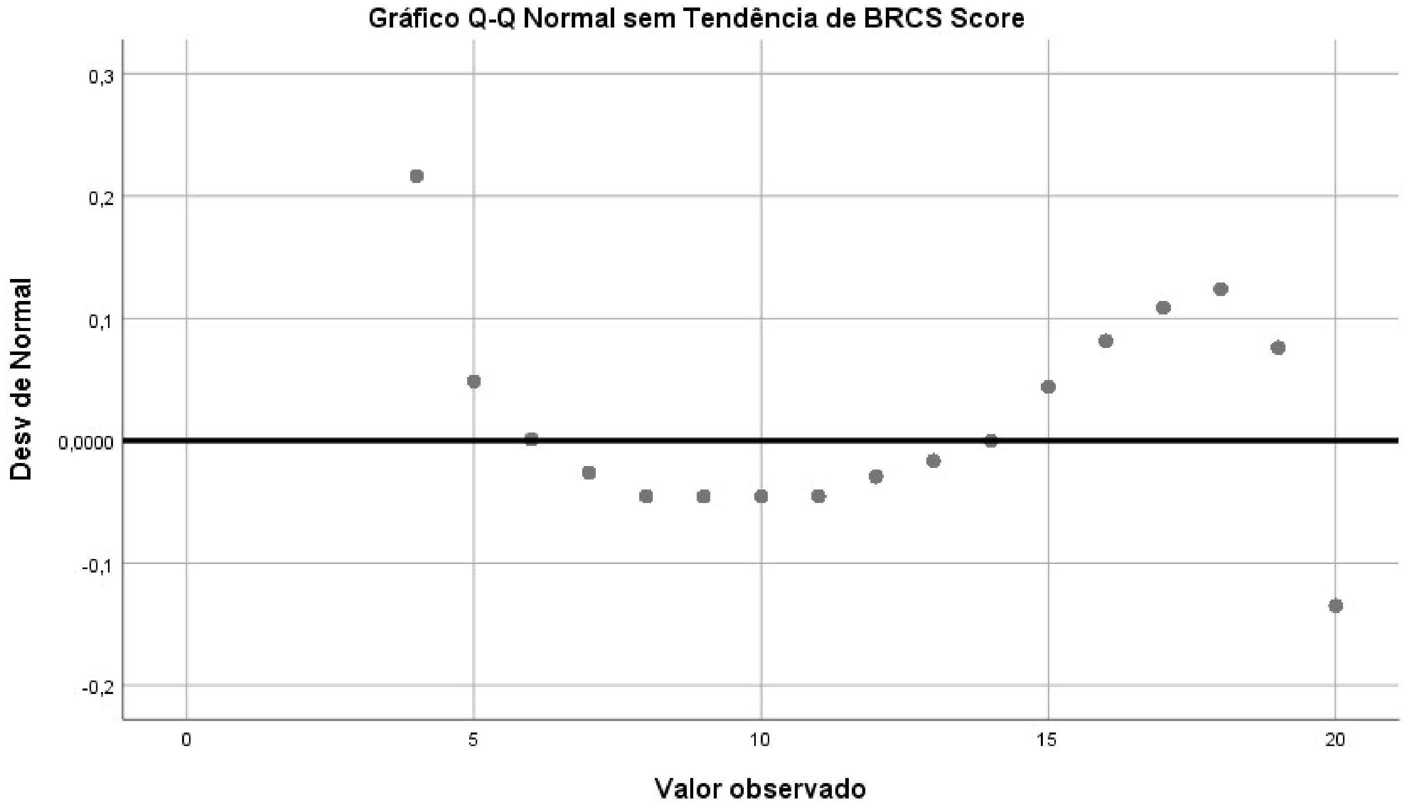

OLBI Disengagement Score

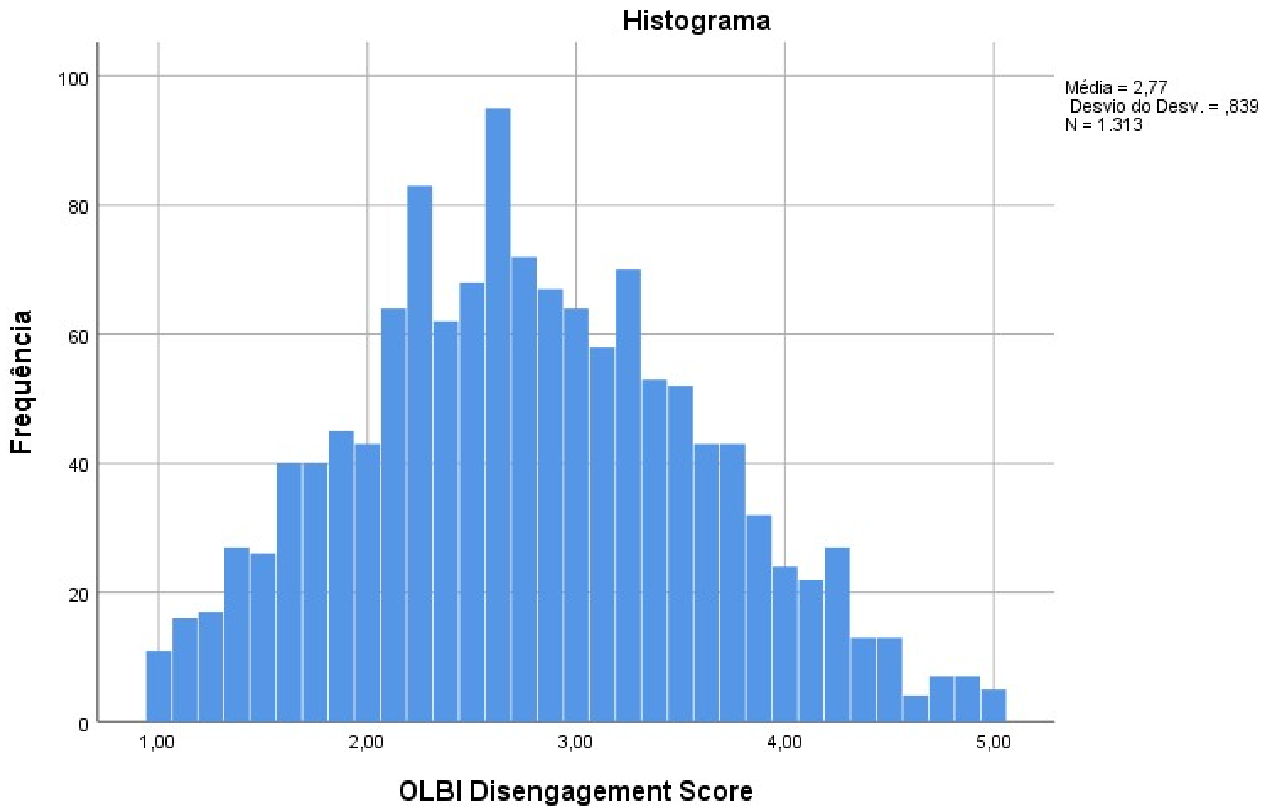
Histograma
Média = 2,77
Desvio do Desv. = ,839
N = 1.313
Frequência
100
80
60
40
20
0
1,00
2,00
3,00
4,00
5,00
OLBI Disengagement Score

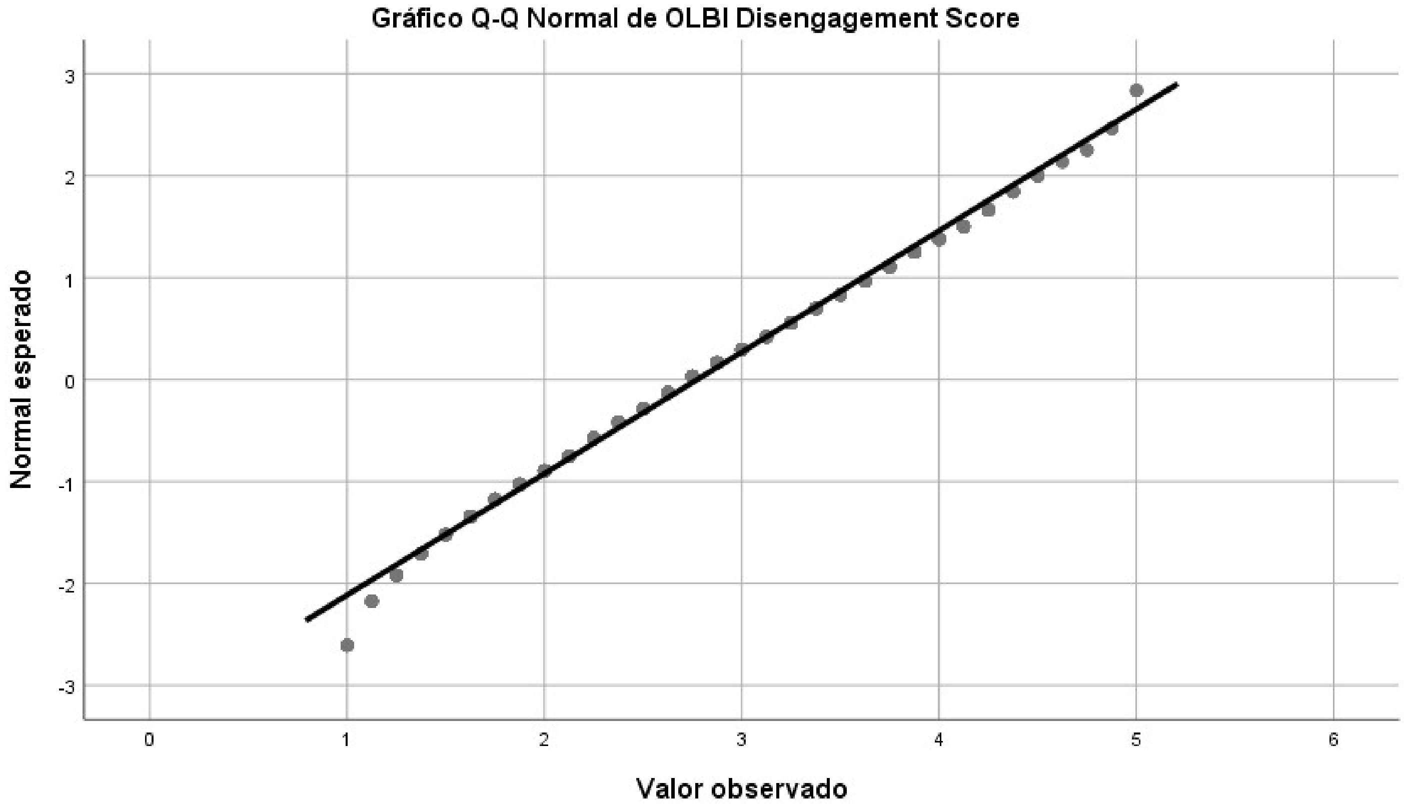
Gráfico Q-Q Normal de OLBI Disengagement Score
Normal esperado
3
2
1
0
-1
-2
-3
0
1
2
3
4
5
6
Valor observado

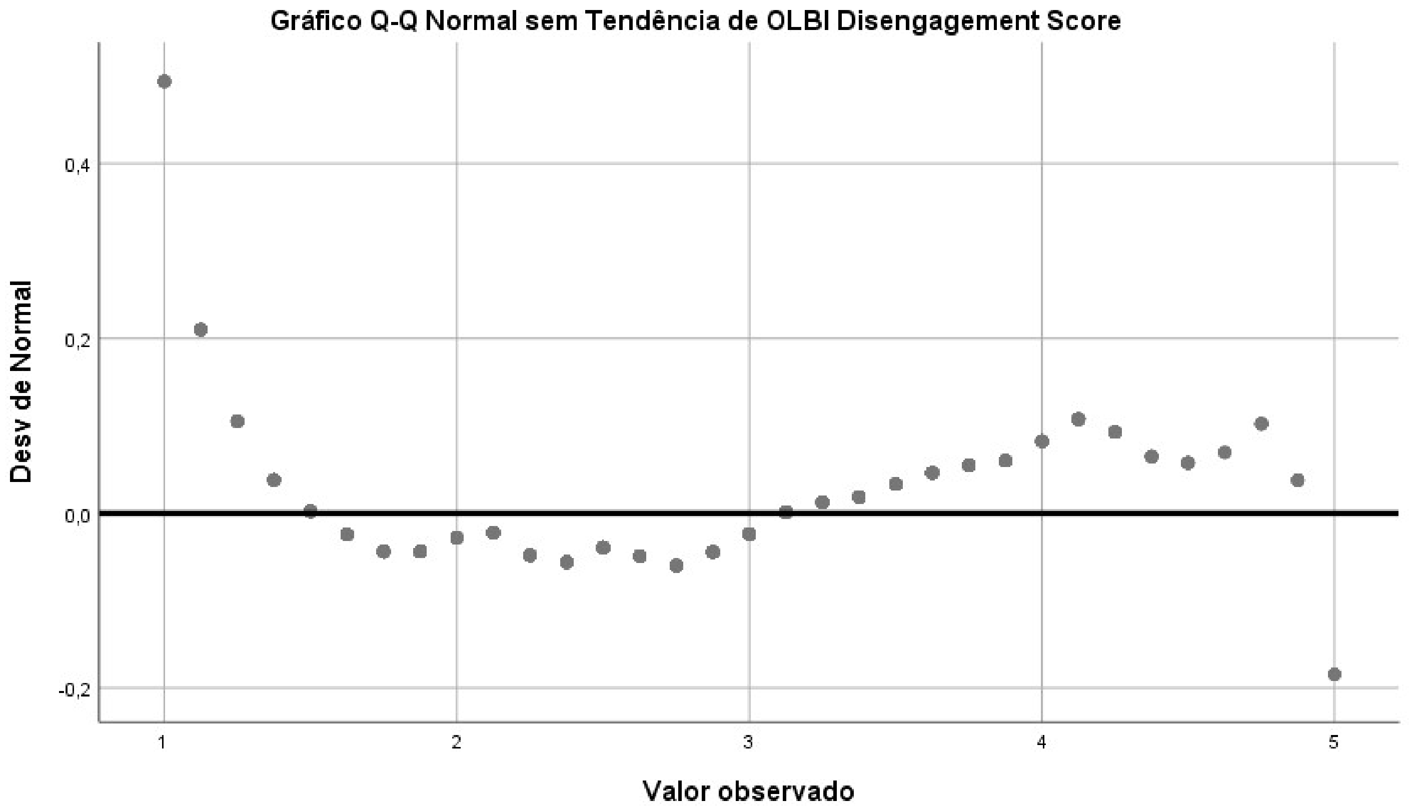

OLBI Exhaustion Score

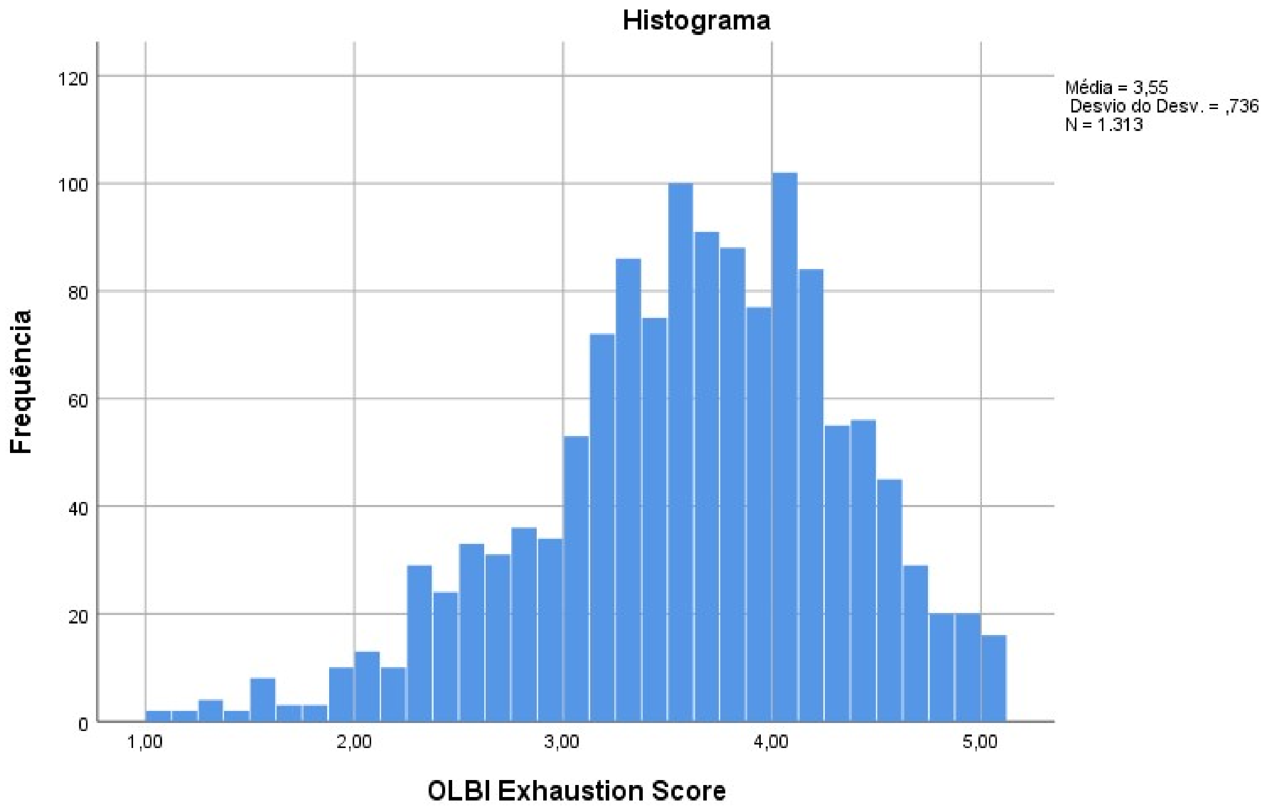

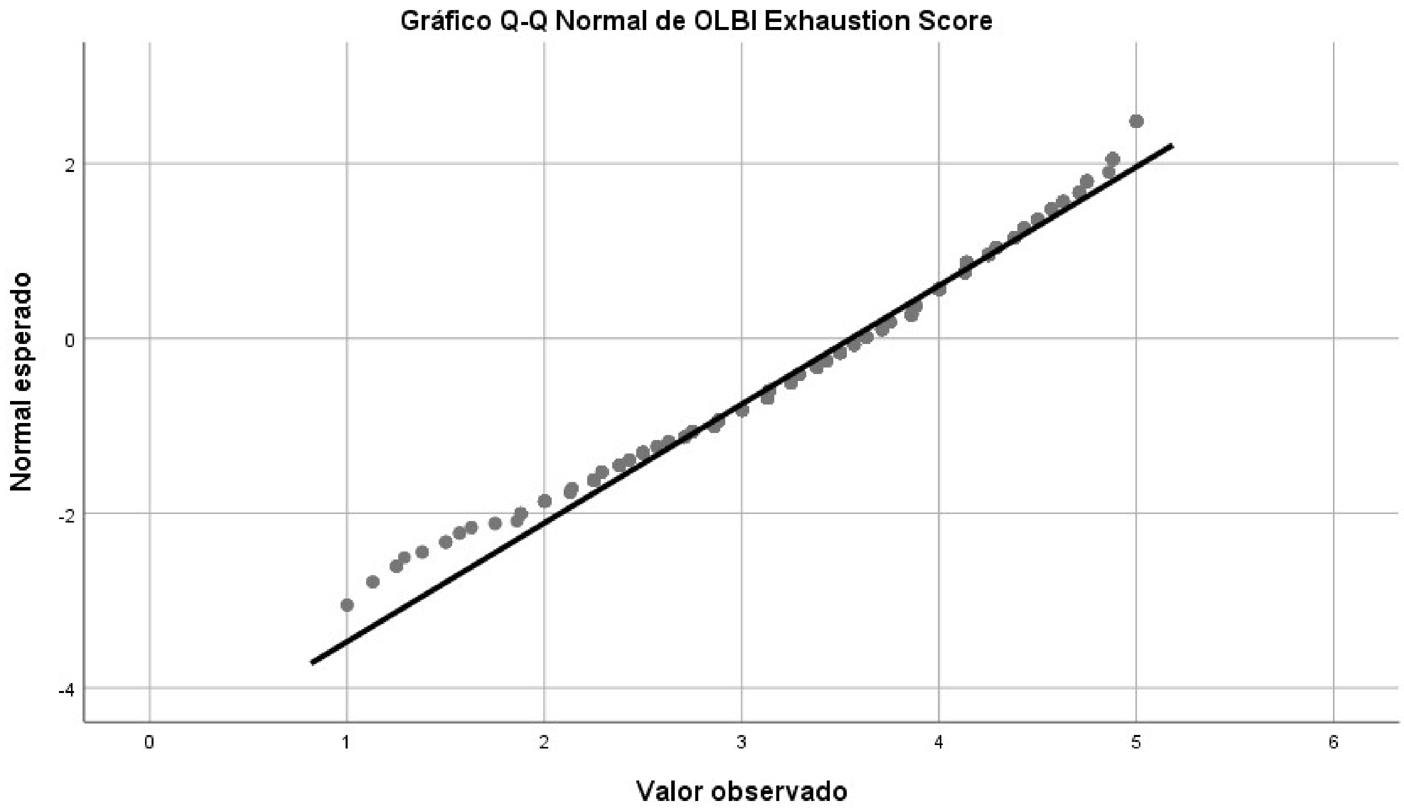

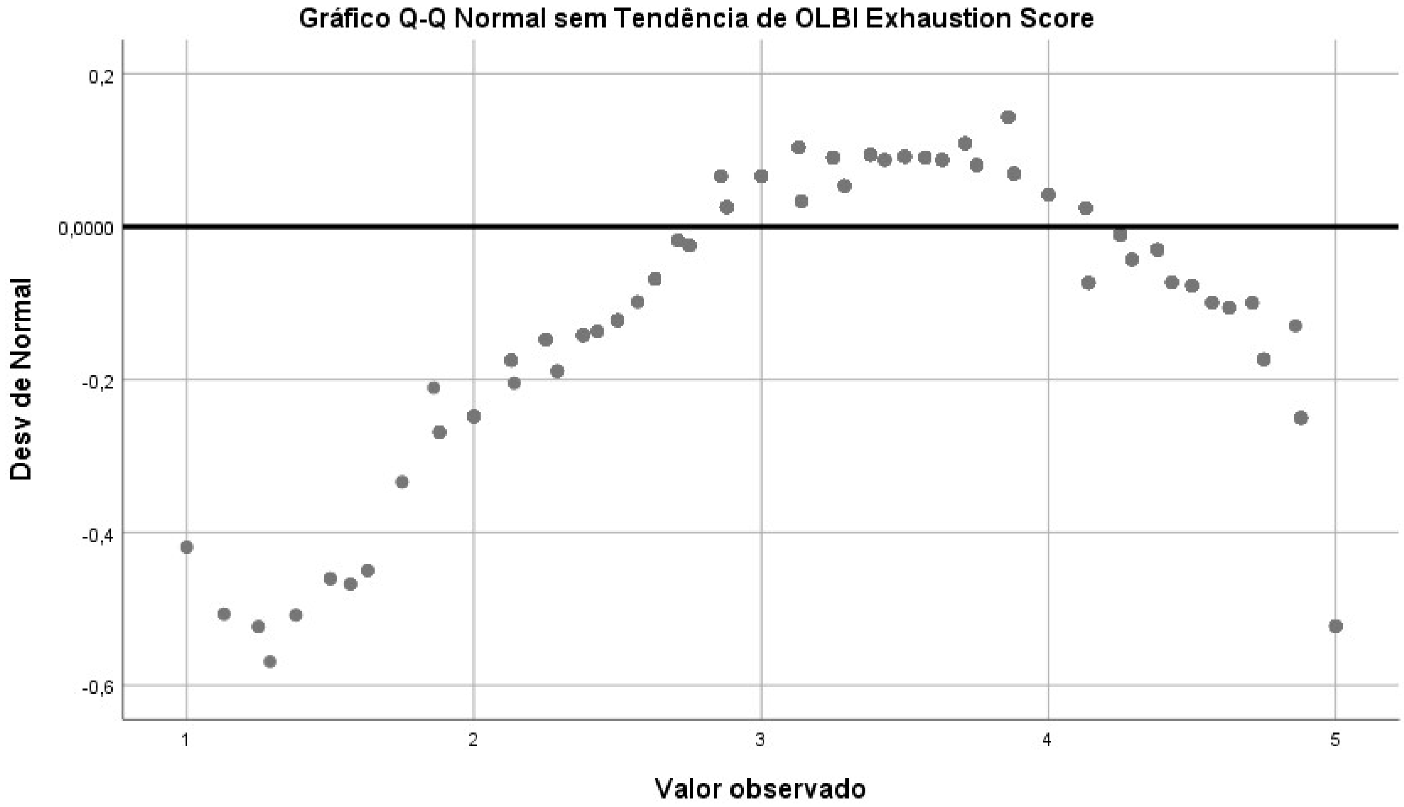

OLBI Total Score

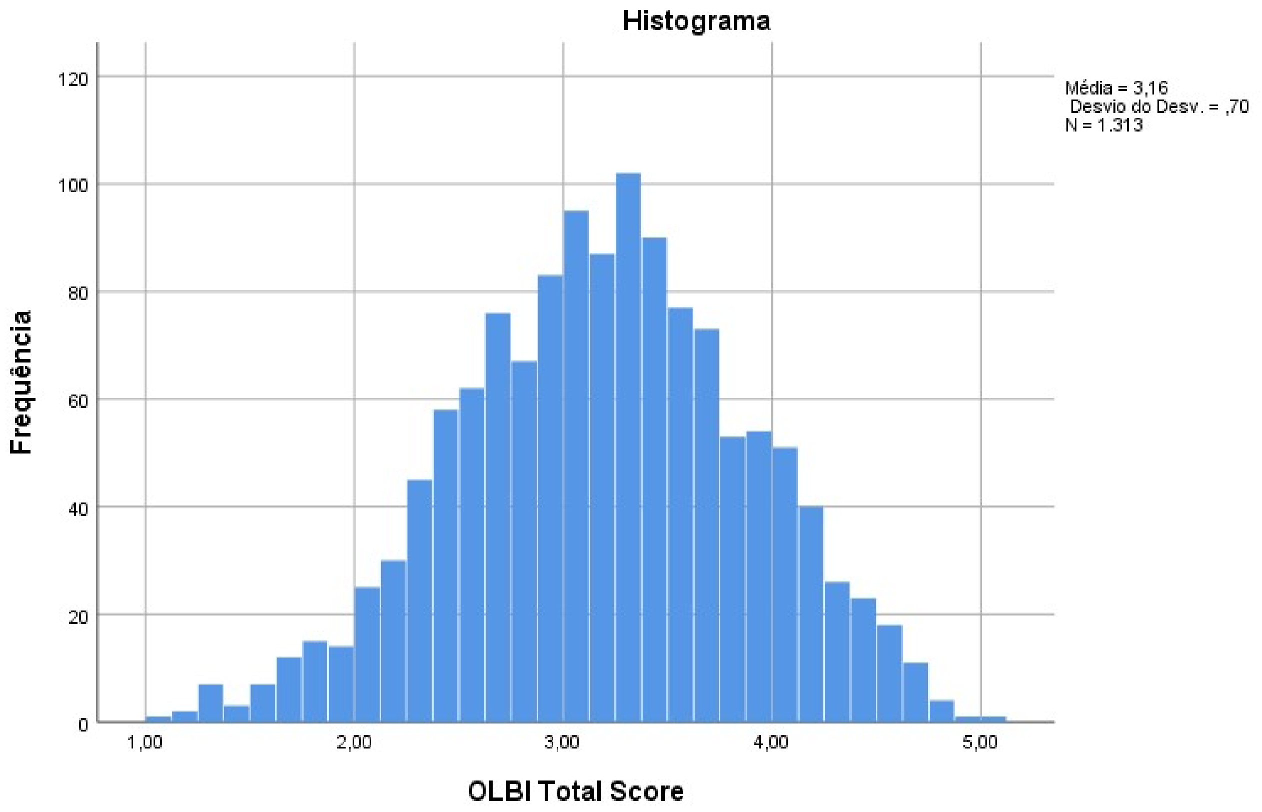
Histograma
Média = 3,16
Desvio do Desv. = ,70
N = 1.313
Frequência
120
100
80
60
40
20
0
1,00
2,00
3,00
4,00
5,00
OLBI Total Score

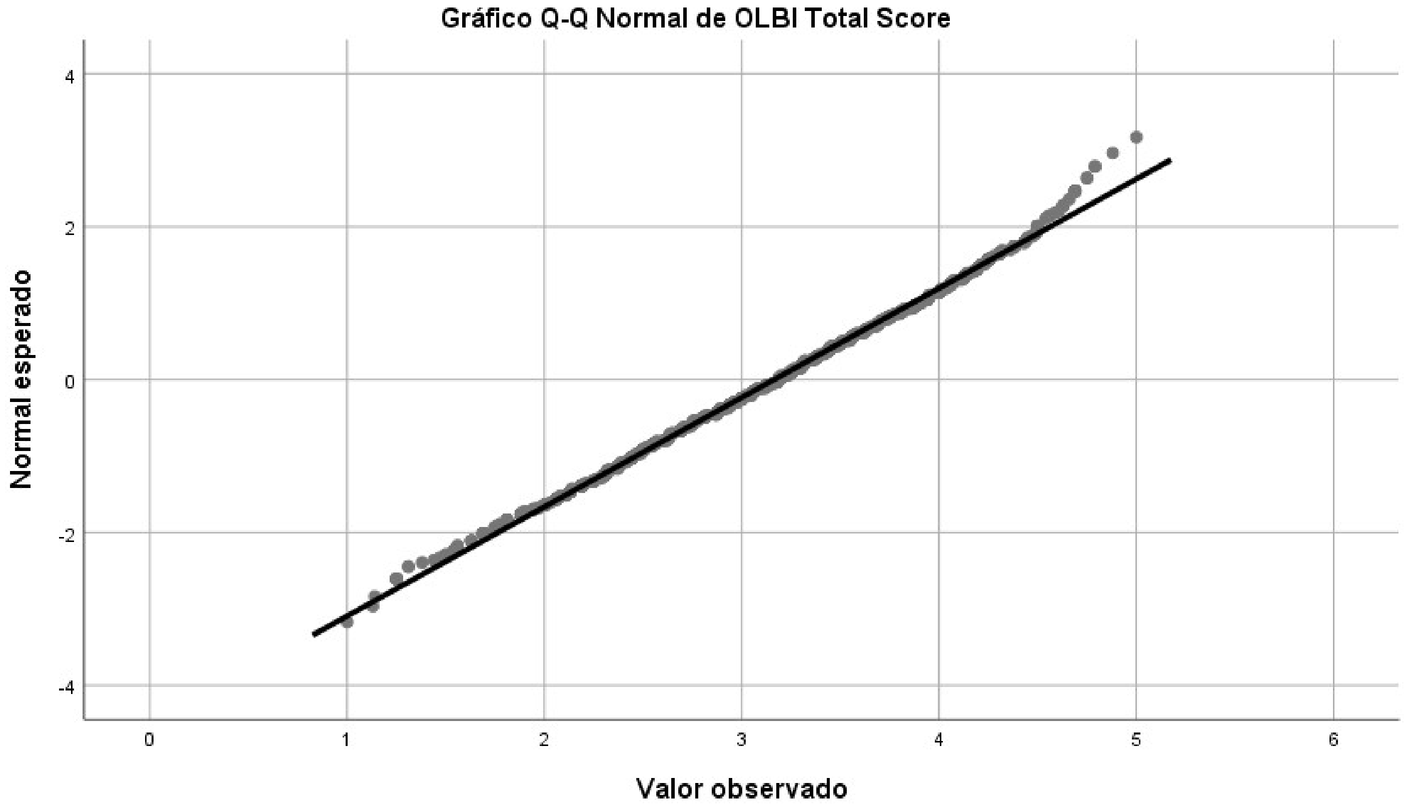
Gráfico Q-Q Normal de OLBI Total Score
Normal esperado
4
2
0
-2
-4
0
1
2
3
4
5
6
Valor observado

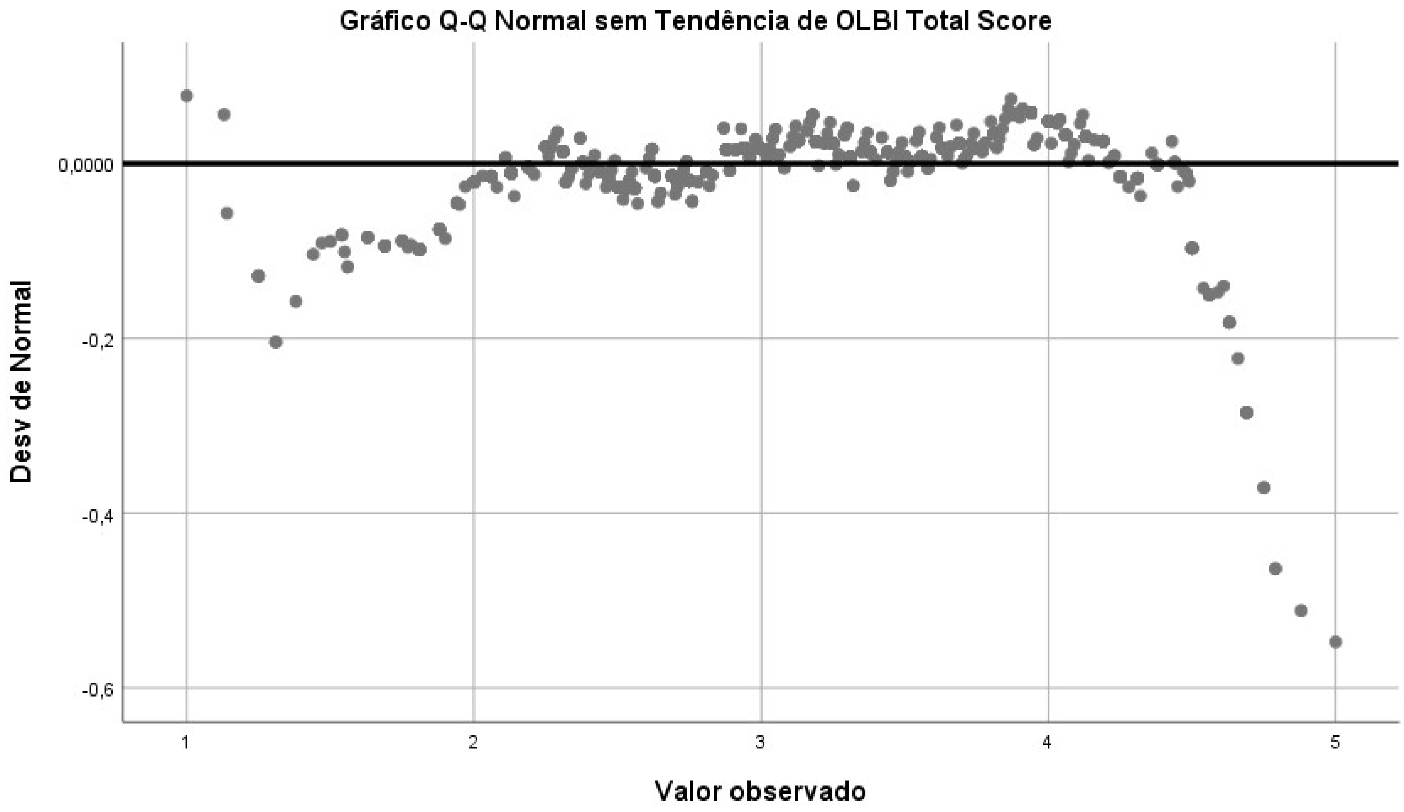


```
FREQUENCIES VARIABLES=DASS21_Classif_D5c DASS21_Classif_A5c DASS21_Classif_S5c DASS21_Classif_D2c
    DASS21_Classif_A2c DASS21_Classif_S2c PHQ9_Classif BRCS_Classif OLBI_Classif_2c
  /BARCHART PERCENT
  /ORDER=VARIABLE.
```

Frequências

Observações

Saída criada		20-SEP-2020 11:25:30
Comentários		
Entrada	Dados	C:\Users\User\Documents\Pesquisa\Fellow\FellowGenData_V1.sav
	Conjunto de dados ativo	ConjuntodeDados1
	Filtro	<none>
	Ponderação	<none>
	Arquivo Dividido	<none>
	N de linhas em arquivo de dados de trabalho	1313
Tratamento de valor omisso	Definição de omisso	Os valores omissos definidos pelo usuário são tratados como omissos.
	Casos utilizados	As estatísticas estão baseadas em todos os casos com dados válidos.

Sintaxe		FREQUENCIES VARIABLES=DASS21_Classif_D5c DASS21_Classif_A5c DASS21_Classif_S5c DASS21_Classif_D2c DASS21_Classif_A2c DASS21_Classif_S2c PHQ9_Classif BRCS_Classif OLBI_Classif_2c /BARCHART PERCENT /ORDER=VARIABLE.
Recursos	Tempo do processador	00:00:01,44
	Tempo decorrido	00:00:01,45

DASS21 Classification - Depression

Estatísticas

DASS21 Classification - Depression

N	Válido	1313
	Omisso	0

DASS21 Classification - Depression

		Frequência	Porcentagem	Porcentagem válida	Porcentagem acumulativa
Válido	Normal	870	66,3	66,3	66,3
	Minimal	226	17,2	17,2	83,5
	Moderate	190	14,5	14,5	97,9
	High	27	2,1	2,1	100,0
	Total	1313	100,0	100,0	

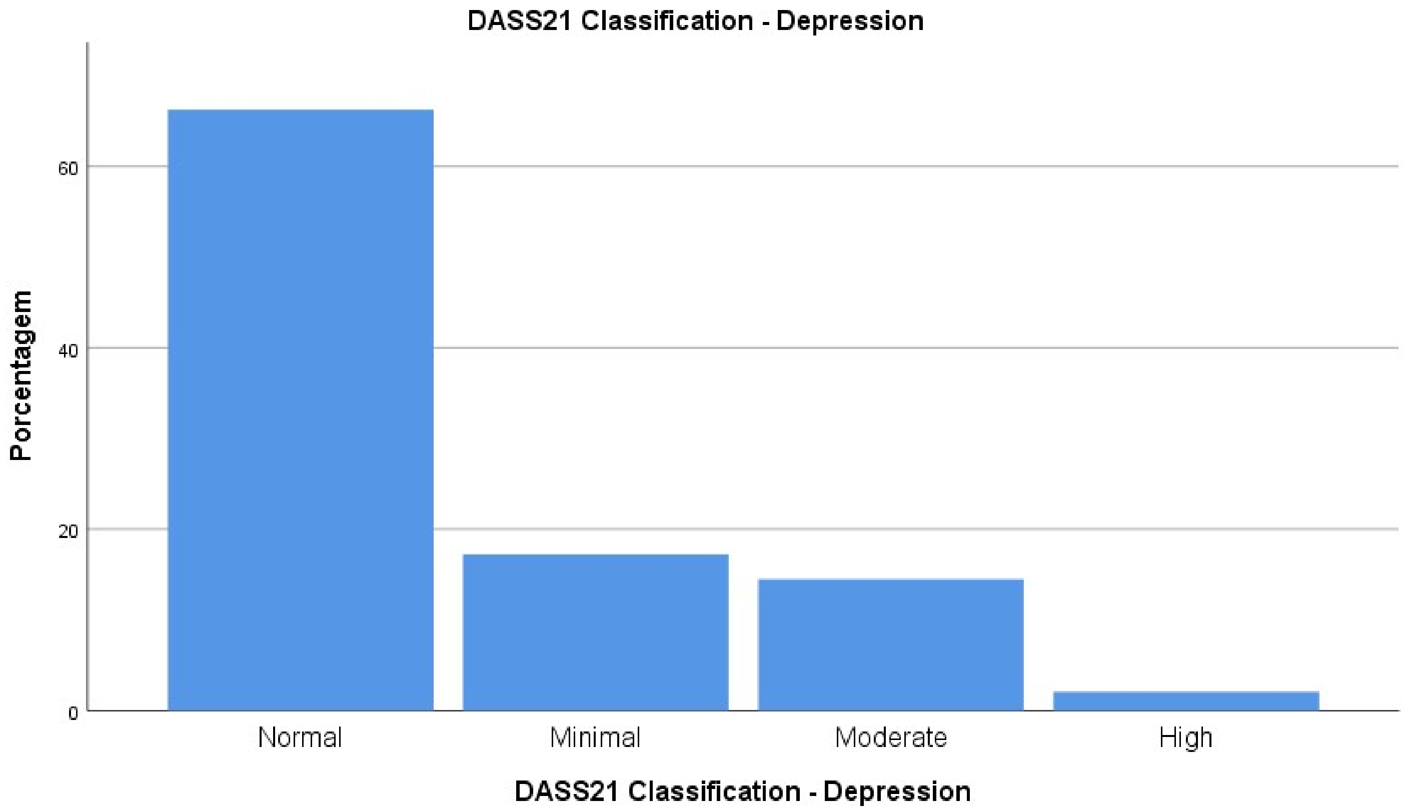

DASS21 Classification - Anxiety

Estatísticas

DASS21 Classification - Anxiety

N	Válido	1313
	Omisso	0

DASS21 Classification - Anxiety

		Frequência	Porcentagem	Porcentagem válida	Porcentagem acumulativa
Válido	Normal	861	65,6	65,6	65,6
	Minimal	139	10,6	10,6	76,2
	Moderate	217	16,5	16,5	92,7
	High	72	5,5	5,5	98,2
	Very High	24	1,8	1,8	100,0
	Total	1313	100,0	100,0	

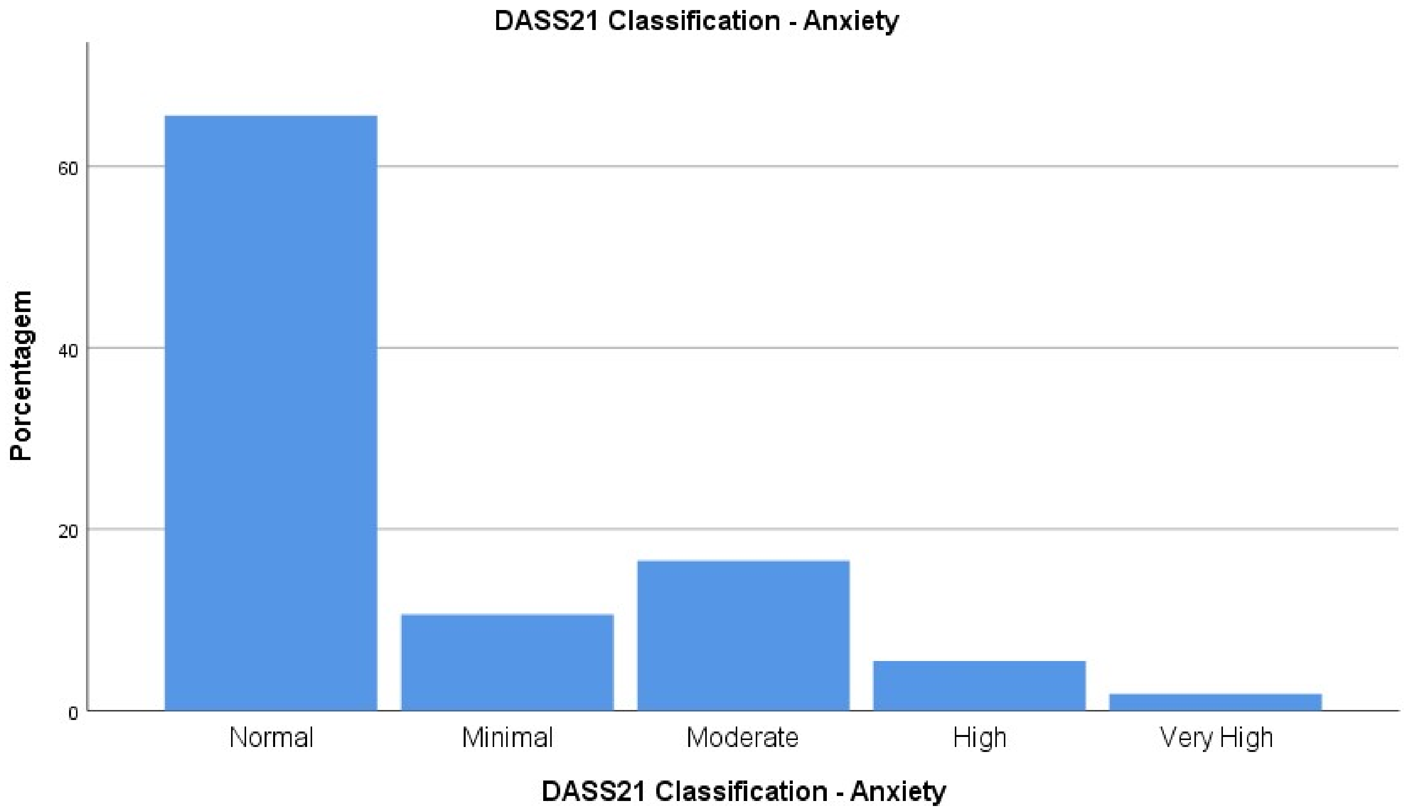

DASS21 Classification - Stress

Estatísticas

DASS21 Classification - Stress

N	Válido	1313
	Omisso	0

DASS21 Classification - Stress

		Frequência	Porcentagem	Porcentagem válida	Porcentagem acumulativa
Válido	Normal	1012	77,1	77,1	77,1
	Minimal	203	15,5	15,5	92,5
	Moderate	98	7,5	7,5	100,0
	Total	1313	100,0	100,0	

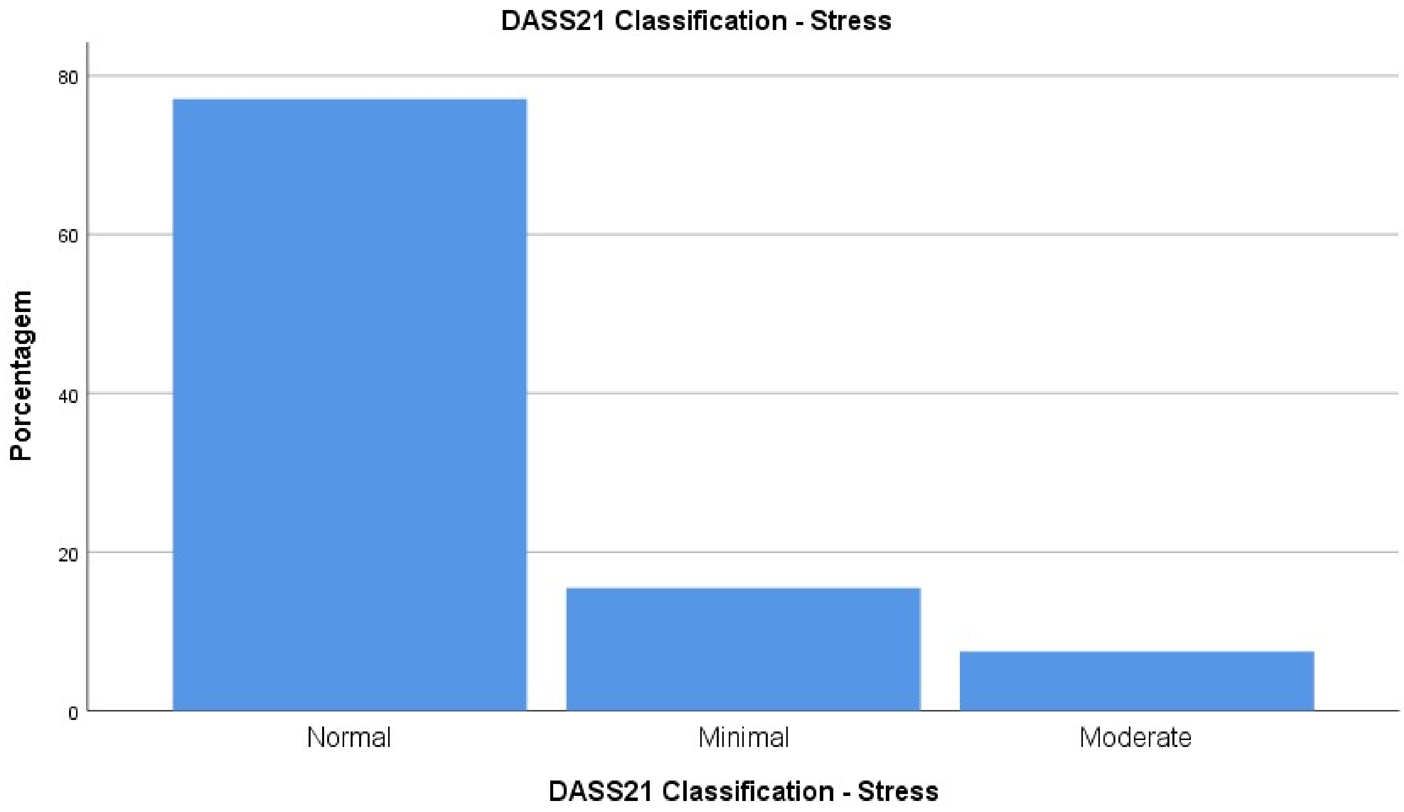

DASS21 Classification - Depression (dichotomous)

Estatísticas

DASS21 Classification - Depression (dichotomous)

N	Válido	1313
	Omisso	0

DASS21 Classification - Depression (dichotomous)

		Frequência	Porcentagem	Porcentagem válida	Porcentagem acumulativa
Válido	Normal	870	66,3	66,3	66,3
	Abnormal (elevated)	443	33,7	33,7	100,0
	Total	1313	100,0	100,0	

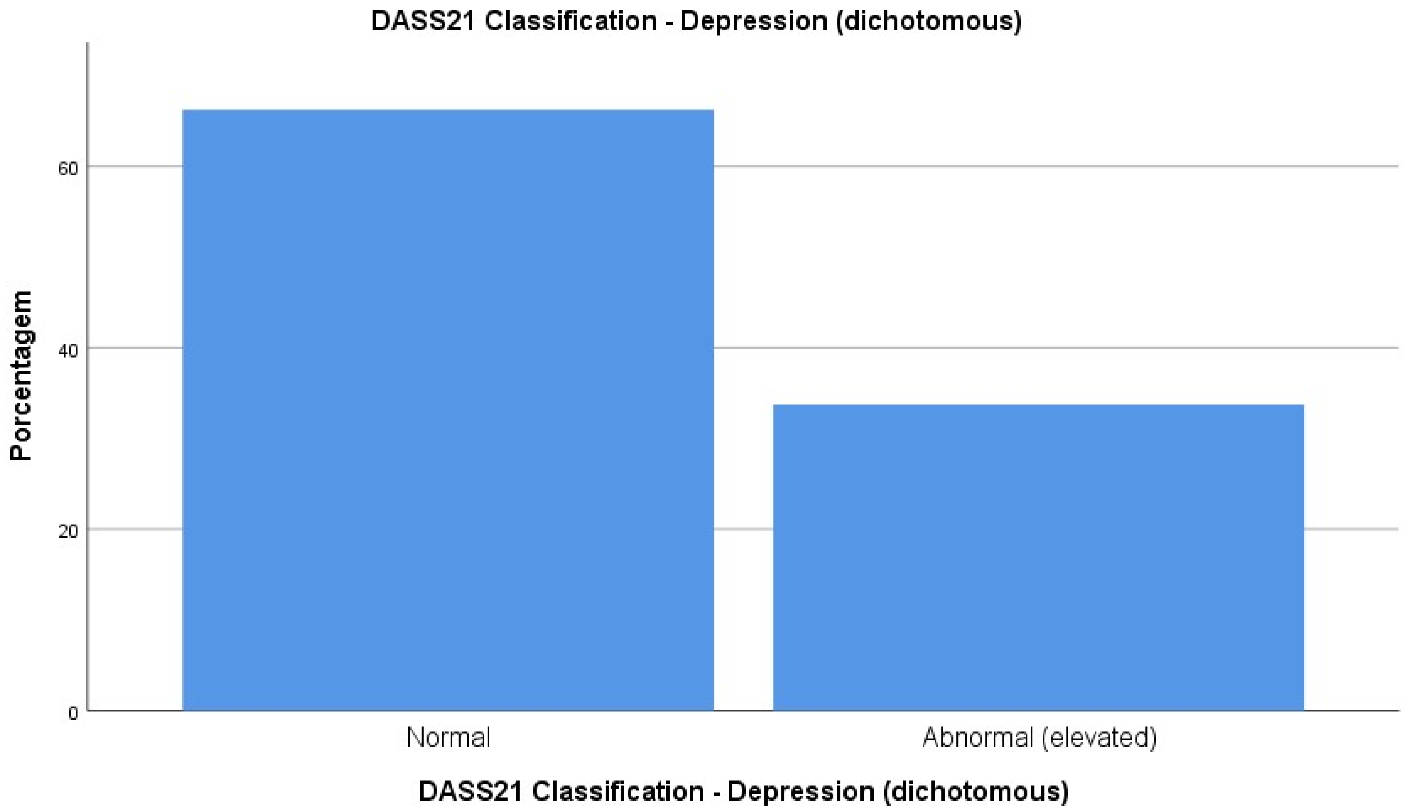

DASS21 Classification - Anxiety (dichotomous)

Estatísticas

DASS21 Classification - Anxiety (dichotomous)

N	Válido	1313
	Omisso	0

DASS21 Classification - Anxiety (dichotomous)

		Frequência	Porcentagem	Porcentagem válida	Porcentagem acumulativa
Válido	Normal	861	65,6	65,6	65,6
	Abnormal (elevated)	452	34,4	34,4	100,0
	Total	1313	100,0	100,0	

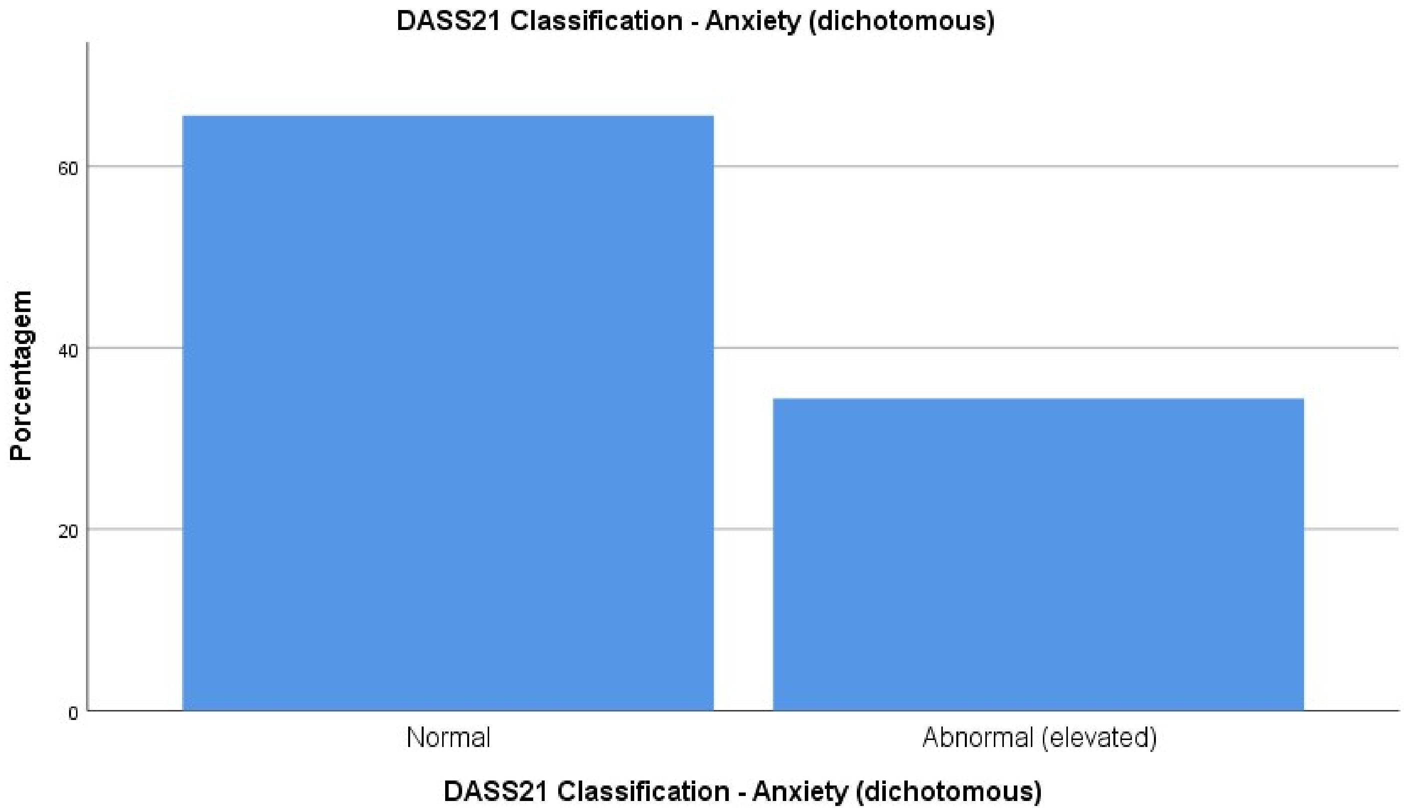

DASS21 Classification - Stress (dichotomous)

Estatísticas

DASS21 Classification - Stress (dichotomous)

N	Válido	1313
	Omisso	0

DASS21 Classification - Stress (dichotomous)

		Frequência	Porcentagem	Porcentagem válida	Porcentagem acumulativa
Válido	Normal	1012	77,1	77,1	77,1
	Abnormal (elevated)	301	22,9	22,9	100,0
	Total	1313	100,0	100,0	

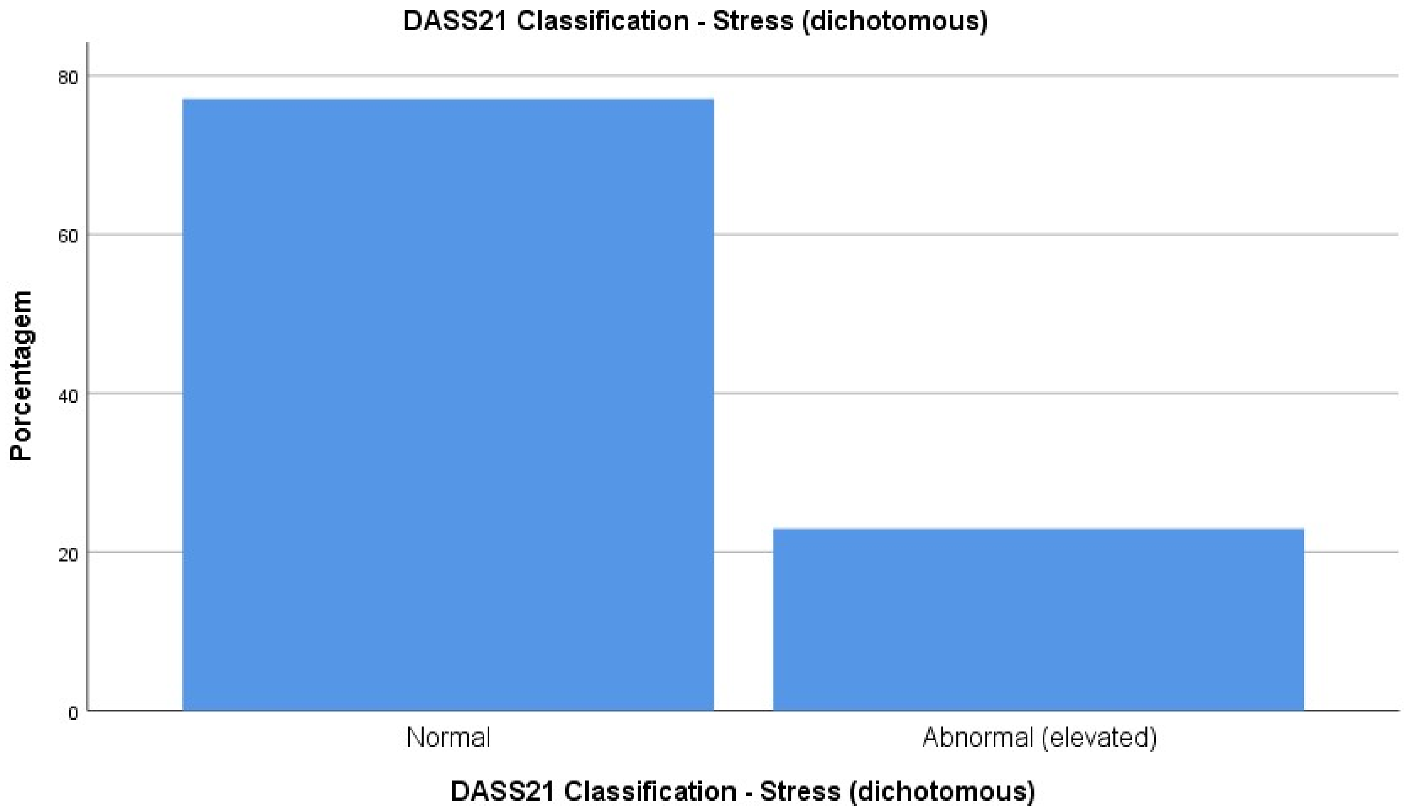

PHQ9 Depression Classification - Risk estimate for having current major depressive disorder

Estatísticas

PHQ9 Depression Classification - Risk estimate for having current major depressive disorder

N	Válido	1313
	Omisso	0

PHQ9 Depression Classification - Risk estimate for having current major depressive disorder

		Frequência	Porcentagem	Porcentagem válida	Porcentagem acumulativa
Válido	Low	429	32,7	32,7	32,7
	High	884	67,3	67,3	100,0
	Total	1313	100,0	100,0	

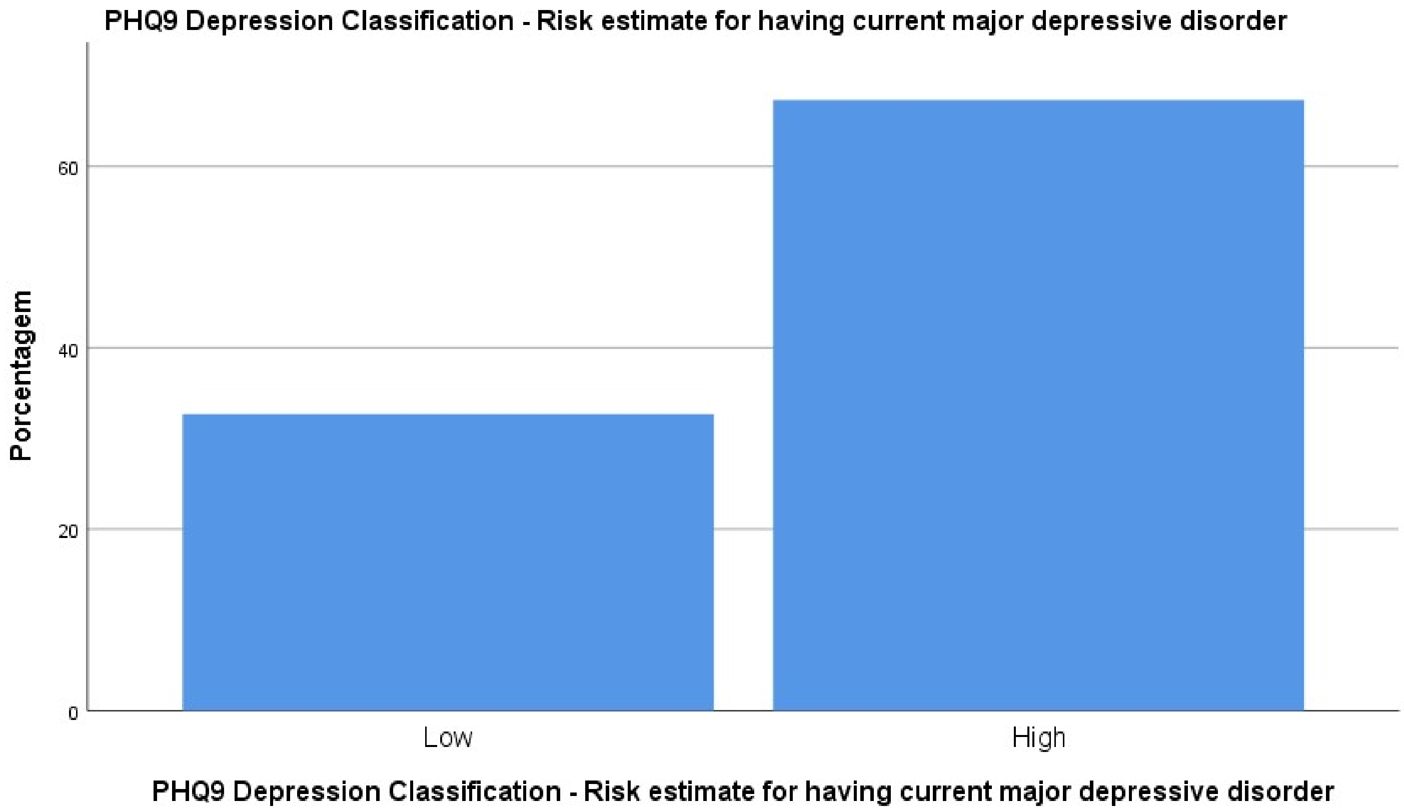

BRCS Classification

Estatísticas

BRCS Classification

N	Válido	1313
	Omisso	0

BRCS Classification

		Frequência	Porcentagem	Porcentagem válida	Porcentagem acumulativa
Válido	Low resilience	813	61,9	61,9	61,9
	Moderate to High	500	38,1	38,1	100,0
	Total	1313	100,0	100,0	

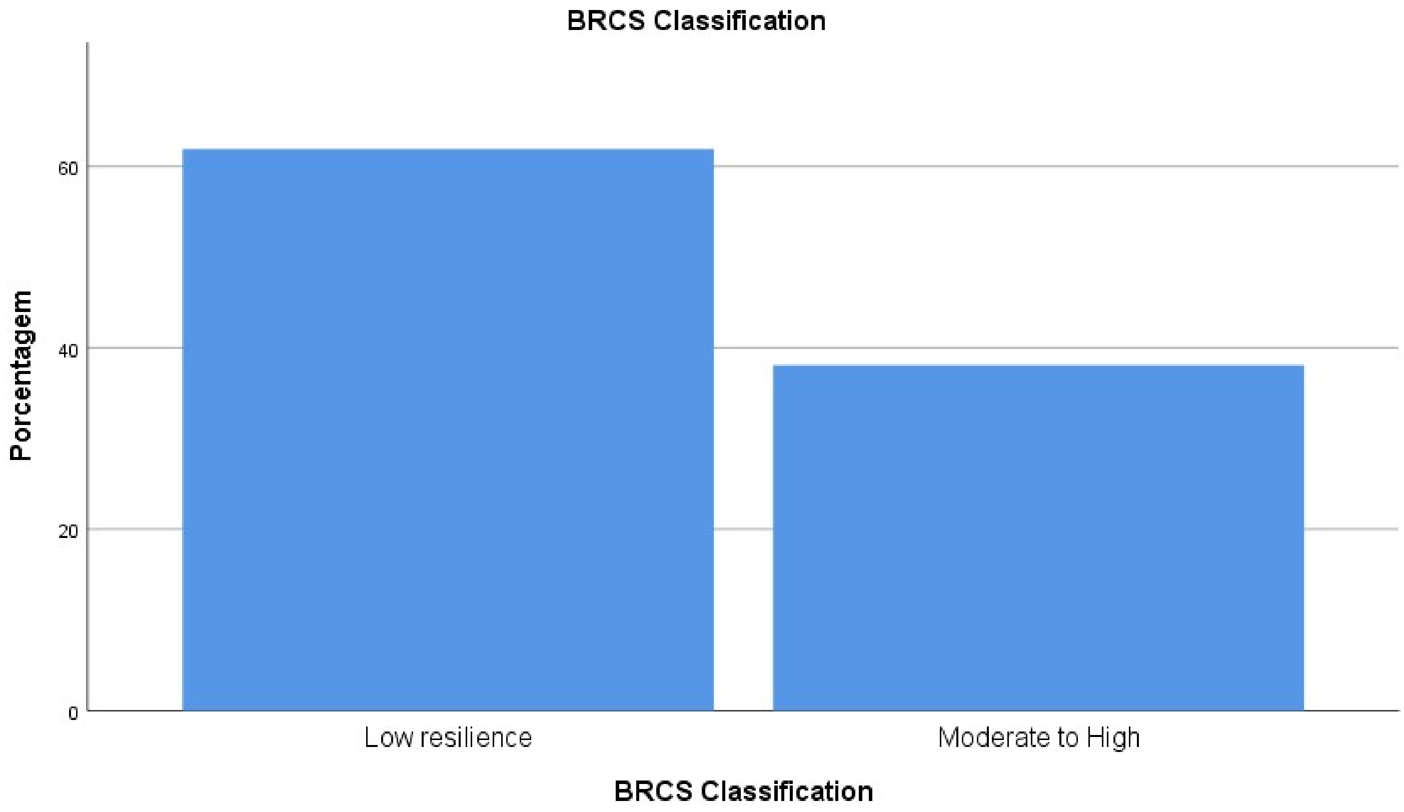

OLBI Score Classification

Estatísticas

OLBI Score Classification

N	Válido	1313
	Omisso	0

OLBI Score Classification

		Frequência	Porcentagem	Porcentagem válida	Porcentagem acumulativa
Válido	Low to Moderate	875	66,6	66,6	66,6
	High	438	33,4	33,4	100,0
	Total	1313	100,0	100,0	

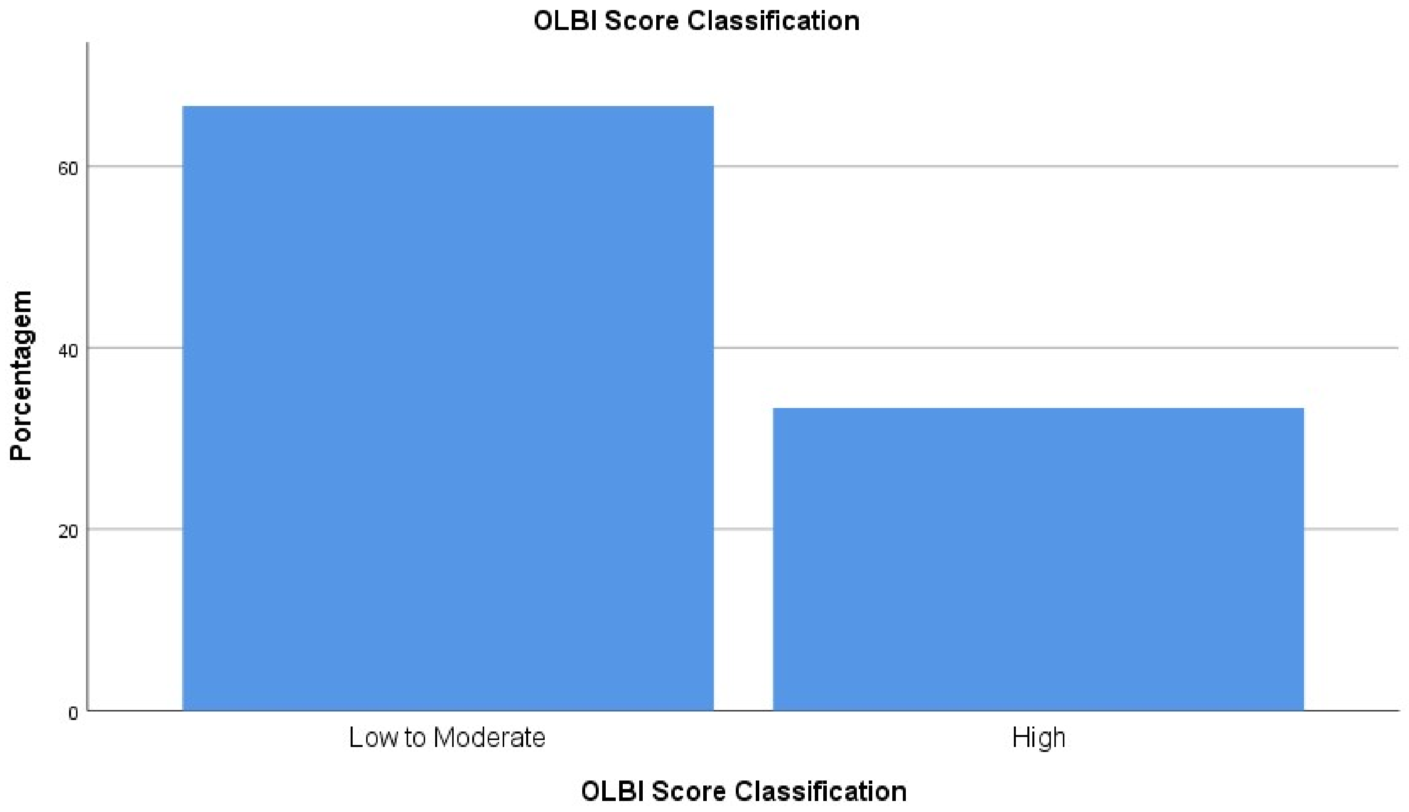

FREQUENCIES VARIABLES=DASS21_Q1S DASS21_Q2A DASS21_Q3D DASS21_Q4A DASS21_Q5D DASS21_Q6S DASS21_Q7A
DASS21_Q8S DASS21_Q9A DASS21_Q10D DASS21_Q11S DASS21_Q12S DASS21_Q13D DASS21_Q14S DASS21_Q15A
DASS21_Q16D DASS21_Q17D DASS21_Q18S DASS21_Q19A DASS21_Q20A DASS21_Q21D
/ORDER=VARIABLE.

Frequências

Observações

Saída criada		20-SEP-2020 11:27:47
Comentários		
Entrada	Dados	C:\Users\User\Documents\Pesquisa\Fellow\FellowGenData_V1.sav
	Conjunto de dados ativo	ConjuntodeDados1
	Filtro	<none>
	Ponderação	<none>
	Arquivo Dividido	<none>
	N de linhas em arquivo de dados de trabalho	1313
Tratamento de valor omisso	Definição de omisso	Os valores omissos definidos pelo usuário são tratados como omissos.

	Casos utilizados	As estatísticas estão baseadas em todos os casos com dados válidos.
Sintaxe		FREQUENCIES VARIABLES=DASS21_Q1S DASS21_Q2A DASS21_Q3D DASS21_Q4A DASS21_Q5D DASS21_Q6S DASS21_Q7A DASS21_Q8S DASS21_Q9A DASS21_Q10D DASS21_Q11S DASS21_Q12S DASS21_Q13D DASS21_Q14S DASS21_Q15A DASS21_Q16D DASS21_Q17D DASS21_Q18S DASS21_Q19A DASS21_Q20A DASS21_Q21D /ORDER=VARIABLE.
Recursos	Tempo do processador	00:00:00,02
	Tempo decorrido	00:00:00,02

```
Achei difícil me acalmar
```

Estatísticas

Achei difícil me acalmar

N	Válido	1313
	Omisso	0

Achei difícil me acalmar

		Frequência	Porcentagem	Porcentagem válida	Porcentagem acumulativa
Válido	Não se aplica a mim	223	17,0	17,0	17,0
	Aplicou-se em algum grau, por pouco tempo	542	41,3	41,3	58,3
	Aplicou-se consideravelmente, boa parte do tempo	378	28,8	28,8	87,1
	Aplicou-se muito, a maior parte do tempo	170	12,9	12,9	100,0
	Total	1313	100,0	100,0	

```
Senti minha boca seca
```

Estatísticas

Senti minha boca seca

N	Válido	1313
	Omisso	0

Senti minha boca seca

		Frequência	Porcentagem	Porcentagem válida	Porcentagem acumulativa
Válido	Não se aplica a mim	634	48,3	48,3	48,3
	Aplicou-se em algum grau, por pouco tempo	381	29,0	29,0	77,3
	Aplicou-se consideravelmente, boa parte do tempo	194	14,8	14,8	92,1
	Aplicou-se muito, a maior parte do tempo	104	7,9	7,9	100,0
	Total	1313	100,0	100,0	

Não consegui vivenciar nenhum sentimento positivo

Estatísticas

Não consegui vivenciar nenhum sentimento positivo

N	Válido	1313
	Omisso	0

Não consegui vivenciar nenhum sentimento positivo

		Frequência	Porcentagem	Porcentagem válida	Porcentagem acumulativa
Válido	Não se aplica a mim	541	41,2	41,2	41,2
	Aplicou-se em algum grau, por pouco tempo	491	37,4	37,4	78,6
	Aplicou-se consideravelmente, boa parte do tempo	202	15,4	15,4	94,0
	Aplicou-se muito, a maior parte do tempo	79	6,0	6,0	100,0
	Total	1313	100,0	100,0	

Tive dificuldade em respirar em alguns momentos (sem ter feito nenhum esforço físico)

Estatísticas

Tive dificuldade em respirar em alguns momentos (sem ter feito nenhum esforço físico)

N	Válido	1313
	Omisso	0

Tive dificuldade em respirar em alguns momentos (sem ter feito nenhum esforço físico)

		Frequência	Porcentagem	Porcentagem válida	Porcentagem acumulativa
Válido	Não se aplica a mim	611	46,5	46,5	46,5
	Aplicou-se em algum grau, por pouco tempo	406	30,9	30,9	77,5
	Aplicou-se consideravelmente, boa parte do tempo	211	16,1	16,1	93,5
	Aplicou-se muito, a maior parte do tempo	85	6,5	6,5	100,0
	Total	1313	100,0	100,0	

```
Achei difícil ter iniciativa para fazer as coisas
```

Estatísticas

Achei difícil ter iniciativa para fazer as coisas

N	Válido	1313
	Omisso	0

Achei difícil ter iniciativa para fazer as coisas

		Frequência	Porcentagem	Porcentagem válida	Porcentagem acumulativa
Válido	Não se aplica a mim	211	16,1	16,1	16,1
	Aplicou-se em algum grau, por pouco tempo	431	32,8	32,8	48,9
	Aplicou-se consideravelmente, boa parte do tempo	409	31,2	31,2	80,0
	Aplicou-se muito, a maior parte do tempo	262	20,0	20,0	100,0
	Total	1313	100,0	100,0	

Tive a tendência de reagir de forma exagerada às situações

Estatísticas

Tive a tendência de reagir de forma exagerada às situações

N	Válido	1313
	Omisso	0

Tive a tendência de reagir de forma exagerada às situações

		Frequência	Porcentagem	Porcentagem válida	Porcentagem acumulativa
Válido	Não se aplica a mim	279	21,2	21,2	21,2
	Aplicou-se em algum grau, por pouco tempo	470	35,8	35,8	57,0
	Aplicou-se consideravelmente, boa parte do tempo	360	27,4	27,4	84,5
	Aplicou-se muito, a maior parte do tempo	204	15,5	15,5	100,0
	Total	1313	100,0	100,0	

Senti tremores (ex.: nas mãos)

Estatísticas

Senti tremores (ex.: nas mãos)

N	Válido	1313
	Omisso	0

Senti tremores (ex.: nas mãos)

		Frequência	Porcentagem	Porcentagem válida	Porcentagem acumulativa
Válido	Não se aplica a mim	823	62,7	62,7	62,7
	Aplicou-se em algum grau, por pouco tempo	296	22,5	22,5	85,2
	Aplicou-se consideravelmente, boa parte do tempo	122	9,3	9,3	94,5

		Frequência	Porcentagem	Porcentagem válida	Porcentagem acumulativa
	Aplicou-se muito, a maior parte do tempo	72	5,5	5,5	100,0
	Total	1313	100,0	100,0	

Senti que estava sempre nervoso(a)

Estatísticas

Senti que estava sempre nervoso(a)

N	Válido	1313
	Omisso	0

Senti que estava sempre nervoso(a)

		Frequência	Porcentagem	Porcentagem válida	Porcentagem acumulativa
Válido	Não se aplica a mim	262	20,0	20,0	20,0
	Aplicou-se em algum grau, por pouco tempo	480	36,6	36,6	56,5
	Aplicou-se consideravelmente, boa parte do tempo	337	25,7	25,7	82,2
	Aplicou-se muito, a maior parte do tempo	234	17,8	17,8	100,0
	Total	1313	100,0	100,0	

Preocupei-me com situações em que eu pudesse entrar em pânico e parecesse ridículo(a)

Estatísticas

Preocupei-me com situações em que eu pudesse entrar em pânico e parecesse ridículo(a)

N	Válido	1313
	Omisso	0

Preocupei-me com situações em que eu pudesse entrar em pânico e parecesse ridículo(a)

		Frequência	Porcentagem	Porcentagem válida	Porcentagem acumulativa
Válido	Não se aplica a mim	518	39,5	39,5	39,5

	Aplicou-se em algum grau, por pouco tempo	381	29,0	29,0	68,5
	Aplicou-se consideravelmente, boa parte do tempo	243	18,5	18,5	87,0
	Aplicou-se muito, a maior parte do tempo	171	13,0	13,0	100,0
	Total	1313	100,0	100,0	

```
Senti que não tinha nada a desejar
```

Estatísticas

Senti que não tinha nada a desejar

N	Válido	1313
	Omisso	0

Senti que não tinha nada a desejar

		Frequência	Porcentagem	Porcentagem válida	Porcentagem acumulativa
Válido	Não se aplica a mim	571	43,5	43,5	43,5
	Aplicou-se em algum grau, por pouco tempo	384	29,2	29,2	72,7
	Aplicou-se consideravelmente, boa parte do tempo	196	14,9	14,9	87,7
	Aplicou-se muito, a maior parte do tempo	162	12,3	12,3	100,0
	Total	1313	100,0	100,0	

```
Senti-me agitado(a)
```

Estatísticas

Senti-me agitado(a)

N	Válido	1313
	Omisso	0

Senti-me agitado(a)

		Frequência	Porcentagem	Porcentagem válida	Porcentagem acumulativa

Válido	Não se aplica a mim	242	18,4	18,4	18,4
	Aplicou-se em algum grau, por pouco tempo	461	35,1	35,1	53,5
	Aplicou-se consideravelmente, boa parte do tempo	387	29,5	29,5	83,0
	Aplicou-se muito, a maior parte do tempo	223	17,0	17,0	100,0
	Total	1313	100,0	100,0	

Achei difícil relaxar

Estatísticas

Achei difícil relaxar

N	Válido	1313
	Omisso	0

Achei difícil relaxar

		Frequência	Porcentagem	Porcentagem válida	Porcentagem acumulativa
Válido	Não se aplica a mim	127	9,7	9,7	9,7
	Aplicou-se em algum grau, por pouco tempo	384	29,2	29,2	38,9
	Aplicou-se consideravelmente, boa parte do tempo	452	34,4	34,4	73,3
	Aplicou-se muito, a maior parte do tempo	350	26,7	26,7	100,0
	Total	1313	100,0	100,0	

Senti-me depressivo(a) e sem ânimo

Estatísticas

Senti-me depressivo(a) e sem ânimo

N	Válido	1313
	Omisso	0

Senti-me depressivo(a) e sem ânimo

		Frequência	Porcentagem	Porcentagem válida	Porcentagem acumulativa
Válido	Não se aplica a mim	246	18,7	18,7	18,7
	Aplicou-se em algum grau, por pouco tempo	434	33,1	33,1	51,8
	Aplicou-se consideravelmente, boa parte do tempo	376	28,6	28,6	80,4
	Aplicou-se muito, a maior parte do tempo	257	19,6	19,6	100,0
	Total	1313	100,0	100,0	

Fui intolerante com as coisas que me impediam de continuar o que eu estava fazendo

Estatísticas

Fui intolerante com as coisas que me impediam de continuar o que eu estava fazendo

N	Válido	1313
	Omisso	0

Fui intolerante com as coisas que me impediam de continuar o que eu estava fazendo

		Frequência	Porcentagem	Porcentagem válida	Porcentagem acumulativa
Válido	Não se aplica a mim	386	29,4	29,4	29,4
	Aplicou-se em algum grau, por pouco tempo	512	39,0	39,0	68,4
	Aplicou-se consideravelmente, boa parte do tempo	268	20,4	20,4	88,8
	Aplicou-se muito, a maior parte do tempo	147	11,2	11,2	100,0
	Total	1313	100,0	100,0	

Senti que ia entrar em pânico

Estatísticas

Senti que ia entrar em pânico

N	Válido	1313
	Omisso	0

Senti que ia entrar em pânico

		Frequência	Porcentagem	Porcentagem válida	Porcentagem acumulativa
Válido	Não se aplica a mim	667	50,8	50,8	50,8
	Aplicou-se em algum grau, por pouco tempo	361	27,5	27,5	78,3
	Aplicou-se consideravelmente, boa parte do tempo	177	13,5	13,5	91,8
	Aplicou-se muito, a maior parte do tempo	108	8,2	8,2	100,0
	Total	1313	100,0	100,0	

```
Não consegui me entusiasmar com nada
```

Estatísticas

Não consegui me entusiasmar com nada

N	Válido	1313
	Omisso	0

Não consegui me entusiasmar com nada

		Frequência	Porcentagem	Porcentagem válida	Porcentagem acumulativa
Válido	Não se aplica a mim	436	33,2	33,2	33,2
	Aplicou-se em algum grau, por pouco tempo	417	31,8	31,8	65,0
	Aplicou-se consideravelmente, boa parte do tempo	272	20,7	20,7	85,7
	Aplicou-se muito, a maior parte do tempo	188	14,3	14,3	100,0
	Total	1313	100,0	100,0	

```
Senti que não tinha valor como pessoa
```

Estatísticas

Senti que não tinha valor como pessoa

N	Válido	1313

	Omisso	0

Senti que não tinha valor como pessoa

		Frequência	Porcentagem	Porcentagem válida	Porcentagem acumulativa
Válido	Não se aplica a mim	630	48,0	48,0	48,0
	Aplicou-se em algum grau, por pouco tempo	297	22,6	22,6	70,6
	Aplicou-se consideravelmente, boa parte do tempo	202	15,4	15,4	86,0
	Aplicou-se muito, a maior parte do tempo	184	14,0	14,0	100,0
	Total	1313	100,0	100,0	

Senti que estava um pouco emotivo(a)/sensível demais

Estatísticas

Senti que estava um pouco emotivo(a)/sensível demais

N	Válido	1313
	Omisso	0

Senti que estava um pouco emotivo(a)/sensível demais

		Frequência	Porcentagem	Porcentagem válida	Porcentagem acumulativa
Válido	Não se aplica a mim	201	15,3	15,3	15,3
	Aplicou-se em algum grau, por pouco tempo	398	30,3	30,3	45,6
	Aplicou-se consideravelmente, boa parte do tempo	405	30,8	30,8	76,5
	Aplicou-se muito, a maior parte do tempo	309	23,5	23,5	100,0
	Total	1313	100,0	100,0	

Sabia que meu coração estava alterado mesmo não tendo feito nenhum esforço físico (ex.: aumento da frequência cardíaca, disritmia cardíaca)

Estatísticas

Sabia que meu coração estava alterado mesmo não tendo feito nenhum esforço físico (ex.: aumento da frequência cardíaca, disritmia cardíaca)

N	Válido	1313
	Omisso	0

Sabia que meu coração estava alterado mesmo não tendo feito nenhum esforço físico (ex.: aumento da frequência cardíaca, disritmia cardíaca)

		Frequência	Porcentagem	Porcentagem válida	Porcentagem acumulativa
Válido	Não se aplica a mim	592	45,1	45,1	45,1
	Aplicou-se em algum grau, por pouco tempo	357	27,2	27,2	72,3
	Aplicou-se consideravelmente, boa parte do tempo	222	16,9	16,9	89,2
	Aplicou-se muito, a maior parte do tempo	142	10,8	10,8	100,0
	Total	1313	100,0	100,0	

Senti medo sem motivo

Estatísticas

Senti medo sem motivo

N	Válido	1313
	Omisso	0

Senti medo sem motivo

		Frequência	Porcentagem	Porcentagem válida	Porcentagem acumulativa
Válido	Não se aplica a mim	530	40,4	40,4	40,4
	Aplicou-se em algum grau, por pouco tempo	385	29,3	29,3	69,7
	Aplicou-se consideravelmente, boa parte do tempo	226	17,2	17,2	86,9
	Aplicou-se muito, a maior parte do tempo	172	13,1	13,1	100,0
	Total	1313	100,0	100,0	

Senti que a vida não tinha sentido

Estatísticas

Senti que a vida não tinha sentido

N	Válido	1313
	Omisso	0

Senti que a vida não tinha sentido

		Frequência	Porcentagem	Porcentagem válida	Porcentagem acumulativa
Válido	Não se aplica a mim	816	62,1	62,1	62,1
	Aplicou-se em algum grau, por pouco tempo	241	18,4	18,4	80,5
	Aplicou-se consideravelmente, boa parte do tempo	140	10,7	10,7	91,2
	Aplicou-se muito, a maior parte do tempo	116	8,8	8,8	100,0
	Total	1313	100,0	100,0	

```
FREQUENCIES VARIABLES=PHQ9_Q1 PHQ9_Q2 PHQ9_Q3 PHQ9_Q4 PHQ9_Q5 PHQ9_Q6 PHQ9_Q7 PHQ9_Q8
PHQ9_Q9
  /ORDER=VARIABLE.
```

Frequências

Observações

Saída criada		20-SEP-2020 11:28:17
Comentários		
Entrada	Dados	C:\Users\User\Documents\Pesquisa\Fellow\FellowGenData_V1.sav
	Conjunto de dados ativo	ConjuntodeDados1
	Filtro	<none>
	Ponderação	<none>
	Arquivo Dividido	<none>
	N de linhas em arquivo de dados de trabalho	1313

Tratamento de valor omisso	Definição de omisso	Os valores omissos definidos pelo usuário são tratados como omissos.
	Casos utilizados	As estatísticas estão baseadas em todos os casos com dados válidos.
Sintaxe		FREQUENCIES VARIABLES=PHQ9_Q1 PHQ9_Q2 PHQ9_Q3 PHQ9_Q4 PHQ9_Q5 PHQ9_Q6 PHQ9_Q7 PHQ9_Q8 PHQ9_Q9 /ORDER=VARIABLE.
Recursos	Tempo do processador	00:00:00,02
	Tempo decorrido	00:00:00,02

```
Nas últimas duas semanas, em qual proporção de tempo o(a) sr.(a) teve pouco interesse ou pouco prazer
em fazer as coisas?
```

Estatísticas

Nas últimas duas semanas, em qual proporção de tempo o(a) sr.(a) teve pouco interesse ou pouco prazer em fazer as coisas?

N	Válido	1313
	Omisso	0

Nas últimas duas semanas, em qual proporção de tempo o(a) sr.(a) teve pouco interesse ou pouco prazer em fazer as coisas?

		Frequência	Porcentagem	Porcentagem válida	Porcentagem acumulativa
Válido	Nenhum dia	214	16,3	16,3	16,3
	Menos de 1 semana	532	40,5	40,5	56,8
	1 semana ou mais	304	23,2	23,2	80,0
	Quase todos os dias	263	20,0	20,0	100,0
	Total	1313	100,0	100,0	

```
Nas últimas duas semanas, em qual proporção de tempo o(a) sr.(a) se sentiu para baixo, deprimido(a) ou
sem
```

Estatísticas

Nas últimas duas semanas, em qual proporção de tempo o(a) sr.(a) se sentiu para baixo, deprimido(a) ou sem

N	Válido	1313
	Omisso	0

Nas últimas duas semanas, em qual proporção de tempo o(a) sr.(a) se sentiu para baixo, deprimido(a) ou sem

		Frequência	Porcentagem	Porcentagem válida	Porcentagem acumulativa
Válido	Nenhum dia	287	21,9	21,9	21,9
	Menos de 1 semana	493	37,5	37,5	59,4
	1 semana ou mais	327	24,9	24,9	84,3
	Quase todos os dias	206	15,7	15,7	100,0
	Total	1313	100,0	100,0	

Nas últimas duas semanas, em qual proporção de tempo o(a) sr.(a) teve dificuldade para pegar no sono ou permanecer dormindo ou dormiu mais do que de costume?

Estatísticas

Nas últimas duas semanas, em qual proporção de tempo o(a) sr.(a) teve dificuldade para pegar no sono ou permanecer dormindo ou dormiu mais do que de costume?

N	Válido	1313
	Omisso	0

Nas últimas duas semanas, em qual proporção de tempo o(a) sr.(a) teve dificuldade para pegar no sono ou permanecer dormindo ou dormiu mais do que de costume?

		Frequência	Porcentagem	Porcentagem válida	Porcentagem acumulativa
Válido	Nenhum dia	251	19,1	19,1	19,1
	Menos de 1 semana	338	25,7	25,7	44,9
	1 semana ou mais	353	26,9	26,9	71,7
	Quase todos os dias	371	28,3	28,3	100,0
	Total	1313	100,0	100,0	

Nas últimas duas semanas, em qual proporção de tempo o(a) sr.(a) se sentiu cansado(a) ou com pouca energia?

Estatísticas

Nas últimas duas semanas, em qual proporção de tempo o(a) sr.(a) se sentiu cansado(a) ou com pouca energia?

N	Válido	1313
	Omisso	0

Nas últimas duas semanas, em qual proporção de tempo o(a) sr.(a) se sentiu cansado(a) ou com pouca energia?

		Frequência	Porcentagem	Porcentagem válida	Porcentagem acumulativa
Válido	Nenhum dia	79	6,0	6,0	6,0
	Menos de 1 semana	320	24,4	24,4	30,4
	1 semana ou mais	410	31,2	31,2	61,6
	Quase todos os dias	504	38,4	38,4	100,0
	Total	1313	100,0	100,0	

Nas últimas duas semanas, em qual proporção de tempo o(a) sr.(a) teve falta de apetite ou comeu demais?

Estatísticas

Nas últimas duas semanas, em qual proporção de tempo o(a) sr.(a) teve falta de apetite ou comeu demais?

N	Válido	1313
	Omisso	0

Nas últimas duas semanas, em qual proporção de tempo o(a) sr.(a) teve falta de apetite ou comeu demais?

		Frequência	Porcentagem	Porcentagem válida	Porcentagem acumulativa
Válido	Nenhum dia	274	20,9	20,9	20,9
	Menos de 1 semana	343	26,1	26,1	47,0
	1 semana ou mais	335	25,5	25,5	72,5
	Quase todos os dias	361	27,5	27,5	100,0
	Total	1313	100,0	100,0	

```
Nas últimas duas semanas, em qual proporção de tempo o(a) sr.(a ) se sentiu mal consigo mesmo(a) ou achou
que é um fracasso ou que decepcionou sua família ou a você mesmo(a)?
```

Estatísticas

Nas últimas duas semanas, em qual proporção de tempo o(a) sr.(a) se sentiu mal consigo mesmo(a) ou achou que é um fracasso ou que decepcionou sua família ou a você mesmo(a)?

N	Válido	1313
	Omisso	0

Nas últimas duas semanas, em qual proporção de tempo o(a) sr.(a) se sentiu mal consigo mesmo(a) ou achou que é um fracasso ou que decepcionou sua família ou a você mesmo(a)?

		Frequência	Porcentagem	Porcentagem válida	Porcentagem acumulativa
Válido	Nenhum dia	445	33,9	33,9	33,9
	Menos de 1 semana	382	29,1	29,1	63,0
	1 semana ou mais	273	20,8	20,8	83,8
	Quase todos os dias	213	16,2	16,2	100,0
	Total	1313	100,0	100,0	

```
Nas últimas duas semanas, em qual proporção de tempo o(a) sr.(a) teve dificuldade para se concentrar
nas coisas (como ler o jornal ou ver televisão)?
```

Estatísticas

Nas últimas duas semanas, em qual proporção de tempo o(a) sr.(a) teve dificuldade para se concentrar nas coisas (como ler o jornal ou ver televisão)?

N	Válido	1313
	Omisso	0

Nas últimas duas semanas, em qual proporção de tempo o(a) sr.(a) teve dificuldade para se concentrar nas coisas (como ler o jornal ou ver televisão)?

		Frequência	Porcentagem	Porcentagem válida	Porcentagem acumulativa
Válido	Nenhum dia	219	16,7	16,7	16,7
	Menos de 1 semana	407	31,0	31,0	47,7

	1 semana ou mais	397	30,2	30,2	77,9
	Quase todos os dias	290	22,1	22,1	100,0
	Total	1313	100,0	100,0	

```
Nas últimas duas semanas, em qual proporção de tempo o(a) sr.(a) teve lentidão para se movimentar ou
falar (a ponto das outras pessoas perceberem), ou ao contrário, esteve tão agitado(a) que você ...
```

Estatísticas

Nas últimas duas semanas, em qual proporção de tempo o(a) sr.(a) teve lentidão para se movimentar ou falar (a ponto das outras pessoas perceberem), ou ao contrário, esteve tão agitado(a) que você ...

N	Válido	1313
	Omisso	0

Nas últimas duas semanas, em qual proporção de tempo o(a) sr.(a) teve lentidão para se movimentar ou falar (a ponto das outras pessoas perceberem), ou ao contrário, esteve tão agitado(a) que você ...

		Frequência	Porcentagem	Porcentagem válida	Porcentagem acumulativa
Válido	Nenhum dia	619	47,1	47,1	47,1
	Menos de 1 semana	352	26,8	26,8	74,0
	1 semana ou mais	217	16,5	16,5	90,5
	Quase todos os dias	125	9,5	9,5	100,0
	Total	1313	100,0	100,0	

```
Nas últimas duas semanas,  em qual proporção de tempo o(a) sr.(a) pensou em se ferir de alguma maneira
ou que seria melhor estar morto(a)?
```

Estatísticas

Nas últimas duas semanas, em qual proporção de tempo o(a) sr.(a) pensou em se ferir de alguma maneira ou que seria melhor estar morto(a)?

N	Válido	1313
	Omisso	0

Nas últimas duas semanas, em qual proporção de tempo o(a) sr.(a) pensou em se ferir de alguma maneira ou que seria melhor estar morto(a)?

		Frequência	Porcentagem	Porcentagem válida	Porcentagem acumulativa
Válido	Nenhum dia	1081	82,3	82,3	82,3
	Menos de 1 semana	143	10,9	10,9	93,2
	1 semana ou mais	52	4,0	4,0	97,2
	Quase todos os dias	37	2,8	2,8	100,0
	Total	1313	100,0	100,0	

```
FREQUENCIES VARIABLES=BRCS_Q1 BRCS_Q2 BRCS_Q3 BRCS_Q4
  /ORDER=VARIABLE.
```

Frequências

Observações

Saída criada		20-SEP-2020 11:28:41
Comentários		
Entrada	Dados	C:\Users\User\Documents\Pesquisa\Fellow\FellowGenData_V1.sav
	Conjunto de dados ativo	ConjuntodeDados1
	Filtro	<none>
	Ponderação	<none>
	Arquivo Dividido	<none>
	N de linhas em arquivo de dados de trabalho	1313
Tratamento de valor omisso	Definição de omisso	Os valores omissos definidos pelo usuário são tratados como omissos.
	Casos utilizados	As estatísticas estão baseadas em todos os casos com dados válidos.
Sintaxe		FREQUENCIES VARIABLES=BRCS_Q1 BRCS_Q2 BRCS_Q3 BRCS_Q4 /ORDER=VARIABLE.
Recursos	Tempo do processador	00:00:00,02
	Tempo decorrido	00:00:00,02

Procuro formas criativas de superar situações difíceis

Estatísticas

Procuro formas criativas de superar situações difíceis

N	Válido	1313
	Omisso	0

Procuro formas criativas de superar situações difíceis

		Frequência	Porcentagem	Porcentagem válida	Porcentagem acumulativa
Válido	Quase nunca	163	12,4	12,4	12,4
	Ocasionalmente	404	30,8	30,8	43,2
	Muitas vezes	373	28,4	28,4	71,6
	Com muita frequência	200	15,2	15,2	86,8
	Quase sempre	173	13,2	13,2	100,0
	Total	1313	100,0	100,0	

Independentemente do que me possa acontecer acredito que posso controlar

Estatísticas

Independentemente do que me possa acontecer acredito que posso controlar

N	Válido	1313
	Omisso	0

Independentemente do que me possa acontecer acredito que posso controlar

		Frequência	Porcentagem	Porcentagem válida	Porcentagem acumulativa
Válido	Quase nunca	108	8,2	8,2	8,2
	Ocasionalmente	346	26,4	26,4	34,6
	Muitas vezes	438	33,4	33,4	67,9
	Com muita frequência	253	19,3	19,3	87,2
	Quase sempre	168	12,8	12,8	100,0
	Total	1313	100,0	100,0	

Acredito que posso crescer positivamente lidando com situações difíceis.

Estatísticas

Acredito que posso crescer positivamente lidando com situações difíceis.

N	Válido	1313
	Omisso	0

Acredito que posso crescer positivamente lidando com situações difíceis.

		Frequência	Porcentagem	Porcentagem válida	Porcentagem acumulativa
Válido	Quase nunca	64	4,9	4,9	4,9
	Ocasionalmente	215	16,4	16,4	21,2
	Muitas vezes	380	28,9	28,9	50,2
	Com muita frequência	327	24,9	24,9	75,1
	Quase sempre	327	24,9	24,9	100,0
	Total	1313	100,0	100,0	

```
Procuro ativamente formas de substituir as perdas que encontro na vida.
```

Estatísticas

Procuro ativamente formas de substituir as perdas que encontro na vida.

N	Válido	1313
	Omisso	0

Procuro ativamente formas de substituir as perdas que encontro na vida.

		Frequência	Porcentagem	Porcentagem válida	Porcentagem acumulativa
Válido	Quase nunca	136	10,4	10,4	10,4
	Ocasionalmente	335	25,5	25,5	35,9
	Muitas vezes	366	27,9	27,9	63,7
	Com muita frequência	283	21,6	21,6	85,3
	Quase sempre	193	14,7	14,7	100,0
	Total	1313	100,0	100,0	

```
FREQUENCIES VARIABLES=OLBI_Q1D OLBI_Q2D OLBI_Q3D OLBI_Q4D OLBI_Q5D OLBI_Q6D OLBI_Q7D
OLBI_Q8D
    OLBI_Q1E OLBI_Q2E OLBI_Q3E OLBI_Q4E OLBI_Q5E OLBI_Q6E OLBI_Q7E OLBI_Q8E
  /ORDER=VARIABLE.
```

Frequências

Observações

Saída criada		20-SEP-2020 11:29:18
Comentários		
Entrada	Dados	C:\Users\User\Documents\Pesquisa\Fellow\FellowGenData_V1.sav
	Conjunto de dados ativo	ConjuntodeDados1
	Filtro	<none>
	Ponderação	<none>
	Arquivo Dividido	<none>
	N de linhas em arquivo de dados de trabalho	1313
Tratamento de valor omisso	Definição de omisso	Os valores omissos definidos pelo usuário são tratados como omissos.
	Casos utilizados	As estatísticas estão baseadas em todos os casos com dados válidos.
Sintaxe		FREQUENCIES VARIABLES=OLBI_Q1D OLBI_Q2D OLBI_Q3D OLBI_Q4D OLBI_Q5D OLBI_Q6D OLBI_Q7D OLBI_Q8D OLBI_Q1E OLBI_Q2E OLBI_Q3E OLBI_Q4E OLBI_Q5E OLBI_Q6E OLBI_Q7E OLBI_Q8E /ORDER=VARIABLE.
Recursos	Tempo do processador	00:00:00,02
	Tempo decorrido	00:00:00,02

Encontro com frequência assuntos novos e interessantes no meu trabalho (R)

Estatísticas

Encontro com frequência assuntos novos e interessantes no meu trabalho (R)

N	Válido	1313
	Omisso	0

Encontro com frequência assuntos novos e interessantes no meu trabalho (R)

		Frequência	Porcentagem	Porcentagem válida	Porcentagem acumulativa
Válido	Discordo totalmente	201	15,3	15,3	15,3
	Discordo	559	42,6	42,6	57,9
	Nem concordo, nem discordo	330	25,1	25,1	83,0
	Concordo	159	12,1	12,1	95,1
	Concordo totalmente	64	4,9	4,9	100,0
	Total	1313	100,0	100,0	

Cada vez mais falo de forma negativa do meu trabalho

Estatísticas

Cada vez mais falo de forma negativa do meu trabalho

N	Válido	1313
	Omisso	0

Cada vez mais falo de forma negativa do meu trabalho

		Frequência	Porcentagem	Porcentagem válida	Porcentagem acumulativa
Válido	Discordo totalmente	263	20,0	20,0	20,0
	Discordo	352	26,8	26,8	46,8
	Nem concordo, nem discordo	293	22,3	22,3	69,2
	Concordo	219	16,7	16,7	85,8
	Concordo totalmente	186	14,2	14,2	100,0
	Total	1313	100,0	100,0	

Ultimamente tenho pensado menos no meu trabalho e faço as tarefas de forma quase mecânica

Estatísticas

Ultimamente tenho pensado menos no meu trabalho e faço as tarefas de forma quase mecânica

N	Válido	1313
	Omisso	0

Ultimamente tenho pensado menos no meu trabalho e faço as tarefas de forma quase mecânica

		Frequência	Porcentagem	Porcentagem válida	Porcentagem acumulativa
Válido	Discordo totalmente	248	18,9	18,9	18,9
	Discordo	369	28,1	28,1	47,0
	Nem concordo, nem discordo	275	20,9	20,9	67,9
	Concordo	265	20,2	20,2	88,1
	Concordo totalmente	156	11,9	11,9	100,0
	Total	1313	100,0	100,0	

Considero que o meu trabalho é um desafio positivo (R)

Estatísticas

Considero que o meu trabalho é um desafio positivo (R)

N	Válido	1313
	Omisso	0

Considero que o meu trabalho é um desafio positivo (R)

		Frequência	Porcentagem	Porcentagem válida	Porcentagem acumulativa
Válido	Discordo totalmente	316	24,1	24,1	24,1
	Discordo	542	41,3	41,3	65,3
	Nem concordo, nem discordo	296	22,5	22,5	87,9
	Concordo	111	8,5	8,5	96,3
	Concordo totalmente	48	3,7	3,7	100,0
	Total	1313	100,0	100,0	

Com o passar do tempo, sinto-me desligado do meu trabalho

Estatísticas

Com o passar do tempo, sinto-me desligado do meu trabalho

N	Válido	1313
	Omisso	0

Com o passar do tempo, sinto-me desligado do meu trabalho

		Frequência	Porcentagem	Porcentagem válida	Porcentagem acumulativa
Válido	Discordo totalmente	346	26,4	26,4	26,4
	Discordo	369	28,1	28,1	54,5
	Nem concordo, nem discordo	289	22,0	22,0	76,5
	Concordo	211	16,1	16,1	92,5
	Concordo totalmente	98	7,5	7,5	100,0
	Total	1313	100,0	100,0	

```
Às vezes, sinto-me farto das minhas tarefas no trabalho
```

Estatísticas

Às vezes, sinto-me farto das minhas tarefas no trabalho

N	Válido	1313
	Omisso	0

Às vezes, sinto-me farto das minhas tarefas no trabalho

		Frequência	Porcentagem	Porcentagem válida	Porcentagem acumulativa
Válido	Discordo totalmente	135	10,3	10,3	10,3
	Discordo	201	15,3	15,3	25,6
	Nem concordo, nem discordo	229	17,4	17,4	43,0
	Concordo	456	34,7	34,7	77,8
	Concordo totalmente	292	22,2	22,2	100,0
	Total	1313	100,0	100,0	

```
Este é o único tipo de trabalho que me imagino a fazer (R)
```

Estatísticas

Este é o único tipo de trabalho que me imagino a fazer (R)

N	Válido	1313
	Omisso	0

Este é o único tipo de trabalho que me imagino a fazer (R)

		Frequência	Porcentagem	Porcentagem válida	Porcentagem acumulativa
Válido	Discordo totalmente	246	18,7	18,7	18,7
	Discordo	281	21,4	21,4	40,1
	Nem concordo, nem discordo	290	22,1	22,1	62,2
	Concordo	269	20,5	20,5	82,7
	Concordo totalmente	227	17,3	17,3	100,0
	Total	1313	100,0	100,0	

Sinto-me cada vez mais empenhado no meu trabalho (R)

Estatísticas

Sinto-me cada vez mais empenhado no meu trabalho (R)

N	Válido	1313
	Omisso	0

Sinto-me cada vez mais empenhado no meu trabalho (R)

		Frequência	Porcentagem	Porcentagem válida	Porcentagem acumulativa
Válido	Discordo totalmente	124	9,4	9,4	9,4
	Discordo	325	24,8	24,8	34,2
	Nem concordo, nem discordo	452	34,4	34,4	68,6
	Concordo	274	20,9	20,9	89,5
	Concordo totalmente	138	10,5	10,5	100,0
	Total	1313	100,0	100,0	

Há dias em que me sinto cansado antes mesmo de chegar ao trabalho

Estatísticas

Há dias em que me sinto cansado antes mesmo de chegar ao trabalho

N	Válido	1313
	Omisso	0

Há dias em que me sinto cansado antes mesmo de chegar ao trabalho

		Frequência	Porcentagem	Porcentagem válida	Porcentagem acumulativa
Válido	Discordo totalmente	48	3,7	3,7	3,7
	Discordo	99	7,5	7,5	11,2
	Nem concordo, nem discordo	123	9,4	9,4	20,6
	Concordo	492	37,5	37,5	58,0
	Concordo totalmente	551	42,0	42,0	100,0
	Total	1313	100,0	100,0	

```
Depois do trabalho, preciso de mais tempo para relaxar e sentir-me melhor do que precisava antigamente
```

Estatísticas

Depois do trabalho, preciso de mais tempo para relaxar e sentir-me melhor do que precisava antigamente

N	Válido	1313
	Omisso	0

Depois do trabalho, preciso de mais tempo para relaxar e sentir-me melhor do que precisava antigamente

		Frequência	Porcentagem	Porcentagem válida	Porcentagem acumulativa
Válido	Discordo totalmente	51	3,9	3,9	3,9
	Discordo	104	7,9	7,9	11,8
	Nem concordo, nem discordo	153	11,7	11,7	23,5
	Concordo	444	33,8	33,8	57,3
	Concordo totalmente	561	42,7	42,7	100,0
	Total	1313	100,0	100,0	

```
Consigo aguentar bem a pressão do meu trabalho (R)
```

Estatísticas

Consigo aguentar bem a pressão do meu trabalho (R)

N	Válido	1313
	Omisso	0

Consigo aguentar bem a pressão do meu trabalho (R)

		Frequência	Porcentagem	Porcentagem válida	Porcentagem acumulativa
Válido	Discordo totalmente	108	8,2	8,2	8,2
	Discordo	456	34,7	34,7	43,0
	Nem concordo, nem discordo	417	31,8	31,8	74,7
	Concordo	242	18,4	18,4	93,1
	Concordo totalmente	90	6,9	6,9	100,0
	Total	1313	100,0	100,0	

Durante o meu trabalho, muitas vezes sinto-me emocionalmente esgotado

Estatísticas

Durante o meu trabalho, muitas vezes sinto-me emocionalmente esgotado

N	Válido	1313
	Omisso	0

Durante o meu trabalho, muitas vezes sinto-me emocionalmente esgotado

		Frequência	Porcentagem	Porcentagem válida	Porcentagem acumulativa
Válido	Discordo totalmente	53	4,0	4,0	4,0
	Discordo	160	12,2	12,2	16,2
	Nem concordo, nem discordo	226	17,2	17,2	33,4
	Concordo	475	36,2	36,2	69,6
	Concordo totalmente	399	30,4	30,4	100,0
	Total	1313	100,0	100,0	

Depois do trabalho, tenho energia suficiente para minhas atividades de lazer (R)

Estatísticas

Depois do trabalho, tenho energia suficiente para minhas atividades de lazer (R)

N	Válido	1313
	Omisso	0

Depois do trabalho, tenho energia suficiente para minhas atividades de lazer (R)

		Frequência	Porcentagem	Porcentagem válida	Porcentagem acumulativa
Válido	Discordo totalmente	40	3,0	3,0	3,0
	Discordo	158	12,0	12,0	15,1
	Nem concordo, nem discordo	247	18,8	18,8	33,9
	Concordo	536	40,8	40,8	74,7
	Concordo totalmente	332	25,3	25,3	100,0
	Total	1313	100,0	100,0	

```
Depois do trabalho sinto-me cansado e sem energia
```

Estatísticas

Depois do trabalho sinto-me cansado e sem energia

N	Válido	1313
	Omisso	0

Depois do trabalho sinto-me cansado e sem energia

		Frequência	Porcentagem	Porcentagem válida	Porcentagem acumulativa
Válido	Discordo totalmente	59	4,5	4,5	4,5
	Discordo	129	9,8	9,8	14,3
	Nem concordo, nem discordo	183	13,9	13,9	28,3
	Concordo	477	36,3	36,3	64,6
	Concordo totalmente	465	35,4	35,4	100,0
	Total	1313	100,0	100,0	

```
De uma forma geral, consigo administrar bem a quantidade de trabalho que tenho (R)
```

Estatísticas

De uma forma geral, consigo administrar bem a quantidade de trabalho que tenho (R)

N	Válido	1313
	Omisso	0

De uma forma geral, consigo administrar bem a quantidade de trabalho que tenho (R)

		Frequência	Porcentagem	Porcentagem válida	Porcentagem acumulativa
Válido	Discordo totalmente	77	5,9	5,9	5,9
	Discordo	367	28,0	28,0	33,8
	Nem concordo, nem discordo	412	31,4	31,4	65,2
	Concordo	339	25,8	25,8	91,0
	Concordo totalmente	118	9,0	9,0	100,0
	Total	1313	100,0	100,0	

Quando trabalho, geralmente sinto-me com energia (R)

Estatísticas

Quando trabalho, geralmente sinto-me com energia (R)

N	Válido	1032
	Omisso	281

Quando trabalho, geralmente sinto-me com energia (R)

		Frequência	Porcentagem	Porcentagem válida	Porcentagem acumulativa
Válido	Discordo totalmente	46	3,5	4,5	4,5
	Discordo	366	27,9	35,5	39,9
	Nem concordo, nem discordo	290	22,1	28,1	68,0
	Concordo	277	21,1	26,8	94,9
	Concordo totalmente	53	4,0	5,1	100,0
	Total	1032	78,6	100,0	
Omisso	Sistema	281	21,4		
Total		1313	100,0		

```
CROSSTABS
  /TABLES=Sex Race ComorbAny HRiskFactor UnivHosp Autonomy_Classif PedagStr_Classif
    PPEAvail_Classif Wkload_6060 Wkload_9090 ExternWork CovidCare BY DASS21_Classif_D2c
    DASS21_Classif_A2c DASS21_Classif_S2c PHQ9_Classif BRCS_Classif OLBI_Classif_2c
  /FORMAT=AVALUE TABLES
  /STATISTICS=CHISQ PHI RISK
  /CELLS=COUNT ROW TOTAL
  /COUNT ROUND CELL.
```

Tabulações cruzadas

Observações

Saída criada		20-SEP-2020 11:36:22
Comentários		
Entrada	Dados	C:\Users\User\Documents\Pesquisa\Fellow\FellowGenData_V1.sav
	Conjunto de dados ativo	ConjuntodeDados1
	Filtro	<none>
	Ponderação	<none>
	Arquivo Dividido	<none>
	N de linhas em arquivo de dados de trabalho	1313
Tratamento de valor omisso	Definição de omisso	Os valores omissos definidos pelo usuário são tratados como omissos.
	Casos utilizados	As estatísticas de cada tabela são baseadas em todos os casos com dados válidos na(s) amplitude(s) especificada(s) para todas as variáveis de cada tabela.
Sintaxe		CROSSTABS /TABLES=Sex Race ComorbAny HRiskFactor UnivHosp Autonomy_Classif PedagStr_Classif PPEAvail_Classif Wkload_6060 Wkload_9090 ExternWork CovidCare BY DASS21_Classif_D2c DASS21_Classif_A2c DASS21_Classif_S2c PHQ9_Classif BRCS_Classif OLBI_Classif_2c /FORMAT=AVALUE TABLES /STATISTICS=CHISQ PHI RISK /CELLS=COUNT ROW TOTAL /COUNT ROUND CELL.
Recursos	Tempo do processador	00:00:00,17
	Tempo decorrido	00:00:00,15
	Dimensões solicitadas	2
	Células disponíveis	524245

Sex * DASS21 Classification - Depression (dichotomous)

Crosstab

			DASS21 Classification - Depression (dichotomous)		
			Normal	Abnormal (elevated)	Total
Sex	Male	Contagem	195	90	285
		% em Sex	68,4%	31,6%	100,0%
		% do Total	14,9%	6,9%	21,8%
	Female	Contagem	673	352	1025
		% em Sex	65,7%	34,3%	100,0%
		% do Total	51,4%	26,9%	78,2%
Total		Contagem	868	442	1310
		% em Sex	66,3%	33,7%	100,0%
		% do Total	66,3%	33,7%	100,0%

Testes qui-quadrado

	Valor	gl	Significância Assintótica (Bilateral)	Sig exata (2 lados)	Sig exata (1 lado)
Qui-quadrado de Pearson	,761[a]	1	,383		
Correção de continuidade[b]	,643	1	,423		
Razão de verossimilhança	,767	1	,381		
Teste Exato de Fisher				,396	,212
Associação Linear por Linear	,761	1	,383		
N de Casos Válidos	1310				

a. 0 células (,0%) esperavam uma contagem menor que 5. A contagem mínima esperada é 96,16.

b. Computado apenas para uma tabela 2x2

Medidas Simétricas

		Valor	Significância Aproximada
Nominal por Nominal	Fi	,024	,383
	V de Cramer	,024	,383
N de Casos Válidos		1310	

Estimativa de Risco

	Valor	Intervalo de confiança de 95% Inferior	Superior
Razão de Chances para Sex (Male / Female)	1,133	,856	1,501

Para grupo DASS21 Classification - Depression (dichotomous) = Normal	1,042	,952	1,141
Para grupo DASS21 Classification - Depression (dichotomous) = Abnormal (elevated)	,920	,760	1,113
N de Casos Válidos	1310		

```
Sex * DASS21 Classification - Anxiety (dichotomous)
```

Crosstab

			DASS21 Classification - Anxiety (dichotomous)		
			Normal	Abnormal (elevated)	Total
Sex	Male	Contagem	209	76	285
		% em Sex	73,3%	26,7%	100,0%
		% do Total	16,0%	5,8%	21,8%
	Female	Contagem	649	376	1025
		% em Sex	63,3%	36,7%	100,0%
		% do Total	49,5%	28,7%	78,2%
Total		Contagem	858	452	1310
		% em Sex	65,5%	34,5%	100,0%
		% do Total	65,5%	34,5%	100,0%

Testes qui-quadrado

	Valor	gl	Significância Assintótica (Bilateral)	Sig exata (2 lados)	Sig exata (1 lado)
Qui-quadrado de Pearson	9,900[a]	1	,002		
Correção de continuidade[b]	9,462	1	,002		
Razão de verossimilhança	10,213	1	,001		
Teste Exato de Fisher				,002	,001
Associação Linear por Linear	9,892	1	,002		
N de Casos Válidos	1310				

a. 0 células (,0%) esperavam uma contagem menor que 5. A contagem mínima esperada é 98,34.

b. Computado apenas para uma tabela 2x2

Medidas Simétricas

		Valor	Significância Aproximada
Nominal por Nominal	Fi	,087	,002
	V de Cramer	,087	,002
N de Casos Válidos		1310	

Estimativa de Risco

	Valor	Intervalo de confiança de 95% Inferior	Superior
Razão de Chances para Sex (Male / Female)	1,593	1,190	2,133
Para grupo DASS21 Classification - Anxiety (dichotomous) = Normal	1,158	1,065	1,260
Para grupo DASS21 Classification - Anxiety (dichotomous) = Abnormal (elevated)	,727	,590	,896
N de Casos Válidos	1310		

```
Sex * DASS21 Classification - Stress (dichotomous)
```

Crosstab

			DASS21 Classification - Stress (dichotomous)		
			Normal	Abnormal (elevated)	Total
Sex	Male	Contagem	236	49	285
		% em Sex	82,8%	17,2%	100,0%
		% do Total	18,0%	3,7%	21,8%
	Female	Contagem	773	252	1025
		% em Sex	75,4%	24,6%	100,0%
		% do Total	59,0%	19,2%	78,2%
Total		Contagem	1009	301	1310
		% em Sex	77,0%	23,0%	100,0%
		% do Total	77,0%	23,0%	100,0%

Testes qui-quadrado

	Valor	gl	Significância Assintótica (Bilateral)	Sig exata (2 lados)	Sig exata (1 lado)
Qui-quadrado de Pearson	6,886[a]	1	,009		

Correção de continuidade[b]	6,474	1	,011		
Razão de verossimilhança	7,232	1	,007		
Teste Exato de Fisher				,009	,005
Associação Linear por Linear	6,880	1	,009		
N de Casos Válidos	1310				

a. 0 células (,0%) esperavam uma contagem menor que 5. A contagem mínima esperada é 65,48.

b. Computado apenas para uma tabela 2x2

Medidas Simétricas

		Valor	Significância Aproximada
Nominal por Nominal	Fi	,073	,009
	V de Cramer	,073	,009
N de Casos Válidos		1310	

Estimativa de Risco

	Valor	Intervalo de confiança de 95%	
		Inferior	Superior
Razão de Chances para Sex (Male / Female)	1,570	1,119	2,204
Para grupo DASS21 Classification - Stress (dichotomous) = Normal	1,098	1,031	1,170
Para grupo DASS21 Classification - Stress (dichotomous) = Abnormal (elevated)	,699	,530	,922
N de Casos Válidos	1310		

Sex * PHQ9 Depression Classification - Risk estimate for having current major depressive disorder

Crosstab

			PHQ9 Depression Classification - Risk estimate for having current major depressive disorder		
			Low	High	Total
Sex	Male	Contagem	117	168	285
		% em Sex	41,1%	58,9%	100,0%
		% do Total	8,9%	12,8%	21,8%
	Female	Contagem	311	714	1025
		% em Sex	30,3%	69,7%	100,0%
		% do Total	23,7%	54,5%	78,2%

Total	Contagem	428	882	1310
	% em Sex	32,7%	67,3%	100,0%
	% do Total	32,7%	67,3%	100,0%

Testes qui-quadrado

	Valor	gl	Significância Assintótica (Bilateral)	Sig exata (2 lados)	Sig exata (1 lado)
Qui-quadrado de Pearson	11,631[a]	1	,001		
Correção de continuidade[b]	11,149	1	,001		
Razão de verossimilhança	11,329	1	,001		
Teste Exato de Fisher				,001	,000
Associação Linear por Linear	11,622	1	,001		
N de Casos Válidos	1310				

a. 0 células (,0%) esperavam uma contagem menor que 5. A contagem mínima esperada é 93,11.

b. Computado apenas para uma tabela 2x2

Medidas Simétricas

		Valor	Significância Aproximada
Nominal por Nominal	Fi	,094	,001
	V de Cramer	,094	,001
N de Casos Válidos		1310	

Estimativa de Risco

	Valor	Intervalo de confiança de 95% Inferior	Superior
Razão de Chances para Sex (Male / Female)	1,599	1,219	2,097
Para grupo PHQ9 Depression Classification - Risk estimate for having current major depressive disorder = Low	1,353	1,145	1,599
Para grupo PHQ9 Depression Classification - Risk estimate for having current major depressive disorder = High	,846	,762	,940
N de Casos Válidos	1310		

Sex * BRCS Classification

Crosstab

			BRCS Classification		Total
			Low resilience	Moderate to High	
Sex	Male	Contagem	172	113	285
		% em Sex	60,4%	39,6%	100,0%
		% do Total	13,1%	8,6%	21,8%
	Female	Contagem	639	386	1025
		% em Sex	62,3%	37,7%	100,0%
		% do Total	48,8%	29,5%	78,2%
Total		Contagem	811	499	1310
		% em Sex	61,9%	38,1%	100,0%
		% do Total	61,9%	38,1%	100,0%

Testes qui-quadrado

	Valor	gl	Significância Assintótica (Bilateral)	Sig exata (2 lados)	Sig exata (1 lado)
Qui-quadrado de Pearson	,375[a]	1	,540		
Correção de continuidade[b]	,295	1	,587		
Razão de verossimilhança	,373	1	,541		
Teste Exato de Fisher				,581	,293
Associação Linear por Linear	,374	1	,541		
N de Casos Válidos	1310				

a. 0 células (,0%) esperavam uma contagem menor que 5. A contagem mínima esperada é 108,56.

b. Computado apenas para uma tabela 2x2

Medidas Simétricas

		Valor	Significância Aproximada
Nominal por Nominal	Fi	-,017	,540
	V de Cramer	,017	,540
N de Casos Válidos		1310	

Estimativa de Risco

	Valor	Intervalo de confiança de 95%	
		Inferior	Superior
Razão de Chances para Sex (Male / Female)	,919	,703	1,203
Para grupo BRCS Classification = Low resilience	,968	,871	1,076

Para grupo BRCS Classification = Moderate to High	1,053	,894	1,240
N de Casos Válidos	1310		

```
Sex * OLBI Score Classification
```

Crosstab

			OLBI Score Classification		
			Low to Moderate	High	Total
Sex	Male	Contagem	191	94	285
		% em Sex	67,0%	33,0%	100,0%
		% do Total	14,6%	7,2%	21,8%
	Female	Contagem	683	342	1025
		% em Sex	66,6%	33,4%	100,0%
		% do Total	52,1%	26,1%	78,2%
Total		Contagem	874	436	1310
		% em Sex	66,7%	33,3%	100,0%
		% do Total	66,7%	33,3%	100,0%

Testes qui-quadrado

	Valor	gl	Significância Assintótica (Bilateral)	Sig exata (2 lados)	Sig exata (1 lado)
Qui-quadrado de Pearson	,015[a]	1	,903		
Correção de continuidade[b]	,003	1	,960		
Razão de verossimilhança	,015	1	,903		
Teste Exato de Fisher				,943	,482
Associação Linear por Linear	,015	1	,903		
N de Casos Válidos	1310				

a. 0 células (,0%) esperavam uma contagem menor que 5. A contagem mínima esperada é 94,85.

b. Computado apenas para uma tabela 2x2

Medidas Simétricas

		Valor	Significância Aproximada
Nominal por Nominal	Fi	,003	,903
	V de Cramer	,003	,903
N de Casos Válidos		1310	

Estimativa de Risco

	Valor	Intervalo de confiança de 95% Inferior	Superior
Razão de Chances para Sex (Male / Female)	1,017	,770	1,345
Para grupo OLBI Score Classification = Low to Moderate	1,006	,917	1,103
Para grupo OLBI Score Classification = High	,989	,820	1,191
N de Casos Válidos	1310		

```
Race * DASS21 Classification - Depression (dichotomous)
```

Crosstab

			DASS21 Classification - Depression (dichotomous) Normal	Abnormal (elevated)	Total
Race	White	Contagem	531	247	778
		% em Race	68,3%	31,7%	100,0%
		% do Total	40,4%	18,8%	59,3%
	Pardo	Contagem	276	157	433
		% em Race	63,7%	36,3%	100,0%
		% do Total	21,0%	12,0%	33,0%
	Black	Contagem	52	29	81
		% em Race	64,2%	35,8%	100,0%
		% do Total	4,0%	2,2%	6,2%
	Other	Contagem	11	10	21
		% em Race	52,4%	47,6%	100,0%
		% do Total	0,8%	0,8%	1,6%
Total		Contagem	870	443	1313
		% em Race	66,3%	33,7%	100,0%
		% do Total	66,3%	33,7%	100,0%

Testes qui-quadrado

	Valor	gl	Significância Assintótica (Bilateral)
Qui-quadrado de Pearson	4,573[a]	3	,206
Razão de verossimilhança	4,482	3	,214
Associação Linear por Linear	3,835	1	,050
N de Casos Válidos	1313		

a. 0 células (,0%) esperavam uma contagem menor que 5. A contagem mínima esperada é 7,09.

Medidas Simétricas

		Valor	Significância Aproximada
Nominal por Nominal	Fi	,059	,206
	V de Cramer	,059	,206
N de Casos Válidos		1313	

Estimativa de Risco

	Valor
Razão de Chances para Race (White / Pardo)	[a]

a. Não é possível calcular as estatísticas de Estimativa de Risco. Elas são computadas apenas para uma tabela 2*2 sem células vazias.

```
Race * DASS21 Classification - Anxiety (dichotomous)
```

Crosstab

			DASS21 Classification - Anxiety (dichotomous)		
			Normal	Abnormal (elevated)	Total
Race	White	Contagem	521	257	778
		% em Race	67,0%	33,0%	100,0%
		% do Total	39,7%	19,6%	59,3%
	Pardo	Contagem	275	158	433
		% em Race	63,5%	36,5%	100,0%
		% do Total	20,9%	12,0%	33,0%
	Black	Contagem	49	32	81
		% em Race	60,5%	39,5%	100,0%
		% do Total	3,7%	2,4%	6,2%
	Other	Contagem	16	5	21
		% em Race	76,2%	23,8%	100,0%
		% do Total	1,2%	0,4%	1,6%
Total		Contagem	861	452	1313
		% em Race	65,6%	34,4%	100,0%
		% do Total	65,6%	34,4%	100,0%

Testes qui-quadrado

	Valor	gl	Significância Assintótica (Bilateral)
Qui-quadrado de Pearson	3,460[a]	3	,326
Razão de verossimilhança	3,504	3	,320
Associação Linear por Linear	,789	1	,374
N de Casos Válidos	1313		

a. 0 células (,0%) esperavam uma contagem menor que 5. A contagem mínima esperada é 7,23.

Medidas Simétricas

		Valor	Significância Aproximada
Nominal por Nominal	Fi	,051	,326
	V de Cramer	,051	,326
N de Casos Válidos		1313	

Estimativa de Risco

	Valor
Razão de Chances para Race (White / Pardo)	[a]

a. Não é possível calcular as estatísticas de Estimativa de Risco. Elas são computadas apenas para uma tabela 2*2 sem células vazias.

```
Race * DASS21 Classification - Stress (dichotomous)
```

Crosstab

			DASS21 Classification - Stress (dichotomous)		
			Normal	Abnormal (elevated)	Total
Race	White	Contagem	606	172	778
		% em Race	77,9%	22,1%	100,0%
		% do Total	46,2%	13,1%	59,3%
	Pardo	Contagem	328	105	433
		% em Race	75,8%	24,2%	100,0%

		% do Total	25,0%	8,0%	33,0%
	Black	Contagem	61	20	81
		% em Race	75,3%	24,7%	100,0%
		% do Total	4,6%	1,5%	6,2%
	Other	Contagem	17	4	21
		% em Race	81,0%	19,0%	100,0%
		% do Total	1,3%	0,3%	1,6%
Total		Contagem	1012	301	1313
		% em Race	77,1%	22,9%	100,0%
		% do Total	77,1%	22,9%	100,0%

Testes qui-quadrado

	Valor	gl	Significância Assintótica (Bilateral)
Qui-quadrado de Pearson	1,045[a]	3	,790
Razão de verossimilhança	1,048	3	,790
Associação Linear por Linear	,348	1	,555
N de Casos Válidos	1313		

a. 1 células (12,5%) esperavam uma contagem menor que 5. A contagem mínima esperada é 4,81.

Medidas Simétricas

		Valor	Significância Aproximada
Nominal por Nominal	Fi	,028	,790
	V de Cramer	,028	,790
N de Casos Válidos		1313	

Estimativa de Risco

	Valor
Razão de Chances para Race (White / Pardo)	[a]

a. Não é possível calcular as estatísticas de Estimativa de Risco. Elas são computadas apenas para uma tabela 2*2 sem células vazias.

Race * PHQ9 Depression Classification - Risk estimate for having current major depressive disorder

Crosstab

			PHQ9 Depression Classification - Risk estimate for having current major depressive disorder		
			Low	High	Total
Race	White	Contagem	247	531	778
		% em Race	31,7%	68,3%	100,0%
		% do Total	18,8%	40,4%	59,3%
	Pardo	Contagem	145	288	433
		% em Race	33,5%	66,5%	100,0%
		% do Total	11,0%	21,9%	33,0%
	Black	Contagem	31	50	81
		% em Race	38,3%	61,7%	100,0%
		% do Total	2,4%	3,8%	6,2%
	Other	Contagem	6	15	21
		% em Race	28,6%	71,4%	100,0%
		% do Total	0,5%	1,1%	1,6%
Total		Contagem	429	884	1313
		% em Race	32,7%	67,3%	100,0%
		% do Total	32,7%	67,3%	100,0%

Testes qui-quadrado

	Valor	gl	Significância Assintótica (Bilateral)
Qui-quadrado de Pearson	1,748[a]	3	,626
Razão de verossimilhança	1,723	3	,632
Associação Linear por Linear	,739	1	,390
N de Casos Válidos	1313		

a. 0 células (,0%) esperavam uma contagem menor que 5. A contagem mínima esperada é 6,86.

Medidas Simétricas

		Valor	Significância Aproximada
Nominal por Nominal	Fi	,036	,626
	V de Cramer	,036	,626
N de Casos Válidos		1313	

Estimativa de Risco

	Valor

Razão de Chances para Race (White / Pardo)	[a]

a. Não é possível calcular as estatísticas de Estimativa de Risco. Elas são computadas apenas para uma tabela 2*2 sem células vazias.

```
Race * BRCS Classification
```

Crosstab

			BRCS Classification		
			Low resilience	Moderate to High	Total
Race	White	Contagem	479	299	778
		% em Race	61,6%	38,4%	100,0%
		% do Total	36,5%	22,8%	59,3%
	Pardo	Contagem	268	165	433
		% em Race	61,9%	38,1%	100,0%
		% do Total	20,4%	12,6%	33,0%
	Black	Contagem	50	31	81
		% em Race	61,7%	38,3%	100,0%
		% do Total	3,8%	2,4%	6,2%
	Other	Contagem	16	5	21
		% em Race	76,2%	23,8%	100,0%
		% do Total	1,2%	0,4%	1,6%
Total		Contagem	813	500	1313
		% em Race	61,9%	38,1%	100,0%
		% do Total	61,9%	38,1%	100,0%

Testes qui-quadrado

	Valor	gl	Significância Assintótica (Bilateral)
Qui-quadrado de Pearson	1,856[a]	3	,603
Razão de verossimilhança	1,983	3	,576
Associação Linear por Linear	,505	1	,477
N de Casos Válidos	1313		

a. 0 células (,0%) esperavam uma contagem menor que 5. A contagem mínima esperada é 8,00.

Medidas Simétricas

		Valor	Significância Aproximada
Nominal por Nominal	Fi	,038	,603
	V de Cramer	,038	,603
N de Casos Válidos		1313	

Estimativa de Risco

	Valor
Razão de Chances para Race (White / Pardo)	[a]

a. Não é possível calcular as estatísticas de Estimativa de Risco. Elas são computadas apenas para uma tabela 2*2 sem células vazias.

```
Race * OLBI Score Classification
```

Crosstab

			OLBI Score Classification		
			Low to Moderate	High	Total
Race	White	Contagem	499	279	778
		% em Race	64,1%	35,9%	100,0%
		% do Total	38,0%	21,2%	59,3%
	Pardo	Contagem	305	128	433
		% em Race	70,4%	29,6%	100,0%
		% do Total	23,2%	9,7%	33,0%
	Black	Contagem	58	23	81
		% em Race	71,6%	28,4%	100,0%
		% do Total	4,4%	1,8%	6,2%
	Other	Contagem	13	8	21
		% em Race	61,9%	38,1%	100,0%
		% do Total	1,0%	0,6%	1,6%
Total		Contagem	875	438	1313
		% em Race	66,6%	33,4%	100,0%
		% do Total	66,6%	33,4%	100,0%

Testes qui-quadrado

	Valor	gl	Significância Assintótica (Bilateral)
Qui-quadrado de Pearson	6,110[a]	3	,106

Razão de verossimilhança	6,163	3	,104
Associação Linear por Linear	3,372	1	,066
N de Casos Válidos	1313		

a. 0 células (,0%) esperavam uma contagem menor que 5. A contagem mínima esperada é 7,01.

Medidas Simétricas

		Valor	Significância Aproximada
Nominal por Nominal	Fi	,068	,106
	V de Cramer	,068	,106
N de Casos Válidos		1313	

Estimativa de Risco

	Valor
Razão de Chances para Race (White / Pardo)	[a]

a. Não é possível calcular as estatísticas de Estimativa de Risco. Elas são computadas apenas para uma tabela 2*2 sem células vazias.

```
Any comorbidity (regardless COVID risk) * DASS21 Classification - Depression (dichotomous)
```

Crosstab

			DASS21 Classification - Depression (dichotomous)		
			Normal	Abnormal (elevated)	Total
Any comorbidity (regardless COVID risk)	No	Contagem	750	321	1071
		% em Any comorbidity (regardless COVID risk)	70,0%	30,0%	100,0%
		% do Total	57,5%	24,6%	82,1%
	Yes	Contagem	115	119	234
		% em Any comorbidity (regardless COVID risk)	49,1%	50,9%	100,0%
		% do Total	8,8%	9,1%	17,9%
Total		Contagem	865	440	1305
		% em Any comorbidity (regardless COVID risk)	66,3%	33,7%	100,0%
		% do Total	66,3%	33,7%	100,0%

Testes qui-quadrado

	Valor	gl	Significância Assintótica (Bilateral)	Sig exata (2 lados)	Sig exata (1 lado)
Qui-quadrado de Pearson	37,473[a]	1	,000		
Correção de continuidade[b]	36,545	1	,000		
Razão de verossimilhança	35,860	1	,000		
Teste Exato de Fisher				,000	,000
Associação Linear por Linear	37,444	1	,000		
N de Casos Válidos	1305				

a. 0 células (,0%) esperavam uma contagem menor que 5. A contagem mínima esperada é 78,90.

b. Computado apenas para uma tabela 2x2

Medidas Simétricas

		Valor	Significância Aproximada
Nominal por Nominal	Fi	,169	,000
	V de Cramer	,169	,000
N de Casos Válidos		1305	

Estimativa de Risco

	Valor	Intervalo de confiança de 95%	
		Inferior	Superior
Razão de Chances para Any comorbidity (regardless COVID risk) (No / Yes)	2,418	1,813	3,224
Para grupo DASS21 Classification - Depression (dichotomous) = Normal	1,425	1,244	1,633
Para grupo DASS21 Classification - Depression (dichotomous) = Abnormal (elevated)	,589	,504	,689
N de Casos Válidos	1305		

Any comorbidity (regardless COVID risk) * DASS21 Classification - Anxiety (dichotomous)

Crosstab

			DASS21 Classification - Anxiety (dichotomous)		Total
			Normal	Abnormal (elevated)	
Any comorbidity (regardless COVID risk)	No	Contagem	732	339	1071
		% em Any comorbidity (regardless COVID risk)	68,3%	31,7%	100,0%
		% do Total	56,1%	26,0%	82,1%
	Yes	Contagem	125	109	234
		% em Any comorbidity (regardless COVID risk)	53,4%	46,6%	100,0%
		% do Total	9,6%	8,4%	17,9%
Total		Contagem	857	448	1305
		% em Any comorbidity (regardless COVID risk)	65,7%	34,3%	100,0%
		% do Total	65,7%	34,3%	100,0%

Testes qui-quadrado

	Valor	gl	Significância Assintótica (Bilateral)	Sig exata (2 lados)	Sig exata (1 lado)
Qui-quadrado de Pearson	18,984[a]	1	,000		
Correção de continuidade[b]	18,328	1	,000		
Razão de verossimilhança	18,359	1	,000		
Teste Exato de Fisher				,000	,000
Associação Linear por Linear	18,970	1	,000		
N de Casos Válidos	1305				

a. 0 células (,0%) esperavam uma contagem menor que 5. A contagem mínima esperada é 80,33.

b. Computado apenas para uma tabela 2x2

Medidas Simétricas

		Valor	Significância Aproximada
Nominal por Nominal	Fi	,121	,000
	V de Cramer	,121	,000
N de Casos Válidos		1305	

Estimativa de Risco

	Valor	Intervalo de confiança de 95%	
		Inferior	Superior
Razão de Chances para Any comorbidity (regardless COVID risk) (No / Yes)	1,883	1,413	2,510

Para grupo DASS21 Classification - Anxiety (dichotomous) = Normal	1,279	1,128	1,452
Para grupo DASS21 Classification - Anxiety (dichotomous) = Abnormal (elevated)	,680	,577	,800
N de Casos Válidos	1305		

```
Any comorbidity (regardless COVID risk) * DASS21 Classification - Stress (dichotomous)
```

Crosstab

			DASS21 Classification - Stress (dichotomous)		
			Normal	Abnormal (elevated)	Total
Any comorbidity (regardless COVID risk)	No	Contagem	838	233	1071
		% em Any comorbidity (regardless COVID risk)	78,2%	21,8%	100,0%
		% do Total	64,2%	17,9%	82,1%
	Yes	Contagem	169	65	234
		% em Any comorbidity (regardless COVID risk)	72,2%	27,8%	100,0%
		% do Total	13,0%	5,0%	17,9%
Total		Contagem	1007	298	1305
		% em Any comorbidity (regardless COVID risk)	77,2%	22,8%	100,0%
		% do Total	77,2%	22,8%	100,0%

Testes qui-quadrado

	Valor	gl	Significância Assintótica (Bilateral)	Sig exata (2 lados)	Sig exata (1 lado)
Qui-quadrado de Pearson	3,953[a]	1	,047		
Correção de continuidade[b]	3,618	1	,057		
Razão de verossimilhança	3,814	1	,051		
Teste Exato de Fisher				,048	,030
Associação Linear por Linear	3,950	1	,047		
N de Casos Válidos	1305				

a. 0 células (,0%) esperavam uma contagem menor que 5. A contagem mínima esperada é 53,43.

b. Computado apenas para uma tabela 2x2

Medidas Simétricas

		Valor	Significância Aproximada
Nominal por Nominal	Fi	,055	,047
	V de Cramer	,055	,047
N de Casos Válidos		1305	

Estimativa de Risco

	Valor	Intervalo de confiança de 95% Inferior	Superior
Razão de Chances para Any comorbidity (regardless COVID risk) (No / Yes)	1,383	1,004	1,906
Para grupo DASS21 Classification - Stress (dichotomous) = Normal	1,083	,995	1,180
Para grupo DASS21 Classification - Stress (dichotomous) = Abnormal (elevated)	,783	,619	,991
N de Casos Válidos	1305		

```
Any comorbidity (regardless COVID risk) * PHQ9 Depression Classification - Risk estimate for having
current major depressive disorder
```

Crosstab

			PHQ9 Depression Classification - Risk estimate for having current major depressive disorder Low	High	Total
Any comorbidity (regardless COVID risk)	No	Contagem	383	688	1071
		% em Any comorbidity (regardless COVID risk)	35,8%	64,2%	100,0%
		% do Total	29,3%	52,7%	82,1%
	Yes	Contagem	45	189	234
		% em Any comorbidity (regardless COVID risk)	19,2%	80,8%	100,0%
		% do Total	3,4%	14,5%	17,9%
Total		Contagem	428	877	1305
		% em Any comorbidity (regardless COVID risk)	32,8%	67,2%	100,0%
		% do Total	32,8%	67,2%	100,0%

Testes qui-quadrado

	Valor	gl	Significância Assintótica (Bilateral)	Sig exata (2 lados)	Sig exata (1 lado)
Qui-quadrado de Pearson	23,808[a]	1	,000		
Correção de continuidade[b]	23,064	1	,000		
Razão de verossimilhança	25,669	1	,000		
Teste Exato de Fisher				,000	,000
Associação Linear por Linear	23,790	1	,000		
N de Casos Válidos	1305				

a. 0 células (,0%) esperavam uma contagem menor que 5. A contagem mínima esperada é 76,74.

b. Computado apenas para uma tabela 2x2

Medidas Simétricas

		Valor	Significância Aproximada
Nominal por Nominal	Fi	,135	,000
	V de Cramer	,135	,000
N de Casos Válidos		1305	

Estimativa de Risco

	Valor	Intervalo de confiança de 95% Inferior	Superior
Razão de Chances para Any comorbidity (regardless COVID risk) (No / Yes)	2,338	1,650	3,312
Para grupo PHQ9 Depression Classification - Risk estimate for having current major depressive disorder = Low	1,860	1,413	2,447
Para grupo PHQ9 Depression Classification - Risk estimate for having current major depressive disorder = High	,795	,737	,859
N de Casos Válidos	1305		

Any comorbidity (regardless COVID risk) * BRCS Classification

Crosstab

	BRCS Classification Low resilience	Moderate to High	Total

Any comorbidity (regardless COVID risk)	No	Contagem	656	415	1071
		% em Any comorbidity (regardless COVID risk)	61,3%	38,7%	100,0%
		% do Total	50,3%	31,8%	82,1%
	Yes	Contagem	154	80	234
		% em Any comorbidity (regardless COVID risk)	65,8%	34,2%	100,0%
		% do Total	11,8%	6,1%	17,9%
Total		Contagem	810	495	1305
		% em Any comorbidity (regardless COVID risk)	62,1%	37,9%	100,0%
		% do Total	62,1%	37,9%	100,0%

Testes qui-quadrado

	Valor	gl	Significância Assintótica (Bilateral)	Sig exata (2 lados)	Sig exata (1 lado)
Qui-quadrado de Pearson	1,697[a]	1	,193		
Correção de continuidade[b]	1,509	1	,219		
Razão de verossimilhança	1,715	1	,190		
Teste Exato de Fisher				,206	,109
Associação Linear por Linear	1,695	1	,193		
N de Casos Válidos	1305				

a. 0 células (,0%) esperavam uma contagem menor que 5. A contagem mínima esperada é 88,76.

b. Computado apenas para uma tabela 2x2

Medidas Simétricas

		Valor	Significância Aproximada
Nominal por Nominal	Fi	-,036	,193
	V de Cramer	,036	,193
N de Casos Válidos		1305	

Estimativa de Risco

	Valor	Intervalo de confiança de 95% Inferior	Superior
Razão de Chances para Any comorbidity (regardless COVID risk) (No / Yes)	,821	,610	1,105
Para grupo BRCS Classification = Low resilience	,931	,839	1,033
Para grupo BRCS Classification = Moderate to High	1,133	,934	1,375

N de Casos Válidos	1305		

Any comorbidity (regardless COVID risk) * OLBI Score Classification

Crosstab

			OLBI Score Classification		
			Low to Moderate	High	Total
Any comorbidity (regardless COVID risk)	No	Contagem	740	331	1071
		% em Any comorbidity (regardless COVID risk)	69,1%	30,9%	100,0%
		% do Total	56,7%	25,4%	82,1%
	Yes	Contagem	129	105	234
		% em Any comorbidity (regardless COVID risk)	55,1%	44,9%	100,0%
		% do Total	9,9%	8,0%	17,9%
Total		Contagem	869	436	1305
		% em Any comorbidity (regardless COVID risk)	66,6%	33,4%	100,0%
		% do Total	66,6%	33,4%	100,0%

Testes qui-quadrado

	Valor	gl	Significância Assintótica (Bilateral)	Sig exata (2 lados)	Sig exata (1 lado)
Qui-quadrado de Pearson	16,837[a]	1	,000		
Correção de continuidade[b]	16,215	1	,000		
Razão de verossimilhança	16,265	1	,000		
Teste Exato de Fisher				,000	,000
Associação Linear por Linear	16,824	1	,000		
N de Casos Válidos	1305				

a. 0 células (,0%) esperavam uma contagem menor que 5. A contagem mínima esperada é 78,18.

b. Computado apenas para uma tabela 2x2

Medidas Simétricas

		Valor	Significância Aproximada
Nominal por Nominal	Fi	,114	,000
	V de Cramer	,114	,000
N de Casos Válidos		1305	

Estimativa de Risco

	Valor	Intervalo de confiança de 95% Inferior	Superior
Razão de Chances para Any comorbidity (regardless COVID risk) (No / Yes)	1,820	1,364	2,428
Para grupo OLBI Score Classification = Low to Moderate	1,253	1,109	1,416
Para grupo OLBI Score Classification = High	,689	,582	,815
N de Casos Válidos	1305		

```
Risk factor(s) for severe COVID19 * DASS21 Classification - Depression (dichotomous)
```

Crosstab

			DASS21 Classification - Depression (dichotomous) Normal	Abnormal (elevated)	Total
Risk factor(s) for severe COVID19	No	Contagem	761	326	1087
		% em Risk factor(s) for severe COVID19	70,0%	30,0%	100,0%
		% do Total	58,3%	25,0%	83,3%
	Yes	Contagem	104	114	218
		% em Risk factor(s) for severe COVID19	47,7%	52,3%	100,0%
		% do Total	8,0%	8,7%	16,7%
Total		Contagem	865	440	1305
		% em Risk factor(s) for severe COVID19	66,3%	33,7%	100,0%
		% do Total	66,3%	33,7%	100,0%

Testes qui-quadrado

	Valor	gl	Significância Assintótica (Bilateral)	Sig exata (2 lados)	Sig exata (1 lado)
Qui-quadrado de Pearson	40,415[a]	1	,000		
Correção de continuidade[b]	39,423	1	,000		
Razão de verossimilhança	38,545	1	,000		
Teste Exato de Fisher				,000	,000
Associação Linear por Linear	40,384	1	,000		

N de Casos Válidos	1305				

a. 0 células (,0%) esperavam uma contagem menor que 5. A contagem mínima esperada é 73,50.

b. Computado apenas para uma tabela 2x2

Medidas Simétricas

		Valor	Significância Aproximada
Nominal por Nominal	Fi	,176	,000
	V de Cramer	,176	,000
N de Casos Válidos		1305	

Estimativa de Risco

	Valor	Intervalo de confiança de 95% Inferior	Superior
Razão de Chances para Risk factor(s) for severe COVID19 (No / Yes)	2,559	1,904	3,439
Para grupo DASS21 Classification - Depression (dichotomous) = Normal	1,468	1,270	1,695
Para grupo DASS21 Classification - Depression (dichotomous) = Abnormal (elevated)	,574	,491	,670
N de Casos Válidos	1305		

```
Risk factor(s) for severe COVID19 * DASS21 Classification - Anxiety (dichotomous)
```

Crosstab

			DASS21 Classification - Anxiety (dichotomous)		
			Normal	Abnormal (elevated)	Total
Risk factor(s) for severe COVID19	No	Contagem	741	346	1087
		% em Risk factor(s) for severe COVID19	68,2%	31,8%	100,0%
		% do Total	56,8%	26,5%	83,3%
	Yes	Contagem	116	102	218
		% em Risk factor(s) for severe COVID19	53,2%	46,8%	100,0%
		% do Total	8,9%	7,8%	16,7%

Total	Contagem	857	448	1305
	% em Risk factor(s) for severe COVID19	65,7%	34,3%	100,0%
	% do Total	65,7%	34,3%	100,0%

Testes qui-quadrado

	Valor	gl	Significância Assintótica (Bilateral)	Sig exata (2 lados)	Sig exata (1 lado)
Qui-quadrado de Pearson	18,022[a]	1	,000		
Correção de continuidade[b]	17,364	1	,000		
Razão de verossimilhança	17,405	1	,000		
Teste Exato de Fisher				,000	,000
Associação Linear por Linear	18,008	1	,000		
N de Casos Válidos	1305				

a. 0 células (,0%) esperavam uma contagem menor que 5. A contagem mínima esperada é 74,84.

b. Computado apenas para uma tabela 2x2

Medidas Simétricas

		Valor	Significância Aproximada
Nominal por Nominal	Fi	,118	,000
	V de Cramer	,118	,000
N de Casos Válidos		1305	

Estimativa de Risco

	Valor	Intervalo de confiança de 95% Inferior	Superior
Razão de Chances para Risk factor(s) for severe COVID19 (No / Yes)	1,883	1,402	2,529
Para grupo DASS21 Classification - Anxiety (dichotomous) = Normal	1,281	1,124	1,460
Para grupo DASS21 Classification - Anxiety (dichotomous) = Abnormal (elevated)	,680	,576	,803
N de Casos Válidos	1305		

```
Risk factor(s) for severe COVID19 * DASS21 Classification - Stress (dichotomous)
```

Crosstab

			DASS21 Classification - Stress (dichotomous)		
			Normal	Abnormal (elevated)	Total
Risk factor(s) for severe COVID19	No	Contagem	850	237	1087
		% em Risk factor(s) for severe COVID19	78,2%	21,8%	100,0%
		% do Total	65,1%	18,2%	83,3%
	Yes	Contagem	157	61	218
		% em Risk factor(s) for severe COVID19	72,0%	28,0%	100,0%
		% do Total	12,0%	4,7%	16,7%
Total		Contagem	1007	298	1305
		% em Risk factor(s) for severe COVID19	77,2%	22,8%	100,0%
		% do Total	77,2%	22,8%	100,0%

Testes qui-quadrado

	Valor	gl	Significância Assintótica (Bilateral)	Sig exata (2 lados)	Sig exata (1 lado)
Qui-quadrado de Pearson	3,934[a]	1	,047		
Correção de continuidade[b]	3,591	1	,058		
Razão de verossimilhança	3,787	1	,052		
Teste Exato de Fisher				,052	,031
Associação Linear por Linear	3,931	1	,047		
N de Casos Válidos	1305				

a. 0 células (,0%) esperavam uma contagem menor que 5. A contagem mínima esperada é 49,78.

b. Computado apenas para uma tabela 2x2

Medidas Simétricas

		Valor	Significância Aproximada
Nominal por Nominal	Fi	,055	,047
	V de Cramer	,055	,047
N de Casos Válidos		1305	

Estimativa de Risco

	Valor	Intervalo de confiança de 95%	
		Inferior	Superior

Razão de Chances para Risk factor(s) for severe COVID19 (No / Yes)	1,393	1,003	1,936
Para grupo DASS21 Classification - Stress (dichotomous) = Normal	1,086	,994	1,186
Para grupo DASS21 Classification - Stress (dichotomous) = Abnormal (elevated)	,779	,612	,991
N de Casos Válidos	1305		

```
Risk factor(s) for severe COVID19 * PHQ9 Depression Classification - Risk estimate for having current
major depressive disorder
```

Crosstab

			PHQ9 Depression Classification - Risk estimate for having current major depressive disorder		
			Low	High	Total
Risk factor(s) for severe COVID19	No	Contagem	385	702	1087
		% em Risk factor(s) for severe COVID19	35,4%	64,6%	100,0%
		% do Total	29,5%	53,8%	83,3%
	Yes	Contagem	43	175	218
		% em Risk factor(s) for severe COVID19	19,7%	80,3%	100,0%
		% do Total	3,3%	13,4%	16,7%
Total		Contagem	428	877	1305
		% em Risk factor(s) for severe COVID19	32,8%	67,2%	100,0%
		% do Total	32,8%	67,2%	100,0%

Testes qui-quadrado

	Valor	gl	Significância Assintótica (Bilateral)	Sig exata (2 lados)	Sig exata (1 lado)
Qui-quadrado de Pearson	20,291[a]	1	,000		
Correção de continuidade[b]	19,586	1	,000		
Razão de verossimilhança	21,828	1	,000		
Teste Exato de Fisher				,000	,000
Associação Linear por Linear	20,276	1	,000		
N de Casos Válidos	1305				

a. 0 células (,0%) esperavam uma contagem menor que 5. A contagem mínima esperada é 71,50.

b. Computado apenas para uma tabela 2x2

Medidas Simétricas

		Valor	Significância Aproximada
Nominal por Nominal	Fi	,125	,000
	V de Cramer	,125	,000
N de Casos Válidos		1305	

Estimativa de Risco

	Valor	Intervalo de confiança de 95%	
		Inferior	Superior
Razão de Chances para Risk factor(s) for severe COVID19 (No / Yes)	2,232	1,563	3,186
Para grupo PHQ9 Depression Classification - Risk estimate for having current major depressive disorder = Low	1,796	1,358	2,375
Para grupo PHQ9 Depression Classification - Risk estimate for having current major depressive disorder = High	,804	,743	,871
N de Casos Válidos	1305		

```
Risk factor(s) for severe COVID19 * BRCS Classification
```

Crosstab

			BRCS Classification		Total
			Low resilience	Moderate to High	
Risk factor(s) for severe COVID19	No	Contagem	665	422	1087
		% em Risk factor(s) for severe COVID19	61,2%	38,8%	100,0%
		% do Total	51,0%	32,3%	83,3%
	Yes	Contagem	145	73	218
		% em Risk factor(s) for severe COVID19	66,5%	33,5%	100,0%
		% do Total	11,1%	5,6%	16,7%
Total		Contagem	810	495	1305
		% em Risk factor(s) for severe COVID19	62,1%	37,9%	100,0%

% do Total	62,1%	37,9%	100,0%

Testes qui-quadrado

	Valor	gl	Significância Assintótica (Bilateral)	Sig exata (2 lados)	Sig exata (1 lado)
Qui-quadrado de Pearson	2,196[a]	1	,138		
Correção de continuidade[b]	1,975	1	,160		
Razão de verossimilhança	2,226	1	,136		
Teste Exato de Fisher				,147	,079
Associação Linear por Linear	2,195	1	,139		
N de Casos Válidos	1305				

a. 0 células (,0%) esperavam uma contagem menor que 5. A contagem mínima esperada é 82,69.

b. Computado apenas para uma tabela 2x2

Medidas Simétricas

		Valor	Significância Aproximada
Nominal por Nominal	Fi	-,041	,138
	V de Cramer	,041	,138
N de Casos Válidos		1305	

Estimativa de Risco

	Valor	Intervalo de confiança de 95% Inferior	Superior
Razão de Chances para Risk factor(s) for severe COVID19 (No / Yes)	,793	,584	1,078
Para grupo BRCS Classification = Low resilience	,920	,828	1,022
Para grupo BRCS Classification = Moderate to High	1,159	,948	1,418
N de Casos Válidos	1305		

```
Risk factor(s) for severe COVID19 * OLBI Score Classification
```

Crosstab

	OLBI Score Classification		Total
	Low to Moderate	High	

Risk factor(s) for severe COVID19	No	Contagem	752	335	1087
		% em Risk factor(s) for severe COVID19	69,2%	30,8%	100,0%
		% do Total	57,6%	25,7%	83,3%
	Yes	Contagem	117	101	218
		% em Risk factor(s) for severe COVID19	53,7%	46,3%	100,0%
		% do Total	9,0%	7,7%	16,7%
Total		Contagem	869	436	1305
		% em Risk factor(s) for severe COVID19	66,6%	33,4%	100,0%
		% do Total	66,6%	33,4%	100,0%

Testes qui-quadrado

	Valor	gl	Significância Assintótica (Bilateral)	Sig exata (2 lados)	Sig exata (1 lado)
Qui-quadrado de Pearson	19,638[a]	1	,000		
Correção de continuidade[b]	18,947	1	,000		
Razão de verossimilhança	18,892	1	,000		
Teste Exato de Fisher				,000	,000
Associação Linear por Linear	19,623	1	,000		
N de Casos Válidos	1305				

a. 0 células (,0%) esperavam uma contagem menor que 5. A contagem mínima esperada é 72,83.

b. Computado apenas para uma tabela 2x2

Medidas Simétricas

		Valor	Significância Aproximada
Nominal por Nominal	Fi	,123	,000
	V de Cramer	,123	,000
N de Casos Válidos		1305	

Estimativa de Risco

	Valor	Intervalo de confiança de 95% Inferior	Superior
Razão de Chances para Risk factor(s) for severe COVID19 (No / Yes)	1,938	1,442	2,605
Para grupo OLBI Score Classification = Low to Moderate	1,289	1,132	1,467
Para grupo OLBI Score Classification = High	,665	,562	,787

N de Casos Válidos	1305		

The institution is a university hospital * DASS21 Classification - Depression (dichotomous)

Crosstab

			DASS21 Classification - Depression (dichotomous)		Total
			Normal	Abnormal (elevated)	
The institution is a university hospital	No	Contagem	84	52	136
		% em The institution is a university hospital	61,8%	38,2%	100,0%
		% do Total	6,4%	4,0%	10,4%
	Yes	Contagem	786	391	1177
		% em The institution is a university hospital	66,8%	33,2%	100,0%
		% do Total	59,9%	29,8%	89,6%
Total		Contagem	870	443	1313
		% em The institution is a university hospital	66,3%	33,7%	100,0%
		% do Total	66,3%	33,7%	100,0%

Testes qui-quadrado

	Valor	gl	Significância Assintótica (Bilateral)	Sig exata (2 lados)	Sig exata (1 lado)
Qui-quadrado de Pearson	1,372[a]	1	,242		
Correção de continuidade[b]	1,156	1	,282		
Razão de verossimilhança	1,348	1	,246		
Teste Exato de Fisher				,251	,141
Associação Linear por Linear	1,371	1	,242		
N de Casos Válidos	1313				

a. 0 células (,0%) esperavam uma contagem menor que 5. A contagem mínima esperada é 45,89.

b. Computado apenas para uma tabela 2x2

Medidas Simétricas

		Valor	Significância Aproximada
Nominal por Nominal	Fi	-,032	,242
	V de Cramer	,032	,242

N de Casos Válidos	1313	

Estimativa de Risco

	Valor	Intervalo de confiança de 95% Inferior	Superior
Razão de Chances para The institution is a university hospital (No / Yes)	,804	,557	1,159
Para grupo DASS21 Classification - Depression (dichotomous) = Normal	,925	,805	1,062
Para grupo DASS21 Classification - Depression (dichotomous) = Abnormal (elevated)	1,151	,916	1,446
N de Casos Válidos	1313		

```
The institution is a university hospital * DASS21 Classification - Anxiety (dichotomous)
```

Crosstab

			DASS21 Classification - Anxiety (dichotomous)		
			Normal	Abnormal (elevated)	Total
The institution is a university hospital	No	Contagem	77	59	136
		% em The institution is a university hospital	56,6%	43,4%	100,0%
		% do Total	5,9%	4,5%	10,4%
	Yes	Contagem	784	393	1177
		% em The institution is a university hospital	66,6%	33,4%	100,0%
		% do Total	59,7%	29,9%	89,6%
Total		Contagem	861	452	1313
		% em The institution is a university hospital	65,6%	34,4%	100,0%
		% do Total	65,6%	34,4%	100,0%

Testes qui-quadrado

	Valor	gl	Significância Assintótica (Bilateral)	Sig exata (2 lados)	Sig exata (1 lado)

Qui-quadrado de Pearson	5,392[a]	1	,020		
Correção de continuidade[b]	4,959	1	,026		
Razão de verossimilhança	5,233	1	,022		
Teste Exato de Fisher				,022	,014
Associação Linear por Linear	5,388	1	,020		
N de Casos Válidos	1313				

a. 0 células (,0%) esperavam uma contagem menor que 5. A contagem mínima esperada é 46,82.

b. Computado apenas para uma tabela 2x2

Medidas Simétricas

		Valor	Significância Aproximada
Nominal por Nominal	Fi	-,064	,020
	V de Cramer	,064	,020
N de Casos Válidos		1313	

Estimativa de Risco

	Valor	Intervalo de confiança de 95% Inferior	Superior
Razão de Chances para The institution is a university hospital (No / Yes)	,654	,456	,938
Para grupo DASS21 Classification - Anxiety (dichotomous) = Normal	,850	,730	,990
Para grupo DASS21 Classification - Anxiety (dichotomous) = Abnormal (elevated)	1,299	1,055	1,600
N de Casos Válidos	1313		

```
The institution is a university hospital * DASS21 Classification - Stress (dichotomous)
```

Crosstab

			DASS21 Classification - Stress (dichotomous) Normal	Abnormal (elevated)	Total
The institution is a university hospital	No	Contagem	100	36	136
		% em The institution is a university hospital	73,5%	26,5%	100,0%
		% do Total	7,6%	2,7%	10,4%

	Yes	Contagem	912	265	1177
		% em The institution is a university hospital	77,5%	22,5%	100,0%
		% do Total	69,5%	20,2%	89,6%
Total		Contagem	1012	301	1313
		% em The institution is a university hospital	77,1%	22,9%	100,0%
		% do Total	77,1%	22,9%	100,0%

Testes qui-quadrado

	Valor	gl	Significância Assintótica (Bilateral)	Sig exata (2 lados)	Sig exata (1 lado)
Qui-quadrado de Pearson	1,080[a]	1	,299		
Correção de continuidade[b]	,867	1	,352		
Razão de verossimilhança	1,048	1	,306		
Teste Exato de Fisher				,332	,175
Associação Linear por Linear	1,079	1	,299		
N de Casos Válidos	1313				

a. 0 células (,0%) esperavam uma contagem menor que 5. A contagem mínima esperada é 31,18.

b. Computado apenas para uma tabela 2x2

Medidas Simétricas

		Valor	Significância Aproximada
Nominal por Nominal	Fi	-,029	,299
	V de Cramer	,029	,299
N de Casos Válidos		1313	

Estimativa de Risco

	Valor	Intervalo de confiança de 95% Inferior	Superior
Razão de Chances para The institution is a university hospital (No / Yes)	,807	,538	1,210
Para grupo DASS21 Classification - Stress (dichotomous) = Normal	,949	,854	1,054
Para grupo DASS21 Classification - Stress (dichotomous) = Abnormal (elevated)	1,176	,871	1,586
N de Casos Válidos	1313		

```
The institution is a university hospital * PHQ9 Depression Classification - Risk estimate for having
current major depressive disorder
```

Crosstab

			PHQ9 Depression Classification - Risk estimate for having current major depressive disorder		
			Low	High	Total
The institution is a university hospital	No	Contagem	30	106	136
		% em The institution is a university hospital	22,1%	77,9%	100,0%
		% do Total	2,3%	8,1%	10,4%
	Yes	Contagem	399	778	1177
		% em The institution is a university hospital	33,9%	66,1%	100,0%
		% do Total	30,4%	59,3%	89,6%
Total		Contagem	429	884	1313
		% em The institution is a university hospital	32,7%	67,3%	100,0%
		% do Total	32,7%	67,3%	100,0%

Testes qui-quadrado

	Valor	gl	Significância Assintótica (Bilateral)	Sig exata (2 lados)	Sig exata (1 lado)
Qui-quadrado de Pearson	7,770[a]	1	,005		
Correção de continuidade[b]	7,241	1	,007		
Razão de verossimilhança	8,265	1	,004		
Teste Exato de Fisher				,005	,003
Associação Linear por Linear	7,764	1	,005		
N de Casos Válidos	1313				

a. 0 células (,0%) esperavam uma contagem menor que 5. A contagem mínima esperada é 44,44.

b. Computado apenas para uma tabela 2x2

Medidas Simétricas

		Valor	Significância Aproximada
Nominal por Nominal	Fi	-,077	,005
	V de Cramer	,077	,005
N de Casos Válidos		1313	

Estimativa de Risco

	Valor	Intervalo de confiança de 95% Inferior	Superior
Razão de Chances para The institution is a university hospital (No / Yes)	,552	,362	,842
Para grupo PHQ9 Depression Classification - Risk estimate for having current major depressive disorder = Low	,651	,470	,901
Para grupo PHQ9 Depression Classification - Risk estimate for having current major depressive disorder = High	1,179	1,069	1,301
N de Casos Válidos	1313		

```
The institution is a university hospital * BRCS Classification
```

Crosstab

			BRCS Classification Low resilience	Moderate to High	Total
The institution is a university hospital	No	Contagem	83	53	136
		% em The institution is a university hospital	61,0%	39,0%	100,0%
		% do Total	6,3%	4,0%	10,4%
	Yes	Contagem	730	447	1177
		% em The institution is a university hospital	62,0%	38,0%	100,0%
		% do Total	55,6%	34,0%	89,6%
Total		Contagem	813	500	1313
		% em The institution is a university hospital	61,9%	38,1%	100,0%
		% do Total	61,9%	38,1%	100,0%

Testes qui-quadrado

	Valor	gl	Significância Assintótica (Bilateral)	Sig exata (2 lados)	Sig exata (1 lado)
Qui-quadrado de Pearson	,051[a]	1	,821		
Correção de continuidade[b]	,018	1	,895		
Razão de verossimilhança	,051	1	,822		

Teste Exato de Fisher				,852	,445
Associação Linear por Linear	,051	1	,821		
N de Casos Válidos	1313				

a. 0 células (,0%) esperavam uma contagem menor que 5. A contagem mínima esperada é 51,79.

b. Computado apenas para uma tabela 2x2

Medidas Simétricas

		Valor	Significância Aproximada
Nominal por Nominal	Fi	-,006	,821
	V de Cramer	,006	,821
N de Casos Válidos		1313	

Estimativa de Risco

	Valor	Intervalo de confiança de 95% Inferior	Intervalo de confiança de 95% Superior
Razão de Chances para The institution is a university hospital (No / Yes)	,959	,666	1,380
Para grupo BRCS Classification = Low resilience	,984	,854	1,134
Para grupo BRCS Classification = Moderate to High	1,026	,821	1,282
N de Casos Válidos	1313		

```
The institution is a university hospital * OLBI Score Classification
```

Crosstab

			OLBI Score Classification		
			Low to Moderate	High	Total
The institution is a university hospital	No	Contagem	83	53	136
		% em The institution is a university hospital	61,0%	39,0%	100,0%
		% do Total	6,3%	4,0%	10,4%
	Yes	Contagem	792	385	1177
		% em The institution is a university hospital	67,3%	32,7%	100,0%
		% do Total	60,3%	29,3%	89,6%
Total		Contagem	875	438	1313

	% em The institution is a university hospital	66,6%	33,4%	100,0%
	% do Total	66,6%	33,4%	100,0%

Testes qui-quadrado

	Valor	gl	Significância Assintótica (Bilateral)	Sig exata (2 lados)	Sig exata (1 lado)
Qui-quadrado de Pearson	2,149[a]	1	,143		
Correção de continuidade[b]	1,877	1	,171		
Razão de verossimilhança	2,102	1	,147		
Teste Exato de Fisher				,150	,086
Associação Linear por Linear	2,148	1	,143		
N de Casos Válidos	1313				

a. 0 células (,0%) esperavam uma contagem menor que 5. A contagem mínima esperada é 45,37.

b. Computado apenas para uma tabela 2x2

Medidas Simétricas

		Valor	Significância Aproximada
Nominal por Nominal	Fi	-,040	,143
	V de Cramer	,040	,143
N de Casos Válidos		1313	

Estimativa de Risco

	Valor	Intervalo de confiança de 95% Inferior	Superior
Razão de Chances para The institution is a university hospital (No / Yes)	,761	,528	1,097
Para grupo OLBI Score Classification = Low to Moderate	,907	,788	1,043
Para grupo OLBI Score Classification = High	1,191	,951	1,493
N de Casos Válidos	1313		

Classification of the percieved autonomy to self-conduct in the residency program * DASS21 Classification - Depression (dichotomous)

Crosstab

			DASS21 Classification - Depression (dichotomous)		
			Normal	Abnormal (elevated)	Total
Classification of the percieved autonomy to self-conduct in the residency program	Low autonomy	Contagem	104	120	224
		% em Classification of the percieved autonomy to self-conduct in the residency program	46,4%	53,6%	100,0%
		% do Total	7,9%	9,1%	17,1%
	Moderate to high autonomy	Contagem	766	323	1089
		% em Classification of the percieved autonomy to self-conduct in the residency program	70,3%	29,7%	100,0%
		% do Total	58,3%	24,6%	82,9%
Total		Contagem	870	443	1313
		% em Classification of the percieved autonomy to self-conduct in the residency program	66,3%	33,7%	100,0%
		% do Total	66,3%	33,7%	100,0%

Testes qui-quadrado

	Valor	gl	Significância Assintótica (Bilateral)	Sig exata (2 lados)	Sig exata (1 lado)
Qui-quadrado de Pearson	47,514[a]	1	,000		
Correção de continuidade[b]	46,450	1	,000		
Razão de verossimilhança	45,264	1	,000		
Teste Exato de Fisher				,000	,000
Associação Linear por Linear	47,478	1	,000		
N de Casos Válidos	1313				

a. 0 células (,0%) esperavam uma contagem menor que 5. A contagem mínima esperada é 75,58.

b. Computado apenas para uma tabela 2x2

Medidas Simétricas

		Valor	Significância Aproximada
Nominal por Nominal	Fi	-,190	,000
	V de Cramer	,190	,000
N de Casos Válidos		1313	

Estimativa de Risco

	Valor	Intervalo de confiança de 95%

		Inferior	Superior
Razão de Chances para Classification of the percieved autonomy to self-conduct in the residency program (Low autonomy / Moderate to high autonomy)	,365	,273	,490
Para grupo DASS21 Classification - Depression (dichotomous) = Normal	,660	,570	,764
Para grupo DASS21 Classification - Depression (dichotomous) = Abnormal (elevated)	1,806	1,551	2,104
N de Casos Válidos	1313		

```
Classification of the percieved autonomy to self-conduct in the residency program * DASS21 Classification
- Anxiety (dichotomous)
```

Crosstab

			DASS21 Classification - Anxiety (dichotomous)		
			Normal	Abnormal (elevated)	Total
Classification of the percieved autonomy to self-conduct in the residency program	Low autonomy	Contagem	119	105	224
		% em Classification of the percieved autonomy to self-conduct in the residency program	53,1%	46,9%	100,0%
		% do Total	9,1%	8,0%	17,1%
	Moderate to high autonomy	Contagem	742	347	1089
		% em Classification of the percieved autonomy to self-conduct in the residency program	68,1%	31,9%	100,0%
		% do Total	56,5%	26,4%	82,9%
Total		Contagem	861	452	1313
		% em Classification of the percieved autonomy to self-conduct in the residency program	65,6%	34,4%	100,0%
		% do Total	65,6%	34,4%	100,0%

Testes qui-quadrado

	Valor	gl	Significância Assintótica (Bilateral)	Sig exata (2 lados)	Sig exata (1 lado)

Qui-quadrado de Pearson	18,544[a]	1	,000		
Correção de continuidade[b]	17,885	1	,000		
Razão de verossimilhança	17,920	1	,000		
Teste Exato de Fisher				,000	,000
Associação Linear por Linear	18,530	1	,000		
N de Casos Válidos	1313				

a. 0 células (,0%) esperavam uma contagem menor que 5. A contagem mínima esperada é 77,11.

b. Computado apenas para uma tabela 2x2

Medidas Simétricas

		Valor	Significância Aproximada
Nominal por Nominal	Fi	-,119	,000
	V de Cramer	,119	,000
N de Casos Válidos		1313	

Estimativa de Risco

	Valor	Intervalo de confiança de 95% Inferior	Superior
Razão de Chances para Classification of the percieved autonomy to self-conduct in the residency program (Low autonomy / Moderate to high autonomy)	,530	,396	,710
Para grupo DASS21 Classification - Anxiety (dichotomous) = Normal	,780	,685	,888
Para grupo DASS21 Classification - Anxiety (dichotomous) = Abnormal (elevated)	1,471	1,248	1,734
N de Casos Válidos	1313		

Classification of the percieved autonomy to self-conduct in the residency program * DASS21 Classification - Stress (dichotomous)

Crosstab

			DASS21 Classification - Stress (dichotomous) Normal	Abnormal (elevated)	Total
Classification of the percieved	Low autonomy	Contagem	147	77	224

autonomy to self-conduct in the residency program		% em Classification of the percieved autonomy to self-conduct in the residency program	65,6%	34,4%	100,0%
		% do Total	11,2%	5,9%	17,1%
	Moderate to high autonomy	Contagem	865	224	1089
		% em Classification of the percieved autonomy to self-conduct in the residency program	79,4%	20,6%	100,0%
		% do Total	65,9%	17,1%	82,9%
Total		Contagem	1012	301	1313
		% em Classification of the percieved autonomy to self-conduct in the residency program	77,1%	22,9%	100,0%
		% do Total	77,1%	22,9%	100,0%

Testes qui-quadrado

	Valor	gl	Significância Assintótica (Bilateral)	Sig exata (2 lados)	Sig exata (1 lado)
Qui-quadrado de Pearson	20,041[a]	1	,000		
Correção de continuidade[b]	19,267	1	,000		
Razão de verossimilhança	18,611	1	,000		
Teste Exato de Fisher				,000	,000
Associação Linear por Linear	20,025	1	,000		
N de Casos Válidos	1313				

a. 0 células (,0%) esperavam uma contagem menor que 5. A contagem mínima esperada é 51,35.

b. Computado apenas para uma tabela 2x2

Medidas Simétricas

		Valor	Significância Aproximada
Nominal por Nominal	Fi	-,124	,000
	V de Cramer	,124	,000
N de Casos Válidos		1313	

Estimativa de Risco

	Valor	Intervalo de confiança de 95% Inferior	Superior
Razão de Chances para Classification of the percieved autonomy to self-conduct in the residency program (Low autonomy / Moderate to high autonomy)	,494	,362	,676

Para grupo DASS21 Classification - Stress (dichotomous) = Normal	,826	,748	,913
Para grupo DASS21 Classification - Stress (dichotomous) = Abnormal (elevated)	1,671	1,347	2,073
N de Casos Válidos	1313		

```
Classification of the percieved autonomy to self-conduct in the residency program * PHQ9 Depression
Classification - Risk estimate for having current major depressive disorder
```

Crosstab

			PHQ9 Depression Classification - Risk estimate for having current major depressive disorder		
			Low	High	Total
Classification of the percieved autonomy to self-conduct in the residency program	Low autonomy	Contagem	46	178	224
		% em Classification of the percieved autonomy to self-conduct in the residency program	20,5%	79,5%	100,0%
		% do Total	3,5%	13,6%	17,1%
	Moderate to high autonomy	Contagem	383	706	1089
		% em Classification of the percieved autonomy to self-conduct in the residency program	35,2%	64,8%	100,0%
		% do Total	29,2%	53,8%	82,9%
Total		Contagem	429	884	1313
		% em Classification of the percieved autonomy to self-conduct in the residency program	32,7%	67,3%	100,0%
		% do Total	32,7%	67,3%	100,0%

Testes qui-quadrado

	Valor	gl	Significância Assintótica (Bilateral)	Sig exata (2 lados)	Sig exata (1 lado)
Qui-quadrado de Pearson	18,087[a]	1	,000		
Correção de continuidade[b]	17,428	1	,000		
Razão de verossimilhança	19,330	1	,000		
Teste Exato de Fisher				,000	,000
Associação Linear por Linear	18,073	1	,000		
N de Casos Válidos	1313				

a. 0 células (,0%) esperavam uma contagem menor que 5. A contagem mínima esperada é 73,19.

b. Computado apenas para uma tabela 2x2

Medidas Simétricas

		Valor	Significância Aproximada
Nominal por Nominal	Fi	-,117	,000
	V de Cramer	,117	,000
N de Casos Válidos		1313	

Estimativa de Risco

	Valor	Intervalo de confiança de 95% Inferior	Superior
Razão de Chances para Classification of the percieved autonomy to self-conduct in the residency program (Low autonomy / Moderate to high autonomy)	,476	,337	,674
Para grupo PHQ9 Depression Classification - Risk estimate for having current major depressive disorder = Low	,584	,446	,765
Para grupo PHQ9 Depression Classification - Risk estimate for having current major depressive disorder = High	1,226	1,132	1,327
N de Casos Válidos	1313		

Classification of the percieved autonomy to self-conduct in the residency program * BRCS Classification

Crosstab

			BRCS Classification Low resilience	Moderate to High	Total
Classification of the percieved autonomy to self-conduct in the residency program	Low autonomy	Contagem	168	56	224
		% em Classification of the percieved autonomy to self-conduct in the residency program	75,0%	25,0%	100,0%
		% do Total	12,8%	4,3%	17,1%
	Moderate to high autonomy	Contagem	645	444	1089
		% em Classification of the percieved autonomy to self-conduct in the residency program	59,2%	40,8%	100,0%

	% do Total	49,1%	33,8%	82,9%
Total	Contagem	813	500	1313
	% em Classification of the percieved autonomy to self-conduct in the residency program	61,9%	38,1%	100,0%
	% do Total	61,9%	38,1%	100,0%

Testes qui-quadrado

	Valor	gl	Significância Assintótica (Bilateral)	Sig exata (2 lados)	Sig exata (1 lado)
Qui-quadrado de Pearson	19,598[a]	1	,000		
Correção de continuidade[b]	18,935	1	,000		
Razão de verossimilhança	20,579	1	,000		
Teste Exato de Fisher				,000	,000
Associação Linear por Linear	19,583	1	,000		
N de Casos Válidos	1313				

a. 0 células (,0%) esperavam uma contagem menor que 5. A contagem mínima esperada é 85,30.

b. Computado apenas para uma tabela 2x2

Medidas Simétricas

		Valor	Significância Aproximada
Nominal por Nominal	Fi	,122	,000
	V de Cramer	,122	,000
N de Casos Válidos		1313	

Estimativa de Risco

	Valor	Intervalo de confiança de 95% Inferior	Superior
Razão de Chances para Classification of the percieved autonomy to self-conduct in the residency program (Low autonomy / Moderate to high autonomy)	2,065	1,491	2,860
Para grupo BRCS Classification = Low resilience	1,266	1,157	1,386
Para grupo BRCS Classification = Moderate to High	,613	,483	,778
N de Casos Válidos	1313		

Classification of the percieved autonomy to self-conduct in the residency program * OLBI Score Classification

Crosstab

			OLBI Score Classification		
			Low to Moderate	High	Total
Classification of the percieved autonomy to self-conduct in the residency program	Low autonomy	Contagem	92	132	224
		% em Classification of the percieved autonomy to self-conduct in the residency program	41,1%	58,9%	100,0%
		% do Total	7,0%	10,1%	17,1%
	Moderate to high autonomy	Contagem	783	306	1089
		% em Classification of the percieved autonomy to self-conduct in the residency program	71,9%	28,1%	100,0%
		% do Total	59,6%	23,3%	82,9%
Total		Contagem	875	438	1313
		% em Classification of the percieved autonomy to self-conduct in the residency program	66,6%	33,4%	100,0%
		% do Total	66,6%	33,4%	100,0%

Testes qui-quadrado

	Valor	gl	Significância Assintótica (Bilateral)	Sig exata (2 lados)	Sig exata (1 lado)
Qui-quadrado de Pearson	79,431[a]	1	,000		
Correção de continuidade[b]	78,050	1	,000		
Razão de verossimilhança	75,112	1	,000		
Teste Exato de Fisher				,000	,000
Associação Linear por Linear	79,370	1	,000		
N de Casos Válidos	1313				

a. 0 células (,0%) esperavam uma contagem menor que 5. A contagem mínima esperada é 74,72.

b. Computado apenas para uma tabela 2x2

Medidas Simétricas

		Valor	Significância Aproximada
Nominal por Nominal	Fi	-,246	,000
	V de Cramer	,246	,000

N de Casos Válidos	1313	

Estimativa de Risco

	Valor	Intervalo de confiança de 95% Inferior	Superior
Razão de Chances para Classification of the percieved autonomy to self-conduct in the residency program (Low autonomy / Moderate to high autonomy)	,272	,202	,367
Para grupo OLBI Score Classification = Low to Moderate	,571	,486	,671
Para grupo OLBI Score Classification = High	2,097	1,814	2,424
N de Casos Válidos	1313		

```
Percieved adequacy of the pedagogic structure and availability of resouces in the residency program *
DASS21 Classification - Depression (dichotomous)
```

Crosstab

			DASS21 Classification - Depression (dichotomous)		
			Normal	Abnormal (elevated)	Total
Percieved adequacy of the pedagogic structure and availability of resouces in the residency program	Poor adequacy	Contagem	309	249	558
		% em Percieved adequacy of the pedagogic structure and availability of resouces in the residency program	55,4%	44,6%	100,0%
		% do Total	23,5%	19,0%	42,5%
	Moderate to good adequacy	Contagem	561	194	755
		% em Percieved adequacy of the pedagogic structure and availability of resouces in the residency program	74,3%	25,7%	100,0%
		% do Total	42,7%	14,8%	57,5%
Total		Contagem	870	443	1313
		% em Percieved adequacy of the pedagogic structure and availability of resouces in the residency program	66,3%	33,7%	100,0%
		% do Total	66,3%	33,7%	100,0%

Testes qui-quadrado

	Valor	gl	Significância Assintótica (Bilateral)	Sig exata (2 lados)	Sig exata (1 lado)
Qui-quadrado de Pearson	51,422[a]	1	,000		
Correção de continuidade[b]	50,578	1	,000		
Razão de verossimilhança	51,226	1	,000		
Teste Exato de Fisher				,000	,000
Associação Linear por Linear	51,382	1	,000		
N de Casos Válidos	1313				

a. 0 células (,0%) esperavam uma contagem menor que 5. A contagem mínima esperada é 188,27.

b. Computado apenas para uma tabela 2x2

Medidas Simétricas

		Valor	Significância Aproximada
Nominal por Nominal	Fi	-,198	,000
	V de Cramer	,198	,000
N de Casos Válidos		1313	

Estimativa de Risco

	Valor	Intervalo de confiança de 95%	
		Inferior	Superior
Razão de Chances para Percieved adequacy of the pedagogic structure and availability of resouces in the residency program (Poor adequacy / Moderate to good adequacy)	,429	,340	,542
Para grupo DASS21 Classification - Depression (dichotomous) = Normal	,745	,684	,812
Para grupo DASS21 Classification - Depression (dichotomous) = Abnormal (elevated)	1,737	1,491	2,023
N de Casos Válidos	1313		

Percieved adequacy of the pedagogic structure and availability of resouces in the residency program * DASS21 Classification - Anxiety (dichotomous)

Crosstab

			DASS21 Classification - Anxiety (dichotomous)		
			Normal	Abnormal (elevated)	Total
Percieved adequacy of the pedagogic structure and availability of resouces in the residency program	Poor adequacy	Contagem	313	245	558
		% em Percieved adequacy of the pedagogic structure and availability of resouces in the residency program	56,1%	43,9%	100,0%
		% do Total	23,8%	18,7%	42,5%
	Moderate to good adequacy	Contagem	548	207	755
		% em Percieved adequacy of the pedagogic structure and availability of resouces in the residency program	72,6%	27,4%	100,0%
		% do Total	41,7%	15,8%	57,5%
Total		Contagem	861	452	1313
		% em Percieved adequacy of the pedagogic structure and availability of resouces in the residency program	65,6%	34,4%	100,0%
		% do Total	65,6%	34,4%	100,0%

Testes qui-quadrado

	Valor	gl	Significância Assintótica (Bilateral)	Sig exata (2 lados)	Sig exata (1 lado)
Qui-quadrado de Pearson	38,648[a]	1	,000		
Correção de continuidade[b]	37,921	1	,000		
Razão de verossimilhança	38,491	1	,000		
Teste Exato de Fisher				,000	,000
Associação Linear por Linear	38,618	1	,000		
N de Casos Válidos	1313				

a. 0 células (,0%) esperavam uma contagem menor que 5. A contagem mínima esperada é 192,09.

b. Computado apenas para uma tabela 2x2

Medidas Simétricas

		Valor	Significância Aproximada
Nominal por Nominal	Fi	-,172	,000
	V de Cramer	,172	,000

N de Casos Válidos	1313	

Estimativa de Risco

	Valor	Intervalo de confiança de 95% Inferior	Superior
Razão de Chances para Percieved adequacy of the pedagogic structure and availability of resouces in the residency program (Poor adequacy / Moderate to good adequacy)	,483	,383	,608
Para grupo DASS21 Classification - Anxiety (dichotomous) = Normal	,773	,709	,842
Para grupo DASS21 Classification - Anxiety (dichotomous) = Abnormal (elevated)	1,601	1,379	1,859
N de Casos Válidos	1313		

Percieved adequacy of the pedagogic structure and availability of resouces in the residency program * DASS21 Classification - Stress (dichotomous)

Crosstab

			DASS21 Classification - Stress (dichotomous) Normal	Abnormal (elevated)	Total
Percieved adequacy of the pedagogic structure and availability of resouces in the residency program	Poor adequacy	Contagem	370	188	558
		% em Percieved adequacy of the pedagogic structure and availability of resouces in the residency program	66,3%	33,7%	100,0%
		% do Total	28,2%	14,3%	42,5%
	Moderate to good adequacy	Contagem	642	113	755
		% em Percieved adequacy of the pedagogic structure and availability of resouces in the residency program	85,0%	15,0%	100,0%
		% do Total	48,9%	8,6%	57,5%
Total		Contagem	1012	301	1313

	% em Percieved adequacy of the pedagogic structure and availability of resouces in the residency program	77,1%	22,9%	100,0%
	% do Total	77,1%	22,9%	100,0%

Testes qui-quadrado

	Valor	gl	Significância Assintótica (Bilateral)	Sig exata (2 lados)	Sig exata (1 lado)
Qui-quadrado de Pearson	63,670[a]	1	,000		
Correção de continuidade[b]	62,615	1	,000		
Razão de verossimilhança	63,230	1	,000		
Teste Exato de Fisher				,000	,000
Associação Linear por Linear	63,622	1	,000		
N de Casos Válidos	1313				

a. 0 células (,0%) esperavam uma contagem menor que 5. A contagem mínima esperada é 127,92.

b. Computado apenas para uma tabela 2x2

Medidas Simétricas

		Valor	Significância Aproximada
Nominal por Nominal	Fi	-,220	,000
	V de Cramer	,220	,000
N de Casos Válidos		1313	

Estimativa de Risco

	Valor	Intervalo de confiança de 95%	
		Inferior	Superior
Razão de Chances para Percieved adequacy of the pedagogic structure and availability of resouces in the residency program (Poor adequacy / Moderate to good adequacy)	,346	,265	,452
Para grupo DASS21 Classification - Stress (dichotomous) = Normal	,780	,730	,833
Para grupo DASS21 Classification - Stress (dichotomous) = Abnormal (elevated)	2,251	1,832	2,766
N de Casos Válidos	1313		

Percieved adequacy of the pedagogic structure and availability of resouces in the residency program * PHQ9 Depression Classification - Risk estimate for having current major depressive disorder

Crosstab

			PHQ9 Depression Classification - Risk estimate for having current major depressive disorder		
			Low	High	Total
Percieved adequacy of the pedagogic structure and availability of resouces in the residency program	Poor adequacy	Contagem	117	441	558
		% em Percieved adequacy of the pedagogic structure and availability of resouces in the residency program	21,0%	79,0%	100,0%
		% do Total	8,9%	33,6%	42,5%
	Moderate to good adequacy	Contagem	312	443	755
		% em Percieved adequacy of the pedagogic structure and availability of resouces in the residency program	41,3%	58,7%	100,0%
		% do Total	23,8%	33,7%	57,5%
Total		Contagem	429	884	1313
		% em Percieved adequacy of the pedagogic structure and availability of resouces in the residency program	32,7%	67,3%	100,0%
		% do Total	32,7%	67,3%	100,0%

Testes qui-quadrado

	Valor	gl	Significância Assintótica (Bilateral)	Sig exata (2 lados)	Sig exata (1 lado)
Qui-quadrado de Pearson	60,444[a]	1	,000		
Correção de continuidade[b]	59,522	1	,000		
Razão de verossimilhança	62,308	1	,000		
Teste Exato de Fisher				,000	,000
Associação Linear por Linear	60,398	1	,000		
N de Casos Válidos	1313				

a. 0 células (,0%) esperavam uma contagem menor que 5. A contagem mínima esperada é 182,32.

b. Computado apenas para uma tabela 2x2

Medidas Simétricas

		Valor	Significância Aproximada
Nominal por Nominal	Fi	-,215	,000
	V de Cramer	,215	,000
N de Casos Válidos		1313	

Estimativa de Risco

	Valor	Intervalo de confiança de 95%	
		Inferior	Superior
Razão de Chances para Percieved adequacy of the pedagogic structure and availability of resouces in the residency program (Poor adequacy / Moderate to good adequacy)	,377	,293	,484
Para grupo PHQ9 Depression Classification - Risk estimate for having current major depressive disorder = Low	,507	,423	,609
Para grupo PHQ9 Depression Classification - Risk estimate for having current major depressive disorder = High	1,347	1,251	1,450
N de Casos Válidos	1313		

Percieved adequacy of the pedagogic structure and availability of resouces in the residency program * BRCS Classification

Crosstab

			BRCS Classification		Total
			Low resilience	Moderate to High	
Percieved adequacy of the pedagogic structure and availability of resouces in the residency program	Poor adequacy	Contagem	398	160	558
		% em Percieved adequacy of the pedagogic structure and availability of resouces in the residency program	71,3%	28,7%	100,0%
		% do Total	30,3%	12,2%	42,5%
	Moderate to good adequacy	Contagem	415	340	755
		% em Percieved adequacy of the pedagogic structure and availability of resouces in the residency program	55,0%	45,0%	100,0%

	% do Total	31,6%	25,9%	57,5%
Total	Contagem	813	500	1313
	% em Percieved adequacy of the pedagogic structure and availability of resouces in the residency program	61,9%	38,1%	100,0%
	% do Total	61,9%	38,1%	100,0%

Testes qui-quadrado

	Valor	gl	Significância Assintótica (Bilateral)	Sig exata (2 lados)	Sig exata (1 lado)
Qui-quadrado de Pearson	36,418[a]	1	,000		
Correção de continuidade[b]	35,727	1	,000		
Razão de verossimilhança	36,964	1	,000		
Teste Exato de Fisher				,000	,000
Associação Linear por Linear	36,390	1	,000		
N de Casos Válidos	1313				

a. 0 células (,0%) esperavam uma contagem menor que 5. A contagem mínima esperada é 212,49.

b. Computado apenas para uma tabela 2x2

Medidas Simétricas

		Valor	Significância Aproximada
Nominal por Nominal	Fi	,167	,000
	V de Cramer	,167	,000
N de Casos Válidos		1313	

Estimativa de Risco

	Valor	Intervalo de confiança de 95% Inferior	Superior
Razão de Chances para Percieved adequacy of the pedagogic structure and availability of resouces in the residency program (Poor adequacy / Moderate to good adequacy)	2,038	1,615	2,572
Para grupo BRCS Classification = Low resilience	1,298	1,194	1,410
Para grupo BRCS Classification = Moderate to High	,637	,547	,742
N de Casos Válidos	1313		

Percieved adequacy of the pedagogic structure and availability of resouces in the residency program * OLBI Score Classification

Crosstab

			OLBI Score Classification		
			Low to Moderate	High	Total
Percieved adequacy of the pedagogic structure and availability of resouces in the residency program	Poor adequacy	Contagem	278	280	558
		% em Percieved adequacy of the pedagogic structure and availability of resouces in the residency program	49,8%	50,2%	100,0%
		% do Total	21,2%	21,3%	42,5%
	Moderate to good adequacy	Contagem	597	158	755
		% em Percieved adequacy of the pedagogic structure and availability of resouces in the residency program	79,1%	20,9%	100,0%
		% do Total	45,5%	12,0%	57,5%
Total		Contagem	875	438	1313
		% em Percieved adequacy of the pedagogic structure and availability of resouces in the residency program	66,6%	33,4%	100,0%
		% do Total	66,6%	33,4%	100,0%

Testes qui-quadrado

	Valor	gl	Significância Assintótica (Bilateral)	Sig exata (2 lados)	Sig exata (1 lado)
Qui-quadrado de Pearson	123,503[a]	1	,000		
Correção de continuidade[b]	122,190	1	,000		
Razão de verossimilhança	123,788	1	,000		
Teste Exato de Fisher				,000	,000
Associação Linear por Linear	123,409	1	,000		
N de Casos Válidos	1313				

a. 0 células (,0%) esperavam uma contagem menor que 5. A contagem mínima esperada é 186,14.

b. Computado apenas para uma tabela 2x2

Medidas Simétricas

		Valor	Significância Aproximada
Nominal por Nominal	Fi	-,307	,000
	V de Cramer	,307	,000
N de Casos Válidos		1313	

Estimativa de Risco

	Valor	Intervalo de confiança de 95% Inferior	Superior
Razão de Chances para Percieved adequacy of the pedagogic structure and availability of resouces in the residency program (Poor adequacy / Moderate to good adequacy)	,263	,206	,335
Para grupo OLBI Score Classification = Low to Moderate	,630	,575	,690
Para grupo OLBI Score Classification = High	2,398	2,040	2,818
N de Casos Válidos	1313		

```
Perceived adequacy of the availability of personal protective equipment, when providing care for patients
in the residency program * DASS21 Classification - Depression (dichotomous)
```

Crosstab

			DASS21 Classification - Depression (dichotomous) Normal	Abnormal (elevated)	Total
Perceived adequacy of the availability of personal protective equipment, when providing care for patients in the residency program	Poor adequacy	Contagem	135	146	281
		% em Perceived adequacy of the availability of personal protective equipment, when providing care for patients in the residency program	48,0%	52,0%	100,0%
		% do Total	10,3%	11,1%	21,4%
	Good adequacy	Contagem	735	297	1032
		% em Perceived adequacy of the availability of personal protective equipment, when providing care for patients in the residency program	71,2%	28,8%	100,0%

	% do Total	56,0%	22,6%	78,6%
Total	Contagem	870	443	1313
	% em Perceived adequacy of the availability of personal protective equipment, when providing care for patients in the residency program	66,3%	33,7%	100,0%
	% do Total	66,3%	33,7%	100,0%

Testes qui-quadrado

	Valor	gl	Significância Assintótica (Bilateral)	Sig exata (2 lados)	Sig exata (1 lado)
Qui-quadrado de Pearson	53,075[a]	1	,000		
Correção de continuidade[b]	52,043	1	,000		
Razão de verossimilhança	50,931	1	,000		
Teste Exato de Fisher				,000	,000
Associação Linear por Linear	53,034	1	,000		
N de Casos Válidos	1313				

a. 0 células (,0%) esperavam uma contagem menor que 5. A contagem mínima esperada é 94,81.

b. Computado apenas para uma tabela 2x2

Medidas Simétricas

		Valor	Significância Aproximada
Nominal por Nominal	Fi	-,201	,000
	V de Cramer	,201	,000
N de Casos Válidos		1313	

Estimativa de Risco

	Valor	Intervalo de confiança de 95% Inferior	Superior
Razão de Chances para Perceived adequacy of the availability of personal protective equipment, when providing care for patients in the residency program (Poor adequacy / Good adequacy)	,374	,285	,489
Para grupo DASS21 Classification - Depression (dichotomous) = Normal	,675	,594	,766

Para grupo DASS21 Classification - Depression (dichotomous) = Abnormal (elevated)	1,805	1,557	2,093
N de Casos Válidos	1313		

```
Perceived adequacy of the availability of personal protective equipment, when providing care for patients
in the residency program * DASS21 Classification - Anxiety (dichotomous)
```

Crosstab

			DASS21 Classification - Anxiety (dichotomous)		
			Normal	Abnormal (elevated)	Total
Perceived adequacy of the availability of personal protective equipment, when providing care for patients in the residency program	Poor adequacy	Contagem	151	130	281
		% em Perceived adequacy of the availability of personal protective equipment, when providing care for patients in the residency program	53,7%	46,3%	100,0%
		% do Total	11,5%	9,9%	21,4%
	Good adequacy	Contagem	710	322	1032
		% em Perceived adequacy of the availability of personal protective equipment, when providing care for patients in the residency program	68,8%	31,2%	100,0%
		% do Total	54,1%	24,5%	78,6%
Total		Contagem	861	452	1313
		% em Perceived adequacy of the availability of personal protective equipment, when providing care for patients in the residency program	65,6%	34,4%	100,0%
		% do Total	65,6%	34,4%	100,0%

Testes qui-quadrado

	Valor	gl	Significância Assintótica (Bilateral)	Sig exata (2 lados)	Sig exata (1 lado)
Qui-quadrado de Pearson	22,195[a]	1	,000		
Correção de continuidade[b]	21,533	1	,000		
Razão de verossimilhança	21,546	1	,000		

Teste Exato de Fisher				,000	,000
Associação Linear por Linear	22,178	1	,000		
N de Casos Válidos	1313				

a. 0 células (,0%) esperavam uma contagem menor que 5. A contagem mínima esperada é 96,73.

b. Computado apenas para uma tabela 2x2

Medidas Simétricas

		Valor	Significância Aproximada
Nominal por Nominal	Fi	-,130	,000
	V de Cramer	,130	,000
N de Casos Válidos		1313	

Estimativa de Risco

	Valor	Intervalo de confiança de 95% Inferior	Superior
Razão de Chances para Perceived adequacy of the availability of personal protective equipment, when providing care for patients in the residency program (Poor adequacy / Good adequacy)	,527	,403	,689
Para grupo DASS21 Classification - Anxiety (dichotomous) = Normal	,781	,696	,877
Para grupo DASS21 Classification - Anxiety (dichotomous) = Abnormal (elevated)	1,483	1,270	1,732
N de Casos Válidos	1313		

```
Perceived adequacy of the availability of personal protective equipment, when providing care for patients
in the residency program * DASS21 Classification - Stress (dichotomous)
```

Crosstab

			DASS21 Classification - Stress (dichotomous) Normal	Abnormal (elevated)	Total
Perceived adequacy of the	Poor adequacy	Contagem	184	97	281

availability of personal protective equipment, when providing care for patients in the residency program		% em Perceived adequacy of the availability of personal protective equipment, when providing care for patients in the residency program	65,5%	34,5%	100,0%
		% do Total	14,0%	7,4%	21,4%
	Good adequacy	Contagem	828	204	1032
		% em Perceived adequacy of the availability of personal protective equipment, when providing care for patients in the residency program	80,2%	19,8%	100,0%
		% do Total	63,1%	15,5%	78,6%
Total		Contagem	1012	301	1313
		% em Perceived adequacy of the availability of personal protective equipment, when providing care for patients in the residency program	77,1%	22,9%	100,0%
		% do Total	77,1%	22,9%	100,0%

Testes qui-quadrado

	Valor	gl	Significância Assintótica (Bilateral)	Sig exata (2 lados)	Sig exata (1 lado)
Qui-quadrado de Pearson	27,203[a]	1	,000		
Correção de continuidade[b]	26,374	1	,000		
Razão de verossimilhança	25,437	1	,000		
Teste Exato de Fisher				,000	,000
Associação Linear por Linear	27,182	1	,000		
N de Casos Válidos	1313				

a. 0 células (,0%) esperavam uma contagem menor que 5. A contagem mínima esperada é 64,42.

b. Computado apenas para uma tabela 2x2

Medidas Simétricas

		Valor	Significância Aproximada
Nominal por Nominal	Fi	-,144	,000
	V de Cramer	,144	,000
N de Casos Válidos		1313	

Estimativa de Risco

Valor	Intervalo de confiança de 95%

		Inferior	Superior
Razão de Chances para Perceived adequacy of the availability of personal protective equipment, when providing care for patients in the residency program (Poor adequacy / Good adequacy)	,467	,350	,624
Para grupo DASS21 Classification - Stress (dichotomous) = Normal	,816	,746	,893
Para grupo DASS21 Classification - Stress (dichotomous) = Abnormal (elevated)	1,746	1,426	2,138
N de Casos Válidos	1313		

```
Perceived adequacy of the availability of personal protective equipment, when providing care for patients
in the residency program * PHQ9 Depression Classification - Risk estimate for having current major
depressive disorder
```

Crosstab

			PHQ9 Depression Classification - Risk estimate for having current major depressive disorder		
			Low	High	Total
Perceived adequacy of the availability of personal protective equipment, when providing care for patients in the residency program	Poor adequacy	Contagem	59	222	281
		% em Perceived adequacy of the availability of personal protective equipment, when providing care for patients in the residency program	21,0%	79,0%	100,0%
		% do Total	4,5%	16,9%	21,4%
	Good adequacy	Contagem	370	662	1032
		% em Perceived adequacy of the availability of personal protective equipment, when providing care for patients in the residency program	35,9%	64,1%	100,0%
		% do Total	28,2%	50,4%	78,6%
Total		Contagem	429	884	1313
		% em Perceived adequacy of the availability of personal protective equipment, when providing care for patients in the residency program	32,7%	67,3%	100,0%

% do Total	32,7%	67,3%	100,0%

Testes qui-quadrado

	Valor	gl	Significância Assintótica (Bilateral)	Sig exata (2 lados)	Sig exata (1 lado)
Qui-quadrado de Pearson	22,160[a]	1	,000		
Correção de continuidade[b]	21,489	1	,000		
Razão de verossimilhança	23,500	1	,000		
Teste Exato de Fisher				,000	,000
Associação Linear por Linear	22,143	1	,000		
N de Casos Válidos	1313				

a. 0 células (,0%) esperavam uma contagem menor que 5. A contagem mínima esperada é 91,81.

b. Computado apenas para uma tabela 2x2

Medidas Simétricas

		Valor	Significância Aproximada
Nominal por Nominal	Fi	-,130	,000
	V de Cramer	,130	,000
N de Casos Válidos		1313	

Estimativa de Risco

	Valor	Intervalo de confiança de 95%	
		Inferior	Superior
Razão de Chances para Perceived adequacy of the availability of personal protective equipment, when providing care for patients in the residency program (Poor adequacy / Good adequacy)	,476	,347	,651
Para grupo PHQ9 Depression Classification - Risk estimate for having current major depressive disorder = Low	,586	,460	,745
Para grupo PHQ9 Depression Classification - Risk estimate for having current major depressive disorder = High	1,232	1,142	1,328
N de Casos Válidos	1313		

Perceived adequacy of the availability of personal protective equipment, when providing care for patients in the residency program * BRCS Classification

Crosstab

			BRCS Classification		
			Low resilience	Moderate to High	Total
Perceived adequacy of the availability of personal protective equipment, when providing care for patients in the residency program	Poor adequacy	Contagem	193	88	281
		% em Perceived adequacy of the availability of personal protective equipment, when providing care for patients in the residency program	68,7%	31,3%	100,0%
		% do Total	14,7%	6,7%	21,4%
	Good adequacy	Contagem	620	412	1032
		% em Perceived adequacy of the availability of personal protective equipment, when providing care for patients in the residency program	60,1%	39,9%	100,0%
		% do Total	47,2%	31,4%	78,6%
Total		Contagem	813	500	1313
		% em Perceived adequacy of the availability of personal protective equipment, when providing care for patients in the residency program	61,9%	38,1%	100,0%
		% do Total	61,9%	38,1%	100,0%

Testes qui-quadrado

	Valor	gl	Significância Assintótica (Bilateral)	Sig exata (2 lados)	Sig exata (1 lado)
Qui-quadrado de Pearson	6,937[a]	1	,008		
Correção de continuidade[b]	6,577	1	,010		
Razão de verossimilhança	7,076	1	,008		
Teste Exato de Fisher				,008	,005
Associação Linear por Linear	6,932	1	,008		
N de Casos Válidos	1313				

a. 0 células (,0%) esperavam uma contagem menor que 5. A contagem mínima esperada é 107,01.

b. Computado apenas para uma tabela 2x2

Medidas Simétricas

		Valor	Significância Aproximada
Nominal por Nominal	Fi	,073	,008
	V de Cramer	,073	,008
N de Casos Válidos		1313	

Estimativa de Risco

	Valor	Intervalo de confiança de 95% Inferior	Superior
Razão de Chances para Perceived adequacy of the availability of personal protective equipment, when providing care for patients in the residency program (Poor adequacy / Good adequacy)	1,457	1,100	1,931
Para grupo BRCS Classification = Low resilience	1,143	1,041	1,255
Para grupo BRCS Classification = Moderate to High	,784	,650	,947
N de Casos Válidos	1313		

Perceived adequacy of the availability of personal protective equipment, when providing care for patients in the residency program * OLBI Score Classification

Crosstab

			OLBI Score Classification Low to Moderate	High	Total
Perceived adequacy of the availability of personal protective equipment, when providing care for patients in the residency program	Poor adequacy	Contagem	141	140	281
		% em Perceived adequacy of the availability of personal protective equipment, when providing care for patients in the residency program	50,2%	49,8%	100,0%
		% do Total	10,7%	10,7%	21,4%
	Good adequacy	Contagem	734	298	1032
		% em Perceived adequacy of the availability of personal protective equipment, when providing care for patients in the residency program	71,1%	28,9%	100,0%
		% do Total	55,9%	22,7%	78,6%

Total	Contagem	875	438	1313
	% em Perceived adequacy of the availability of personal protective equipment, when providing care for patients in the residency program	66,6%	33,4%	100,0%
	% do Total	66,6%	33,4%	100,0%

Testes qui-quadrado

	Valor	gl	Significância Assintótica (Bilateral)	Sig exata (2 lados)	Sig exata (1 lado)
Qui-quadrado de Pearson	43,589[a]	1	,000		
Correção de continuidade[b]	42,652	1	,000		
Razão de verossimilhança	41,862	1	,000		
Teste Exato de Fisher				,000	,000
Associação Linear por Linear	43,556	1	,000		
N de Casos Válidos	1313				

a. 0 células (,0%) esperavam uma contagem menor que 5. A contagem mínima esperada é 93,74.

b. Computado apenas para uma tabela 2x2

Medidas Simétricas

		Valor	Significância Aproximada
Nominal por Nominal	Fi	-,182	,000
	V de Cramer	,182	,000
N de Casos Válidos		1313	

Estimativa de Risco

	Valor	Intervalo de confiança de 95% Inferior	Superior
Razão de Chances para Perceived adequacy of the availability of personal protective equipment, when providing care for patients in the residency program (Poor adequacy / Good adequacy)	,409	,312	,536
Para grupo OLBI Score Classification = Low to Moderate	,705	,624	,798
Para grupo OLBI Score Classification = High	1,725	1,483	2,008
N de Casos Válidos	1313		

Cumulative weekly workload < or >= 60h (dichotomous) * DASS21 Classification - Depression (dichotomous)

Crosstab

			DASS21 Classification - Depression (dichotomous)		
			Normal	Abnormal (elevated)	Total
Cumulative weekly workload < or >= 60h (dichotomous)	<=60h	Contagem	373	168	541
		% em Cumulative weekly workload < or >= 60h (dichotomous)	68,9%	31,1%	100,0%
		% do Total	28,4%	12,8%	41,2%
	>60h	Contagem	497	275	772
		% em Cumulative weekly workload < or >= 60h (dichotomous)	64,4%	35,6%	100,0%
		% do Total	37,9%	20,9%	58,8%
Total		Contagem	870	443	1313
		% em Cumulative weekly workload < or >= 60h (dichotomous)	66,3%	33,7%	100,0%
		% do Total	66,3%	33,7%	100,0%

Testes qui-quadrado

	Valor	gl	Significância Assintótica (Bilateral)	Sig exata (2 lados)	Sig exata (1 lado)
Qui-quadrado de Pearson	2,969[a]	1	,085		
Correção de continuidade[b]	2,768	1	,096		
Razão de verossimilhança	2,983	1	,084		
Teste Exato de Fisher				,086	,048
Associação Linear por Linear	2,967	1	,085		
N de Casos Válidos	1313				

a. 0 células (,0%) esperavam uma contagem menor que 5. A contagem mínima esperada é 182,53.

b. Computado apenas para uma tabela 2x2

Medidas Simétricas

	Valor	Significância Aproximada

Nominal por Nominal	Fi	,048	,085
	V de Cramer	,048	,085
N de Casos Válidos		1313	

Estimativa de Risco

	Valor	Intervalo de confiança de 95% Inferior	Superior
Razão de Chances para Cumulative weekly workload < or >= 60h (dichotomous) (<=60h / >60h)	1,229	,972	1,553
Para grupo DASS21 Classification - Depression (dichotomous) = Normal	1,071	,991	1,157
Para grupo DASS21 Classification - Depression (dichotomous) = Abnormal (elevated)	,872	,745	1,020
N de Casos Válidos	1313		

Cumulative weekly workload < or >= 60h (dichotomous) * DASS21 Classification - Anxiety (dichotomous)

Crosstab

			DASS21 Classification - Anxiety (dichotomous) Normal	Abnormal (elevated)	Total
Cumulative weekly workload < or >= 60h (dichotomous)	<=60h	Contagem	355	186	541
		% em Cumulative weekly workload < or >= 60h (dichotomous)	65,6%	34,4%	100,0%
		% do Total	27,0%	14,2%	41,2%
	>60h	Contagem	506	266	772
		% em Cumulative weekly workload < or >= 60h (dichotomous)	65,5%	34,5%	100,0%
		% do Total	38,5%	20,3%	58,8%
Total		Contagem	861	452	1313
		% em Cumulative weekly workload < or >= 60h (dichotomous)	65,6%	34,4%	100,0%
		% do Total	65,6%	34,4%	100,0%

Testes qui-quadrado

	Valor	gl	Significância Assintótica (Bilateral)	Sig exata (2 lados)	Sig exata (1 lado)
Qui-quadrado de Pearson	,001[a]	1	,977		
Correção de continuidade[b]	,000	1	1,000		
Razão de verossimilhança	,001	1	,977		
Teste Exato de Fisher				1,000	,513
Associação Linear por Linear	,001	1	,977		
N de Casos Válidos	1313				

a. 0 células (,0%) esperavam uma contagem menor que 5. A contagem mínima esperada é 186,24.

b. Computado apenas para uma tabela 2x2

Medidas Simétricas

		Valor	Significância Aproximada
Nominal por Nominal	Fi	,001	,977
	V de Cramer	,001	,977
N de Casos Válidos		1313	

Estimativa de Risco

	Valor	Intervalo de confiança de 95%	
		Inferior	Superior
Razão de Chances para Cumulative weekly workload < or >= 60h (dichotomous) (<=60h / >60h)	1,003	,796	1,264
Para grupo DASS21 Classification - Anxiety (dichotomous) = Normal	1,001	,925	1,084
Para grupo DASS21 Classification - Anxiety (dichotomous) = Abnormal (elevated)	,998	,857	1,161
N de Casos Válidos	1313		

Cumulative weekly workload < or >= 60h (dichotomous) * DASS21 Classification - Stress (dichotomous)

Crosstab

	DASS21 Classification - Stress (dichotomous)	Total

			Normal	Abnormal (elevated)	
Cumulative weekly workload < or >= 60h (dichotomous)	<=60h	Contagem	417	124	541
		% em Cumulative weekly workload < or >= 60h (dichotomous)	77,1%	22,9%	100,0%
		% do Total	31,8%	9,4%	41,2%
	>60h	Contagem	595	177	772
		% em Cumulative weekly workload < or >= 60h (dichotomous)	77,1%	22,9%	100,0%
		% do Total	45,3%	13,5%	58,8%
Total		Contagem	1012	301	1313
		% em Cumulative weekly workload < or >= 60h (dichotomous)	77,1%	22,9%	100,0%
		% do Total	77,1%	22,9%	100,0%

Testes qui-quadrado

	Valor	gl	Significância Assintótica (Bilateral)	Sig exata (2 lados)	Sig exata (1 lado)
Qui-quadrado de Pearson	,000[a]	1	,998		
Correção de continuidade[b]	,000	1	1,000		
Razão de verossimilhança	,000	1	,998		
Teste Exato de Fisher				1,000	,526
Associação Linear por Linear	,000	1	,998		
N de Casos Válidos	1313				

a. 0 células (,0%) esperavam uma contagem menor que 5. A contagem mínima esperada é 124,02.

b. Computado apenas para uma tabela 2x2

Medidas Simétricas

		Valor	Significância Aproximada
Nominal por Nominal	Fi	,000	,998
	V de Cramer	,000	,998
N de Casos Válidos		1313	

Estimativa de Risco

	Valor	Intervalo de confiança de 95%	
		Inferior	Superior

Razão de Chances para Cumulative weekly workload < or >= 60h (dichotomous) (<=60h / >60h)	1,000	,770	1,299
Para grupo DASS21 Classification - Stress (dichotomous) = Normal	1,000	,942	1,062
Para grupo DASS21 Classification - Stress (dichotomous) = Abnormal (elevated)	1,000	,817	1,223
N de Casos Válidos	1313		

```
Cumulative weekly workload < or >= 60h (dichotomous) * PHQ9 Depression Classification - Risk estimate
for having current major depressive disorder
```

Crosstab

			PHQ9 Depression Classification - Risk estimate for having current major depressive disorder		
			Low	High	Total
Cumulative weekly workload < or >= 60h (dichotomous)	<=60h	Contagem	198	343	541
		% em Cumulative weekly workload < or >= 60h (dichotomous)	36,6%	63,4%	100,0%
		% do Total	15,1%	26,1%	41,2%
	>60h	Contagem	231	541	772
		% em Cumulative weekly workload < or >= 60h (dichotomous)	29,9%	70,1%	100,0%
		% do Total	17,6%	41,2%	58,8%
Total		Contagem	429	884	1313
		% em Cumulative weekly workload < or >= 60h (dichotomous)	32,7%	67,3%	100,0%
		% do Total	32,7%	67,3%	100,0%

Testes qui-quadrado

	Valor	gl	Significância Assintótica (Bilateral)	Sig exata (2 lados)	Sig exata (1 lado)
Qui-quadrado de Pearson	6,446[a]	1	,011		
Correção de continuidade[b]	6,146	1	,013		
Razão de verossimilhança	6,415	1	,011		

Teste Exato de Fisher				,012	,007
Associação Linear por Linear	6,441	1	,011		
N de Casos Válidos	1313				

a. 0 células (,0%) esperavam uma contagem menor que 5. A contagem mínima esperada é 176,76.

b. Computado apenas para uma tabela 2x2

Medidas Simétricas

		Valor	Significância Aproximada
Nominal por Nominal	Fi	,070	,011
	V de Cramer	,070	,011
N de Casos Válidos		1313	

Estimativa de Risco

	Valor	Intervalo de confiança de 95% Inferior	Intervalo de confiança de 95% Superior
Razão de Chances para Cumulative weekly workload < or >= 60h (dichotomous) (<=60h / >60h)	1,352	1,071	1,707
Para grupo PHQ9 Depression Classification - Risk estimate for having current major depressive disorder = Low	1,223	1,048	1,428
Para grupo PHQ9 Depression Classification - Risk estimate for having current major depressive disorder = High	,905	,836	,979
N de Casos Válidos	1313		

Cumulative weekly workload < or >= 60h (dichotomous) * BRCS Classification

Crosstab

			BRCS Classification		
			Low resilience	Moderate to High	Total
Cumulative weekly workload < or >= 60h (dichotomous)	<=60h	Contagem	346	195	541
		% em Cumulative weekly workload < or >= 60h (dichotomous)	64,0%	36,0%	100,0%
		% do Total	26,4%	14,9%	41,2%
	>60h	Contagem	467	305	772

	% em Cumulative weekly workload < or >= 60h (dichotomous)	60,5%	39,5%	100,0%
	% do Total	35,6%	23,2%	58,8%
Total	Contagem	813	500	1313
	% em Cumulative weekly workload < or >= 60h (dichotomous)	61,9%	38,1%	100,0%
	% do Total	61,9%	38,1%	100,0%

Testes qui-quadrado

	Valor	gl	Significância Assintótica (Bilateral)	Sig exata (2 lados)	Sig exata (1 lado)
Qui-quadrado de Pearson	1,618[a]	1	,203		
Correção de continuidade[b]	1,475	1	,225		
Razão de verossimilhança	1,622	1	,203		
Teste Exato de Fisher				,205	,112
Associação Linear por Linear	1,617	1	,204		
N de Casos Válidos	1313				

a. 0 células (,0%) esperavam uma contagem menor que 5. A contagem mínima esperada é 206,02.

b. Computado apenas para uma tabela 2x2

Medidas Simétricas

		Valor	Significância Aproximada
Nominal por Nominal	Fi	,035	,203
	V de Cramer	,035	,203
N de Casos Válidos		1313	

Estimativa de Risco

	Valor	Intervalo de confiança de 95% Inferior	Superior
Razão de Chances para Cumulative weekly workload < or >= 60h (dichotomous) (<=60h / >60h)	1,159	,923	1,454
Para grupo BRCS Classification = Low resilience	1,057	,971	1,151
Para grupo BRCS Classification = Moderate to High	,912	,791	1,052
N de Casos Válidos	1313		

```
Cumulative weekly workload < or >= 60h (dichotomous) * OLBI Score Classification
```

Crosstab

			OLBI Score Classification		
			Low to Moderate	High	Total
Cumulative weekly workload < or >= 60h (dichotomous)	<=60h	Contagem	380	161	541
		% em Cumulative weekly workload < or >= 60h (dichotomous)	70,2%	29,8%	100,0%
		% do Total	28,9%	12,3%	41,2%
	>60h	Contagem	495	277	772
		% em Cumulative weekly workload < or >= 60h (dichotomous)	64,1%	35,9%	100,0%
		% do Total	37,7%	21,1%	58,8%
Total		Contagem	875	438	1313
		% em Cumulative weekly workload < or >= 60h (dichotomous)	66,6%	33,4%	100,0%
		% do Total	66,6%	33,4%	100,0%

Testes qui-quadrado

	Valor	gl	Significância Assintótica (Bilateral)	Sig exata (2 lados)	Sig exata (1 lado)
Qui-quadrado de Pearson	5,361[a]	1	,021		
Correção de continuidade[b]	5,089	1	,024		
Razão de verossimilhança	5,396	1	,020		
Teste Exato de Fisher				,024	,012
Associação Linear por Linear	5,357	1	,021		
N de Casos Válidos	1313				

a. 0 células (,0%) esperavam uma contagem menor que 5. A contagem mínima esperada é 180,47.

b. Computado apenas para uma tabela 2x2

Medidas Simétricas

		Valor	Significância Aproximada
Nominal por Nominal	Fi	,064	,021
	V de Cramer	,064	,021

N de Casos Válidos	1313	

Estimativa de Risco

	Valor	Intervalo de confiança de 95% Inferior	Superior
Razão de Chances para Cumulative weekly workload < or >= 60h (dichotomous) (<=60h / >60h)	1,321	1,043	1,672
Para grupo OLBI Score Classification = Low to Moderate	1,095	1,015	1,182
Para grupo OLBI Score Classification = High	,829	,707	,973
N de Casos Válidos	1313		

```
Cumulative weekly workload < or >= 90h (dichotomous) * DASS21 Classification - Depression (dichotomous)
```

Crosstab

			DASS21 Classification - Depression (dichotomous)		
			Normal	Abnormal (elevated)	Total
Cumulative weekly workload < or >= 90h (dichotomous)	<=90H	Contagem	824	399	1223
		% em Cumulative weekly workload < or >= 90h (dichotomous)	67,4%	32,6%	100,0%
		% do Total	62,8%	30,4%	93,1%
	>90H	Contagem	46	44	90
		% em Cumulative weekly workload < or >= 90h (dichotomous)	51,1%	48,9%	100,0%
		% do Total	3,5%	3,4%	6,9%
Total		Contagem	870	443	1313
		% em Cumulative weekly workload < or >= 90h (dichotomous)	66,3%	33,7%	100,0%
		% do Total	66,3%	33,7%	100,0%

Testes qui-quadrado

	Valor	gl	Significância Assintótica (Bilateral)	Sig exata (2 lados)	Sig exata (1 lado)
Qui-quadrado de Pearson	9,919[a]	1	,002		
Correção de continuidade[b]	9,205	1	,002		
Razão de verossimilhança	9,439	1	,002		
Teste Exato de Fisher				,002	,001
Associação Linear por Linear	9,912	1	,002		
N de Casos Válidos	1313				

a. 0 células (,0%) esperavam uma contagem menor que 5. A contagem mínima esperada é 30,37.

b. Computado apenas para uma tabela 2x2

Medidas Simétricas

		Valor	Significância Aproximada
Nominal por Nominal	Fi	,087	,002
	V de Cramer	,087	,002
N de Casos Válidos		1313	

Estimativa de Risco

	Valor	Intervalo de confiança de 95% Inferior	Superior
Razão de Chances para Cumulative weekly workload < or >= 90h (dichotomous) (<=90H / >90H)	1,975	1,285	3,037
Para grupo DASS21 Classification - Depression (dichotomous) = Normal	1,318	1,073	1,619
Para grupo DASS21 Classification - Depression (dichotomous) = Abnormal (elevated)	,667	,532	,837
N de Casos Válidos	1313		

Cumulative weekly workload < or >= 90h (dichotomous) * DASS21 Classification - Anxiety (dichotomous)

Crosstab

	DASS21 Classification - Anxiety (dichotomous) Normal	Abnormal (elevated)	Total

Cumulative weekly workload < or >= 90h (dichotomous)	<=90H	Contagem	803	420	1223
		% em Cumulative weekly workload < or >= 90h (dichotomous)	65,7%	34,3%	100,0%
		% do Total	61,2%	32,0%	93,1%
	>90H	Contagem	58	32	90
		% em Cumulative weekly workload < or >= 90h (dichotomous)	64,4%	35,6%	100,0%
		% do Total	4,4%	2,4%	6,9%
Total		Contagem	861	452	1313
		% em Cumulative weekly workload < or >= 90h (dichotomous)	65,6%	34,4%	100,0%
		% do Total	65,6%	34,4%	100,0%

Testes qui-quadrado

	Valor	gl	Significância Assintótica (Bilateral)	Sig exata (2 lados)	Sig exata (1 lado)
Qui-quadrado de Pearson	,055[a]	1	,815		
Correção de continuidade[b]	,014	1	,905		
Razão de verossimilhança	,054	1	,815		
Teste Exato de Fisher				,819	,449
Associação Linear por Linear	,055	1	,815		
N de Casos Válidos	1313				

a. 0 células (,0%) esperavam uma contagem menor que 5. A contagem mínima esperada é 30,98.

b. Computado apenas para uma tabela 2x2

Medidas Simétricas

		Valor	Significância Aproximada
Nominal por Nominal	Fi	,006	,815
	V de Cramer	,006	,815
N de Casos Válidos		1313	

Estimativa de Risco

	Valor	Intervalo de confiança de 95%	
		Inferior	Superior
Razão de Chances para Cumulative weekly workload < or >= 90h (dichotomous) (<=90H / >90H)	1,055	,674	1,650

Para grupo DASS21 Classification - Anxiety (dichotomous) = Normal	1,019	,869	1,194
Para grupo DASS21 Classification - Anxiety (dichotomous) = Abnormal (elevated)	,966	,724	1,289
N de Casos Válidos	1313		

```
Cumulative weekly workload < or >= 90h (dichotomous) * DASS21 Classification - Stress (dichotomous)
```

Crosstab

			DASS21 Classification - Stress (dichotomous)		
			Normal	Abnormal (elevated)	Total
Cumulative weekly workload < or >= 90h (dichotomous)	<=90H	Contagem	953	270	1223
		% em Cumulative weekly workload < or >= 90h (dichotomous)	77,9%	22,1%	100,0%
		% do Total	72,6%	20,6%	93,1%
	>90H	Contagem	59	31	90
		% em Cumulative weekly workload < or >= 90h (dichotomous)	65,6%	34,4%	100,0%
		% do Total	4,5%	2,4%	6,9%
Total		Contagem	1012	301	1313
		% em Cumulative weekly workload < or >= 90h (dichotomous)	77,1%	22,9%	100,0%
		% do Total	77,1%	22,9%	100,0%

Testes qui-quadrado

	Valor	gl	Significância Assintótica (Bilateral)	Sig exata (2 lados)	Sig exata (1 lado)
Qui-quadrado de Pearson	7,257[a]	1	,007		
Correção de continuidade[b]	6,574	1	,010		
Razão de verossimilhança	6,642	1	,010		
Teste Exato de Fisher				,009	,007
Associação Linear por Linear	7,251	1	,007		
N de Casos Válidos	1313				

a. 0 células (,0%) esperavam uma contagem menor que 5. A contagem mínima esperada é 20,63.

b. Computado apenas para uma tabela 2x2

Medidas Simétricas

		Valor	Significância Aproximada
Nominal por Nominal	Fi	,074	,007
	V de Cramer	,074	,007
N de Casos Válidos		1313	

Estimativa de Risco

	Valor	Intervalo de confiança de 95% Inferior	Superior
Razão de Chances para Cumulative weekly workload < or >= 90h (dichotomous) (<=90H / >90H)	1,855	1,176	2,924
Para grupo DASS21 Classification - Stress (dichotomous) = Normal	1,189	1,020	1,385
Para grupo DASS21 Classification - Stress (dichotomous) = Abnormal (elevated)	,641	,473	,869
N de Casos Válidos	1313		

```
Cumulative weekly workload < or >= 90h (dichotomous) * PHQ9 Depression Classification - Risk estimate
for having current major depressive disorder
```

Crosstab

			PHQ9 Depression Classification - Risk estimate for having current major depressive disorder: Low	High	Total
Cumulative weekly workload < or >= 90h (dichotomous)	<=90H	Contagem	406	817	1223
		% em Cumulative weekly workload < or >= 90h (dichotomous)	33,2%	66,8%	100,0%
		% do Total	30,9%	62,2%	93,1%
	>90H	Contagem	23	67	90
		% em Cumulative weekly workload < or >= 90h (dichotomous)	25,6%	74,4%	100,0%
		% do Total	1,8%	5,1%	6,9%
Total		Contagem	429	884	1313

	% em Cumulative weekly workload < or >= 90h (dichotomous)	32,7%	67,3%	100,0%
	% do Total	32,7%	67,3%	100,0%

Testes qui-quadrado

	Valor	gl	Significância Assintótica (Bilateral)	Sig exata (2 lados)	Sig exata (1 lado)
Qui-quadrado de Pearson	2,225[a]	1	,136		
Correção de continuidade[b]	1,891	1	,169		
Razão de verossimilhança	2,316	1	,128		
Teste Exato de Fisher				,162	,083
Associação Linear por Linear	2,224	1	,136		
N de Casos Válidos	1313				

a. 0 células (,0%) esperavam uma contagem menor que 5. A contagem mínima esperada é 29,41.

b. Computado apenas para uma tabela 2x2

Medidas Simétricas

		Valor	Significância Aproximada
Nominal por Nominal	Fi	,041	,136
	V de Cramer	,041	,136
N de Casos Válidos		1313	

Estimativa de Risco

	Valor	Intervalo de confiança de 95% Inferior	Superior
Razão de Chances para Cumulative weekly workload < or >= 90h (dichotomous) (<=90H / >90H)	1,448	,888	2,359
Para grupo PHQ9 Depression Classification - Risk estimate for having current major depressive disorder = Low	1,299	,905	1,865
Para grupo PHQ9 Depression Classification - Risk estimate for having current major depressive disorder = High	,897	,790	1,019
N de Casos Válidos	1313		

```
Cumulative weekly workload < or >= 90h (dichotomous) * BRCS Classification
```

Crosstab

			BRCS Classification: Low resilience	BRCS Classification: Moderate to High	Total
Cumulative weekly workload < or >= 90h (dichotomous)	<=90H	Contagem	753	470	1223
		% em Cumulative weekly workload < or >= 90h (dichotomous)	61,6%	38,4%	100,0%
		% do Total	57,3%	35,8%	93,1%
	>90H	Contagem	60	30	90
		% em Cumulative weekly workload < or >= 90h (dichotomous)	66,7%	33,3%	100,0%
		% do Total	4,6%	2,3%	6,9%
Total		Contagem	813	500	1313
		% em Cumulative weekly workload < or >= 90h (dichotomous)	61,9%	38,1%	100,0%
		% do Total	61,9%	38,1%	100,0%

Testes qui-quadrado

	Valor	gl	Significância Assintótica (Bilateral)	Sig exata (2 lados)	Sig exata (1 lado)
Qui-quadrado de Pearson	,924[a]	1	,337		
Correção de continuidade[b]	,720	1	,396		
Razão de verossimilhança	,939	1	,333		
Teste Exato de Fisher				,369	,199
Associação Linear por Linear	,923	1	,337		
N de Casos Válidos	1313				

a. 0 células (,0%) esperavam uma contagem menor que 5. A contagem mínima esperada é 34,27.

b. Computado apenas para uma tabela 2x2

Medidas Simétricas

		Valor	Significância Aproximada
Nominal por Nominal	Fi	-,027	,337
	V de Cramer	,027	,337
N de Casos Válidos		1313	

Estimativa de Risco

	Valor	Intervalo de confiança de 95% Inferior	Superior
Razão de Chances para Cumulative weekly workload < or >= 90h (dichotomous) (<=90H / >90H)	,801	,509	1,260
Para grupo BRCS Classification = Low resilience	,924	,793	1,076
Para grupo BRCS Classification = Moderate to High	1,153	,854	1,557
N de Casos Válidos	1313		

```
Cumulative weekly workload < or >= 90h (dichotomous) * OLBI Score Classification
```

Crosstab

			OLBI Score Classification		
			Low to Moderate	High	Total
Cumulative weekly workload < or >= 90h (dichotomous)	<=90H	Contagem	829	394	1223
		% em Cumulative weekly workload < or >= 90h (dichotomous)	67,8%	32,2%	100,0%
		% do Total	63,1%	30,0%	93,1%
	>90H	Contagem	46	44	90
		% em Cumulative weekly workload < or >= 90h (dichotomous)	51,1%	48,9%	100,0%
		% do Total	3,5%	3,4%	6,9%
Total		Contagem	875	438	1313
		% em Cumulative weekly workload < or >= 90h (dichotomous)	66,6%	33,4%	100,0%
		% do Total	66,6%	33,4%	100,0%

Testes qui-quadrado

	Valor	gl	Significância Assintótica (Bilateral)	Sig exata (2 lados)	Sig exata (1 lado)
Qui-quadrado de Pearson	10,483[a]	1	,001		
Correção de continuidade[b]	9,746	1	,002		

Razão de verossimilhança	9,949	1	,002		
Teste Exato de Fisher				,002	,001
Associação Linear por Linear	10,475	1	,001		
N de Casos Válidos	1313				

a. 0 células (,0%) esperavam uma contagem menor que 5. A contagem mínima esperada é 30,02.

b. Computado apenas para uma tabela 2x2

Medidas Simétricas

		Valor	Significância Aproximada
Nominal por Nominal	Fi	,089	,001
	V de Cramer	,089	,001
N de Casos Válidos		1313	

Estimativa de Risco

	Valor	Intervalo de confiança de 95%	
		Inferior	Superior
Razão de Chances para Cumulative weekly workload < or >= 90h (dichotomous) (<=90H / >90H)	2,013	1,309	3,095
Para grupo OLBI Score Classification = Low to Moderate	1,326	1,080	1,629
Para grupo OLBI Score Classification = High	,659	,525	,826
N de Casos Válidos	1313		

The participant exerts professional activity external to the residency program * DASS21 Classification - Depression (dichotomous)

Crosstab

			DASS21 Classification - Depression (dichotomous)		Total
			Normal	Abnormal (elevated)	
The participant exerts professional activity external to the residency program	No	Contagem	584	305	889
		% em The participant exerts professional activity external to the residency program	65,7%	34,3%	100,0%
		% do Total	44,5%	23,2%	67,7%
	Yes	Contagem	286	138	424

	% em The participant exerts professional activity external to the residency program	67,5%	32,5%	100,0%
	% do Total	21,8%	10,5%	32,3%
Total	Contagem	870	443	1313
	% em The participant exerts professional activity external to the residency program	66,3%	33,7%	100,0%
	% do Total	66,3%	33,7%	100,0%

Testes qui-quadrado

	Valor	gl	Significância Assintótica (Bilateral)	Sig exata (2 lados)	Sig exata (1 lado)
Qui-quadrado de Pearson	,398[a]	1	,528		
Correção de continuidade[b]	,323	1	,570		
Razão de verossimilhança	,399	1	,527		
Teste Exato de Fisher				,533	,285
Associação Linear por Linear	,398	1	,528		
N de Casos Válidos	1313				

a. 0 células (,0%) esperavam uma contagem menor que 5. A contagem mínima esperada é 143,06.

b. Computado apenas para uma tabela 2x2

Medidas Simétricas

		Valor	Significância Aproximada
Nominal por Nominal	Fi	-,017	,528
	V de Cramer	,017	,528
N de Casos Válidos		1313	

Estimativa de Risco

	Valor	Intervalo de confiança de 95% Inferior	Superior
Razão de Chances para The participant exerts professional activity external to the residency program (No / Yes)	,924	,723	1,181
Para grupo DASS21 Classification - Depression (dichotomous) = Normal	,974	,898	1,056
Para grupo DASS21 Classification - Depression (dichotomous) = Abnormal (elevated)	1,054	,894	1,243

N de Casos Válidos	1313		

```
The participant exerts professional activity external to the residency program * DASS21 Classification
- Anxiety (dichotomous)
```

Crosstab

			DASS21 Classification - Anxiety (dichotomous)		
			Normal	Abnormal (elevated)	Total
The participant exerts professional activity external to the residency program	No	Contagem	563	326	889
		% em The participant exerts professional activity external to the residency program	63,3%	36,7%	100,0%
		% do Total	42,9%	24,8%	67,7%
	Yes	Contagem	298	126	424
		% em The participant exerts professional activity external to the residency program	70,3%	29,7%	100,0%
		% do Total	22,7%	9,6%	32,3%
Total		Contagem	861	452	1313
		% em The participant exerts professional activity external to the residency program	65,6%	34,4%	100,0%
		% do Total	65,6%	34,4%	100,0%

Testes qui-quadrado

	Valor	gl	Significância Assintótica (Bilateral)	Sig exata (2 lados)	Sig exata (1 lado)
Qui-quadrado de Pearson	6,149[a]	1	,013		
Correção de continuidade[b]	5,845	1	,016		
Razão de verossimilhança	6,230	1	,013		
Teste Exato de Fisher				,013	,008
Associação Linear por Linear	6,144	1	,013		
N de Casos Válidos	1313				

a. 0 células (,0%) esperavam uma contagem menor que 5. A contagem mínima esperada é 145,96.

b. Computado apenas para uma tabela 2x2

Medidas Simétricas

		Valor	Significância Aproximada
Nominal por Nominal	Fi	-,068	,013
	V de Cramer	,068	,013
N de Casos Válidos		1313	

Estimativa de Risco

	Valor	Intervalo de confiança de 95%	
		Inferior	Superior
Razão de Chances para The participant exerts professional activity external to the residency program (No / Yes)	,730	,569	,937
Para grupo DASS21 Classification - Anxiety (dichotomous) = Normal	,901	,832	,976
Para grupo DASS21 Classification - Anxiety (dichotomous) = Abnormal (elevated)	1,234	1,041	1,463
N de Casos Válidos	1313		

```
The participant exerts professional activity external to the residency program * DASS21 Classification
- Stress (dichotomous)
```

Crosstab

			DASS21 Classification - Stress (dichotomous)		Total
			Normal	Abnormal (elevated)	
The participant exerts professional activity external to the residency program	No	Contagem	680	209	889
		% em The participant exerts professional activity external to the residency program	76,5%	23,5%	100,0%
		% do Total	51,8%	15,9%	67,7%
	Yes	Contagem	332	92	424
		% em The participant exerts professional activity external to the residency program	78,3%	21,7%	100,0%
		% do Total	25,3%	7,0%	32,3%
Total		Contagem	1012	301	1313
		% em The participant exerts professional activity external to the residency program	77,1%	22,9%	100,0%

% do Total	77,1%	22,9%	100,0%

Testes qui-quadrado

	Valor	gl	Significância Assintótica (Bilateral)	Sig exata (2 lados)	Sig exata (1 lado)
Qui-quadrado de Pearson	,533[a]	1	,465		
Correção de continuidade[b]	,436	1	,509		
Razão de verossimilhança	,537	1	,464		
Teste Exato de Fisher				,483	,255
Associação Linear por Linear	,533	1	,465		
N de Casos Válidos	1313				

a. 0 células (,0%) esperavam uma contagem menor que 5. A contagem mínima esperada é 97,20.

b. Computado apenas para uma tabela 2x2

Medidas Simétricas

		Valor	Significância Aproximada
Nominal por Nominal	Fi	-,020	,465
	V de Cramer	,020	,465
N de Casos Válidos		1313	

Estimativa de Risco

	Valor	Intervalo de confiança de 95%	
		Inferior	Superior
Razão de Chances para The participant exerts professional activity external to the residency program (No / Yes)	,902	,683	1,191
Para grupo DASS21 Classification - Stress (dichotomous) = Normal	,977	,918	1,039
Para grupo DASS21 Classification - Stress (dichotomous) = Abnormal (elevated)	1,083	,873	1,345
N de Casos Válidos	1313		

The participant exerts professional activity external to the residency program * PHQ9 Depression Classification - Risk estimate for having current major depressive disorder

Crosstab

			PHQ9 Depression Classification - Risk estimate for having current major depressive disorder		
			Low	High	Total
The participant exerts professional activity external to the residency program	No	Contagem	282	607	889
		% em The participant exerts professional activity external to the residency program	31,7%	68,3%	100,0%
		% do Total	21,5%	46,2%	67,7%
	Yes	Contagem	147	277	424
		% em The participant exerts professional activity external to the residency program	34,7%	65,3%	100,0%
		% do Total	11,2%	21,1%	32,3%
Total		Contagem	429	884	1313
		% em The participant exerts professional activity external to the residency program	32,7%	67,3%	100,0%
		% do Total	32,7%	67,3%	100,0%

Testes qui-quadrado

	Valor	gl	Significância Assintótica (Bilateral)	Sig exata (2 lados)	Sig exata (1 lado)
Qui-quadrado de Pearson	1,135[a]	1	,287		
Correção de continuidade[b]	1,005	1	,316		
Razão de verossimilhança	1,129	1	,288		
Teste Exato de Fisher				,286	,158
Associação Linear por Linear	1,134	1	,287		
N de Casos Válidos	1313				

a. 0 células (,0%) esperavam uma contagem menor que 5. A contagem mínima esperada é 138,53.

b. Computado apenas para uma tabela 2x2

Medidas Simétricas

		Valor	Significância Aproximada
Nominal por Nominal	Fi	-,029	,287
	V de Cramer	,029	,287
N de Casos Válidos		1313	

Estimativa de Risco

	Valor	Intervalo de confiança de 95%

		Inferior	Superior
Razão de Chances para The participant exerts professional activity external to the residency program (No / Yes)	,875	,685	1,118
Para grupo PHQ9 Depression Classification - Risk estimate for having current major depressive disorder = Low	,915	,778	1,076
Para grupo PHQ9 Depression Classification - Risk estimate for having current major depressive disorder = High	1,045	,962	1,135
N de Casos Válidos	1313		

```
The participant exerts professional activity external to the residency program * BRCS Classification
```

Crosstab

			BRCS Classification		
			Low resilience	Moderate to High	Total
The participant exerts professional activity external to the residency program	No	Contagem	569	320	889
		% em The participant exerts professional activity external to the residency program	64,0%	36,0%	100,0%
		% do Total	43,3%	24,4%	67,7%
	Yes	Contagem	244	180	424
		% em The participant exerts professional activity external to the residency program	57,5%	42,5%	100,0%
		% do Total	18,6%	13,7%	32,3%
Total		Contagem	813	500	1313
		% em The participant exerts professional activity external to the residency program	61,9%	38,1%	100,0%
		% do Total	61,9%	38,1%	100,0%

Testes qui-quadrado

	Valor	gl	Significância Assintótica (Bilateral)	Sig exata (2 lados)	Sig exata (1 lado)
Qui-quadrado de Pearson	5,077[a]	1	,024		
Correção de continuidade[b]	4,806	1	,028		

Razão de verossimilhança	5,044	1	,025		
Teste Exato de Fisher				,025	,014
Associação Linear por Linear	5,073	1	,024		
N de Casos Válidos	1313				

a. 0 células (,0%) esperavam uma contagem menor que 5. A contagem mínima esperada é 161,46.

b. Computado apenas para uma tabela 2x2

Medidas Simétricas

		Valor	Significância Aproximada
Nominal por Nominal	Fi	,062	,024
	V de Cramer	,062	,024
N de Casos Válidos		1313	

Estimativa de Risco

	Valor	Intervalo de confiança de 95%	
		Inferior	Superior
Razão de Chances para The participant exerts professional activity external to the residency program (No / Yes)	1,312	1,036	1,661
Para grupo BRCS Classification = Low resilience	1,112	1,011	1,224
Para grupo BRCS Classification = Moderate to High	,848	,736	,977
N de Casos Válidos	1313		

The participant exerts professional activity external to the residency program * OLBI Score Classification

Crosstab

			OLBI Score Classification		
			Low to Moderate	High	Total
The participant exerts professional activity external to the residency program	No	Contagem	598	291	889
		% em The participant exerts professional activity external to the residency program	67,3%	32,7%	100,0%
		% do Total	45,5%	22,2%	67,7%
	Yes	Contagem	277	147	424

	% em The participant exerts professional activity external to the residency program	65,3%	34,7%	100,0%
	% do Total	21,1%	11,2%	32,3%
Total	Contagem	875	438	1313
	% em The participant exerts professional activity external to the residency program	66,6%	33,4%	100,0%
	% do Total	66,6%	33,4%	100,0%

Testes qui-quadrado

	Valor	gl	Significância Assintótica (Bilateral)	Sig exata (2 lados)	Sig exata (1 lado)
Qui-quadrado de Pearson	,484[a]	1	,487		
Correção de continuidade[b]	,401	1	,527		
Razão de verossimilhança	,483	1	,487		
Teste Exato de Fisher				,492	,263
Associação Linear por Linear	,484	1	,487		
N de Casos Válidos	1313				

a. 0 células (,0%) esperavam uma contagem menor que 5. A contagem mínima esperada é 141,44.

b. Computado apenas para uma tabela 2x2

Medidas Simétricas

		Valor	Significância Aproximada
Nominal por Nominal	Fi	,019	,487
	V de Cramer	,019	,487
N de Casos Válidos		1313	

Estimativa de Risco

	Valor	Intervalo de confiança de 95% Inferior	Superior
Razão de Chances para The participant exerts professional activity external to the residency program (No / Yes)	1,091	,854	1,392
Para grupo OLBI Score Classification = Low to Moderate	1,030	,948	1,119
Para grupo OLBI Score Classification = High	,944	,804	1,109
N de Casos Válidos	1313		

```
The participant provides direct health care for COVID19 patients * DASS21 Classification - Depression
(dichotomous)
```

Crosstab

			DASS21 Classification - Depression (dichotomous)		
			Normal	Abnormal (elevated)	Total
The participant provides direct health care for COVID19 patients	No	Contagem	356	167	523
		% em The participant provides direct health care for COVID19 patients	68,1%	31,9%	100,0%
		% do Total	27,1%	12,7%	39,8%
	Yes	Contagem	514	276	790
		% em The participant provides direct health care for COVID19 patients	65,1%	34,9%	100,0%
		% do Total	39,1%	21,0%	60,2%
Total		Contagem	870	443	1313
		% em The participant provides direct health care for COVID19 patients	66,3%	33,7%	100,0%
		% do Total	66,3%	33,7%	100,0%

Testes qui-quadrado

	Valor	gl	Significância Assintótica (Bilateral)	Sig exata (2 lados)	Sig exata (1 lado)
Qui-quadrado de Pearson	1,271[a]	1	,259		
Correção de continuidade[b]	1,141	1	,286		
Razão de verossimilhança	1,276	1	,259		
Teste Exato de Fisher				,283	,143
Associação Linear por Linear	1,271	1	,260		
N de Casos Válidos	1313				

a. 0 células (,0%) esperavam uma contagem menor que 5. A contagem mínima esperada é 176,46.

b. Computado apenas para uma tabela 2x2

Medidas Simétricas

	Valor	Significância Aproximada

Nominal por Nominal	Fi	,031	,259
	V de Cramer	,031	,259
N de Casos Válidos		1313	

Estimativa de Risco

	Valor	Intervalo de confiança de 95% Inferior	Superior
Razão de Chances para The participant provides direct health care for COVID19 patients (No / Yes)	1,145	,905	1,448
Para grupo DASS21 Classification - Depression (dichotomous) = Normal	1,046	,968	1,131
Para grupo DASS21 Classification - Depression (dichotomous) = Abnormal (elevated)	,914	,781	1,070
N de Casos Válidos	1313		

```
The participant provides direct health care for COVID19 patients * DASS21 Classification - Anxiety
(dichotomous)
```

Crosstab

			DASS21 Classification - Anxiety (dichotomous) Normal	Abnormal (elevated)	Total
The participant provides direct health care for COVID19 patients	No	Contagem	346	177	523
		% em The participant provides direct health care for COVID19 patients	66,2%	33,8%	100,0%
		% do Total	26,4%	13,5%	39,8%
	Yes	Contagem	515	275	790
		% em The participant provides direct health care for COVID19 patients	65,2%	34,8%	100,0%
		% do Total	39,2%	20,9%	60,2%
Total		Contagem	861	452	1313
		% em The participant provides direct health care for COVID19 patients	65,6%	34,4%	100,0%
		% do Total	65,6%	34,4%	100,0%

Testes qui-quadrado

	Valor	gl	Significância Assintótica (Bilateral)	Sig exata (2 lados)	Sig exata (1 lado)
Qui-quadrado de Pearson	,130[a]	1	,718		
Correção de continuidade[b]	,091	1	,763		
Razão de verossimilhança	,130	1	,718		
Teste Exato de Fisher				,722	,382
Associação Linear por Linear	,130	1	,718		
N de Casos Válidos	1313				

a. 0 células (,0%) esperavam uma contagem menor que 5. A contagem mínima esperada é 180,04.

b. Computado apenas para uma tabela 2x2

Medidas Simétricas

		Valor	Significância Aproximada
Nominal por Nominal	Fi	,010	,718
	V de Cramer	,010	,718
N de Casos Válidos		1313	

Estimativa de Risco

	Valor	Intervalo de confiança de 95%	
		Inferior	Superior
Razão de Chances para The participant provides direct health care for COVID19 patients (No / Yes)	1,044	,827	1,318
Para grupo DASS21 Classification - Anxiety (dichotomous) = Normal	1,015	,937	1,099
Para grupo DASS21 Classification - Anxiety (dichotomous) = Abnormal (elevated)	,972	,834	1,133
N de Casos Válidos	1313		

The participant provides direct health care for COVID19 patients * DASS21 Classification - Stress (dichotomous)

Crosstab

			DASS21 Classification - Stress (dichotomous)		
			Normal	Abnormal (elevated)	Total
The participant provides direct health care for COVID19 patients	No	Contagem	406	117	523
		% em The participant provides direct health care for COVID19 patients	77,6%	22,4%	100,0%
		% do Total	30,9%	8,9%	39,8%
	Yes	Contagem	606	184	790
		% em The participant provides direct health care for COVID19 patients	76,7%	23,3%	100,0%
		% do Total	46,2%	14,0%	60,2%
Total		Contagem	1012	301	1313
		% em The participant provides direct health care for COVID19 patients	77,1%	22,9%	100,0%
		% do Total	77,1%	22,9%	100,0%

Testes qui-quadrado

	Valor	gl	Significância Assintótica (Bilateral)	Sig exata (2 lados)	Sig exata (1 lado)
Qui-quadrado de Pearson	,151[a]	1	,698		
Correção de continuidade[b]	,103	1	,748		
Razão de verossimilhança	,151	1	,697		
Teste Exato de Fisher				,738	,375
Associação Linear por Linear	,151	1	,698		
N de Casos Válidos	1313				

a. 0 células (,0%) esperavam uma contagem menor que 5. A contagem mínima esperada é 119,90.

b. Computado apenas para uma tabela 2x2

Medidas Simétricas

		Valor	Significância Aproximada
Nominal por Nominal	Fi	,011	,698
	V de Cramer	,011	,698
N de Casos Válidos		1313	

Estimativa de Risco

	Valor	Intervalo de confiança de 95%	
		Inferior	Superior

Razão de Chances para The participant provides direct health care for COVID19 patients (No / Yes)	1,054	,809	1,371
Para grupo DASS21 Classification - Stress (dichotomous) = Normal	1,012	,953	1,075
Para grupo DASS21 Classification - Stress (dichotomous) = Abnormal (elevated)	,960	,783	1,178
N de Casos Válidos	1313		

```
The participant provides direct health care for COVID19 patients * PHQ9 Depression Classification - Risk
estimate for having current major depressive disorder
```

Crosstab

			PHQ9 Depression Classification - Risk estimate for having current major depressive disorder		
			Low	High	Total
The participant provides direct health care for COVID19 patients	No	Contagem	183	340	523
		% em The participant provides direct health care for COVID19 patients	35,0%	65,0%	100,0%
		% do Total	13,9%	25,9%	39,8%
	Yes	Contagem	246	544	790
		% em The participant provides direct health care for COVID19 patients	31,1%	68,9%	100,0%
		% do Total	18,7%	41,4%	60,2%
Total		Contagem	429	884	1313
		% em The participant provides direct health care for COVID19 patients	32,7%	67,3%	100,0%
		% do Total	32,7%	67,3%	100,0%

Testes qui-quadrado

	Valor	gl	Significância Assintótica (Bilateral)	Sig exata (2 lados)	Sig exata (1 lado)
Qui-quadrado de Pearson	2,122[a]	1	,145		
Correção de continuidade[b]	1,950	1	,163		
Razão de verossimilhança	2,114	1	,146		

Teste Exato de Fisher				,150	,081
Associação Linear por Linear	2,120	1	,145		
N de Casos Válidos	1313				

a. 0 células (,0%) esperavam uma contagem menor que 5. A contagem mínima esperada é 170,88.

b. Computado apenas para uma tabela 2x2

Medidas Simétricas

		Valor	Significância Aproximada
Nominal por Nominal	Fi	,040	,145
	V de Cramer	,040	,145
N de Casos Válidos		1313	

Estimativa de Risco

	Valor	Intervalo de confiança de 95%	
		Inferior	Superior
Razão de Chances para The participant provides direct health care for COVID19 patients (No / Yes)	1,190	,941	1,505
Para grupo PHQ9 Depression Classification - Risk estimate for having current major depressive disorder = Low	1,124	,961	1,314
Para grupo PHQ9 Depression Classification - Risk estimate for having current major depressive disorder = High	,944	,873	1,021
N de Casos Válidos	1313		

The participant provides direct health care for COVID19 patients * BRCS Classification

Crosstab

			BRCS Classification		Total
			Low resilience	Moderate to High	
The participant provides direct health care for COVID19 patients	No	Contagem	333	190	523
		% em The participant provides direct health care for COVID19 patients	63,7%	36,3%	100,0%
		% do Total	25,4%	14,5%	39,8%
	Yes	Contagem	480	310	790

	% em The participant provides direct health care for COVID19 patients	60,8%	39,2%	100,0%
	% do Total	36,6%	23,6%	60,2%
Total	Contagem	813	500	1313
	% em The participant provides direct health care for COVID19 patients	61,9%	38,1%	100,0%
	% do Total	61,9%	38,1%	100,0%

Testes qui-quadrado

	Valor	gl	Significância Assintótica (Bilateral)	Sig exata (2 lados)	Sig exata (1 lado)
Qui-quadrado de Pearson	1,131[a]	1	,287		
Correção de continuidade[b]	1,011	1	,315		
Razão de verossimilhança	1,134	1	,287		
Teste Exato de Fisher				,297	,157
Associação Linear por Linear	1,131	1	,288		
N de Casos Válidos	1313				

a. 0 células (,0%) esperavam uma contagem menor que 5. A contagem mínima esperada é 199,16.

b. Computado apenas para uma tabela 2x2

Medidas Simétricas

		Valor	Significância Aproximada
Nominal por Nominal	Fi	,029	,287
	V de Cramer	,029	,287
N de Casos Válidos		1313	

Estimativa de Risco

	Valor	Intervalo de confiança de 95% Inferior	Superior
Razão de Chances para The participant provides direct health care for COVID19 patients (No / Yes)	1,132	,901	1,422
Para grupo BRCS Classification = Low resilience	1,048	,962	1,142
Para grupo BRCS Classification = Moderate to High	,926	,803	1,068
N de Casos Válidos	1313		

```
The participant provides direct health care for COVID19 patients * OLBI Score Classification
```

Crosstab

			OLBI Score Classification		Total
			Low to Moderate	High	
The participant provides direct health care for COVID19 patients	No	Contagem	352	171	523
		% em The participant provides direct health care for COVID19 patients	67,3%	32,7%	100,0%
		% do Total	26,8%	13,0%	39,8%
	Yes	Contagem	523	267	790
		% em The participant provides direct health care for COVID19 patients	66,2%	33,8%	100,0%
		% do Total	39,8%	20,3%	60,2%
Total		Contagem	875	438	1313
		% em The participant provides direct health care for COVID19 patients	66,6%	33,4%	100,0%
		% do Total	66,6%	33,4%	100,0%

Testes qui-quadrado

	Valor	gl	Significância Assintótica (Bilateral)	Sig exata (2 lados)	Sig exata (1 lado)
Qui-quadrado de Pearson	,172[a]	1	,679		
Correção de continuidade[b]	,126	1	,723		
Razão de verossimilhança	,172	1	,678		
Teste Exato de Fisher				,720	,362
Associação Linear por Linear	,172	1	,679		
N de Casos Válidos	1313				

a. 0 células (,0%) esperavam uma contagem menor que 5. A contagem mínima esperada é 174,47.

b. Computado apenas para uma tabela 2x2

Medidas Simétricas

		Valor	Significância Aproximada
Nominal por Nominal	Fi	,011	,679
	V de Cramer	,011	,679

N de Casos Válidos	1313	

Estimativa de Risco

	Valor	Intervalo de confiança de 95% Inferior	Superior
Razão de Chances para The participant provides direct health care for COVID19 patients (No / Yes)	1,051	,831	1,329
Para grupo OLBI Score Classification = Low to Moderate	1,017	,941	1,099
Para grupo OLBI Score Classification = High	,967	,827	1,132
N de Casos Válidos	1313		

```
CROSSTABS
  /TABLES=BRCS_Classif BY DASS21_Classif_D2c DASS21_Classif_A2c DASS21_Classif_S2c PHQ9_Classif
    OLBI_Classif_2c
  /FORMAT=AVALUE TABLES
  /STATISTICS=CHISQ PHI RISK
  /CELLS=COUNT ROW TOTAL
  /COUNT ROUND CELL.
```

Tabulações cruzadas

Observações

Saída criada		20-SEP-2020 11:50:52
Comentários		
Entrada	Dados	C:\Users\User\Documents\Pesquisa\Fellow\FellowGenData_V1.sav
	Conjunto de dados ativo	ConjuntodeDados1
	Filtro	<none>
	Ponderação	<none>
	Arquivo Dividido	<none>
	N de linhas em arquivo de dados de trabalho	1313
Tratamento de valor omisso	Definição de omisso	Os valores omissos definidos pelo usuário são tratados como omissos.

	Casos utilizados	As estatísticas de cada tabela são baseadas em todos os casos com dados válidos na(s) amplitude(s) especificada(s) para todas as variáveis de cada tabela.
Sintaxe		CROSSTABS /TABLES=BRCS_Classif BY DASS21_Classif_D2c DASS21_Classif_A2c DASS21_Classif_S2c PHQ9_Classif OLBI_Classif_2c /FORMAT=AVALUE TABLES /STATISTICS=CHISQ PHI RISK /CELLS=COUNT ROW TOTAL /COUNT ROUND CELL.
Recursos	Tempo do processador	00:00:00,02
	Tempo decorrido	00:00:00,02
	Dimensões solicitadas	2
	Células disponíveis	524245

BRCS Classification * DASS21 Classification - Depression (dichotomous)

Crosstab

			DASS21 Classification - Depression (dichotomous)		
			Normal	Abnormal (elevated)	Total
BRCS Classification	Low resilience	Contagem	460	353	813
		% em BRCS Classification	56,6%	43,4%	100,0%
		% do Total	35,0%	26,9%	61,9%
	Moderate to High	Contagem	410	90	500
		% em BRCS Classification	82,0%	18,0%	100,0%
		% do Total	31,2%	6,9%	38,1%
Total		Contagem	870	443	1313
		% em BRCS Classification	66,3%	33,7%	100,0%
		% do Total	66,3%	33,7%	100,0%

Testes qui-quadrado

	Valor	gl	Significância Assintótica (Bilateral)	Sig exata (2 lados)	Sig exata (1 lado)

Qui-quadrado de Pearson	89,482[a]	1	,000		
Correção de continuidade[b]	88,348	1	,000		
Razão de verossimilhança	94,455	1	,000		
Teste Exato de Fisher				,000	,000
Associação Linear por Linear	89,414	1	,000		
N de Casos Válidos	1313				

a. 0 células (,0%) esperavam uma contagem menor que 5. A contagem mínima esperada é 168,70.

b. Computado apenas para uma tabela 2x2

Medidas Simétricas

		Valor	Significância Aproximada
Nominal por Nominal	Fi	-,261	,000
	V de Cramer	,261	,000
N de Casos Válidos		1313	

Estimativa de Risco

	Valor	Intervalo de confiança de 95% Inferior	Superior
Razão de Chances para BRCS Classification (Low resilience / Moderate to High)	,286	,219	,374
Para grupo DASS21 Classification - Depression (dichotomous) = Normal	,690	,642	,742
Para grupo DASS21 Classification - Depression (dichotomous) = Abnormal (elevated)	2,412	1,969	2,955
N de Casos Válidos	1313		

BRCS Classification * DASS21 Classification - Anxiety (dichotomous)

Crosstab

			DASS21 Classification - Anxiety (dichotomous)		
			Normal	Abnormal (elevated)	Total
BRCS Classification	Low resilience	Contagem	478	335	813
		% em BRCS Classification	58,8%	41,2%	100,0%
		% do Total	36,4%	25,5%	61,9%

	Moderate to High	Contagem	383	117	500
		% em BRCS Classification	76,6%	23,4%	100,0%
		% do Total	29,2%	8,9%	38,1%
Total		Contagem	861	452	1313
		% em BRCS Classification	65,6%	34,4%	100,0%
		% do Total	65,6%	34,4%	100,0%

Testes qui-quadrado

	Valor	gl	Significância Assintótica (Bilateral)	Sig exata (2 lados)	Sig exata (1 lado)
Qui-quadrado de Pearson	43,480[a]	1	,000		
Correção de continuidade[b]	42,695	1	,000		
Razão de verossimilhança	44,818	1	,000		
Teste Exato de Fisher				,000	,000
Associação Linear por Linear	43,447	1	,000		
N de Casos Válidos	1313				

a. 0 células (,0%) esperavam uma contagem menor que 5. A contagem mínima esperada é 172,12.

b. Computado apenas para uma tabela 2x2

Medidas Simétricas

		Valor	Significância Aproximada
Nominal por Nominal	Fi	-,182	,000
	V de Cramer	,182	,000
N de Casos Válidos		1313	

Estimativa de Risco

	Valor	Intervalo de confiança de 95% Inferior	Superior
Razão de Chances para BRCS Classification (Low resilience / Moderate to High)	,436	,340	,560
Para grupo DASS21 Classification - Anxiety (dichotomous) = Normal	,768	,712	,828
Para grupo DASS21 Classification - Anxiety (dichotomous) = Abnormal (elevated)	1,761	1,473	2,105
N de Casos Válidos	1313		

BRCS Classification * DASS21 Classification - Stress (dichotomous)

Crosstab

			DASS21 Classification - Stress (dichotomous) Normal	Abnormal (elevated)	Total
BRCS Classification	Low resilience	Contagem	577	236	813
		% em BRCS Classification	71,0%	29,0%	100,0%
		% do Total	43,9%	18,0%	61,9%
	Moderate to High	Contagem	435	65	500
		% em BRCS Classification	87,0%	13,0%	100,0%
		% do Total	33,1%	5,0%	38,1%
Total		Contagem	1012	301	1313
		% em BRCS Classification	77,1%	22,9%	100,0%
		% do Total	77,1%	22,9%	100,0%

Testes qui-quadrado

	Valor	gl	Significância Assintótica (Bilateral)	Sig exata (2 lados)	Sig exata (1 lado)
Qui-quadrado de Pearson	45,015[a]	1	,000		
Correção de continuidade[b]	44,112	1	,000		
Razão de verossimilhança	47,846	1	,000		
Teste Exato de Fisher				,000	,000
Associação Linear por Linear	44,980	1	,000		
N de Casos Válidos	1313				

a. 0 células (,0%) esperavam uma contagem menor que 5. A contagem mínima esperada é 114,62.

b. Computado apenas para uma tabela 2x2

Medidas Simétricas

		Valor	Significância Aproximada
Nominal por Nominal	Fi	-,185	,000
	V de Cramer	,185	,000
N de Casos Válidos		1313	

Estimativa de Risco

	Valor	Intervalo de confiança de 95% Inferior	Superior

Razão de Chances para BRCS Classification (Low resilience / Moderate to High)	,365	,270	,494
Para grupo DASS21 Classification - Stress (dichotomous) = Normal	,816	,772	,862
Para grupo DASS21 Classification - Stress (dichotomous) = Abnormal (elevated)	2,233	1,737	2,870
N de Casos Válidos	1313		

```
BRCS Classification * PHQ9 Depression Classification - Risk estimate for having current major depressive
disorder
```

Crosstab

			PHQ9 Depression Classification - Risk estimate for having current major depressive disorder		
			Low	High	Total
BRCS Classification	Low resilience	Contagem	180	633	813
		% em BRCS Classification	22,1%	77,9%	100,0%
		% do Total	13,7%	48,2%	61,9%
	Moderate to High	Contagem	249	251	500
		% em BRCS Classification	49,8%	50,2%	100,0%
		% do Total	19,0%	19,1%	38,1%
Total		Contagem	429	884	1313
		% em BRCS Classification	32,7%	67,3%	100,0%
		% do Total	32,7%	67,3%	100,0%

Testes qui-quadrado

	Valor	gl	Significância Assintótica (Bilateral)	Sig exata (2 lados)	Sig exata (1 lado)
Qui-quadrado de Pearson	107,675[a]	1	,000		
Correção de continuidade[b]	106,421	1	,000		
Razão de verossimilhança	106,445	1	,000		
Teste Exato de Fisher				,000	,000
Associação Linear por Linear	107,593	1	,000		
N de Casos Válidos	1313				

a. 0 células (,0%) esperavam uma contagem menor que 5. A contagem mínima esperada é 163,37.

b. Computado apenas para uma tabela 2x2

Medidas Simétricas

		Valor	Significância Aproximada
Nominal por Nominal	Fi	-,286	,000
	V de Cramer	,286	,000
N de Casos Válidos		1313	

Estimativa de Risco

	Valor	Intervalo de confiança de 95% Inferior	Superior
Razão de Chances para BRCS Classification (Low resilience / Moderate to High)	,287	,225	,365
Para grupo PHQ9 Depression Classification - Risk estimate for having current major depressive disorder = Low	,445	,380	,520
Para grupo PHQ9 Depression Classification - Risk estimate for having current major depressive disorder = High	1,551	1,411	1,705
N de Casos Válidos	1313		

```
BRCS Classification * OLBI Score Classification
```

Crosstab

			OLBI Score Classification		
			Low to Moderate	High	Total
BRCS Classification	Low resilience	Contagem	468	345	813
		% em BRCS Classification	57,6%	42,4%	100,0%
		% do Total	35,6%	26,3%	61,9%
	Moderate to High	Contagem	407	93	500
		% em BRCS Classification	81,4%	18,6%	100,0%
		% do Total	31,0%	7,1%	38,1%
Total		Contagem	875	438	1313
		% em BRCS Classification	66,6%	33,4%	100,0%
		% do Total	66,6%	33,4%	100,0%

Testes qui-quadrado

	Valor	gl	Significância Assintótica (Bilateral)	Sig exata (2 lados)	Sig exata (1 lado)
Qui-quadrado de Pearson	79,120[a]	1	,000		
Correção de continuidade[b]	78,052	1	,000		
Razão de verossimilhança	83,200	1	,000		
Teste Exato de Fisher				,000	,000
Associação Linear por Linear	79,060	1	,000		
N de Casos Válidos	1313				

a. 0 células (,0%) esperavam uma contagem menor que 5. A contagem mínima esperada é 166,79.

b. Computado apenas para uma tabela 2x2

Medidas Simétricas

		Valor	Significância Aproximada
Nominal por Nominal	Fi	-,245	,000
	V de Cramer	,245	,000
N de Casos Válidos		1313	

Estimativa de Risco

	Valor	Intervalo de confiança de 95%	
		Inferior	Superior
Razão de Chances para BRCS Classification (Low resilience / Moderate to High)	,310	,238	,404
Para grupo OLBI Score Classification = Low to Moderate	,707	,658	,760
Para grupo OLBI Score Classification = High	2,281	1,868	2,787
N de Casos Válidos	1313		

```
CROSSTABS
  /TABLES=RsPrgType BY Sex Race ComorbAny HRiskFactor DASS21_Classif_D2c DASS21_Classif_A2c
    DASS21_Classif_S2c PHQ9_Classif BRCS_Classif OLBI_Classif_2c Autonomy_Classif PedagStr_Classif
    PPEAvail_Classif ExternWork CovidCare Wkload_5P
  /FORMAT=AVALUE TABLES
  /STATISTICS=CHISQ PHI RISK
  /CELLS=COUNT ROW TOTAL
  /COUNT ROUND CELL.
```

Tabulações cruzadas

Observações

Saída criada		20-SEP-2020 12:03:39
Comentários		
Entrada	Dados	C:\Users\User\Documents\Pesquisa\Fellow\FellowGenData_V1.sav
	Conjunto de dados ativo	ConjuntodeDados1
	Filtro	<none>
	Ponderação	<none>
	Arquivo Dividido	<none>
	N de linhas em arquivo de dados de trabalho	1313
Tratamento de valor omisso	Definição de omisso	Os valores omissos definidos pelo usuário são tratados como omissos.
	Casos utilizados	As estatísticas de cada tabela são baseadas em todos os casos com dados válidos na(s) amplitude(s) especificada(s) para todas as variáveis de cada tabela.
Sintaxe		CROSSTABS<br>/TABLES=RsPrgType BY Sex Race ComorbAny HRiskFactor DASS21_Classif_D2c DASS21_Classif_A2c<br>DASS21_Classif_S2c PHQ9_Classif BRCS_Classif OLBI_Classif_2c Autonomy_Classif PedagStr_Classif<br>PPEAvail_Classif ExternWork CovidCare Wkload_5P<br>/FORMAT=AVALUE TABLES<br>/STATISTICS=CHISQ PHI RISK<br>/CELLS=COUNT ROW TOTAL<br>/COUNT ROUND CELL.
Recursos	Tempo do processador	00:00:00,05
	Tempo decorrido	00:00:00,05
	Dimensões solicitadas	2
	Células disponíveis	524245

Resumo de processamento de casos

	Casos					
	Válidos		Omissos		Total	
	N	Porcentagem	N	Porcentagem	N	Porcentagem
Type of residency program * Sex	1310	99,8%	3	0,2%	1313	100,0%

Type of residency program * Race	1313	100,0%	0	0,0%	1313	100,0%
Type of residency program * Any comorbidity (regardless COVID risk)	1305	99,4%	8	0,6%	1313	100,0%
Type of residency program * Risk factor(s) for severe COVID19	1305	99,4%	8	0,6%	1313	100,0%
Type of residency program * DASS21 Classification - Depression (dichotomous)	1313	100,0%	0	0,0%	1313	100,0%
Type of residency program * DASS21 Classification - Anxiety (dichotomous)	1313	100,0%	0	0,0%	1313	100,0%
Type of residency program * DASS21 Classification - Stress (dichotomous)	1313	100,0%	0	0,0%	1313	100,0%
Type of residency program * PHQ9 Depression Classification - Risk estimate for having current major depressive disorder	1313	100,0%	0	0,0%	1313	100,0%
Type of residency program * BRCS Classification	1313	100,0%	0	0,0%	1313	100,0%
Type of residency program * OLBI Score Classification	1313	100,0%	0	0,0%	1313	100,0%
Type of residency program * Classification of the percieved autonomy to self-conduct in the residency program	1313	100,0%	0	0,0%	1313	100,0%
Type of residency program * Percieved adequacy of the pedagogic structure and availability of resouces in the residency program	1313	100,0%	0	0,0%	1313	100,0%
Type of residency program * Perceived adequacy of the availability of personal protective equipment, when providing care for patients in the residency program	1313	100,0%	0	0,0%	1313	100,0%
Type of residency program * The participant exerts professional activity external to the residency program	1313	100,0%	0	0,0%	1313	100,0%

Type of residency program * The participant provides direct health care for COVID19 patients	1313	100,0%	0	0,0%	1313	100,0%
Type of residency program * Cumulative weekly workload (five categories)	1313	100,0%	0	0,0%	1313	100,0%

```
Type of residency program * Sex
```

Crosstab

			Sex		Total
			Male	Female	
Type of residency program	Non-Medical (Other HCP)	Contagem	87	549	636
		% em Type of residency program	13,7%	86,3%	100,0%
		% do Total	6,6%	41,9%	48,5%
	Medical	Contagem	198	476	674
		% em Type of residency program	29,4%	70,6%	100,0%
		% do Total	15,1%	36,3%	51,5%
Total		Contagem	285	1025	1310
		% em Type of residency program	21,8%	78,2%	100,0%
		% do Total	21,8%	78,2%	100,0%

Testes qui-quadrado

	Valor	gl	Significância Assintótica (Bilateral)	Sig exata (2 lados)	Sig exata (1 lado)
Qui-quadrado de Pearson	47,368[a]	1	,000		
Correção de continuidade[b]	46,450	1	,000		
Razão de verossimilhança	48,498	1	,000		
Teste Exato de Fisher				,000	,000
Associação Linear por Linear	47,332	1	,000		
N de Casos Válidos	1310				

a. 0 células (,0%) esperavam uma contagem menor que 5. A contagem mínima esperada é 138,37.

b. Computado apenas para uma tabela 2x2

Medidas Simétricas

		Valor	Significância Aproximada
Nominal por Nominal	Fi	-,190	,000

	V de Cramer	,190	,000
N de Casos Válidos		1310	

Estimativa de Risco

	Valor	Intervalo de confiança de 95%	
		Inferior	Superior
Razão de Chances para Type of residency program (Non-Medical (Other HCP) / Medical)	,381	,288	,504
Para grupo Sex = Male	,466	,371	,585
Para grupo Sex = Female	1,222	1,154	1,295
N de Casos Válidos	1310		

```
Type of residency program * Race
```

Crosstab

			Race				Total
			White	Pardo	Black	Other	
Type of residency program	Non-Medical (Other HCP)	Contagem	338	237	54	10	639
		% em Type of residency program	52,9%	37,1%	8,5%	1,6%	100,0%
		% do Total	25,7%	18,1%	4,1%	0,8%	48,7%
	Medical	Contagem	440	196	27	11	674
		% em Type of residency program	65,3%	29,1%	4,0%	1,6%	100,0%
		% do Total	33,5%	14,9%	2,1%	0,8%	51,3%
Total		Contagem	778	433	81	21	1313
		% em Type of residency program	59,3%	33,0%	6,2%	1,6%	100,0%
		% do Total	59,3%	33,0%	6,2%	1,6%	100,0%

Testes qui-quadrado

	Valor	gl	Significância Assintótica (Bilateral)
Qui-quadrado de Pearson	25,388[a]	3	,000
Razão de verossimilhança	25,588	3	,000
Associação Linear por Linear	19,470	1	,000
N de Casos Válidos	1313		

a. 0 células (,0%) esperavam uma contagem menor que 5. A contagem mínima esperada é 10,22.

Medidas Simétricas

		Valor	Significância Aproximada
Nominal por Nominal	Fi	,139	,000
	V de Cramer	,139	,000
N de Casos Válidos		1313	

Estimativa de Risco

	Valor
Razão de Chances para Type of residency program (Non-Medical (Other HCP) / Medical)	[a]

a. Não é possível calcular as estatísticas de Estimativa de Risco. Elas são computadas apenas para uma tabela 2*2 sem células vazias.

```
Type of residency program * Any comorbidity (regardless COVID risk)
```

Crosstab

			Any comorbidity (regardless COVID risk)		
			No	Yes	Total
Type of residency program	Non-Medical (Other HCP)	Contagem	530	104	634
		% em Type of residency program	83,6%	16,4%	100,0%
		% do Total	40,6%	8,0%	48,6%
	Medical	Contagem	541	130	671
		% em Type of residency program	80,6%	19,4%	100,0%
		% do Total	41,5%	10,0%	51,4%
Total		Contagem	1071	234	1305
		% em Type of residency program	82,1%	17,9%	100,0%
		% do Total	82,1%	17,9%	100,0%

Testes qui-quadrado

	Valor	gl	Significância Assintótica (Bilateral)	Sig exata (2 lados)	Sig exata (1 lado)
Qui-quadrado de Pearson	1,954[a]	1	,162		

Correção de continuidade[b]	1,758	1	,185		
Razão de verossimilhança	1,959	1	,162		
Teste Exato de Fisher				,171	,092
Associação Linear por Linear	1,953	1	,162		
N de Casos Válidos	1305				

a. 0 células (,0%) esperavam uma contagem menor que 5. A contagem mínima esperada é 113,68.

b. Computado apenas para uma tabela 2x2

Medidas Simétricas

		Valor	Significância Aproximada
Nominal por Nominal	Fi	,039	,162
	V de Cramer	,039	,162
N de Casos Válidos		1305	

Estimativa de Risco

	Valor	Intervalo de confiança de 95%	
		Inferior	Superior
Razão de Chances para Type of residency program (Non-Medical (Other HCP) / Medical)	1,225	,922	1,627
Para grupo Any comorbidity (regardless COVID risk) = No	1,037	,986	1,091
Para grupo Any comorbidity (regardless COVID risk) = Yes	,847	,670	1,070
N de Casos Válidos	1305		

```
Type of residency program * Risk factor(s) for severe COVID19
```

Crosstab

			Risk factor(s) for severe COVID19		
			No	Yes	Total
Type of residency program	Non-Medical (Other HCP)	Contagem	539	95	634
		% em Type of residency program	85,0%	15,0%	100,0%
		% do Total	41,3%	7,3%	48,6%
	Medical	Contagem	548	123	671
		% em Type of residency program	81,7%	18,3%	100,0%
		% do Total	42,0%	9,4%	51,4%
Total		Contagem	1087	218	1305
		% em Type of residency program	83,3%	16,7%	100,0%

% do Total	83,3%	16,7%	100,0%

Testes qui-quadrado

	Valor	gl	Significância Assintótica (Bilateral)	Sig exata (2 lados)	Sig exata (1 lado)
Qui-quadrado de Pearson	2,624[a]	1	,105		
Correção de continuidade[b]	2,389	1	,122		
Razão de verossimilhança	2,632	1	,105		
Teste Exato de Fisher				,119	,061
Associação Linear por Linear	2,622	1	,105		
N de Casos Válidos	1305				

a. 0 células (,0%) esperavam uma contagem menor que 5. A contagem mínima esperada é 105,91.

b. Computado apenas para uma tabela 2x2

Medidas Simétricas

		Valor	Significância Aproximada
Nominal por Nominal	Fi	,045	,105
	V de Cramer	,045	,105
N de Casos Válidos		1305	

Estimativa de Risco

	Valor	Intervalo de confiança de 95% Inferior	Superior
Razão de Chances para Type of residency program (Non-Medical (Other HCP) / Medical)	1,273	,950	1,707
Para grupo Risk factor(s) for severe COVID19 = No	1,041	,992	1,093
Para grupo Risk factor(s) for severe COVID19 = Yes	,817	,640	1,044
N de Casos Válidos	1305		

Type of residency program * DASS21 Classification - Depression (dichotomous)

Crosstab

	DASS21 Classification - Depression (dichotomous)	Total

			Normal	Abnormal (elevated)	
Type of residency program	Non-Medical (Other HCP)	Contagem	427	212	639
		% em Type of residency program	66,8%	33,2%	100,0%
		% do Total	32,5%	16,1%	48,7%
	Medical	Contagem	443	231	674
		% em Type of residency program	65,7%	34,3%	100,0%
		% do Total	33,7%	17,6%	51,3%
Total		Contagem	870	443	1313
		% em Type of residency program	66,3%	33,7%	100,0%
		% do Total	66,3%	33,7%	100,0%

Testes qui-quadrado

	Valor	gl	Significância Assintótica (Bilateral)	Sig exata (2 lados)	Sig exata (1 lado)
Qui-quadrado de Pearson	,176[a]	1	,675		
Correção de continuidade[b]	,131	1	,718		
Razão de verossimilhança	,176	1	,675		
Teste Exato de Fisher				,683	,359
Associação Linear por Linear	,176	1	,675		
N de Casos Válidos	1313				

a. 0 células (,0%) esperavam uma contagem menor que 5. A contagem mínima esperada é 215,60.

b. Computado apenas para uma tabela 2x2

Medidas Simétricas

		Valor	Significância Aproximada
Nominal por Nominal	Fi	,012	,675
	V de Cramer	,012	,675
N de Casos Válidos		1313	

Estimativa de Risco

	Valor	Intervalo de confiança de 95% Inferior	Superior
Razão de Chances para Type of residency program (Non-Medical (Other HCP) / Medical)	1,050	,835	1,320
Para grupo DASS21 Classification - Depression (dichotomous) = Normal	1,017	,941	1,098
Para grupo DASS21 Classification - Depression (dichotomous) = Abnormal (elevated)	,968	,832	1,127

N de Casos Válidos	1313		

Type of residency program * DASS21 Classification - Anxiety (dichotomous)

Crosstab

			DASS21 Classification - Anxiety (dichotomous)		
			Normal	Abnormal (elevated)	Total
Type of residency program	Non-Medical (Other HCP)	Contagem	398	241	639
		% em Type of residency program	62,3%	37,7%	100,0%
		% do Total	30,3%	18,4%	48,7%
	Medical	Contagem	463	211	674
		% em Type of residency program	68,7%	31,3%	100,0%
		% do Total	35,3%	16,1%	51,3%
Total		Contagem	861	452	1313
		% em Type of residency program	65,6%	34,4%	100,0%
		% do Total	65,6%	34,4%	100,0%

Testes qui-quadrado

	Valor	gl	Significância Assintótica (Bilateral)	Sig exata (2 lados)	Sig exata (1 lado)
Qui-quadrado de Pearson	5,969[a]	1	,015		
Correção de continuidade[b]	5,689	1	,017		
Razão de verossimilhança	5,971	1	,015		
Teste Exato de Fisher				,015	,009
Associação Linear por Linear	5,965	1	,015		
N de Casos Válidos	1313				

a. 0 células (,0%) esperavam uma contagem menor que 5. A contagem mínima esperada é 219,98.

b. Computado apenas para uma tabela 2x2

Medidas Simétricas

		Valor	Significância Aproximada
Nominal por Nominal	Fi	-,067	,015
	V de Cramer	,067	,015
N de Casos Válidos		1313	

Estimativa de Risco

	Valor	Intervalo de confiança de 95% Inferior	Superior
Razão de Chances para Type of residency program (Non-Medical (Other HCP) / Medical)	,753	,599	,946
Para grupo DASS21 Classification - Anxiety (dichotomous) = Normal	,907	,838	,981
Para grupo DASS21 Classification - Anxiety (dichotomous) = Abnormal (elevated)	1,205	1,037	1,399
N de Casos Válidos	1313		

```
Type of residency program * DASS21 Classification - Stress (dichotomous)
```

Crosstab

			DASS21 Classification - Stress (dichotomous)		
			Normal	Abnormal (elevated)	Total
Type of residency program	Non-Medical (Other HCP)	Contagem	482	157	639
		% em Type of residency program	75,4%	24,6%	100,0%
		% do Total	36,7%	12,0%	48,7%
	Medical	Contagem	530	144	674
		% em Type of residency program	78,6%	21,4%	100,0%
		% do Total	40,4%	11,0%	51,3%
Total		Contagem	1012	301	1313
		% em Type of residency program	77,1%	22,9%	100,0%
		% do Total	77,1%	22,9%	100,0%

Testes qui-quadrado

	Valor	gl	Significância Assintótica (Bilateral)	Sig exata (2 lados)	Sig exata (1 lado)
Qui-quadrado de Pearson	1,907[a]	1	,167		
Correção de continuidade[b]	1,729	1	,188		
Razão de verossimilhança	1,906	1	,167		
Teste Exato de Fisher				,169	,094
Associação Linear por Linear	1,905	1	,168		
N de Casos Válidos	1313				

a. 0 células (,0%) esperavam uma contagem menor que 5. A contagem mínima esperada é 146,49.

b. Computado apenas para uma tabela 2x2

Medidas Simétricas

		Valor	Significância Aproximada
Nominal por Nominal	Fi	-,038	,167
	V de Cramer	,038	,167
N de Casos Válidos		1313	

Estimativa de Risco

	Valor	Intervalo de confiança de 95%	
		Inferior	Superior
Razão de Chances para Type of residency program (Non-Medical (Other HCP) / Medical)	,834	,645	1,079
Para grupo DASS21 Classification - Stress (dichotomous) = Normal	,959	,904	1,018
Para grupo DASS21 Classification - Stress (dichotomous) = Abnormal (elevated)	1,150	,943	1,403
N de Casos Válidos	1313		

```
Type of residency program * PHQ9 Depression Classification - Risk estimate for having current major
depressive disorder
```

Crosstab

			PHQ9 Depression Classification - Risk estimate for having current major depressive disorder		Total
			Low	High	
Type of residency program	Non-Medical (Other HCP)	Contagem	196	443	639
		% em Type of residency program	30,7%	69,3%	100,0%
		% do Total	14,9%	33,7%	48,7%
	Medical	Contagem	233	441	674
		% em Type of residency program	34,6%	65,4%	100,0%
		% do Total	17,7%	33,6%	51,3%
Total		Contagem	429	884	1313
		% em Type of residency program	32,7%	67,3%	100,0%
		% do Total	32,7%	67,3%	100,0%

Testes qui-quadrado

	Valor	gl	Significância Assintótica (Bilateral)	Sig exata (2 lados)	Sig exata (1 lado)
Qui-quadrado de Pearson	2,264[a]	1	,132		
Correção de continuidade[b]	2,091	1	,148		
Razão de verossimilhança	2,267	1	,132		
Teste Exato de Fisher				,141	,074
Associação Linear por Linear	2,263	1	,133		
N de Casos Válidos	1313				

a. 0 células (,0%) esperavam uma contagem menor que 5. A contagem mínima esperada é 208,78.

b. Computado apenas para uma tabela 2x2

Medidas Simétricas

		Valor	Significância Aproximada
Nominal por Nominal	Fi	-,042	,132
	V de Cramer	,042	,132
N de Casos Válidos		1313	

Estimativa de Risco

	Valor	Intervalo de confiança de 95%	
		Inferior	Superior
Razão de Chances para Type of residency program (Non-Medical (Other HCP) / Medical)	,837	,665	1,055
Para grupo PHQ9 Depression Classification - Risk estimate for having current major depressive disorder = Low	,887	,759	1,037
Para grupo PHQ9 Depression Classification - Risk estimate for having current major depressive disorder = High	1,060	,983	1,142
N de Casos Válidos	1313		

```
Type of residency program * BRCS Classification
```

Crosstab

			BRCS Classification		
			Low resilience	Moderate to High	Total
Type of residency program	Non-Medical (Other HCP)	Contagem	414	225	639

		% em Type of residency program	64,8%	35,2%	100,0%
		% do Total	31,5%	17,1%	48,7%
	Medical	Contagem	399	275	674
		% em Type of residency program	59,2%	40,8%	100,0%
		% do Total	30,4%	20,9%	51,3%
Total		Contagem	813	500	1313
		% em Type of residency program	61,9%	38,1%	100,0%
		% do Total	61,9%	38,1%	100,0%

Testes qui-quadrado

	Valor	gl	Significância Assintótica (Bilateral)	Sig exata (2 lados)	Sig exata (1 lado)
Qui-quadrado de Pearson	4,347[a]	1	,037		
Correção de continuidade[b]	4,113	1	,043		
Razão de verossimilhança	4,352	1	,037		
Teste Exato de Fisher				,041	,021
Associação Linear por Linear	4,344	1	,037		
N de Casos Válidos	1313				

a. 0 células (,0%) esperavam uma contagem menor que 5. A contagem mínima esperada é 243,34.

b. Computado apenas para uma tabela 2x2

Medidas Simétricas

		Valor	Significância Aproximada
Nominal por Nominal	Fi	,058	,037
	V de Cramer	,058	,037
N de Casos Válidos		1313	

Estimativa de Risco

	Valor	Intervalo de confiança de 95% Inferior	Superior
Razão de Chances para Type of residency program (Non-Medical (Other HCP) / Medical)	1,268	1,014	1,586
Para grupo BRCS Classification = Low resilience	1,094	1,005	1,191
Para grupo BRCS Classification = Moderate to High	,863	,751	,992
N de Casos Válidos	1313		

Type of residency program * OLBI Score Classification

Crosstab

			OLBI Score Classification		
			Low to Moderate	High	Total
Type of residency program	Non-Medical (Other HCP)	Contagem	437	202	639
		% em Type of residency program	68,4%	31,6%	100,0%
		% do Total	33,3%	15,4%	48,7%
	Medical	Contagem	438	236	674
		% em Type of residency program	65,0%	35,0%	100,0%
		% do Total	33,4%	18,0%	51,3%
Total		Contagem	875	438	1313
		% em Type of residency program	66,6%	33,4%	100,0%
		% do Total	66,6%	33,4%	100,0%

Testes qui-quadrado

	Valor	gl	Significância Assintótica (Bilateral)	Sig exata (2 lados)	Sig exata (1 lado)
Qui-quadrado de Pearson	1,709[a]	1	,191		
Correção de continuidade[b]	1,559	1	,212		
Razão de verossimilhança	1,710	1	,191		
Teste Exato de Fisher				,198	,106
Associação Linear por Linear	1,707	1	,191		
N de Casos Válidos	1313				

a. 0 células (,0%) esperavam uma contagem menor que 5. A contagem mínima esperada é 213,16.

b. Computado apenas para uma tabela 2x2

Medidas Simétricas

		Valor	Significância Aproximada
Nominal por Nominal	Fi	,036	,191
	V de Cramer	,036	,191
N de Casos Válidos		1313	

Estimativa de Risco

Valor	Intervalo de confiança de 95%

		Inferior	Superior
Razão de Chances para Type of residency program (Non-Medical (Other HCP) / Medical)	1,166	,926	1,467
Para grupo OLBI Score Classification = Low to Moderate	1,052	,975	1,136
Para grupo OLBI Score Classification = High	,903	,774	1,053
N de Casos Válidos	1313		

Type of residency program * Classification of the percieved autonomy to self-conduct in the residency program

Crosstab

			Classification of the percieved autonomy to self-conduct in the residency program		
			Low autonomy	Moderate to high autonomy	Total
Type of residency program	Non-Medical (Other HCP)	Contagem	104	535	639
		% em Type of residency program	16,3%	83,7%	100,0%
		% do Total	7,9%	40,7%	48,7%
	Medical	Contagem	120	554	674
		% em Type of residency program	17,8%	82,2%	100,0%
		% do Total	9,1%	42,2%	51,3%
Total		Contagem	224	1089	1313
		% em Type of residency program	17,1%	82,9%	100,0%
		% do Total	17,1%	82,9%	100,0%

Testes qui-quadrado

	Valor	gl	Significância Assintótica (Bilateral)	Sig exata (2 lados)	Sig exata (1 lado)
Qui-quadrado de Pearson	,542[a]	1	,462		
Correção de continuidade[b]	,439	1	,508		
Razão de verossimilhança	,542	1	,461		
Teste Exato de Fisher				,464	,254
Associação Linear por Linear	,541	1	,462		
N de Casos Válidos	1313				

a. 0 células (,0%) esperavam uma contagem menor que 5. A contagem mínima esperada é 109,01.

b. Computado apenas para uma tabela 2x2

Medidas Simétricas

		Valor	Significância Aproximada
Nominal por Nominal	Fi	-,020	,462
	V de Cramer	,020	,462
N de Casos Válidos		1313	

Estimativa de Risco

	Valor	Intervalo de confiança de 95% Inferior	Superior
Razão de Chances para Type of residency program (Non-Medical (Other HCP) / Medical)	,897	,673	1,197
Para grupo Classification of the percieved autonomy to self-conduct in the residency program = Low autonomy	,914	,720	1,161
Para grupo Classification of the percieved autonomy to self-conduct in the residency program = Moderate to high autonomy	1,019	,970	1,070
N de Casos Válidos	1313		

```
Type of residency program * Percieved adequacy of the pedagogic structure and availability of resouces
in the residency program
```

Crosstab

			Percieved adequacy of the pedagogic structure and availability of resouces in the residency program		
			Poor adequacy	Moderate to good adequacy	Total
Type of residency program	Non-Medical (Other HCP)	Contagem	327	312	639
		% em Type of residency program	51,2%	48,8%	100,0%
		% do Total	24,9%	23,8%	48,7%
	Medical	Contagem	231	443	674
		% em Type of residency program	34,3%	65,7%	100,0%
		% do Total	17,6%	33,7%	51,3%
Total		Contagem	558	755	1313
		% em Type of residency program	42,5%	57,5%	100,0%
		% do Total	42,5%	57,5%	100,0%

Testes qui-quadrado

	Valor	gl	Significância Assintótica (Bilateral)	Sig exata (2 lados)	Sig exata (1 lado)
Qui-quadrado de Pearson	38,340[a]	1	,000		
Correção de continuidade[b]	37,652	1	,000		
Razão de verossimilhança	38,511	1	,000		
Teste Exato de Fisher				,000	,000
Associação Linear por Linear	38,311	1	,000		
N de Casos Válidos	1313				

a. 0 células (,0%) esperavam uma contagem menor que 5. A contagem mínima esperada é 271,56.

b. Computado apenas para uma tabela 2x2

Medidas Simétricas

		Valor	Significância Aproximada
Nominal por Nominal	Fi	,171	,000
	V de Cramer	,171	,000
N de Casos Válidos		1313	

Estimativa de Risco

	Valor	Intervalo de confiança de 95%	
		Inferior	Superior
Razão de Chances para Type of residency program (Non-Medical (Other HCP) / Medical)	2,010	1,610	2,510
Para grupo Percieved adequacy of the pedagogic structure and availability of resouces in the residency program = Poor adequacy	1,493	1,312	1,699
Para grupo Percieved adequacy of the pedagogic structure and availability of resouces in the residency program = Moderate to good adequacy	,743	,675	,818
N de Casos Válidos	1313		

Type of residency program * Perceived adequacy of the availability of personal protective equipment, when providing care for patients in the residency program

Crosstab

			Perceived adequacy of the availability of personal protective equipment, when providing care for patients in the residency program		
			Poor adequacy	Good adequacy	Total
Type of residency program	Non-Medical (Other HCP)	Contagem	124	515	639
		% em Type of residency program	19,4%	80,6%	100,0%
		% do Total	9,4%	39,2%	48,7%
	Medical	Contagem	157	517	674
		% em Type of residency program	23,3%	76,7%	100,0%
		% do Total	12,0%	39,4%	51,3%
Total		Contagem	281	1032	1313
		% em Type of residency program	21,4%	78,6%	100,0%
		% do Total	21,4%	78,6%	100,0%

Testes qui-quadrado

	Valor	gl	Significância Assintótica (Bilateral)	Sig exata (2 lados)	Sig exata (1 lado)
Qui-quadrado de Pearson	2,948[a]	1	,086		
Correção de continuidade[b]	2,722	1	,099		
Razão de verossimilhança	2,955	1	,086		
Teste Exato de Fisher				,092	,049
Associação Linear por Linear	2,946	1	,086		
N de Casos Válidos	1313				

a. 0 células (,0%) esperavam uma contagem menor que 5. A contagem mínima esperada é 136,75.

b. Computado apenas para uma tabela 2x2

Medidas Simétricas

		Valor	Significância Aproximada
Nominal por Nominal	Fi	-,047	,086
	V de Cramer	,047	,086
N de Casos Válidos		1313	

Estimativa de Risco

Valor	Intervalo de confiança de 95%	
	Inferior	Superior

Razão de Chances para Type of residency program (Non-Medical (Other HCP) / Medical)	,793	,608	1,034
Para grupo Perceived adequacy of the availability of personal protective equipment, when providing care for patients in the residency program = Poor adequacy	,833	,676	1,027
Para grupo Perceived adequacy of the availability of personal protective equipment, when providing care for patients in the residency program = Good adequacy	1,051	,993	1,112
N de Casos Válidos	1313		

Type of residency program * The participant exerts professional activity external to the residency program

Crosstab

			The participant exerts professional activity external to the residency program		
			No	Yes	Total
Type of residency program	Non-Medical (Other HCP)	Contagem	631	8	639
		% em Type of residency program	98,7%	1,3%	100,0%
		% do Total	48,1%	0,6%	48,7%
	Medical	Contagem	258	416	674
		% em Type of residency program	38,3%	61,7%	100,0%
		% do Total	19,6%	31,7%	51,3%
Total		Contagem	889	424	1313
		% em Type of residency program	67,7%	32,3%	100,0%
		% do Total	67,7%	32,3%	100,0%

Testes qui-quadrado

	Valor	gl	Significância Assintótica (Bilateral)	Sig exata (2 lados)	Sig exata (1 lado)
Qui-quadrado de Pearson	548,561[a]	1	,000		
Correção de continuidade[b]	545,799	1	,000		
Razão de verossimilhança	668,933	1	,000		
Teste Exato de Fisher				,000	,000
Associação Linear por Linear	548,143	1	,000		

N de Casos Válidos	1313				

a. 0 células (,0%) esperavam uma contagem menor que 5. A contagem mínima esperada é 206,35.

b. Computado apenas para uma tabela 2x2

Medidas Simétricas

		Valor	Significância Aproximada
Nominal por Nominal	Fi	,646	,000
	V de Cramer	,646	,000
N de Casos Válidos		1313	

Estimativa de Risco

	Valor	Intervalo de confiança de 95% Inferior	Superior
Razão de Chances para Type of residency program (Non-Medical (Other HCP) / Medical)	127,178	62,251	259,825
Para grupo The participant exerts professional activity external to the residency program = No	2,580	2,343	2,840
Para grupo The participant exerts professional activity external to the residency program = Yes	,020	,010	,040
N de Casos Válidos	1313		

```
Type of residency program * The participant provides direct health care for COVID19 patients
```

Crosstab

			The participant provides direct health care for COVID19 patients No	Yes	Total
Type of residency program	Non-Medical (Other HCP)	Contagem	393	246	639
		% em Type of residency program	61,5%	38,5%	100,0%
		% do Total	29,9%	18,7%	48,7%
	Medical	Contagem	130	544	674
		% em Type of residency program	19,3%	80,7%	100,0%
		% do Total	9,9%	41,4%	51,3%
Total		Contagem	523	790	1313
		% em Type of residency program	39,8%	60,2%	100,0%
		% do Total	39,8%	60,2%	100,0%

Testes qui-quadrado

	Valor	gl	Significância Assintótica (Bilateral)	Sig exata (2 lados)	Sig exata (1 lado)
Qui-quadrado de Pearson	243,905[a]	1	,000		
Correção de continuidade[b]	242,147	1	,000		
Razão de verossimilhança	252,790	1	,000		
Teste Exato de Fisher				,000	,000
Associação Linear por Linear	243,719	1	,000		
N de Casos Válidos	1313				

a. 0 células (,0%) esperavam uma contagem menor que 5. A contagem mínima esperada é 254,53.

b. Computado apenas para uma tabela 2x2

Medidas Simétricas

		Valor	Significância Aproximada
Nominal por Nominal	Fi	,431	,000
	V de Cramer	,431	,000
N de Casos Válidos		1313	

Estimativa de Risco

	Valor	Intervalo de confiança de 95%	
		Inferior	Superior
Razão de Chances para Type of residency program (Non-Medical (Other HCP) / Medical)	6,685	5,212	8,575
Para grupo The participant provides direct health care for COVID19 patients = No	3,189	2,700	3,765
Para grupo The participant provides direct health care for COVID19 patients = Yes	,477	,430	,530
N de Casos Válidos	1313		

```
Type of residency program * Cumulative weekly workload (five categories)
```

Crosstab

	Cumulative weekly workload (five categories)	Total

			|--- 24h	>24h --- 60h	>60h --- 90h	>90h --- 120h	>120h	
Type of residency program	Non-Medical (Other HCP)	Contagem	8	337	292	2	0	639
		% em Type of residency program	1,3%	52,7%	45,7%	0,3%	0,0%	100,0%
		% do Total	0,6%	25,7%	22,2%	0,2%	0,0%	48,7%
	Medical	Contagem	3	193	390	79	9	674
		% em Type of residency program	0,4%	28,6%	57,9%	11,7%	1,3%	100,0%
		% do Total	0,2%	14,7%	29,7%	6,0%	0,7%	51,3%
Total		Contagem	11	530	682	81	9	1313
		% em Type of residency program	0,8%	40,4%	51,9%	6,2%	0,7%	100,0%
		% do Total	0,8%	40,4%	51,9%	6,2%	0,7%	100,0%

Testes qui-quadrado

	Valor	gl	Significância Assintótica (Bilateral)
Qui-quadrado de Pearson	136,841[a]	4	,000
Razão de verossimilhança	161,188	4	,000
Associação Linear por Linear	127,465	1	,000
N de Casos Válidos	1313		

a. 2 células (20,0%) esperavam uma contagem menor que 5. A contagem mínima esperada é 4,38.

Medidas Simétricas

		Valor	Significância Aproximada
Nominal por Nominal	Fi	,323	,000
	V de Cramer	,323	,000
N de Casos Válidos		1313	

Estimativa de Risco

	Valor
Razão de Chances para Type of residency program (Non-Medical (Other HCP) / Medical)	[a]

a. Não é possível calcular as estatísticas de Estimativa de Risco. Elas são computadas apenas para uma tabela 2*2 sem células vazias.

Tabulações cruzadas

Observações

Saída criada		20-SEP-2020 12:04:57
Comentários		
Entrada	Dados	C:\Users\User\Documents\Pesquisa\Fellow\FellowGenData_V1.sav
	Conjunto de dados ativo	ConjuntodeDados1
	Filtro	<none>
	Ponderação	<none>
	Arquivo Dividido	<none>
	N de linhas em arquivo de dados de trabalho	1313
Tratamento de valor omisso	Definição de omisso	Os valores omissos definidos pelo usuário são tratados como omissos.
	Casos utilizados	As estatísticas de cada tabela são baseadas em todos os casos com dados válidos na(s) amplitude(s) especificada(s) para todas as variáveis de cada tabela.
Sintaxe		CROSSTABS /TABLES=RsPrgType BY Wkload_6060 /FORMAT=AVALUE TABLES /STATISTICS=CHISQ PHI RISK /CELLS=COUNT ROW TOTAL /COUNT ROUND CELL.
Recursos	Tempo do processador	00:00:00,00
	Tempo decorrido	00:00:00,00
	Dimensões solicitadas	2
	Células disponíveis	524245

Tabulação cruzada Type of residency program * Cumulative weekly workload < or >= 60h (dichotomous)

			Cumulative weekly workload < or >= 60h (dichotomous)		
			<=60h	>60h	Total
Type of residency program	Non-Medical (Other HCP)	Contagem	345	294	639
		% em Type of residency program	54,0%	46,0%	100,0%
		% do Total	26,3%	22,4%	48,7%
	Medical	Contagem	196	478	674
		% em Type of residency program	29,1%	70,9%	100,0%
		% do Total	14,9%	36,4%	51,3%
Total		Contagem	541	772	1313

	% em Type of residency program	41,2%	58,8%	100,0%
	% do Total	41,2%	58,8%	100,0%

Testes qui-quadrado

	Valor	gl	Significância Assintótica (Bilateral)	Sig exata (2 lados)	Sig exata (1 lado)
Qui-quadrado de Pearson	84,019[a]	1	,000		
Correção de continuidade[b]	82,994	1	,000		
Razão de verossimilhança	84,919	1	,000		
Teste Exato de Fisher				,000	,000
Associação Linear por Linear	83,955	1	,000		
N de Casos Válidos	1313				

a. 0 células (,0%) esperavam uma contagem menor que 5. A contagem mínima esperada é 263,29.

b. Computado apenas para uma tabela 2x2

Medidas Simétricas

		Valor	Significância Aproximada
Nominal por Nominal	Fi	,253	,000
	V de Cramer	,253	,000
N de Casos Válidos		1313	

Estimativa de Risco

	Valor	Intervalo de confiança de 95% Inferior	Superior
Razão de Chances para Type of residency program (Non-Medical (Other HCP) / Medical)	2,862	2,279	3,594
Para grupo Cumulative weekly workload < or >= 60h (dichotomous) = <=60h	1,857	1,617	2,131
Para grupo Cumulative weekly workload < or >= 60h (dichotomous) = >60h	,649	,589	,715
N de Casos Válidos	1313		

```
EXAMINE VARIABLES=Age DASS21_D DASS21_A DASS21_S PHQ9_Score BRCS_Score OLBI_D OLBI_E
OLBI_Total
    Autonomy PedagStruct BY RsPrgType
  /PLOT BOXPLOT HISTOGRAM NPPLOT
  /COMPARE GROUPS
  /PERCENTILES(5,10,25,50,75,90,95) HAVERAGE
  /STATISTICS DESCRIPTIVES
  /CINTERVAL 95
```

/MISSING PAIRWISE
/NOTOTAL.

Explorar

Observações

Saída criada		20-SEP-2020 12:10:04
Comentários		
Entrada	Dados	C:\Users\User\Documents\Pesquisa\Fellow\FellowGenData_V1.sav
	Conjunto de dados ativo	ConjuntodeDados1
	Filtro	<none>
	Ponderação	<none>
	Arquivo Dividido	<none>
	N de linhas em arquivo de dados de trabalho	1313
Tratamento de valor omisso	Definição de omisso	Os valores omissos definidos pelo usuário para variáveis dependentes são tratados como omissos.
	Casos utilizados	As estatísticas são baseadas em casos sem valores omissos para a variável dependente ou fatores que estão sendo analisados.
Sintaxe		EXAMINE VARIABLES=Age DASS21_D DASS21_A DASS21_S PHQ9_Score BRCS_Score OLBI_D OLBI_E OLBI_Total Autonomy PedagStruct BY RsPrgType /PLOT BOXPLOT HISTOGRAM NPPLOT /COMPARE GROUPS /PERCENTILES(5,10,25,50,75,90,95) HAVERAGE /STATISTICS DESCRIPTIVES /CINTERVAL 95 /MISSING PAIRWISE /NOTOTAL.
Recursos	Tempo do processador	00:00:13,33
	Tempo decorrido	00:00:10,91

Type of residency program

Descritivos

	Type of residency program			Estatística	Erro Erro
Age	Non-Medical (Other HCP)	Média		26,44	,180
		95% Intervalo de Confiança para Média	Limite inferior	26,09	
			Limite superior	26,79	
		5% da média aparada		25,90	
		Mediana		25,00	
		Variância		19,417	
		Erro Desvio		4,407	
		Mínimo		20	
		Máximo		55	
		Intervalo		35	
		Amplitude interquartil		3	
		Assimetria		2,651	,100
		Curtose		9,829	,199
	Medical	Média		29,21	,158
		95% Intervalo de Confiança para Média	Limite inferior	28,90	
			Limite superior	29,52	
		5% da média aparada		28,85	
		Mediana		28,00	
		Variância		15,749	
		Erro Desvio		3,968	
		Mínimo		23	
		Máximo		61	
		Intervalo		38	
		Amplitude interquartil		4	
		Assimetria		2,545	,097
		Curtose		13,368	,194
DASS21 Depression Score	Non-Medical (Other HCP)	Média		7,64	,216
		95% Intervalo de Confiança para Média	Limite inferior	7,22	
			Limite superior	8,07	
		5% da média aparada		7,39	
		Mediana		7,00	
		Variância		29,853	
		Erro Desvio		5,464	
		Mínimo		0	
		Máximo		21	
		Intervalo		21	
		Amplitude interquartil		8	
		Assimetria		,615	,097

		Curtose		-,447	,193
	Medical	Média		7,64	,225
		95% Intervalo de Confiança para Média	Limite inferior	7,20	
			Limite superior	8,08	
		5% da média aparada		7,36	
		Mediana		7,00	
		Variância		34,053	
		Erro Desvio		5,835	
		Mínimo		0	
		Máximo		21	
		Intervalo		21	
		Amplitude interquartil		8	
		Assimetria		,608	,094
		Curtose		-,587	,188
DASS21 Anxietty Score	Non-Medical (Other HCP)	Média		6,61	,208
		95% Intervalo de Confiança para Média	Limite inferior	6,20	
			Limite superior	7,02	
		5% da média aparada		6,29	
		Mediana		6,00	
		Variância		27,614	
		Erro Desvio		5,255	
		Mínimo		0	
		Máximo		21	
		Intervalo		21	
		Amplitude interquartil		8	
		Assimetria		,734	,097
		Curtose		-,236	,193
	Medical	Média		5,48	,193
		95% Intervalo de Confiança para Média	Limite inferior	5,10	
			Limite superior	5,86	
		5% da média aparada		5,08	
		Mediana		4,00	
		Variância		24,999	
		Erro Desvio		5,000	
		Mínimo		0	
		Máximo		21	
		Intervalo		21	
		Amplitude interquartil		8	
		Assimetria		,930	,094
		Curtose		,350	,188
DASS21 Stress Score	Non-Medical (Other HCP)	Média		10,27	,209
		95% Intervalo de Confiança para Média	Limite inferior	9,86	
			Limite superior	10,68	
		5% da média aparada		10,26	
		Mediana		10,00	
		Variância		27,830	

		Erro Desvio		5,275	
		Mínimo		0	
		Máximo		21	
		Intervalo		21	
		Amplitude interquartil		8	
		Assimetria		,082	,097
		Curtose		-,781	,193
	Medical	Média		10,04	,211
		95% Intervalo de Confiança para Média	Limite inferior	9,63	
			Limite superior	10,46	
		5% da média aparada		10,00	
		Mediana		10,00	
		Variância		29,900	
		Erro Desvio		5,468	
		Mínimo		0	
		Máximo		21	
		Intervalo		21	
		Amplitude interquartil		8	
		Assimetria		,133	,094
		Curtose		-,761	,188
PHQ9 Depression Score	Non-Medical (Other HCP)	Média		12,31	,248
		95% Intervalo de Confiança para Média	Limite inferior	11,82	
			Limite superior	12,80	
		5% da média aparada		12,26	
		Mediana		12,00	
		Variância		39,402	
		Erro Desvio		6,277	
		Mínimo		0	
		Máximo		27	
		Intervalo		27	
		Amplitude interquartil		10	
		Assimetria		,122	,097
		Curtose		-,744	,193
	Medical	Média		11,71	,254
		95% Intervalo de Confiança para Média	Limite inferior	11,21	
			Limite superior	12,21	
		5% da média aparada		11,58	
		Mediana		11,00	
		Variância		43,625	
		Erro Desvio		6,605	
		Mínimo		0	
		Máximo		27	
		Intervalo		27	
		Amplitude interquartil		10	
		Assimetria		,256	,094
		Curtose		-,747	,188

BRCS Score	Non-Medical (Other HCP)	Média		12,26	,145
		95% Intervalo de Confiança para Média	Limite inferior	11,98	
			Limite superior	12,55	
		5% da média aparada		12,25	
		Mediana		12,00	
		Variância		13,350	
		Erro Desvio		3,654	
		Mínimo		4	
		Máximo		20	
		Intervalo		16	
		Amplitude interquartil		5	
		Assimetria		,064	,097
		Curtose		-,619	,193
	Medical	Média		12,56	,150
		95% Intervalo de Confiança para Média	Limite inferior	12,26	
			Limite superior	12,85	
		5% da média aparada		12,58	
		Mediana		12,00	
		Variância		15,070	
		Erro Desvio		3,882	
		Mínimo		4	
		Máximo		20	
		Intervalo		16	
		Amplitude interquartil		5	
		Assimetria		,001	,094
		Curtose		-,615	,188
OLBI Disengagement Score	Non-Medical (Other HCP)	Média		2,7365	,03216
		95% Intervalo de Confiança para Média	Limite inferior	2,6734	
			Limite superior	2,7996	
		5% da média aparada		2,7230	
		Mediana		2,6250	
		Variância		,661	
		Erro Desvio		,81289	
		Mínimo		1,00	
		Máximo		5,00	
		Intervalo		4,00	
		Amplitude interquartil		1,25	
		Assimetria		,204	,097
		Curtose		-,303	,193
	Medical	Média		2,8095	,03324
		95% Intervalo de Confiança para Média	Limite inferior	2,7443	
			Limite superior	2,8748	
		5% da média aparada		2,8018	
		Mediana		2,7500	
		Variância		,745	
		Erro Desvio		,86305	

		Mínimo		1,00	
		Máximo		5,00	
		Intervalo		4,00	
		Amplitude interquartil		1,13	
		Assimetria		,158	,094
		Curtose		-,532	,188
OLBI Exhaustion Score	Non-Medical (Other HCP)	Média		3,5808	,02740
		95% Intervalo de Confiança para Média	Limite inferior	3,5270	
			Limite superior	3,6346	
		5% da média aparada		3,5995	
		Mediana		3,6300	
		Variância		,480	
		Erro Desvio		,69267	
		Mínimo		1,50	
		Máximo		5,00	
		Intervalo		3,50	
		Amplitude interquartil		,87	
		Assimetria		-,373	,097
		Curtose		-,031	,193
	Medical	Média		3,5298	,02985
		95% Intervalo de Confiança para Média	Limite inferior	3,4711	
			Limite superior	3,5884	
		5% da média aparada		3,5584	
		Mediana		3,6300	
		Variância		,601	
		Erro Desvio		,77501	
		Mínimo		1,00	
		Máximo		5,00	
		Intervalo		4,00	
		Amplitude interquartil		1,00	
		Assimetria		-,607	,094
		Curtose		,278	,188
OLBI Total Score	Non-Medical (Other HCP)	Média		3,1586	,02615
		95% Intervalo de Confiança para Média	Limite inferior	3,1073	
			Limite superior	3,2100	
		5% da média aparada		3,1611	
		Mediana		3,1900	
		Variância		,437	
		Erro Desvio		,66095	
		Mínimo		1,25	
		Máximo		4,88	
		Intervalo		3,63	
		Amplitude interquartil		,94	
		Assimetria		-,042	,097
		Curtose		-,272	,193
	Medical	Média		3,1698	,02832

		95% Intervalo de Confiança para Média	Limite inferior	3,1142	
			Limite superior	3,2254	
		5% da média aparada		3,1823	
		Mediana		3,1900	
		Variância		,540	
		Erro Desvio		,73517	
		Mínimo		1,00	
		Máximo		5,00	
		Intervalo		4,00	
		Amplitude interquartil		1,06	
		Assimetria		-,203	,094
		Curtose		-,244	,188
Na sua opinião, qual o seu grau de autonomia para decidir condutas no trabalho? (EAV 1-10)	Non-Medical (Other HCP)	Média		6,58	,083
		95% Intervalo de Confiança para Média	Limite inferior	6,42	
			Limite superior	6,75	
		5% da média aparada		6,68	
		Mediana		7,00	
		Variância		4,388	
		Erro Desvio		2,095	
		Mínimo		1	
		Máximo		10	
		Intervalo		9	
		Amplitude interquartil		3	
		Assimetria		-,728	,097
		Curtose		,050	,193
	Medical	Média		6,43	,081
		95% Intervalo de Confiança para Média	Limite inferior	6,27	
			Limite superior	6,59	
		5% da média aparada		6,51	
		Mediana		7,00	
		Variância		4,472	
		Erro Desvio		2,115	
		Mínimo		1	
		Máximo		10	
		Intervalo		9	
		Amplitude interquartil		3	
		Assimetria		-,664	,094
		Curtose		-,151	,188
Na sua opinião, qual o grau de adequação da organização pedagógica de seu programa de residência profissional? (EAV 1-10)	Non-Medical (Other HCP)	Média		5,34	,099
		95% Intervalo de Confiança para Média	Limite inferior	5,15	
			Limite superior	5,54	
		5% da média aparada		5,35	
		Mediana		5,00	
		Variância		6,260	
		Erro Desvio		2,502	
		Mínimo		1	

		Máximo		10	
		Intervalo		9	
		Amplitude interquartil		4	
		Assimetria		-,143	,097
		Curtose		-,982	,193
	Medical	Média		6,18	,091
		95% Intervalo de Confiança para Média	Limite inferior	6,00	
			Limite superior	6,36	
		5% da média aparada		6,26	
		Mediana		7,00	
		Variância		5,609	
		Erro Desvio		2,368	
		Mínimo		1	
		Máximo		10	
		Intervalo		9	
		Amplitude interquartil		3	
		Assimetria		-,534	,094
		Curtose		-,511	,188

Percentis

			Percentis						
		Type of residency program	5	10	25	50	75	90	95
Média Ponderada (Definição 1)	Age	Non-Medical (Other HCP)	22,00	23,00	24,00	25,00	27,00	31,00	35,00
		Medical	25,00	25,00	27,00	28,00	31,00	34,00	36,00
	DASS21 Depression Score	Non-Medical (Other HCP)	,00	1,00	3,00	7,00	11,00	16,00	19,00
		Medical	,00	1,00	3,00	7,00	11,25	17,00	19,00
	DASS21 Anxietty Score	Non-Medical (Other HCP)	,00	1,00	2,00	6,00	10,00	14,00	17,00
		Medical	,00	,00	1,00	4,00	9,00	12,00	15,00
	DASS21 Stress Score	Non-Medical (Other HCP)	1,00	3,00	6,00	10,00	14,00	17,00	19,00
		Medical	1,00	3,00	6,00	10,00	14,00	18,00	20,00
	PHQ9 Depression Score	Non-Medical (Other HCP)	2,00	4,00	7,00	12,00	17,00	21,00	23,00
		Medical	2,00	3,00	6,00	11,00	16,00	21,00	23,00
	BRCS Score	Non-Medical (Other HCP)	6,00	8,00	10,00	12,00	15,00	17,00	18,00
		Medical	6,00	7,00	10,00	12,00	15,00	18,00	19,00
	OLBI Disengagement Score	Non-Medical (Other HCP)	1,3750	1,6250	2,1250	2,6250	3,3750	3,7500	4,1250
		Medical	1,3750	1,6250	2,2500	2,7500	3,3750	4,0000	4,2500
	OLBI Exhaustion Score	Non-Medical (Other HCP)	2,2900	2,6300	3,1300	3,6300	4,0000	4,5000	4,7100
		Medical	2,1300	2,5000	3,1300	3,6300	4,1300	4,4300	4,6300
	OLBI Total Score	Non-Medical (Other HCP)	2,0600	2,3100	2,6900	3,1900	3,6300	4,0400	4,2500
		Medical	1,8800	2,2500	2,6300	3,1900	3,6900	4,1300	4,3800
	Na sua opinião, qual o seu grau de autonomia para decidir condutas no trabalho? (EAV 1-10)	Non-Medical (Other HCP)	2,00	4,00	5,00	7,00	8,00	9,00	9,00
		Medical	2,00	3,00	5,00	7,00	8,00	9,00	9,00

	Na sua opinião, qual o grau de adequação da organização pedagógica de seu programa de residência profissional? (EAV 1-10)	Non-Medical (Other HCP)	1,00	2,00	3,00	5,00	7,00	8,00	9,00
		Medical	1,75	3,00	5,00	7,00	8,00	9,00	10,00
Teste de Tukey	Age	Non-Medical (Other HCP)			24,00	25,00	27,00		
		Medical			27,00	28,00	31,00		
	DASS21 Depression Score	Non-Medical (Other HCP)			3,00	7,00	11,00		
		Medical			3,00	7,00	11,00		
	DASS21 Anxietty Score	Non-Medical (Other HCP)			2,00	6,00	10,00		
		Medical			1,00	4,00	9,00		
	DASS21 Stress Score	Non-Medical (Other HCP)			6,00	10,00	14,00		
		Medical			6,00	10,00	14,00		
	PHQ9 Depression Score	Non-Medical (Other HCP)			7,00	12,00	17,00		
		Medical			6,00	11,00	16,00		
	BRCS Score	Non-Medical (Other HCP)			10,00	12,00	15,00		
		Medical			10,00	12,00	15,00		
	OLBI Disengagement Score	Non-Medical (Other HCP)			2,1250	2,6250	3,3125		
		Medical			2,2500	2,7500	3,3750		
	OLBI Exhaustion Score	Non-Medical (Other HCP)			3,1300	3,6300	4,0000		
		Medical			3,1300	3,6300	4,1300		
	OLBI Total Score	Non-Medical (Other HCP)			2,6900	3,1900	3,6300		
		Medical			2,6300	3,1900	3,6900		
	Na sua opinião, qual o seu grau de autonomia para decidir condutas no trabalho? (EAV 1-10)	Non-Medical (Other HCP)			5,00	7,00	8,00		
		Medical			5,00	7,00	8,00		
	Na sua opinião, qual o grau de adequação da organização pedagógica de seu programa de residência profissional? (EAV 1-10)	Non-Medical (Other HCP)			3,00	5,00	7,00		
		Medical			5,00	7,00	8,00		

Testes de Normalidade

		Kolmogorov-Smirnov[a]			Shapiro-Wilk		
	Type of residency program	Estatística	df	Sig.	Estatística	df	Sig.
Age	Non-Medical (Other HCP)	,216	599	,000	,749	599	,000
	Medical	,160	631	,000	,823	631	,000
DASS21 Depression Score	Non-Medical (Other HCP)	,104	639	,000	,944	639	,000
	Medical	,117	674	,000	,933	674	,000
DASS21 Anxietty Score	Non-Medical (Other HCP)	,113	639	,000	,929	639	,000
	Medical	,136	674	,000	,902	674	,000
DASS21 Stress Score	Non-Medical (Other HCP)	,077	639	,000	,979	639	,000
	Medical	,075	674	,000	,977	674	,000
PHQ9 Depression Score	Non-Medical (Other HCP)	,069	639	,000	,984	639	,000

	Medical	,073	674	,000	,976	674	,000
BRCS Score	Non-Medical (Other HCP)	,069	639	,000	,983	639	,000
	Medical	,067	674	,000	,981	674	,000
OLBI Disengagement Score	Non-Medical (Other HCP)	,058	639	,000	,991	639	,000
	Medical	,053	674	,000	,989	674	,000
OLBI Exhaustion Score	Non-Medical (Other HCP)	,058	639	,000	,985	639	,000
	Medical	,083	674	,000	,973	674	,000
OLBI Total Score	Non-Medical (Other HCP)	,028	639	,200*	,997	639	,340
	Medical	,032	674	,096	,994	674	,006
Na sua opinião, qual o seu grau de autonomia para decidir condutas no trabalho? (EAV 1-10)	Non-Medical (Other HCP)	,186	639	,000	,932	639	,000
	Medical	,188	674	,000	,935	674	,000
Na sua opinião, qual o grau de adequação da organização pedagógica de seu programa de residência profissional? (EAV 1-10)	Non-Medical (Other HCP)	,134	639	,000	,952	639	,000
	Medical	,169	674	,000	,940	674	,000

*. Este é um limite inferior da significância verdadeira.

a. Correlação de Significância de Lilliefors

Age

Histogramas

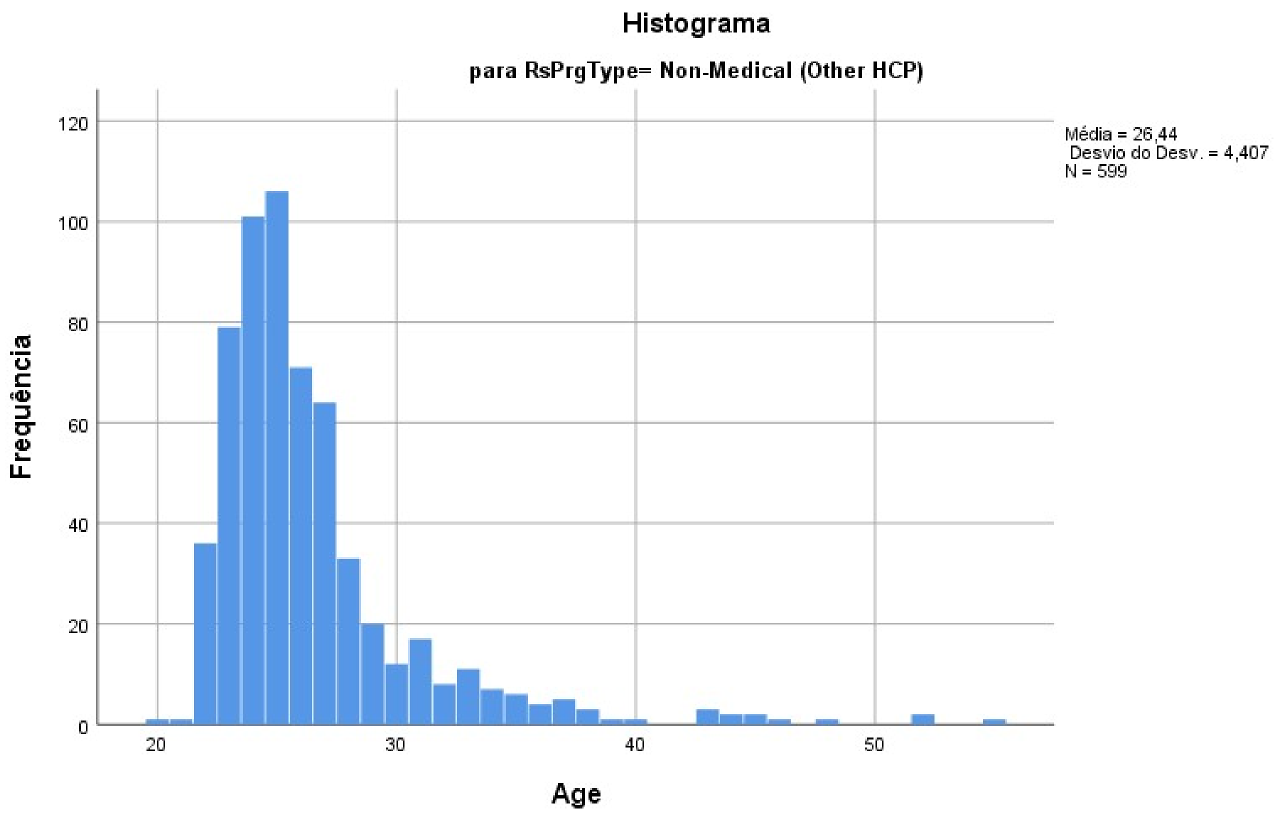

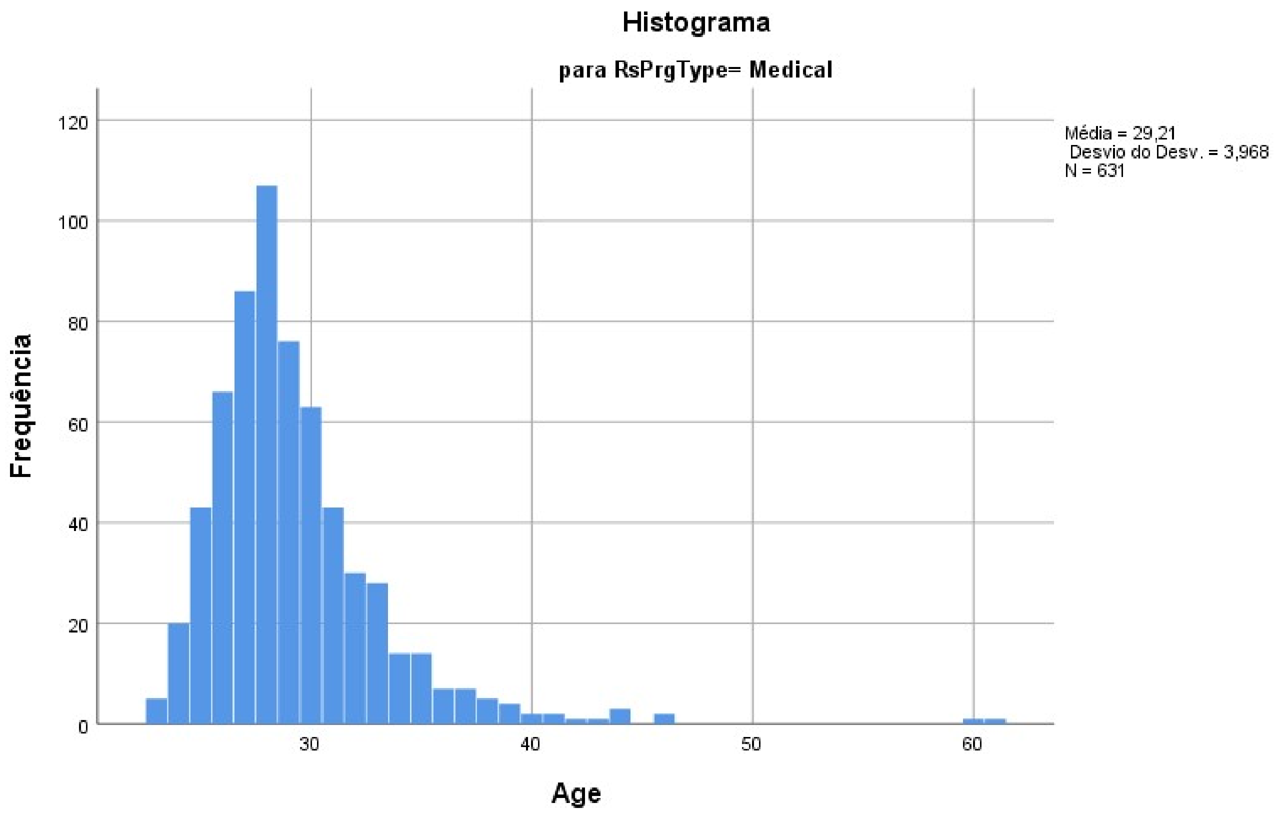

Gráfico Q-Q normais

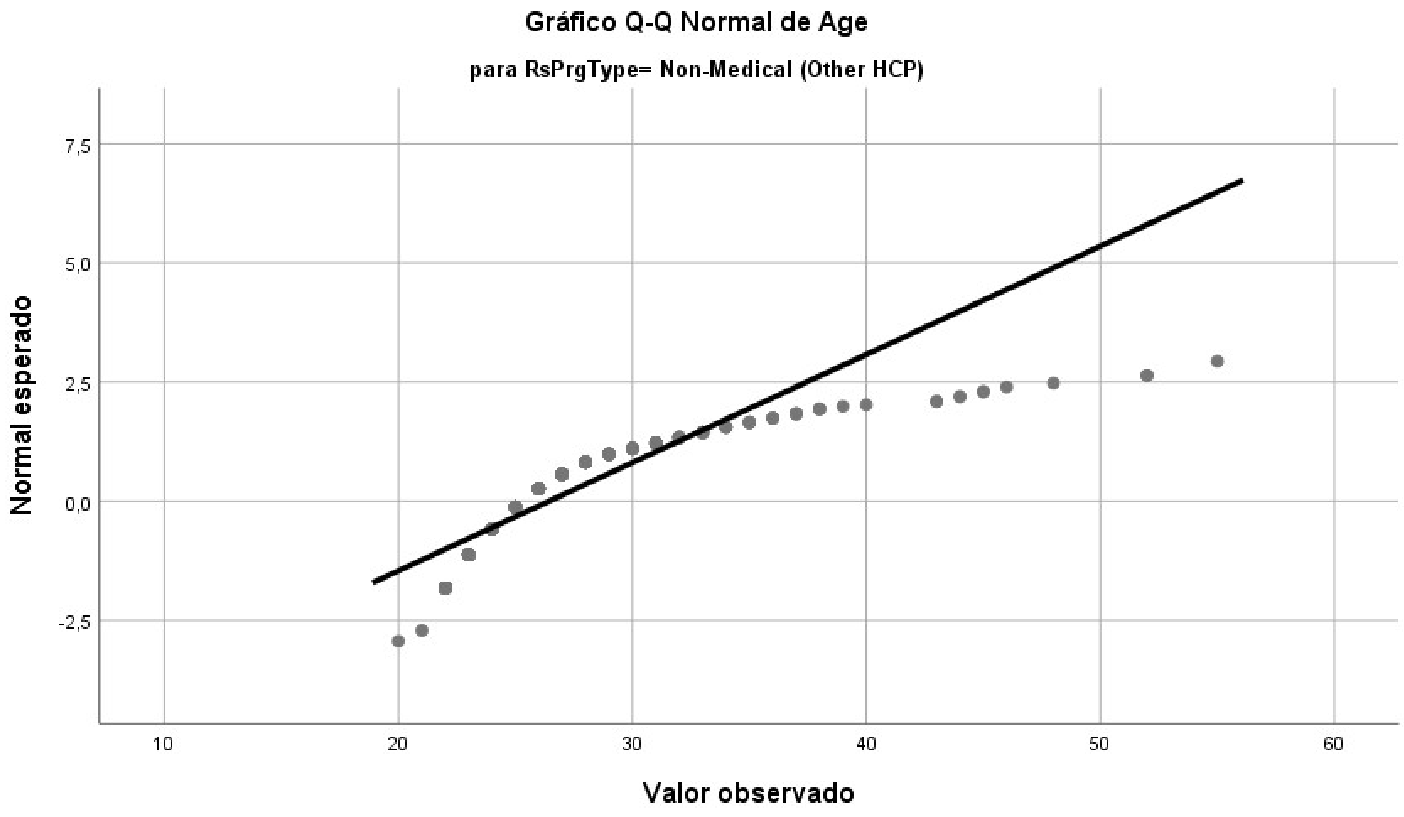

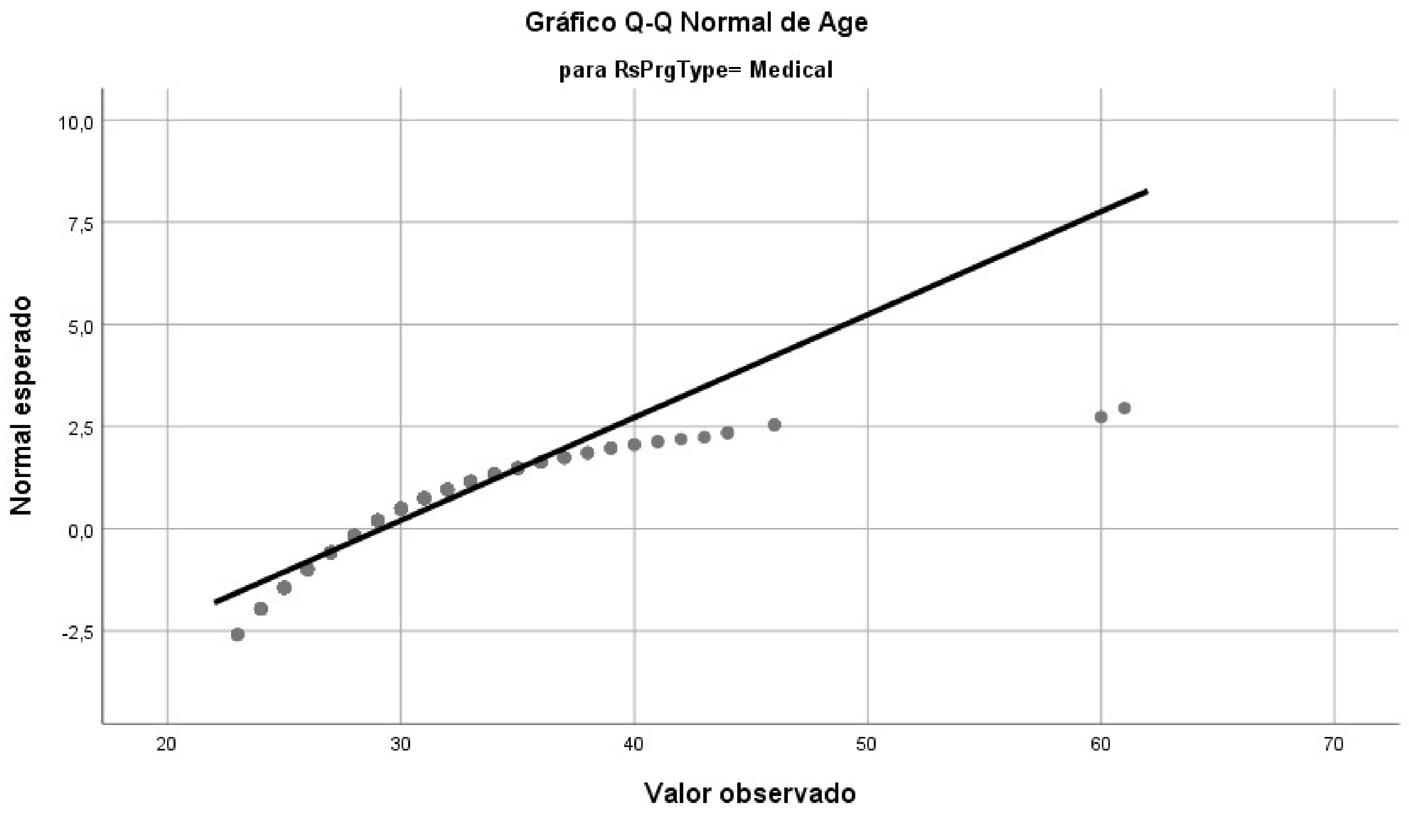

Gráfico Q-Q normais sem tendência

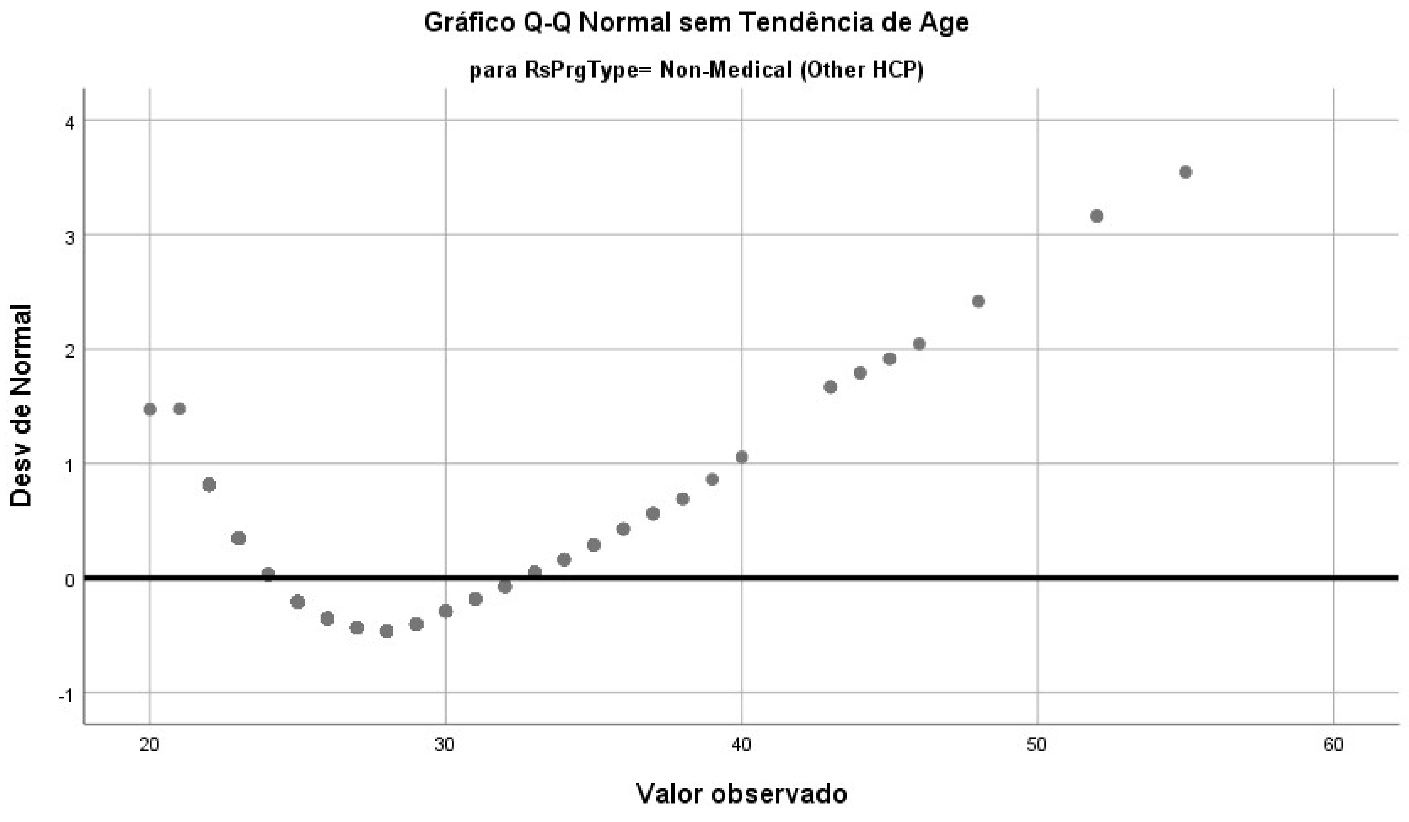

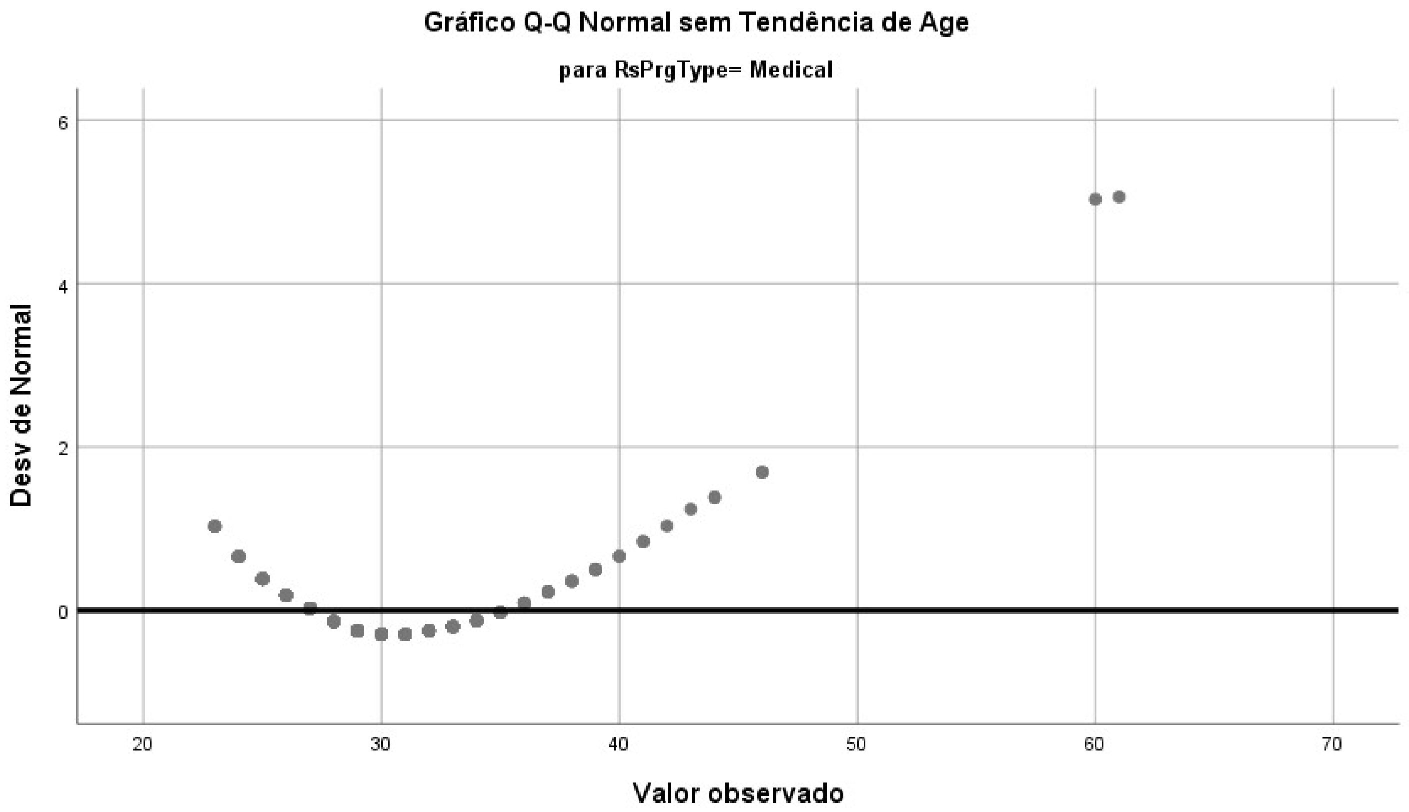

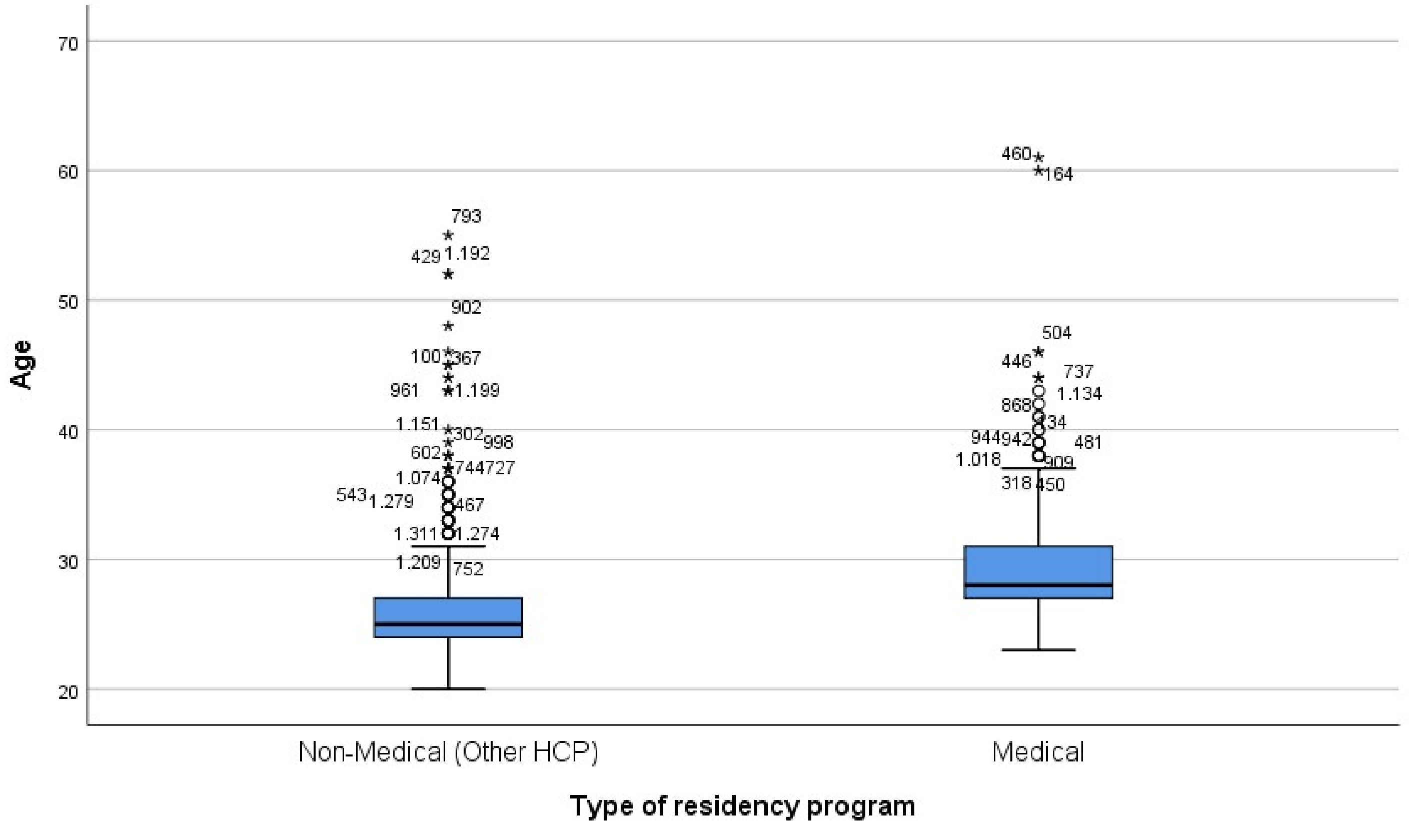

DASS21 Depression Score

Histogramas

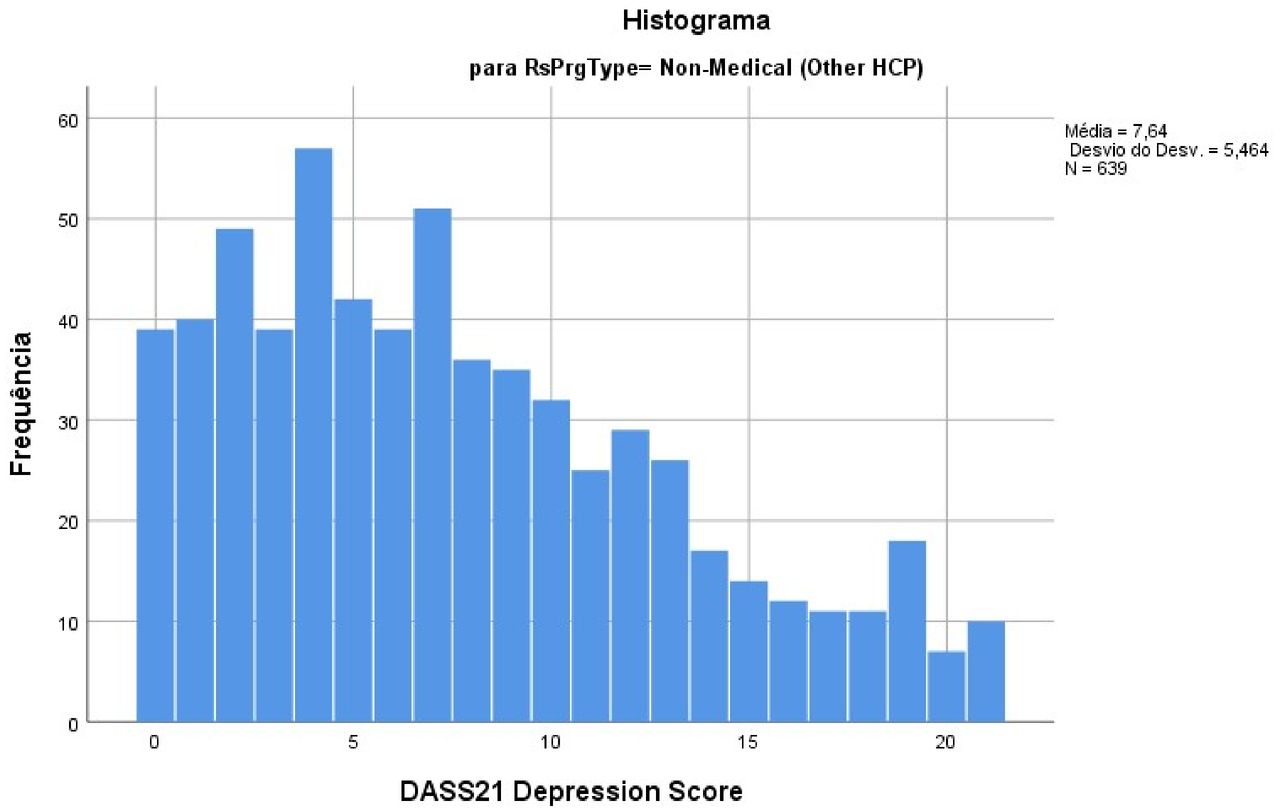

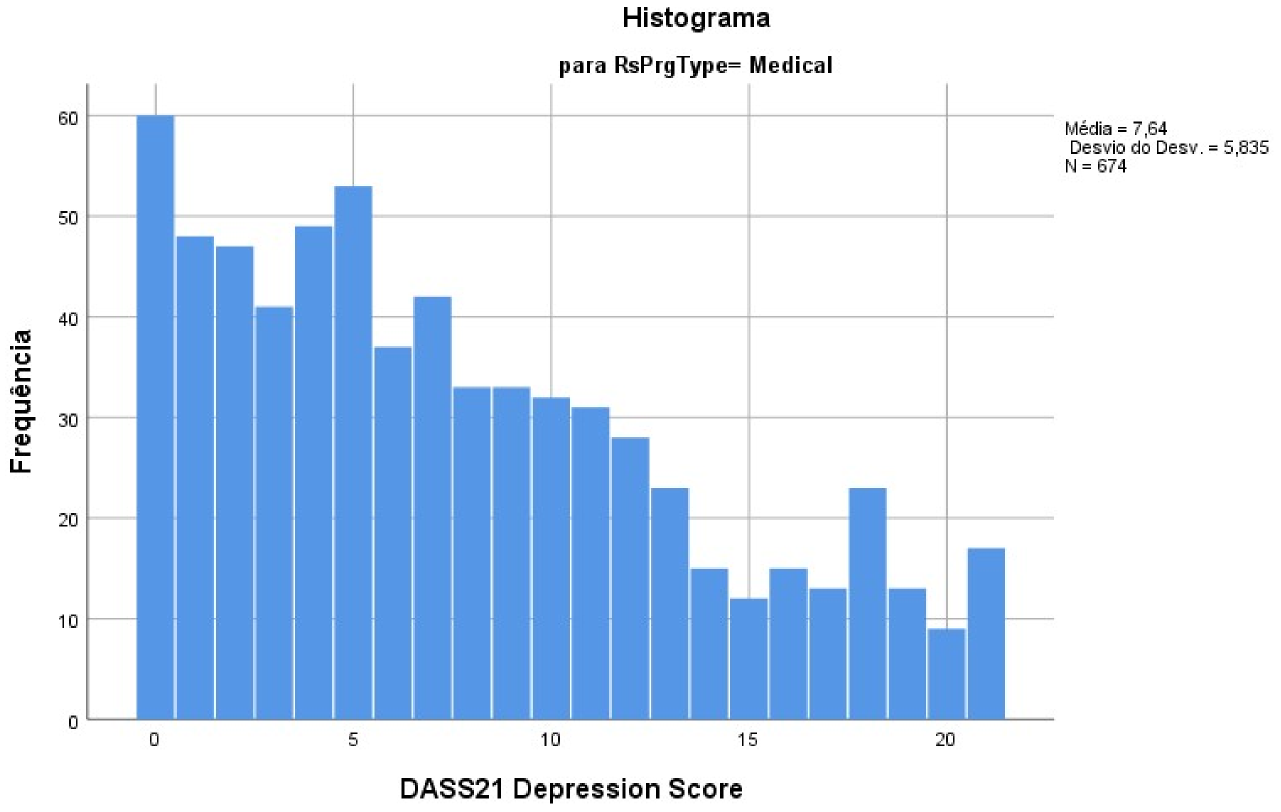

Gráfico Q-Q normais

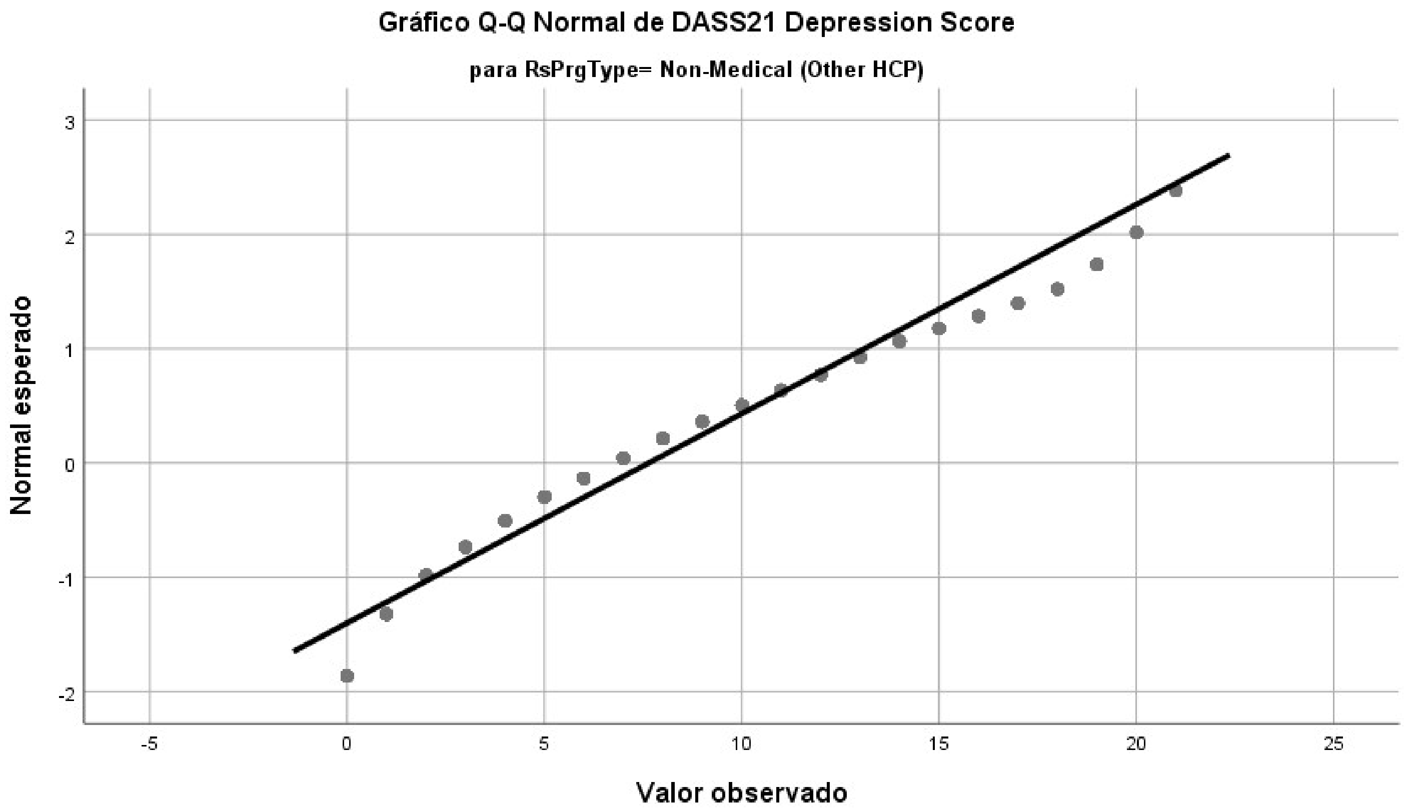

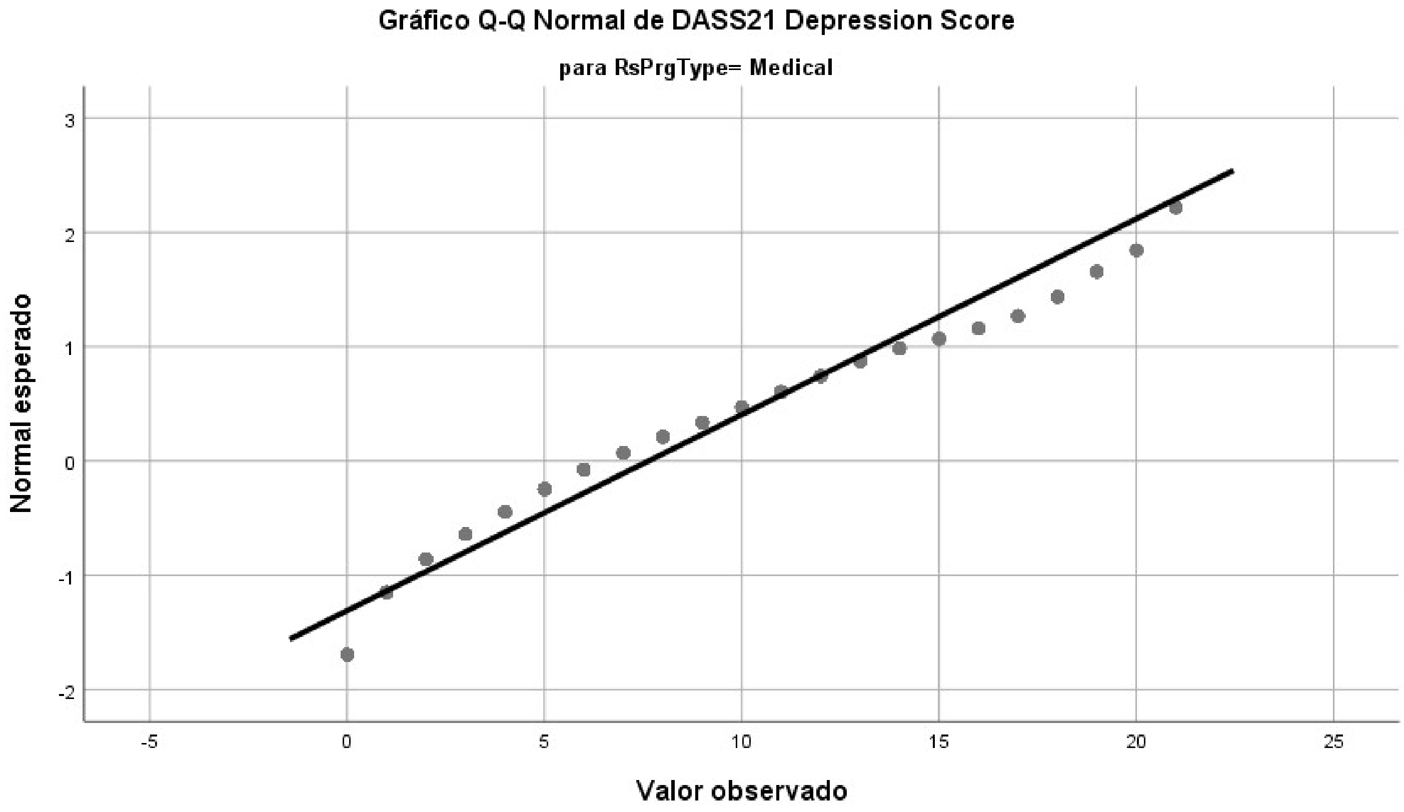

Gráfico Q-Q normais sem tendência

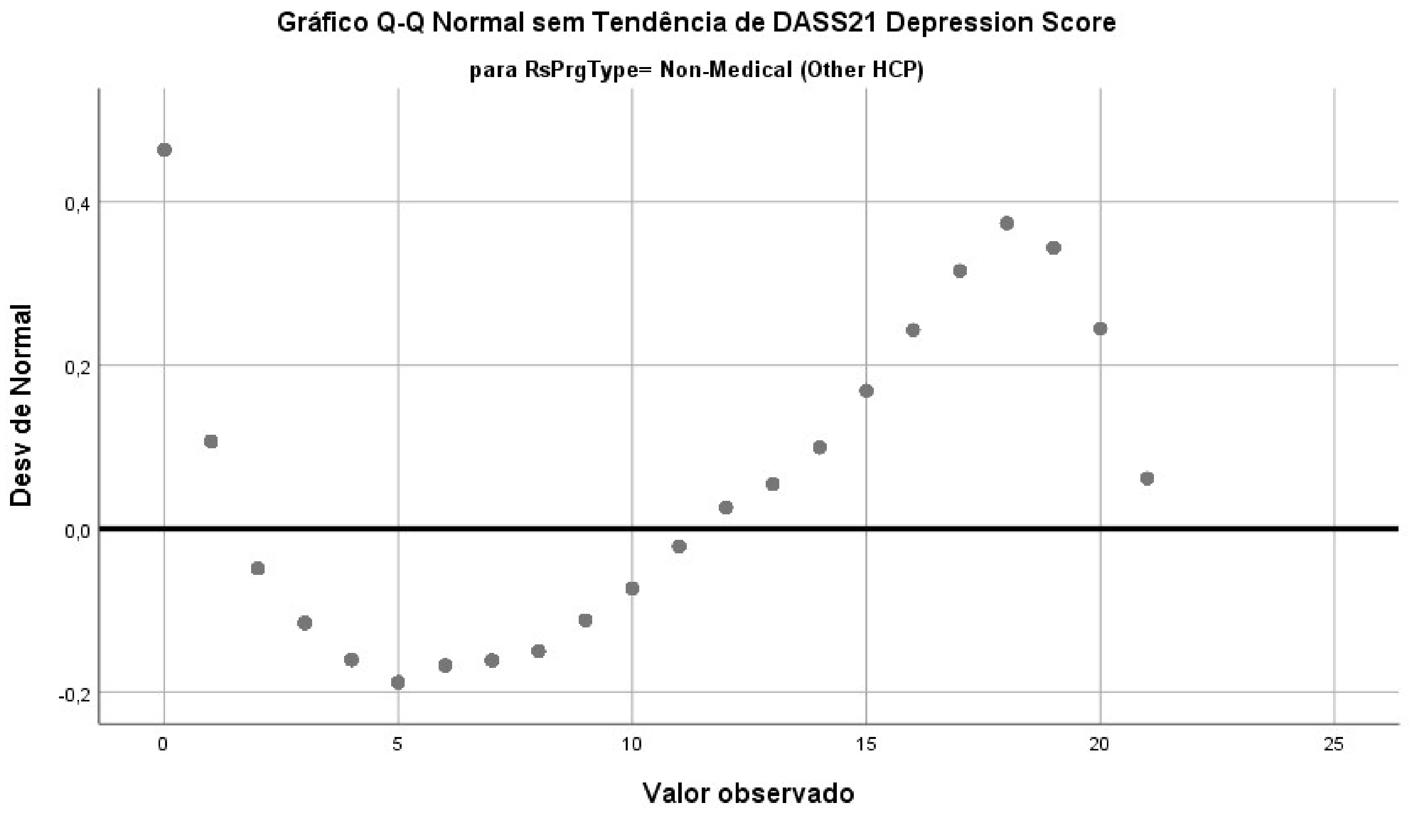

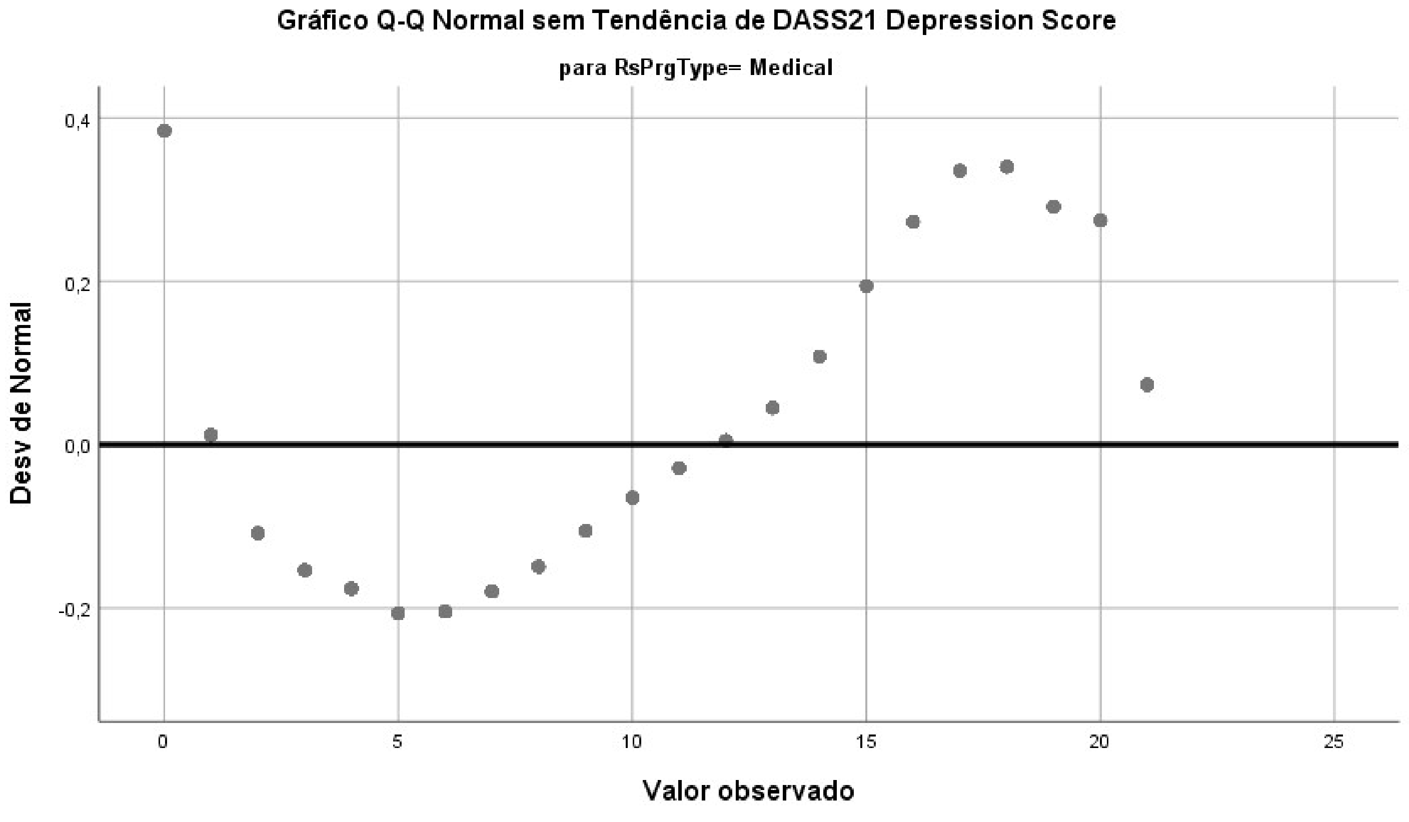

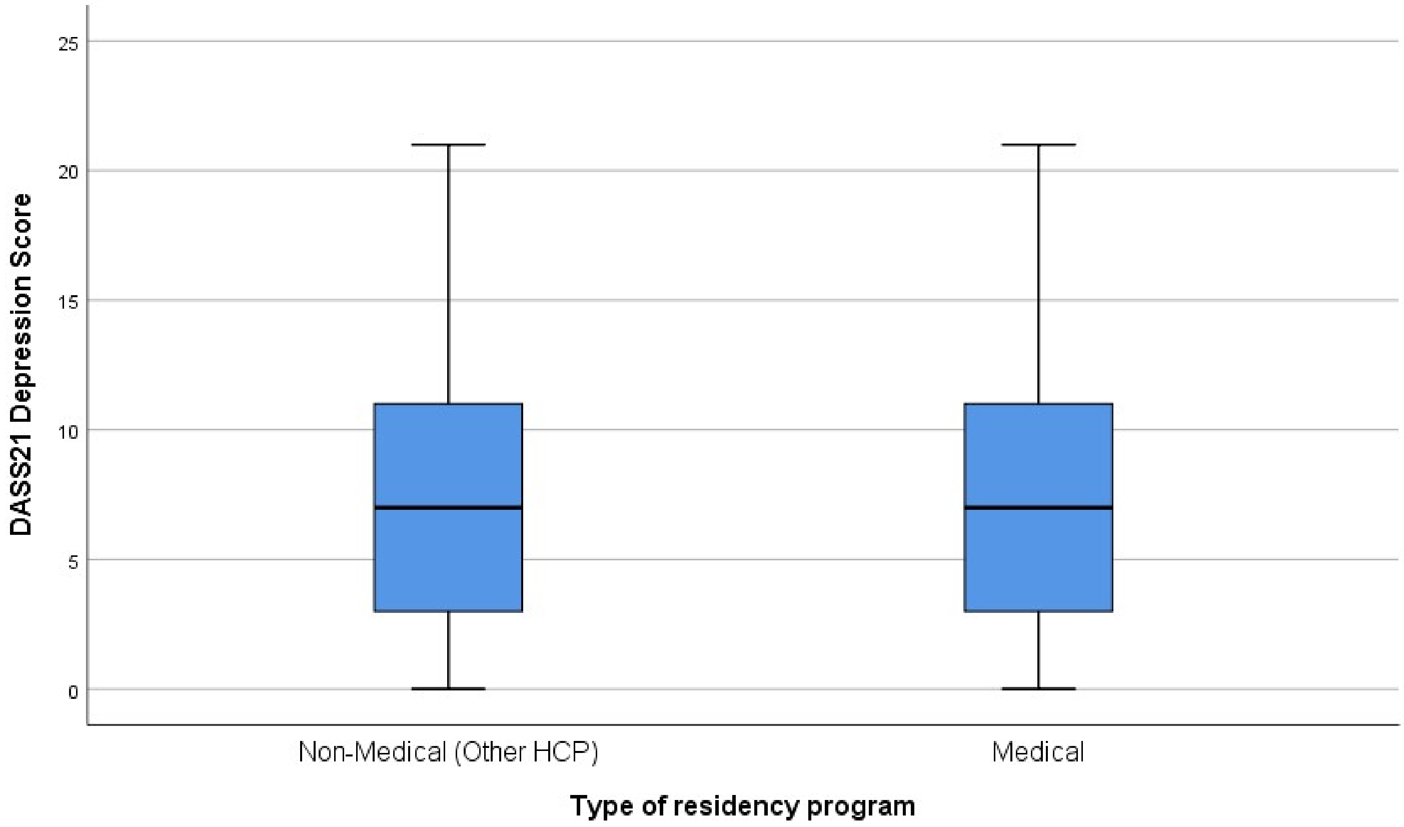

DASS21 Anxietty Score

Histogramas

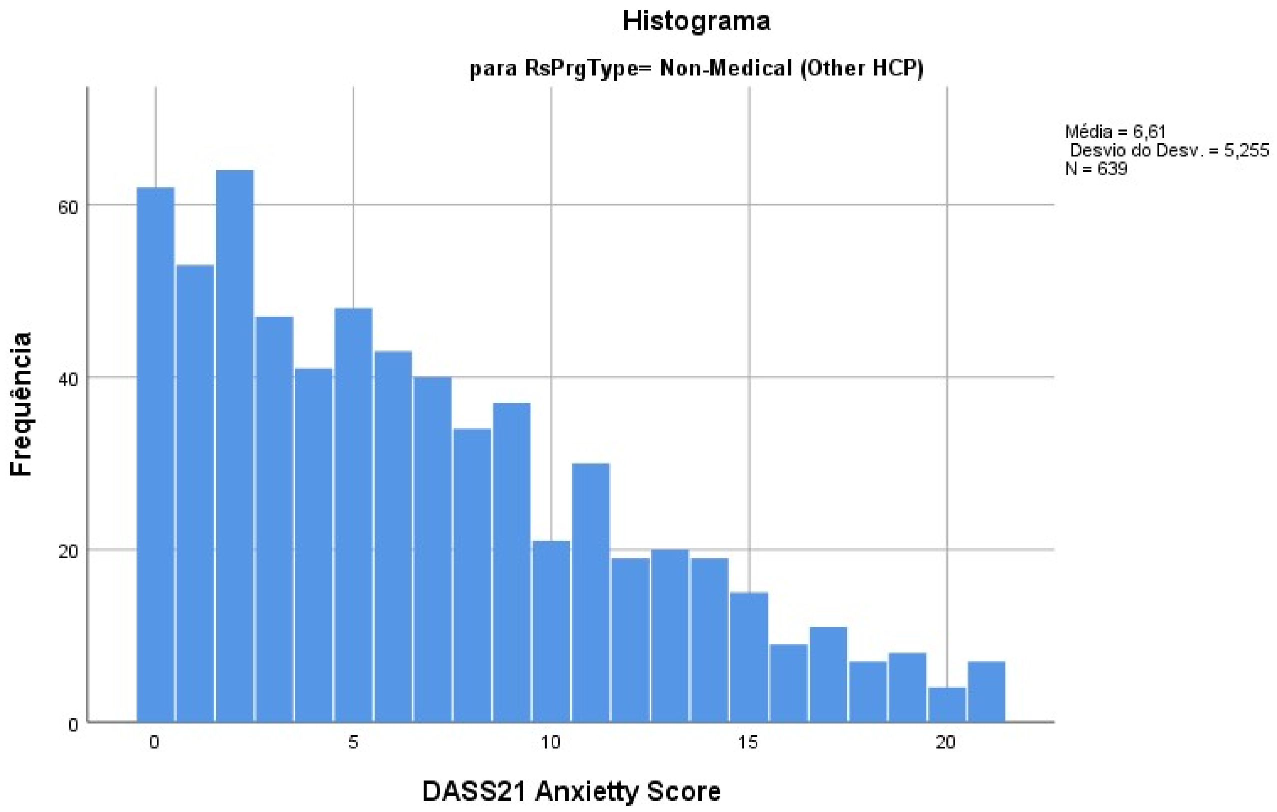

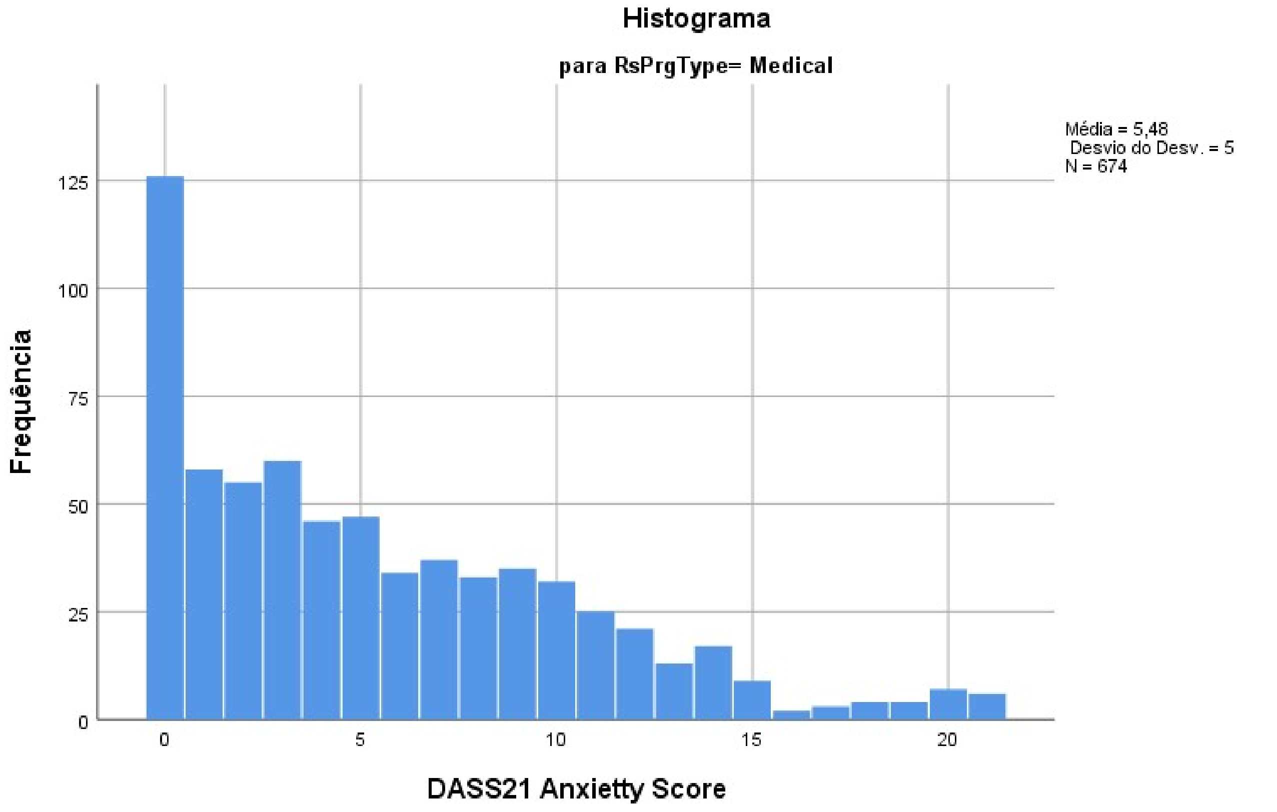

Gráfico Q-Q normais

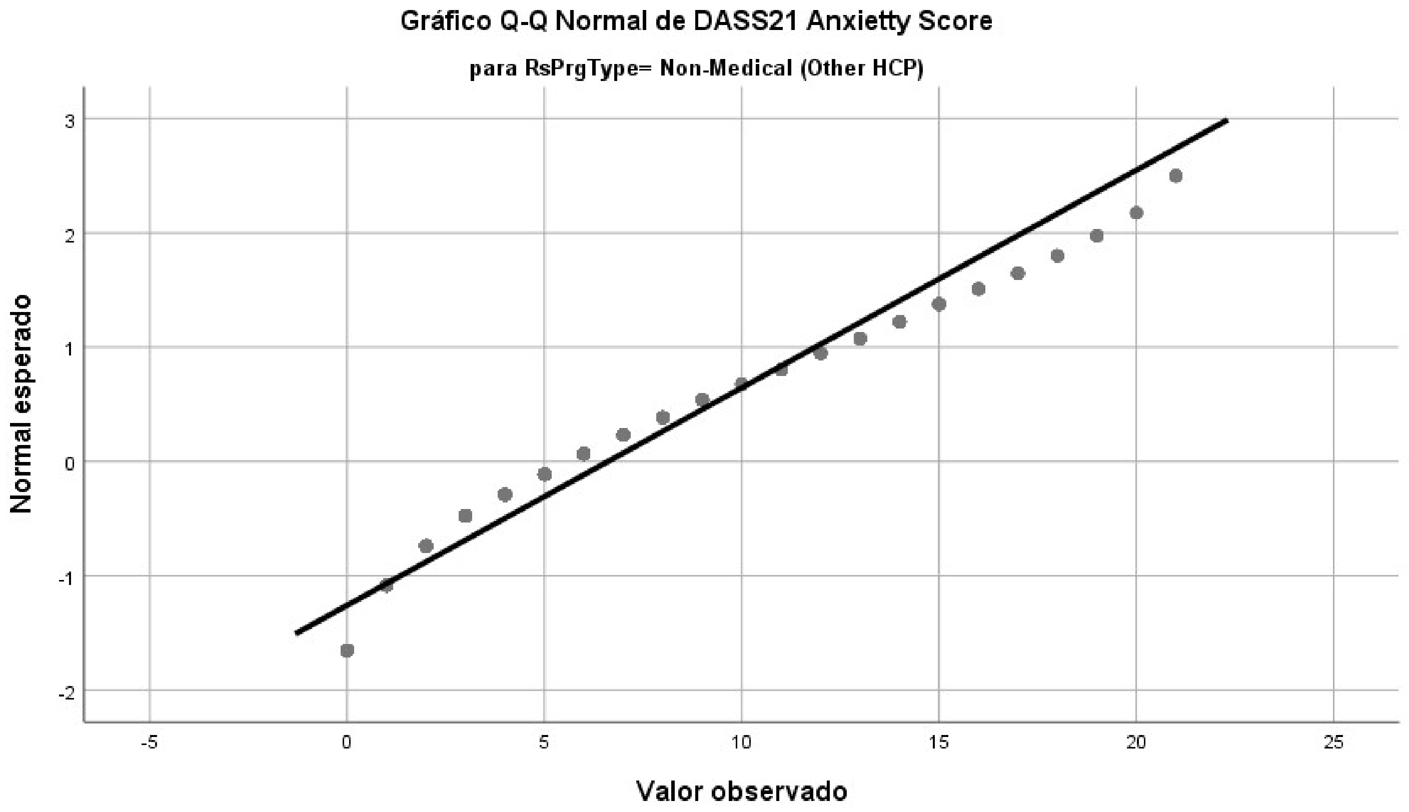

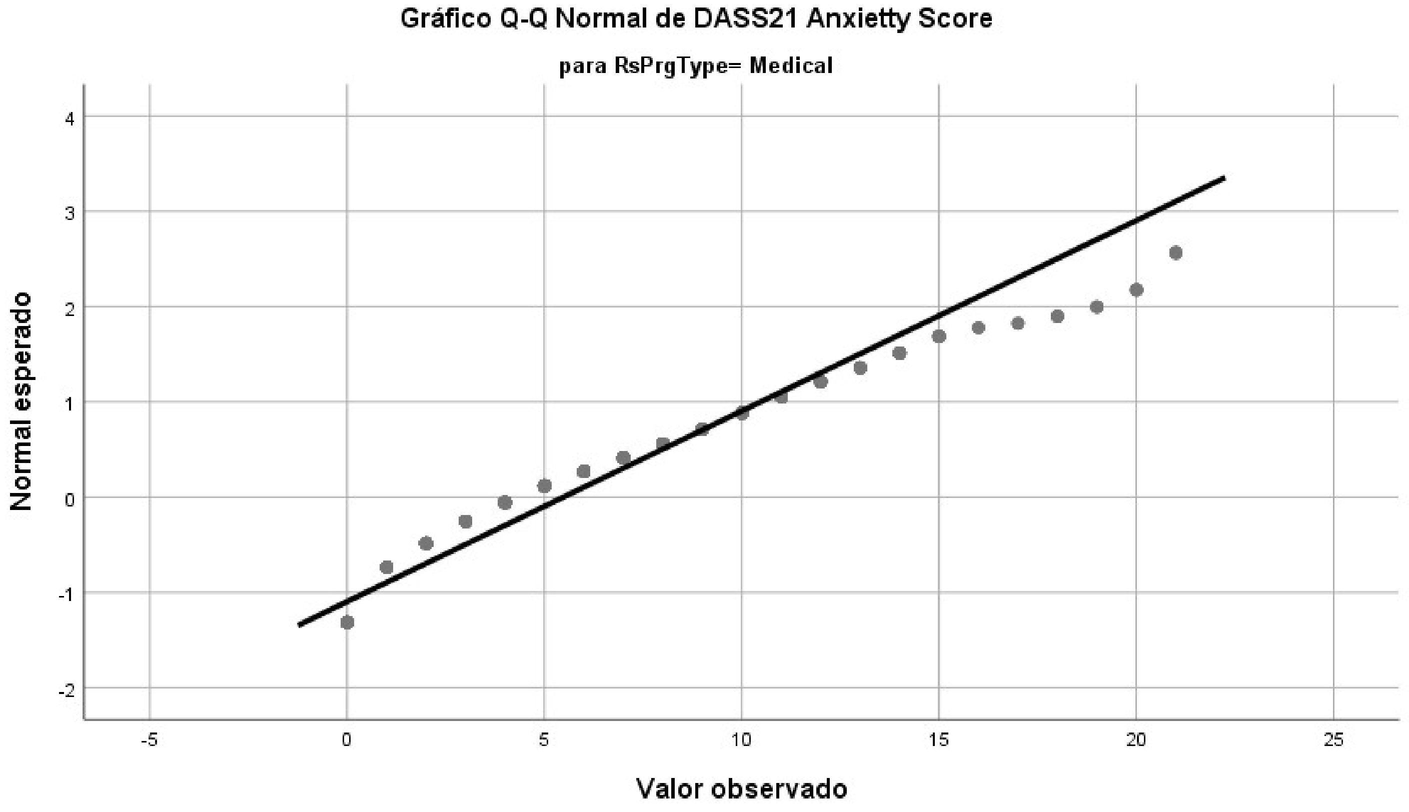

Gráfico Q-Q normais sem tendência

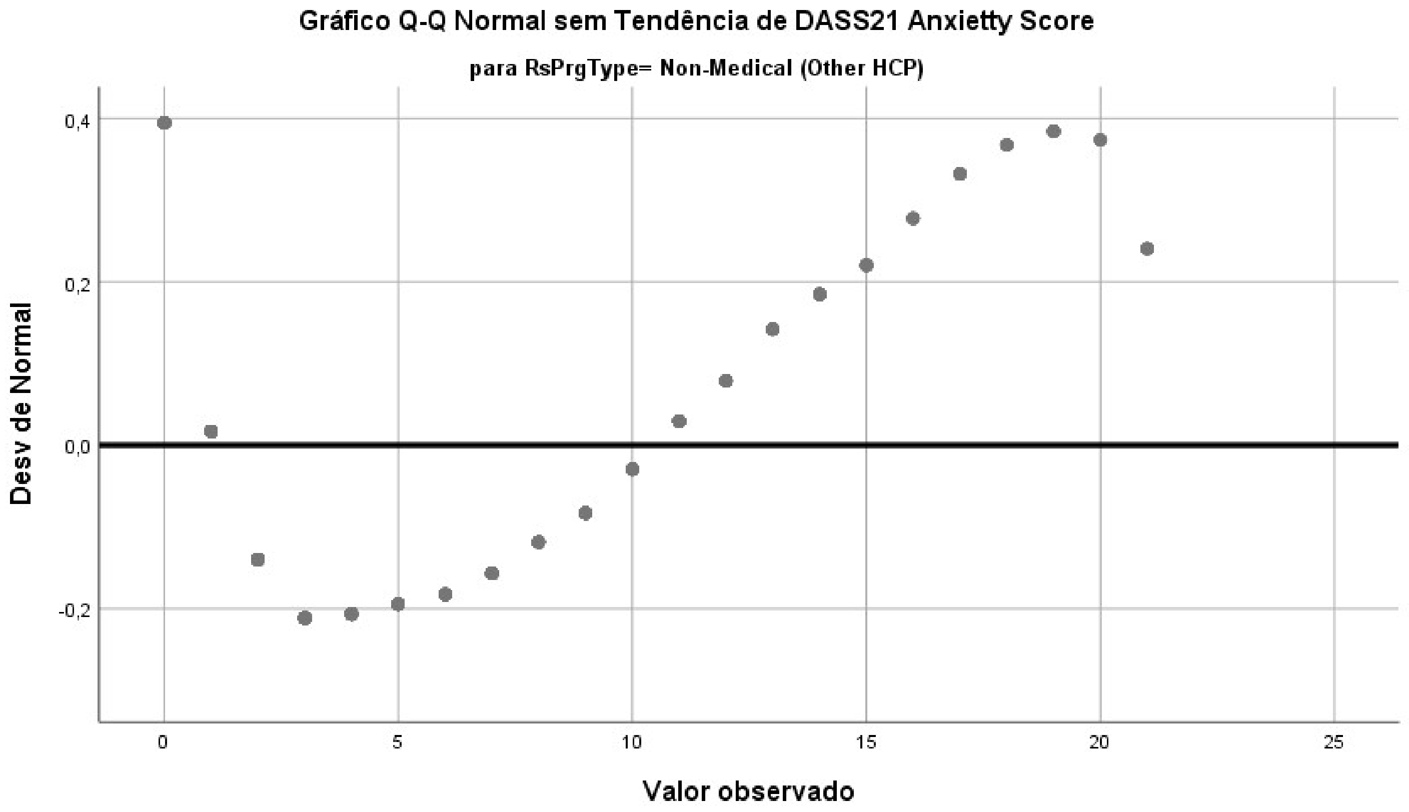
Gráfico Q-Q Normal sem Tendência de DASS21 Anxietty Score
para RsPrgType= Non-Medical (Other HCP)
Desv de Normal
Valor observado
0,4
0,2
0,0
-0,2
0
5
10
15
20
25

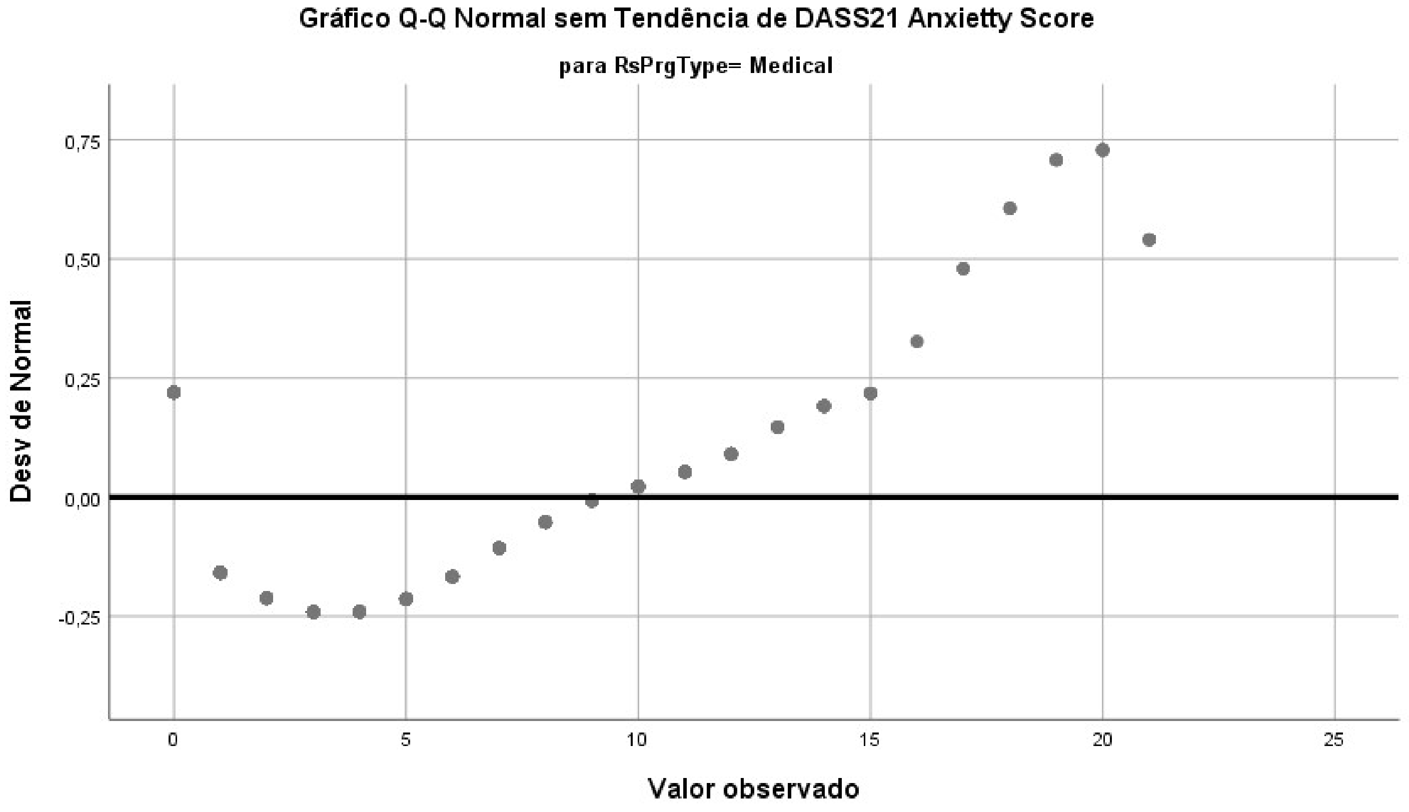
Gráfico Q-Q Normal sem Tendência de DASS21 Anxietty Score
para RsPrgType= Medical
Desv de Normal
Valor observado
0,75
0,50
0,25
0,00
-0,25
0
5
10
15
20
25

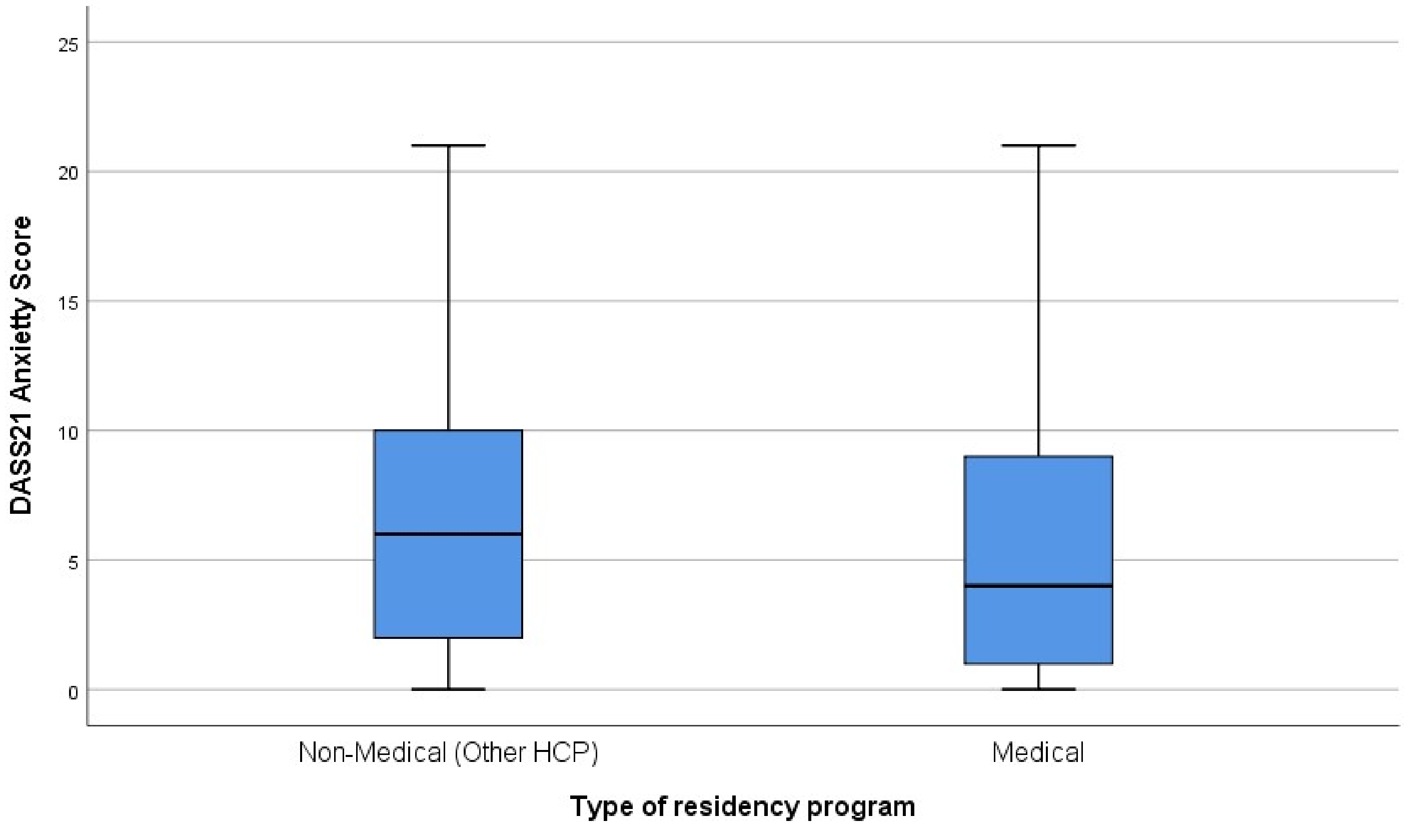

DASS21 Stress Score

Histogramas

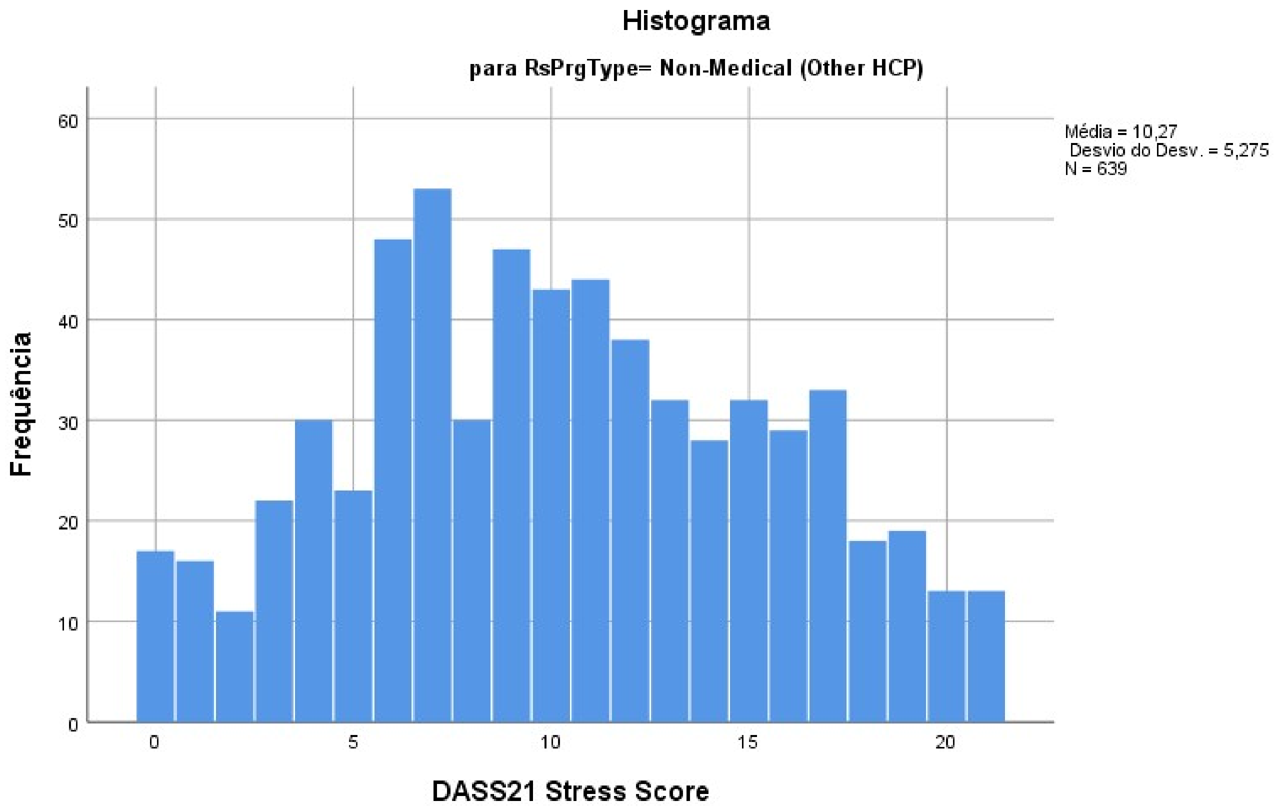

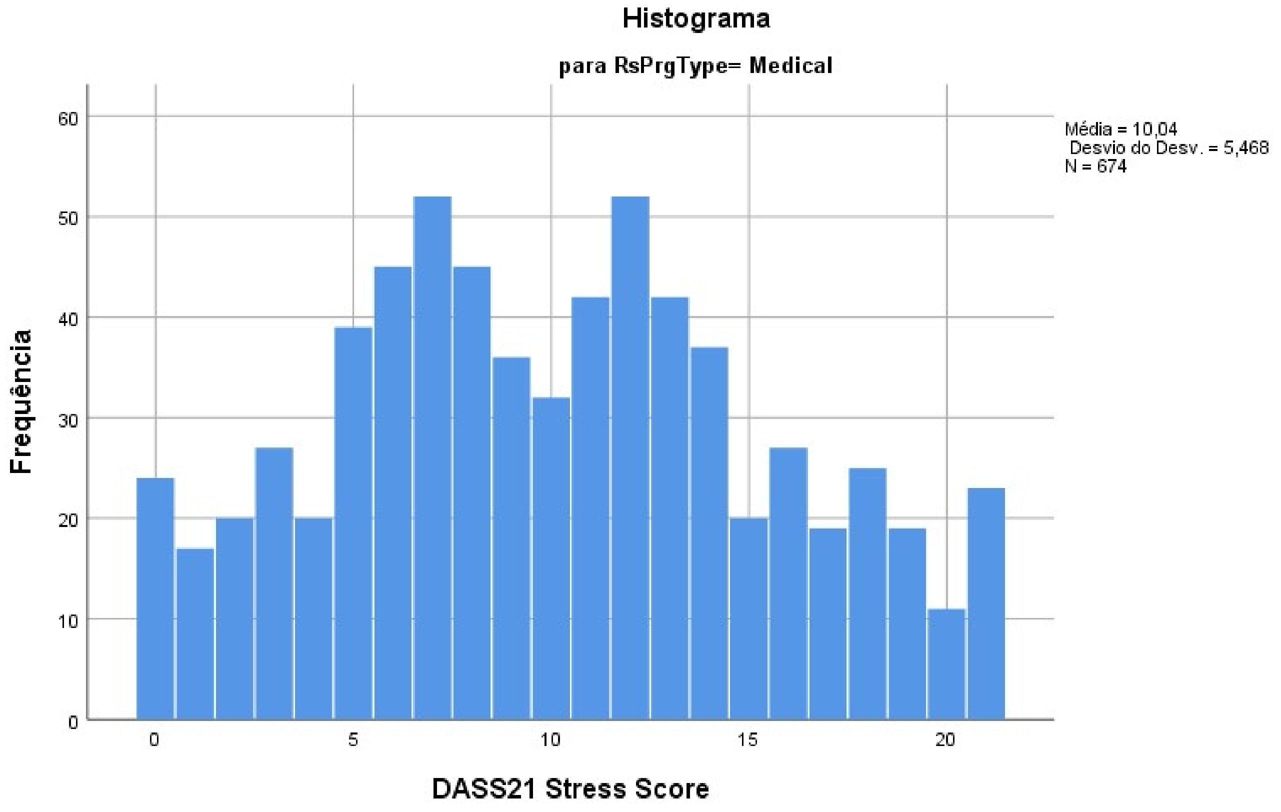

Gráfico Q-Q normais

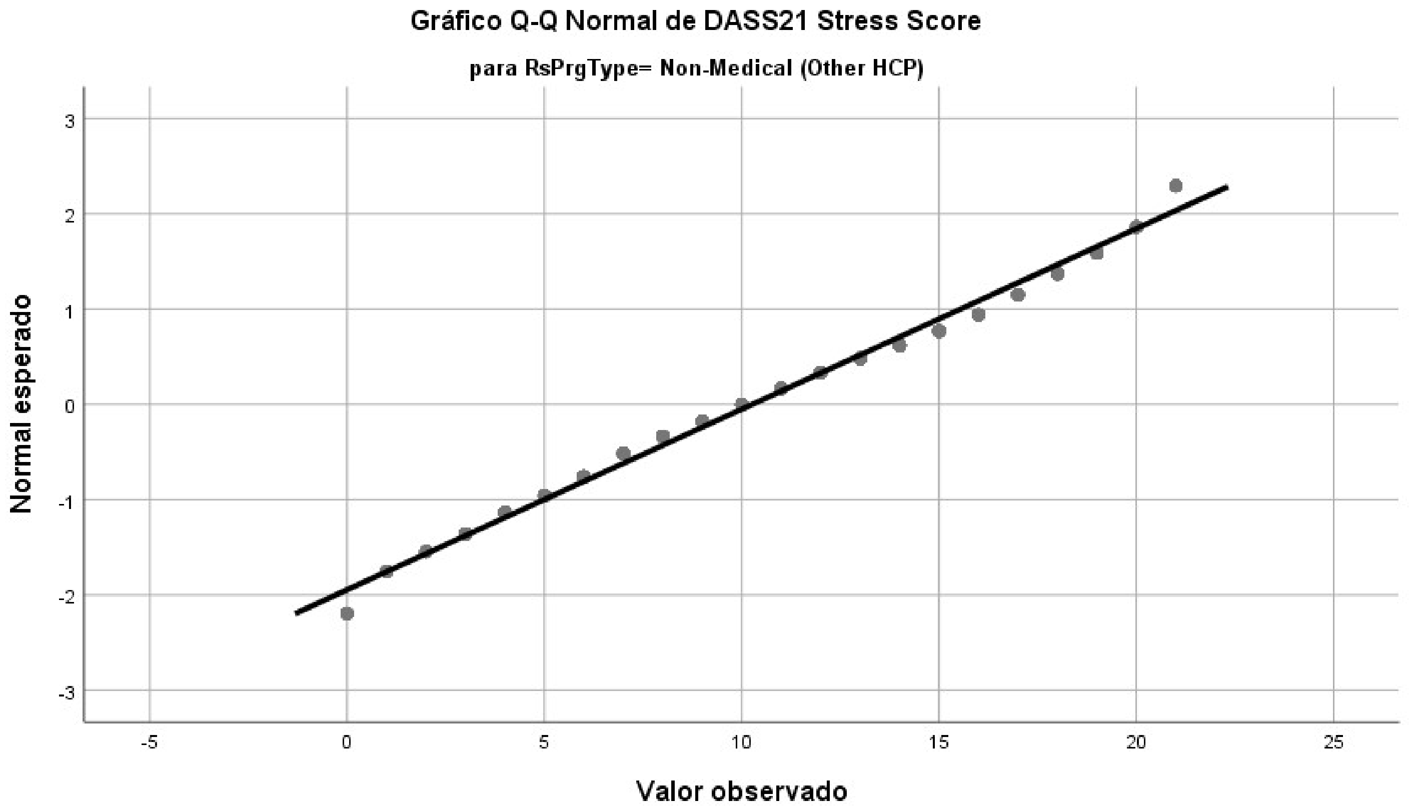

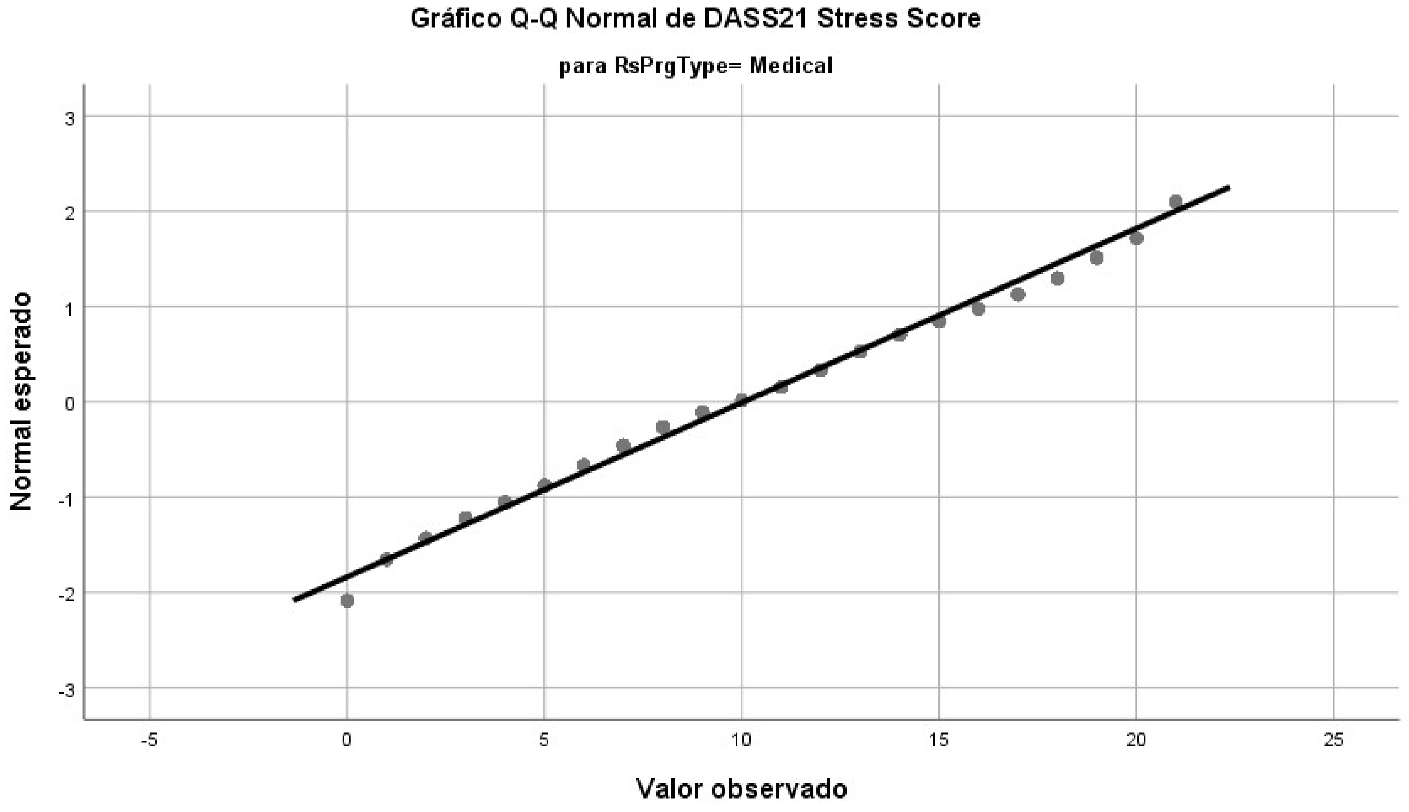

Gráfico Q-Q normais sem tendência

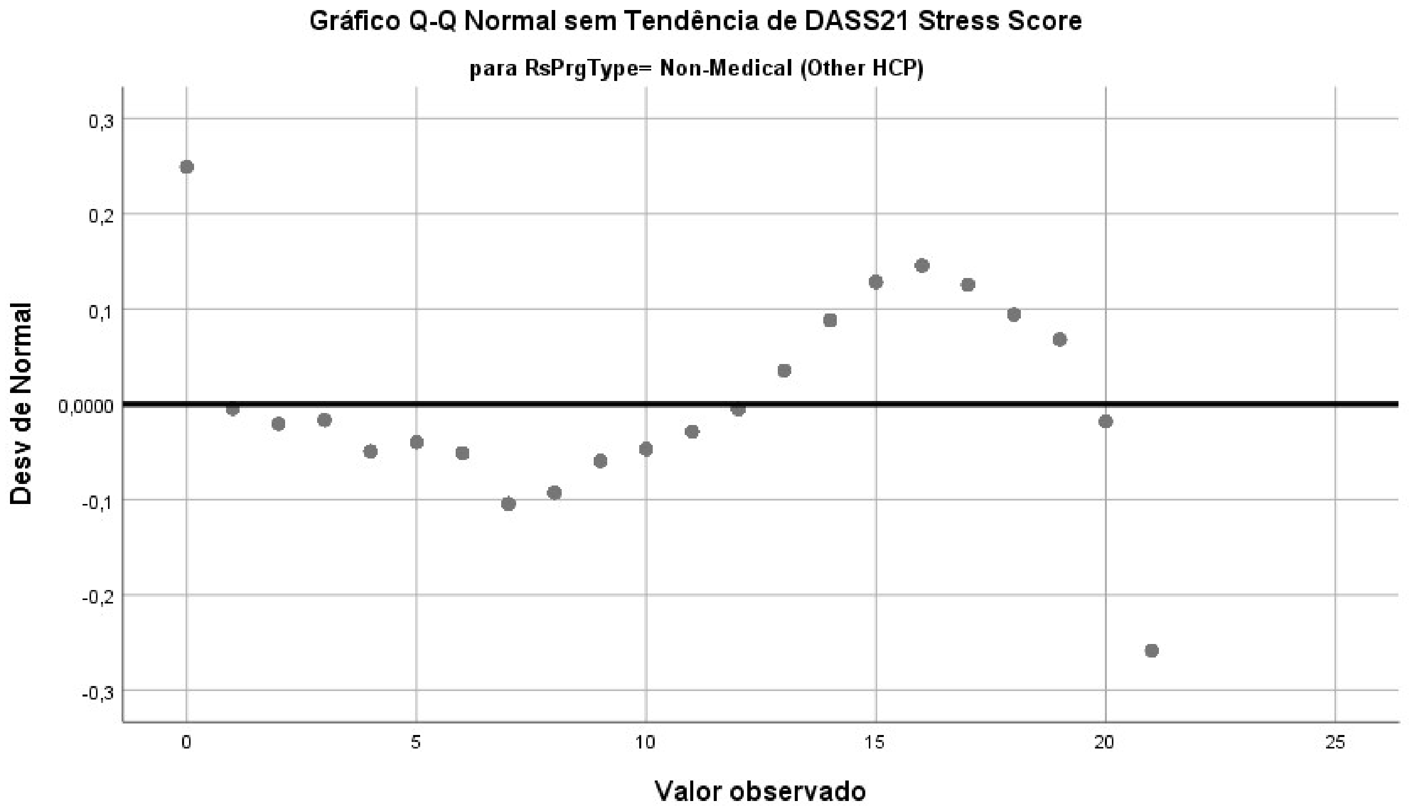

Gráfico Q-Q Normal sem Tendência de DASS21 Stress Score
para RsPrgType= Non-Medical (Other HCP)
Desv de Normal
0,3
0,2
0,1
0,0000
-0,1
-0,2
-0,3
0
5
10
15
20
25
Valor observado

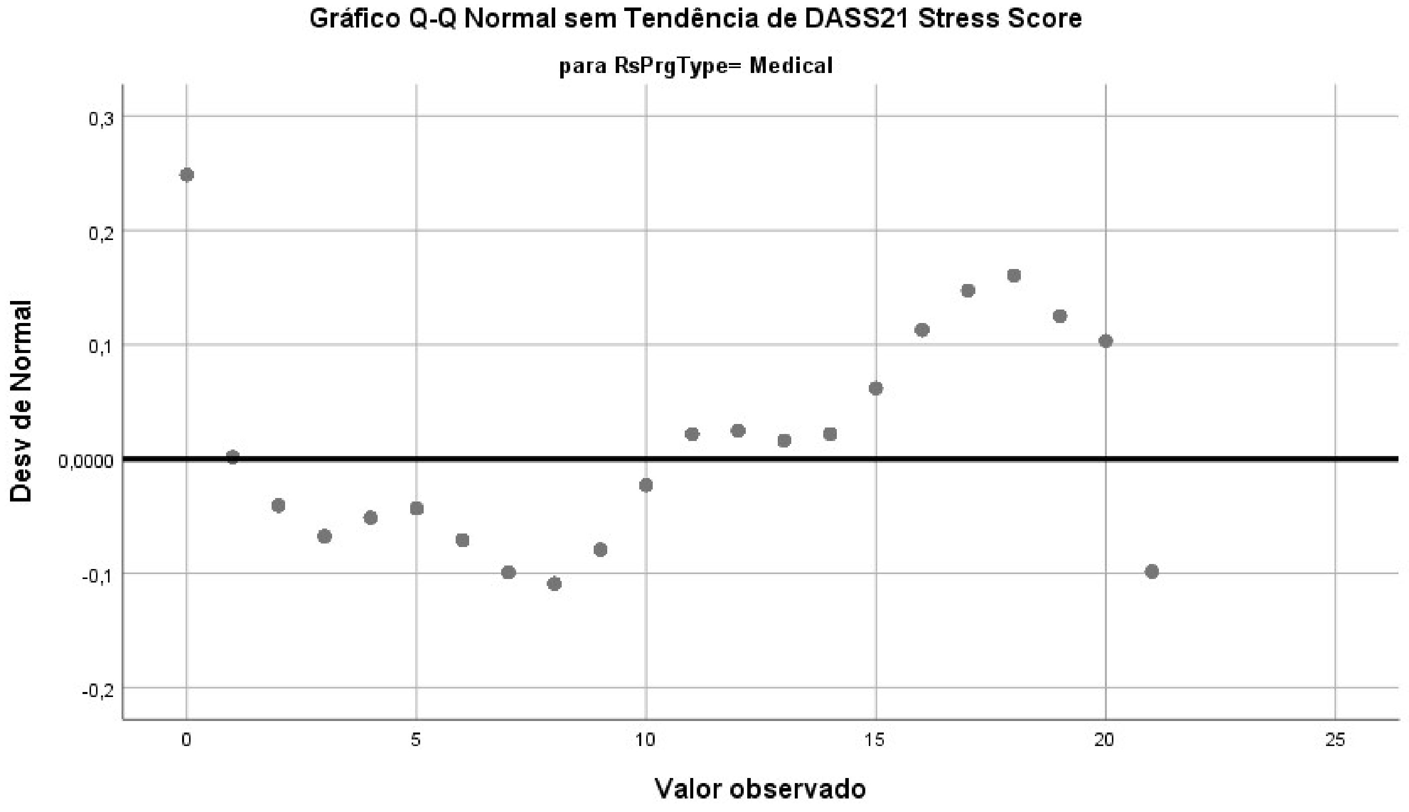

Gráfico Q-Q Normal sem Tendência de DASS21 Stress Score
para RsPrgType= Medical
Desv de Normal
0,3
0,2
0,1
0,0000
-0,1
-0,2
0
5
10
15
20
25
Valor observado

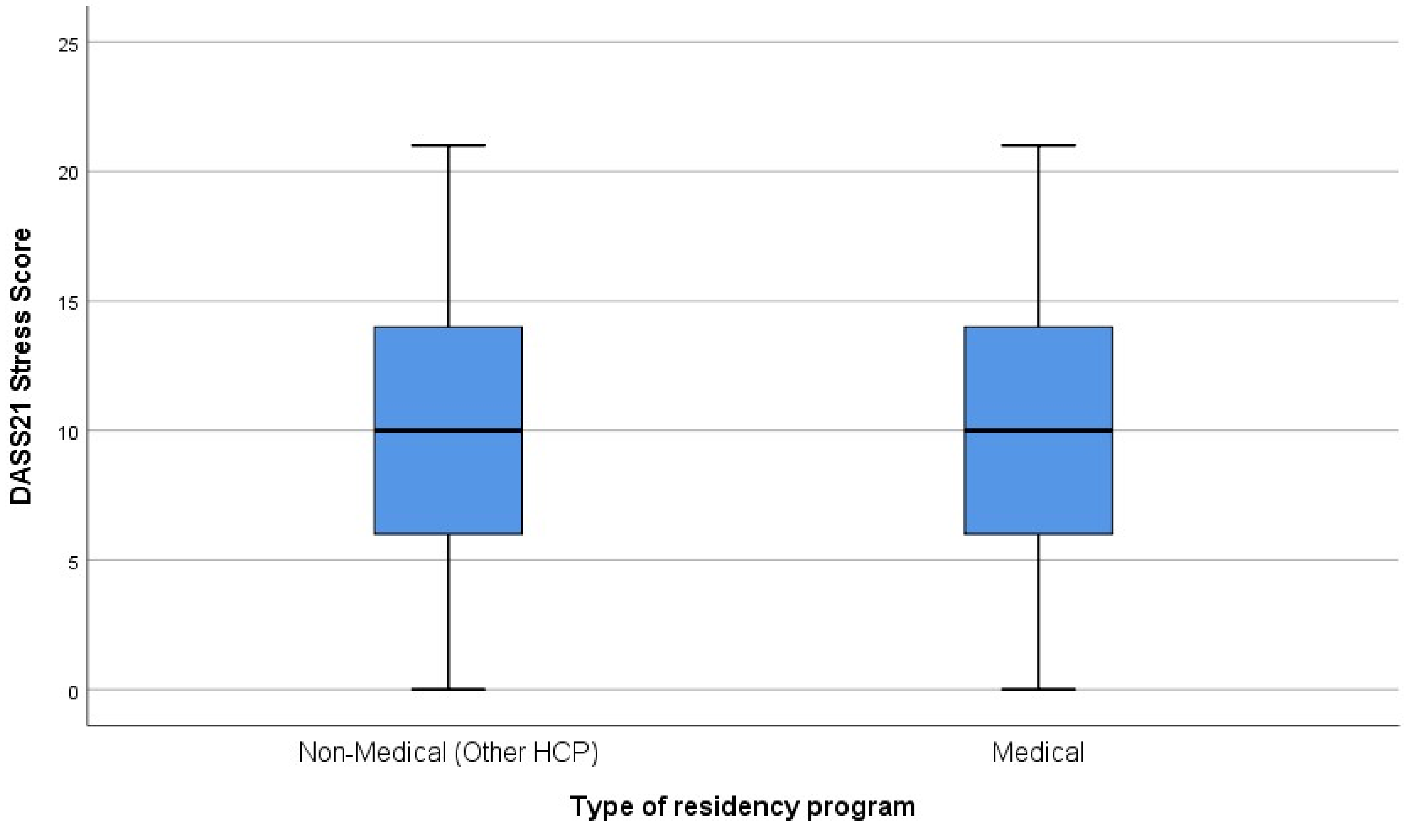

PHQ9 Depression Score

Histogramas

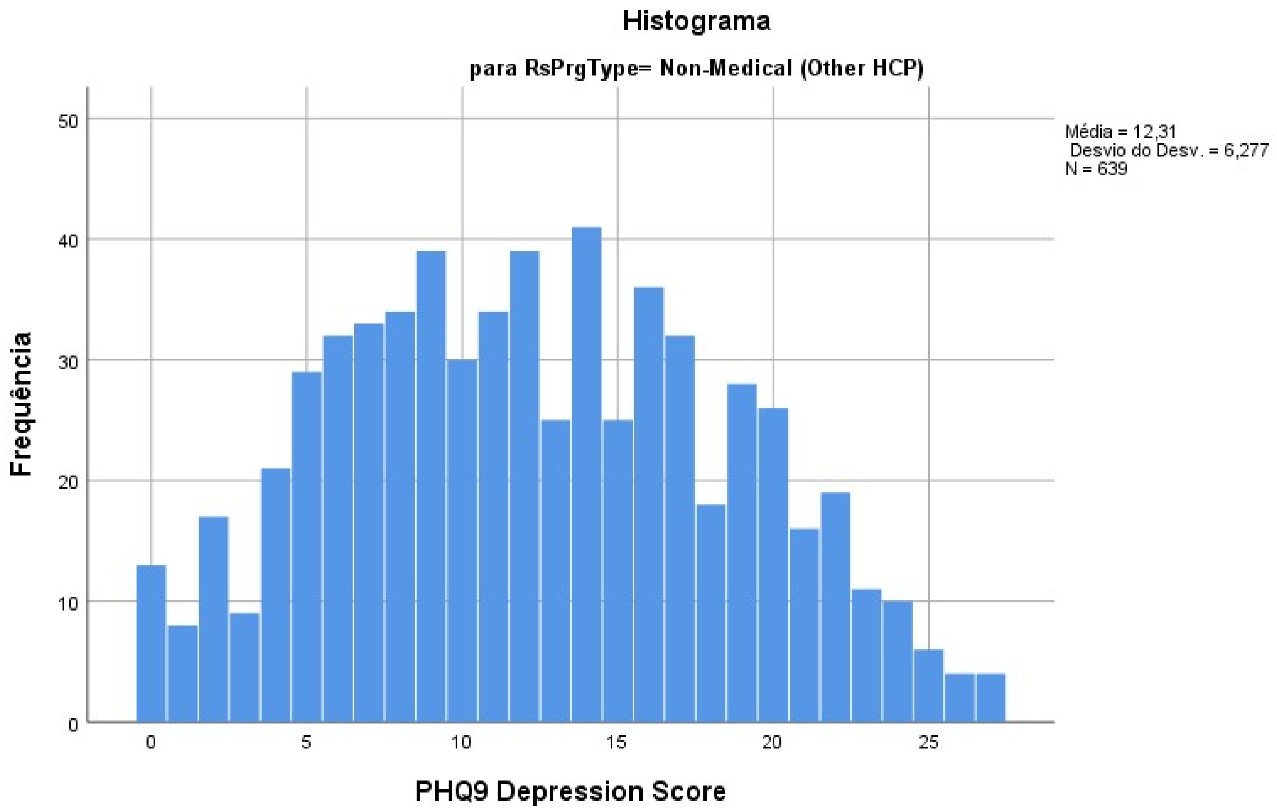

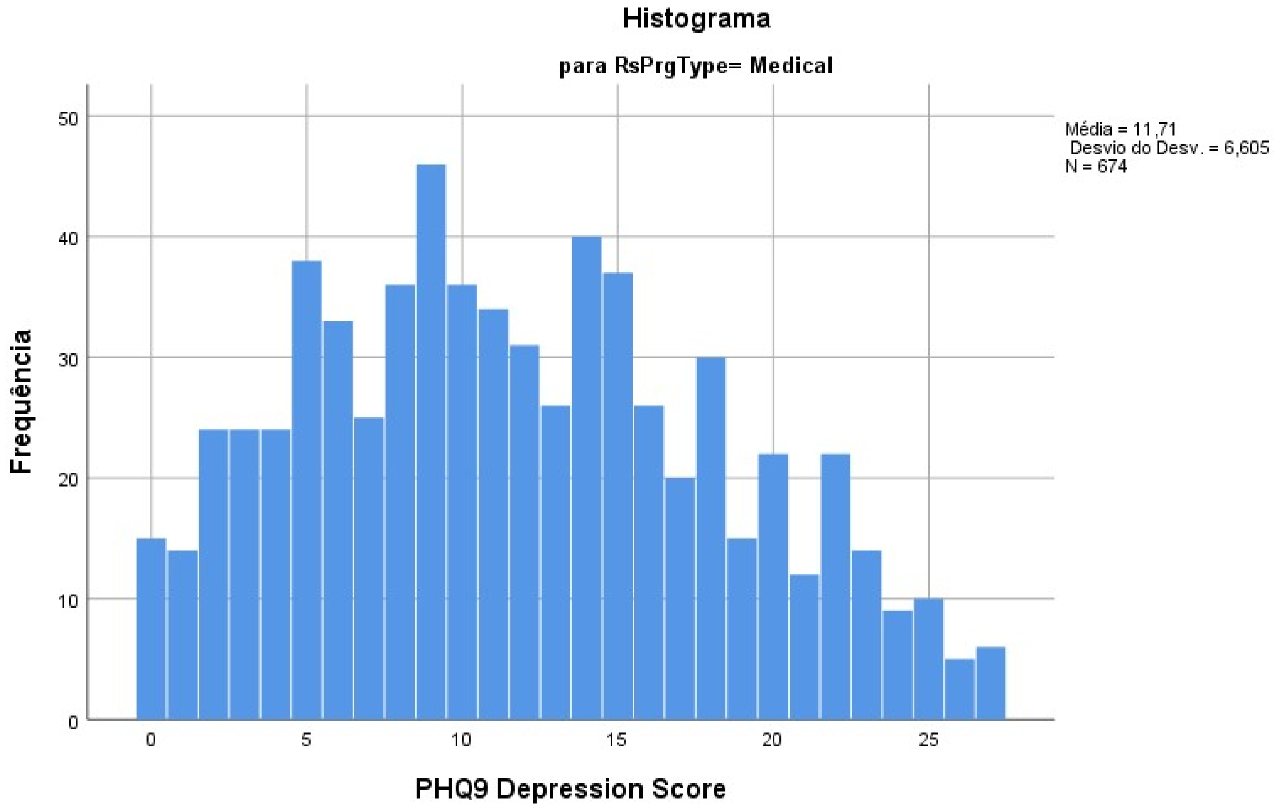

Gráfico Q-Q normais

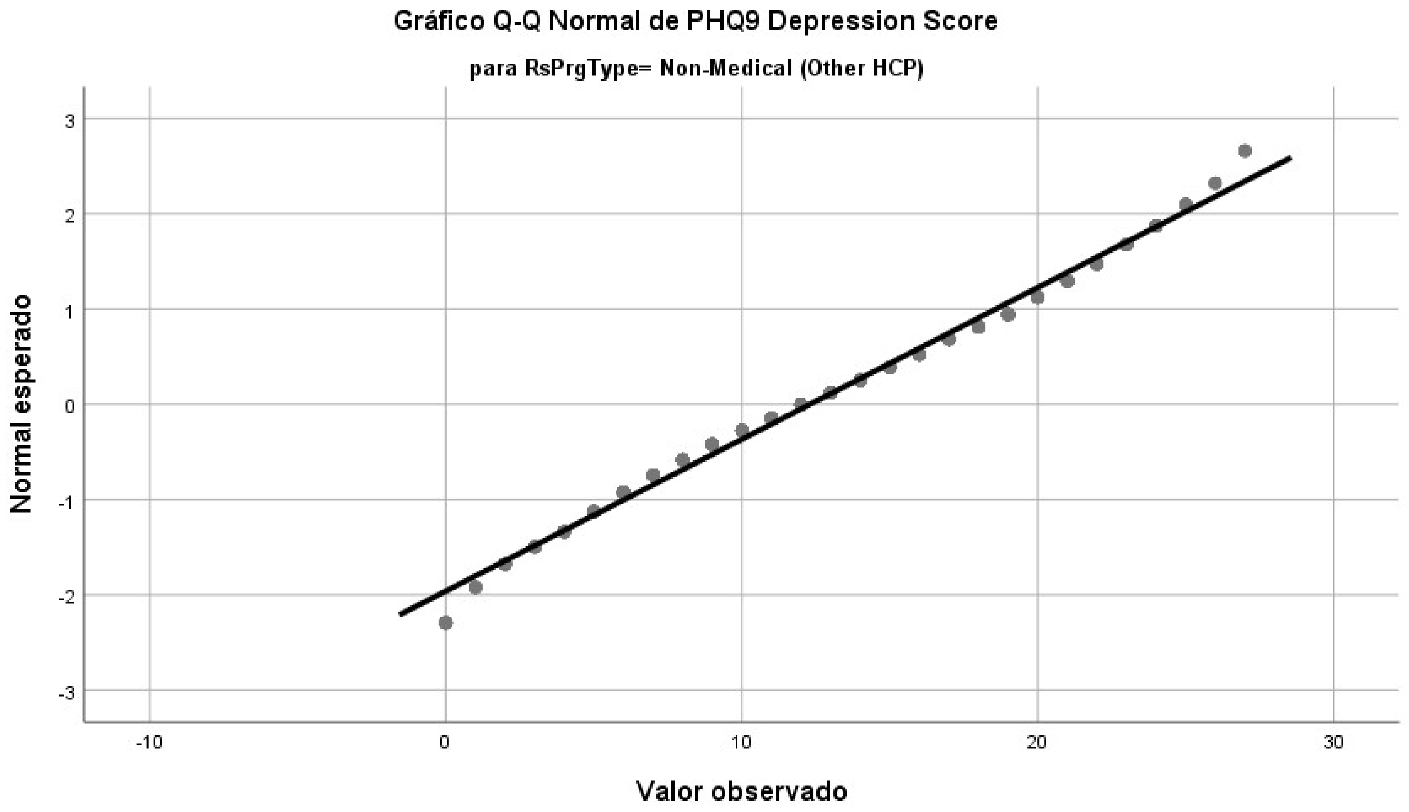

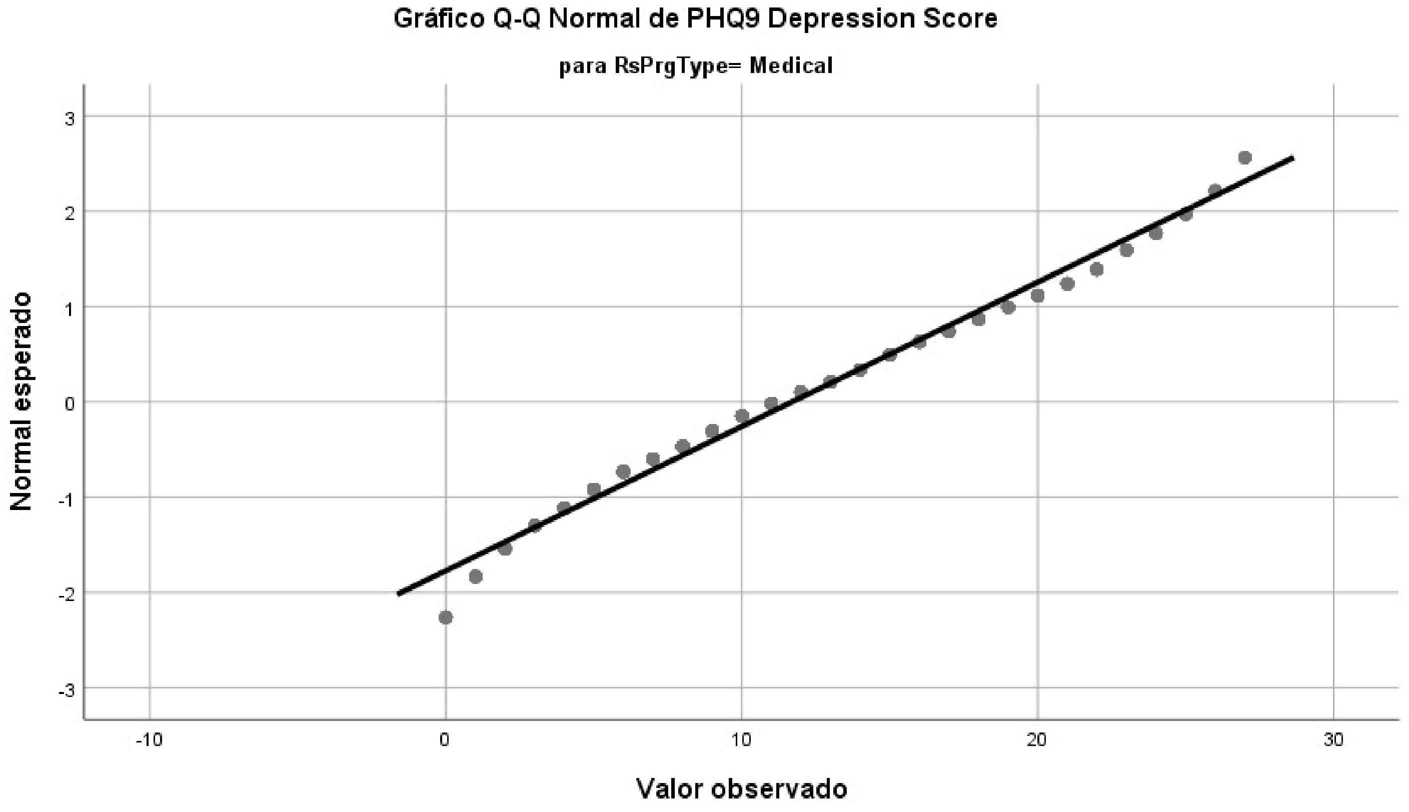

Gráfico Q-Q normais sem tendência

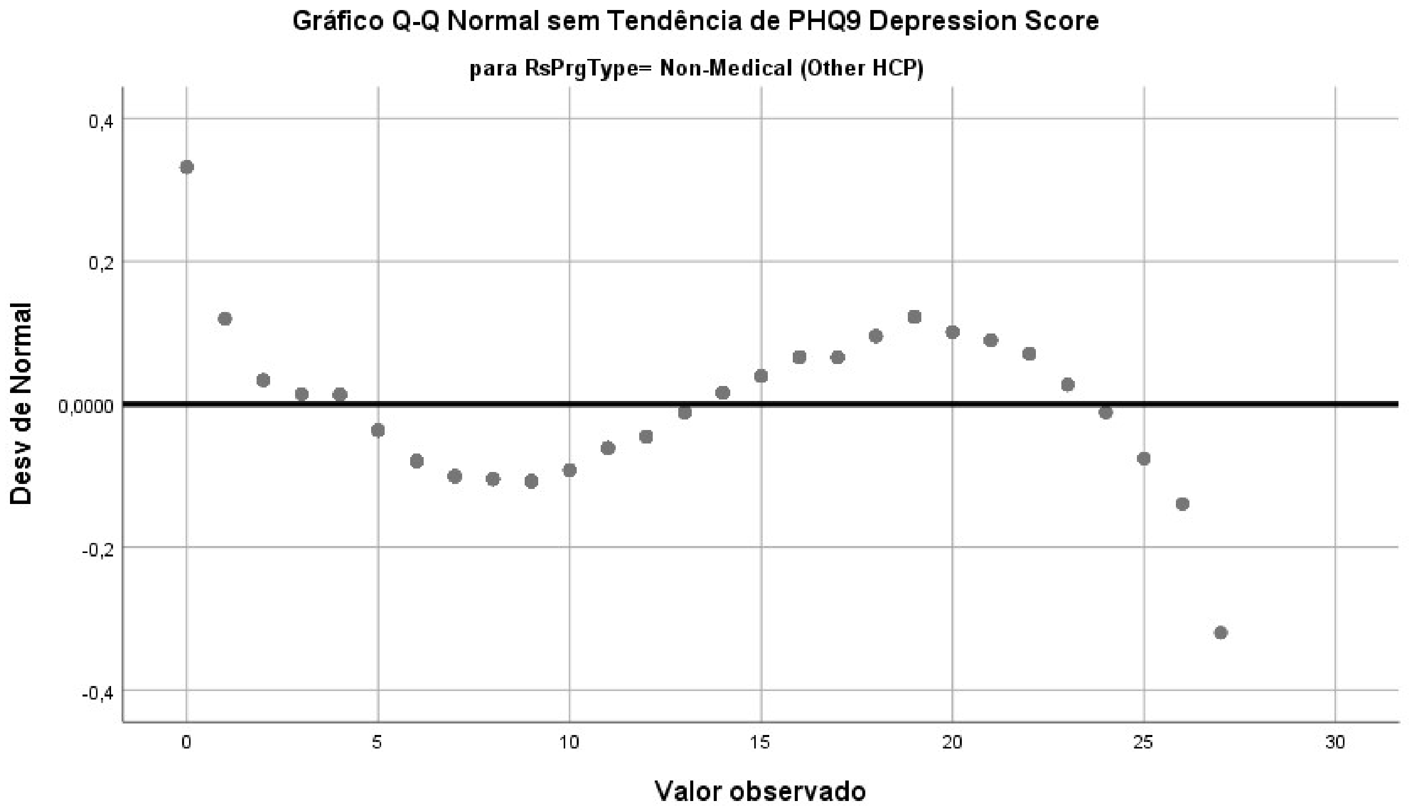
Gráfico Q-Q Normal sem Tendência de PHQ9 Depression Score
para RsPrgType= Non-Medical (Other HCP)
Desv de Normal
0,4
0,2
0,0000
-0,2
-0,4
0
5
10
15
20
25
30
Valor observado

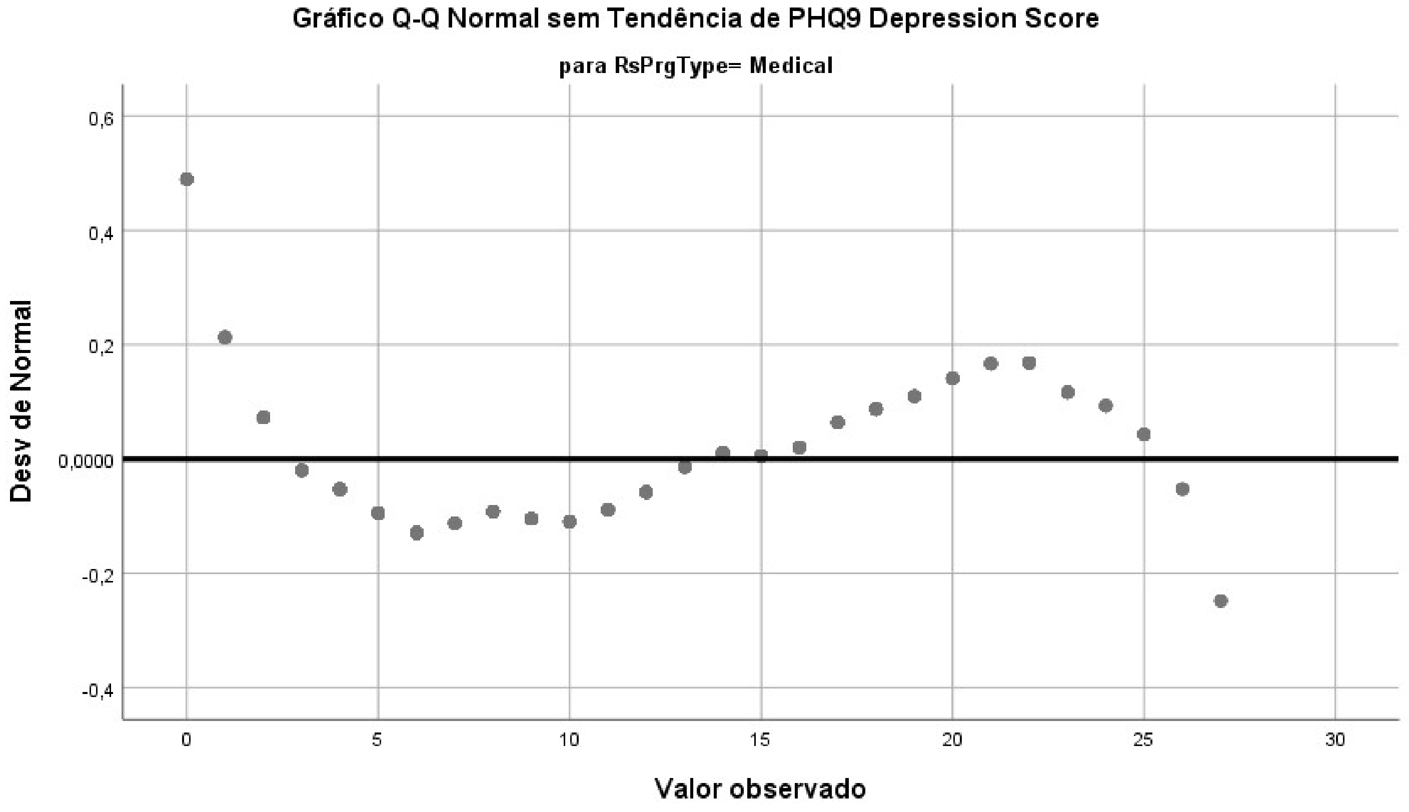
Gráfico Q-Q Normal sem Tendência de PHQ9 Depression Score
para RsPrgType= Medical
Desv de Normal
0,6
0,4
0,2
0,0000
-0,2
-0,4
0
5
10
15
20
25
30
Valor observado

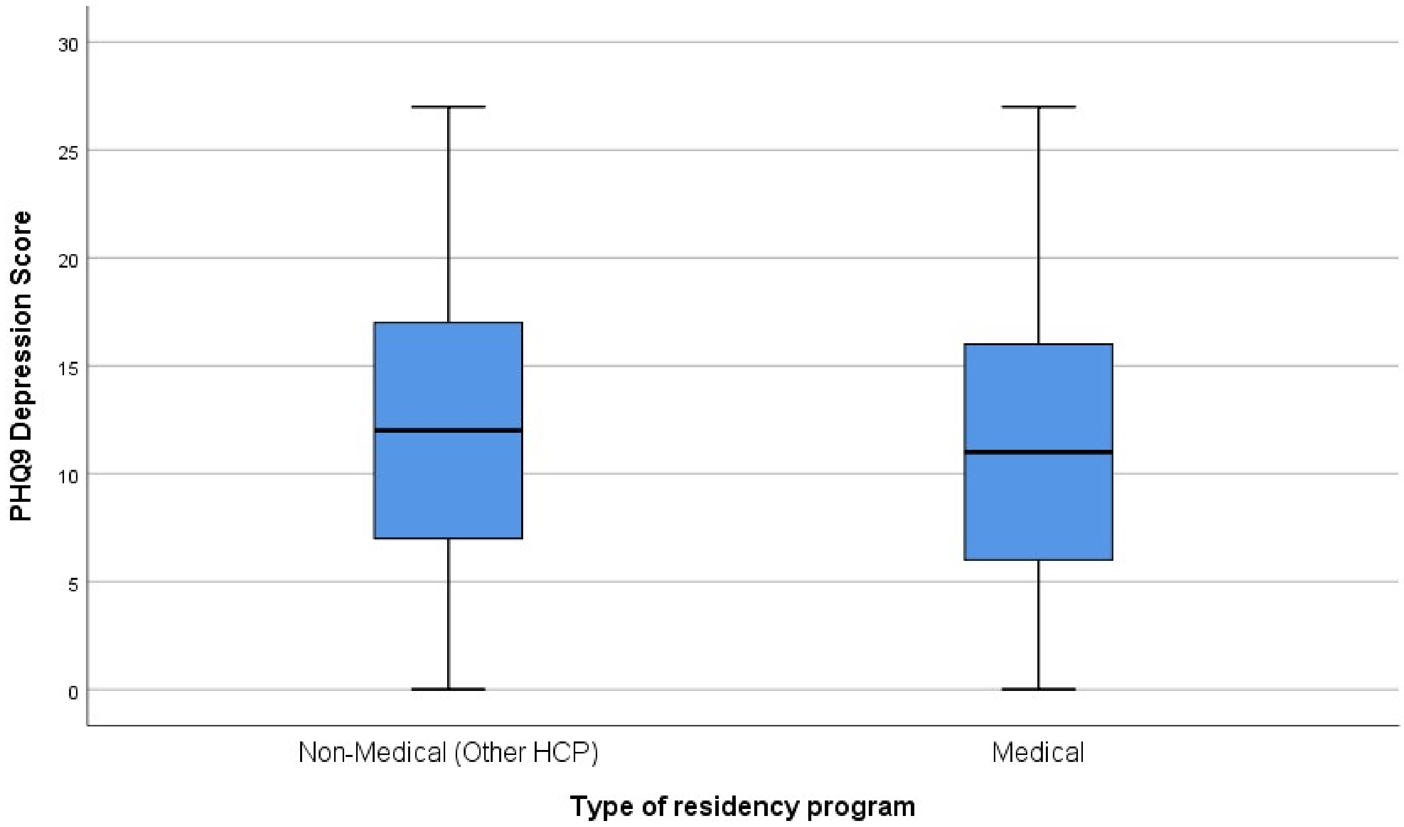

BRCS Score

Histogramas

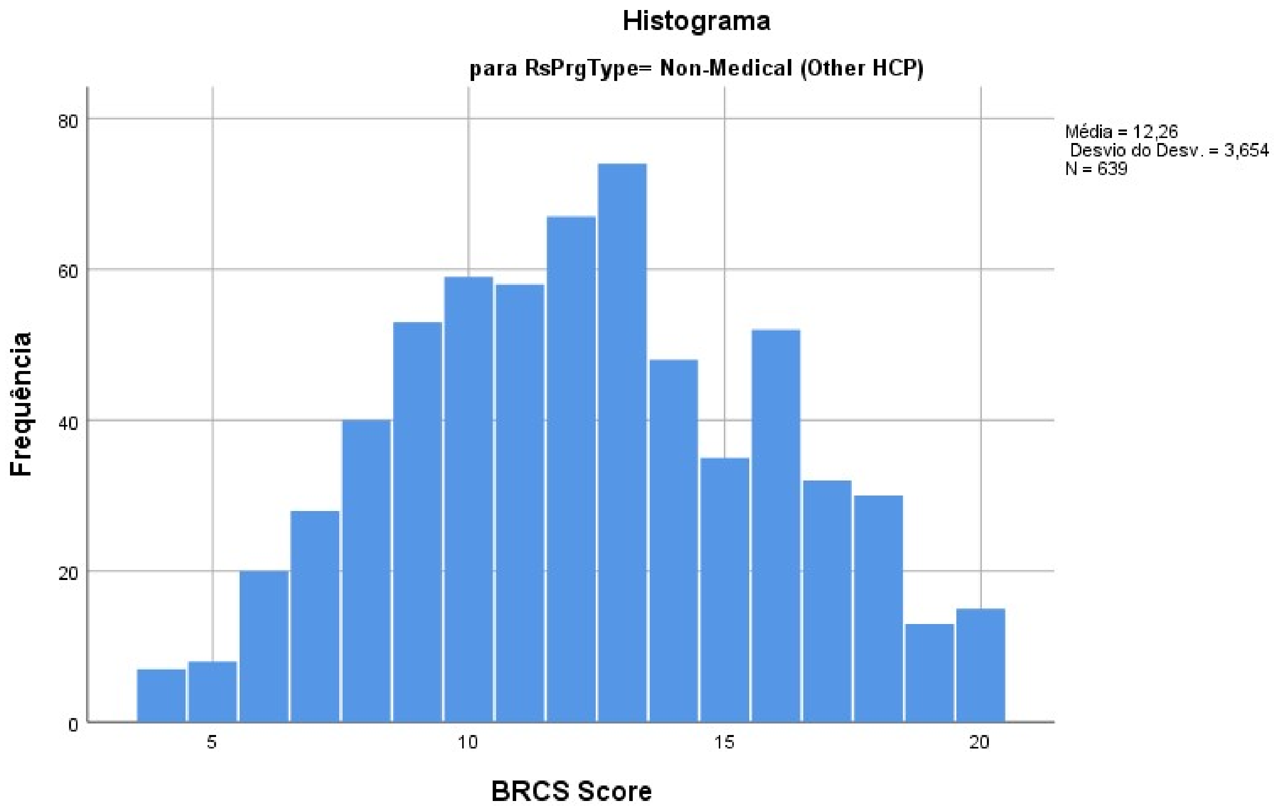

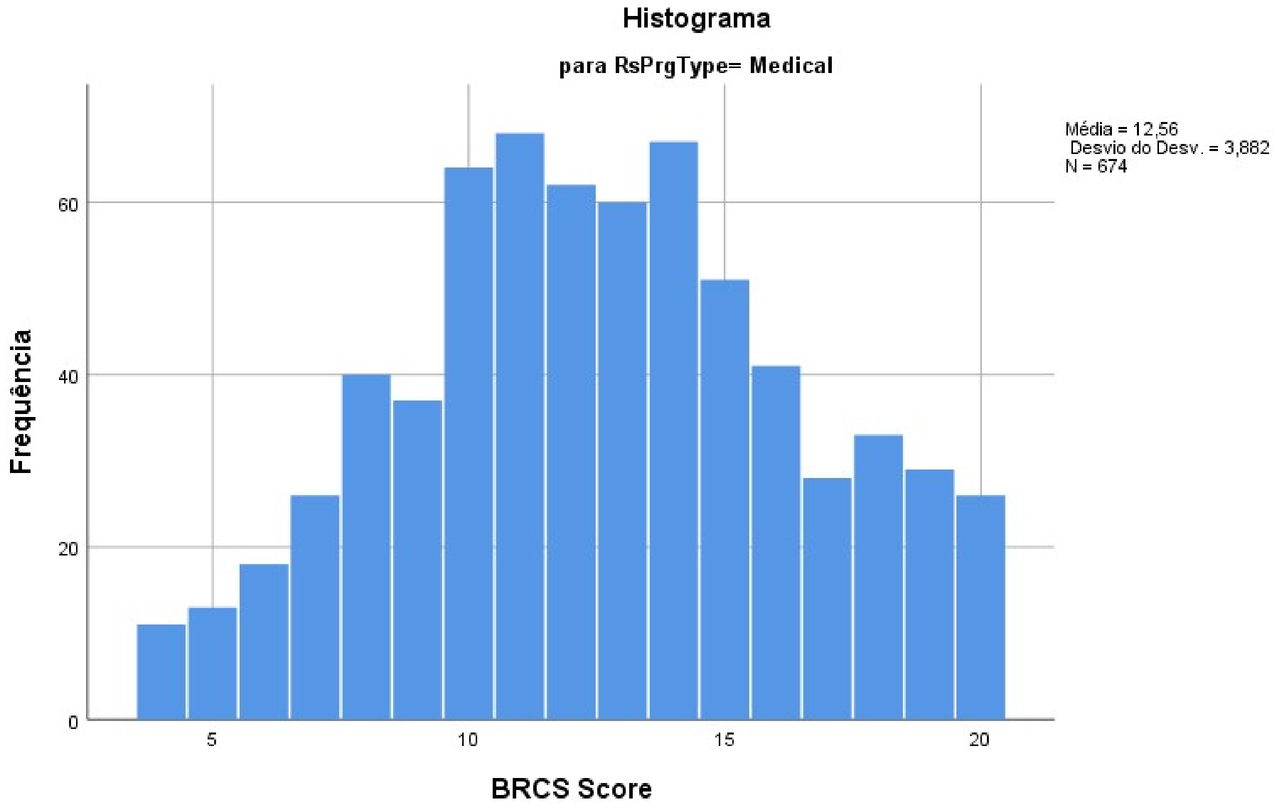

Gráfico Q-Q normais

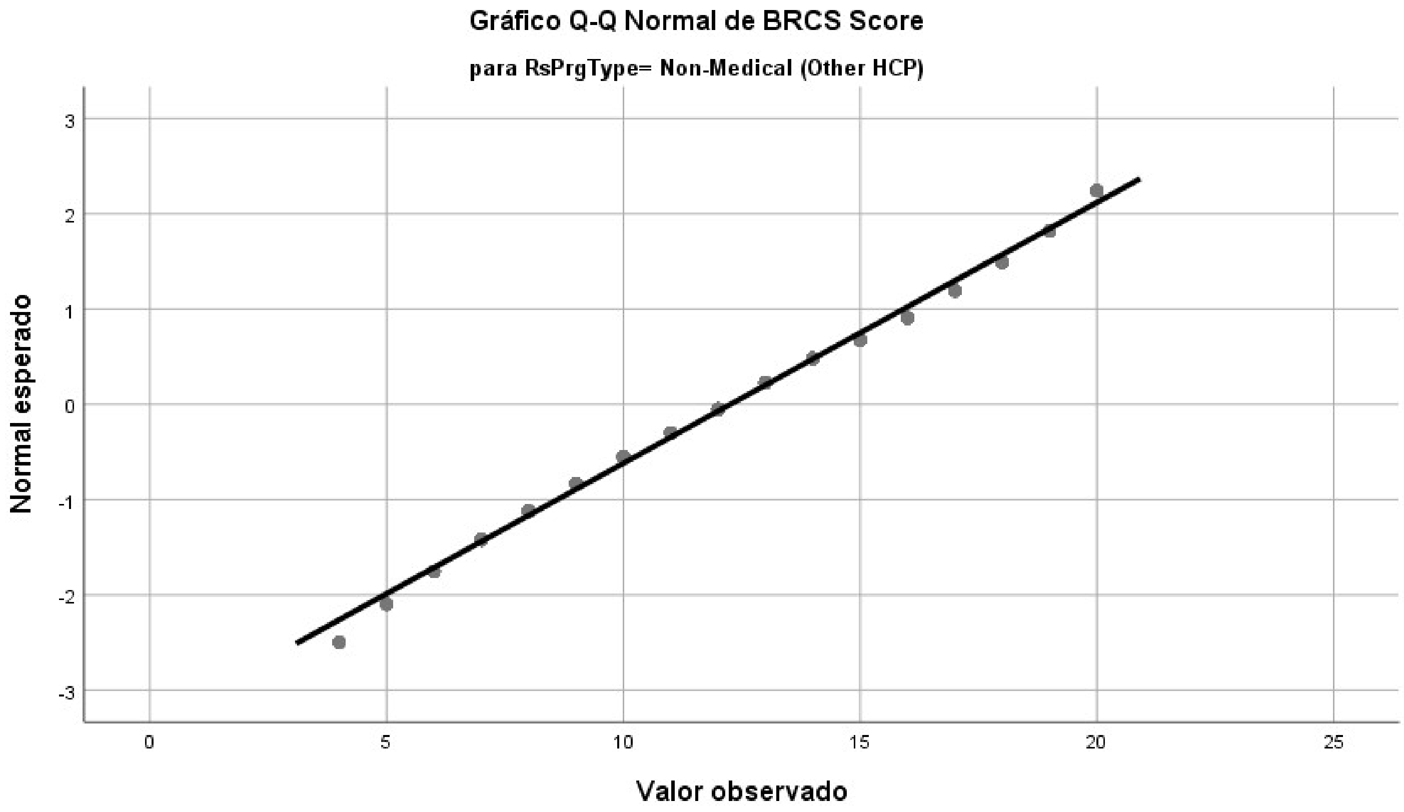

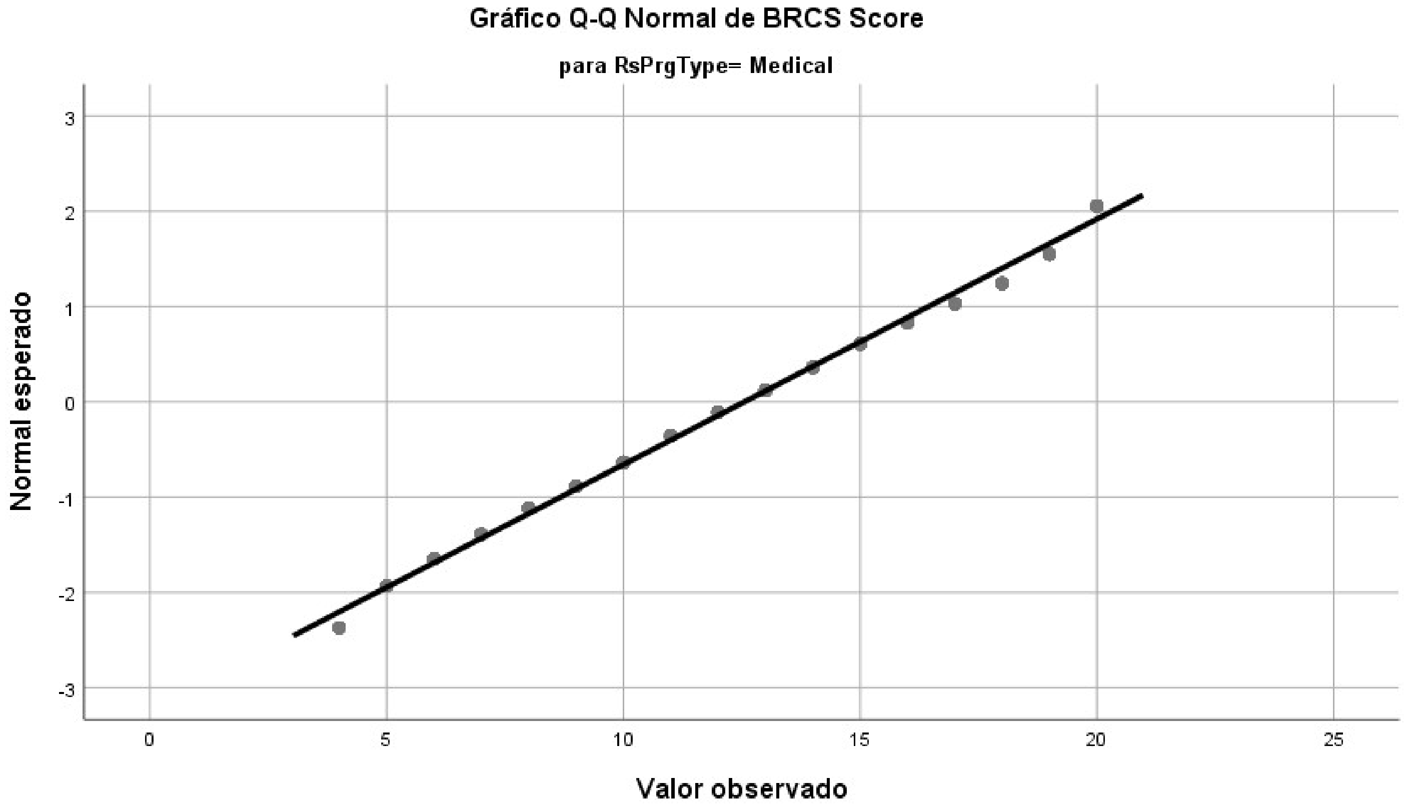

Gráfico Q-Q normais sem tendência

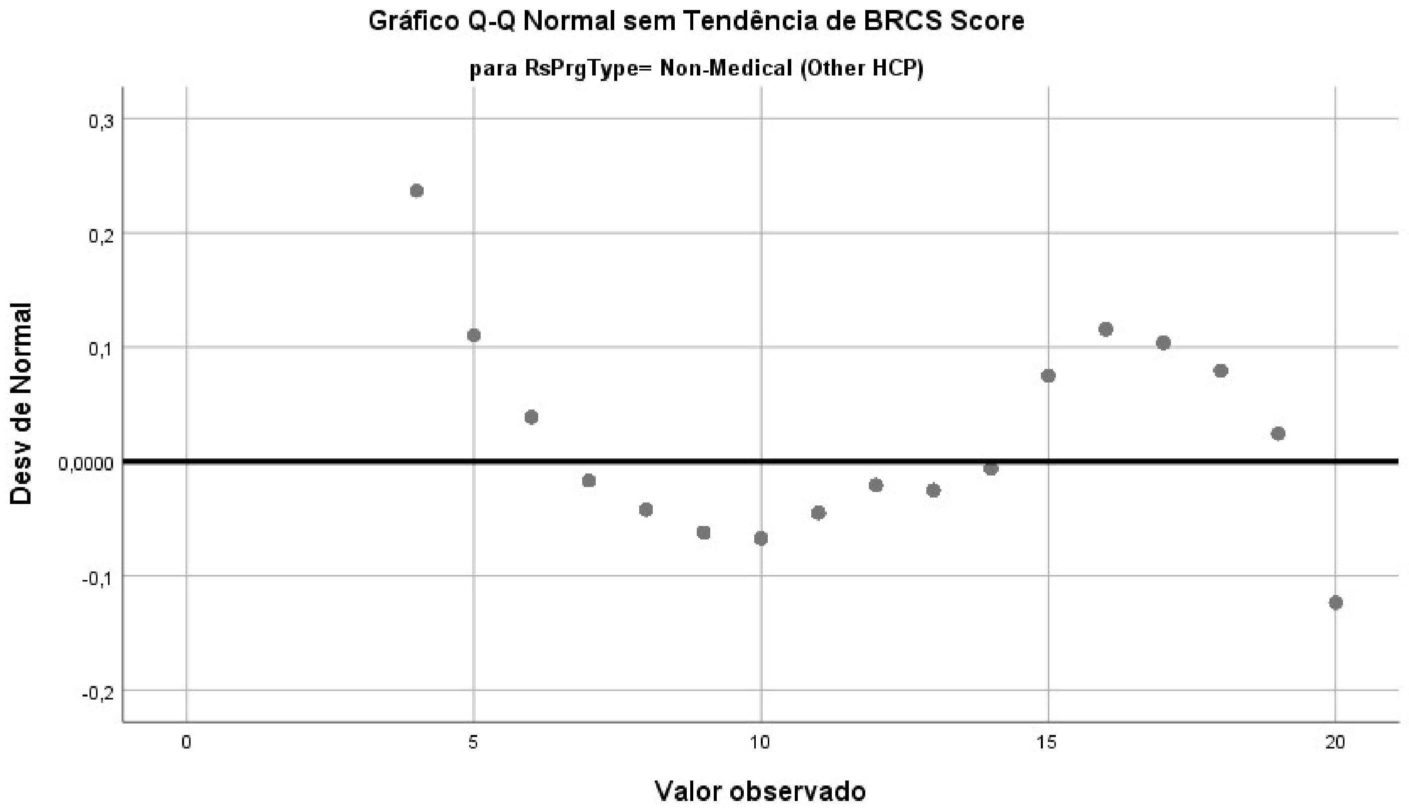
Gráfico Q-Q Normal sem Tendência de BRCS Score
para RsPrgType= Non-Medical (Other HCP)
Desv de Normal
0,3
0,2
0,1
0,0000
-0,1
-0,2
0
5
10
15
20
Valor observado

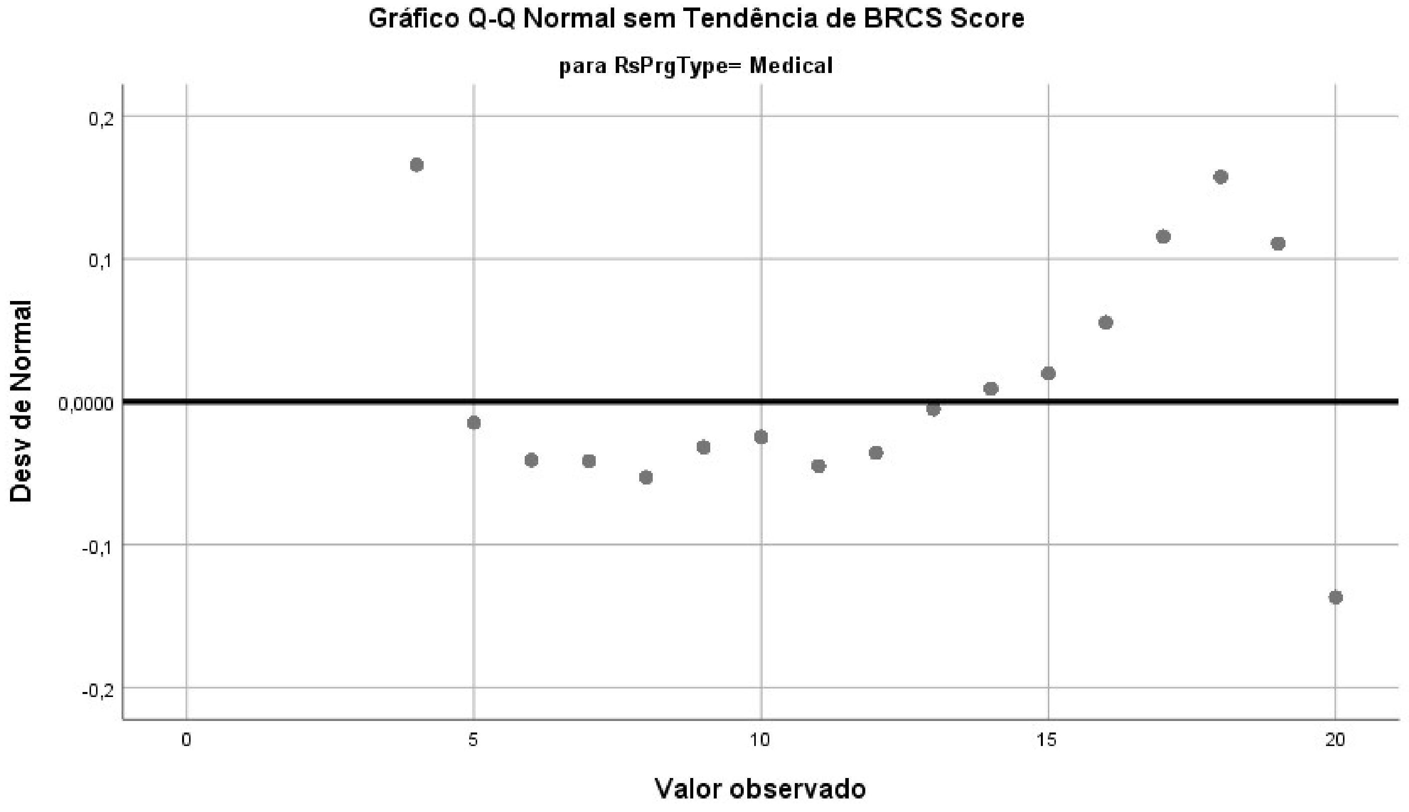
Gráfico Q-Q Normal sem Tendência de BRCS Score
para RsPrgType= Medical
Desv de Normal
0,2
0,1
0,0000
-0,1
-0,2
0
5
10
15
20
Valor observado

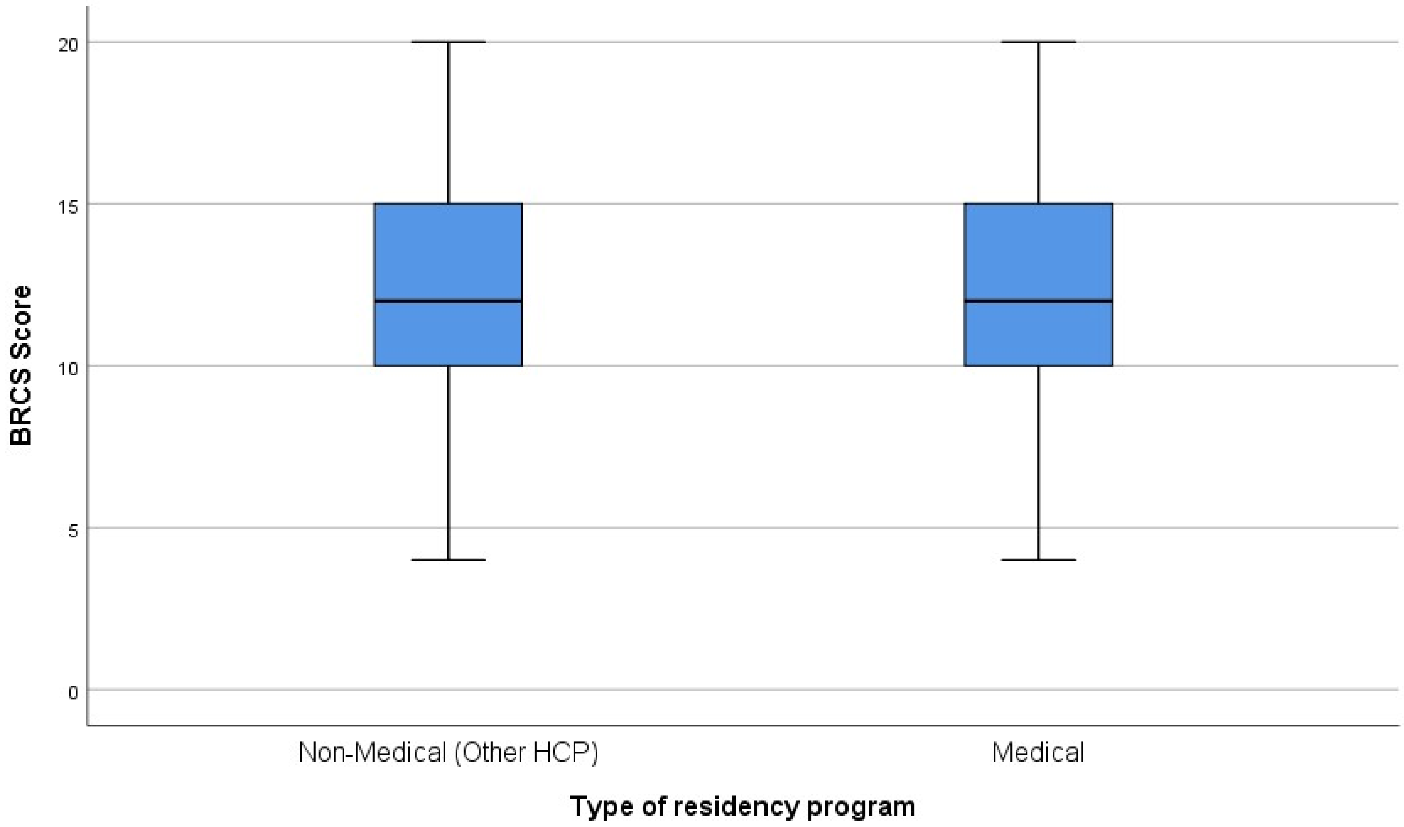

OLBI Disengagement Score

Histogramas

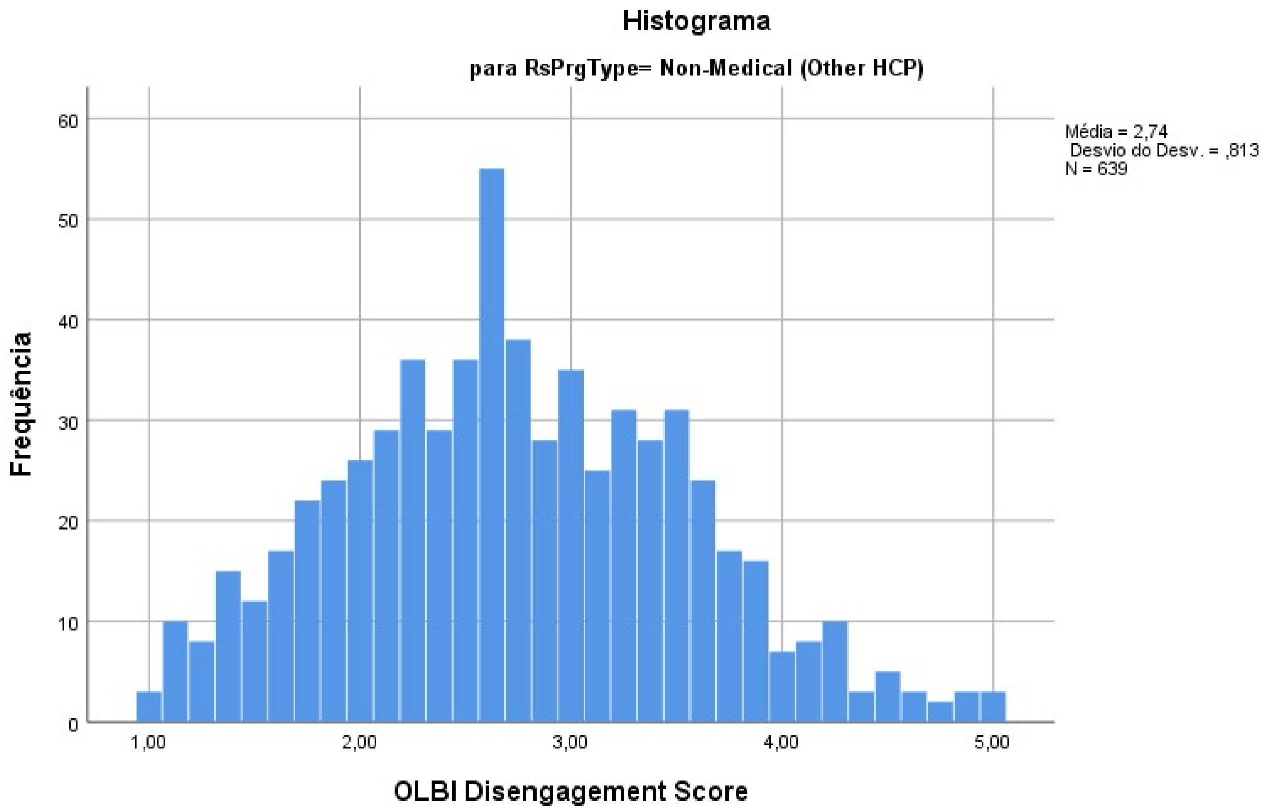

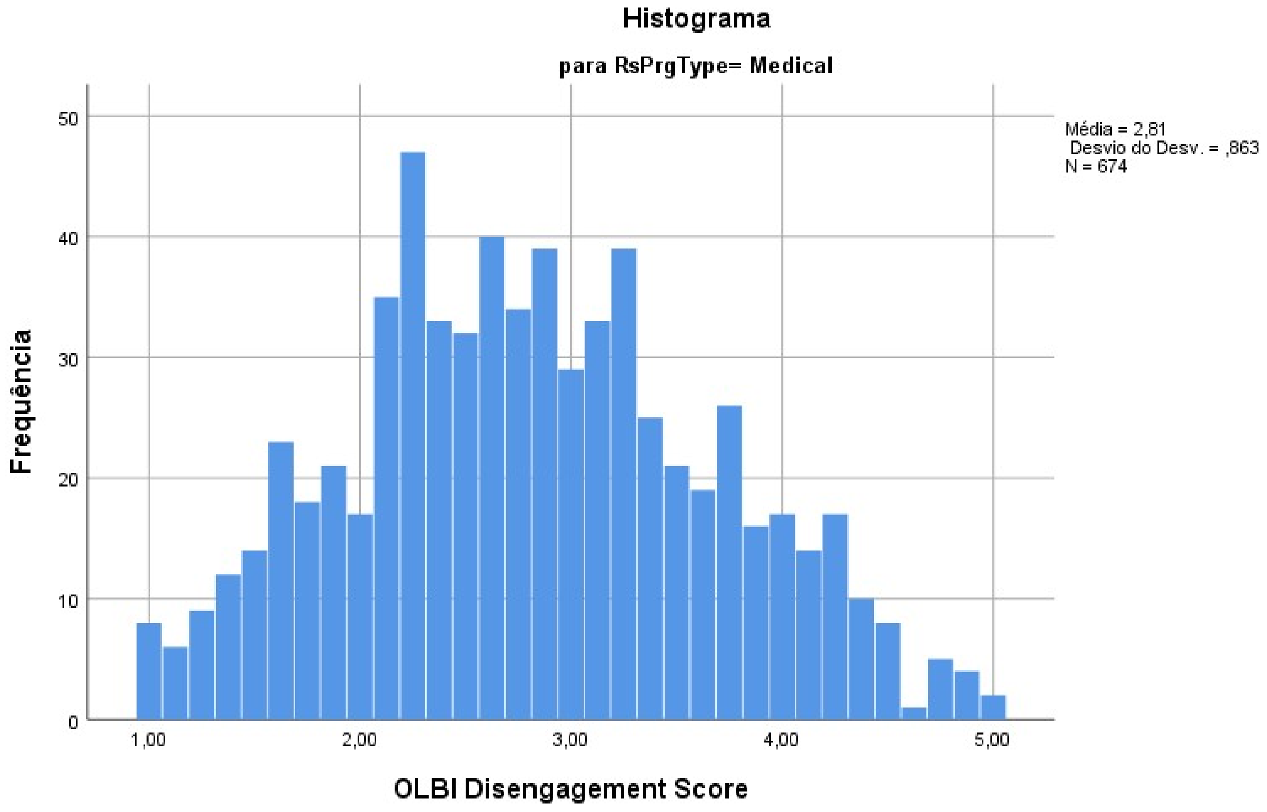

Gráfico Q-Q normais

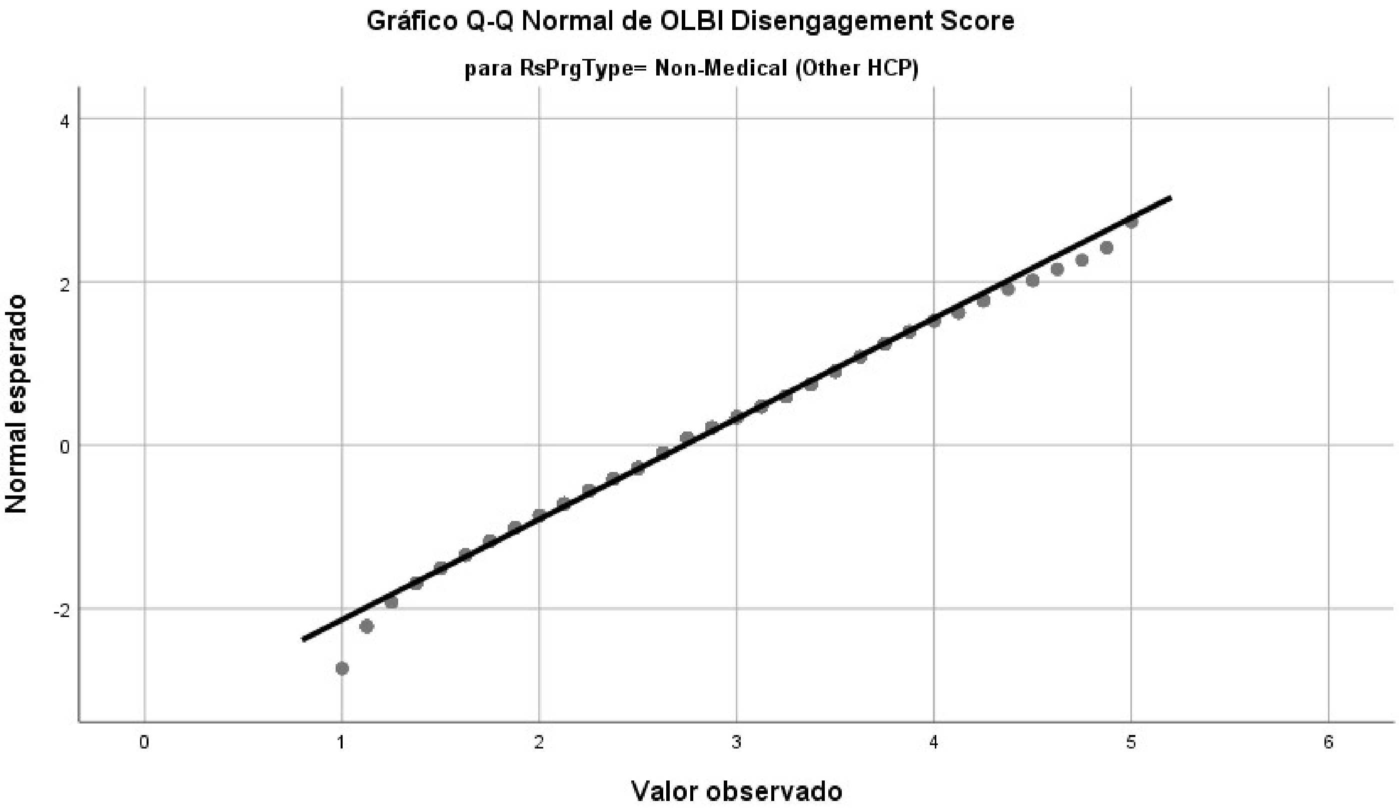

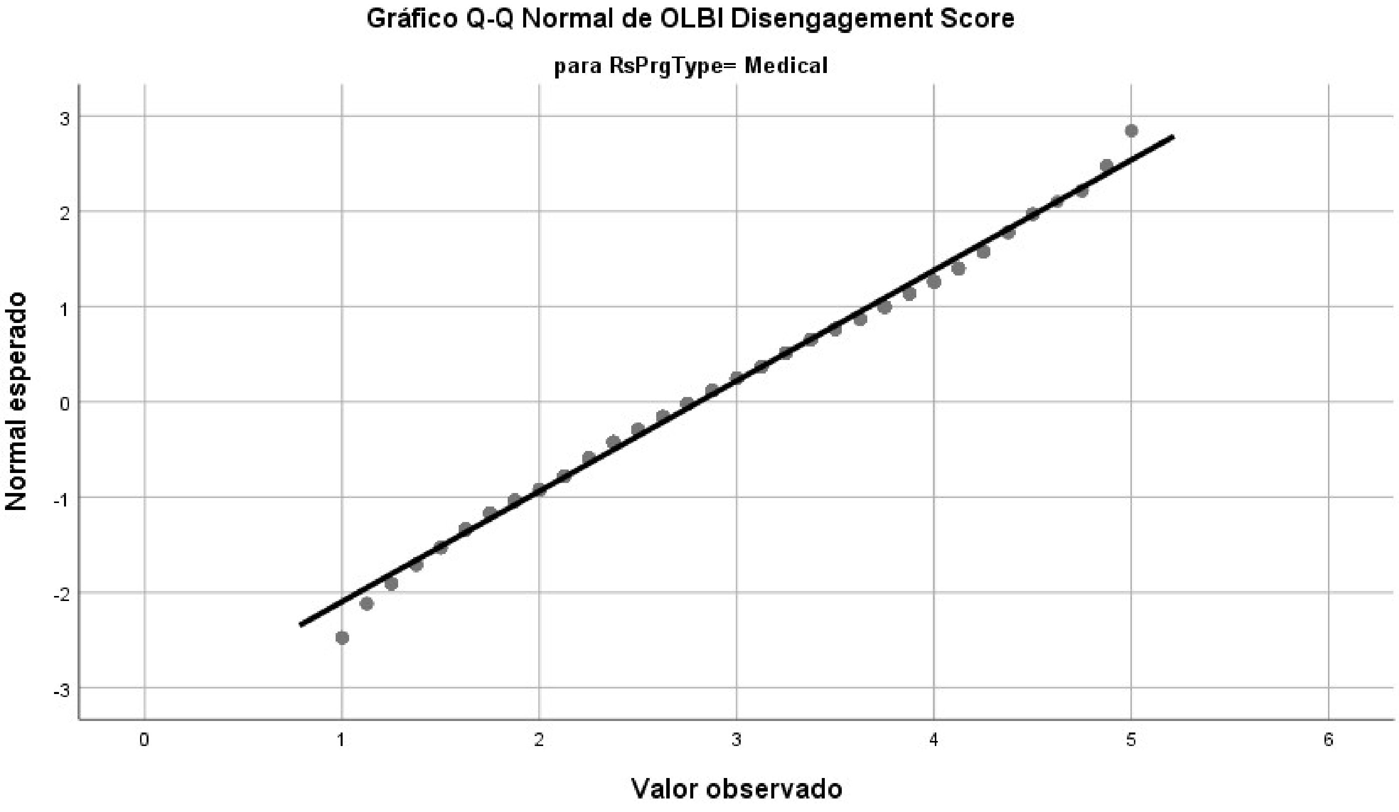

Gráfico Q-Q normais sem tendência

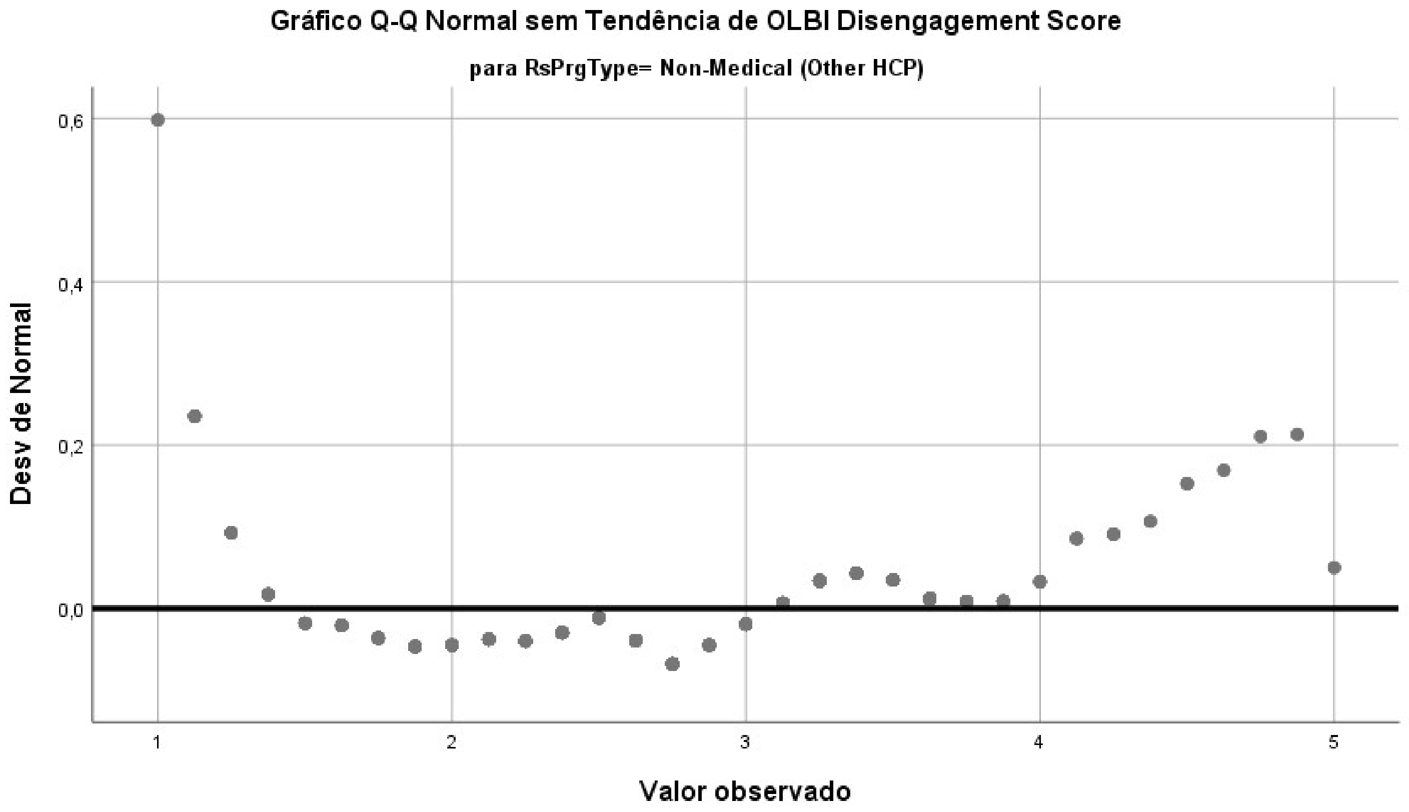

Gráfico Q-Q Normal sem Tendência de OLBI Disengagement Score
para RsPrgType= Non-Medical (Other HCP)
Desv de Normal
0,6
0,4
0,2
0,0
1
2
3
4
5
Valor observado

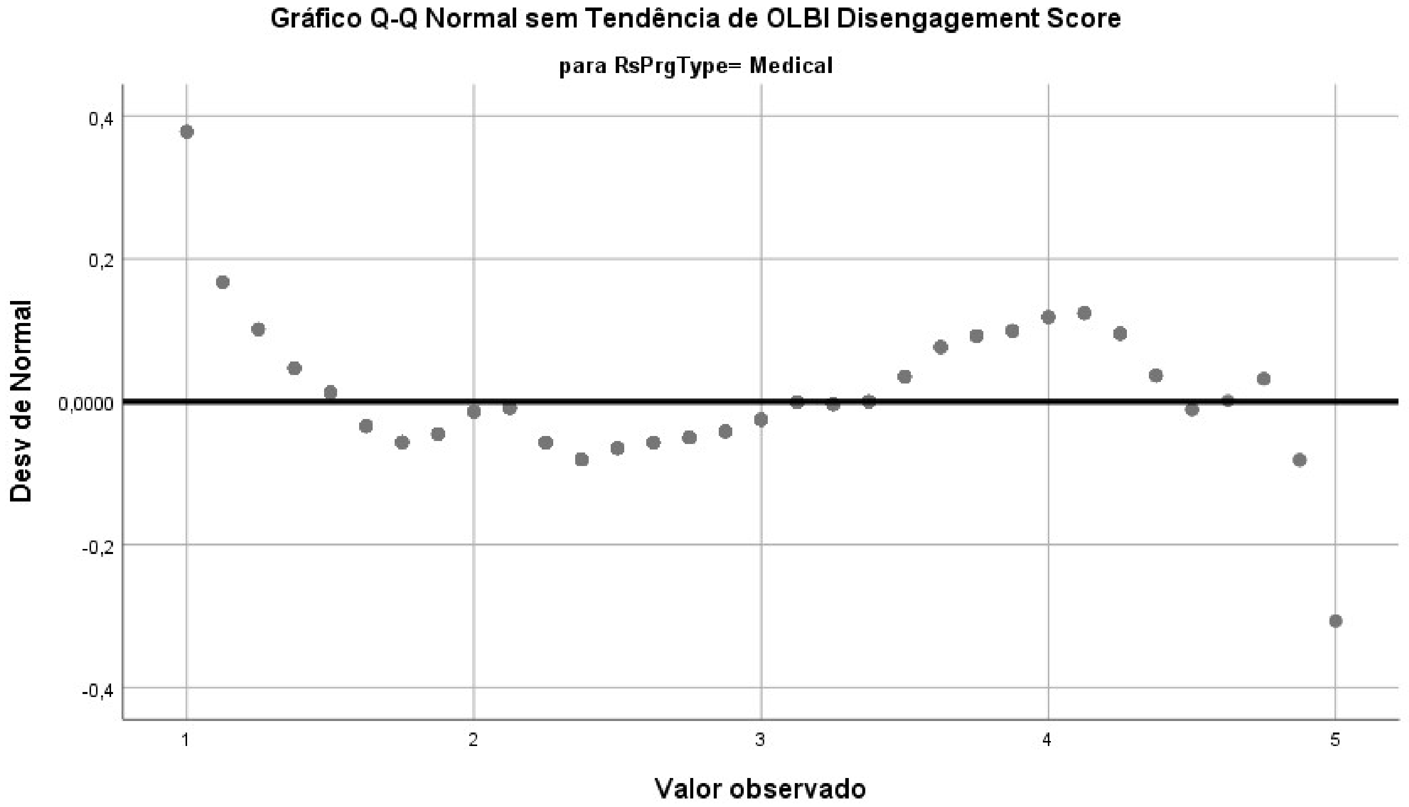

Gráfico Q-Q Normal sem Tendência de OLBI Disengagement Score
para RsPrgType= Medical
Desv de Normal
0,4
0,2
0,0000
-0,2
-0,4
1
2
3
4
5
Valor observado

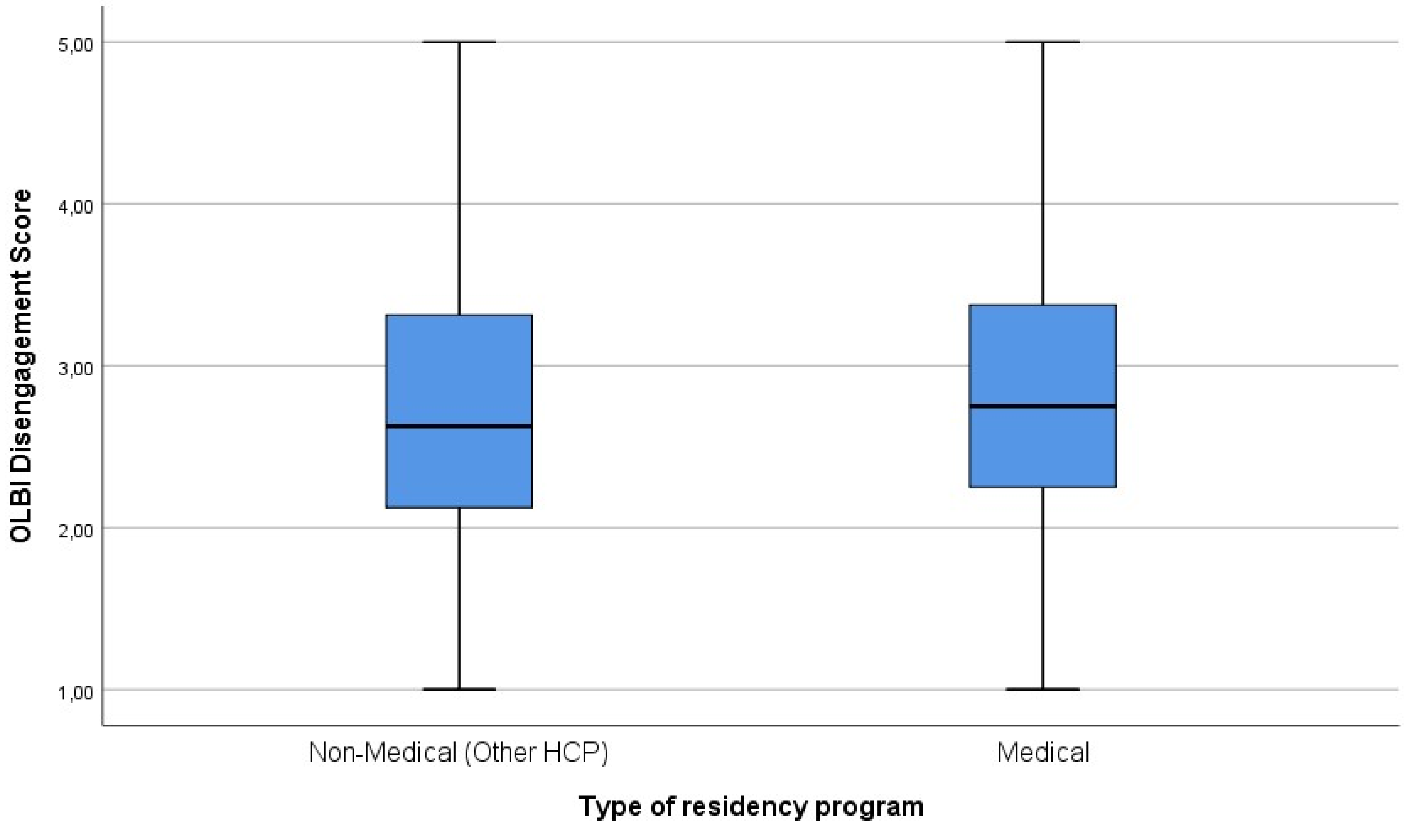

OLBI Exhaustion Score

Histogramas

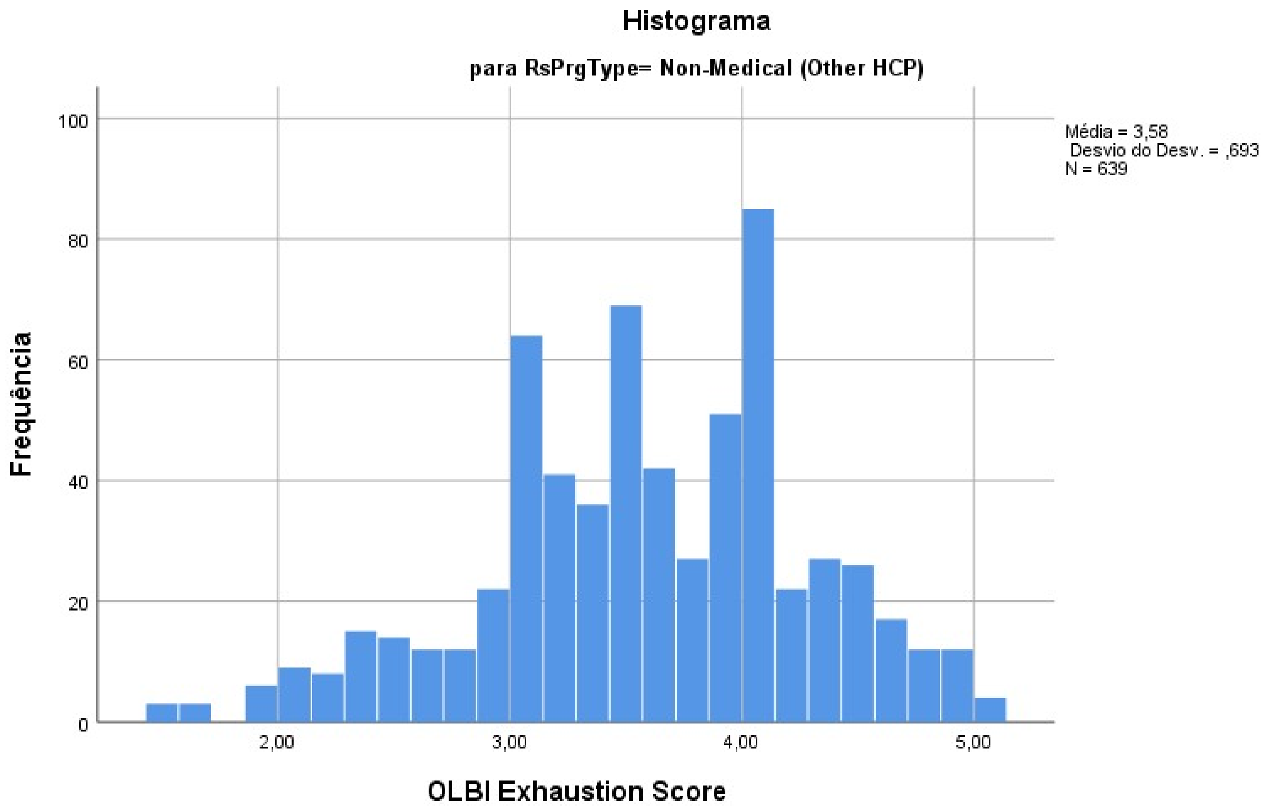

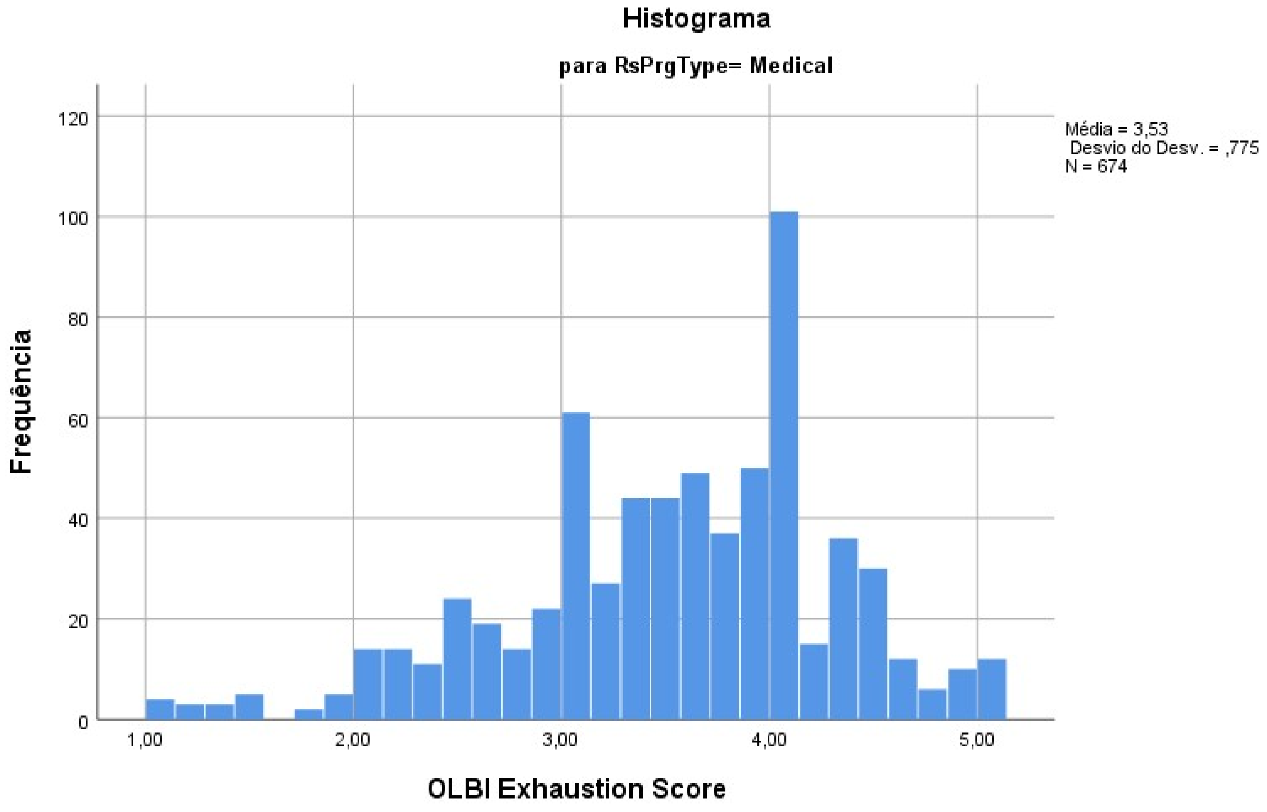

Gráfico Q-Q normais

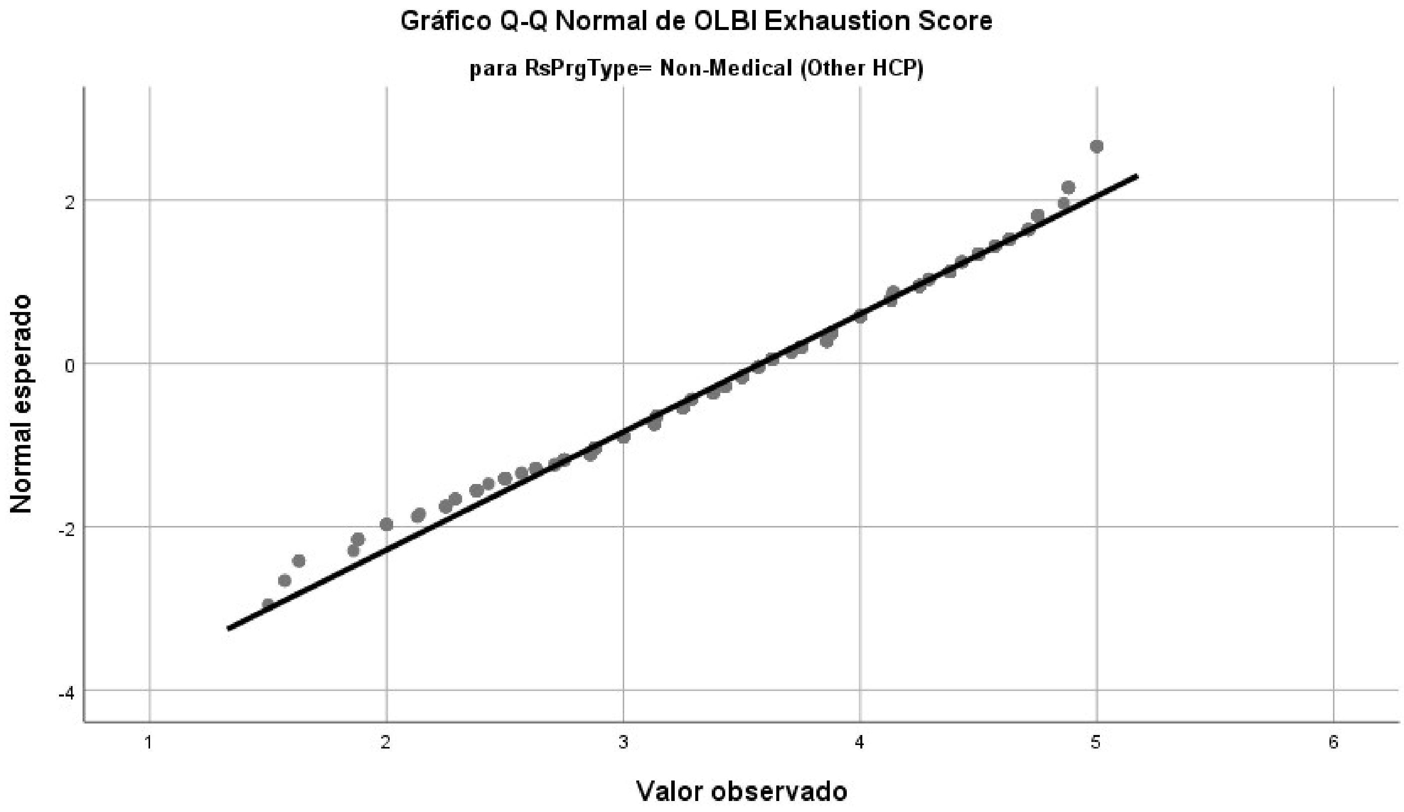

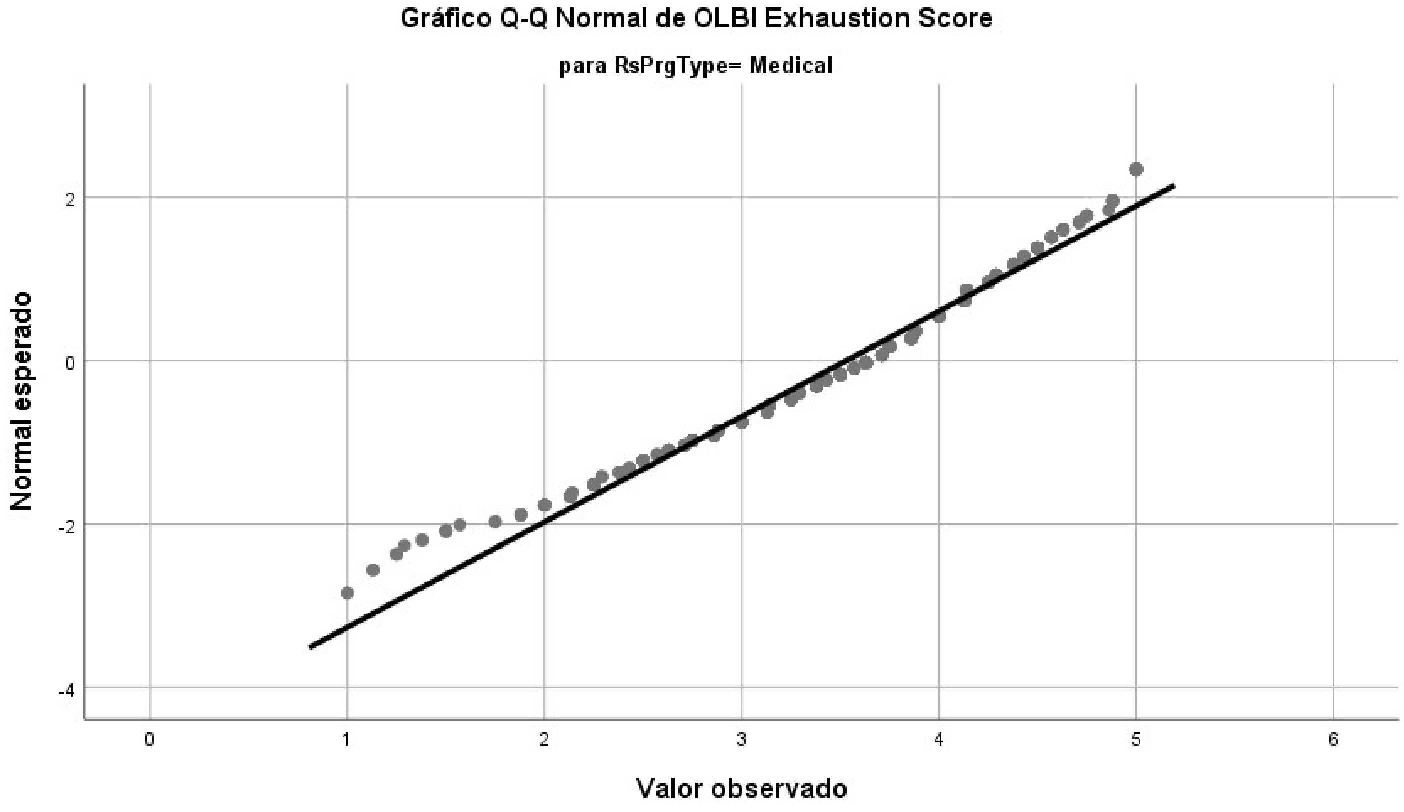

Gráfico Q-Q normais sem tendência

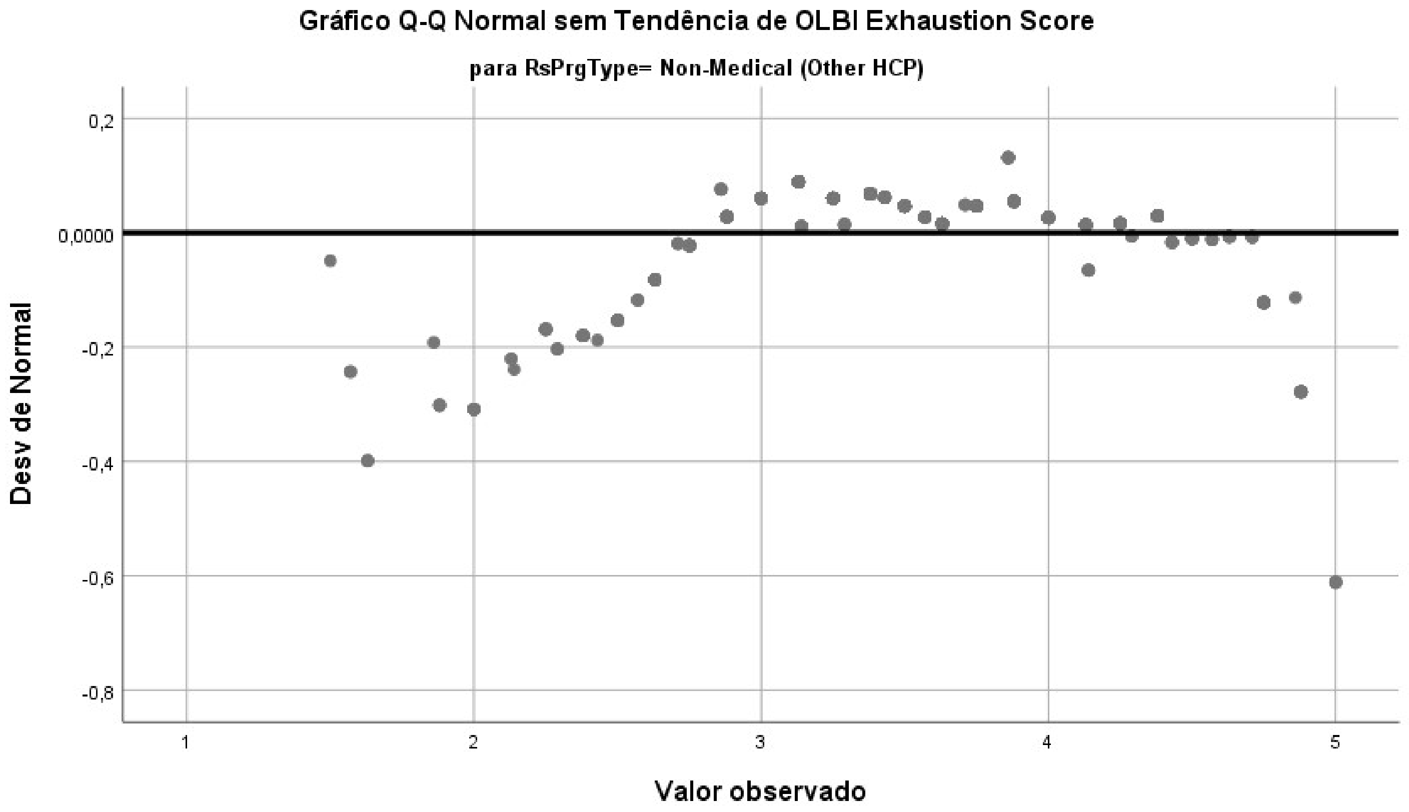
Gráfico Q-Q Normal sem Tendência de OLBI Exhaustion Score
para RsPrgType= Non-Medical (Other HCP)
0,2
0,0000
-0,2
-0,4
-0,6
-0,8
Desv de Normal
1
2
3
4
5
Valor observado

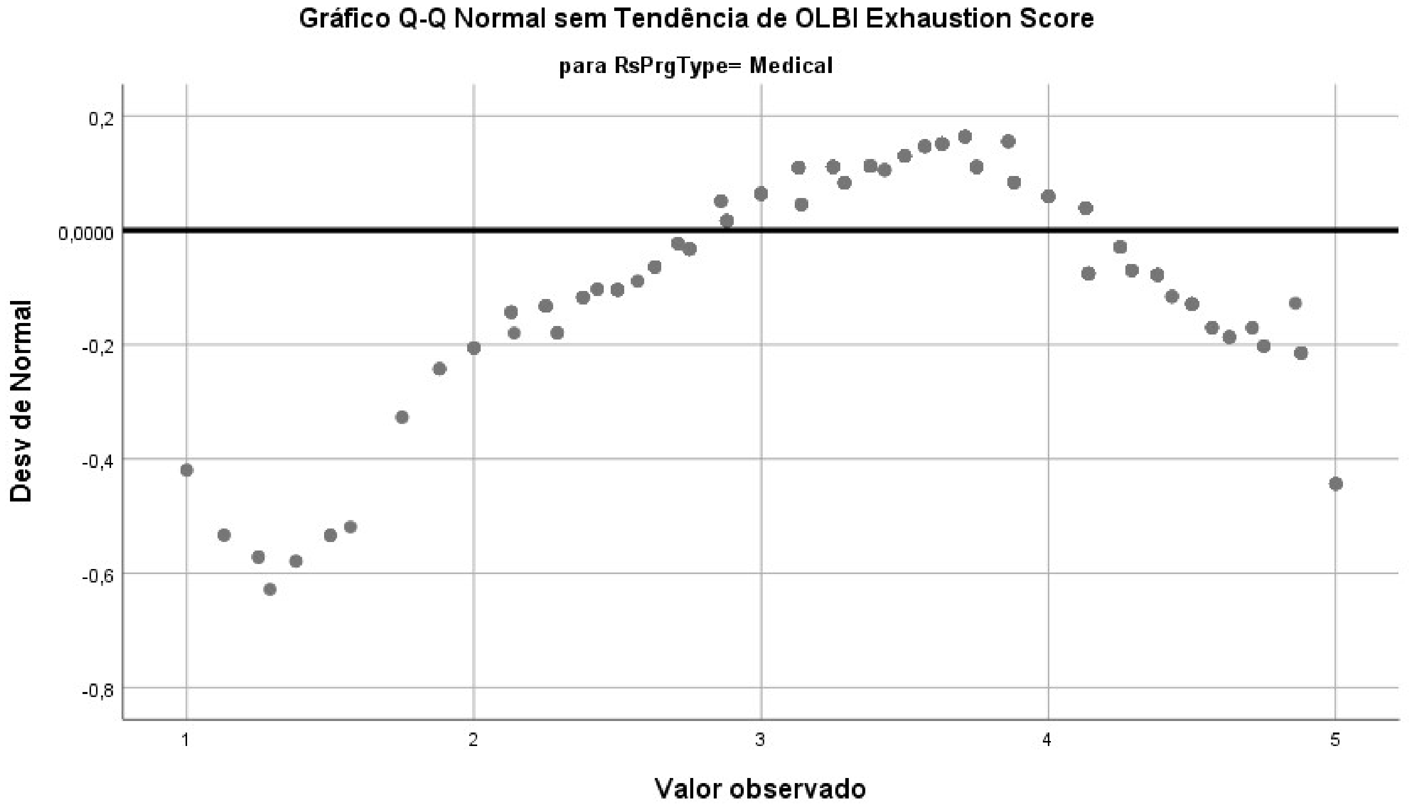
Gráfico Q-Q Normal sem Tendência de OLBI Exhaustion Score
para RsPrgType= Medical
0,2
0,0000
-0,2
-0,4
-0,6
-0,8
Desv de Normal
1
2
3
4
5
Valor observado

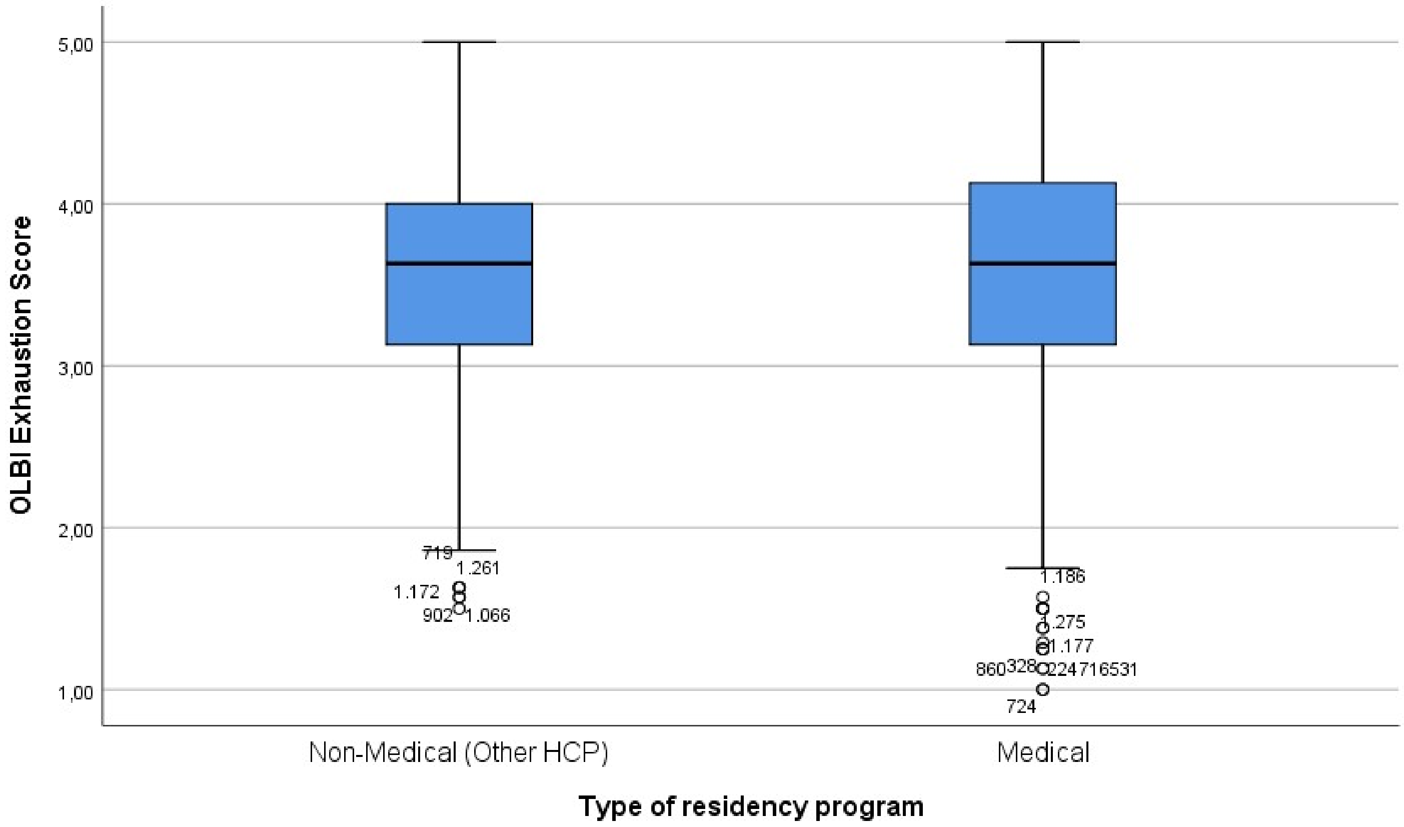

OLBI Total Score

Histogramas

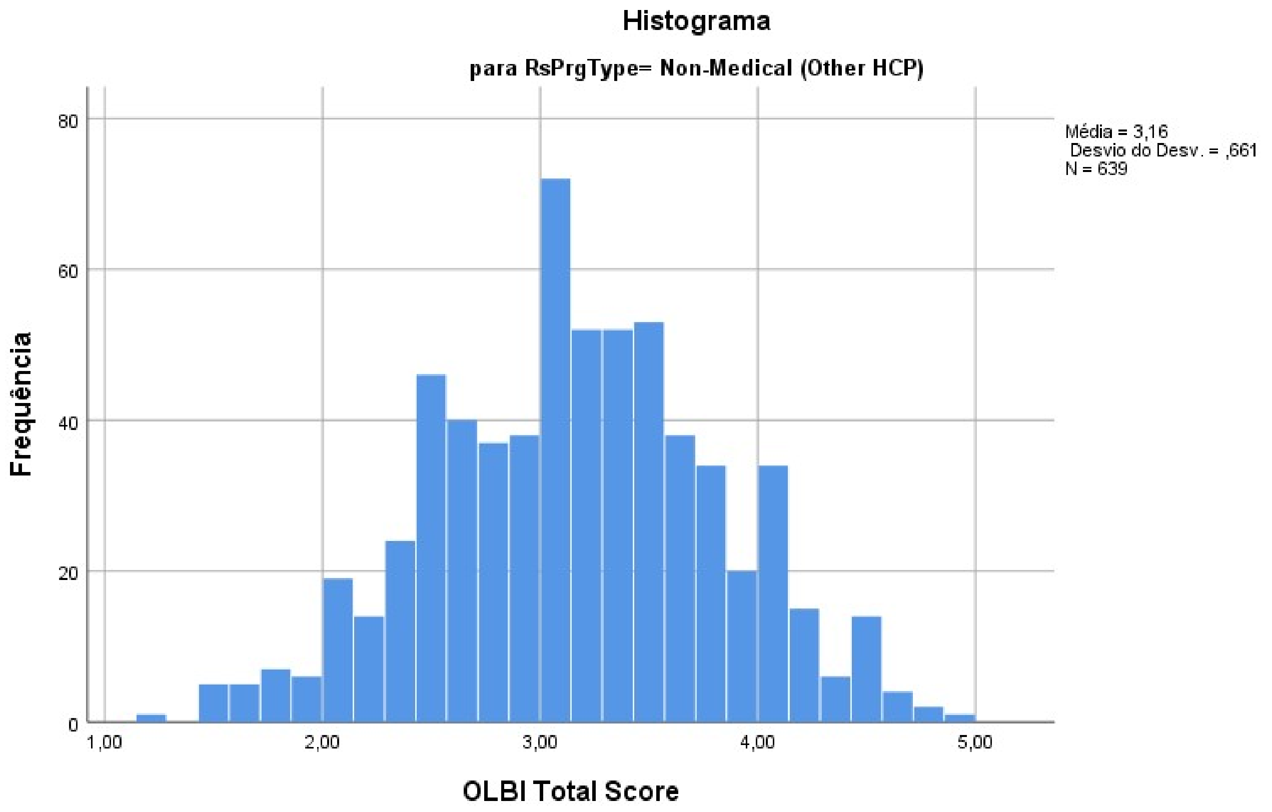

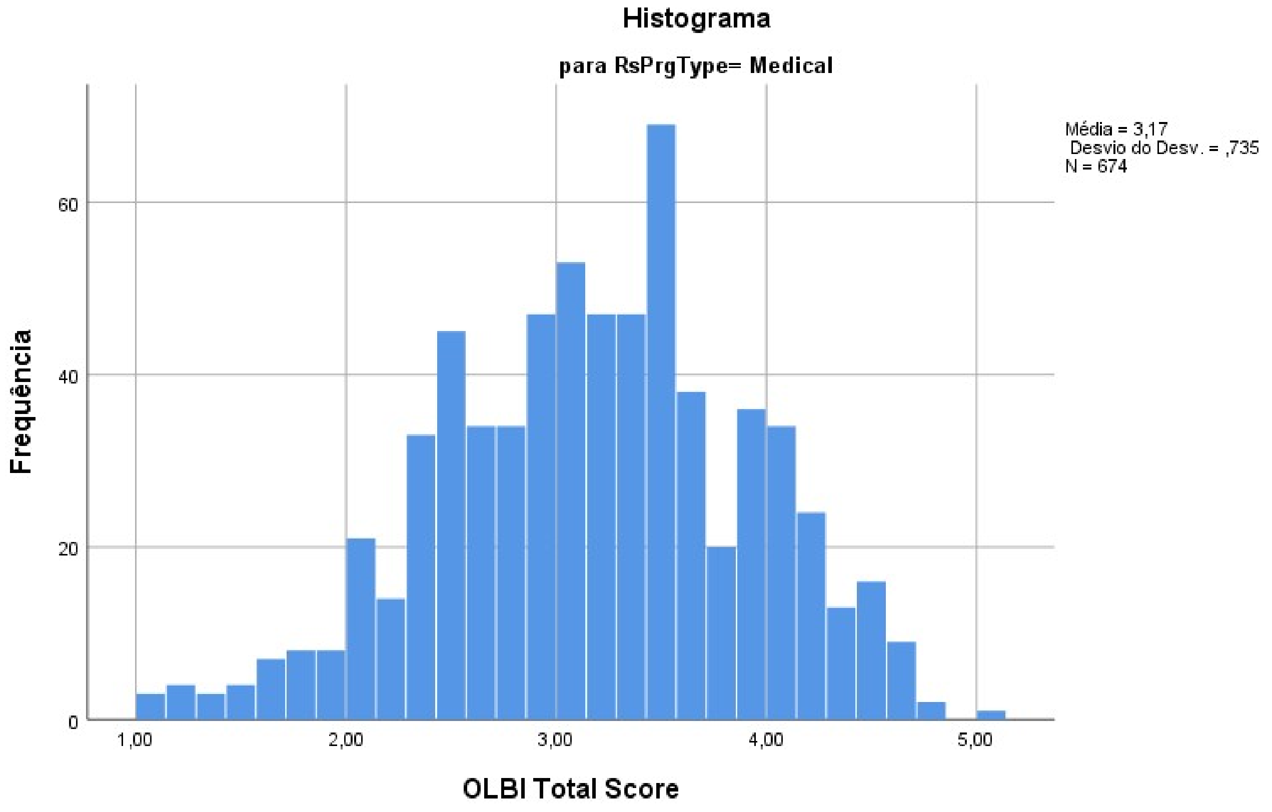

Gráfico Q-Q normais

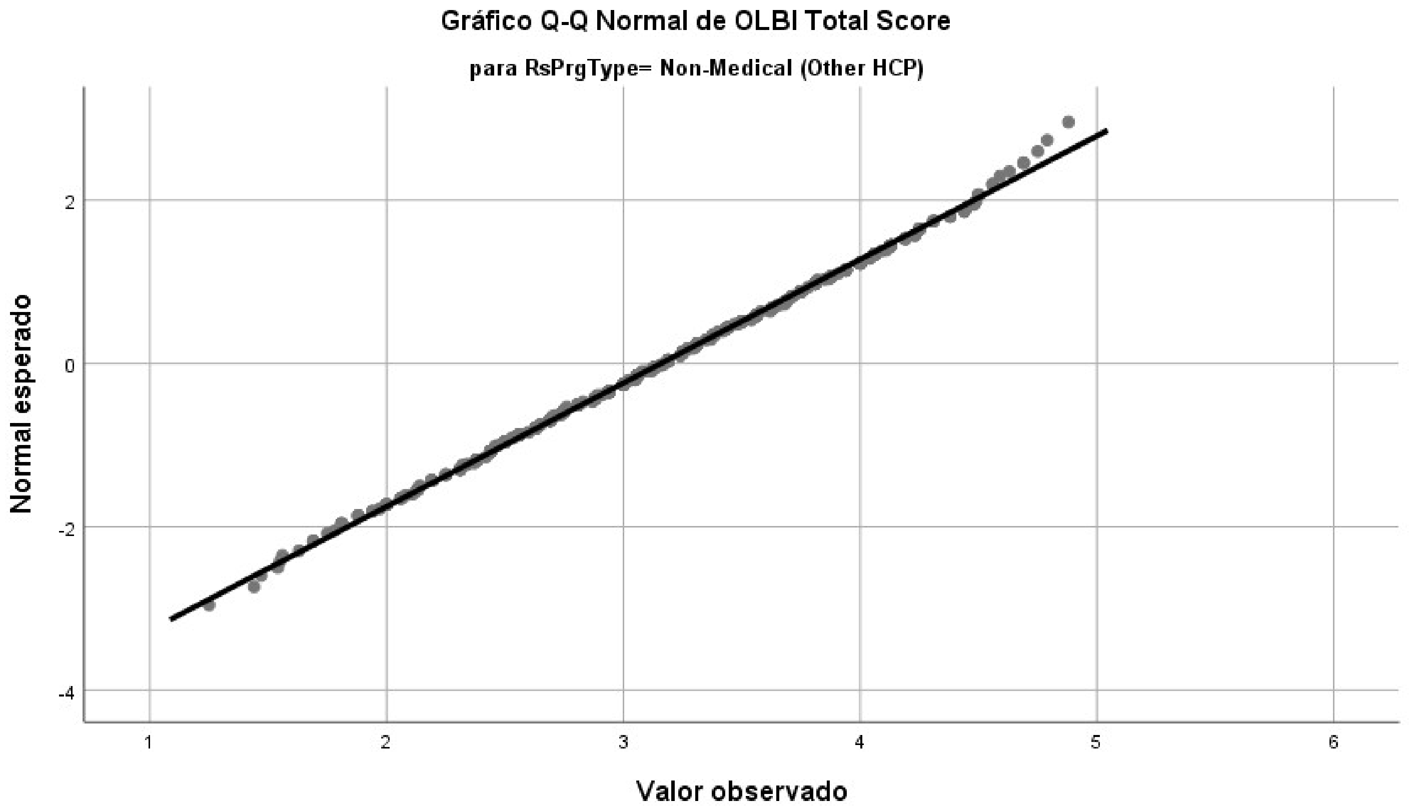

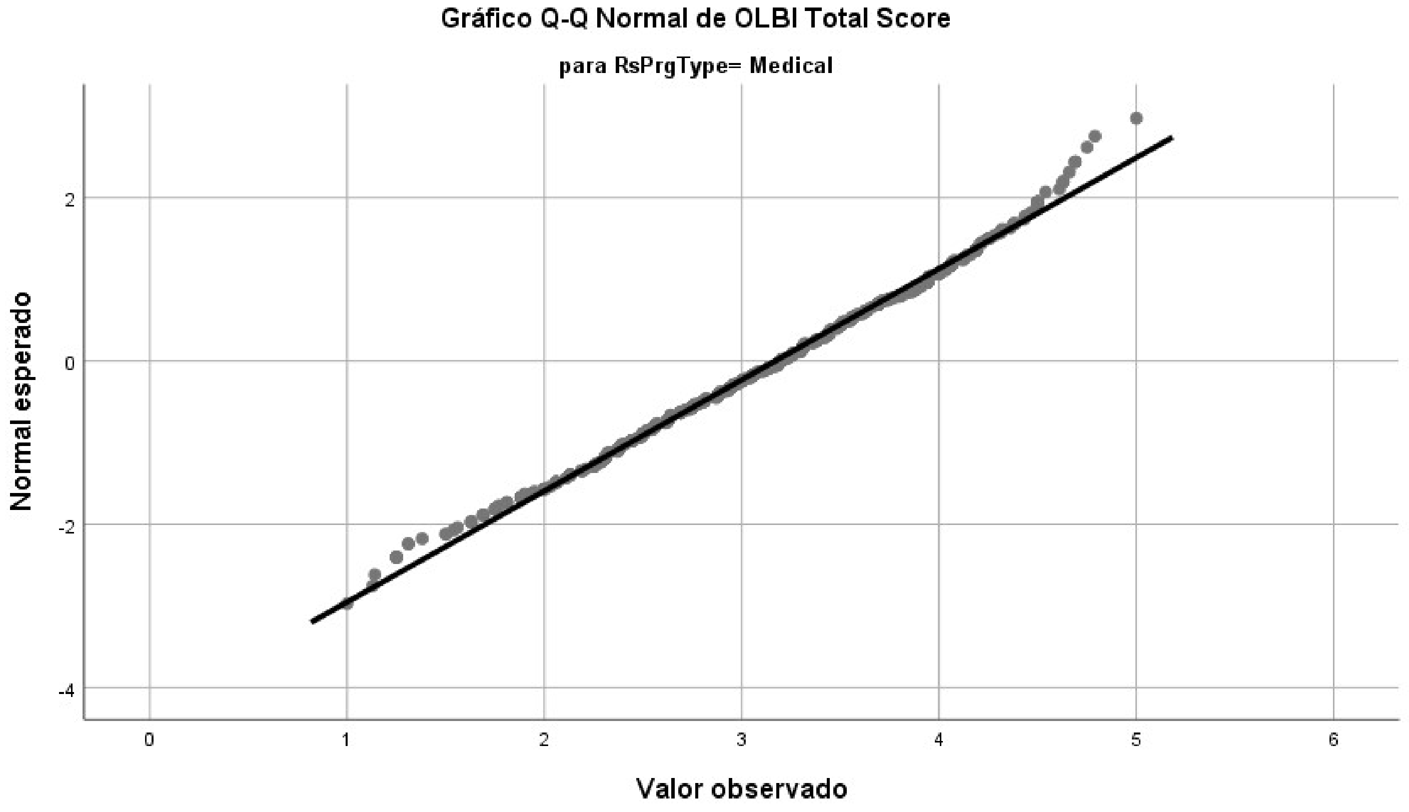

Gráfico Q-Q normais sem tendência

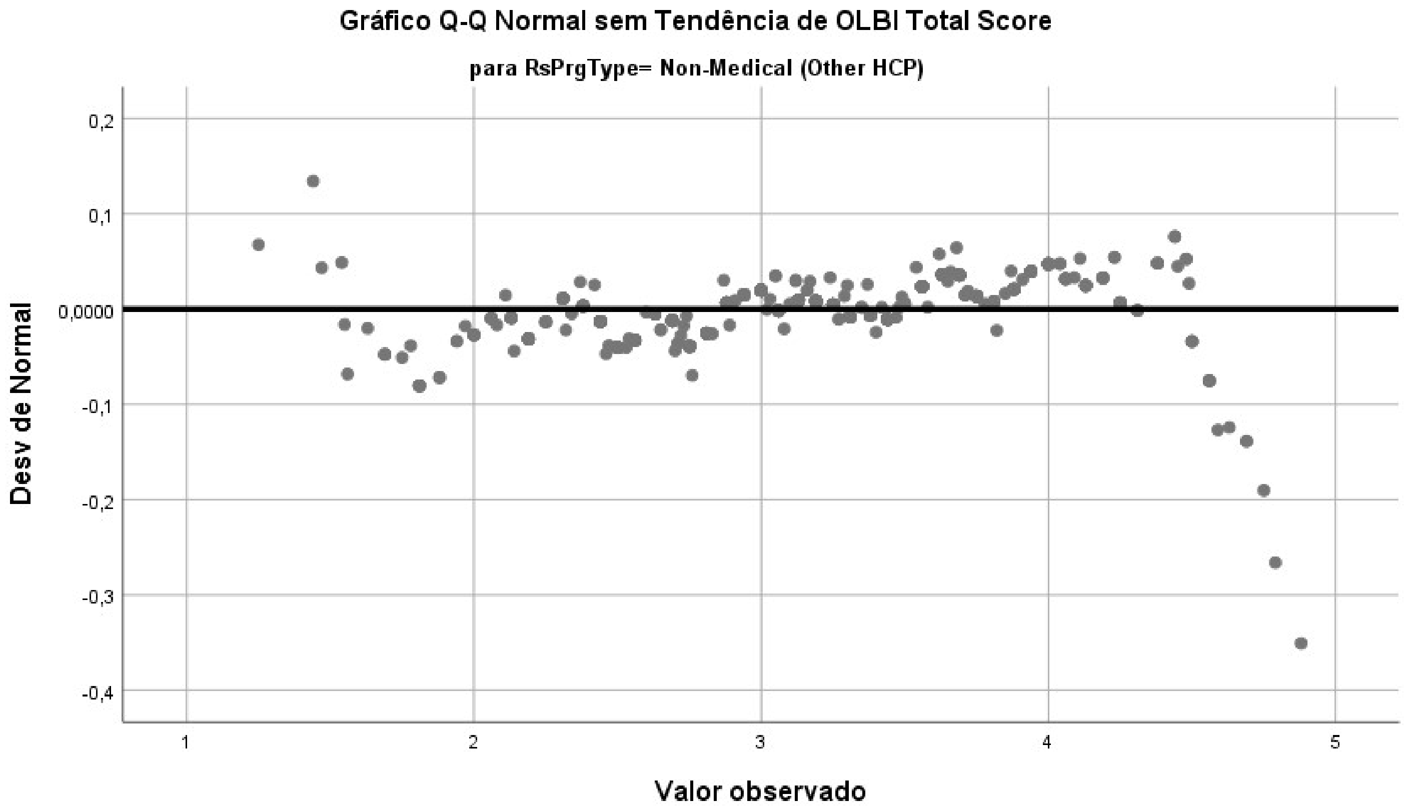
Gráfico Q-Q Normal sem Tendência de OLBI Total Score
para RsPrgType= Non-Medical (Other HCP)
Desv de Normal
0,2
0,1
0,0000
-0,1
-0,2
-0,3
-0,4
1
2
3
4
5
Valor observado

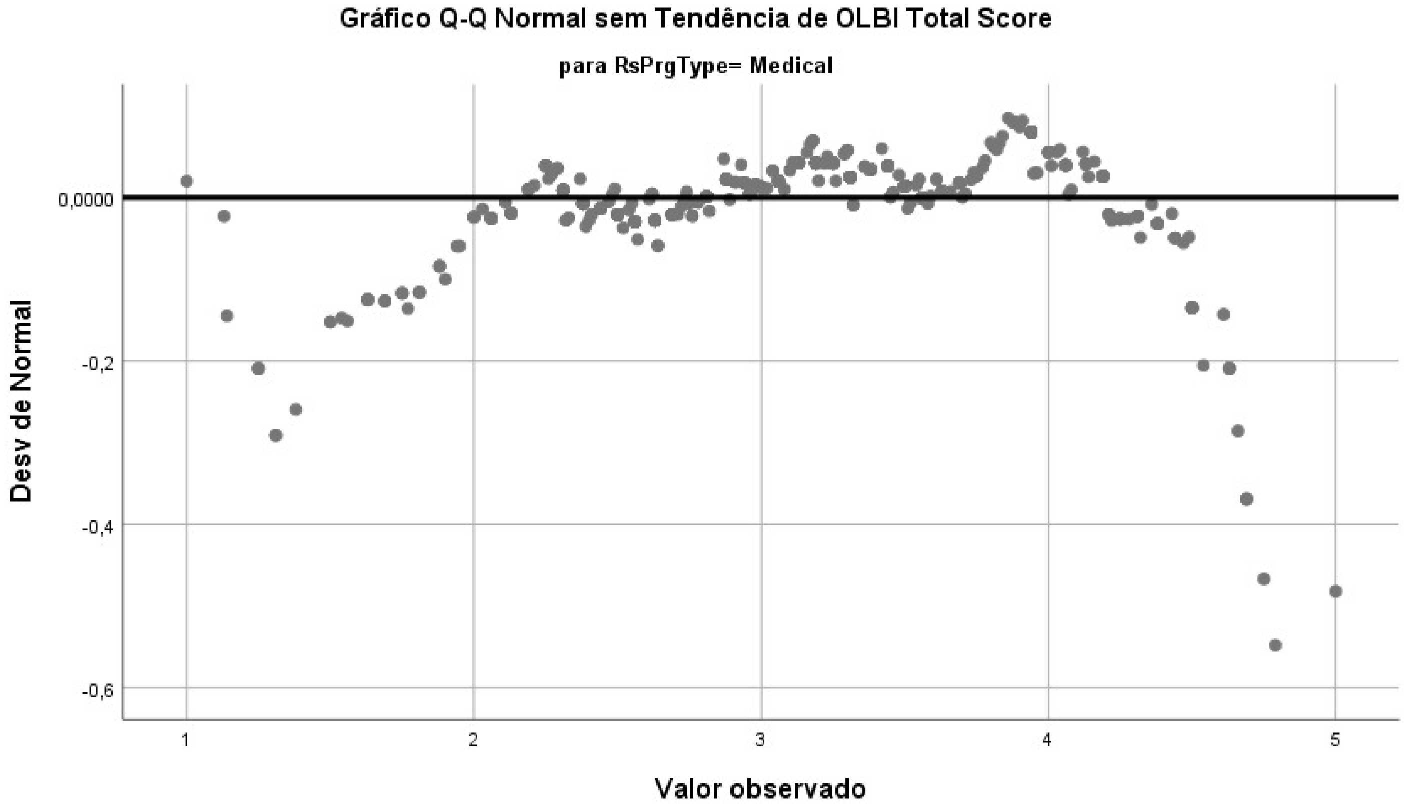
Gráfico Q-Q Normal sem Tendência de OLBI Total Score
para RsPrgType= Medical
Desv de Normal
0,0000
-0,2
-0,4
-0,6
1
2
3
4
5
Valor observado

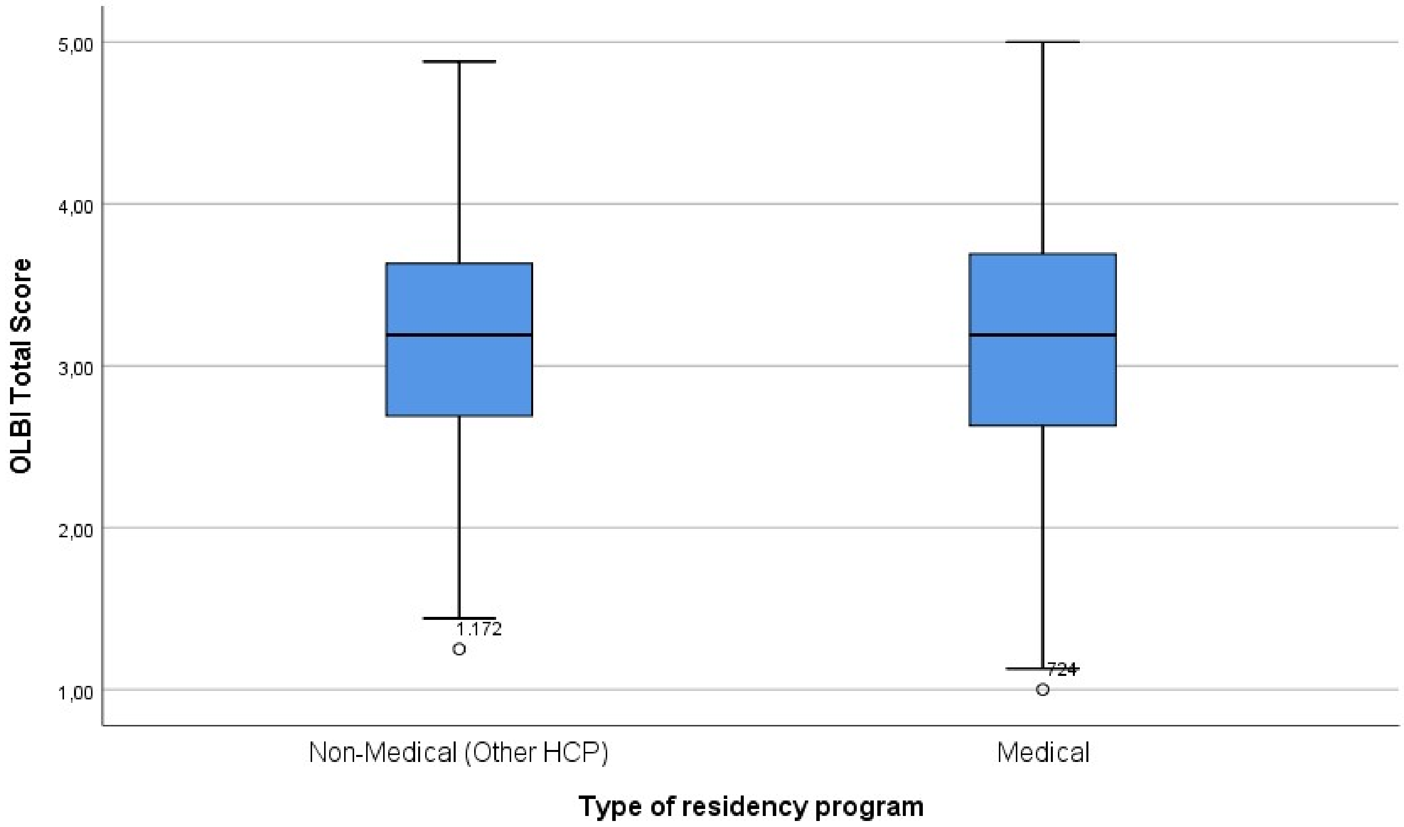

Na sua opinião, qual o seu grau de autonomia para decidir condutas no trabalho? (EAV 1-10)

Histogramas

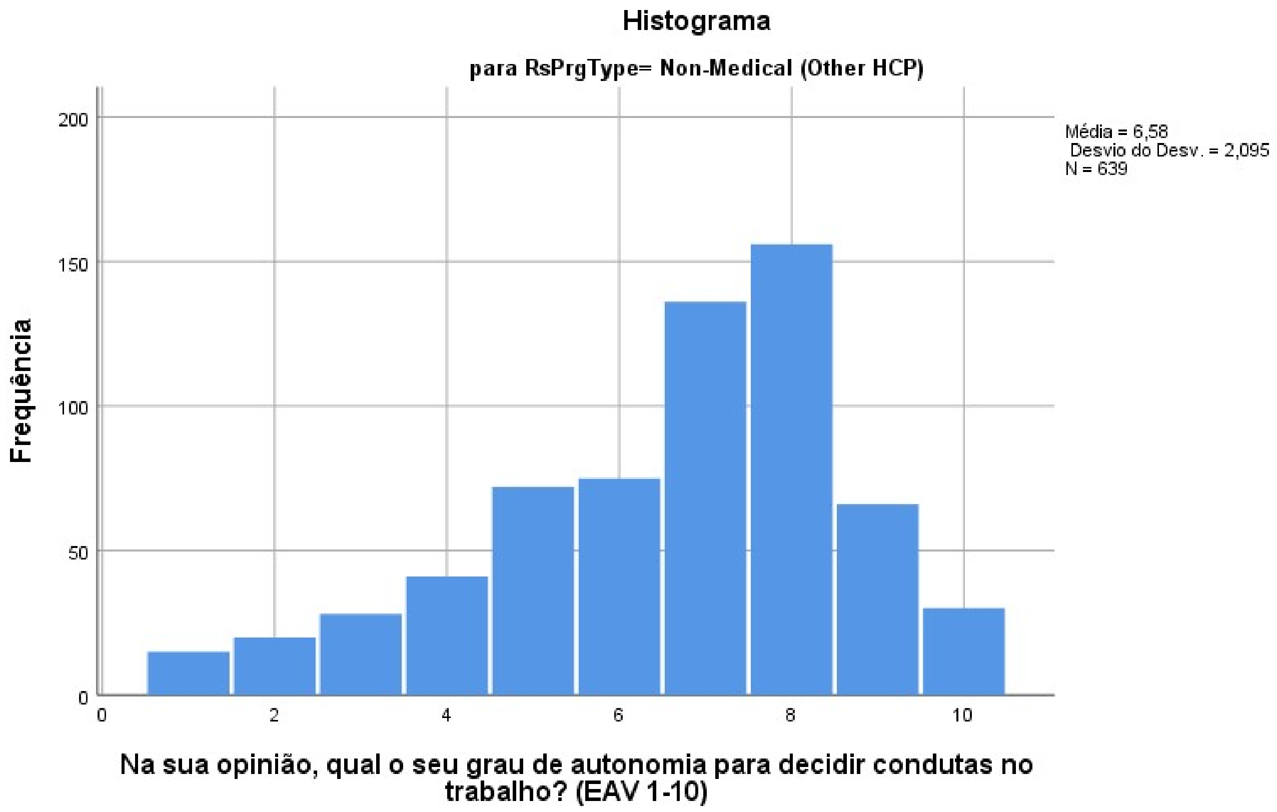

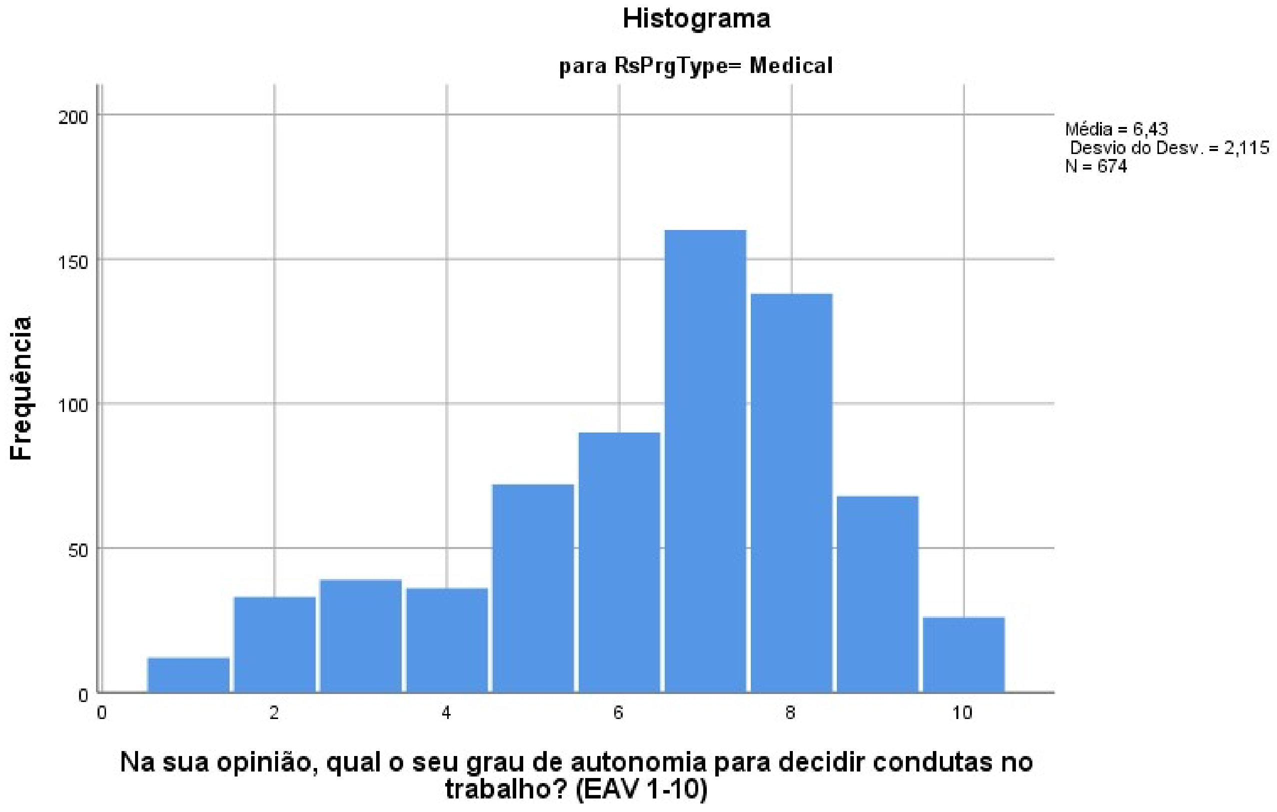

Gráfico Q-Q normais

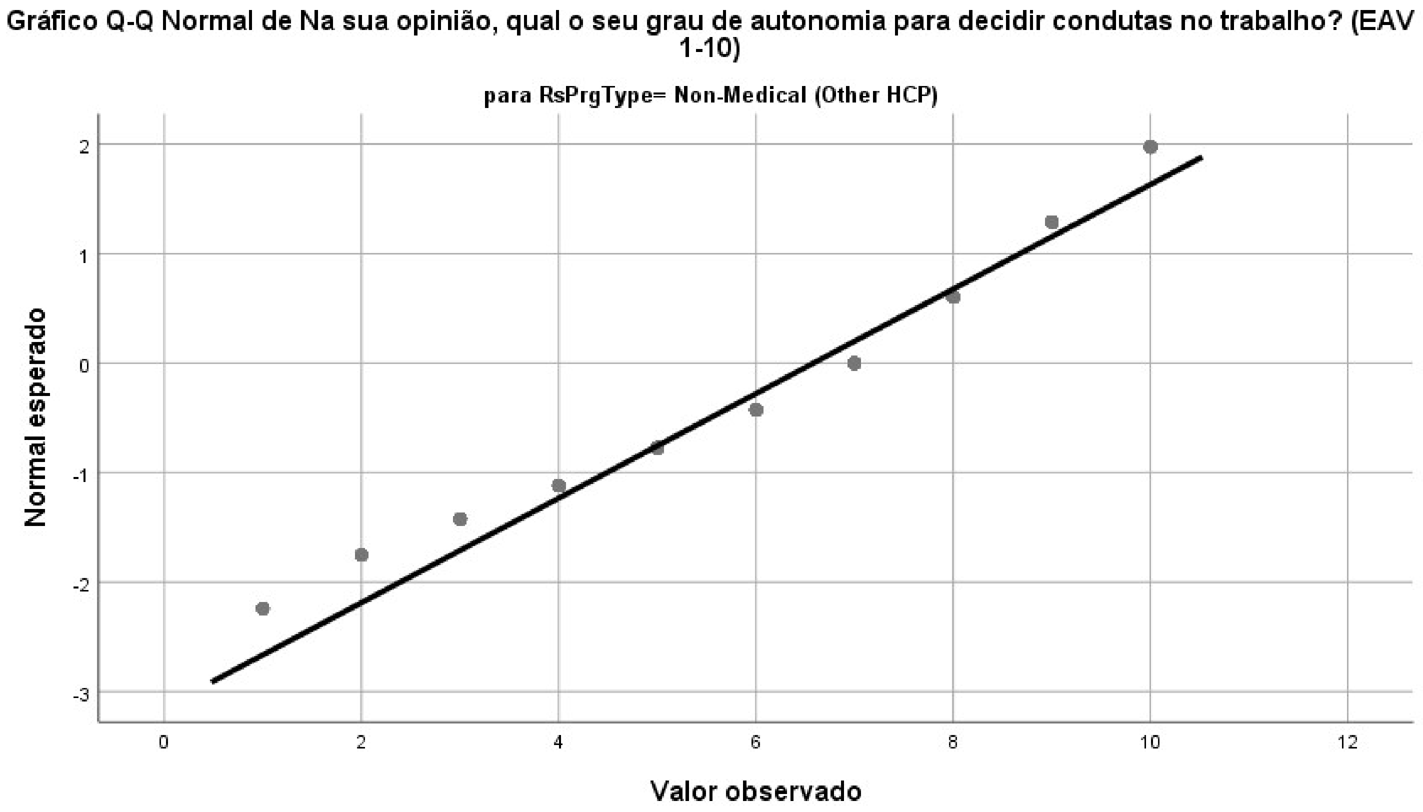

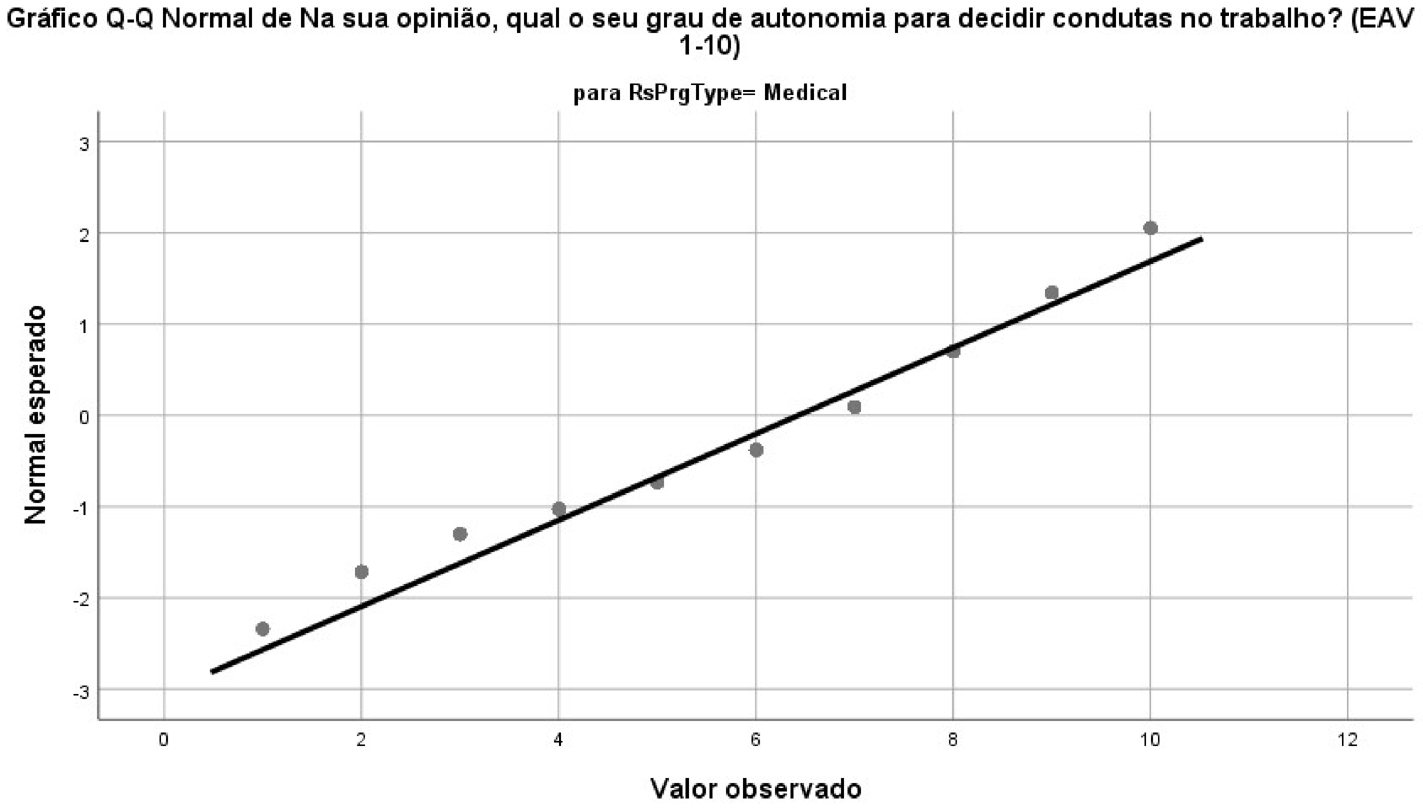

Gráfico Q-Q normais sem tendência

Gráfico Q-Q Normal sem Tendência de Na sua opinião, qual o seu grau de autonomia para decidir condutas no trabalho? (EAV 1-10)

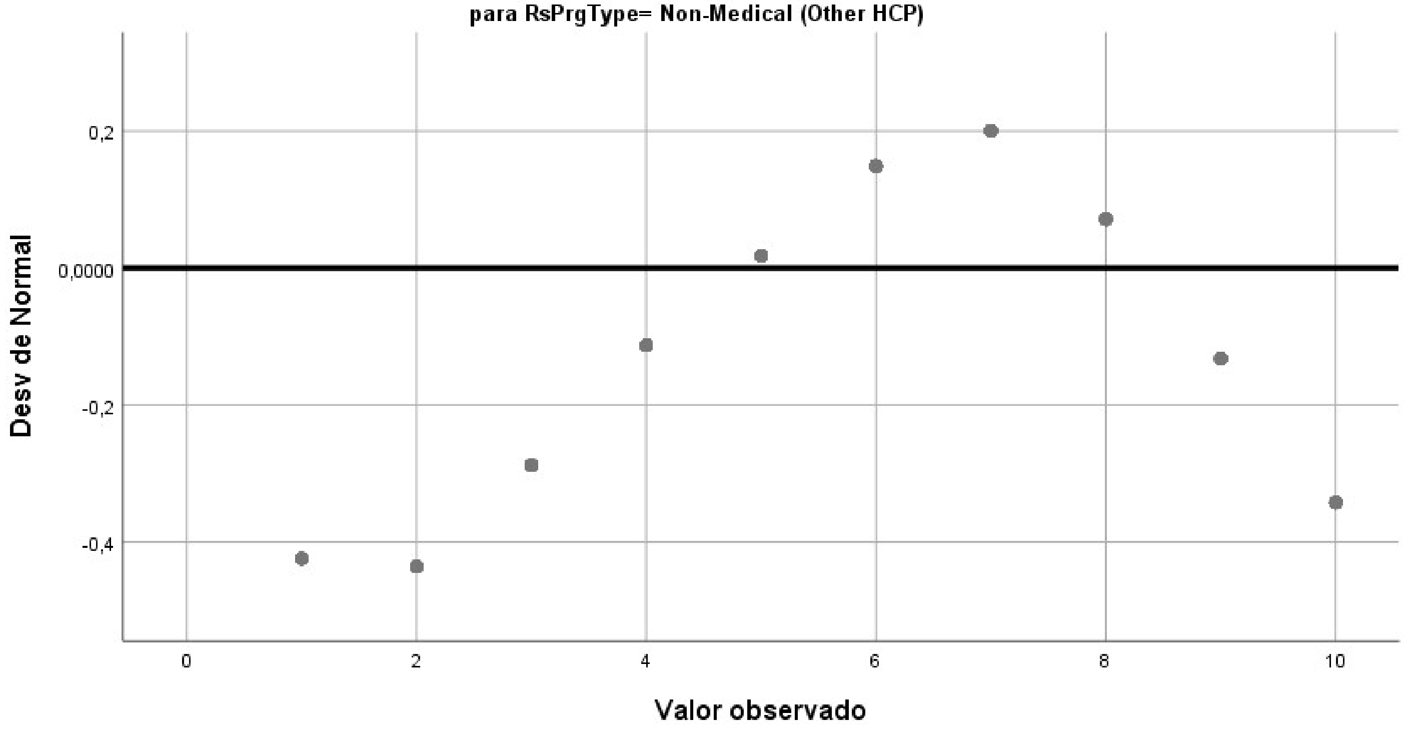

Gráfico Q-Q Normal sem Tendência de Na sua opinião, qual o seu grau de autonomia para decidir condutas no trabalho? (EAV 1-10)

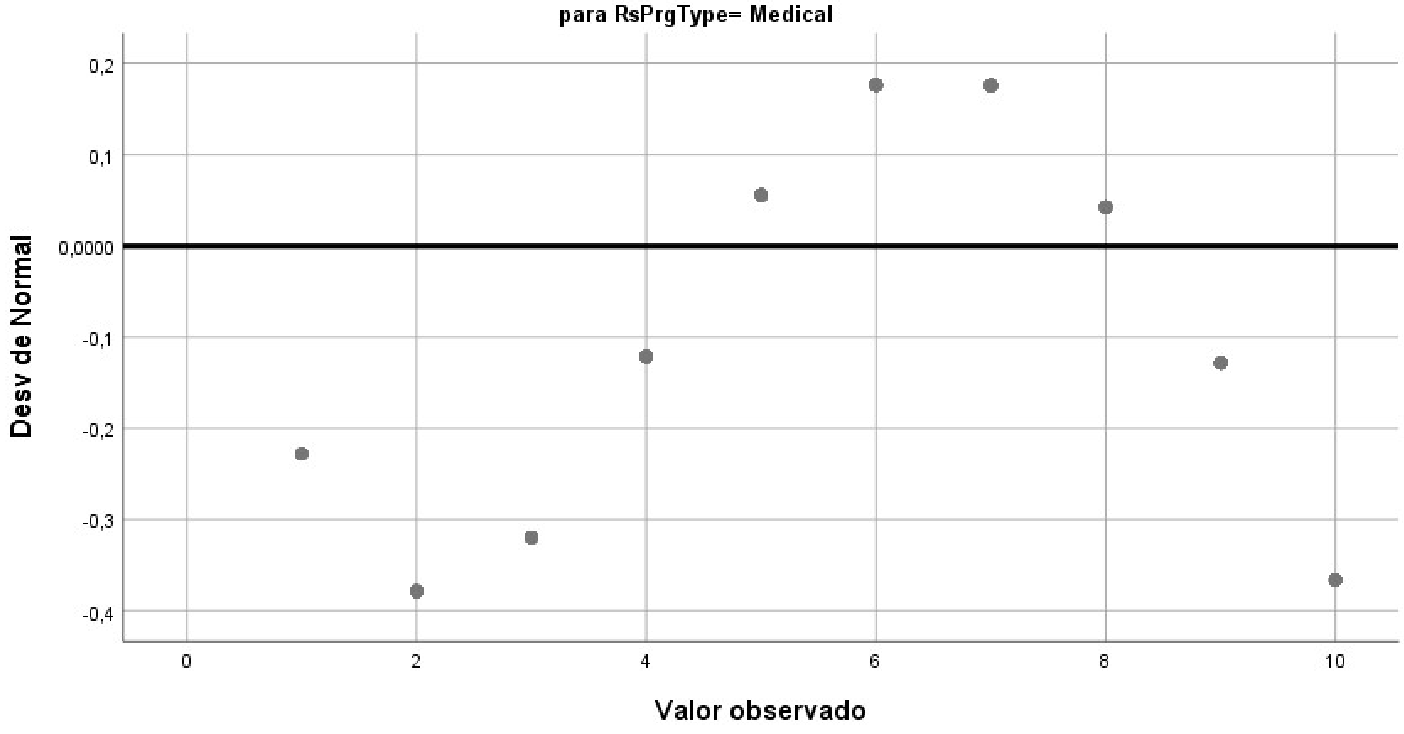

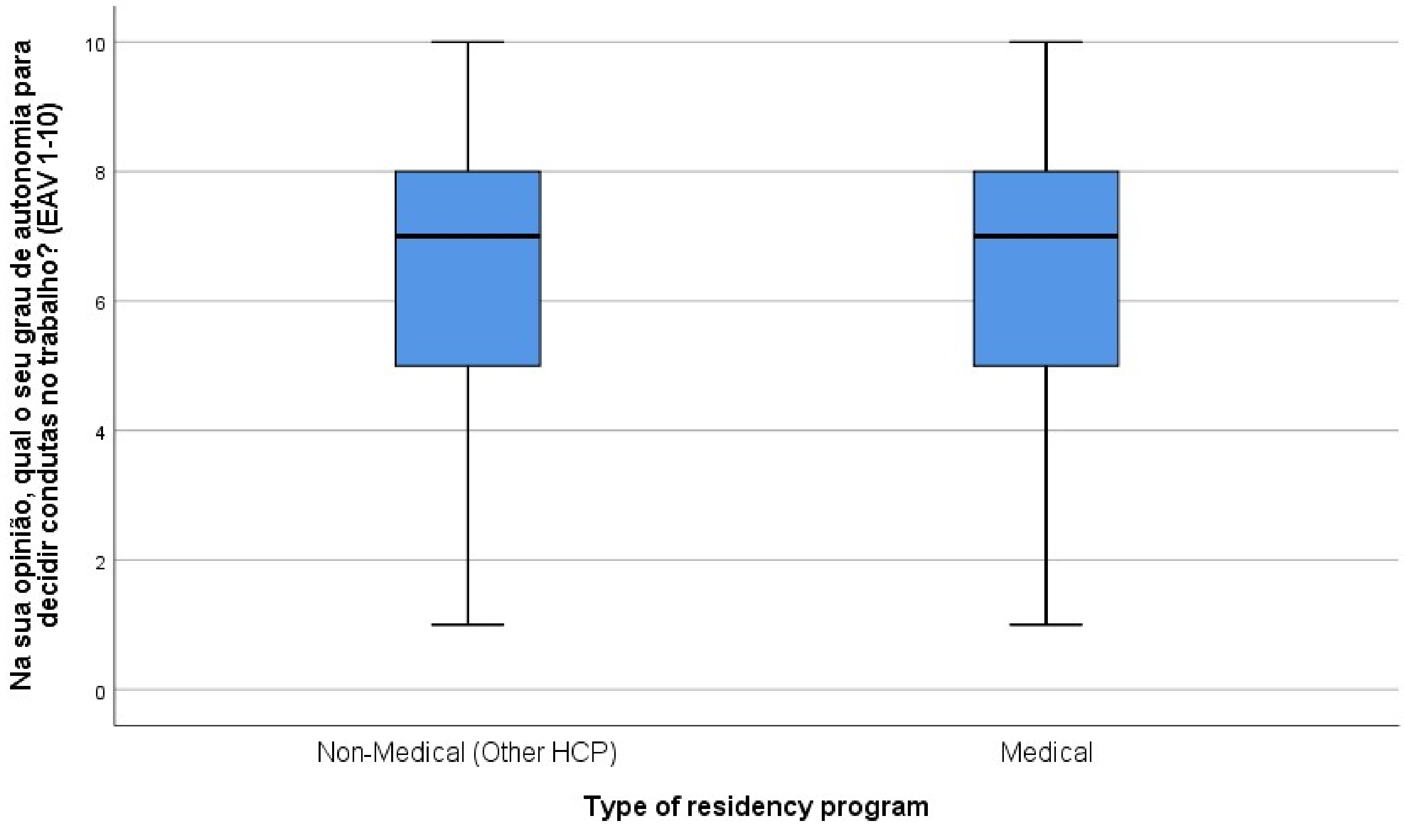

Na sua opinião, qual o grau de adequação da organização pedagógica de seu programa de residência profissional? (EAV 1-10)

Histogramas

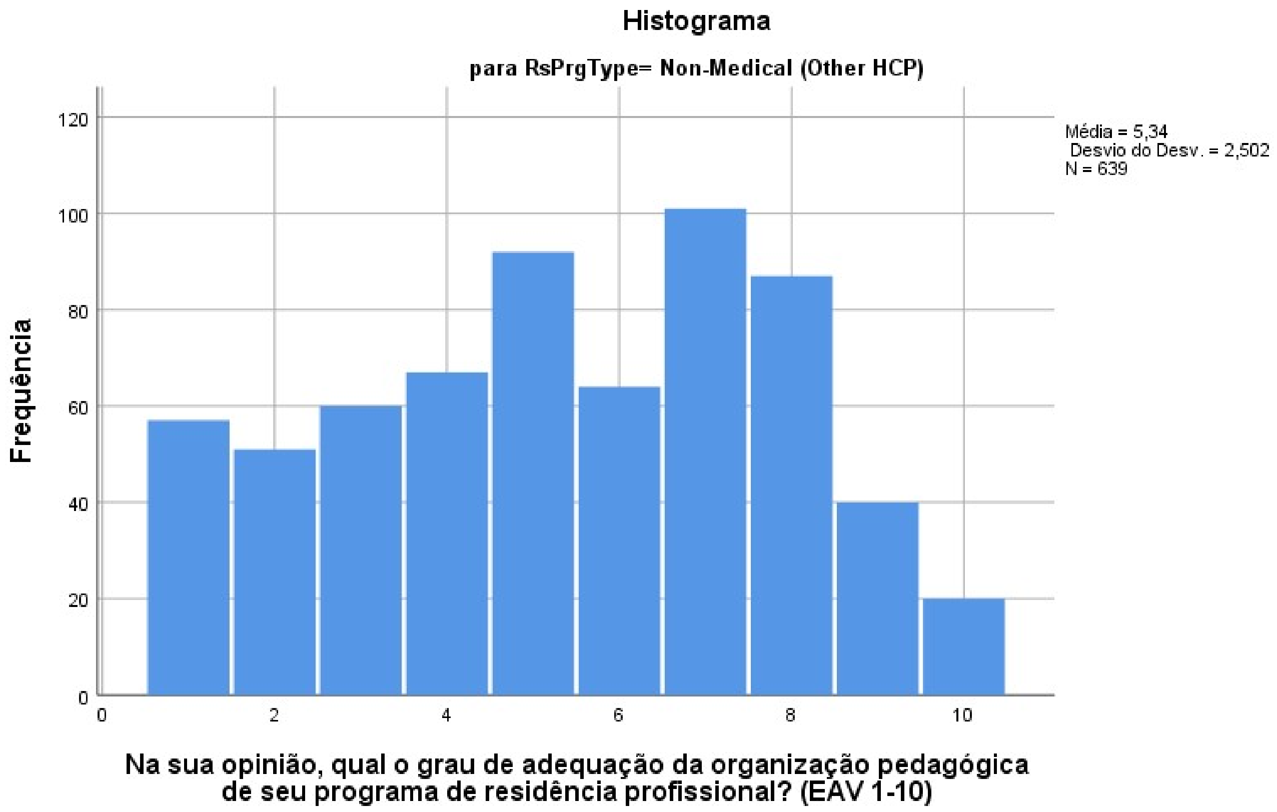

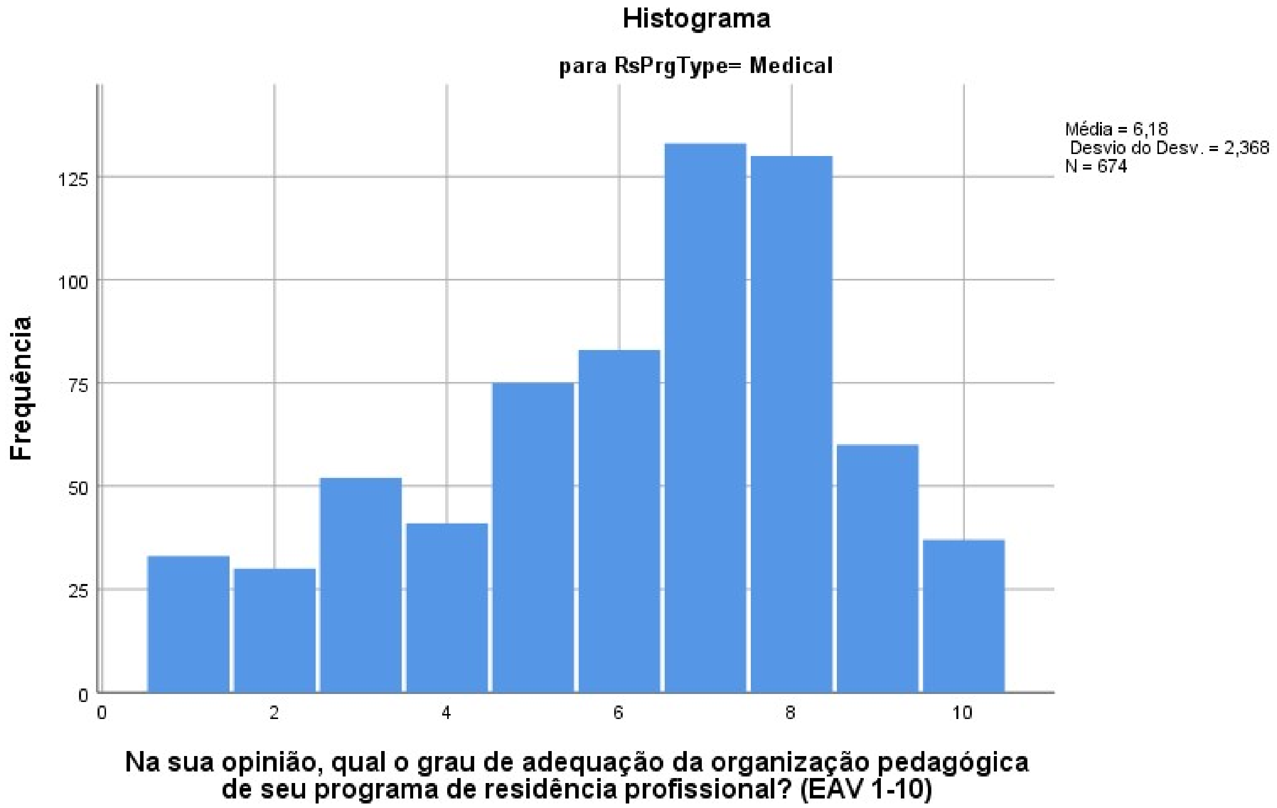

Gráfico Q-Q normais

Gráfico Q-Q Normal de Na sua opinião, qual o grau de adequação da organização pedagógica de seu programa de residência profissional? (EAV 1-10)

para RsPrgType= Non-Medical (Other HCP)

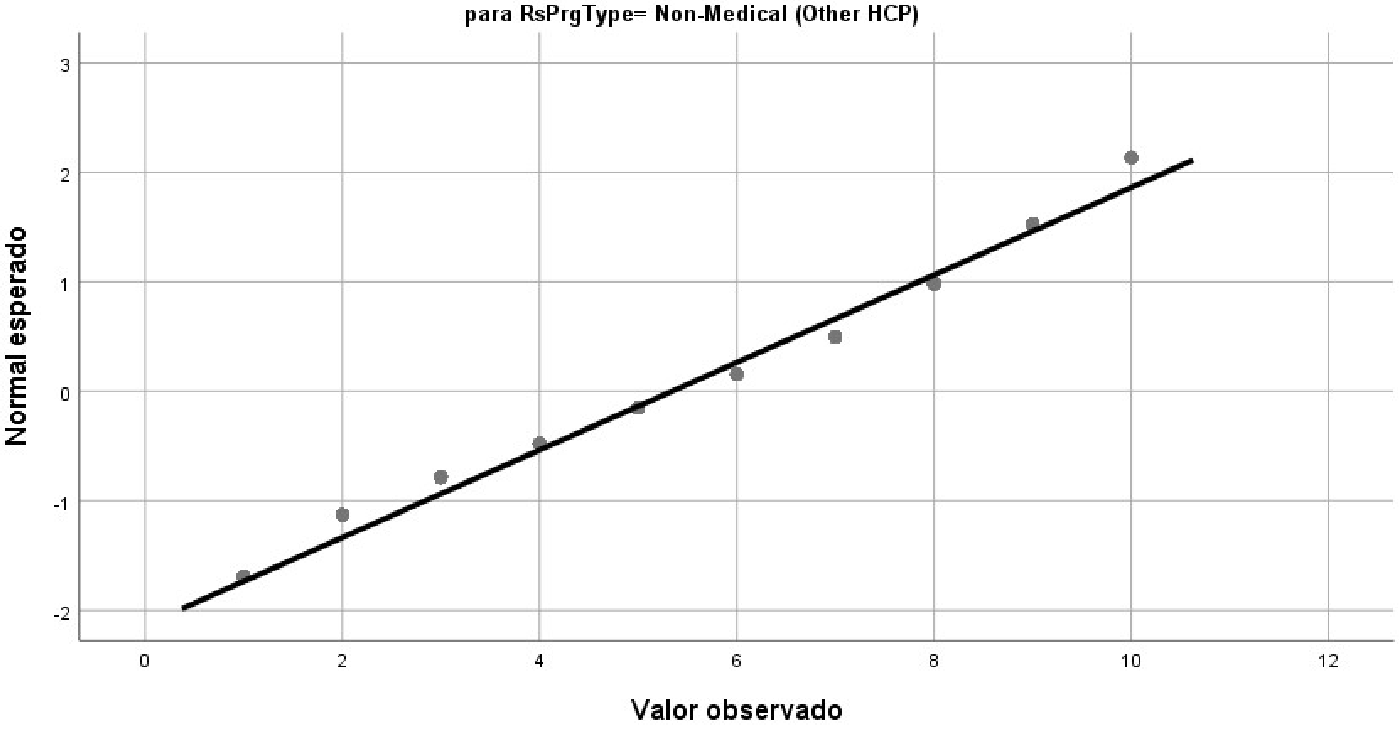

Gráfico Q-Q Normal de Na sua opinião, qual o grau de adequação da organização pedagógica de seu programa de residência profissional? (EAV 1-10)

para RsPrgType= Medical

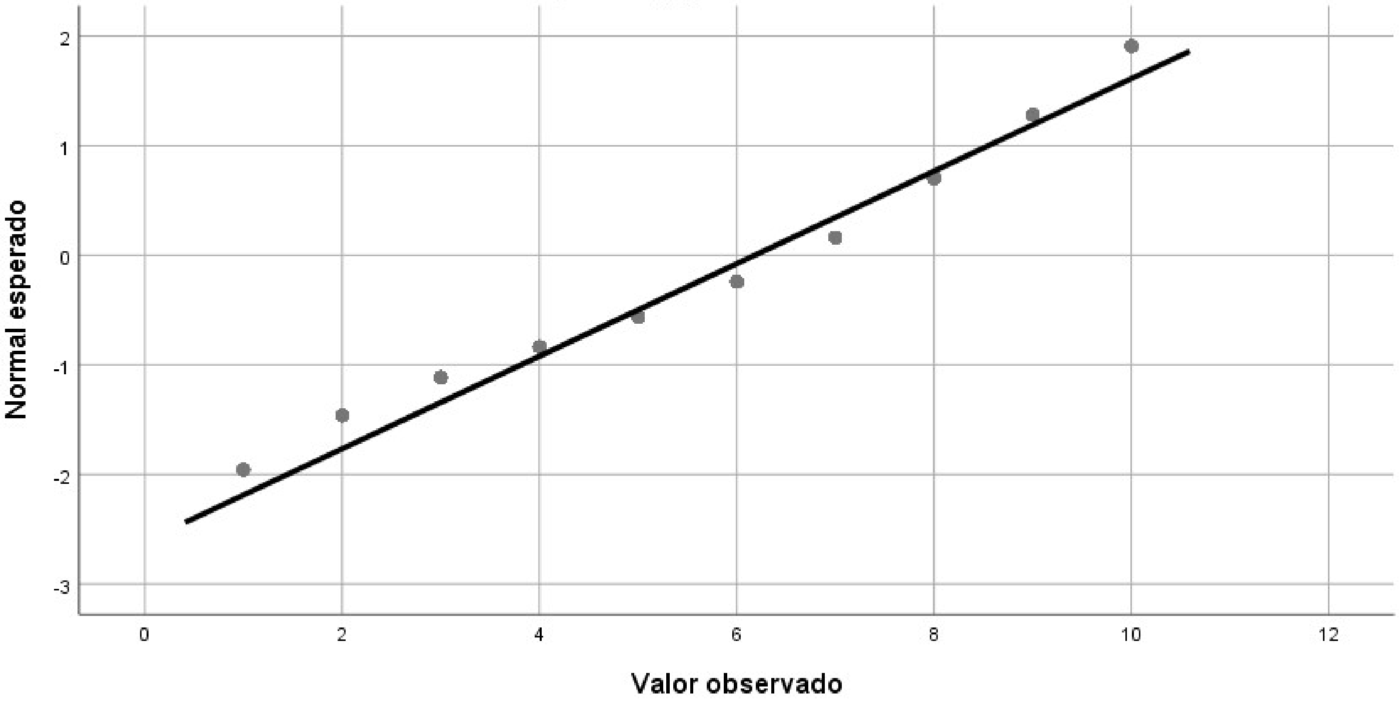

Gráfico Q-Q normais sem tendência

Gráfico Q-Q Normal sem Tendência de Na sua opinião, qual o grau de adequação da organização pedagógica de seu programa de residência profissional? (EAV 1-10)

para RsPrgType= Non-Medical (Other HCP)

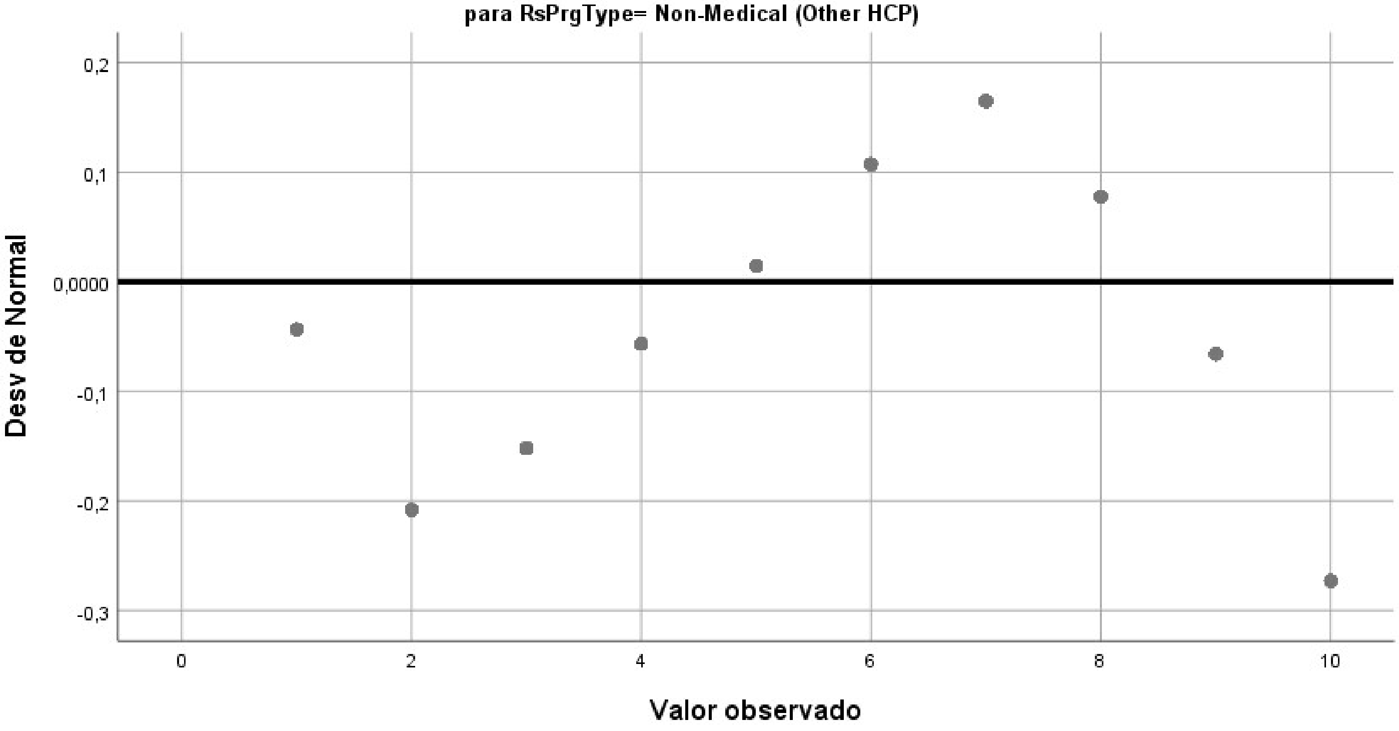

Gráfico Q-Q Normal sem Tendência de Na sua opinião, qual o grau de adequação da organização pedagógica de seu programa de residência profissional? (EAV 1-10)

para RsPrgType= Medical

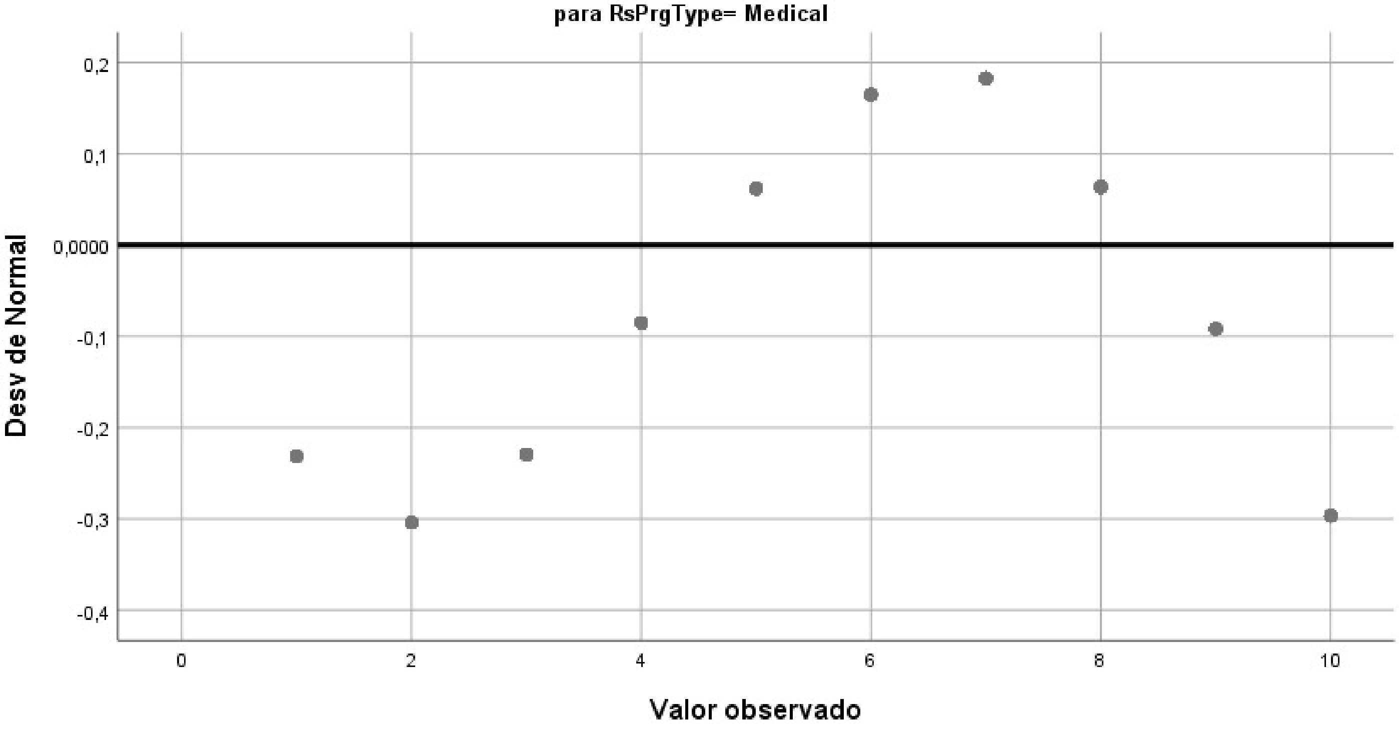

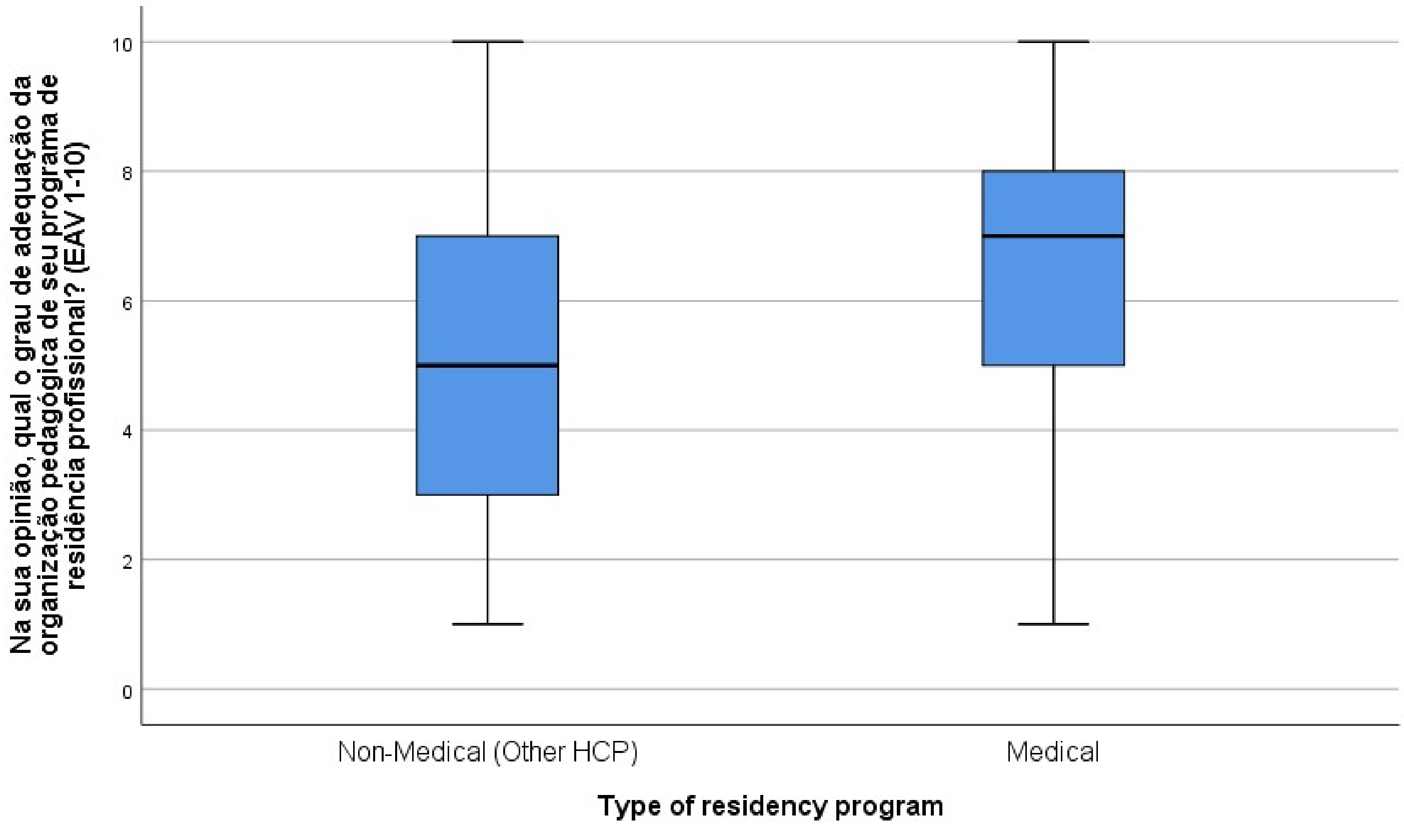


```
T-TEST GROUPS=RsPrgType(0 1)
  /MISSING=ANALYSIS
  /VARIABLES=Age DASS21_D DASS21_A DASS21_S PHQ9_Score BRCS_Score OLBI_D OLBI_E OLBI_Total
Autonomy
    PedagStruct
  /CRITERIA=CI(.95).
```

Teste-T

Observações

Saída criada		20-SEP-2020 12:14:47
Comentários		
Entrada	Dados	C:\Users\User\Documents\Pesquisa\Fellow\FellowGenData_V1.sav
	Conjunto de dados ativo	ConjuntodeDados1
	Filtro	<none>
	Ponderação	<none>
	Arquivo Dividido	<none>
	N de linhas em arquivo de dados de trabalho	1313
Tratamento de valor omisso	Definição de omisso	Os valores omissos definidos pelo usuário são tratados como omissos.

	Casos utilizados	As estatísticas para cada análise são baseadas nos casos sem dados omissos ou fora do intervalo para qualquer variável da análise.
Sintaxe		T-TEST GROUPS=RsPrgType(0 1)<br>/MISSING=ANALYSIS<br>/VARIABLES=Age DASS21_D DASS21_A DASS21_S PHQ9_Score BRCS_Score OLBI_D OLBI_E OLBI_Total Autonomy<br>PedagStruct<br>/CRITERIA=CI(.95).
Recursos	Tempo do processador	00:00:00,02
	Tempo decorrido	00:00:00,03

Estatísticas de grupo

	Type of residency program	N	Média	Erro Desvio	Erro padrão da média
Age	Non-Medical (Other HCP)	599	26,44	4,407	,180
	Medical	631	29,21	3,968	,158
DASS21 Depression Score	Non-Medical (Other HCP)	639	7,64	5,464	,216
	Medical	674	7,64	5,835	,225
DASS21 Anxietty Score	Non-Medical (Other HCP)	639	6,61	5,255	,208
	Medical	674	5,48	5,000	,193
DASS21 Stress Score	Non-Medical (Other HCP)	639	10,27	5,275	,209
	Medical	674	10,04	5,468	,211
PHQ9 Depression Score	Non-Medical (Other HCP)	639	12,31	6,277	,248
	Medical	674	11,71	6,605	,254
BRCS Score	Non-Medical (Other HCP)	639	12,26	3,654	,145
	Medical	674	12,56	3,882	,150
OLBI Disengagement Score	Non-Medical (Other HCP)	639	2,7365	,81289	,03216
	Medical	674	2,8095	,86305	,03324
OLBI Exhaustion Score	Non-Medical (Other HCP)	639	3,5808	,69267	,02740
	Medical	674	3,5298	,77501	,02985
OLBI Total Score	Non-Medical (Other HCP)	639	3,1586	,66095	,02615
	Medical	674	3,1698	,73517	,02832
Na sua opinião, qual o seu grau de autonomia para decidir condutas no trabalho? (EAV 1-10)	Non-Medical (Other HCP)	639	6,58	2,095	,083
	Medical	674	6,43	2,115	,081
Na sua opinião, qual o grau de	Non-Medical (Other HCP)	639	5,34	2,502	,099

adequação da organização pedagógica de seu programa de residência profissional? (EAV 1-10)	Medical	674	6,18	2,368	,091

Teste de amostras independentes

		Teste de Levene para igualdade de variâncias		teste-t para Igualdade de Médias						
									95% Intervalo de Confiança da Diferença	
		Z	Sig.	t	df	Sig. (2 extremidades)	Diferença média	Erro padrão de diferença	Inferior	Superior
Age	Variâncias iguais assumidas	1,093	,296	-11,583	1228	,000	-2,767	,239	-3,236	-2,298
	Variâncias iguais não assumidas			-11,551	1198,790	,000	-2,767	,240	-3,237	-2,297
DASS21 Depression Score	Variâncias iguais assumidas	4,245	,040	,022	1311	,983	,007	,312	-,606	,620
	Variâncias iguais não assumidas			,022	1310,797	,983	,007	,312	-,605	,619
DASS21 Anxietty Score	Variâncias iguais assumidas	1,973	,160	3,986	1311	,000	1,128	,283	,573	1,683
	Variâncias iguais não assumidas			3,981	1297,238	,000	1,128	,283	,572	1,684
DASS21 Stress Score	Variâncias iguais assumidas	1,084	,298	,757	1311	,449	,225	,297	-,358	,807
	Variâncias iguais não assumidas			,758	1310,599	,449	,225	,297	-,357	,806
PHQ9 Depression Score	Variâncias iguais assumidas	1,906	,168	1,687	1311	,092	,601	,356	-,098	1,299
	Variâncias iguais não assumidas			1,689	1310,992	,091	,601	,356	-,097	1,298
BRCS Score	Variâncias iguais assumidas	2,612	,106	-1,423	1311	,155	-,297	,208	-,705	,112
	Variâncias iguais não assumidas			-1,426	1310,932	,154	-,297	,208	-,705	,111
OLBI Disengagement Score	Variâncias iguais assumidas	3,509	,061	-1,576	1311	,115	-,07303	,04633	-,16391	,01785
	Variâncias iguais não assumidas			-1,579	1310,945	,115	-,07303	,04625	-,16377	,01771
OLBI Exhaustion Score	Variâncias iguais assumidas	7,257	,007	1,256	1311	,209	,05105	,04064	-,02868	,13078
	Variâncias iguais não assumidas			1,260	1306,487	,208	,05105	,04052	-,02844	,13055
OLBI Total Score	Variâncias iguais assumidas	7,200	,007	-,290	1311	,772	-,01120	,03865	-,08703	,06463
	Variâncias iguais não assumidas			-,291	1307,339	,771	-,01120	,03854	-,08681	,06441
Na sua opinião, qual o seu grau de autonomia para decidir condutas no trabalho? (EAV 1-10)	Variâncias iguais assumidas	,060	,806	1,308	1311	,191	,152	,116	-,076	,380
	Variâncias iguais não assumidas			1,308	1308,479	,191	,152	,116	-,076	,380
Na sua opinião, qual o grau de	Variâncias iguais assumidas	6,264	,012	-6,248	1311	,000	-,840	,134	-1,103	-,576

adequação da organização pedagógica de seu programa de residência profissional? (EAV 1-10)	Variâncias iguais não assumidas			-6,239	1295,838	,000	-,840	,135	-1,104	-,576

```
CORRELATIONS
  /VARIABLES=DASS21_D DASS21_A DASS21_S PHQ9_Score BRCS_Score OLBI_D OLBI_E OLBI_Total Autonomy
    PedagStruct PPEAvail_Classif ExternWork CovidCare Wkload_6060
  /PRINT=TWOTAIL NOSIG
  /MISSING=PAIRWISE.
```

Correlações

Observações

Saída criada		20-SEP-2020 12:30:35
Comentários		
Entrada	Dados	C:\Users\User\Documents\Pesquisa\Fellow\FellowGenData_V1.sav
	Conjunto de dados ativo	ConjuntodeDados1
	Filtro	<none>
	Ponderação	<none>
	Arquivo Dividido	<none>
	N de linhas em arquivo de dados de trabalho	1313
Tratamento de valor omisso	Definição de omisso	Os valores omissos definidos pelo usuário são tratados como omissos.
	Casos utilizados	As estatísticas para cada par de variáveis são baseadas em todos os casos com dados válidos para aquele par.
Sintaxe		CORRELATIONS /VARIABLES=DASS21_D DASS21_A DASS21_S PHQ9_Score BRCS_Score OLBI_D OLBI_E OLBI_Total Autonomy PedagStruct PPEAvail_Classif ExternWork CovidCare Wkload_6060 /PRINT=TWOTAIL NOSIG /MISSING=PAIRWISE.
Recursos	Tempo do processador	00:00:00,02
	Tempo decorrido	00:00:00,02

Correlações

		DASS21 Depression Score	DASS21 Anxietty Score	DASS21 Stress Score	PHQ9 Depression Score	BRCS Score	OLBI Disengagement Score	OLBI Exhaustion Score	OLBI Total Score	Na sua opinião, qual o seu grau de autonomia para decidir condutas no trabalho? (EAV 1-10)	Na sua opinião, qual o grau de adequação da organização pedagógica de seu programa de residência profissional? (EAV 1-10)	Perceived adequacy of the availability of personal protective equipment, when providing care for patients in the residency program	The participant exerts professional activity external to the residency program	The participant provides direct health care for COVID19 patients	Cumulative weekly workload < or >= 60h (dichotomous)
DASS21 Depression Score	Correlação de Pearson	1	,640**	,720**	,786**	-,442**	,569**	,545**	,628**	-,273**	-,333**	-,202**	-,032	,020	,044
	Sig. (2 extremidades)		,000	,000	,000	,000	,000	,000	,000	,000	,000	,000	,252	,471	,108
	N	1313	1313	1313	1313	1313	1313	1313	1313	1313	1313	1313	1313	1313	1313
DASS21 Anxietty Score	Correlação de Pearson	,640**	1	,748**	,649**	-,281**	,361**	,482**	,470**	-,160**	-,247**	-,163**	-,094**	,016	,017
	Sig. (2 extremidades)	,000		,000	,000	,000	,000	,000	,000	,000	,000	,000	,001	,553	,536
	N	1313	1313	1313	1313	1313	1313	1313	1313	1313	1313	1313	1313	1313	1313
DASS21 Stress Score	Correlação de Pearson	,720**	,748**	1	,731**	-,355**	,460**	,573**	,577**	-,202**	-,308**	-,178**	-,038	,026	,032
	Sig. (2 extremidades)	,000	,000		,000	,000	,000	,000	,000	,000	,000	,000	,173	,338	,246
	N	1313	1313	1313	1313	1313	1313	1313	1313	1313	1313	1313	1313	1313	1313
PHQ9 Depression Score	Correlação de Pearson	,786**	,649**	,731**	1	-,379**	,527**	,643**	,654**	-,255**	-,321**	-,178**	-,038	,048	,077**
	Sig. (2 extremidades)	,000	,000	,000		,000	,000	,000	,000	,000	,000	,000	,173	,079	,005
	N	1313	1313	1313	1313	1313	1313	1313	1313	1313	1313	1313	1313	1313	1313
BRCS Score	Correlação de Pearson	-,442**	-,281**	-,355**	-,379**	1	-,406**	-,366**	-,435**	,244**	,227**	,098**	,061*	,019	,038
	Sig. (2 extremidades)	,000	,000	,000	,000		,000	,000	,000	,000	,000	,000	,027	,499	,173
	N	1313	1313	1313	1313	1313	1313	1313	1313	1313	1313	1313	1313	1313	1313
OLBI Disengagement Score	Correlação de Pearson	,569**	,361**	,460**	,527**	-,406**	1	,576**	,903**	-,329**	-,439**	-,191**	,029	,014	,031
	Sig. (2 extremidades)	,000	,000	,000	,000	,000		,000	,000	,000	,000	,000	,301	,612	,257
	N	1313	1313	1313	1313	1313	1313	1313	1313	1313	1313	1313	1313	1313	1313
OLBI Exhaustion Score	Correlação de Pearson	,545**	,482**	,573**	,643**	-,366**	,576**	1	,871**	-,262**	-,347**	-,154**	-,038	,057*	,108**
	Sig. (2 extremidades)	,000	,000	,000	,000	,000	,000		,000	,000	,000	,000	,172	,040	,000
	N	1313	1313	1313	1313	1313	1313	1313	1313	1313	1313	1313	1313	1313	1313
OLBI Total Score	Correlação de Pearson	,628**	,470**	,577**	,654**	-,435**	,903**	,871**	1	-,335**	-,446**	-,196**	-,003	,038	,075**
	Sig. (2 extremidades)	,000	,000	,000	,000	,000	,000	,000		,000	,000	,000	,924	,166	,006
	N	1313	1313	1313	1313	1313	1313	1313	1313	1313	1313	1313	1313	1313	1313
Na sua opinião, qual o seu grau de autonomia para decidir condutas no trabalho? (EAV 1-10)	Correlação de Pearson	-,273**	-,160**	-,202**	-,255**	,244**	-,329**	-,262**	-,335**	1	,320**	,112**	,007	-,023	-,022
	Sig. (2 extremidades)	,000	,000	,000	,000	,000	,000	,000	,000		,000	,000	,788	,399	,418
	N	1313	1313	1313	1313	1313	1313	1313	1313	1313	1313	1313	1313	1313	1313
Na sua opinião, qual o grau de adequação da organização pedagógica de seu programa de residência profissional? (EAV 1-10)	Correlação de Pearson	-,333**	-,247**	-,308**	-,321**	,227**	-,439**	-,347**	-,446**	,320**	1	,155**	,127**	,038	,004
	Sig. (2 extremidades)	,000	,000	,000	,000	,000	,000	,000	,000	,000		,000	,000	,173	,880
	N	1313	1313	1313	1313	1313	1313	1313	1313	1313	1313	1313	1313	1313	1313
Perceived adequacy of the	Correlação de Pearson	-,202**	-,163**	-,178**	-,178**	,098**	-,191**	-,154**	-,196**	,112**	,155**	1	-,017	-,041	-,052

availability of personal protective equipment, when providing care for patients in the residency program	Sig. (2 extremidades)	,000	,000	,000	,000	,000	,000	,000	,000	,000	,000		,540	,133	,060
	N	1313	1313	1313	1313	1313	1313	1313	1313	1313	1313	1313	1313	1313	1313
The participant exerts professional activity external to the residency program	Correlação de Pearson	-,032	-,094**	-,038	-,038	,061*	,029	-,038	-,003	,007	,127**	-,017	1	,362**	,257**
	Sig. (2 extremidades)	,252	,001	,173	,173	,027	,301	,172	,924	,788	,000	,540		,000	,000
	N	1313	1313	1313	1313	1313	1313	1313	1313	1313	1313	1313	1313	1313	1313
The participant provides direct health care for COVID19 patients	Correlação de Pearson	,020	,016	,026	,048	,019	,014	,057*	,038	-,023	,038	-,041	,362**	1	,166**
	Sig. (2 extremidades)	,471	,553	,338	,079	,499	,612	,040	,166	,399	,173	,133	,000		,000
	N	1313	1313	1313	1313	1313	1313	1313	1313	1313	1313	1313	1313	1313	1313
Cumulative weekly workload < or >= 60h (dichotomous)	Correlação de Pearson	,044	,017	,032	,077**	,038	,031	,108**	,075**	-,022	,004	-,052	,257**	,166**	1
	Sig. (2 extremidades)	,108	,536	,246	,005	,173	,257	,000	,006	,418	,880	,060	,000	,000	
	N	1313	1313	1313	1313	1313	1313	1313	1313	1313	1313	1313	1313	1313	1313

**. A correlação é significativa no nível 0,01 (2 extremidades).

*. A correlação é significativa no nível 0,05 (2 extremidades).

```
NONPAR CORR
  /VARIABLES=DASS21_D DASS21_A DASS21_S PHQ9_Score BRCS_Score OLBI_D OLBI_E OLBI_Total Autonomy
    PedagStruct PPEAvail_Classif ExternWork CovidCare Wkload_6060
  /PRINT=SPEARMAN TWOTAIL NOSIG
  /MISSING=PAIRWISE.
```

Correlações não paramétricas

Observações

Saída criada		20-SEP-2020 12:30:35
Comentários		
Entrada	Dados	C:\Users\User\Documents\Pesquisa\Fellow\FellowGenData_V1.sav
	Conjunto de dados ativo	ConjuntodeDados1
	Filtro	<none>
	Ponderação	<none>
	Arquivo Dividido	<none>
	N de linhas em arquivo de dados de trabalho	1313
Tratamento de valor omisso	Definição de omisso	Os valores omissos definidos pelo usuário são tratados como omissos.
	Casos utilizados	As estatísticas para cada par de variáveis são baseadas em todos os casos com dados válidos para aquele par.

Sintaxe		NONPAR CORR<br>/VARIABLES=DASS21_D DASS21_A DASS21_S PHQ9_Score BRCS_Score OLBI_D OLBI_E OLBI_Total Autonomy<br>PedagStruct PPEAvail_Classif ExternWork CovidCare Wkload_6060<br>/PRINT=SPEARMAN TWOTAIL NOSIG<br>/MISSING=PAIRWISE.
Recursos	Tempo do processador	00:00:00,03
	Tempo decorrido	00:00:00,02
	Número de Casos Permitidos	185042 casos[a]

a. Baseado na disponibilidade de memória da área de trabalho

Correlações

			DASS21 Depression Score	DASS21 Anxietty Score	DASS21 Stress Score	PHQ9 Depression Score	BRCS Score	OLBI Disengagement Score	OLBI Exhaustion Score	OLBI Total Score	Na sua opinião, qual o seu grau de autonomia para decidir condutas no trabalho? (EAV 1-10)	Na sua opinião, qual o grau de adequação da organização pedagógica de seu programa de residência profissional? (EAV 1-10)	Perceived adequacy of the availability of personal protective equipment, when providing care for patients in the residency program	The participant exerts professional activity external to the residency program	The participant provides direct health care for COVID19 patients	Cumulative weekly workload < or >= 60h (dichotomous)
rô de Spearman	DASS21 Depression Score	Coeficiente de Correlação	1,000	,627**	,717**	,782**	-,448**	,571**	,560**	,636**	-,260**	-,335**	-,192**	-,043	,018	,042
		Sig. (2 extremidades)	.	,000	,000	,000	,000	,000	,000	,000	,000	,000	,000	,120	,518	,126
		N	1313	1313	1313	1313	1313	1313	1313	1313	1313	1313	1313	1313	1313	1313
	DASS21 Anxietty Score	Coeficiente de Correlação	,627**	1,000	,749**	,653**	-,276**	,360**	,495**	,474**	-,138**	-,243**	-,154**	-,103**	,015	,013
		Sig. (2 extremidades)	,000	.	,000	,000	,000	,000	,000	,000	,000	,000	,000	,000	,599	,637
		N	1313	1313	1313	1313	1313	1313	1313	1313	1313	1313	1313	1313	1313	1313
	DASS21 Stress Score	Coeficiente de Correlação	,717**	,749**	1,000	,723**	-,337**	,446**	,562**	,560**	-,198**	-,296**	-,175**	-,039	,029	,036
		Sig. (2 extremidades)	,000	,000	.	,000	,000	,000	,000	,000	,000	,000	,000	,159	,293	,197
		N	1313	1313	1313	1313	1313	1313	1313	1313	1313	1313	1313	1313	1313	1313
	PHQ9 Depression Score	Coeficiente de Correlação	,782**	,653**	,723**	1,000	-,376**	,520**	,646**	,648**	-,237**	-,318**	-,174**	-,038	,050	,076**
		Sig. (2 extremidades)	,000	,000	,000	.	,000	,000	,000	,000	,000	,000	,000	,173	,072	,006
		N	1313	1313	1313	1313	1313	1313	1313	1313	1313	1313	1313	1313	1313	1313
	BRCS Score	Coeficiente de Correlação	-,448**	-,276**	-,337**	-,376**	1,000	-,394**	-,352**	-,418**	,239**	,218**	,097**	,068*	,019	,039
		Sig. (2 extremidades)	,000	,000	,000	,000	.	,000	,000	,000	,000	,000	,000	,014	,484	,163
		N	1313	1313	1313	1313	1313	1313	1313	1313	1313	1313	1313	1313	1313	1313
	OLBI Disengagement Score	Coeficiente de Correlação	,571**	,360**	,446**	,520**	-,394**	1,000	,572**	,905**	-,299**	-,437**	-,187**	,025	,015	,035
		Sig. (2 extremidades)	,000	,000	,000	,000	,000	.	,000	,000	,000	,000	,000	,373	,586	,200
		N	1313	1313	1313	1313	1313	1313	1313	1313	1313	1313	1313	1313	1313	1313

OLBI Exhaustion Score	Coeficiente de Correlação	,560**	,495**	,562**	,646**	-,352**	,572**	1,000	,857**	-,257**	-,345**	-,159**	-,022	,064*	,106**
	Sig. (2 extremidades)	,000	,000	,000	,000	,000	,000	.	,000	,000	,000	,000	,420	,021	,000
	N	1313	1313	1313	1313	1313	1313	1313	1313	1313	1313	1313	1313	1313	1313
OLBI Total Score	Coeficiente de Correlação	,636**	,474**	,560**	,648**	-,418**	,905**	,857**	1,000	-,312**	-,442**	-,195**	,003	,042	,079**
	Sig. (2 extremidades)	,000	,000	,000	,000	,000	,000	,000	.	,000	,000	,000	,922	,125	,004
	N	1313	1313	1313	1313	1313	1313	1313	1313	1313	1313	1313	1313	1313	1313
Na sua opinião, qual o seu grau de autonomia para decidir condutas no trabalho? (EAV 1-10)	Coeficiente de Correlação	-,260**	-,138**	-,198**	-,237**	,239**	-,299**	-,257**	-,312**	1,000	,303**	,104**	,027	-,028	-,029
	Sig. (2 extremidades)	,000	,000	,000	,000	,000	,000	,000	,000	.	,000	,000	,328	,308	,299
	N	1313	1313	1313	1313	1313	1313	1313	1313	1313	1313	1313	1313	1313	1313
Na sua opinião, qual o grau de adequação da organização pedagógica de seu programa de residência profissional? (EAV 1-10)	Coeficiente de Correlação	-,335**	-,243**	-,296**	-,318**	,218**	-,437**	-,345**	-,442**	,303**	1,000	,154**	,134**	,041	,003
	Sig. (2 extremidades)	,000	,000	,000	,000	,000	,000	,000	,000	,000	.	,000	,000	,139	,915
	N	1313	1313	1313	1313	1313	1313	1313	1313	1313	1313	1313	1313	1313	1313
Perceived adequacy of the availability of personal protective equipment, when providing care for patients in the residency program	Coeficiente de Correlação	-,192**	-,154**	-,175**	-,174**	,097**	-,187**	-,159**	-,195**	,104**	,154**	1,000	-,017	-,041	-,052
	Sig. (2 extremidades)	,000	,000	,000	,000	,000	,000	,000	,000	,000	,000	.	,540	,133	,060
	N	1313	1313	1313	1313	1313	1313	1313	1313	1313	1313	1313	1313	1313	1313
The participant exerts professional activity external to the residency program	Coeficiente de Correlação	-,043	-,103**	-,039	-,038	,068*	,025	-,022	,003	,027	,134**	-,017	1,000	,362**	,257**
	Sig. (2 extremidades)	,120	,000	,159	,173	,014	,373	,420	,922	,328	,000	,540	.	,000	,000
	N	1313	1313	1313	1313	1313	1313	1313	1313	1313	1313	1313	1313	1313	1313
The participant provides direct health care for COVID19 patients	Coeficiente de Correlação	,018	,015	,029	,050	,019	,015	,064*	,042	-,028	,041	-,041	,362**	1,000	,166**
	Sig. (2 extremidades)	,518	,599	,293	,072	,484	,586	,021	,125	,308	,139	,133	,000	.	,000
	N	1313	1313	1313	1313	1313	1313	1313	1313	1313	1313	1313	1313	1313	1313
Cumulative weekly workload < or >= 60h (dichotomous)	Coeficiente de Correlação	,042	,013	,036	,076**	,039	,035	,106**	,079**	-,029	,003	-,052	,257**	,166**	1,000
	Sig. (2 extremidades)	,126	,637	,197	,006	,163	,200	,000	,004	,299	,915	,060	,000	,000	.
	N	1313	1313	1313	1313	1313	1313	1313	1313	1313	1313	1313	1313	1313	1313

**. A correlação é significativa no nível 0,01 (2 extremidades).

*. A correlação é significativa no nível 0,05 (2 extremidades).

```
LOGISTIC REGRESSION VARIABLES DASS21_Classif_D2c
  /METHOD=ENTER ComorbAny BRCS_Score Autonomy PedagStruct PPEAvail_Classif Wkload_9090
  /SAVE=COOK ZRESID
  /CASEWISE OUTLIER(3)
  /PRINT=GOODFIT CI(95)
  /CRITERIA=PIN(0.05) POUT(0.10) ITERATE(20) CUT(0.5).
```

Regressão logística

Observações

Saída criada	20-SEP-2020 14:27:41
Comentários	

Entrada	Dados	C:\Users\User\Documents\Pesquisa\Fellow\FellowGenData_V1.sav
	Conjunto de dados ativo	ConjuntodeDados1
	Filtro	<none>
	Ponderação	<none>
	Arquivo Dividido	<none>
	N de linhas em arquivo de dados de trabalho	1313
Tratamento de valor omisso	Definição de omisso	Os valores omissos definidos pelo usuário são tratados como omissos
Sintaxe		LOGISTIC REGRESSION VARIABLES DASS21_Classif_D2c /METHOD=ENTER ComorbAny BRCS_Score Autonomy PedagStruct PPEAvail_Classif Wkload_9090 /SAVE=COOK ZRESID /CASEWISE OUTLIER(3) /PRINT=GOODFIT CI(95) /CRITERIA=PIN(0.05) POUT(0.10) ITERATE(20) CUT(0.5).
Recursos	Tempo do processador	00:00:00,05
	Tempo decorrido	00:00:00,04
Variáveis Criadas ou Modificadas	COO_5	Análogo às estatísticas de influência de Cook
	ZRE_5	Resíduo normalizado

Resumo de processamento do caso

Casos não ponderados[a]		N	Porcentagem
Casos selecionados	Incluído na análise	1305	99,4
	Casos omissos	8	,6
	Total	1313	100,0
Casos não selecionados		0	,0
Total		1313	100,0

a. Se a ponderação estiver em vigor, veja a tabela de classificação para o número total de casos.

Codificação de variável dependente

Valor original	Valor interno
Normal	0
Abnormal (elevated)	1

Bloco 0: Bloco Inicial

Tabela de Classificação[a,b]

Observado			Previsto: DASS21 Classification - Depression (dichotomous): Normal	Previsto: DASS21 Classification - Depression (dichotomous): Abnormal (elevated)	Previsto: Porcentagem correta
Passo 0	DASS21 Classification - Depression (dichotomous)	Normal	865	0	100,0
		Abnormal (elevated)	440	0	,0
	Porcentagem global				66,3

a. A constante está incluída no modelo.

b. O valor de recorte é ,500

Variáveis na equação

		B	S.E.	Wald	df	Sig.	Exp(B)
Passo 0	Constante	-,676	,059	133,258	1	,000	,509

Variáveis não presentes na equação

			Escore	df	Sig.
Passo 0	Variáveis	Any comorbidity (regardless COVID risk)	37,473	1	,000
		BRCS Score	140,405	1	,000
		Na sua opinião, qual o seu grau de autonomia para decidir condutas no trabalho? (EAV 1-10)	62,560	1	,000
		Na sua opinião, qual o grau de adequação da organização pedagógica de seu programa de residência profissional? (EAV 1-10)	68,495	1	,000
		Perceived adequacy of the availability of personal protective equipment, when providing care for patients in the residency program	52,915	1	,000
		Cumulative weekly workload < or >= 90h (dichotomous)	9,957	1	,002
	Estatísticas globais		244,219	6	,000

Bloco 1: Método = Enter

Testes de Omnibus do Modelo de Coeficientes

		Qui-quadrado	df	Sig.
Passo 1	Passo	262,400	6	,000
	Bloco	262,400	6	,000
	Modelo	262,400	6	,000

Resumo do modelo

Passo	Verossimilhança de log -2	R quadrado Cox & Snell	R quadrado Nagelkerke
1	1405,747[a]	,182	,252

a. Estimação finalizada no número de iteração 5 porque as estimativas de parâmetro mudaram foram alteradas para menos de ,001.

Teste de Hosmer e Lemeshow

Passo	Qui-quadrado	df	Sig.
1	12,270	8	,140

Tabela de contingência para teste de Hosmer e Lemeshow

		DASS21 Classification - Depression (dichotomous) = Normal		DASS21 Classification - Depression (dichotomous) = Abnormal (elevated)		
		Observado	Esperado	Observado	Esperado	Total
Passo 1	1	123	121,455	8	9,545	131
	2	118	114,441	13	16,559	131
	3	106	109,643	26	22,357	132
	4	102	103,807	30	28,193	132
	5	86	96,419	45	34,581	131
	6	95	87,916	36	43,084	131
	7	75	78,778	56	52,222	131
	8	77	68,229	54	62,771	131
	9	59	55,463	72	75,537	131
	10	24	28,849	100	95,151	124

Tabela de Classificação[a]

Observado	Previsto: DASS21 Classification - Depression (dichotomous)	Porcentagem correta

			Normal	Abnormal (elevated)	
Passo 1	DASS21 Classification - Depression (dichotomous)	Normal	760	105	87,9
		Abnormal (elevated)	255	185	42,0
	Porcentagem global				72,4

a. O valor de recorte é ,500

Variáveis na equação

		B	S.E.	Wald	df	Sig.	Exp(B)	95% C.I. para EXP(B)	
								Inferior	Superior
Passo 1[a]	Any comorbidity (regardless COVID risk)	,880	,163	29,100	1	,000	2,411	1,751	3,319
	BRCS Score	-,179	,019	86,554	1	,000	,836	,805	,868
	Na sua opinião, qual o seu grau de autonomia para decidir condutas no trabalho? (EAV 1-10)	-,107	,032	10,889	1	,001	,898	,843	,957
	Na sua opinião, qual o grau de adequação da organização pedagógica de seu programa de residência profissional? (EAV 1-10)	-,110	,028	15,419	1	,000	,896	,848	,946
	Perceived adequacy of the availability of personal protective equipment, when providing care for patients in the residency program	-,798	,153	27,286	1	,000	,450	,334	,607
	Cumulative weekly workload < or >= 90h (dichotomous)	,537	,251	4,587	1	,032	1,711	1,047	2,798
	Constante	3,171	,315	101,132	1	,000	23,836		

a. Variável(is) inserida(s) no passo 1: Any comorbidity (regardless COVID risk), BRCS Score, Na sua opinião, qual o seu grau de autonomia para decidir condutas no trabalho? (EAV 1-10), Na sua opinião, qual o grau de adequação da organização pedagógica de seu programa de residência profissional? (EAV 1-10), Perceived adequacy of the availability of personal protective equipment, when providing care for patients in the residency program, Cumulative weekly workload < or >= 90h (dichotomous).

Lista entre casos[a]

a. O plot entre casos não é produzido porque nenhum valor discrepante foi encontrado.

```
LOGISTIC REGRESSION VARIABLES DASS21_Classif_A2c
  /METHOD=ENTER Sex ComorbAny BRCS_Score Autonomy PedagStruct PPEAvail_Classif Wkload_9090
    ExternWork UnivHosp RsPrgType
  /SAVE=COOK ZRESID
  /CASEWISE OUTLIER(3)
```

/PRINT=GOODFIT CI(95)
/CRITERIA=PIN(0.05) POUT(0.10) ITERATE(20) CUT(0.5).

Regressão logística

Observações

Saída criada		20-SEP-2020 18:33:29
Comentários		
Entrada	Dados	C:\Users\User\Documents\Pesquisa\Fellow\FellowGenData_V1.sav
	Conjunto de dados ativo	ConjuntodeDados1
	Filtro	<none>
	Ponderação	<none>
	Arquivo Dividido	<none>
	N de linhas em arquivo de dados de trabalho	1313
Tratamento de valor omisso	Definição de omisso	Os valores omissos definidos pelo usuário são tratados como omissos
Sintaxe		LOGISTIC REGRESSION VARIABLES DASS21_Classif_A2c /METHOD=ENTER Sex ComorbAny BRCS_Score Autonomy PedagStruct PPEAvail_Classif Wkload_9090 ExternWork UnivHosp RsPrgType /SAVE=COOK ZRESID /CASEWISE OUTLIER(3) /PRINT=GOODFIT CI(95) /CRITERIA=PIN(0.05) POUT(0.10) ITERATE(20) CUT(0.5).
Recursos	Tempo do processador	00:00:00,03
	Tempo decorrido	00:00:00,04
Variáveis Criadas ou Modificadas	COO_8	Análogo às estatísticas de influência de Cook
	ZRE_8	Resíduo normalizado

Resumo de processamento do caso

Casos não ponderados[a]		N	Porcentagem
Casos selecionados	Incluído na análise	1302	99,2
	Casos omissos	11	,8

	Total	1313	100,0
Casos não selecionados		0	,0
Total		1313	100,0

a. Se a ponderação estiver em vigor, veja a tabela de classificação para o número total de casos.

Codificação de variável dependente

Valor original	Valor interno
Normal	0
Abnormal (elevated)	1

Bloco 0: Bloco Inicial

Tabela de Classificação[a,b]

	Observado		Previsto: DASS21 Classification - Anxiety (dichotomous) Normal	Previsto: DASS21 Classification - Anxiety (dichotomous) Abnormal (elevated)	Previsto: Porcentagem correta
Passo 0	DASS21 Classification - Anxiety (dichotomous)	Normal	854	0	100,0
		Abnormal (elevated)	448	0	,0
	Porcentagem global				65,6

a. A constante está incluída no modelo.

b. O valor de recorte é ,500

Variáveis na equação

		B	S.E.	Wald	df	Sig.	Exp(B)
Passo 0	Constante	-,645	,058	122,301	1	,000	,525

Variáveis não presentes na equação

			Escore	df	Sig.
Passo 0	Variáveis	Sex	9,736	1	,002
		Any comorbidity (regardless COVID risk)	19,777	1	,000
		BRCS Score	63,779	1	,000
		Na sua opinião, qual o seu grau de autonomia para decidir condutas no trabalho? (EAV 1-10)	20,735	1	,000

		Na sua opinião, qual o grau de adequação da organização pedagógica de seu programa de residência profissional? (EAV 1-10)	54,934	1	,000
		Perceived adequacy of the availability of personal protective equipment, when providing care for patients in the residency program	23,903	1	,000
		Cumulative weekly workload < or >= 90h (dichotomous)	,056	1	,812
		The participant exerts professional activity external to the residency program	6,135	1	,013
		The institution is a university hospital	5,419	1	,020
		Type of residency program	6,524	1	,011
	Estatísticas globais		141,664	10	,000

```
Bloco 1: Método = Enter
```

Testes de Omnibus do Modelo de Coeficientes

		Qui-quadrado	df	Sig.
Passo 1	Passo	148,581	10	,000
	Bloco	148,581	10	,000
	Modelo	148,581	10	,000

Resumo do modelo

Passo	Verossimilhança de log -2	R quadrado Cox & Snell	R quadrado Nagelkerke
1	1527,636[a]	,108	,149

a. Estimação finalizada no número de iteração 4 porque as estimativas de parâmetro mudaram foram alteradas para menos de ,001.

Teste de Hosmer e Lemeshow

Passo	Qui-quadrado	df	Sig.
1	4,217	8	,837

Tabela de contingência para teste de Hosmer e Lemeshow

		DASS21 Classification - Anxiety (dichotomous) = Normal		DASS21 Classification - Anxiety (dichotomous) = Abnormal (elevated)		
		Observado	Esperado	Observado	Esperado	Total
Passo 1	1	111	114,846	19	15,154	130
	2	108	106,895	22	23,105	130
	3	100	101,053	30	28,947	130
	4	101	95,949	29	34,051	130
	5	89	90,829	41	39,171	130
	6	82	85,143	48	44,857	130
	7	85	79,177	45	50,823	130
	8	73	72,298	58	58,702	131
	9	59	62,579	71	67,421	130
	10	46	45,229	85	85,771	131

Tabela de Classificação[a]

			Previsto		
			DASS21 Classification - Anxiety (dichotomous)		
	Observado		Normal	Abnormal (elevated)	Porcentagem correta
Passo 1	DASS21 Classification - Anxiety (dichotomous)	Normal	771	83	90,3
		Abnormal (elevated)	315	133	29,7
	Porcentagem global				69,4

a. O valor de recorte é ,500

Variáveis na equação

		B	S.E.	Wald	df	Sig.	Exp(B)	95% C.I. para EXP(B)	
								Inferior	Superior
Passo 1[a]	Sex	,537	,164	10,722	1	,001	1,711	1,240	2,359
	Any comorbidity (regardless COVID risk)	,621	,156	15,882	1	,000	1,860	1,371	2,524
	BRCS Score	-,107	,018	36,274	1	,000	,899	,868	,931
	Na sua opinião, qual o seu grau de autonomia para decidir condutas no trabalho? (EAV 1-10)	-,034	,031	1,197	1	,274	,966	,909	1,027
	Na sua opinião, qual o grau de adequação da organização pedagógica de seu programa de residência profissional? (EAV 1-10)	-,121	,027	19,666	1	,000	,886	,840	,935

Perceived adequacy of the availability of personal protective equipment, when providing care for patients in the residency program	-,522	,148	12,462	1	,000	,593	,444	,793
Cumulative weekly workload < or >= 90h (dichotomous)	-,005	,259	,000	1	,985	,995	,599	1,655
The participant exerts professional activity external to the residency program	-,066	,178	,137	1	,711	,936	,660	1,327
The institution is a university hospital	-,495	,197	6,302	1	,012	,609	,414	,897
Type of residency program	-,151	,167	,819	1	,366	,860	,620	1,192
Constante	1,939	,371	27,271	1	,000	6,951		

a. Variável(is) inserida(s) no passo 1: Sex, Any comorbidity (regardless COVID risk), BRCS Score, Na sua opinião, qual o seu grau de autonomia para decidir condutas no trabalho? (EAV 1-10), Na sua opinião, qual o grau de adequação da organização pedagógica de seu programa de residência profissional? (EAV 1-10), Perceived adequacy of the availability of personal protective equipment, when providing care for patients in the residency program, Cumulative weekly workload < or >= 90h (dichotomous), The participant exerts professional activity external to the residency program, The institution is a university hospital, Type of residency program.

Lista entre casos[a]

a. O plot entre casos não é produzido porque nenhum valor discrepante foi encontrado.

```
LOGISTIC REGRESSION VARIABLES PHQ9_Classif
  /METHOD=ENTER Sex ComorbAny BRCS_Score Autonomy PedagStruct PPEAvail_Classif Wkload_6060 UnivHosp
  /SAVE=COOK ZRESID
  /CASEWISE OUTLIER(3)
  /PRINT=GOODFIT CI(95)
  /CRITERIA=PIN(0.05) POUT(0.10) ITERATE(20) CUT(0.5).
```

Regressão logística

Observações

Saída criada		20-SEP-2020 18:47:55
Comentários		
Entrada	Dados	C:\Users\User\Documents\Pesquisa\Fellow\FellowGenData_V1.sav
	Conjunto de dados ativo	ConjuntodeDados1
	Filtro	<none>
	Ponderação	<none>

	Arquivo Dividido	<none>
	N de linhas em arquivo de dados de trabalho	1313
Tratamento de valor omisso	Definição de omisso	Os valores omissos definidos pelo usuário são tratados como omissos
Sintaxe		LOGISTIC REGRESSION VARIABLES PHQ9_Classif /METHOD=ENTER Sex ComorbAny BRCS_Score Autonomy PedagStruct PPEAvail_Classif Wkload_6060 UnivHosp /SAVE=COOK ZRESID /CASEWISE OUTLIER(3) /PRINT=GOODFIT CI(95) /CRITERIA=PIN(0.05) POUT(0.10) ITERATE(20) CUT(0.5).
Recursos	Tempo do processador	00:00:00,05
	Tempo decorrido	00:00:00,04
Variáveis Criadas ou Modificadas	COO_10	Análogo às estatísticas de influência de Cook
	ZRE_10	Resíduo normalizado

Resumo de processamento do caso

Casos não ponderados[a]		N	Porcentagem
Casos selecionados	Incluído na análise	1302	99,2
	Casos omissos	11	,8
	Total	1313	100,0
Casos não selecionados		0	,0
Total		1313	100,0

a. Se a ponderação estiver em vigor, veja a tabela de classificação para o número total de casos.

Codificação de variável dependente

Valor original	Valor interno
Low	0
High	1

Bloco 0: Bloco Inicial

Tabela de Classificação[a,b]

Observado			Previsto: PHQ9 Depression Classification - Risk estimate for having current major depressive disorder: Low	Previsto: PHQ9 Depression Classification - Risk estimate for having current major depressive disorder: High	Previsto: Porcentagem correta
Passo 0	PHQ9 Depression Classification - Risk estimate for having current major depressive disorder	Low	0	427	,0
		High	0	875	100,0
	Porcentagem global				67,2

a. A constante está incluída no modelo.

b. O valor de recorte é ,500

Variáveis na equação

		B	S.E.	Wald	df	Sig.	Exp(B)
Passo 0	Constante	,717	,059	147,705	1	,000	2,049

Variáveis não presentes na equação

			Escore	df	Sig.
Passo 0	Variáveis	Sex	11,357	1	,001
		Any comorbidity (regardless COVID risk)	22,996	1	,000
		BRCS Score	123,917	1	,000
		Na sua opinião, qual o seu grau de autonomia para decidir condutas no trabalho? (EAV 1-10)	32,001	1	,000
		Na sua opinião, qual o grau de adequação da organização pedagógica de seu programa de residência profissional? (EAV 1-10)	82,501	1	,000
		Perceived adequacy of the availability of personal protective equipment, when providing care for patients in the residency program	22,835	1	,000
		Cumulative weekly workload < or >= 60h (dichotomous)	5,687	1	,017
		The institution is a university hospital	7,943	1	,005
	Estatísticas globais		220,896	8	,000

```
Bloco 1: Método = Enter
```

Testes de Omnibus do Modelo de Coeficientes

		Qui-quadrado	df	Sig.
Passo 1	Passo	245,760	8	,000
	Bloco	245,760	8	,000
	Modelo	245,760	8	,000

Resumo do modelo

Passo	Verossimilhança de log -2	R quadrado Cox & Snell	R quadrado Nagelkerke
1	1401,849[a]	,172	,240

a. Estimação finalizada no número de iteração 5 porque as estimativas de parâmetro mudaram foram alteradas para menos de ,001.

Teste de Hosmer e Lemeshow

Passo	Qui-quadrado	df	Sig.
1	5,545	8	,698

Tabela de contingência para teste de Hosmer e Lemeshow

		PHQ9 Depression Classification - Risk estimate for having current major depressive disorder = Low		PHQ9 Depression Classification - Risk estimate for having current major depressive disorder = High		
		Observado	Esperado	Observado	Esperado	Total
Passo 1	1	96	93,549	34	36,451	130
	2	69	72,482	61	57,518	130
	3	58	60,017	72	69,983	130
	4	50	50,746	80	79,254	130
	5	39	42,295	91	87,705	130
	6	38	34,778	92	95,222	130
	7	36	28,039	94	101,961	130
	8	20	22,029	110	107,971	130
	9	16	15,562	114	114,438	130
	10	5	7,503	127	124,497	132

Tabela de Classificação[a]

	Observado	Previsto

			PHQ9 Depression Classification - Risk estimate for having current major depressive disorder		Porcentagem
			Low	High	correta
Passo 1	PHQ9 Depression Classification - Risk estimate for having current major depressive disorder	Low	169	258	39,6
		High	100	775	88,6
	Porcentagem global				72,5

a. O valor de recorte é ,500

Variáveis na equação

		B	S.E.	Wald	df	Sig.	Exp(B)	95% C.I. para EXP(B)	
								Inferior	Superior
Passo 1[a]	Sex	,613	,157	15,256	1	,000	1,846	1,357	2,512
	Any comorbidity (regardless COVID risk)	,806	,192	17,647	1	,000	2,238	1,537	3,259
	BRCS Score	-,169	,019	78,619	1	,000	,845	,814	,877
	Na sua opinião, qual o seu grau de autonomia para decidir condutas no trabalho? (EAV 1-10)	-,043	,035	1,490	1	,222	,958	,895	1,026
	Na sua opinião, qual o grau de adequação da organização pedagógica de seu programa de residência profissional? (EAV 1-10)	-,186	,030	38,940	1	,000	,830	,783	,880
	Perceived adequacy of the availability of personal protective equipment, when providing care for patients in the residency program	-,528	,175	9,065	1	,003	,590	,418	,832
	Cumulative weekly workload < or >= 60h (dichotomous)	,386	,133	8,399	1	,004	1,471	1,133	1,909
	The institution is a university hospital	-,679	,234	8,399	1	,004	,507	,320	,803
	Constante	4,510	,442	104,096	1	,000	90,912		

a. Variável(is) inserida(s) no passo 1: Sex, Any comorbidity (regardless COVID risk), BRCS Score, Na sua opinião, qual o seu grau de autonomia para decidir condutas no trabalho? (EAV 1-10), Na sua opinião, qual o grau de adequação da organização pedagógica de seu programa de residência profissional? (EAV 1-10), Perceived adequacy of the availability of personal protective equipment, when providing care for patients in the residency program, Cumulative weekly workload < or >= 60h (dichotomous), The institution is a university hospital.

Lista entre casos[a]

a. O plot entre casos não é produzido porque nenhum valor discrepante foi encontrado.

```
LOGISTIC REGRESSION VARIABLES DASS21_Classif_S2c
  /METHOD=ENTER Sex ComorbAny BRCS_Score Autonomy PedagStruct PPEAvail_Classif UnivHosp Wkload_9090
  /SAVE=COOK ZRESID
  /CASEWISE OUTLIER(3)
  /PRINT=GOODFIT CI(95)
  /CRITERIA=PIN(0.05) POUT(0.10) ITERATE(20) CUT(0.5).
```

Regressão logística

Observações

Saída criada		20-SEP-2020 18:51:01
Comentários		
Entrada	Dados	C:\Users\User\Documents\Pesquisa\Fellow\FellowGenData_V1.sav
	Conjunto de dados ativo	ConjuntodeDados1
	Filtro	<none>
	Ponderação	<none>
	Arquivo Dividido	<none>
	N de linhas em arquivo de dados de trabalho	1313
Tratamento de valor omisso	Definição de omisso	Os valores omissos definidos pelo usuário são tratados como omissos
Sintaxe		LOGISTIC REGRESSION VARIABLES DASS21_Classif_S2c /METHOD=ENTER Sex ComorbAny BRCS_Score Autonomy PedagStruct PPEAvail_Classif UnivHosp Wkload_9090 /SAVE=COOK ZRESID /CASEWISE OUTLIER(3) /PRINT=GOODFIT CI(95) /CRITERIA=PIN(0.05) POUT(0.10) ITERATE(20) CUT(0.5).
Recursos	Tempo do processador	00:00:00,03
	Tempo decorrido	00:00:00,04
Variáveis Criadas ou Modificadas	COO_11	Análogo às estatísticas de influência de Cook
	ZRE_11	Resíduo normalizado

Resumo de processamento do caso

Casos não ponderados[a]		N	Porcentagem
Casos selecionados	Incluído na análise	1302	99,2
	Casos omissos	11	,8
	Total	1313	100,0
Casos não selecionados		0	,0
Total		1313	100,0

a. Se a ponderação estiver em vigor, veja a tabela de classificação para o número total de casos.

Codificação de variável dependente

Valor original	Valor interno
Normal	0
Abnormal (elevated)	1

```
Bloco 0: Bloco Inicial
```

Tabela de Classificação[a,b]

	Observado		Previsto: DASS21 Classification - Stress (dichotomous): Normal	Previsto: DASS21 Classification - Stress (dichotomous): Abnormal (elevated)	Previsto: Porcentagem correta
Passo 0	DASS21 Classification - Stress (dichotomous)	Normal	1004	0	100,0
		Abnormal (elevated)	298	0	,0
	Porcentagem global				77,1

a. A constante está incluída no modelo.

b. O valor de recorte é ,500

Variáveis na equação

		B	S.E.	Wald	df	Sig.	Exp(B)
Passo 0	Constante	-1,215	,066	339,035	1	,000	,297

Variáveis não presentes na equação

			Escore	df	Sig.
Passo 0	Variáveis	Sex	7,019	1	,008

	Any comorbidity (regardless COVID risk)	4,208	1	,040
	BRCS Score	68,242	1	,000
	Na sua opinião, qual o seu grau de autonomia para decidir condutas no trabalho? (EAV 1-10)	23,561	1	,000
	Na sua opinião, qual o grau de adequação da organização pedagógica de seu programa de residência profissional? (EAV 1-10)	74,615	1	,000
	Perceived adequacy of the availability of personal protective equipment, when providing care for patients in the residency program	28,860	1	,000
	The institution is a university hospital	1,104	1	,293
	Cumulative weekly workload < or >= 90h (dichotomous)	7,316	1	,007
Estatísticas globais		146,391	8	,000

```
Bloco 1: Método = Enter
```

Testes de Omnibus do Modelo de Coeficientes

		Qui-quadrado	df	Sig.
Passo 1	Passo	150,901	8	,000
	Bloco	150,901	8	,000
	Modelo	150,901	8	,000

Resumo do modelo

Passo	Verossimilhança de log -2	R quadrado Cox & Snell	R quadrado Nagelkerke
1	1249,837[a]	,109	,166

a. Estimação finalizada no número de iteração 5 porque as estimativas de parâmetro mudaram foram alteradas para menos de ,001.

Teste de Hosmer e Lemeshow

Passo	Qui-quadrado	df	Sig.
1	4,335	8	,826

Tabela de contingência para teste de Hosmer e Lemeshow

		DASS21 Classification - Stress (dichotomous) = Normal		DASS21 Classification - Stress (dichotomous) = Abnormal (elevated)		
		Observado	Esperado	Observado	Esperado	Total
Passo 1	1	123	122,798	7	7,202	130
	2	122	118,062	8	11,938	130
	3	116	114,476	14	15,524	130
	4	108	110,917	22	19,083	130
	5	105	106,757	25	23,243	130
	6	99	102,512	31	27,488	130
	7	98	97,387	32	32,613	130
	8	90	90,292	40	39,708	130
	9	87	81,273	43	48,727	130
	10	56	59,525	76	72,475	132

Tabela de Classificação[a]

			Previsto		
			DASS21 Classification - Stress (dichotomous)		
	Observado		Normal	Abnormal (elevated)	Porcentagem correta
Passo 1	DASS21 Classification - Stress (dichotomous)	Normal	968	36	96,4
		Abnormal (elevated)	251	47	15,8
	Porcentagem global				78,0

a. O valor de recorte é ,500

Variáveis na equação

		B	S.E.	Wald	df	Sig.	Exp(B)	95% C.I. para EXP(B) Inferior	95% C.I. para EXP(B) Superior
Passo 1[a]	Sex	,606	,187	10,469	1	,001	1,832	1,270	2,644
	Any comorbidity (regardless COVID risk)	,245	,177	1,916	1	,166	1,277	,903	1,806
	BRCS Score	-,124	,020	37,137	1	,000	,883	,849	,919
	Na sua opinião, qual o seu grau de autonomia para decidir condutas no trabalho? (EAV 1-10)	-,030	,034	,776	1	,378	,970	,907	1,038
	Na sua opinião, qual o grau de adequação da organização pedagógica de seu programa de residência profissional? (EAV 1-10)	-,178	,030	35,007	1	,000	,837	,789	,888

	Perceived adequacy of the availability of personal protective equipment, when providing care for patients in the residency program	-,547	,160	11,716	1	,001	,578	,423	,791
	The institution is a university hospital	-,268	,223	1,438	1	,230	,765	,494	1,185
	Cumulative weekly workload < or >= 90h (dichotomous)	,539	,258	4,362	1	,037	1,714	1,034	2,841
	Constante	1,484	,400	13,761	1	,000	4,412		

a. Variável(is) inserida(s) no passo 1: Sex, Any comorbidity (regardless COVID risk), BRCS Score, Na sua opinião, qual o seu grau de autonomia para decidir condutas no trabalho? (EAV 1-10), Na sua opinião, qual o grau de adequação da organização pedagógica de seu programa de residência profissional? (EAV 1-10), Perceived adequacy of the availability of personal protective equipment, when providing care for patients in the residency program, The institution is a university hospital, Cumulative weekly workload < or >= 90h (dichotomous).

Lista entre casos[a]

a. O plot entre casos não é produzido porque nenhum valor discrepante foi encontrado.

```
LOGISTIC REGRESSION VARIABLES OLBI_Classif_2c
  /METHOD=ENTER ComorbAny BRCS_Score Autonomy PedagStruct PPEAvail_Classif Wkload_9090
  /SAVE=COOK ZRESID
  /CASEWISE OUTLIER(3)
  /PRINT=GOODFIT CI(95)
  /CRITERIA=PIN(0.05) POUT(0.10) ITERATE(20) CUT(0.5).
```

Regressão logística

Observações

Saída criada		20-SEP-2020 18:56:22
Comentários		
Entrada	Dados	C:\Users\User\Documents\Pesquisa\Fellow\FellowGenData_V1.sav
	Conjunto de dados ativo	ConjuntodeDados1
	Filtro	<none>
	Ponderação	<none>
	Arquivo Dividido	<none>
	N de linhas em arquivo de dados de trabalho	1313

Tratamento de valor omisso	Definição de omisso	Os valores omissos definidos pelo usuário são tratados como omissos
Sintaxe		LOGISTIC REGRESSION VARIABLES OLBI_Classif_2c<br>/METHOD=ENTER ComorbAny BRCS_Score Autonomy PedagStruct PPEAvail_Classif Wkload_9090<br>/SAVE=COOK ZRESID<br>/CASEWISE OUTLIER(3)<br>/PRINT=GOODFIT CI(95)<br>/CRITERIA=PIN(0.05) POUT(0.10) ITERATE(20) CUT(0.5).
Recursos	Tempo do processador	00:00:00,03
	Tempo decorrido	00:00:00,03
Variáveis Criadas ou Modificadas	COO_12	Análogo às estatísticas de influência de Cook
	ZRE_12	Resíduo normalizado

Resumo de processamento do caso

Casos não ponderados[a]		N	Porcentagem
Casos selecionados	Incluído na análise	1305	99,4
	Casos omissos	8	,6
	Total	1313	100,0
Casos não selecionados		0	,0
Total		1313	100,0

a. Se a ponderação estiver em vigor, veja a tabela de classificação para o número total de casos.

Codificação de variável dependente

Valor original	Valor interno
Low to Moderate	0
High	1

Bloco 0: Bloco Inicial

Tabela de Classificação[a,b]

Observado	Previsto

			OLBI Score Classification		Porcentagem
			Low to Moderate	High	correta
Passo 0	OLBI Score Classification	Low to Moderate	869	0	100,0
		High	436	0	,0
	Porcentagem global				66,6

a. A constante está incluída no modelo.

b. O valor de recorte é ,500

Variáveis na equação

		B	S.E.	Wald	df	Sig.	Exp(B)
Passo 0	Constante	-,690	,059	138,108	1	,000	,502

Variáveis não presentes na equação

			Escore	df	Sig.
Passo 0	Variáveis	Any comorbidity (regardless COVID risk)	16,837	1	,000
		BRCS Score	142,676	1	,000
		Na sua opinião, qual o seu grau de autonomia para decidir condutas no trabalho? (EAV 1-10)	95,392	1	,000
		Na sua opinião, qual o grau de adequação da organização pedagógica de seu programa de residência profissional? (EAV 1-10)	165,405	1	,000
		Perceived adequacy of the availability of personal protective equipment, when providing care for patients in the residency program	44,855	1	,000
		Cumulative weekly workload < or >= 90h (dichotomous)	10,411	1	,001
	Estatísticas globais		297,959	6	,000

```
Bloco 1: Método = Enter
```

Testes de Omnibus do Modelo de Coeficientes

		Qui-quadrado	df	Sig.
Passo 1	Passo	325,955	6	,000
	Bloco	325,955	6	,000

	Modelo	325,955	6	,000

Resumo do modelo

Passo	Verossimilhança de log -2	R quadrado Cox & Snell	R quadrado Nagelkerke
1	1336,730[a]	,221	,307

a. Estimação finalizada no número de iteração 5 porque as estimativas de parâmetro mudaram foram alteradas para menos de ,001.

Teste de Hosmer e Lemeshow

Passo	Qui-quadrado	df	Sig.
1	4,500	8	,809

Tabela de contingência para teste de Hosmer e Lemeshow

		OLBI Score Classification = Low to Moderate		OLBI Score Classification = High		
		Observado	Esperado	Observado	Esperado	Total
Passo 1	1	125	123,740	6	7,260	131
	2	120	117,571	11	13,429	131
	3	110	111,885	21	19,115	131
	4	108	106,026	23	24,974	131
	5	97	98,924	34	32,076	131
	6	83	89,702	48	41,298	131
	7	79	79,921	52	51,079	131
	8	74	66,956	58	65,044	132
	9	48	49,658	83	81,342	131
	10	25	24,618	100	100,382	125

Tabela de Classificação[a]

			Previsto		
			OLBI Score Classification		Porcentagem correta
	Observado		Low to Moderate	High	
Passo 1	OLBI Score Classification	Low to Moderate	758	111	87,2
		High	230	206	47,2
	Porcentagem global				73,9

a. O valor de recorte é ,500

Variáveis na equação

	B	S.E.	Wald	df	Sig.	Exp(B)	95% C.I. para EXP(B)	
							Inferior	Superior

Passo 1[a]	Any comorbidity (regardless COVID risk)	,518	,170	9,283	1	,002	1,678	1,203	2,341
	BRCS Score	-,171	,020	74,409	1	,000	,843	,811	,876
	Na sua opinião, qual o seu grau de autonomia para decidir condutas no trabalho? (EAV 1-10)	-,137	,033	16,925	1	,000	,872	,817	,931
	Na sua opinião, qual o grau de adequação da organização pedagógica de seu programa de residência profissional? (EAV 1-10)	-,252	,029	74,328	1	,000	,777	,734	,823
	Perceived adequacy of the availability of personal protective equipment, when providing care for patients in the residency program	-,633	,158	16,101	1	,000	,531	,390	,723
	Cumulative weekly workload < or >= 90h (dichotomous)	,612	,259	5,570	1	,018	1,844	1,109	3,066
	Constante	3,949	,338	136,836	1	,000	51,898		

a. Variável(is) inserida(s) no passo 1: Any comorbidity (regardless COVID risk), BRCS Score, Na sua opinião, qual o seu grau de autonomia para decidir condutas no trabalho? (EAV 1-10), Na sua opinião, qual o grau de adequação da organização pedagógica de seu programa de residência profissional? (EAV 1-10), Perceived adequacy of the availability of personal protective equipment, when providing care for patients in the residency program, Cumulative weekly workload < or >= 90h (dichotomous).

Lista entre casos[a]

a. O plot entre casos não é produzido porque nenhum valor discrepante foi encontrado.

```
*Nonparametric Tests: Independent Samples.
NPTESTS
  /INDEPENDENT TEST (Autonomy PedagStruct) GROUP (UnivHosp) MANN_WHITNEY
  /MISSING SCOPE=ANALYSIS USERMISSING=EXCLUDE
  /CRITERIA ALPHA=0.05   CILEVEL=95.
```

Testes não paramétricos

Observações

Saída criada	20-SEP-2020 23:19:30
Comentários	

Entrada	Dados	C:\Users\User\Documents\Pesquisa\Fellow\FellowGenData_V1.sav
	Conjunto de dados ativo	ConjuntodeDados1
	Filtro	<none>
	Ponderação	<none>
	Arquivo Dividido	<none>
	N de linhas em arquivo de dados de trabalho	1313
Sintaxe		NPTESTS<br>/INDEPENDENT TEST (Autonomy PedagStruct) GROUP (UnivHosp) MANN_WHITNEY<br>/MISSING SCOPE=ANALYSIS USERMISSING=EXCLUDE<br>/CRITERIA ALPHA=0.05 CILEVEL=95.
Recursos	Tempo do processador	00:00:00,33
	Tempo decorrido	00:00:00,40

null : null

Resumo de Teste de Hipótese

	Hipótese nula	Teste	Sig.	Decisão
1	A distribuição de Na sua opinião, qual o seu grau de autonomia para decidir condutas no trabalho? (EAV 1-10) é a mesma entre as categorias de The institution is a university hospital.	Teste U de Mann-Whitney de amostras independentes	,845	Reter a hipótese nula.
2	A distribuição de Na sua opinião, qual o grau de adequação da organização pedagógica de seu programa de residência profissional? (EAV 1-10) é a mesma entre as categorias de The institution is a university hospital.	Teste U de Mann-Whitney de amostras independentes	,350	Reter a hipótese nula.

São exibidas significâncias assintóticas. O nível de significância é ,05.

Teste U de Mann-Whitney de amostras independentes

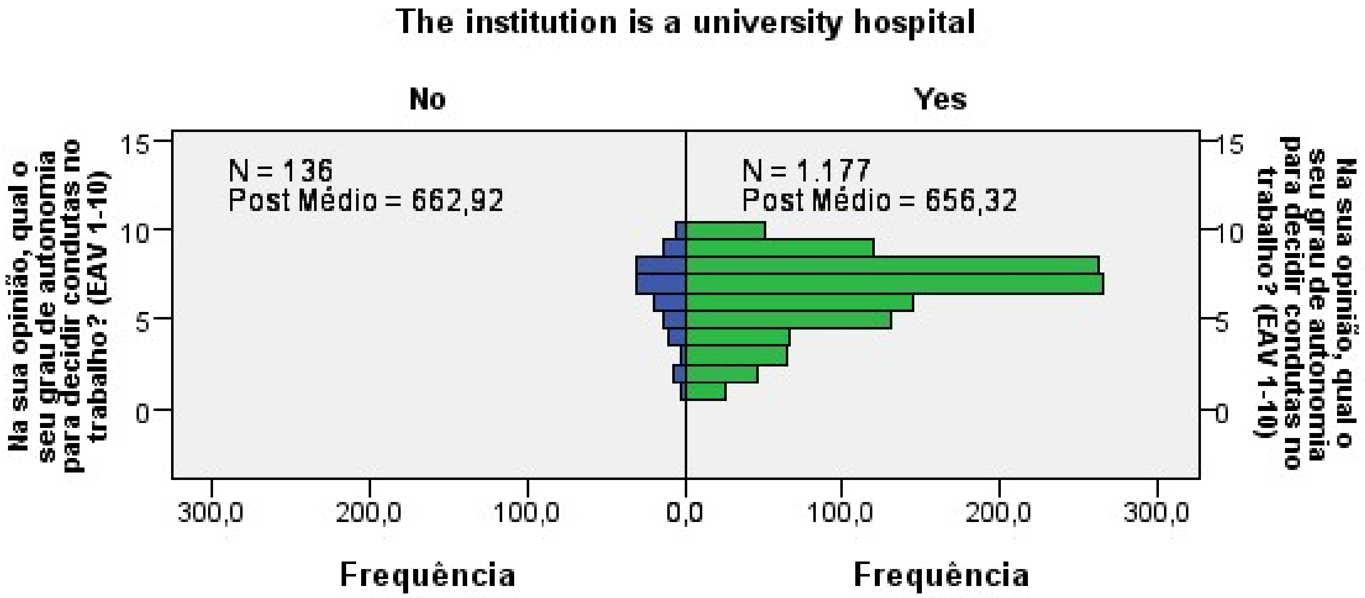

**N total**	1.313
**U de Mann-Whitney**	79.230,500
**Wilcoxon W**	772.483,500
**Estatística de teste**	79.230,500
**Erro padrão**	4.128,587
**Estatística de Teste Padronizado**	-,195
**Sig. assintótico (teste de 2 lados)**	,845

Teste U de Mann-Whitney de amostras independentes

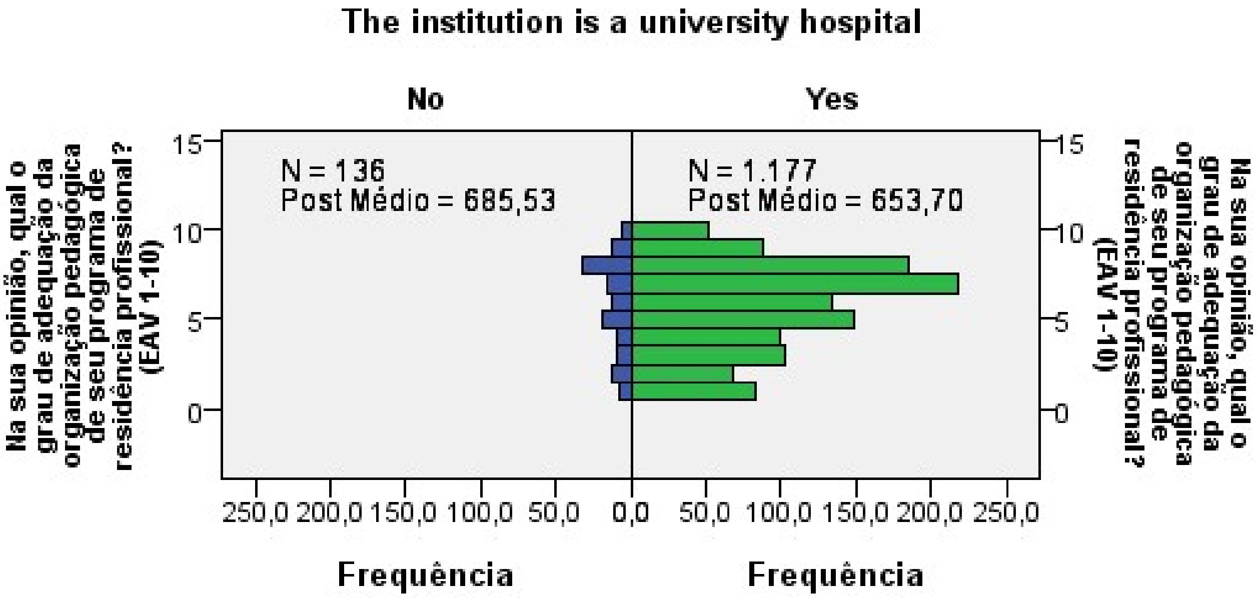

N total	1.313
U de Mann-Whitney	76.156,500
Wilcoxon W	769.409,500
Estatística de teste	76.156,500
Erro padrão	4.153,230
Estatística de Teste Padronizado	-,934
Sig. assintótico (teste de 2 lados)	,350

```
T-TEST GROUPS=RsPrgType(0 1)
  /MISSING=ANALYSIS
  /VARIABLES=OLBI_D OLBI_E
  /CRITERIA=CI(.95).
```

Teste-T

Observações

Saída criada		20-SEP-2020 23:30:21
Comentários		
Entrada	Dados	C:\Users\User\Documents\Pesquisa\Fellow\FellowGenData_V1.sav
	Conjunto de dados ativo	ConjuntodeDados1
	Filtro	<none>
	Ponderação	<none>
	Arquivo Dividido	<none>
	N de linhas em arquivo de dados de trabalho	1313
Tratamento de valor omisso	Definição de omisso	Os valores omissos definidos pelo usuário são tratados como omissos.
	Casos utilizados	As estatísticas para cada análise são baseadas nos casos sem dados omissos ou fora do intervalo para qualquer variável da análise.
Sintaxe		T-TEST GROUPS=RsPrgType(0 1) /MISSING=ANALYSIS /VARIABLES=OLBI_D OLBI_E /CRITERIA=CI(.95).
Recursos	Tempo do processador	00:00:00,02
	Tempo decorrido	00:00:00,02

Estatísticas de grupo

	Type of residency program	N	Média	Erro Desvio	Erro padrão da média
OLBI Disengagement Score	Non-Medical (Other HCP)	639	2,7365	,81289	,03216
	Medical	674	2,8095	,86305	,03324
OLBI Exhaustion Score	Non-Medical (Other HCP)	639	3,5808	,69267	,02740
	Medical	674	3,5298	,77501	,02985

Teste de amostras independentes

		Teste de Levene para igualdade de variâncias		teste-t para Igualdade de Médias						
									95% Intervalo de Confiança da Diferença	
		Z	Sig.	t	df	Sig. (2 extremidades)	Diferença média	Erro padrão de diferença	Inferior	Superior
OLBI Disengagement Score	Variâncias iguais assumidas	3,509	,061	-1,576	1311	,115	-,07303	,04633	-,16391	,01785
	Variâncias iguais não assumidas			-1,579	1310,945	,115	-,07303	,04625	-,16377	,01771

OLBI Exhaustion Score	Variâncias iguais assumidas	7,257	,007	1,256	1311	,209	,05105	,04064	-,02868	,13078
	Variâncias iguais não assumidas			1,260	1306,487	,208	,05105	,04052	-,02844	,13055

```
DATASET ACTIVATE ConjuntodeDados1.

SAVE OUTFILE='C:\Users\User\Documents\Pesquisa\Fellow\FellowGenData_V1.sav'
  /COMPRESSED.
CORRELATIONS
  /VARIABLES=OLBI_D OLBI_E Sex ComorbAny UnivHosp BRCS_Score Autonomy PedagStruct Wkload_5P PPEAvail
  /PRINT=TWOTAIL NOSIG
  /MISSING=PAIRWISE.
```

Correlações

Observações

Saída criada		20-SEP-2020 23:36:37
Comentários		
Entrada	Dados	C:\Users\User\Documents\Pesquisa\Fellow\FellowGenData_V1.sav
	Conjunto de dados ativo	ConjuntodeDados1
	Filtro	<none>
	Ponderação	<none>
	Arquivo Dividido	<none>
	N de linhas em arquivo de dados de trabalho	1313
Tratamento de valor omisso	Definição de omisso	Os valores omissos definidos pelo usuário são tratados como omissos.
	Casos utilizados	As estatísticas para cada par de variáveis são baseadas em todos os casos com dados válidos para aquele par.
Sintaxe		CORRELATIONS /VARIABLES=OLBI_D OLBI_E Sex ComorbAny UnivHosp BRCS_Score Autonomy PedagStruct Wkload_5P PPEAvail /PRINT=TWOTAIL NOSIG /MISSING=PAIRWISE.
Recursos	Tempo do processador	00:00:00,02
	Tempo decorrido	00:00:00,02

Correlações

		OLBI Disengagement Score	OLBI Exhaustion Score	Sex	Any comorbidity (regardless COVID risk)	The institution is a university hospital	BRCS Score	Na sua opinião, qual o seu grau de autonomia para decidir condutas no trabalho? (EAV 1-10)	Na sua opinião, qual o grau de adequação da organização pedagógica de seu programa de residência profissional? (EAV 1-10)	Cumulative weekly workload (five categories)	Na sua prática profissional, especialmente no atendimento a pacientes, por quanto tempo você tem acesso a equipamentos de proteção individual (EPI) suficientes e adequados? (Lickert 1-5)
OLBI Disengagement Score	Correlação de Pearson	1	,576**	-,045	,110**	-,026	-,406**	-,329**	-,439**	,053	-,224**
	Sig. (2 extremidades)		,000	,105	,000	,353	,000	,000	,000	,053	,000
	N	1313	1313	1310	1305	1313	1313	1313	1313	1313	1313
OLBI Exhaustion Score	Correlação de Pearson	,576**	1	,146**	,109**	-,014	-,366**	-,262**	-,347**	,122**	-,193**
	Sig. (2 extremidades)	,000		,000	,000	,622	,000	,000	,000	,000	,000
	N	1313	1313	1310	1305	1313	1313	1313	1313	1313	1313
Sex	Correlação de Pearson	-,045	,146**	1	-,033	,003	-,011	,068*	,012	-,102**	,039
	Sig. (2 extremidades)	,105	,000		,236	,928	,678	,014	,664	,000	,154
	N	1310	1310	1310	1302	1310	1310	1310	1310	1310	1310
Any comorbidity (regardless COVID risk)	Correlação de Pearson	,110**	,109**	-,033	1	-,063*	-,036	-,070*	-,052	,046	-,052
	Sig. (2 extremidades)	,000	,000	,236		,023	,200	,011	,062	,097	,061
	N	1305	1305	1302	1305	1305	1305	1305	1305	1305	1305
The institution is a university hospital	Correlação de Pearson	-,026	-,014	,003	-,063*	1	-,001	-,010	-,020	-,051	,036
	Sig. (2 extremidades)	,353	,622	,928	,023		,966	,724	,469	,067	,188
	N	1313	1313	1310	1305	1313	1313	1313	1313	1313	1313
BRCS Score	Correlação de Pearson	-,406**	-,366**	-,011	-,036	-,001	1	,244**	,227**	,023	,120**
	Sig. (2 extremidades)	,000	,000	,678	,200	,966		,000	,000	,401	,000
	N	1313	1313	1310	1305	1313	1313	1313	1313	1313	1313
Na sua opinião, qual o seu grau de autonomia para decidir condutas no trabalho? (EAV 1-10)	Correlação de Pearson	-,329**	-,262**	,068*	-,070*	-,010	,244**	1	,320**	-,021	,132**
	Sig. (2 extremidades)	,000	,000	,014	,011	,724	,000		,000	,449	,000
	N	1313	1313	1310	1305	1313	1313	1313	1313	1313	1313
Na sua opinião, qual o grau de adequação da organização pedagógica de seu programa de residência profissional? (EAV 1-10)	Correlação de Pearson	-,439**	-,347**	,012	-,052	-,020	,227**	,320**	1	-,004	,170**
	Sig. (2 extremidades)	,000	,000	,664	,062	,469	,000	,000		,882	,000
	N	1313	1313	1310	1305	1313	1313	1313	1313	1313	1313
Cumulative weekly workload (five categories)	Correlação de Pearson	,053	,122**	-,102**	,046	-,051	,023	-,021	-,004	1	-,110**
	Sig. (2 extremidades)	,053	,000	,000	,097	,067	,401	,449	,882		,000
	N	1313	1313	1310	1305	1313	1313	1313	1313	1313	1313
Na sua prática profissional, especialmente no atendimento a pacientes, por quanto tempo você tem acesso a equipamentos de proteção individual (EPI) suficientes e adequados? (Lickert 1-5)	Correlação de Pearson	-,224**	-,193**	,039	-,052	,036	,120**	,132**	,170**	-,110**	1
	Sig. (2 extremidades)	,000	,000	,154	,061	,188	,000	,000	,000	,000	
	N	1313	1313	1310	1305	1313	1313	1313	1313	1313	1313

**. A correlação é significativa no nível 0,01 (2 extremidades).

*. A correlação é significativa no nível 0,05 (2 extremidades).

```
NONPAR CORR
  /VARIABLES=OLBI_D OLBI_E Sex ComorbAny UnivHosp BRCS_Score Autonomy PedagStruct Wkload_5P PPEAvail
  /PRINT=SPEARMAN TWOTAIL NOSIG
  /MISSING=PAIRWISE.
```

Correlações não paramétricas

Observações

Saída criada		20-SEP-2020 23:36:37
Comentários		
Entrada	Dados	C:\Users\User\Documents\Pesquisa\Fellow\FellowGenData_V1.sav
	Conjunto de dados ativo	ConjuntodeDados1
	Filtro	<none>
	Ponderação	<none>
	Arquivo Dividido	<none>
	N de linhas em arquivo de dados de trabalho	1313
Tratamento de valor omisso	Definição de omisso	Os valores omissos definidos pelo usuário são tratados como omissos.
	Casos utilizados	As estatísticas para cada par de variáveis são baseadas em todos os casos com dados válidos para aquele par.
Sintaxe		NONPAR CORR /VARIABLES=OLBI_D OLBI_E Sex ComorbAny UnivHosp BRCS_Score Autonomy PedagStruct Wkload_5P PPEAvail /PRINT=SPEARMAN TWOTAIL NOSIG /MISSING=PAIRWISE.
Recursos	Tempo do processador	00:00:00,02
	Tempo decorrido	00:00:00,02
	Número de Casos Permitidos	241979 casos[a]

a. Baseado na disponibilidade de memória da área de trabalho

Correlações

			OLBI Disengagement Score	OLBI Exhaustion Score	Sex	Any comorbidity (regardless COVID risk)	The institution is a university hospital	BRCS Score	Na sua opinião, qual o seu grau de autonomia para decidir condutas no trabalho? (EAV 1-10)	Na sua opinião, qual o grau de adequação da organização pedagógica de seu programa de residência profissional? (EAV 1-10)	Cumulative weekly workload (five categories)	Na sua prática profissional, especialmente no atendimento a pacientes, por quanto tempo você tem acesso a equipamentos de proteção individual (EPI) suficientes e adequados? (Likert 1-5)
rô de Spearman	OLBI Disengagement Score	Coeficiente de Correlação	1,000	,572**	-,039	,104**	-,021	-,394**	-,299**	-,437**	,054*	-,212**
		Sig. (2 extremidades)	.	,000	,154	,000	,438	,000	,000	,000	,050	,000
		N	1313	1313	1310	1305	1313	1313	1313	1313	1313	1313
	OLBI Exhaustion Score	Coeficiente de Correlação	,572**	1,000	,127**	,114**	-,025	-,352**	-,257**	-,345**	,125**	-,180**
		Sig. (2 extremidades)	,000	.	,000	,000	,364	,000	,000	,000	,000	,000
		N	1313	1313	1310	1305	1313	1313	1313	1313	1313	1313
	Sex	Coeficiente de Correlação	-,039	,127**	1,000	-,033	,003	-,012	,060*	,009	-,089**	,041
		Sig. (2 extremidades)	,154	,000	.	,236	,928	,658	,029	,733	,001	,142
		N	1310	1310	1310	1302	1310	1310	1310	1310	1310	1310
	Any comorbidity (regardless COVID risk)	Coeficiente de Correlação	,104**	,114**	-,033	1,000	-,063*	-,038	-,061*	-,051	,038	-,034
		Sig. (2 extremidades)	,000	,000	,236	.	,023	,174	,028	,068	,167	,220
		N	1305	1305	1302	1305	1305	1305	1305	1305	1305	1305
	The institution is a university hospital	Coeficiente de Correlação	-,021	-,025	,003	-,063*	1,000	-,004	-,005	-,026	-,061*	,028
		Sig. (2 extremidades)	,438	,364	,928	,023	.	,876	,845	,350	,027	,306
		N	1313	1313	1310	1305	1313	1313	1313	1313	1313	1313
	BRCS Score	Coeficiente de Correlação	-,394**	-,352**	-,012	-,038	-,004	1,000	,239**	,218**	,027	,112**
		Sig. (2 extremidades)	,000	,000	,658	,174	,876	.	,000	,000	,335	,000
		N	1313	1313	1310	1305	1313	1313	1313	1313	1313	1313
	Na sua opinião, qual o seu grau de autonomia para decidir condutas no trabalho? (EAV 1-10)	Coeficiente de Correlação	-,299**	-,257**	,060*	-,061*	-,005	,239**	1,000	,303**	-,029	,129**
		Sig. (2 extremidades)	,000	,000	,029	,028	,845	,000	.	,000	,292	,000
		N	1313	1313	1310	1305	1313	1313	1313	1313	1313	1313
	Na sua opinião, qual o grau de adequação da organização pedagógica de seu programa de residência profissional? (EAV 1-10)	Coeficiente de Correlação	-,437**	-,345**	,009	-,051	-,026	,218**	,303**	1,000	-,003	,165**
		Sig. (2 extremidades)	,000	,000	,733	,068	,350	,000	,000	.	,915	,000
		N	1313	1313	1310	1305	1313	1313	1313	1313	1313	1313
	Cumulative weekly workload (five categories)	Coeficiente de Correlação	,054*	,125**	-,089**	,038	-,061*	,027	-,029	-,003	1,000	-,111**
		Sig. (2 extremidades)	,050	,000	,001	,167	,027	,335	,292	,915	.	,000
		N	1313	1313	1310	1305	1313	1313	1313	1313	1313	1313
	Na sua prática profissional, especialmente no atendimento a	Coeficiente de Correlação	-,212**	-,180**	,041	-,034	,028	,112**	,129**	,165**	-,111**	1,000
		Sig. (2 extremidades)	,000	,000	,142	,220	,306	,000	,000	,000	,000	.

pacientes, por quanto tempo você tem acesso a equipamentos de proteção individual (EPI) suficientes e adequados? (Lickert 1-5)	N	1313	1313	1310	1305	1313	1313	1313	1313	1313	1313

**. A correlação é significativa no nível 0,01 (2 extremidades).

*. A correlação é significativa no nível 0,05 (2 extremidades).

```
EXAMINE VARIABLES=OLBI_D OLBI_E BY Sex ComorbAny BRCS_Classif Autonomy_Classif PedagStr_Classif
    PPEAvail_Classif Wkload_6060
  /PLOT HISTOGRAM NPPLOT
  /PERCENTILES(5,10,25,50,75,90,95) HAVERAGE
  /STATISTICS DESCRIPTIVES
  /CINTERVAL 95
  /MISSING PAIRWISE
  /NOTOTAL.
```

Explorar

Observações

Saída criada		21-SEP-2020 00:05:12
Comentários		
Entrada	Dados	C:\Users\User\Documents\Pesquisa\Fellow\FellowGenData_V1.sav
	Conjunto de dados ativo	ConjuntodeDados1
	Filtro	<none>
	Ponderação	<none>
	Arquivo Dividido	<none>
	N de linhas em arquivo de dados de trabalho	1313
Tratamento de valor omisso	Definição de omisso	Os valores omissos definidos pelo usuário para variáveis dependentes são tratados como omissos.
	Casos utilizados	As estatísticas são baseadas em casos sem valores omissos para a variável dependente ou fatores que estão sendo analisados.

Sintaxe		EXAMINE VARIABLES=OLBI_D OLBI_E BY Sex ComorbAny BRCS_Classif Autonomy_Classif PedagStr_Classif PPEAvail_Classif Wkload_6060 /PLOT HISTOGRAM NPPLOT /PERCENTILES(5,10,25,50,75,90,95) HAVERAGE /STATISTICS DESCRIPTIVES /CINTERVAL 95 /MISSING PAIRWISE /NOTOTAL.
Recursos	Tempo do processador	00:00:11,86
	Tempo decorrido	00:00:11,57

Sex

Resumo de processamento do caso

		Casos					
		Válido		Omisso		Total	
	Sex	N	Porcentagem	N	Porcentagem	N	Porcentagem
OLBI Disengagement Score	Male	285	100,0%	0	0,0%	285	100,0%
	Female	1025	100,0%	0	0,0%	1025	100,0%
OLBI Exhaustion Score	Male	285	100,0%	0	0,0%	285	100,0%
	Female	1025	100,0%	0	0,0%	1025	100,0%

Descritivos

	Sex			Estatística	Erro Erro
OLBI Disengagement Score	Male	Média		2,8443	,05217
		95% Intervalo de Confiança para Média	Limite inferior	2,7416	
			Limite superior	2,9470	
		5% da média aparada		2,8349	
		Mediana		2,8750	
		Variância		,776	
		Erro Desvio		,88079	
		Mínimo		1,00	
		Máximo		5,00	
		Intervalo		4,00	
		Amplitude interquartil		1,25	
		Assimetria		,166	,144

		Curtose		-,556	,288
	Female	Média		2,7533	,02582
		95% Intervalo de Confiança para Média	Limite inferior	2,7026	
			Limite superior	2,8040	
		5% da média aparada		2,7417	
		Mediana		2,7500	
		Variância		,683	
		Erro Desvio		,82663	
		Mínimo		1,00	
		Máximo		5,00	
		Intervalo		4,00	
		Amplitude interquartil		1,25	
		Assimetria		,184	,076
		Curtose		-,390	,153
OLBI Exhaustion Score	Male	Média		3,3500	,04911
		95% Intervalo de Confiança para Média	Limite inferior	3,2533	
			Limite superior	3,4466	
		5% da média aparada		3,3748	
		Mediana		3,3800	
		Variância		,687	
		Erro Desvio		,82909	
		Mínimo		1,00	
		Máximo		5,00	
		Intervalo		4,00	
		Amplitude interquartil		1,20	
		Assimetria		-,447	,144
		Curtose		-,113	,288
	Female	Média		3,6104	,02181
		95% Intervalo de Confiança para Média	Limite inferior	3,5676	
			Limite superior	3,6532	
		5% da média aparada		3,6306	
		Mediana		3,6300	
		Variância		,488	
		Erro Desvio		,69830	
		Mínimo		1,00	
		Máximo		5,00	
		Intervalo		4,00	
		Amplitude interquartil		,99	
		Assimetria		-,476	,076
		Curtose		,186	,153

Percentis

			Percentis						
		Sex	5	10	25	50	75	90	95

Média Ponderada (Definição 1)	OLBI Disengagement Score	Male	1,5000	1,6250	2,2500	2,8750	3,5000	4,0500	4,3750
		Female	1,3750	1,6250	2,1250	2,7500	3,3750	3,8750	4,2500
	OLBI Exhaustion Score	Male	1,8800	2,2020	2,8050	3,3800	4,0000	4,2900	4,6300
		Female	2,2900	2,6300	3,1400	3,6300	4,1300	4,5000	4,7100
Teste de Tukey	OLBI Disengagement Score	Male			2,2500	2,8750	3,5000		
		Female			2,1250	2,7500	3,3750		
	OLBI Exhaustion Score	Male			2,8600	3,3800	4,0000		
		Female			3,1400	3,6300	4,1300		

Testes de Normalidade

		Kolmogorov-Smirnov[a]			Shapiro-Wilk		
	Sex	Estatística	df	Sig.	Estatística	df	Sig.
OLBI Disengagement Score	Male	,063	285	,009	,988	285	,014
	Female	,054	1025	,000	,991	1025	,000
OLBI Exhaustion Score	Male	,066	285	,004	,980	285	,001
	Female	,065	1025	,000	,982	1025	,000

a. Correlação de Significância de Lilliefors

OLBI Disengagement Score

Histogramas

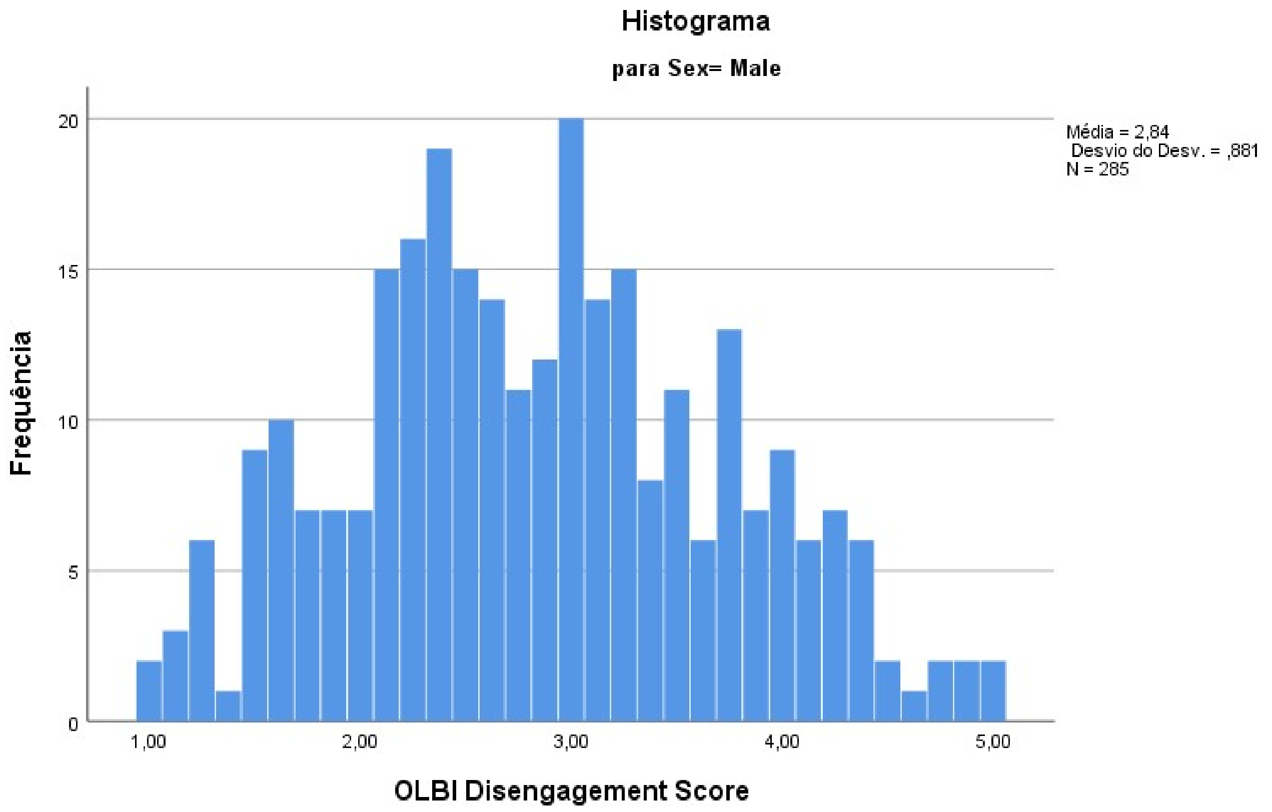

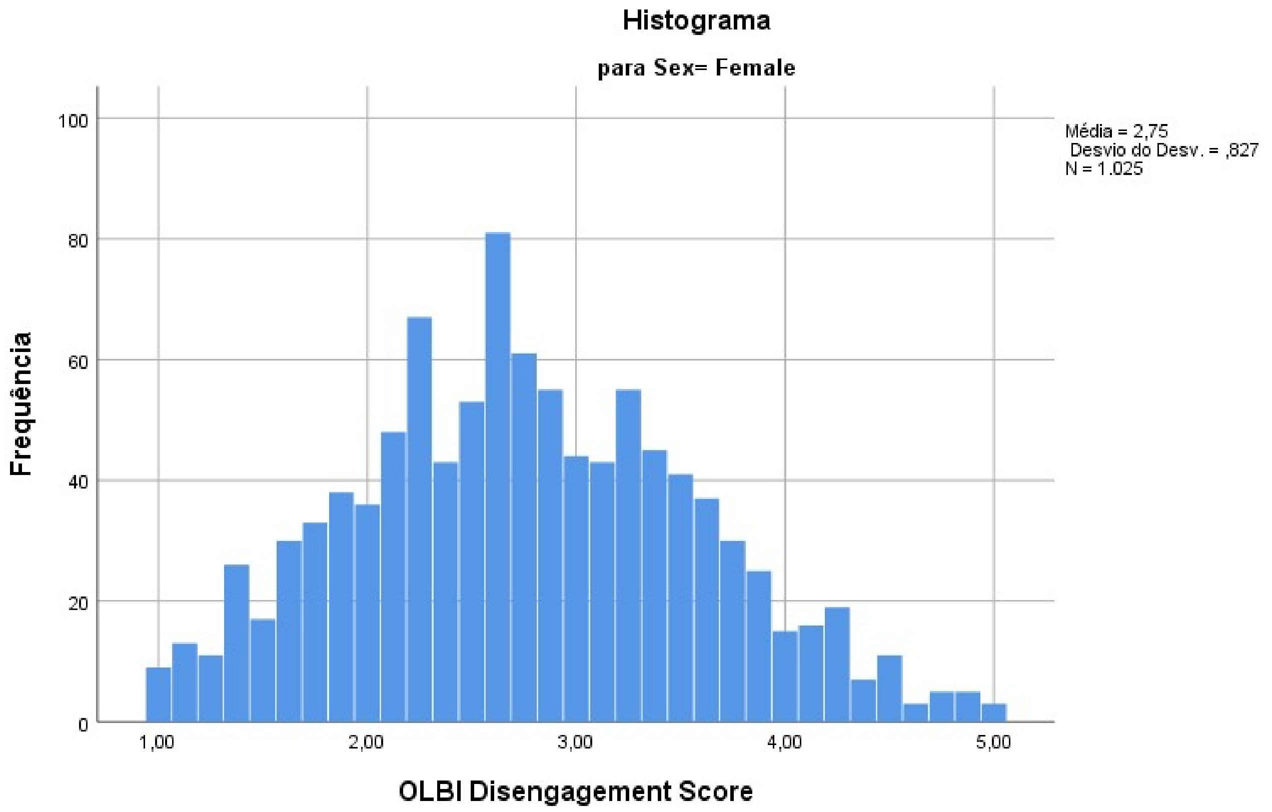

Gráfico Q-Q normais

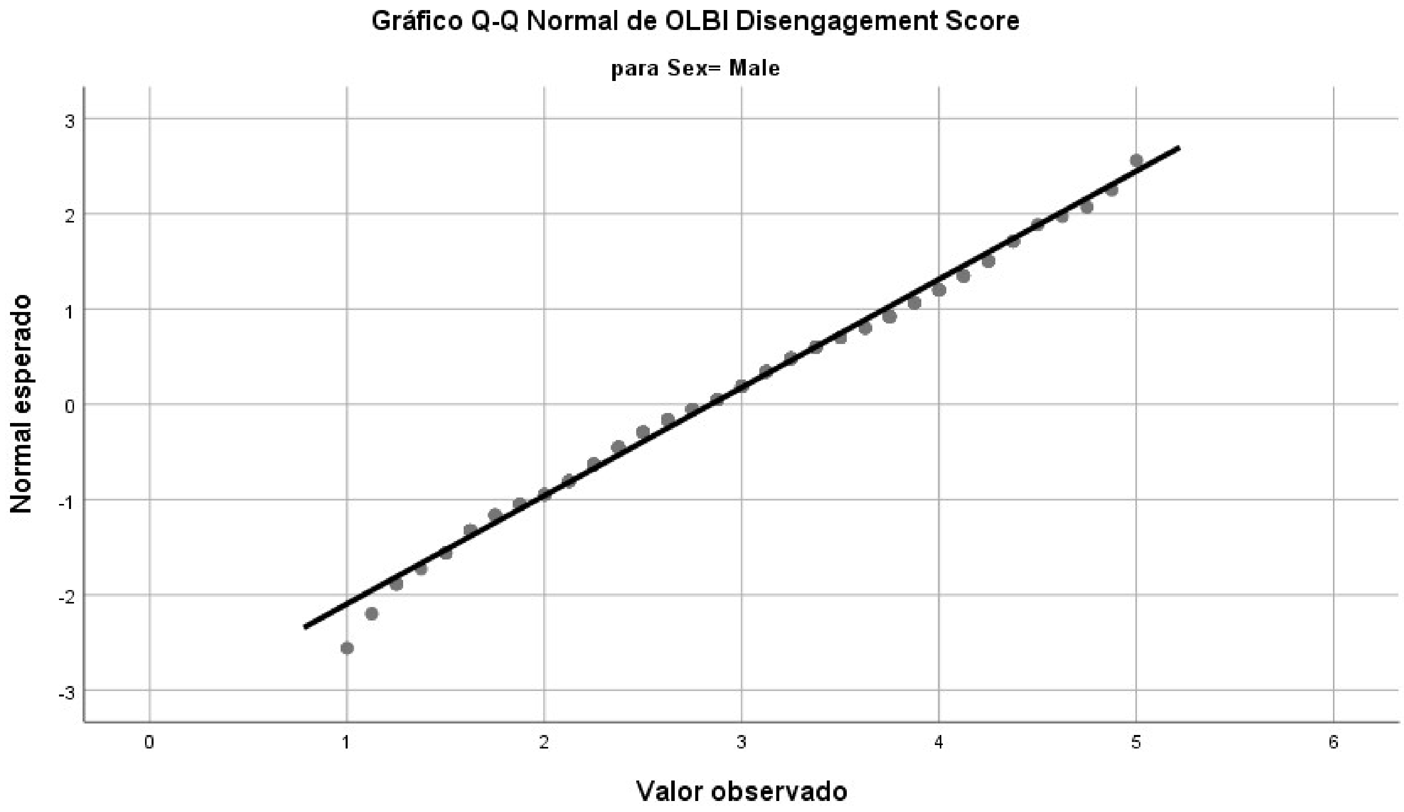

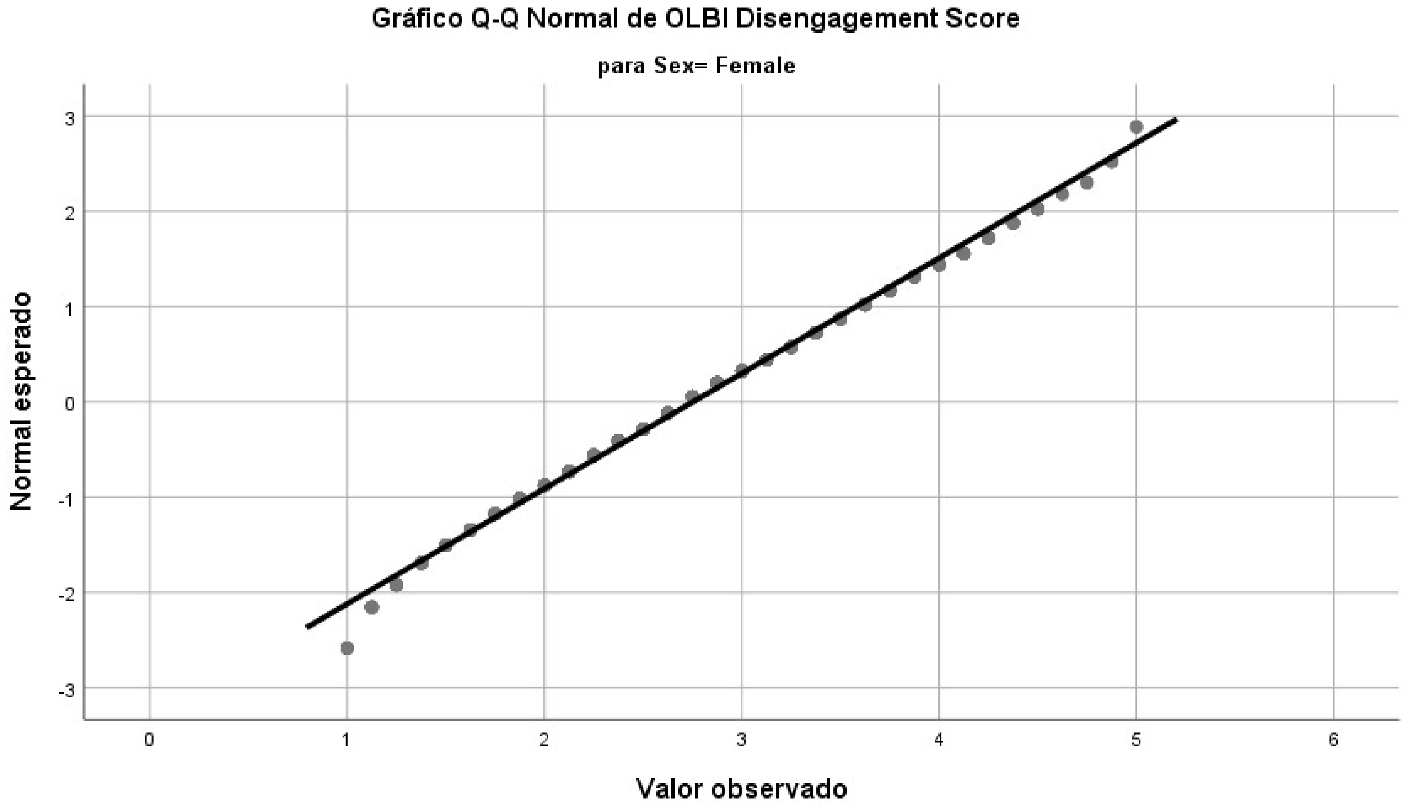

Gráfico Q-Q normais sem tendência

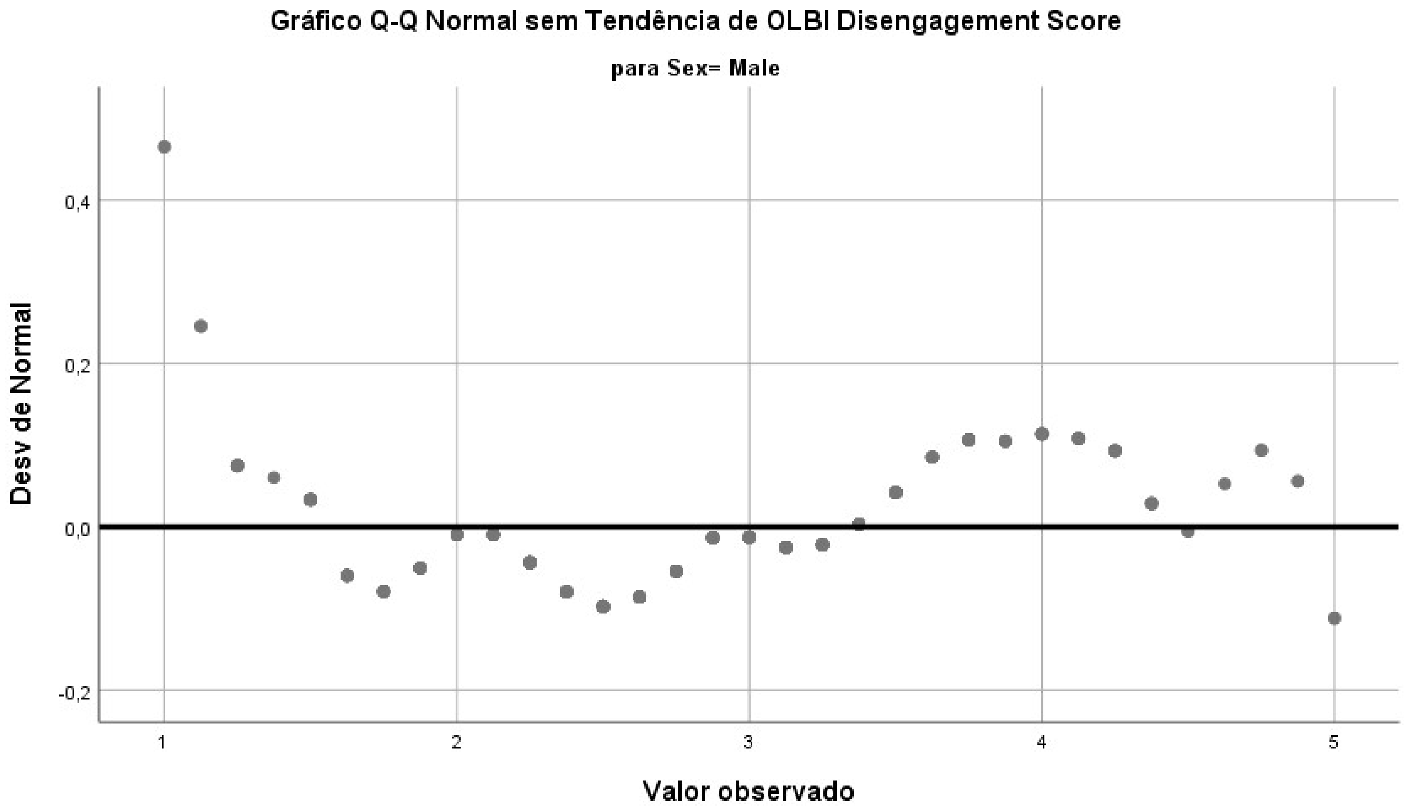

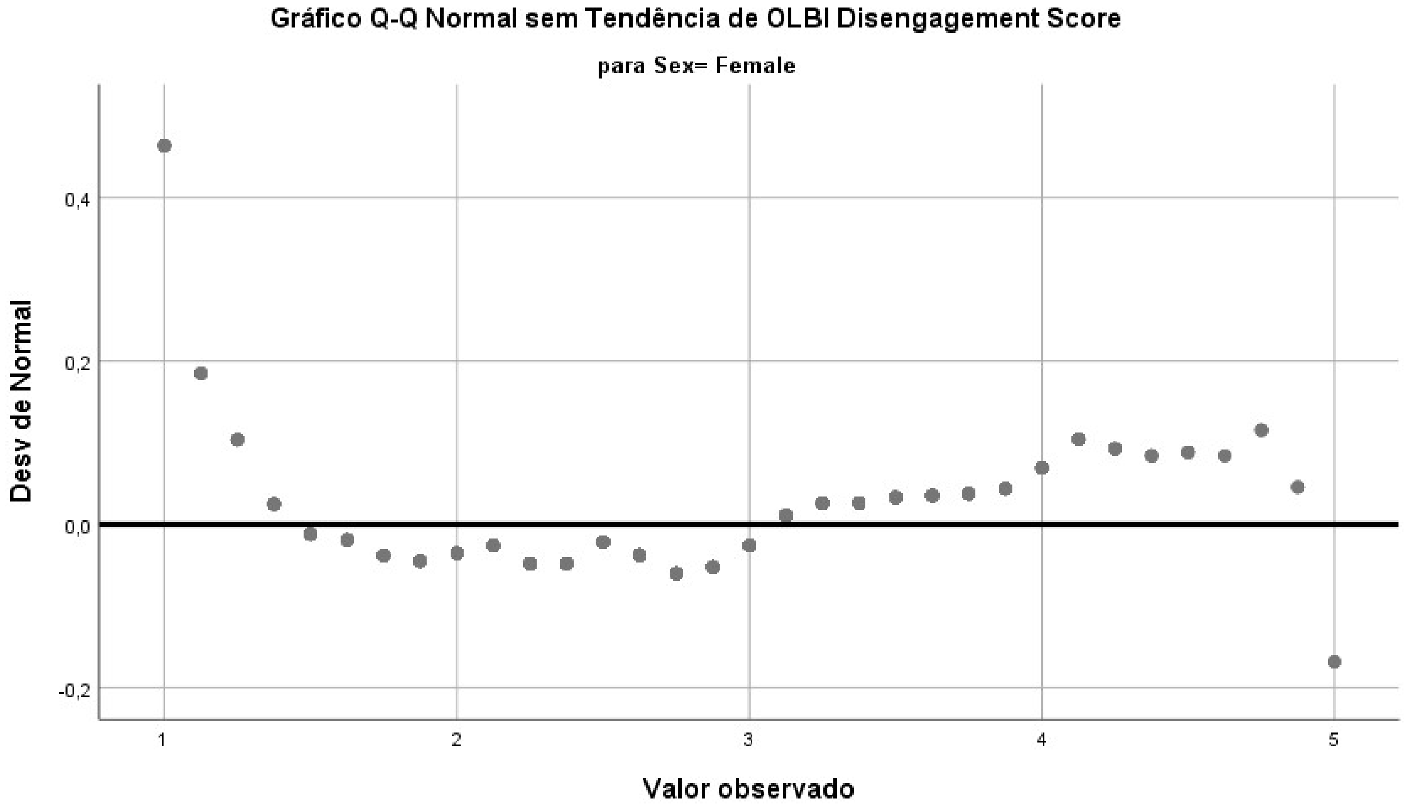

OLBI Exhaustion Score

Histogramas

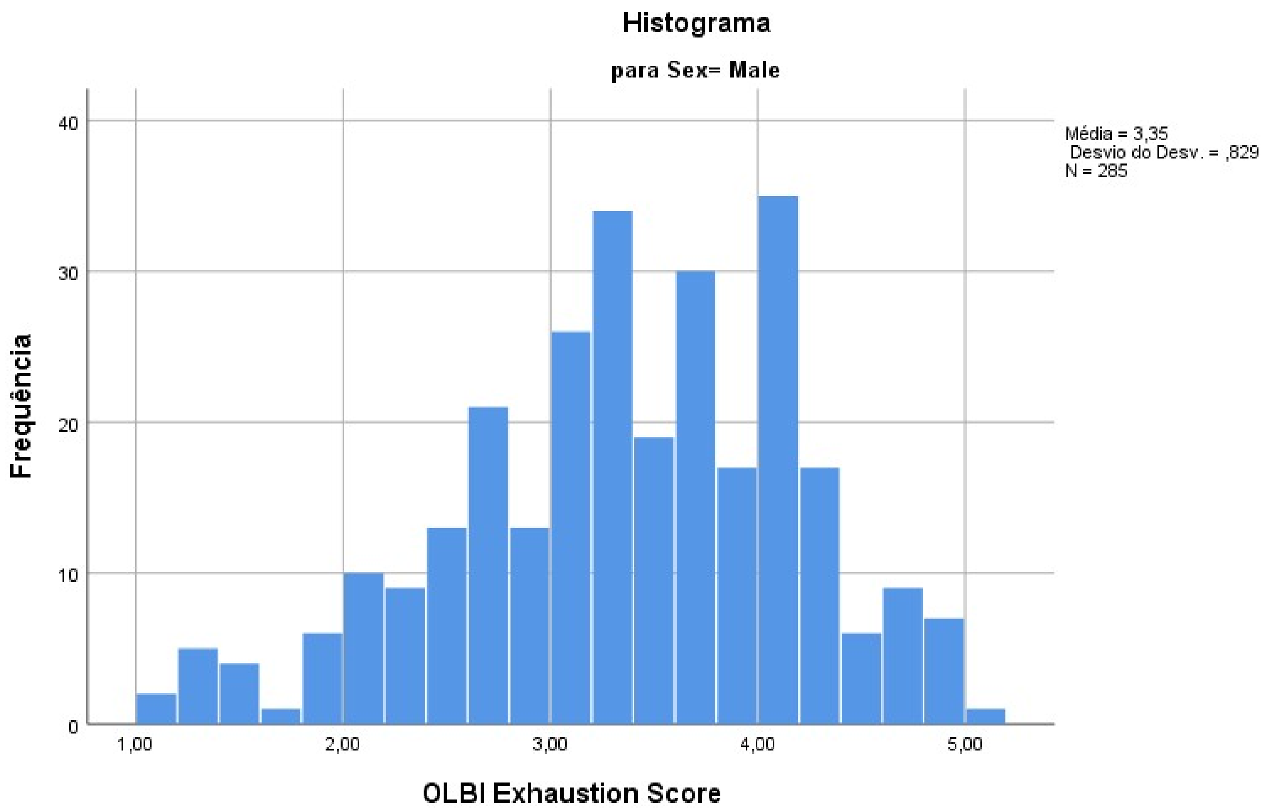

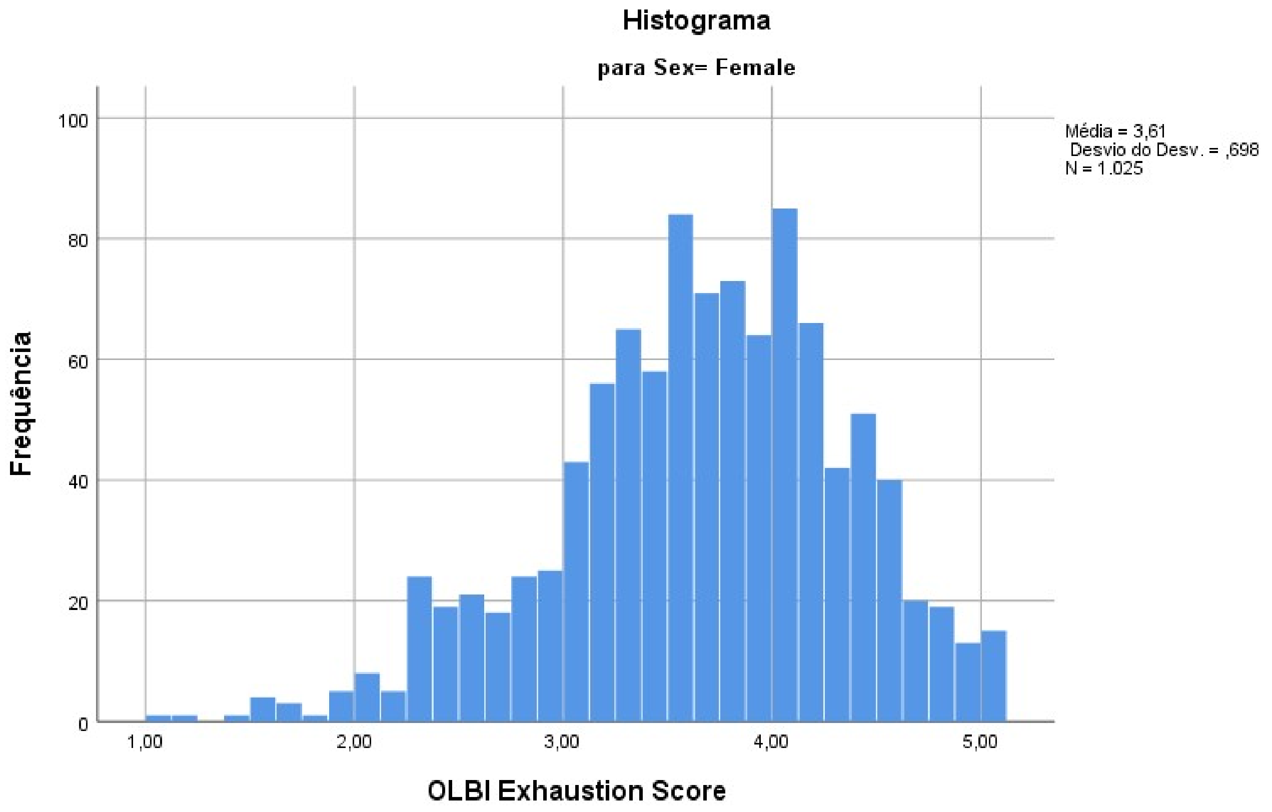

Gráfico Q-Q normais

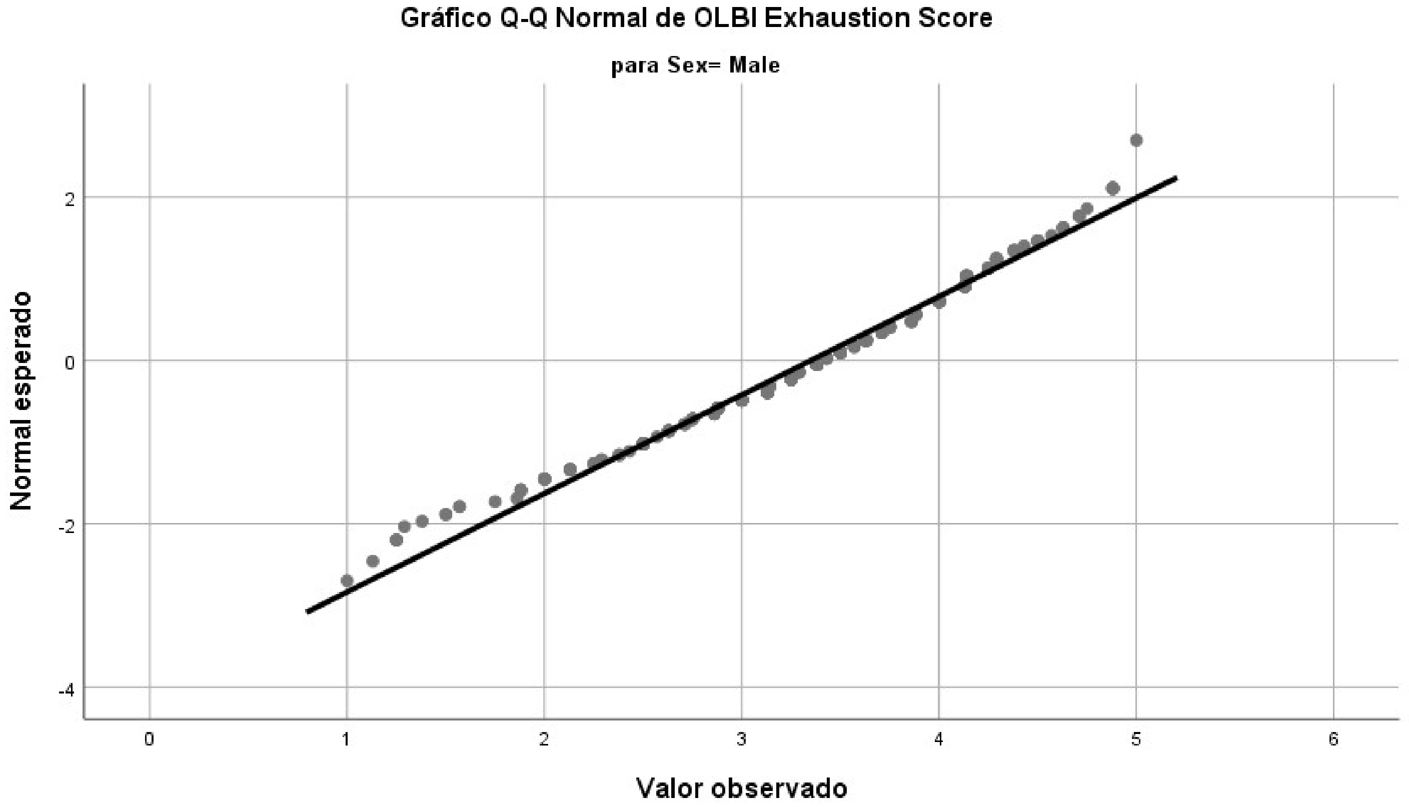

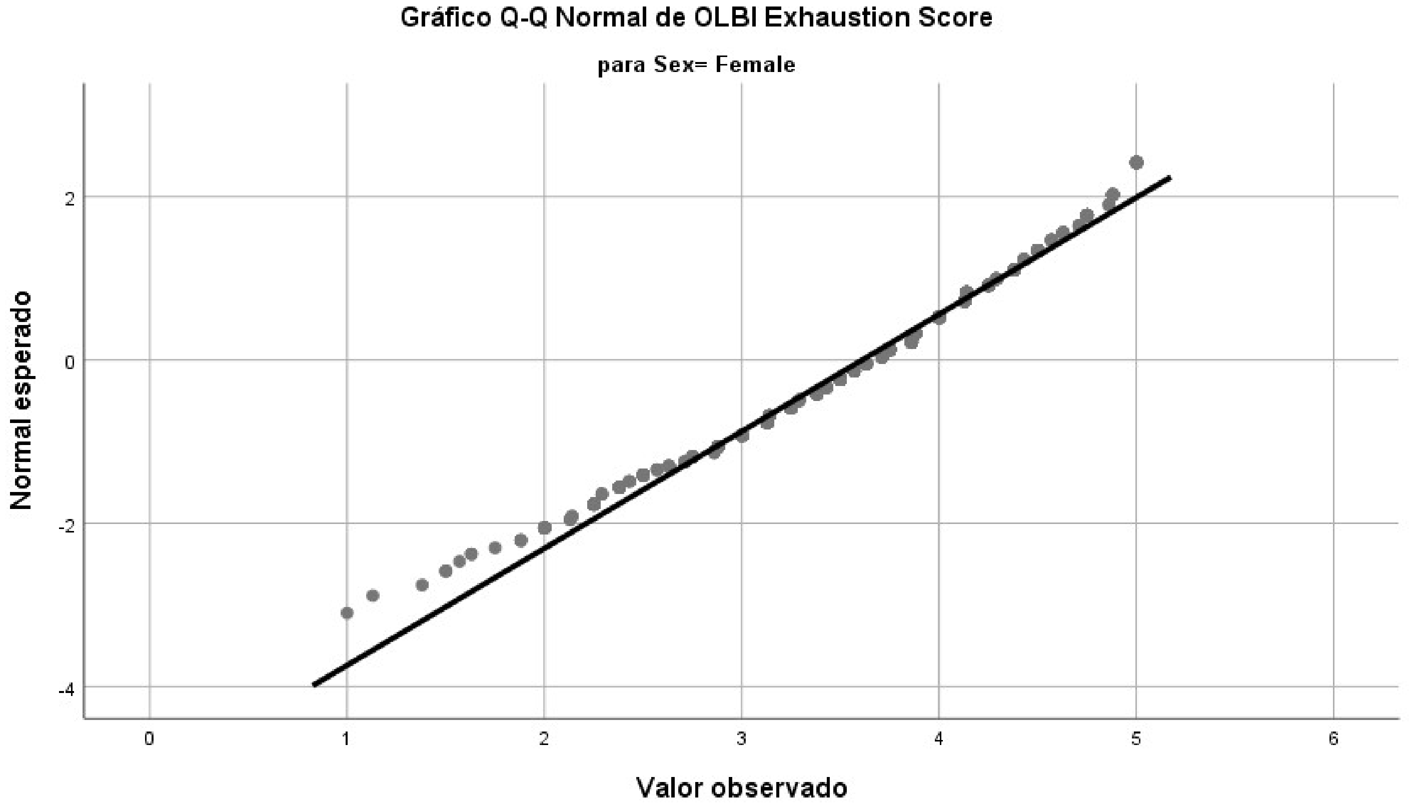

Gráfico Q-Q normais sem tendência

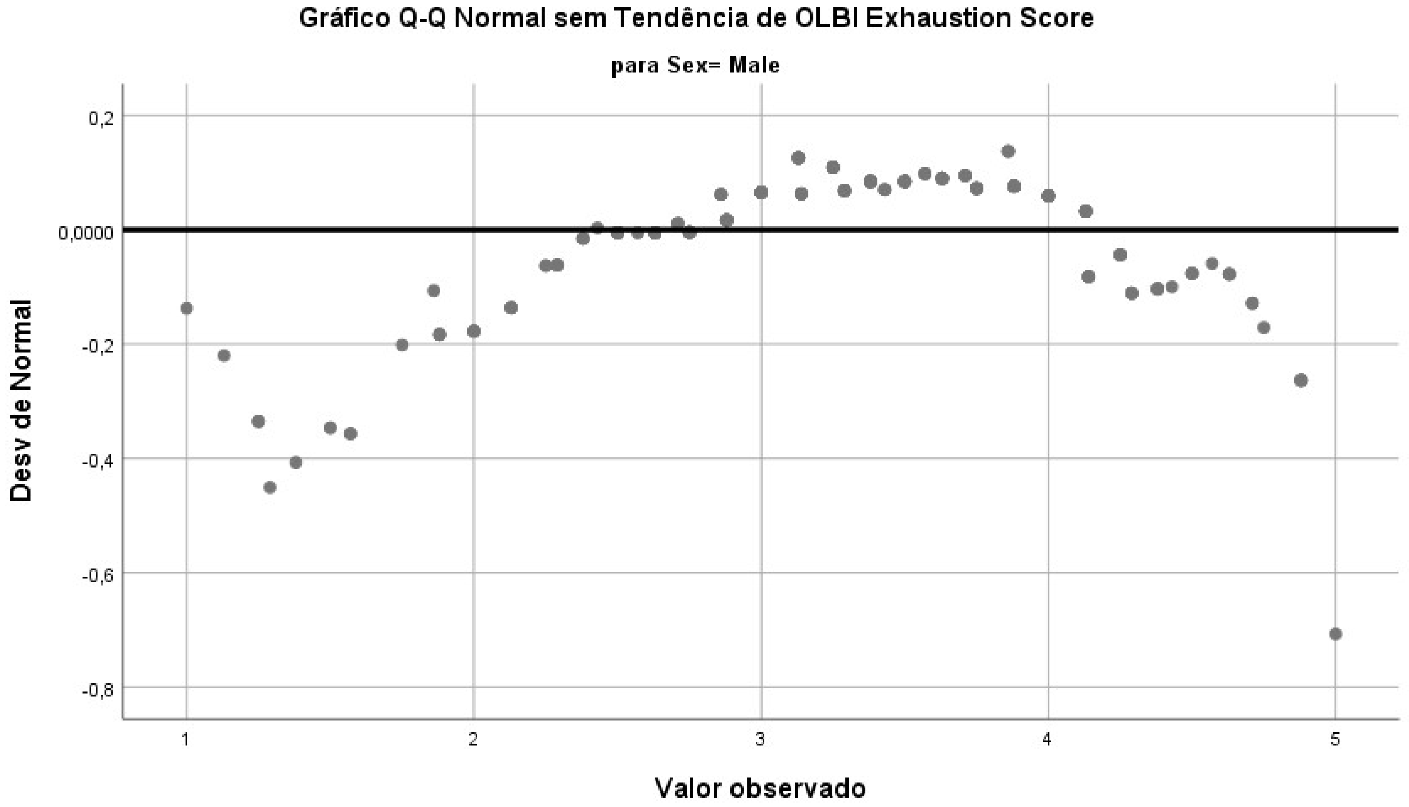

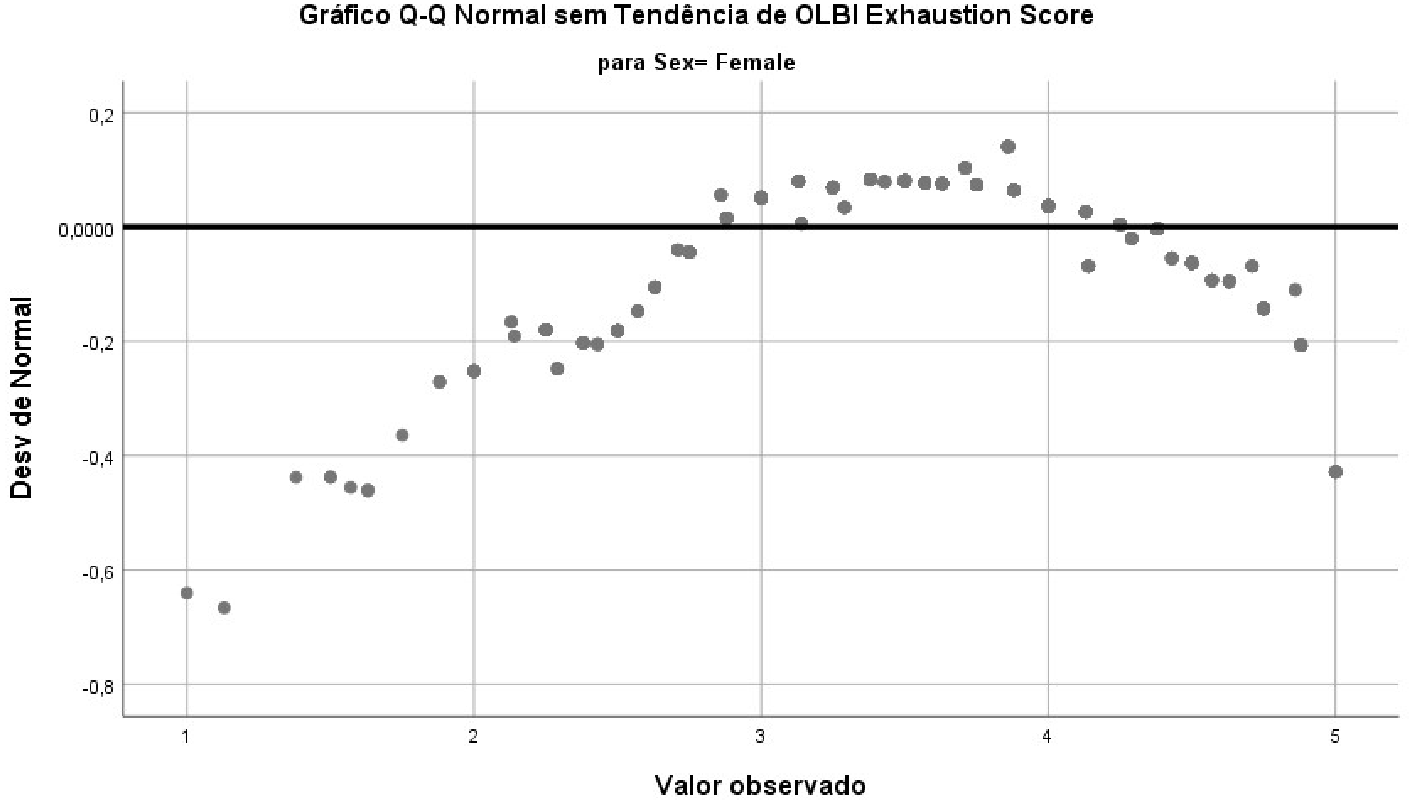

Any comorbidity (regardless COVID risk)

Resumo de processamento do caso

	Any comorbidity (regardless COVID risk)	Casos Válido N	Válido Porcentagem	Omisso N	Omisso Porcentagem	Total N	Total Porcentagem
OLBI Disengagement Score	No	1071	100,0%	0	0,0%	1071	100,0%
	Yes	234	100,0%	0	0,0%	234	100,0%
OLBI Exhaustion Score	No	1071	100,0%	0	0,0%	1071	100,0%
	Yes	234	100,0%	0	0,0%	234	100,0%

Descritivos

	Any comorbidity (regardless COVID risk)			Estatística	Erro Erro
OLBI Disengagement Score	No	Média		2,7317	,02510
		95% Intervalo de Confiança para Média	Limite inferior	2,6824	
			Limite superior	2,7809	
		5% da média aparada		2,7208	
		Mediana		2,6250	
		Variância		,675	
		Erro Desvio		,82132	
		Mínimo		1,00	
		Máximo		5,00	
		Intervalo		4,00	
		Amplitude interquartil		1,13	
		Assimetria		,192	,075
		Curtose		-,431	,149
	Yes	Média		2,9717	,05848
		95% Intervalo de Confiança para Média	Limite inferior	2,8565	
			Limite superior	3,0869	
		5% da média aparada		2,9666	
		Mediana		2,9375	
		Variância		,800	
		Erro Desvio		,89463	
		Mínimo		1,00	
		Máximo		5,00	
		Intervalo		4,00	
		Amplitude interquartil		1,28	
		Assimetria		,078	,159
		Curtose		-,483	,317
OLBI Exhaustion Score	No	Média		3,5151	,02249
		95% Intervalo de Confiança	Limite inferior	3,4710	

		para Média	Limite superior	3,5592	
		5% da média aparada		3,5393	
		Mediana		3,5700	
		Variância		,541	
		Erro Desvio		,73586	
		Mínimo		1,00	
		Máximo		5,00	
		Intervalo		4,00	
		Amplitude interquartil		,87	
		Assimetria		-,508	,075
		Curtose		,213	,149
	Yes	Média		3,7252	,04726
		95% Intervalo de Confiança para Média	Limite inferior	3,6321	
			Limite superior	3,8183	
		5% da média aparada		3,7534	
		Mediana		3,8600	
		Variância		,523	
		Erro Desvio		,72293	
		Mínimo		1,50	
		Máximo		5,00	
		Intervalo		3,50	
		Amplitude interquartil		,75	
		Assimetria		-,619	,159
		Curtose		,337	,317

Percentis

		Any comorbidity (regardless COVID risk)	Percentis 5	10	25	50	75	90	95
Média Ponderada (Definição 1)	OLBI Disengagement Score	No	1,3750	1,6250	2,1250	2,6250	3,2500	3,8750	4,1250
		Yes	1,3750	1,7500	2,3438	2,9375	3,6250	4,2500	4,5000
	OLBI Exhaustion Score	No	2,2500	2,5000	3,1300	3,5700	4,0000	4,3800	4,6300
		Yes	2,2500	2,6700	3,3800	3,8600	4,1300	4,6300	4,8800
Teste de Tukey	OLBI Disengagement Score	No			2,1250	2,6250	3,2500		
		Yes			2,3750	2,9375	3,6250		
	OLBI Exhaustion Score	No			3,1300	3,5700	4,0000		
		Yes			3,3800	3,8600	4,1300		

Testes de Normalidade

	Any comorbidity (regardless COVID risk)	Kolmogorov-Smirnov[a] Estatística	df	Sig.	Shapiro-Wilk Estatística	df	Sig.
OLBI Disengagement Score	No	,059	1071	,000	,990	1071	,000
	Yes	,047	234	,200[*]	,990	234	,091
OLBI Exhaustion Score	No	,062	1071	,000	,981	1071	,000

Yes	,093	234	,000	,963	234	,000

*. Este é um limite inferior da significância verdadeira.

a. Correlação de Significância de Lilliefors

OLBI Disengagement Score

Histogramas

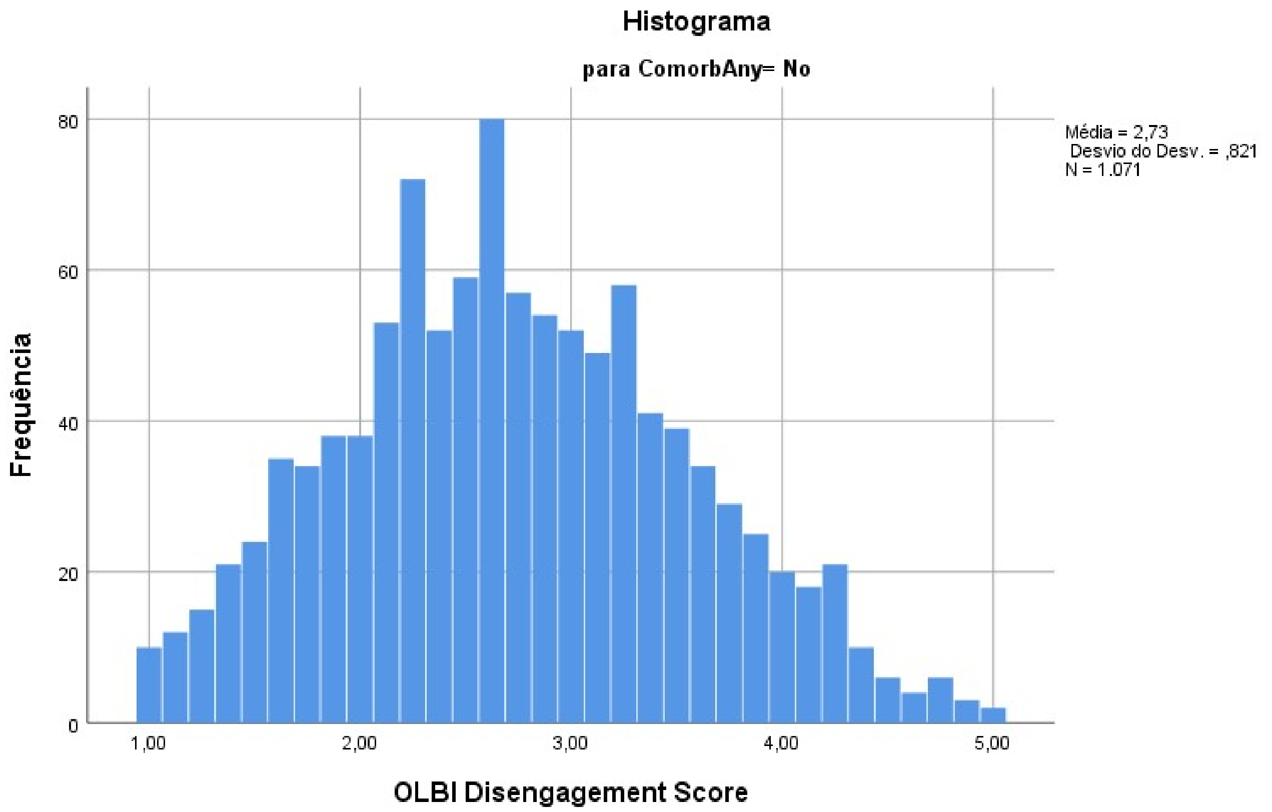

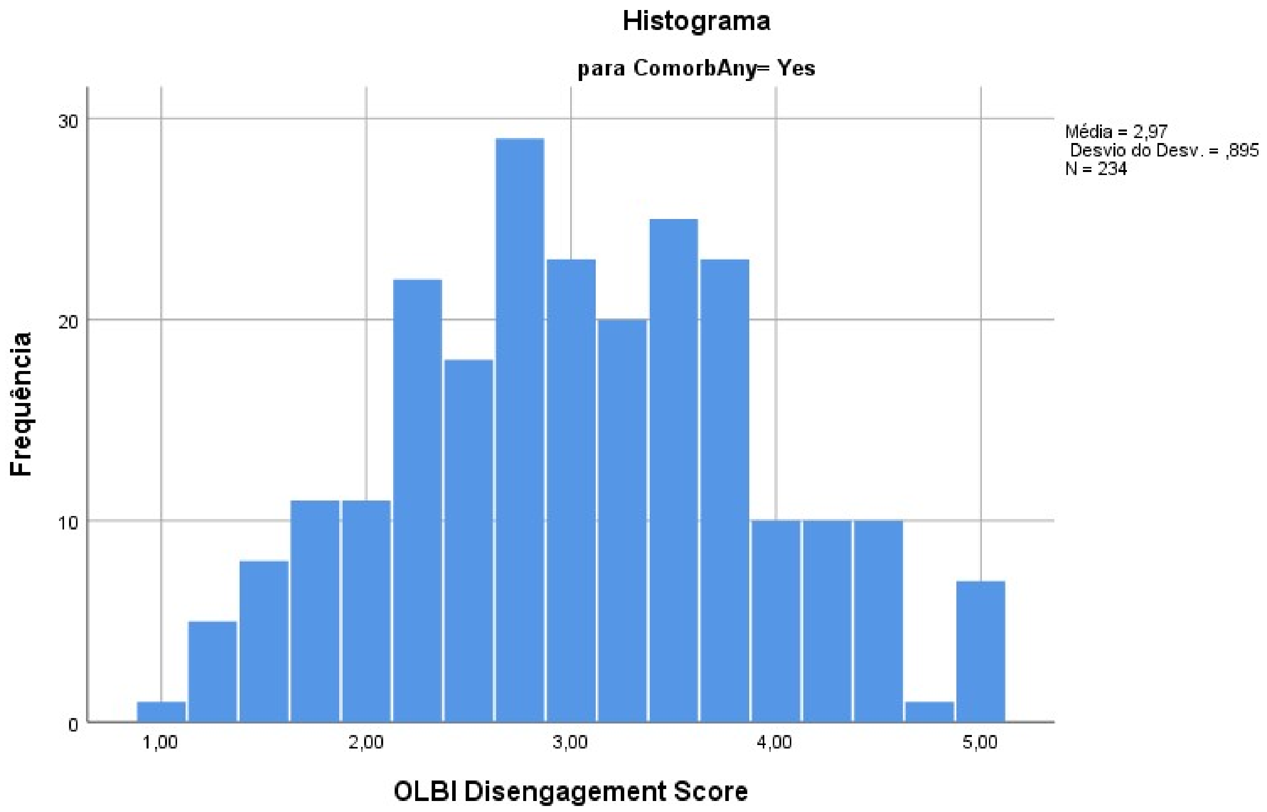

Gráfico Q-Q normais

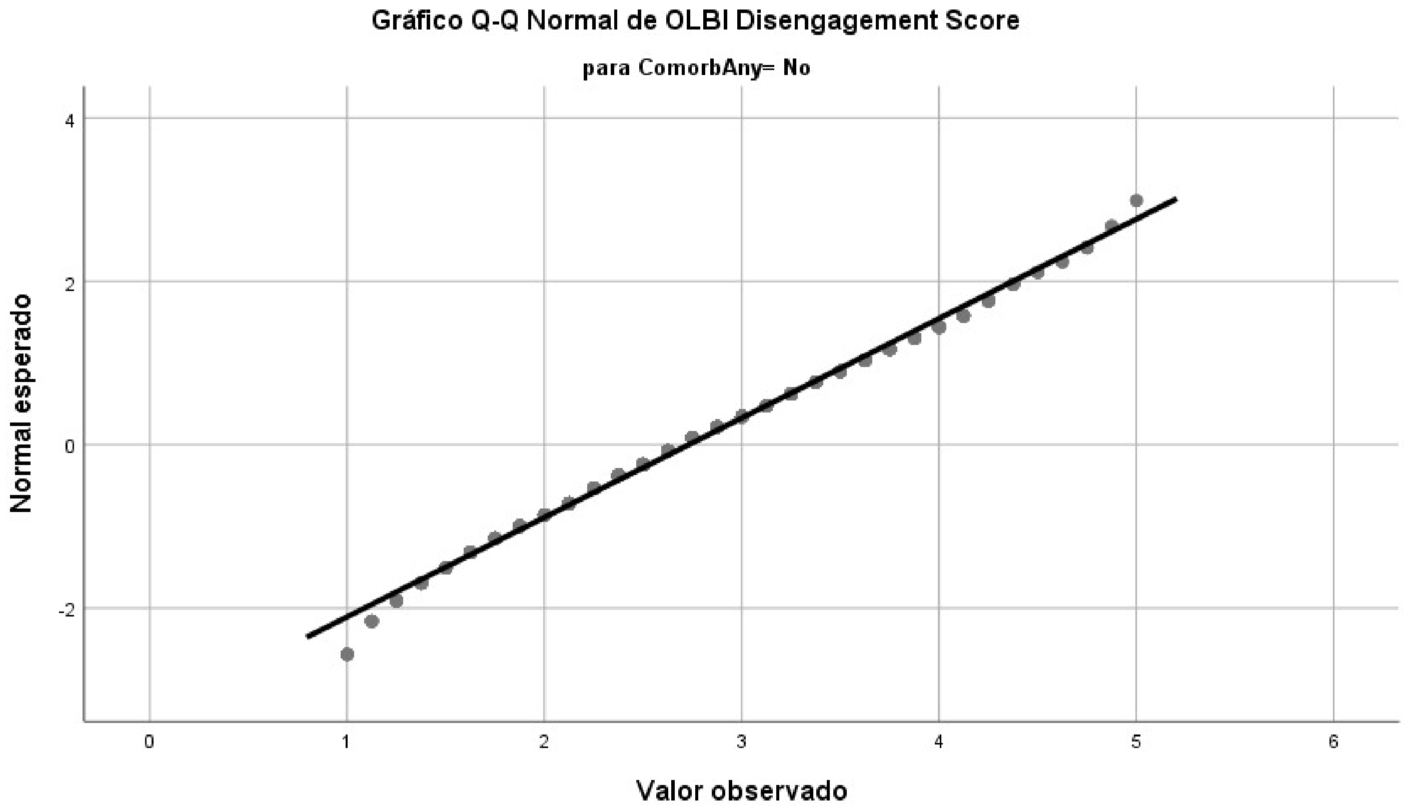

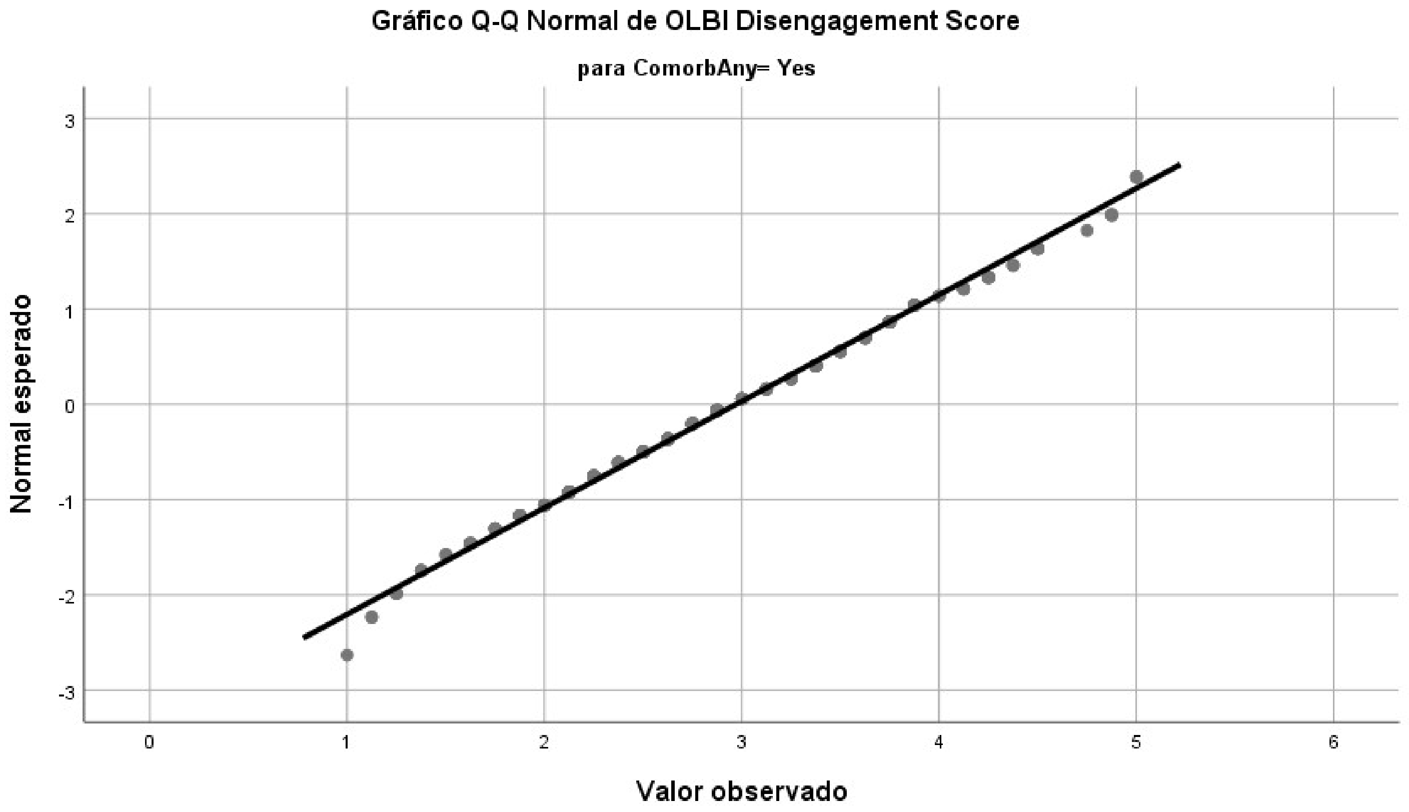

Gráfico Q-Q normais sem tendência

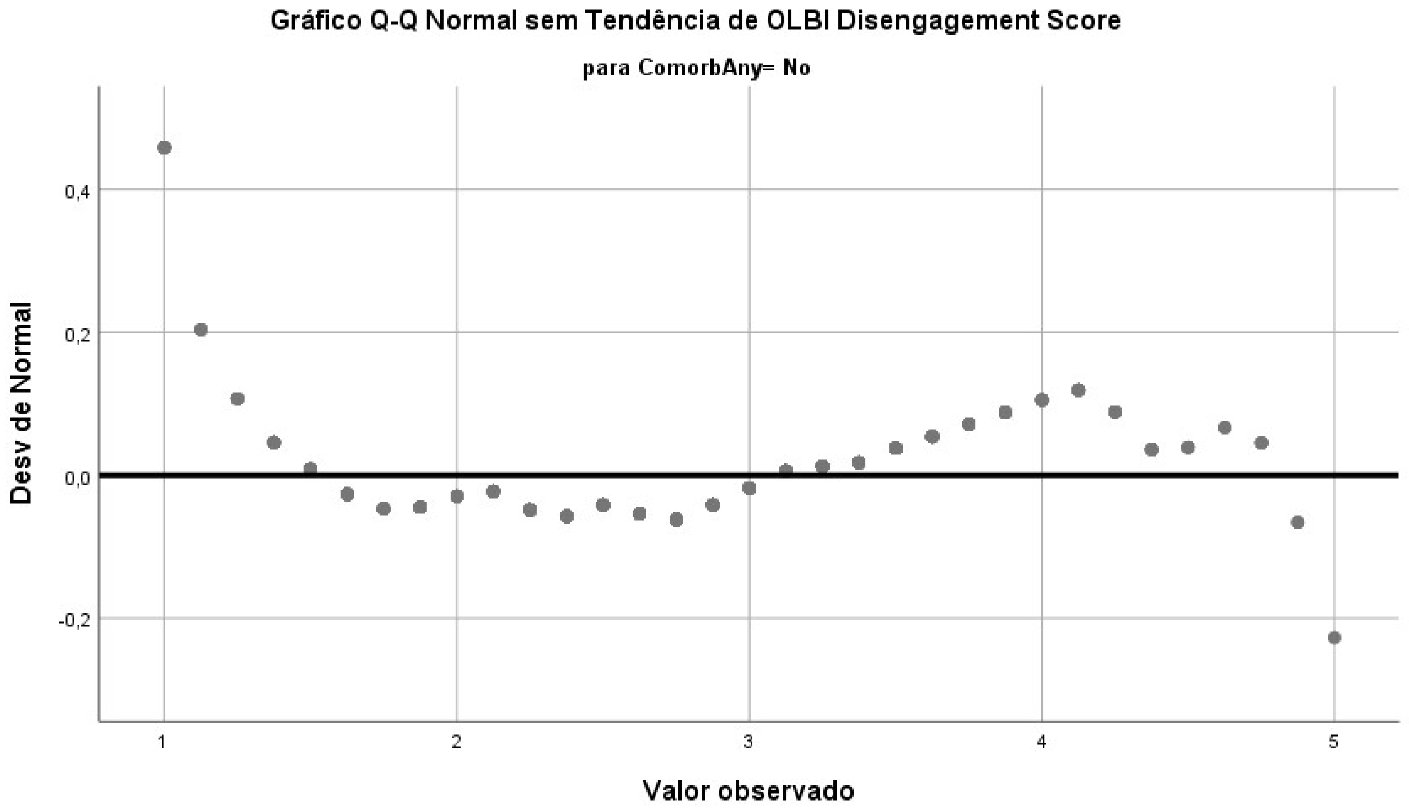

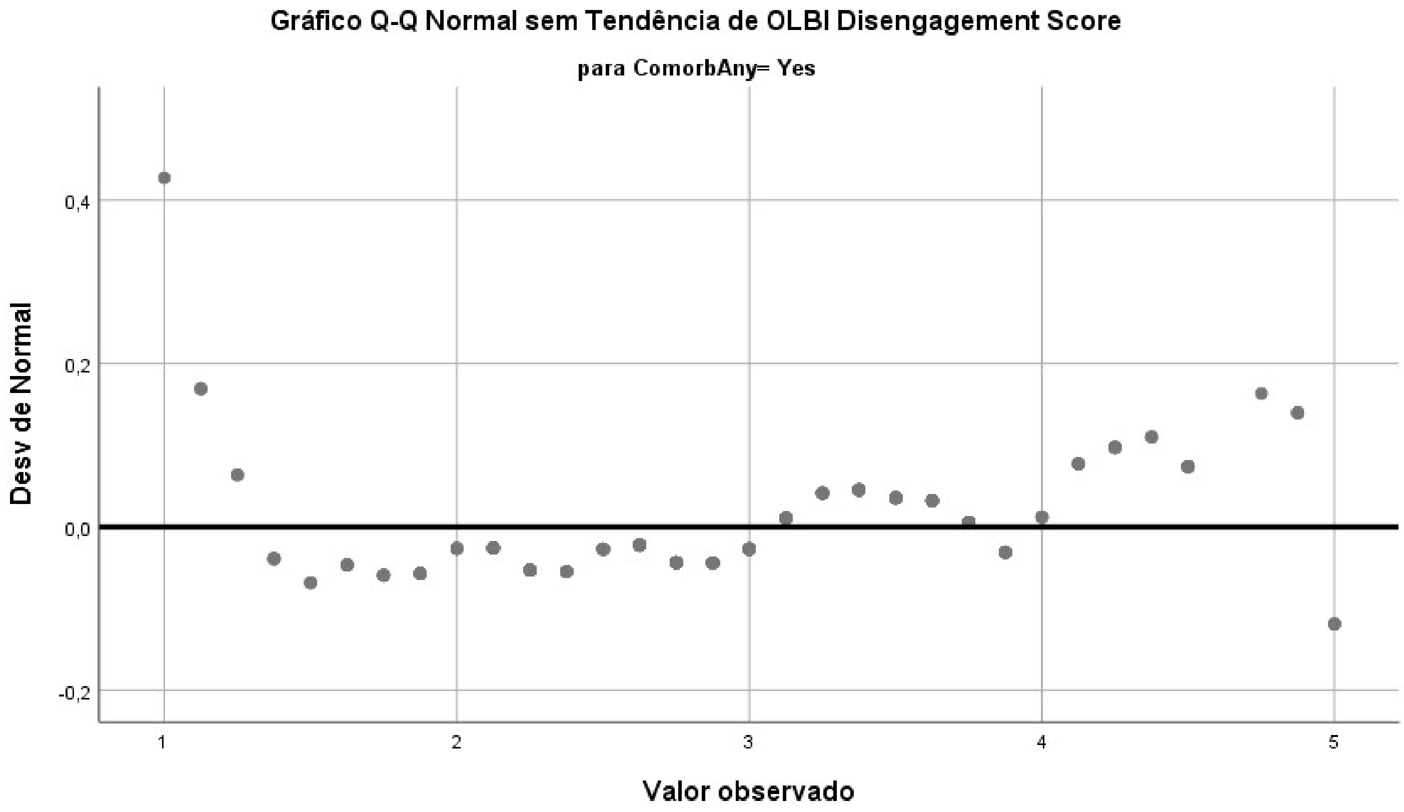

OLBI Exhaustion Score

Histogramas

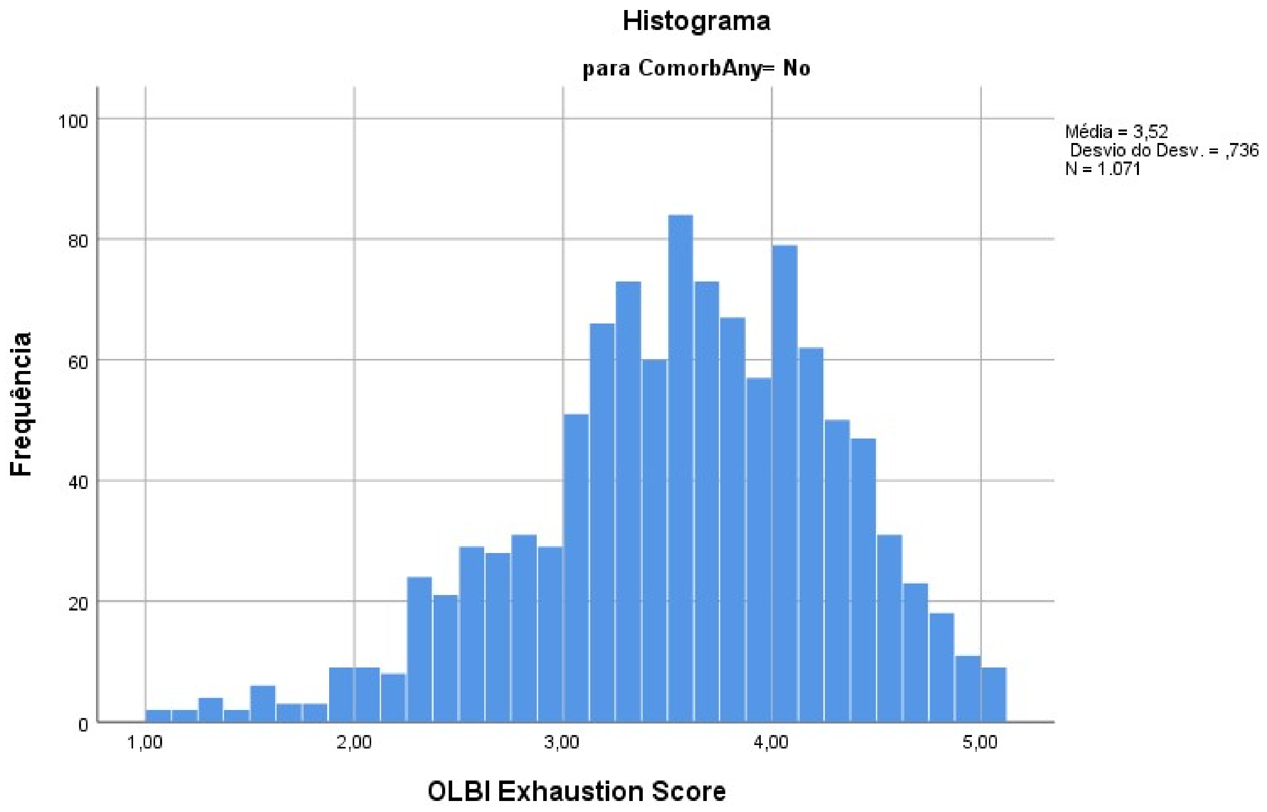

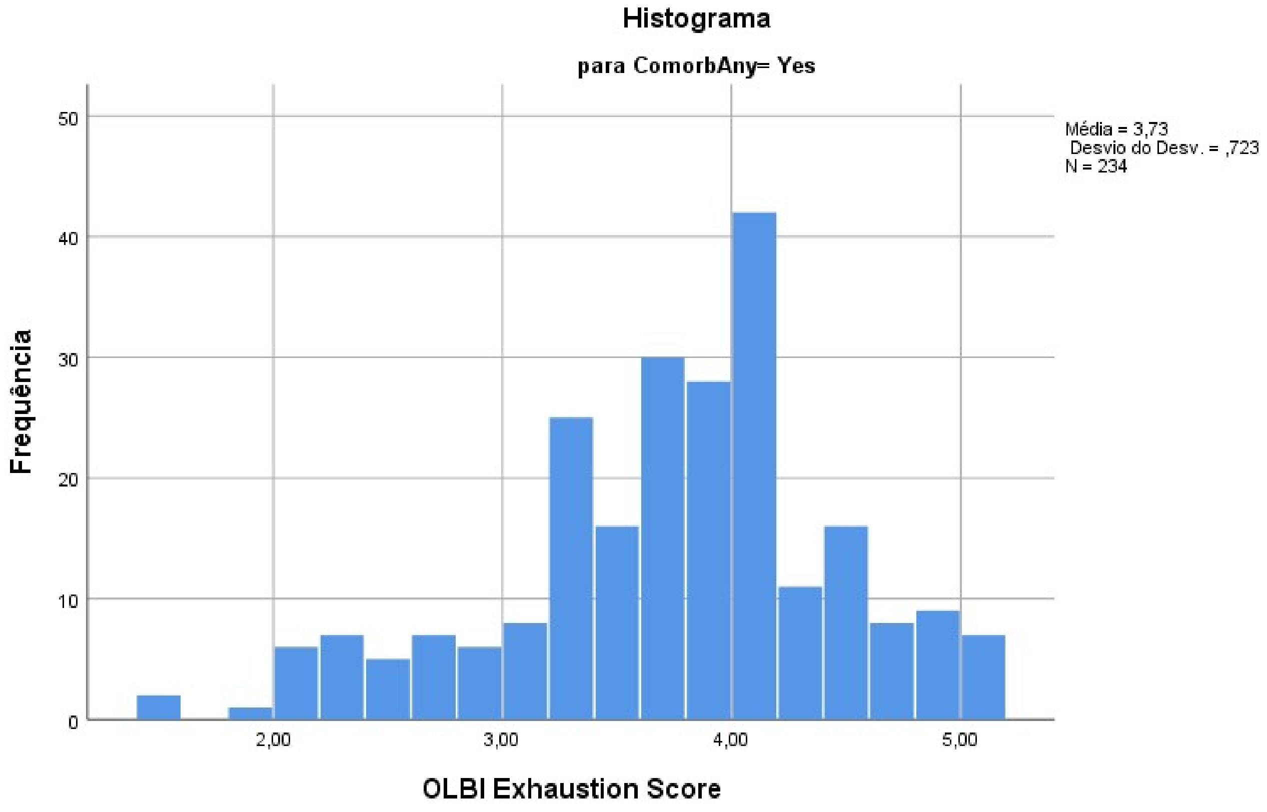

Gráfico Q-Q normais

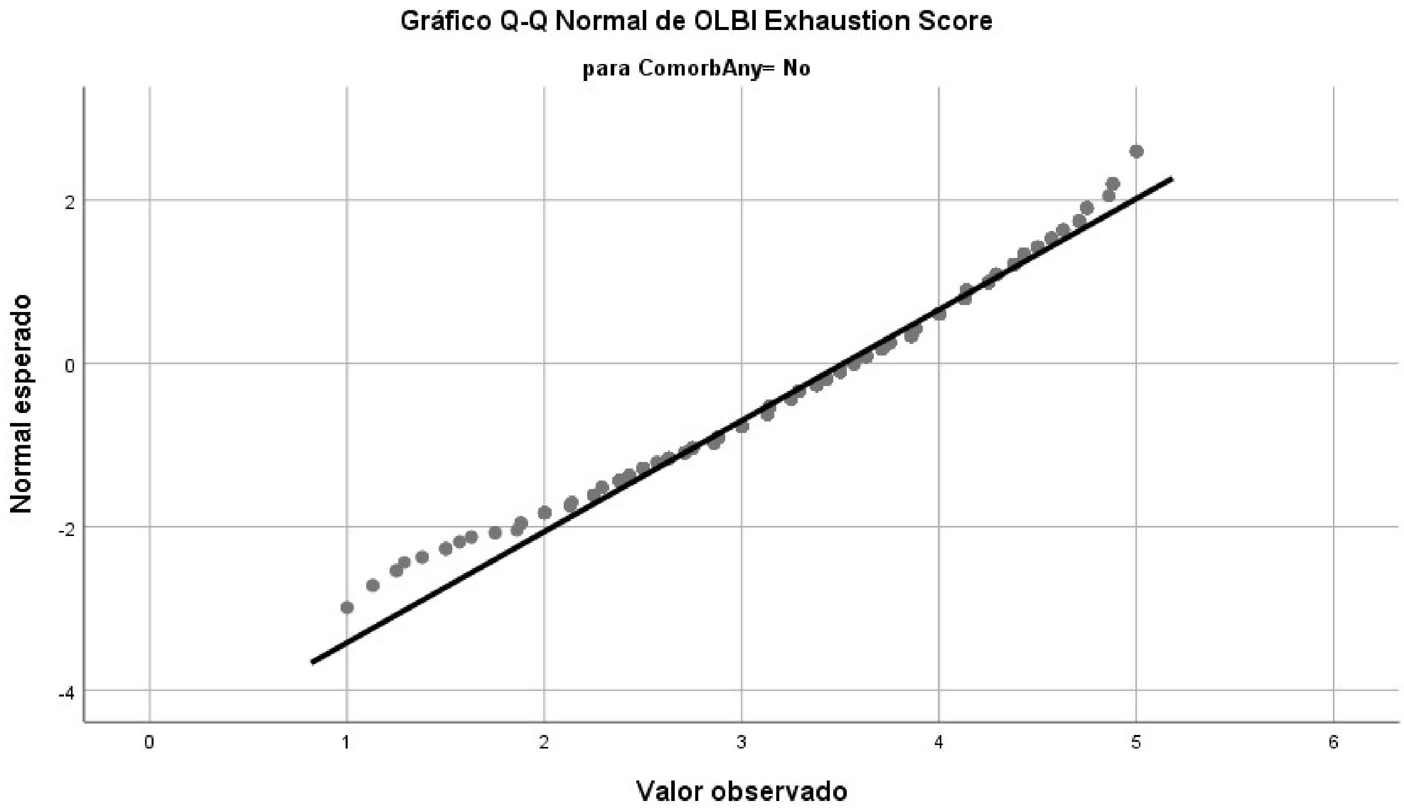

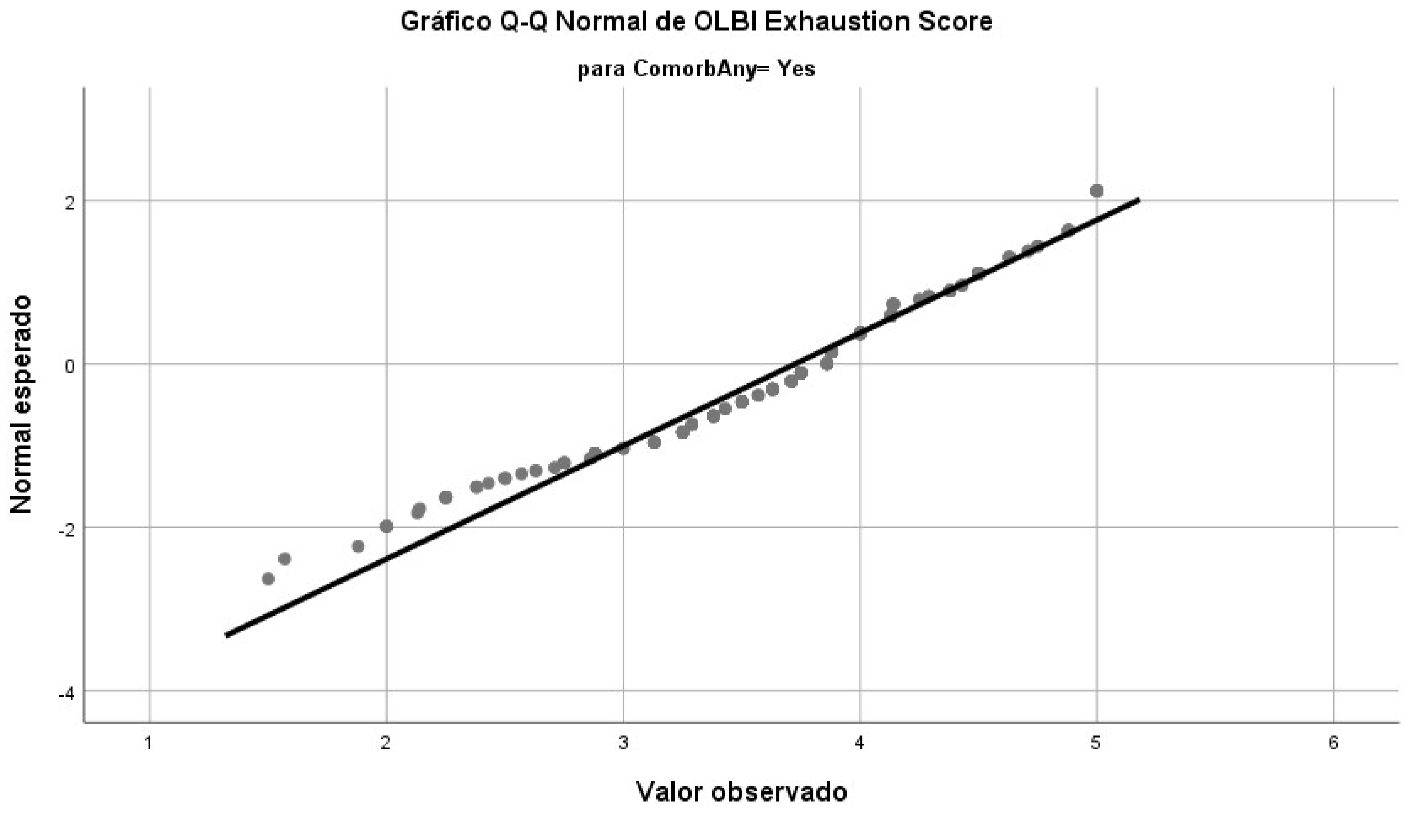

Gráfico Q-Q normais sem tendência

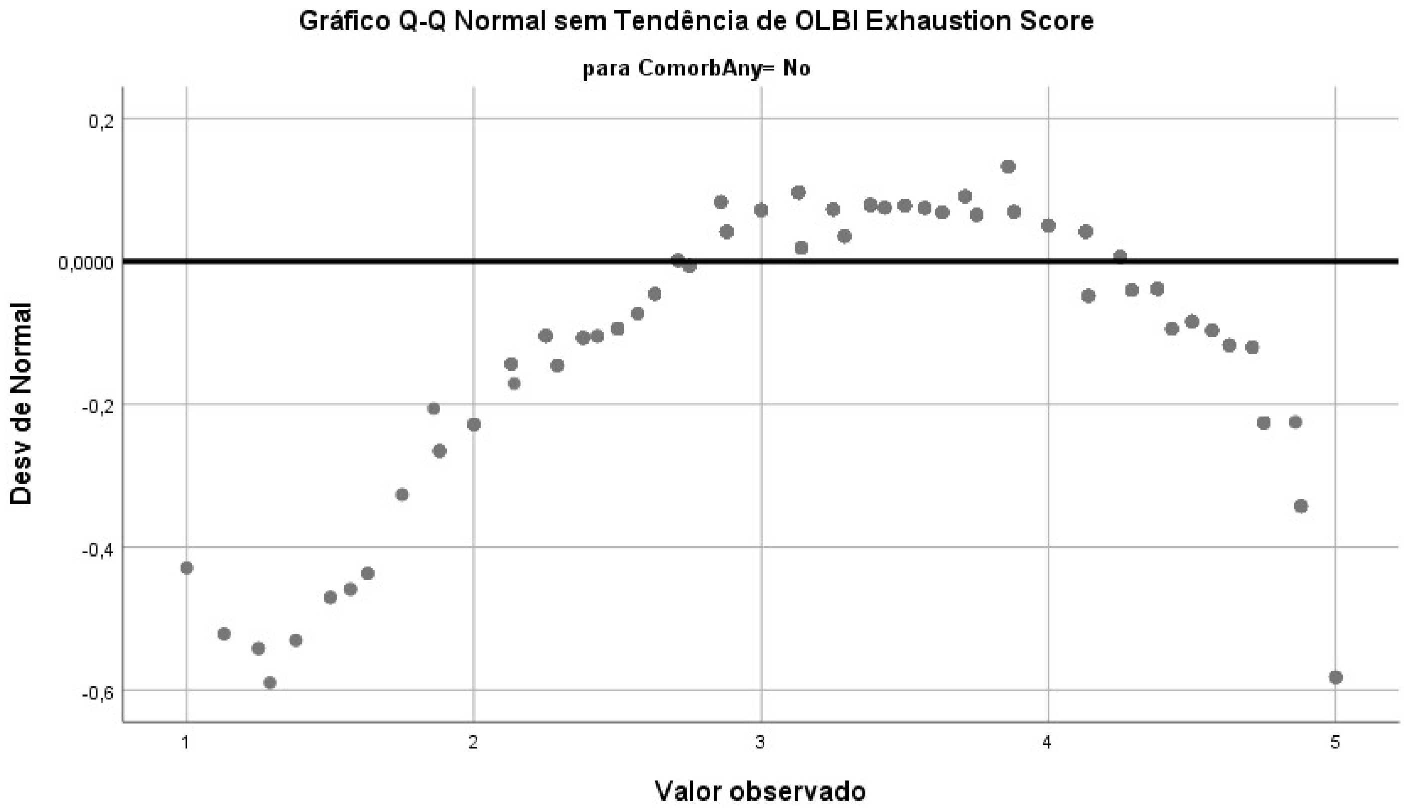

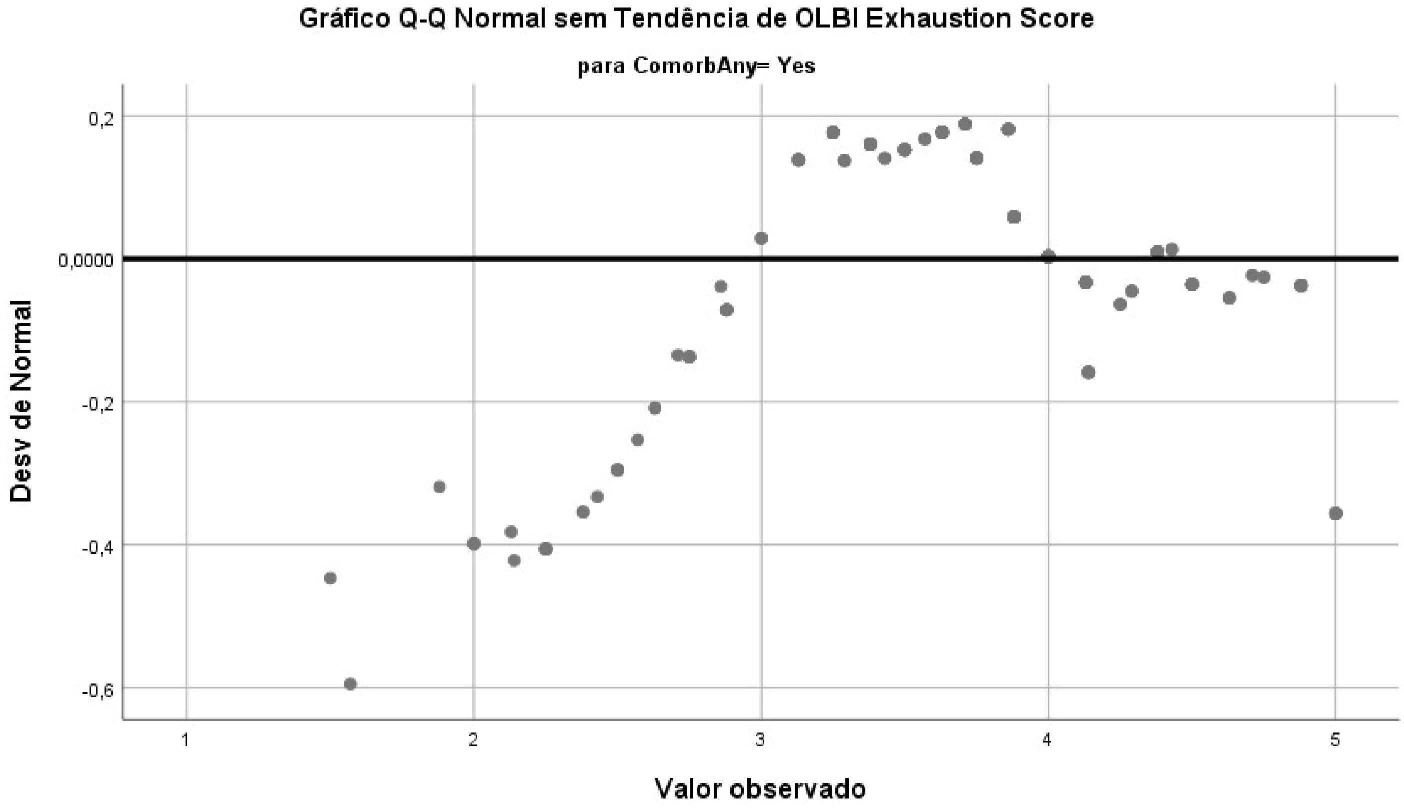

BRCS Classification

Resumo de processamento do caso

	BRCS Classification	Casos					
		Válido		Omisso		Total	
		N	Porcentagem	N	Porcentagem	N	Porcentagem
OLBI Disengagement Score	Low resilience	813	100,0%	0	0,0%	813	100,0%
	Moderate to High	500	100,0%	0	0,0%	500	100,0%
OLBI Exhaustion Score	Low resilience	813	100,0%	0	0,0%	813	100,0%
	Moderate to High	500	100,0%	0	0,0%	500	100,0%

Descritivos

	BRCS Classification			Estatística	Erro Erro
OLBI Disengagement Score	Low resilience	Média		2,9845	,02784
		95% Intervalo de Confiança para Média	Limite inferior	2,9298	
			Limite superior	3,0391	
		5% da média aparada		2,9773	
		Mediana		3,0000	
		Variância		,630	
		Erro Desvio		,79388	
		Mínimo		1,13	
		Máximo		5,00	
		Intervalo		3,88	
		Amplitude interquartil		1,13	
		Assimetria		,153	,086
		Curtose		-,439	,171
	Moderate to High	Média		2,4318	,03572
		95% Intervalo de Confiança para Média	Limite inferior	2,3616	
			Limite superior	2,5019	
		5% da média aparada		2,4086	
		Mediana		2,3750	
		Variância		,638	
		Erro Desvio		,79865	
		Mínimo		1,00	
		Máximo		5,00	
		Intervalo		4,00	
		Amplitude interquartil		1,13	
		Assimetria		,388	,109
		Curtose		-,208	,218
OLBI Exhaustion Score	Low resilience	Média		3,7136	,02398
		95% Intervalo de Confiança para Média	Limite inferior	3,6665	
			Limite superior	3,7606	
		5% da média aparada		3,7349	
		Mediana		3,7500	
		Variância		,467	

		Erro Desvio		,68374	
		Mínimo		1,25	
		Máximo		5,00	
		Intervalo		3,75	
		Amplitude interquartil		,89	
		Assimetria		-,489	,086
		Curtose		,114	,171
	Moderate to High	Média		3,2961	,03336
		95% Intervalo de Confiança para Média	Limite inferior	3,2306	
			Limite superior	3,3617	
		5% da média aparada		3,3209	
		Mediana		3,3800	
		Variância		,556	
		Erro Desvio		,74592	
		Mínimo		1,00	
		Máximo		5,00	
		Intervalo		4,00	
		Amplitude interquartil		1,00	
		Assimetria		-,525	,109
		Curtose		,164	,218

Percentis

			Percentis						
		BRCS Classification	5	10	25	50	75	90	95
Média Ponderada (Definição 1)	OLBI Disengagement Score	Low resilience	1,7500	2,0000	2,3750	3,0000	3,5000	4,1250	4,3750
		Moderate to High	1,2500	1,3750	1,8750	2,3750	3,0000	3,5000	3,8750
	OLBI Exhaustion Score	Low resilience	2,5000	2,7500	3,2500	3,7500	4,1400	4,5700	4,7500
		Moderate to High	2,0000	2,2500	2,8800	3,3800	3,8800	4,1300	4,3800
Teste de Tukey	OLBI Disengagement Score	Low resilience			2,3750	3,0000	3,5000		
		Moderate to High			1,8750	2,3750	3,0000		
	OLBI Exhaustion Score	Low resilience			3,2500	3,7500	4,1400		
		Moderate to High			2,8800	3,3800	3,8800		

Testes de Normalidade

		Kolmogorov-Smirnov[a]			Shapiro-Wilk		
	BRCS Classification	Estatística	df	Sig.	Estatística	df	Sig.
OLBI Disengagement Score	Low resilience	,058	813	,000	,991	813	,000
	Moderate to High	,063	500	,000	,981	500	,000
OLBI Exhaustion Score	Low resilience	,063	813	,000	,980	813	,000
	Moderate to High	,078	500	,000	,978	500	,000

a. Correlação de Significância de Lilliefors

OLBI Disengagement Score

Histogramas

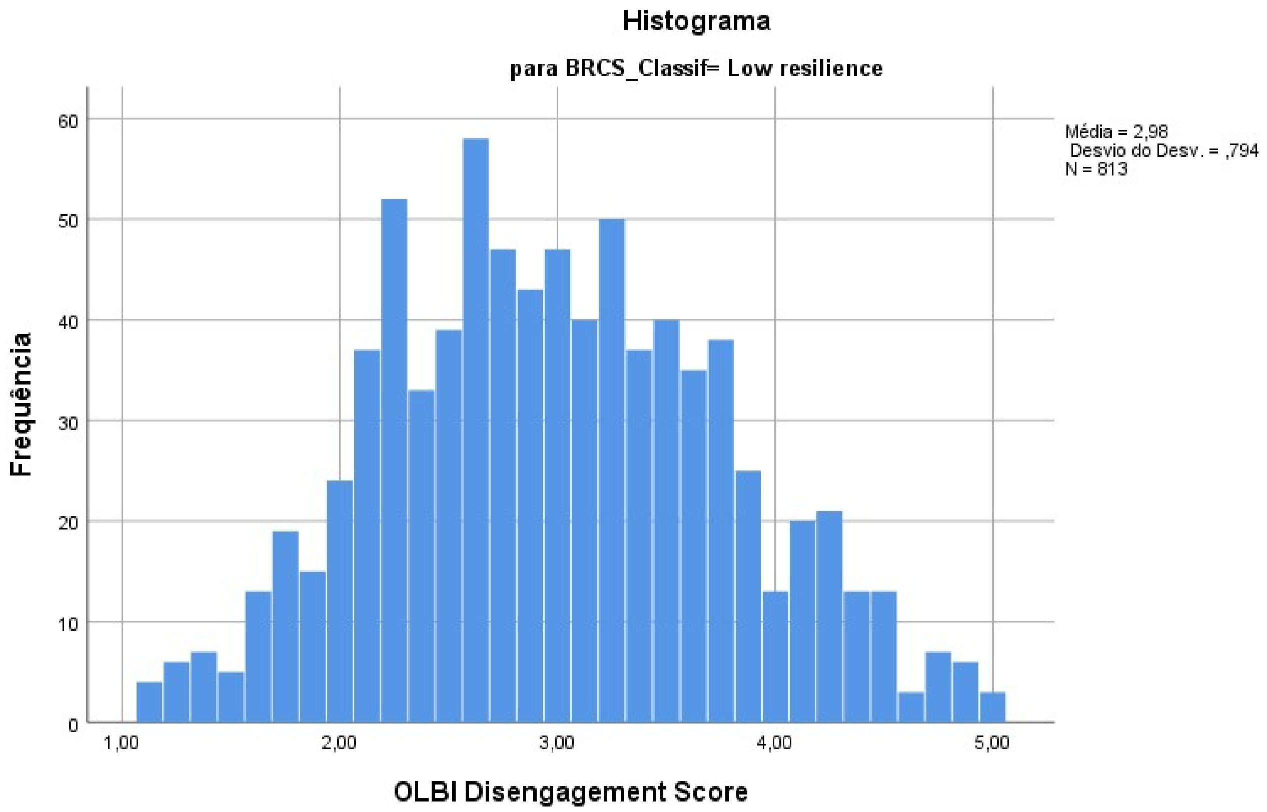

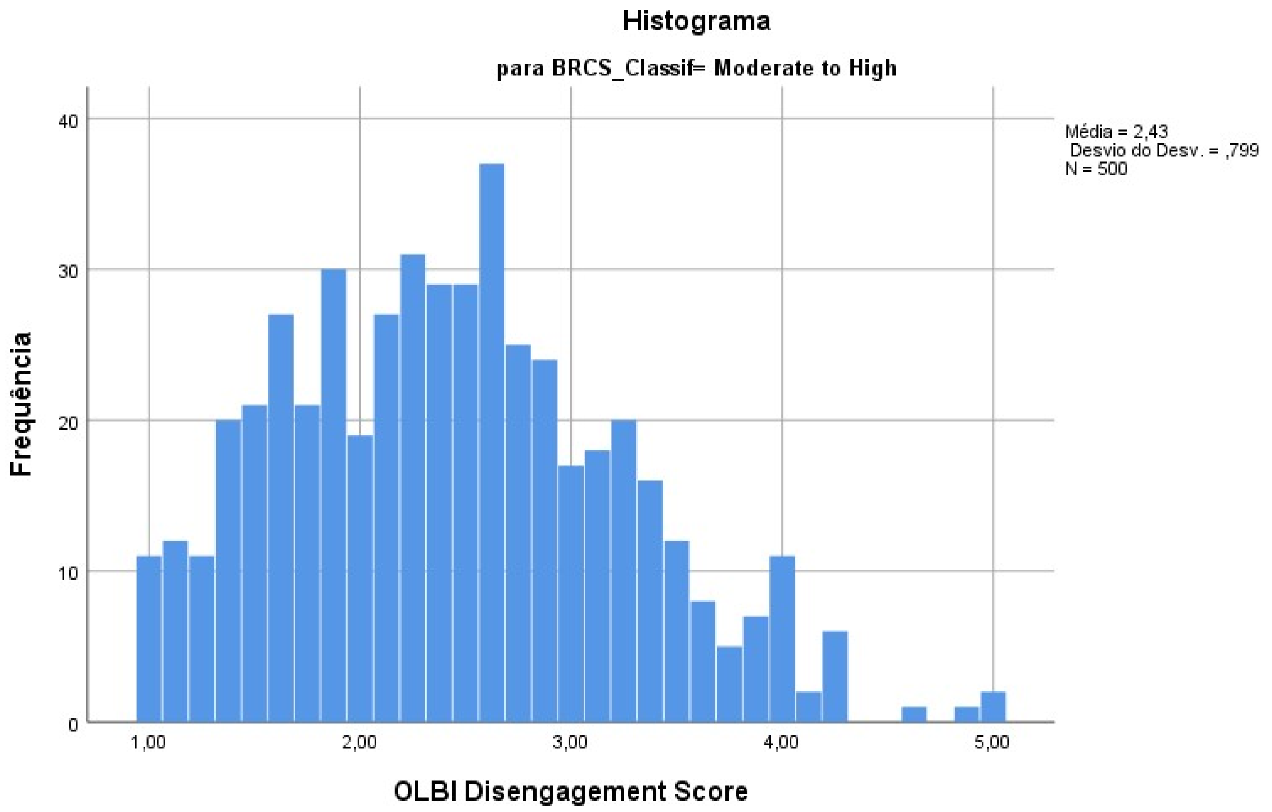

Gráfico Q-Q normais

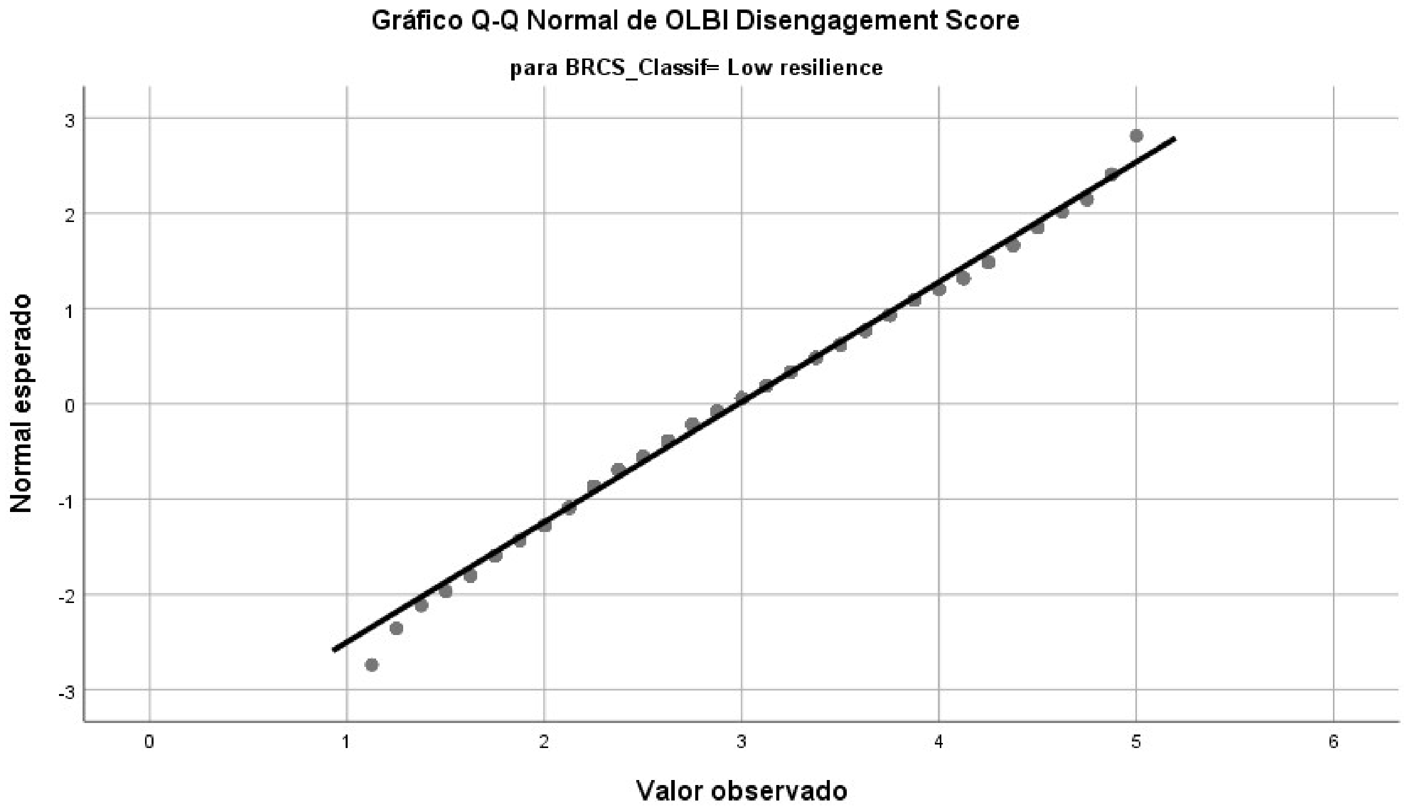

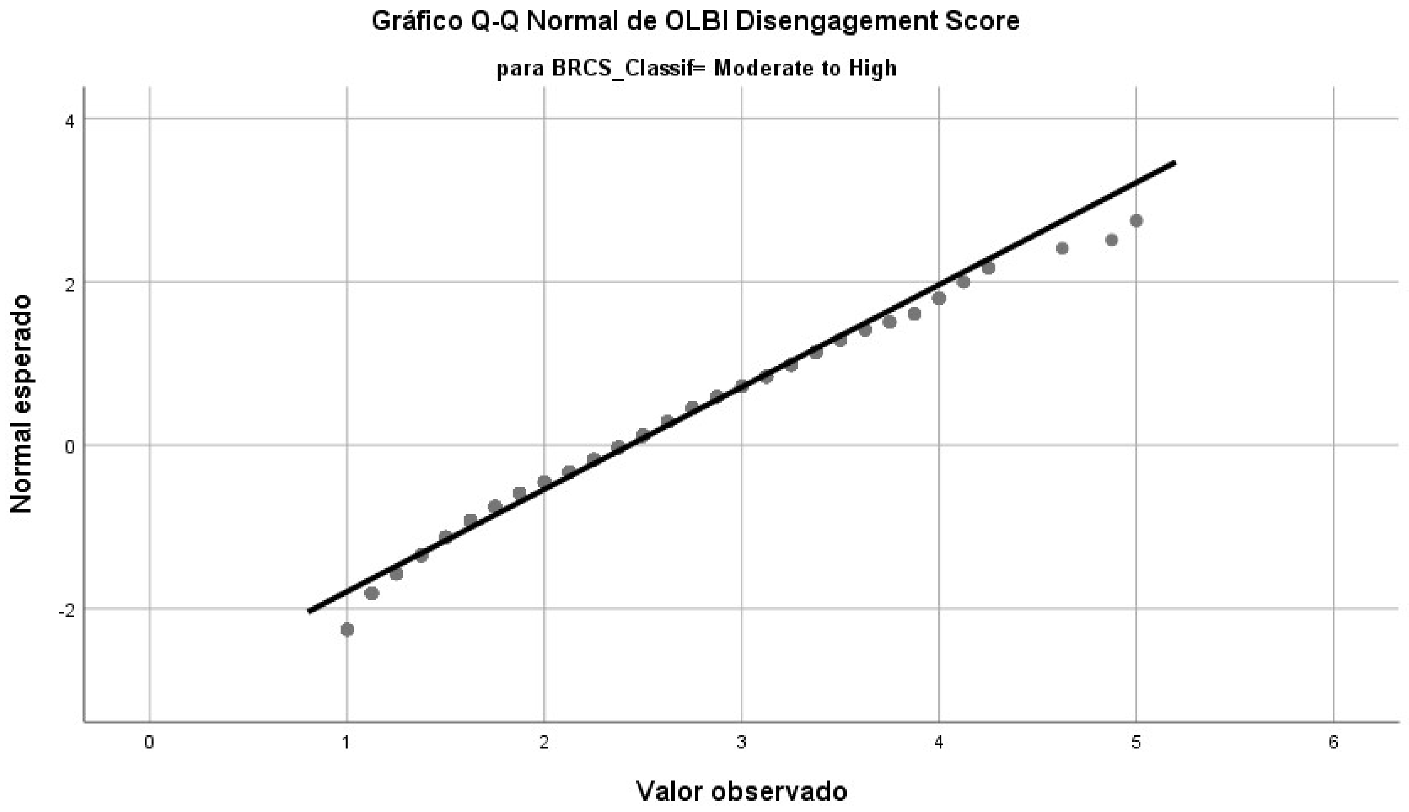

Gráfico Q-Q normais sem tendência

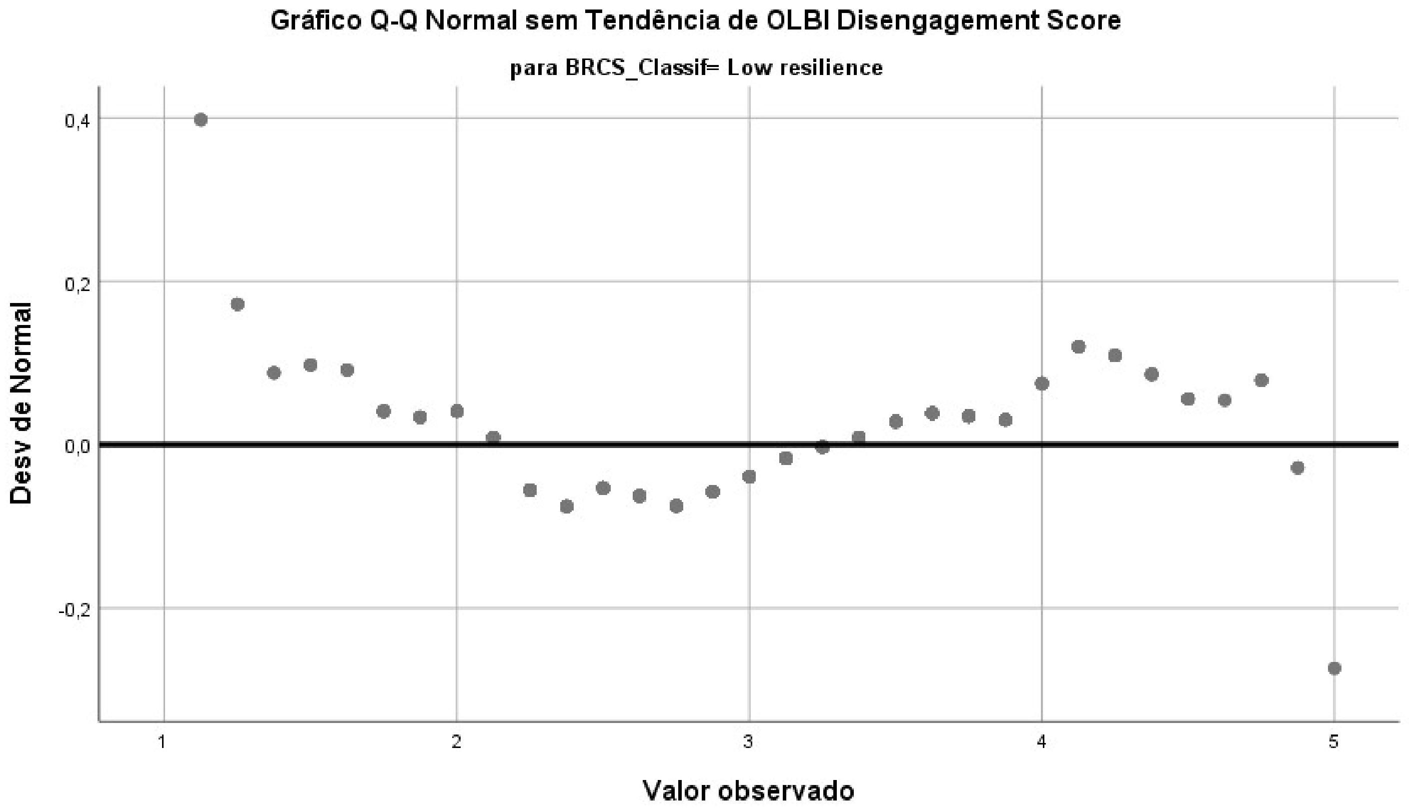

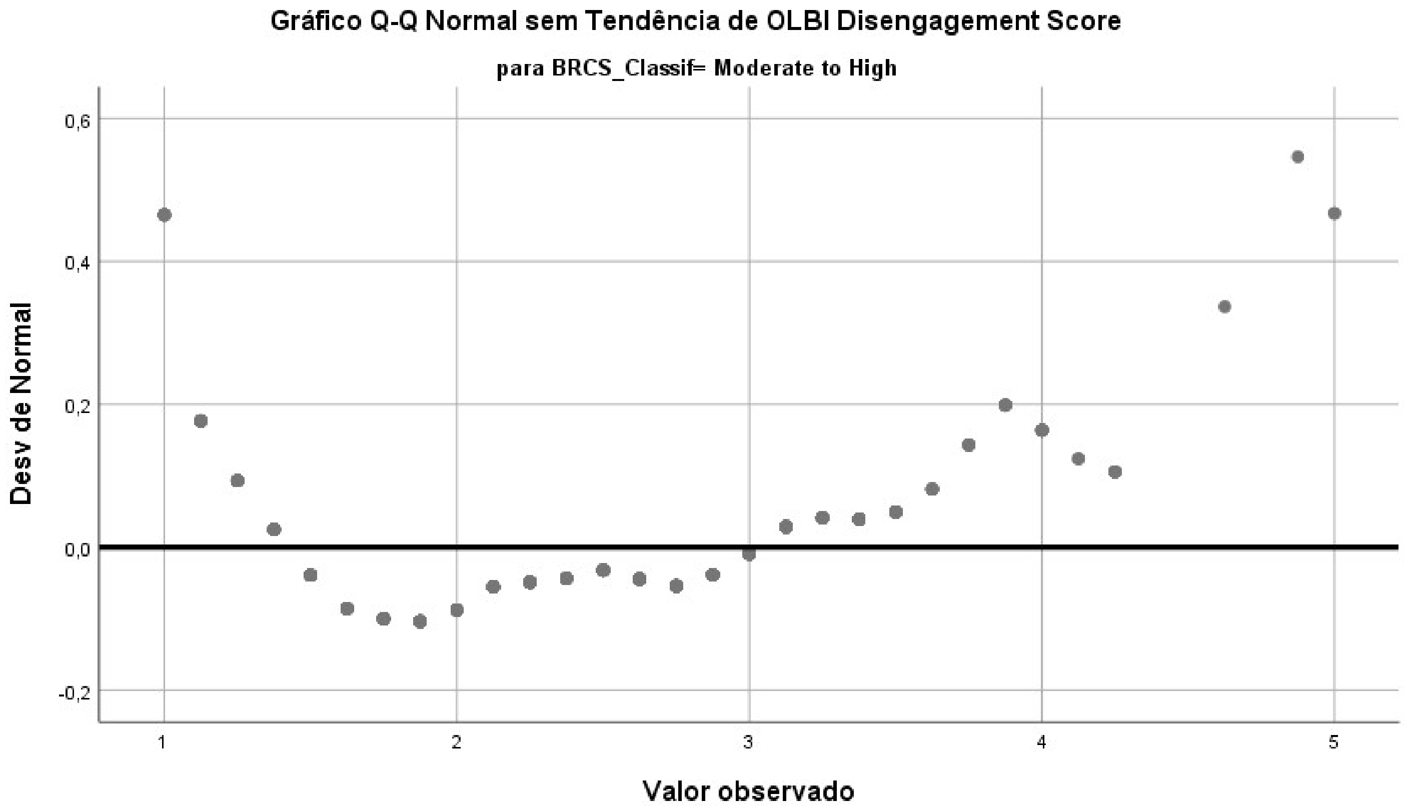

OLBI Exhaustion Score

Histogramas

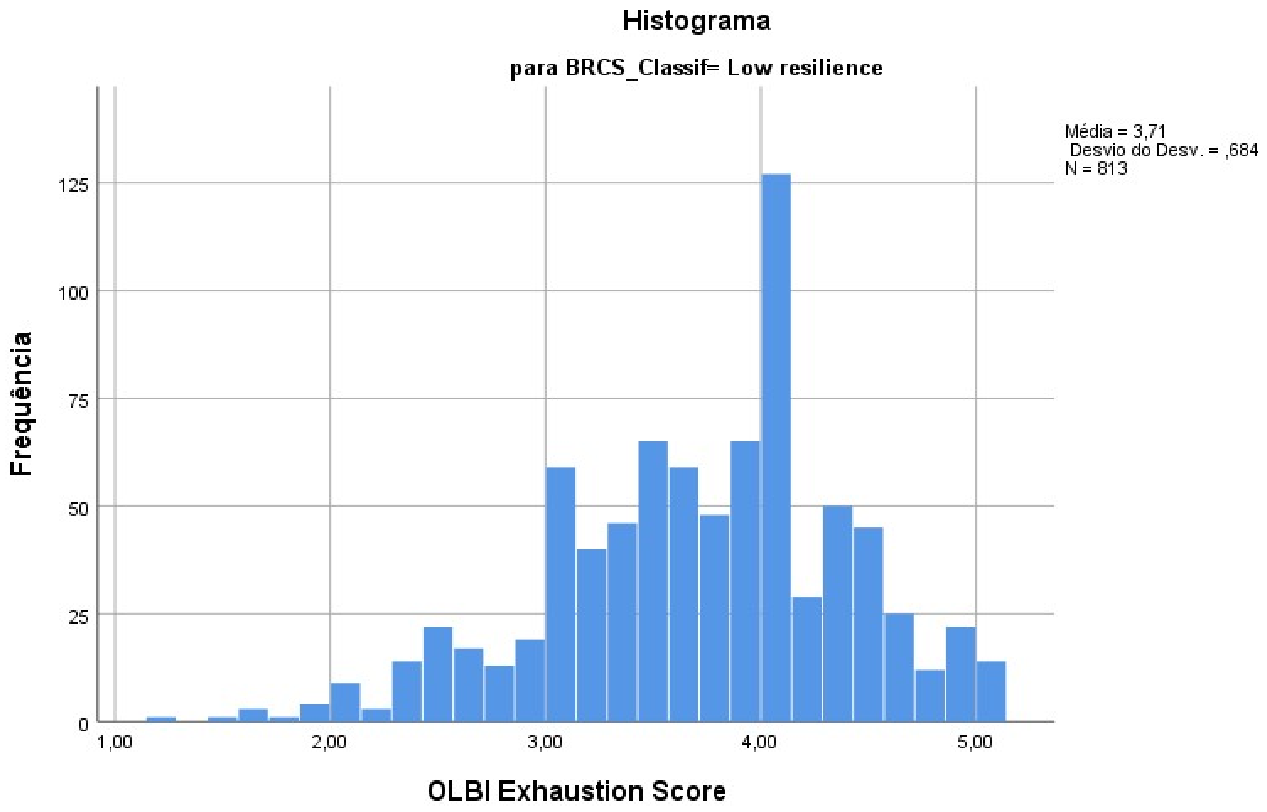

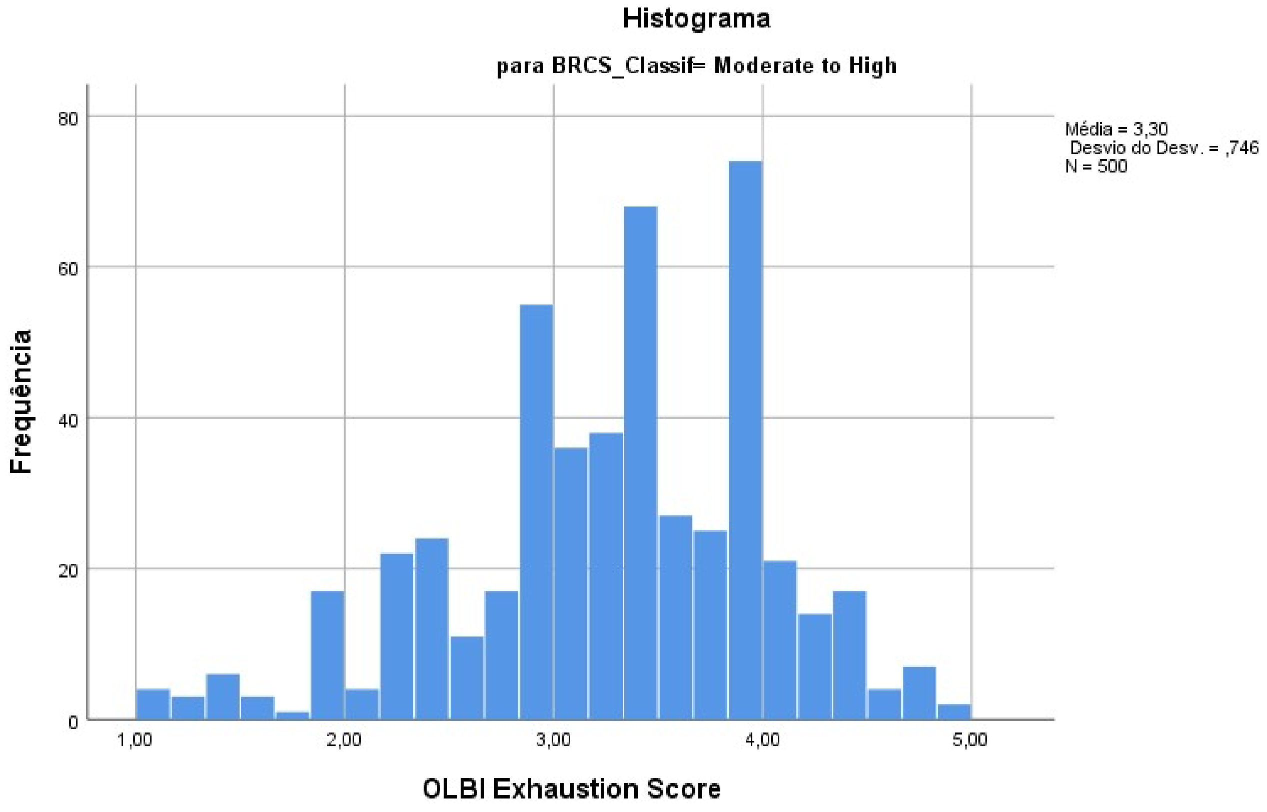

Gráfico Q-Q normais

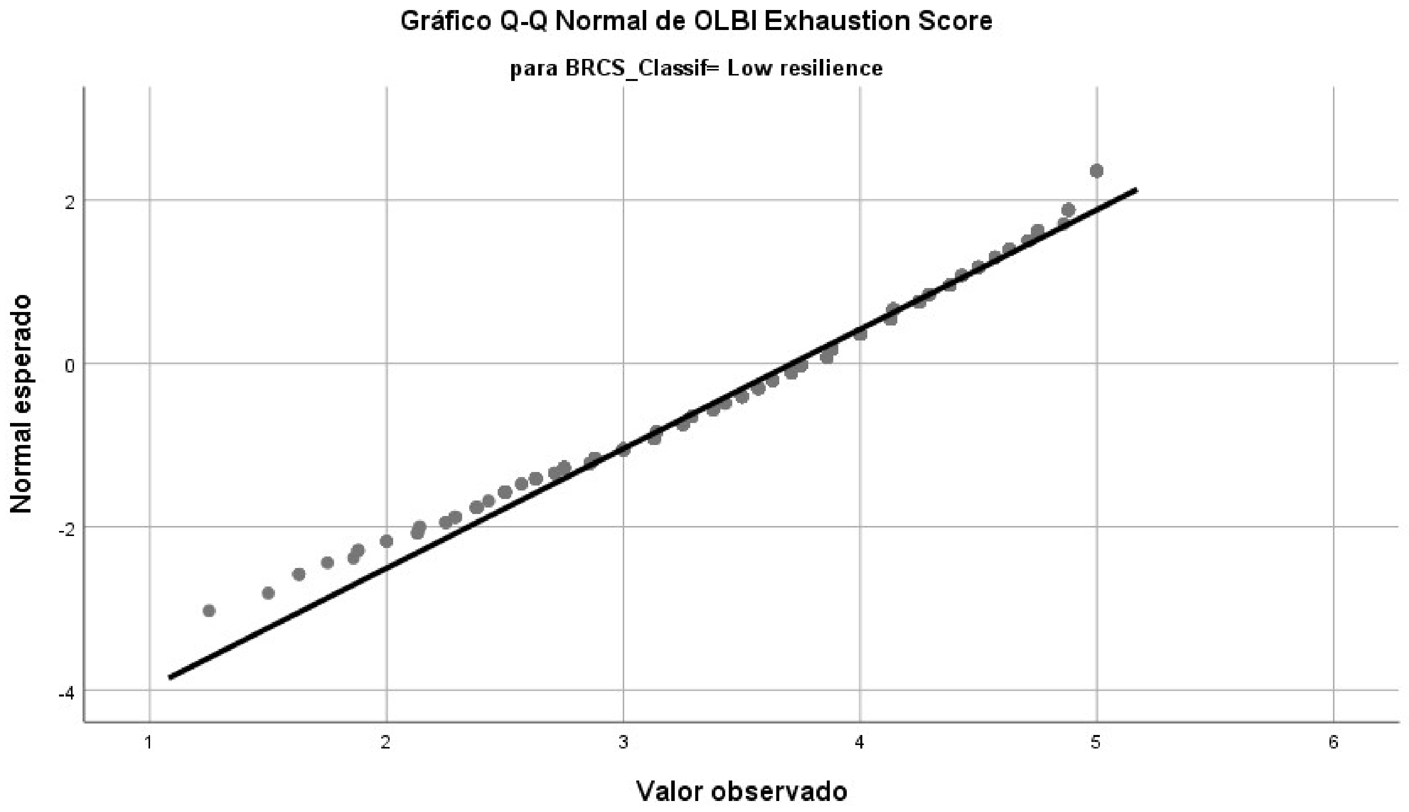

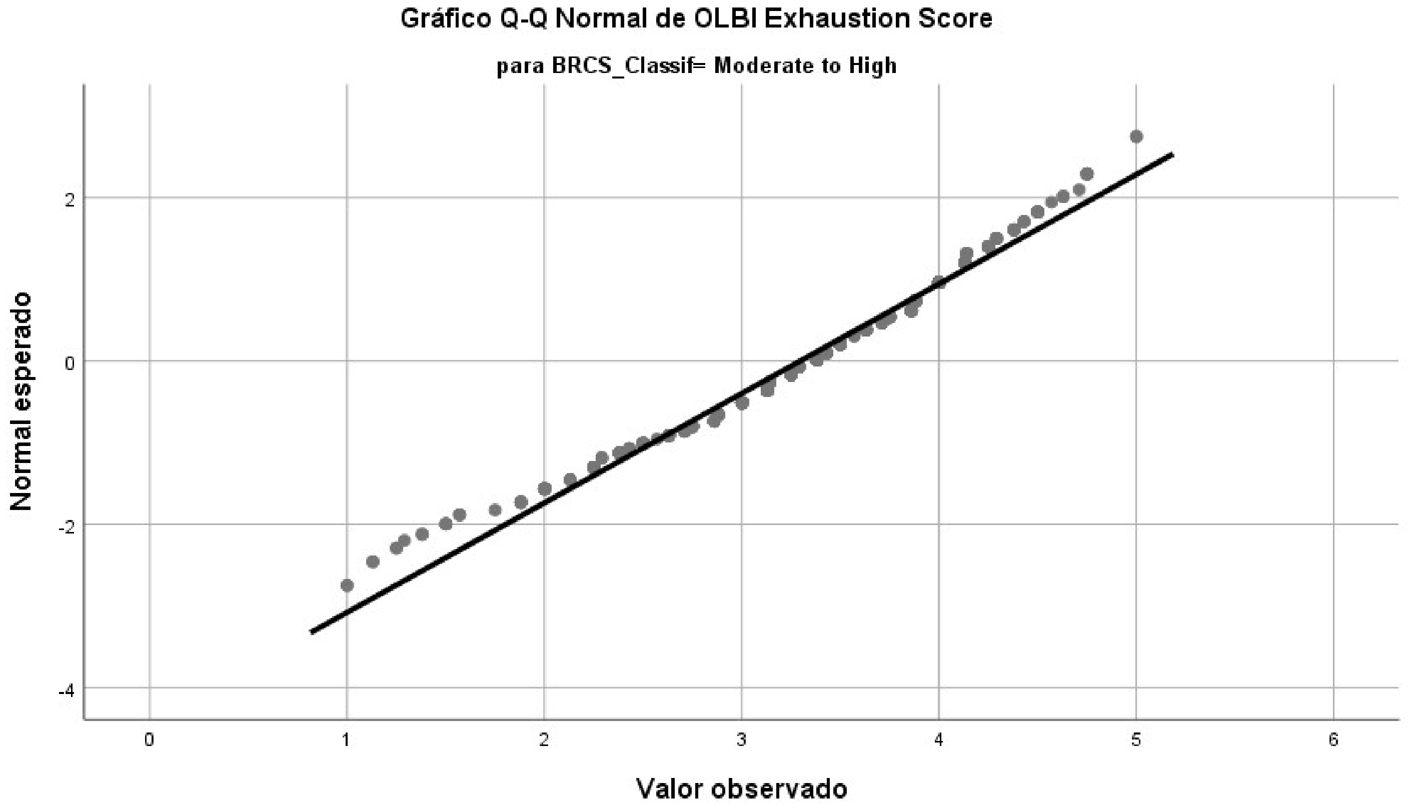

Gráfico Q-Q normais sem tendência

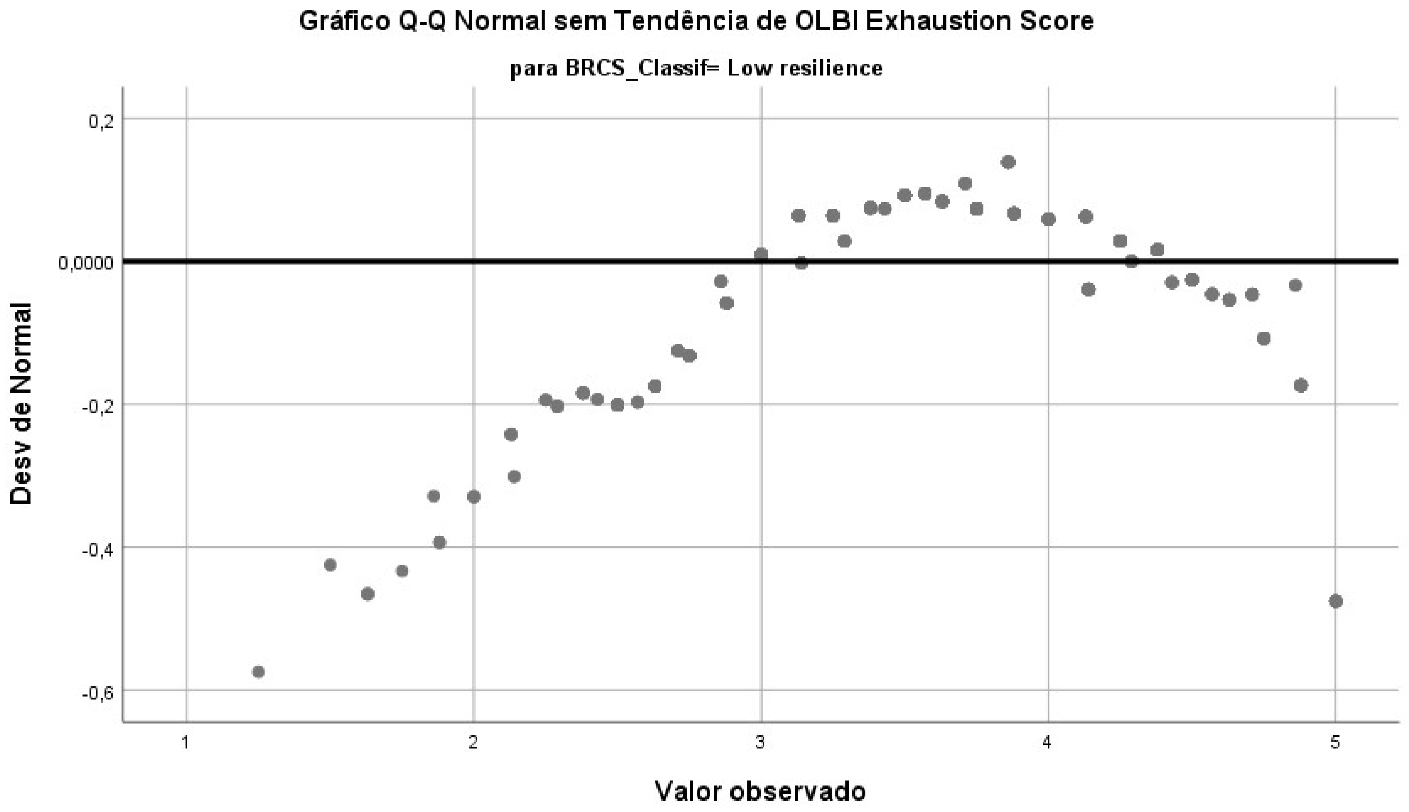

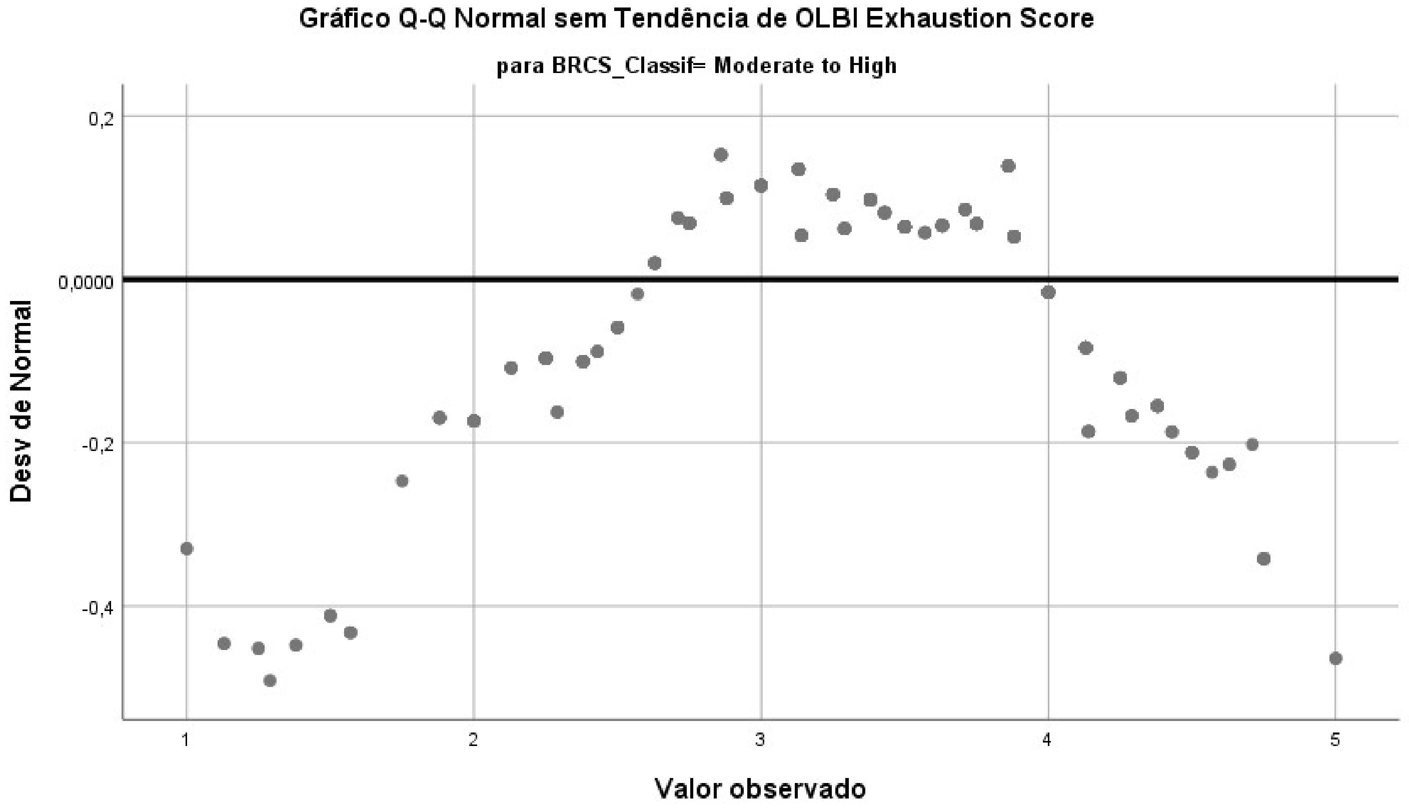

Classification of the percieved autonomy to self-conduct in the residency program

Resumo de processamento do caso

	Classification of the percieved autonomy to self-conduct in the residency program	Casos					
		Válido		Omisso		Total	
		N	Porcentagem	N	Porcentagem	N	Porcentagem
OLBI Disengagement Score	Low autonomy	224	100,0%	0	0,0%	224	100,0%
	Moderate to high autonomy	1089	100,0%	0	0,0%	1089	100,0%
OLBI Exhaustion Score	Low autonomy	224	100,0%	0	0,0%	224	100,0%
	Moderate to high autonomy	1089	100,0%	0	0,0%	1089	100,0%

Descritivos

	Classification of the percieved autonomy to self-conduct in the residency program			Estatística	Erro Erro
OLBI Disengagement Score	Low autonomy	Média		3,2695	,05654
		95% Intervalo de Confiança para Média	Limite inferior	3,1581	
			Limite superior	3,3810	
		5% da média aparada		3,2837	
		Mediana		3,2500	
		Variância		,716	
		Erro Desvio		,84620	
		Mínimo		1,00	
		Máximo		5,00	
		Intervalo		4,00	
		Amplitude interquartil		1,13	
		Assimetria		-,228	,163
		Curtose		-,298	,324
	Moderate to high autonomy	Média		2,6721	,02428
		95% Intervalo de Confiança para Média	Limite inferior	2,6244	
			Limite superior	2,7197	
		5% da média aparada		2,6589	
		Mediana		2,6250	
		Variância		,642	
		Erro Desvio		,80132	
		Mínimo		1,00	
		Máximo		5,00	
		Intervalo		4,00	
		Amplitude interquartil		1,13	
		Assimetria		,223	,074
		Curtose		-,349	,148
OLBI Exhaustion Score	Low autonomy	Média		3,8774	,04612
		95% Intervalo de Confiança para Média	Limite inferior	3,7865	
			Limite superior	3,9682	
		5% da média aparada		3,9138	
		Mediana		4,0000	
		Variância		,476	

		Erro Desvio		,69022	
		Mínimo		1,50	
		Máximo		5,00	
		Intervalo		3,50	
		Amplitude interquartil		,88	
		Assimetria		-,686	,163
		Curtose		,631	,324
	Moderate to high autonomy	Média		3,4882	,02206
		95% Intervalo de Confiança para Média	Limite inferior	3,4449	
			Limite superior	3,5315	
		5% da média aparada		3,5118	
		Mediana		3,5700	
		Variância		,530	
		Erro Desvio		,72813	
		Mínimo		1,00	
		Máximo		5,00	
		Intervalo		4,00	
		Amplitude interquartil		1,00	
		Assimetria		-,524	,074
		Curtose		,215	,148

Percentis

		Classification of the percieved autonomy to self-conduct in the residency program	Percentis 5	10	25	50	75	90	95
Média Ponderada (Definição 1)	OLBI Disengagement Score	Low autonomy	1,6250	2,1875	2,7500	3,2500	3,8750	4,3750	4,6250
		Moderate to high autonomy	1,3750	1,6250	2,1250	2,6250	3,2500	3,7500	4,0625
	OLBI Exhaustion Score	Low autonomy	2,5325	3,0000	3,5000	4,0000	4,3800	4,7500	4,8800
		Moderate to high autonomy	2,2500	2,5000	3,0000	3,5700	4,0000	4,3800	4,5700
Teste de Tukey	OLBI Disengagement Score	Low autonomy			2,7500	3,2500	3,8750		
		Moderate to high autonomy			2,1250	2,6250	3,2500		
	OLBI Exhaustion Score	Low autonomy			3,5000	4,0000	4,3800		
		Moderate to high autonomy			3,0000	3,5700	4,0000		

Testes de Normalidade

	Classification of the percieved autonomy to self-conduct in the residency program	Kolmogorov-Smirnov[a] Estatística	df	Sig.	Shapiro-Wilk Estatística	df	Sig.
OLBI Disengagement Score	Low autonomy	,047	224	,200*	,989	224	,087
	Moderate to high autonomy	,059	1089	,000	,990	1089	,000
OLBI Exhaustion Score	Low autonomy	,088	224	,000	,963	224	,000
	Moderate to high autonomy	,069	1089	,000	,980	1089	,000

*. Este é um limite inferior da significância verdadeira.

a. Correlação de Significância de Lilliefors

OLBI Disengagement Score

Histogramas

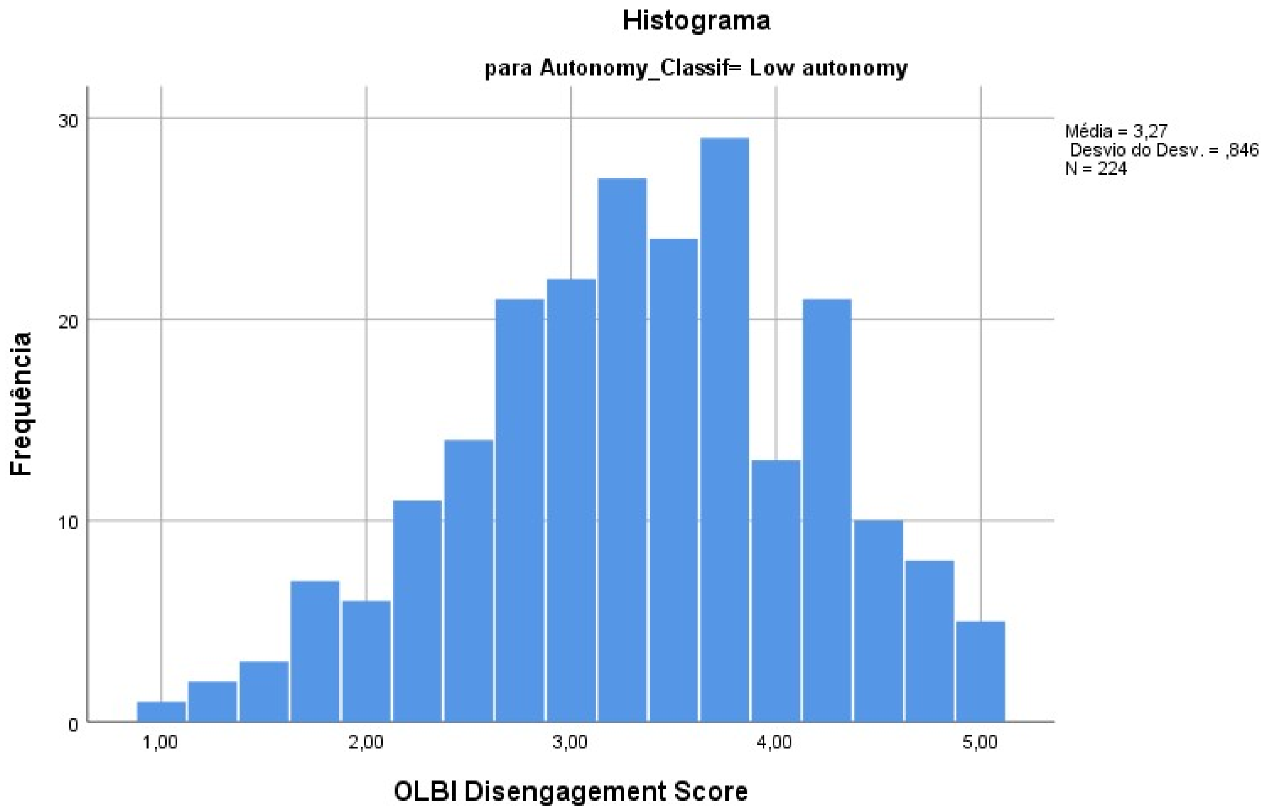

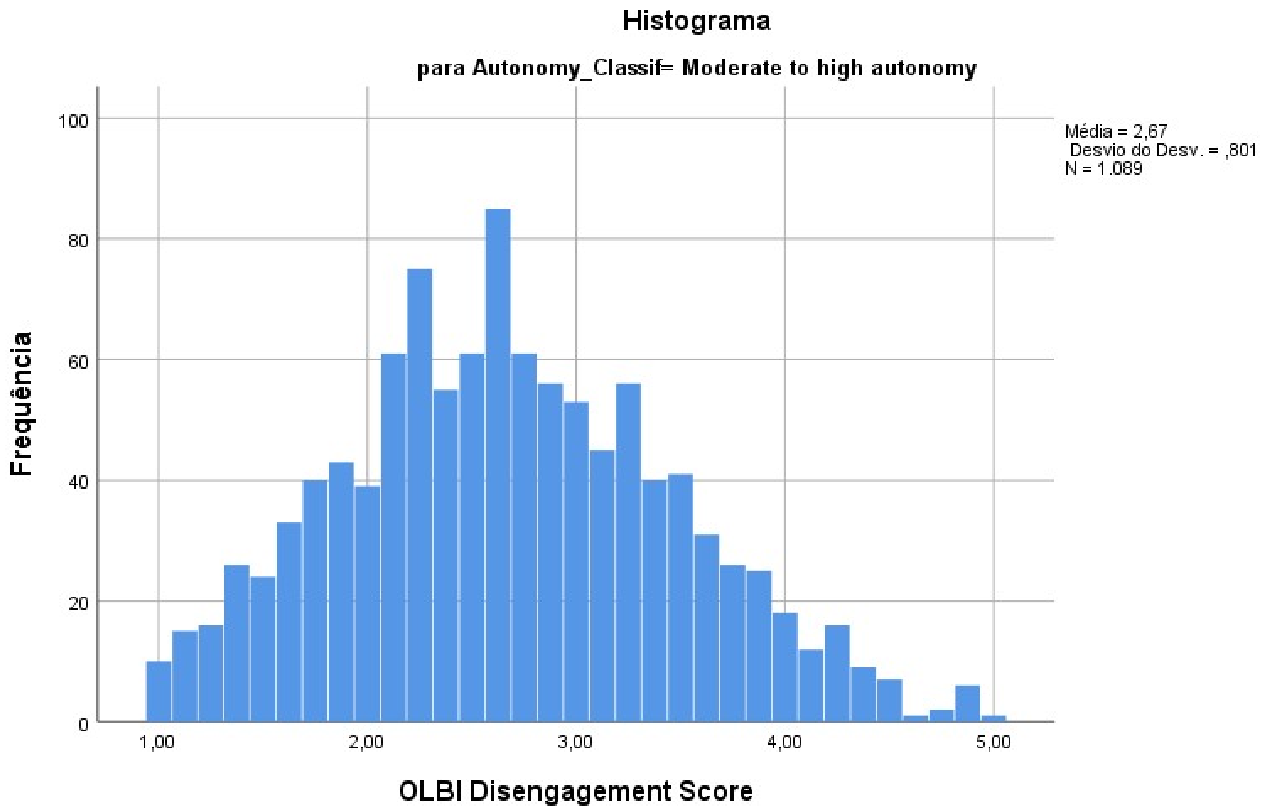

Gráfico Q-Q normais

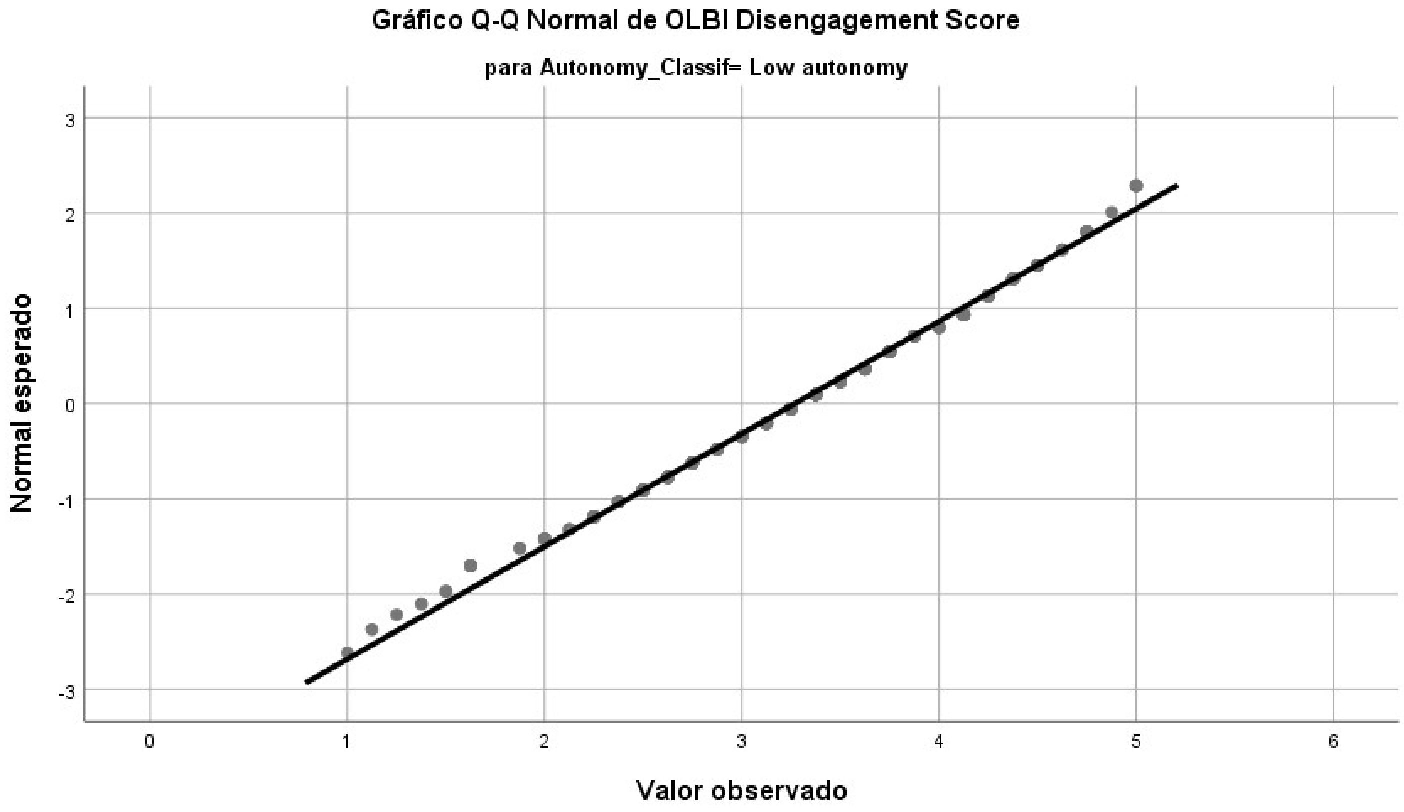

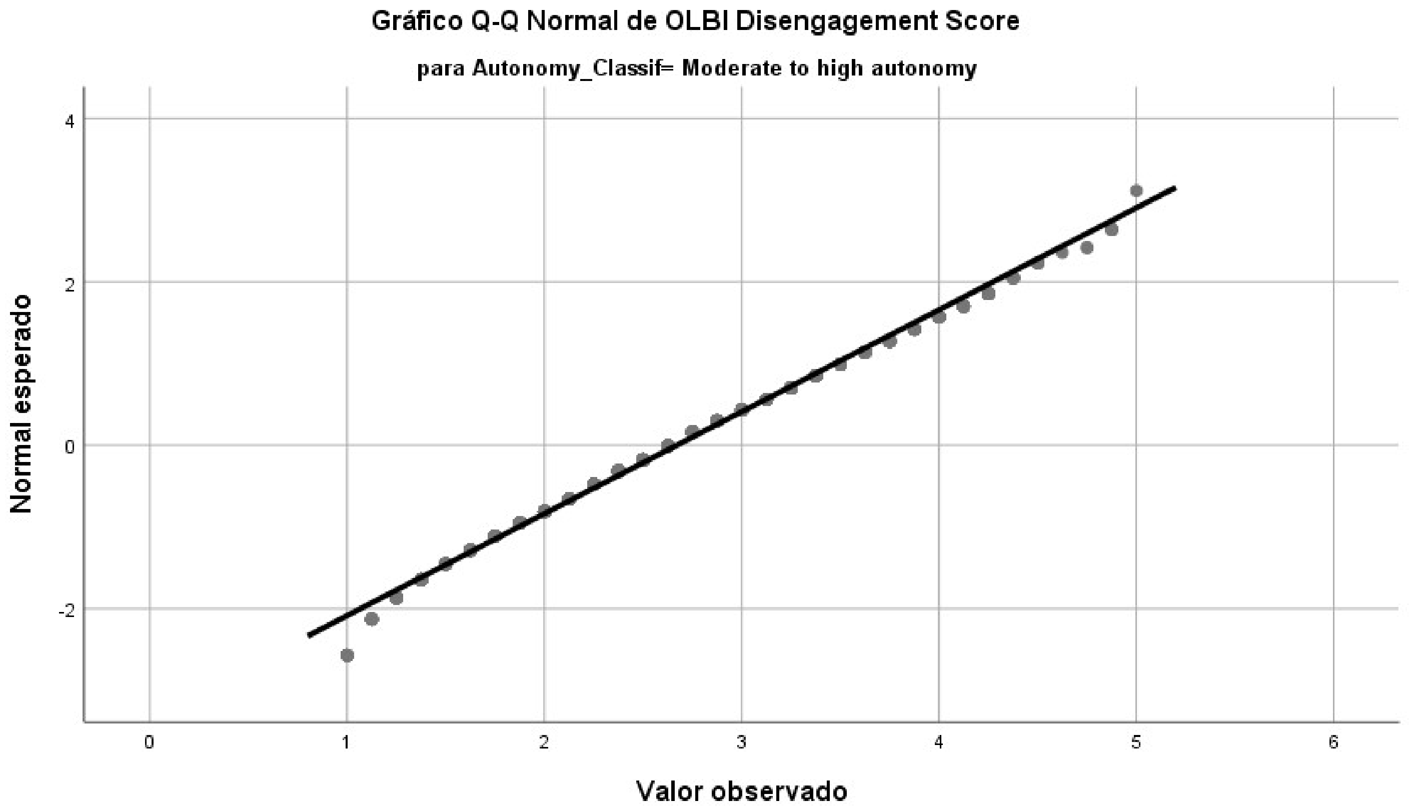

Gráfico Q-Q normais sem tendência

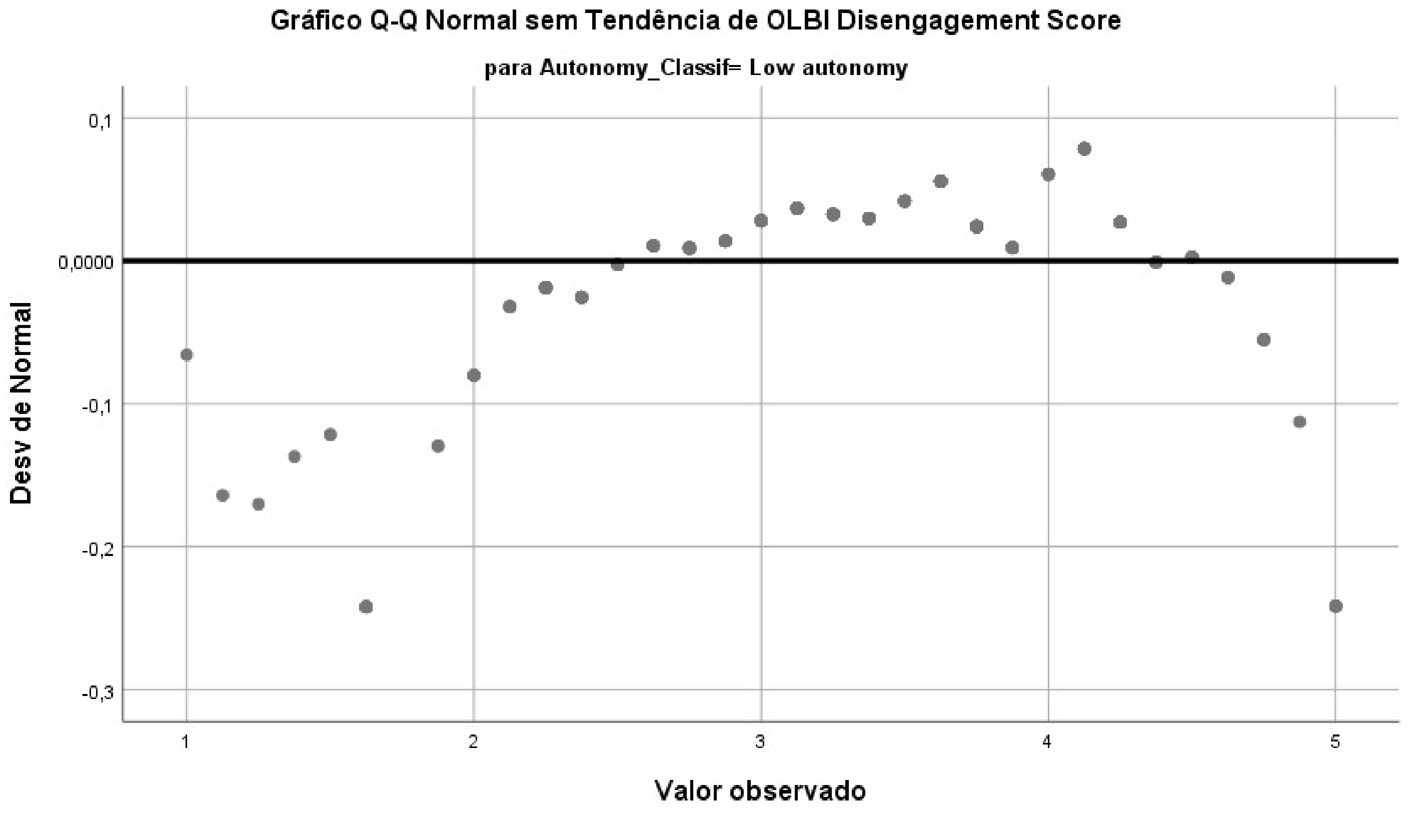

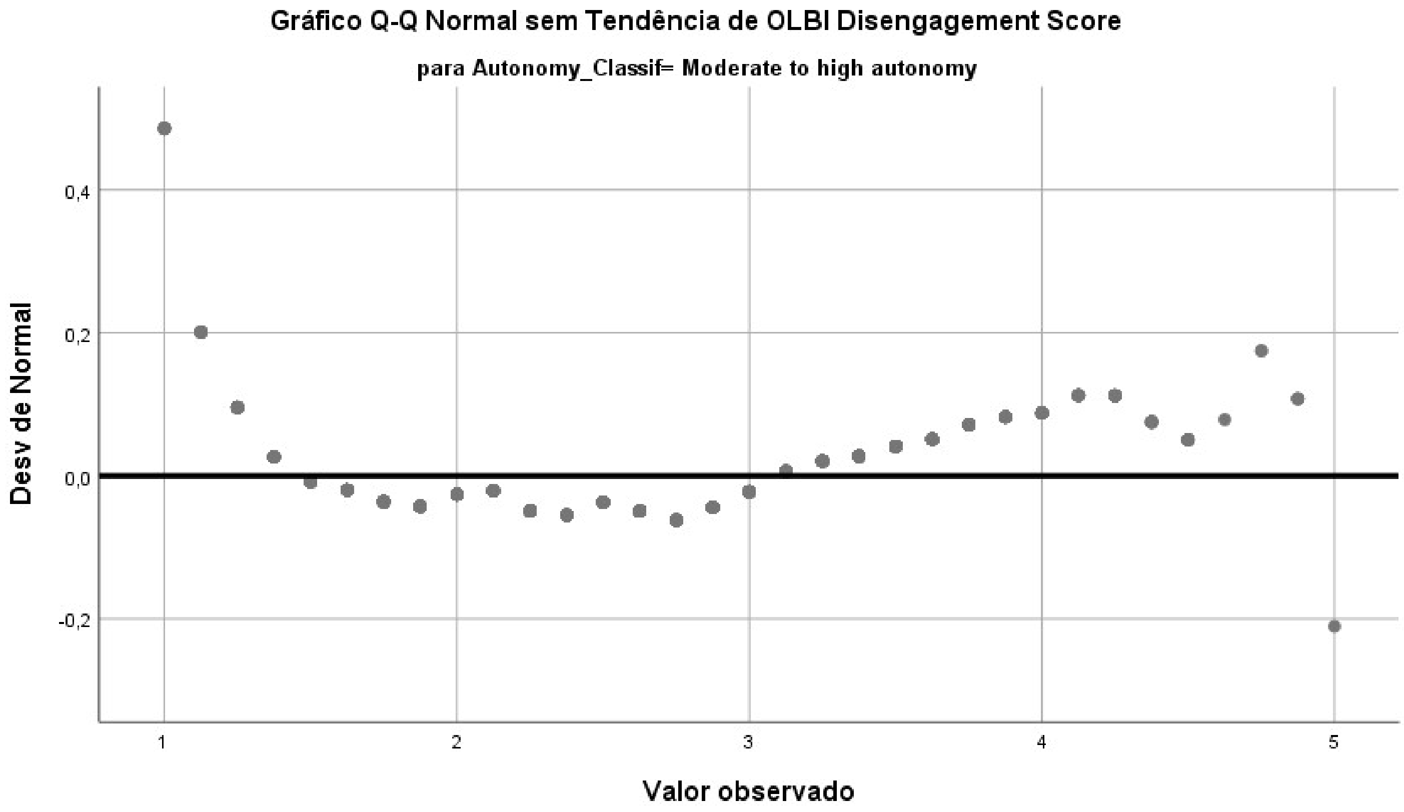

OLBI Exhaustion Score

Histogramas

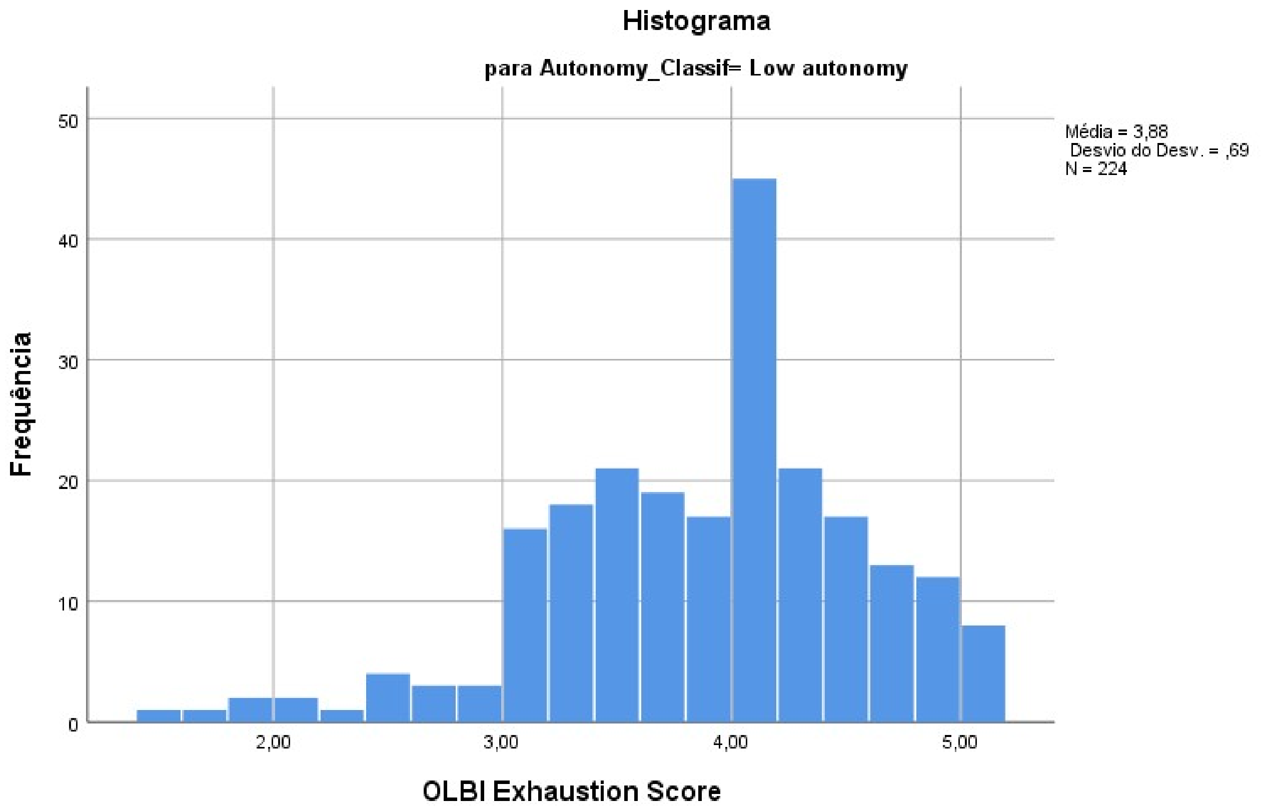

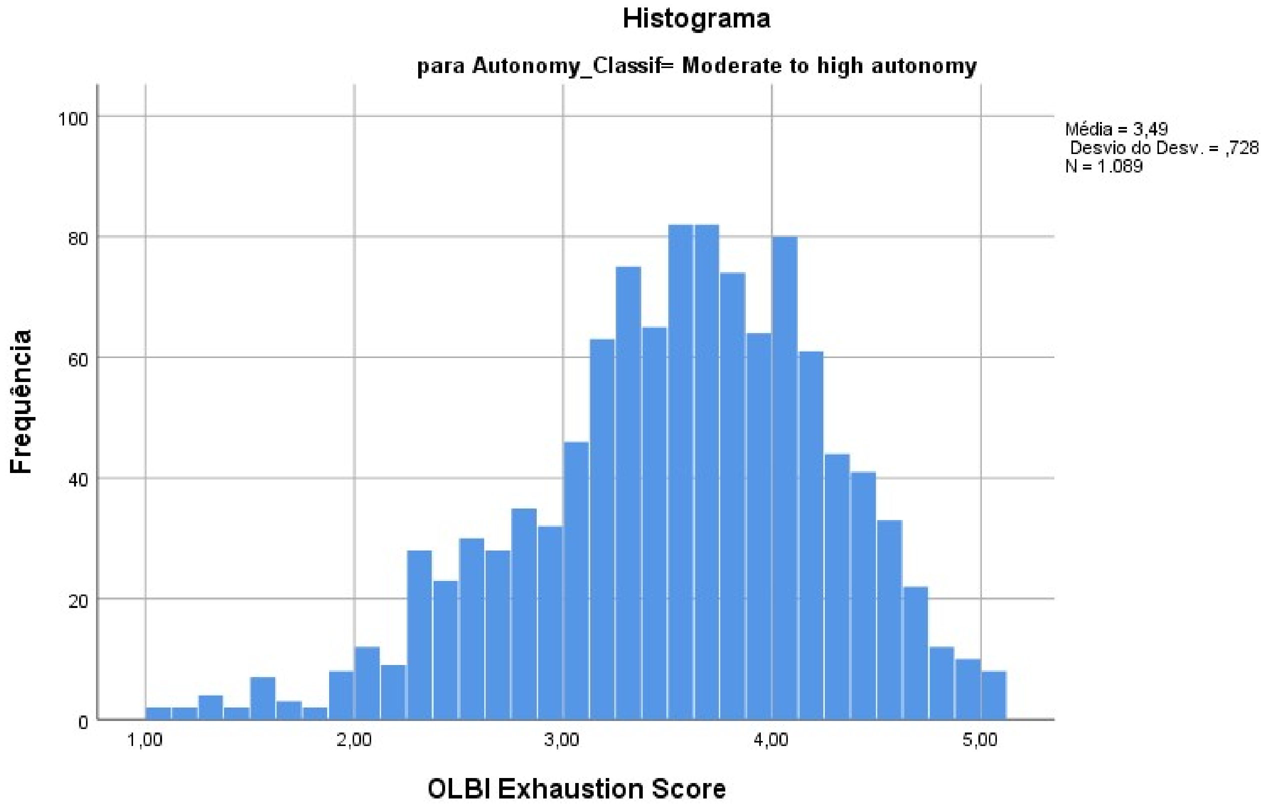

Gráfico Q-Q normais

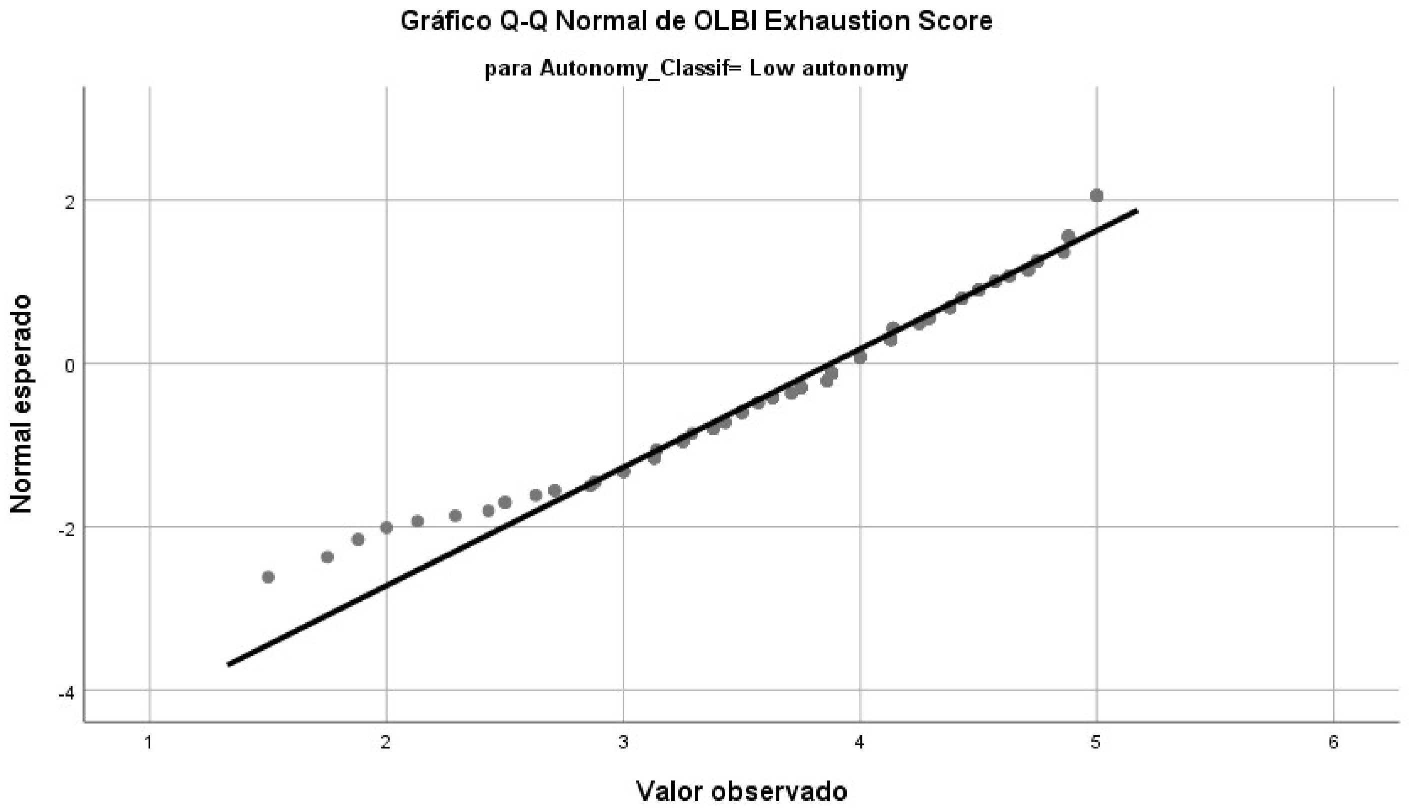

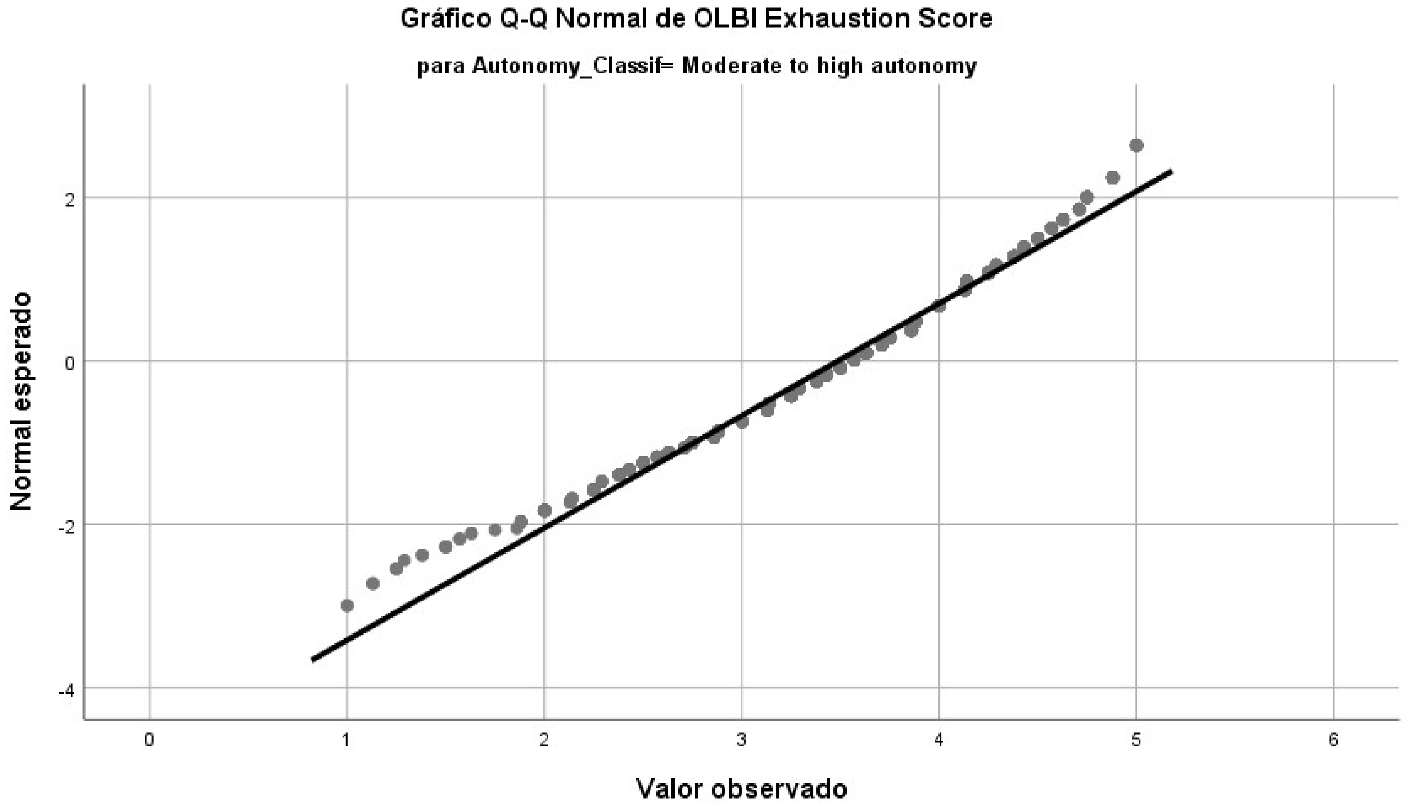

Gráfico Q-Q normais sem tendência

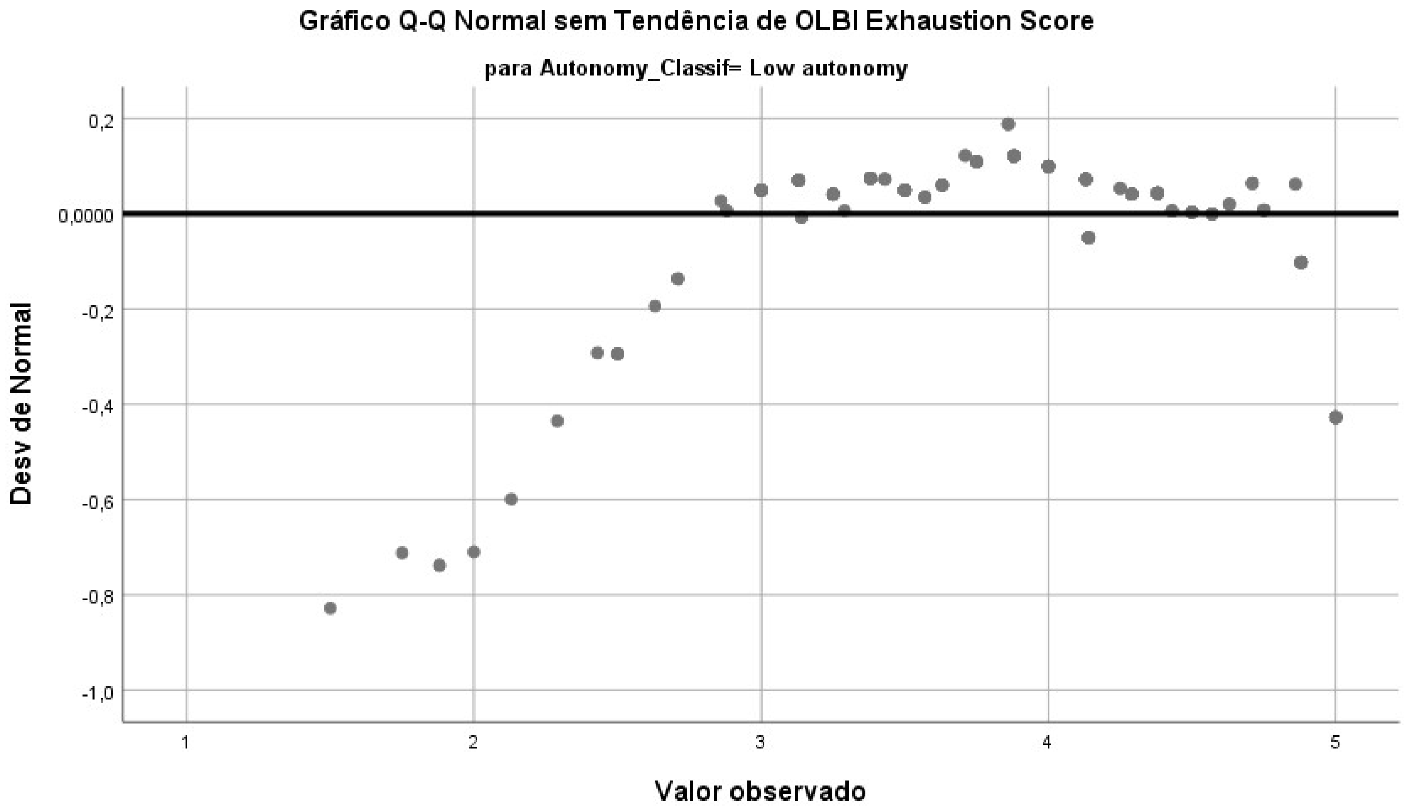

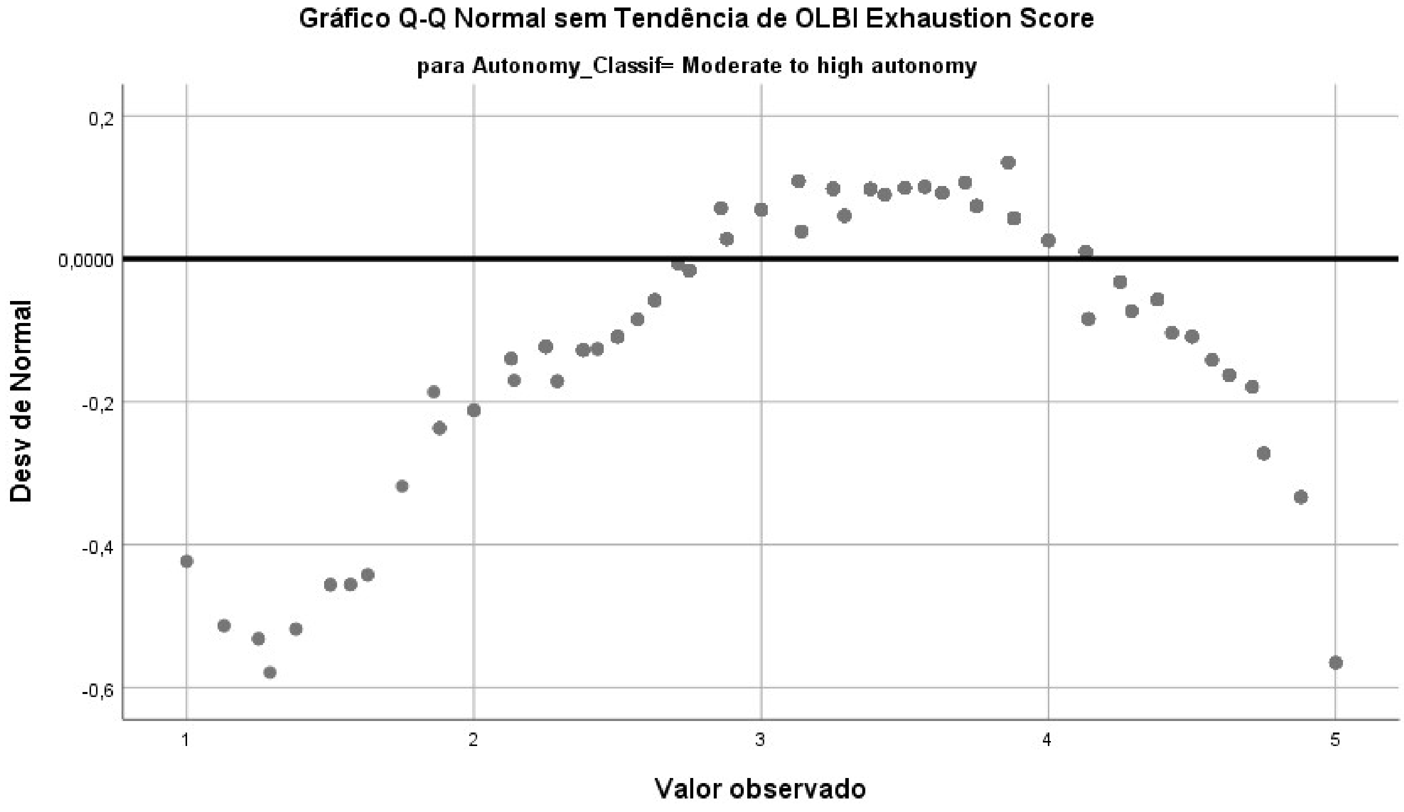

Percieved adequacy of the pedagogic structure and availability of resouces in the residency program

Resumo de processamento do caso

	Percieved adequacy of the pedagogic structure and availability of resouces in the residency program	Casos - Válido N	Válido Porcentagem	Omisso N	Omisso Porcentagem	Total N	Total Porcentagem
OLBI Disengagement Score	Poor adequacy	558	100,0%	0	0,0%	558	100,0%
	Moderate to good adequacy	755	100,0%	0	0,0%	755	100,0%
OLBI Exhaustion Score	Poor adequacy	558	100,0%	0	0,0%	558	100,0%
	Moderate to good adequacy	755	100,0%	0	0,0%	755	100,0%

Descritivos

	Percieved adequacy of the pedagogic structure and availability of resouces in the residency program			Estatística	Erro Erro
OLBI Disengagement Score	Poor adequacy	Média		3,1254	,03341
		95% Intervalo de Confiança para Média	Limite inferior	3,0598	
			Limite superior	3,1911	
		5% da média aparada		3,1250	
		Mediana		3,1250	
		Variância		,623	
		Erro Desvio		,78926	
		Mínimo		1,13	
		Máximo		5,00	
		Intervalo		3,88	
		Amplitude interquartil		1,00	
		Assimetria		-,028	,103
		Curtose		-,263	,206
	Moderate to good adequacy	Média		2,5142	,02836
		95% Intervalo de Confiança para Média	Limite inferior	2,4586	
			Limite superior	2,5699	
		5% da média aparada		2,4931	
		Mediana		2,5000	
		Variância		,607	
		Erro Desvio		,77919	
		Mínimo		1,00	
		Máximo		4,88	
		Intervalo		3,88	
		Amplitude interquartil		1,00	
		Assimetria		,370	,089
		Curtose		-,193	,178
OLBI Exhaustion Score	Poor adequacy	Média		3,8236	,02794
		95% Intervalo de Confiança para Média	Limite inferior	3,7687	
			Limite superior	3,8784	
		5% da média aparada		3,8512	
		Mediana		3,8800	

		Variância		,435	
		Erro Desvio		,65989	
		Mínimo		1,57	
		Máximo		5,00	
		Intervalo		3,43	
		Amplitude interquartil		,86	
		Assimetria		-,565	,103
		Curtose		,292	,206
	Moderate to good adequacy	Média		3,3558	,02646
		95% Intervalo de Confiança para Média	Limite inferior	3,3039	
			Limite superior	3,4078	
		5% da média aparada		3,3799	
		Mediana		3,3800	
		Variância		,528	
		Erro Desvio		,72694	
		Mínimo		1,00	
		Máximo		5,00	
		Intervalo		4,00	
		Amplitude interquartil		1,00	
		Assimetria		-,511	,089
		Curtose		,193	,178

Percentis

		Percieved adequacy of the pedagogic structure and availability of resouces in the residency program	Percentis						
			5	10	25	50	75	90	95
Média Ponderada (Definição 1)	OLBI Disengagement Score	Poor adequacy	1,7500	2,1125	2,6250	3,1250	3,6250	4,1250	4,5000
		Moderate to good adequacy	1,2500	1,5000	2,0000	2,5000	3,0000	3,6250	4,0000
	OLBI Exhaustion Score	Poor adequacy	2,5665	3,0000	3,4300	3,8800	4,2900	4,7100	4,8800
		Moderate to good adequacy	2,0000	2,3800	2,8800	3,3800	3,8800	4,2500	4,3900
Teste de Tukey	OLBI Disengagement Score	Poor adequacy			2,6250	3,1250	3,6250		
		Moderate to good adequacy			2,0000	2,5000	3,0000		
	OLBI Exhaustion Score	Poor adequacy			3,4300	3,8800	4,2900		
		Moderate to good adequacy			2,8800	3,3800	3,8800		

Testes de Normalidade

	Percieved adequacy of the pedagogic structure and availability of resouces in the residency program	Kolmogorov-Smirnov[a]			Shapiro-Wilk		
		Estatística	df	Sig.	Estatística	df	Sig.
OLBI Disengagement Score	Poor adequacy	,041	558	,024	,994	558	,019
	Moderate to good adequacy	,069	755	,000	,984	755	,000

OLBI Exhaustion Score	Poor adequacy	,073	558	,000	,974	558	,000
	Moderate to good adequacy	,070	755	,000	,981	755	,000

a. Correlação de Significância de Lilliefors

OLBI Disengagement Score

Histogramas

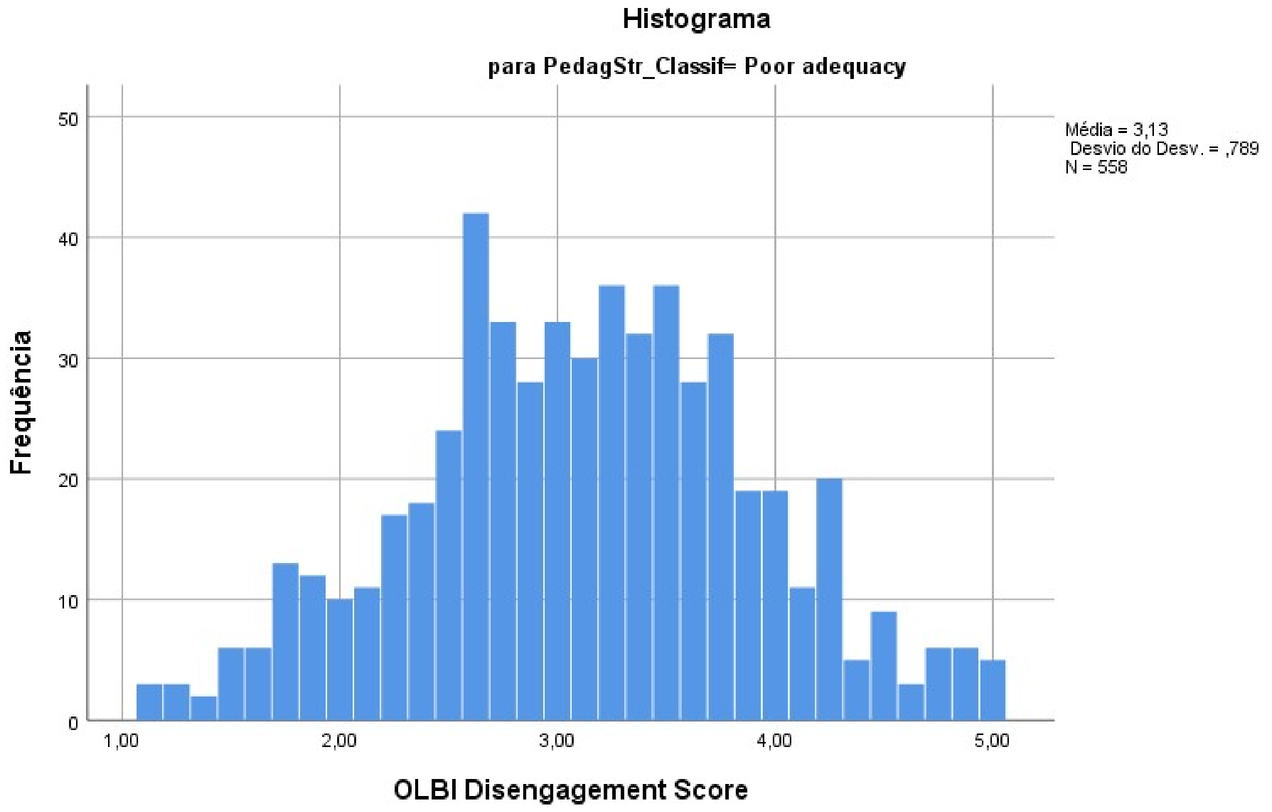

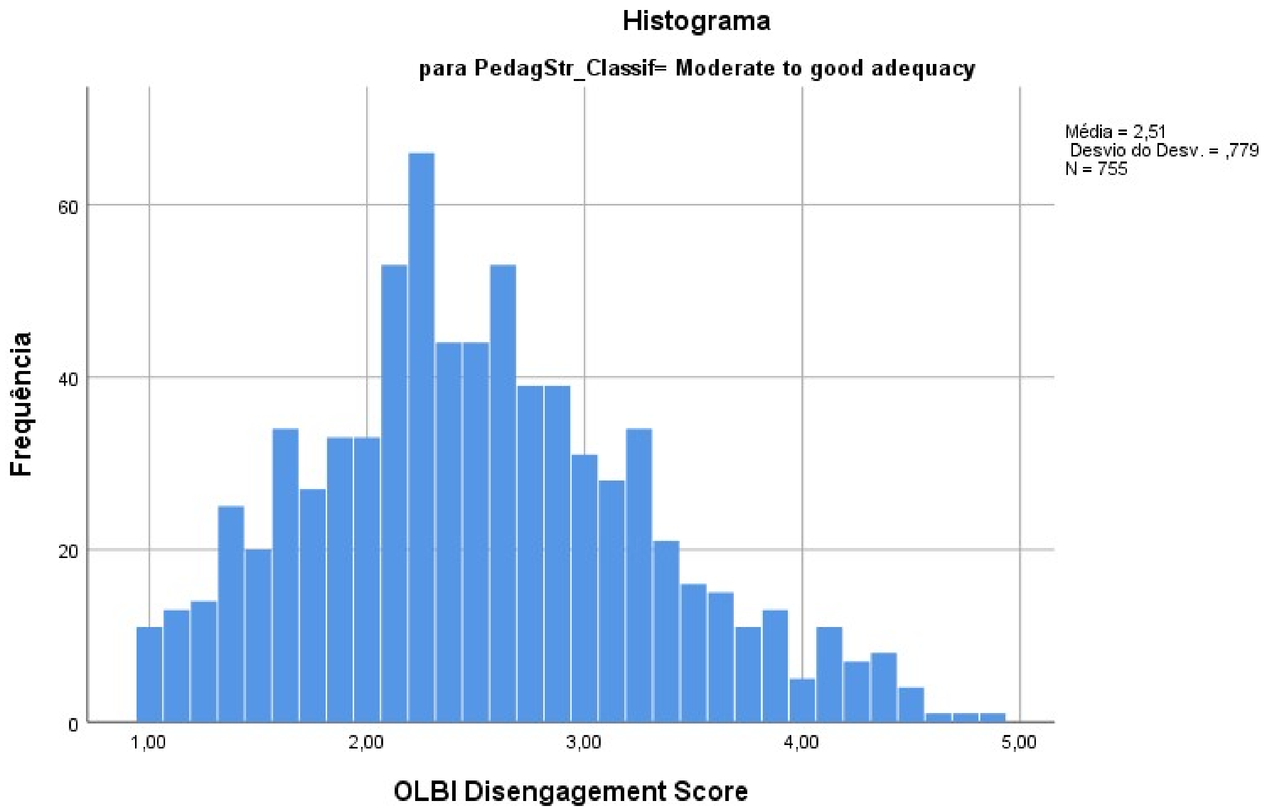

Gráfico Q-Q normais

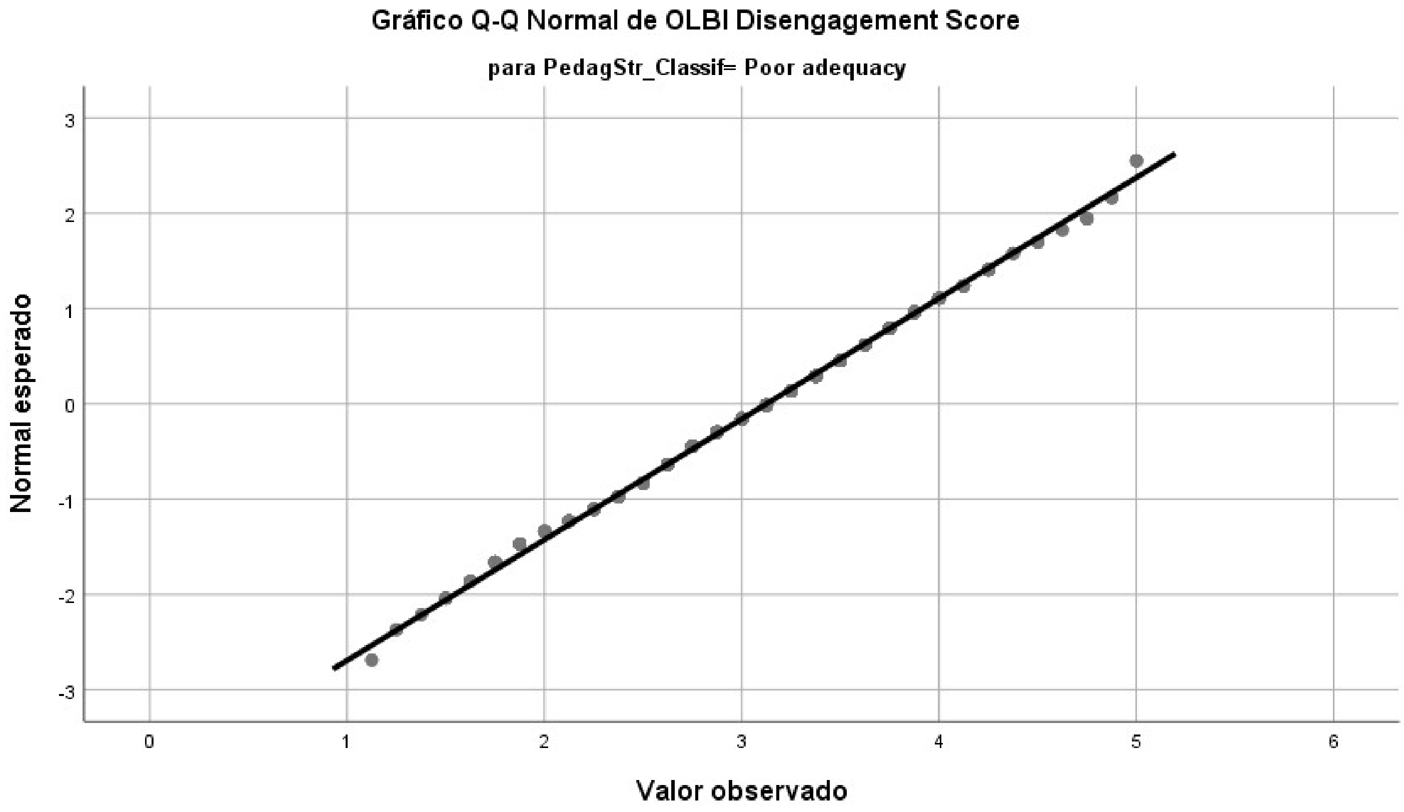

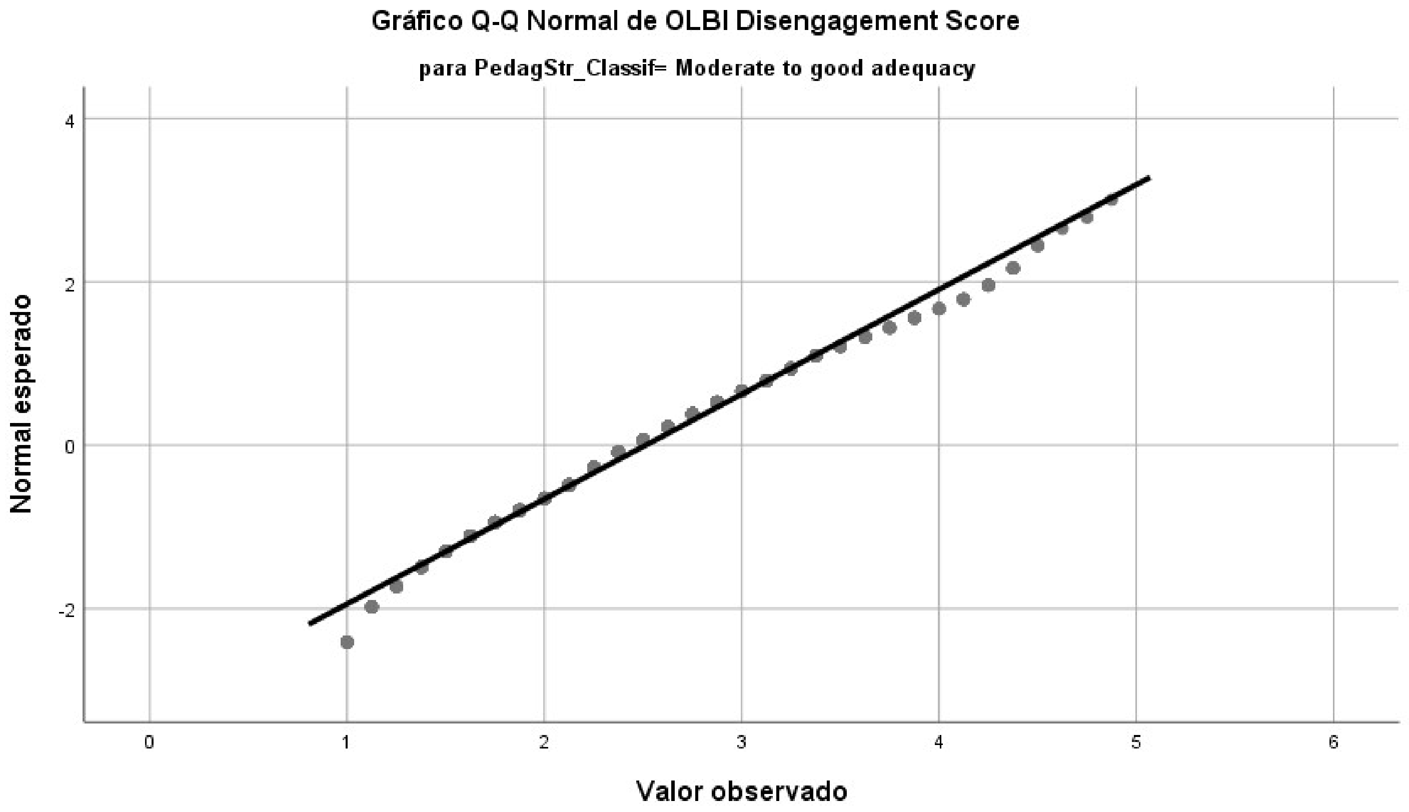

Gráfico Q-Q normais sem tendência

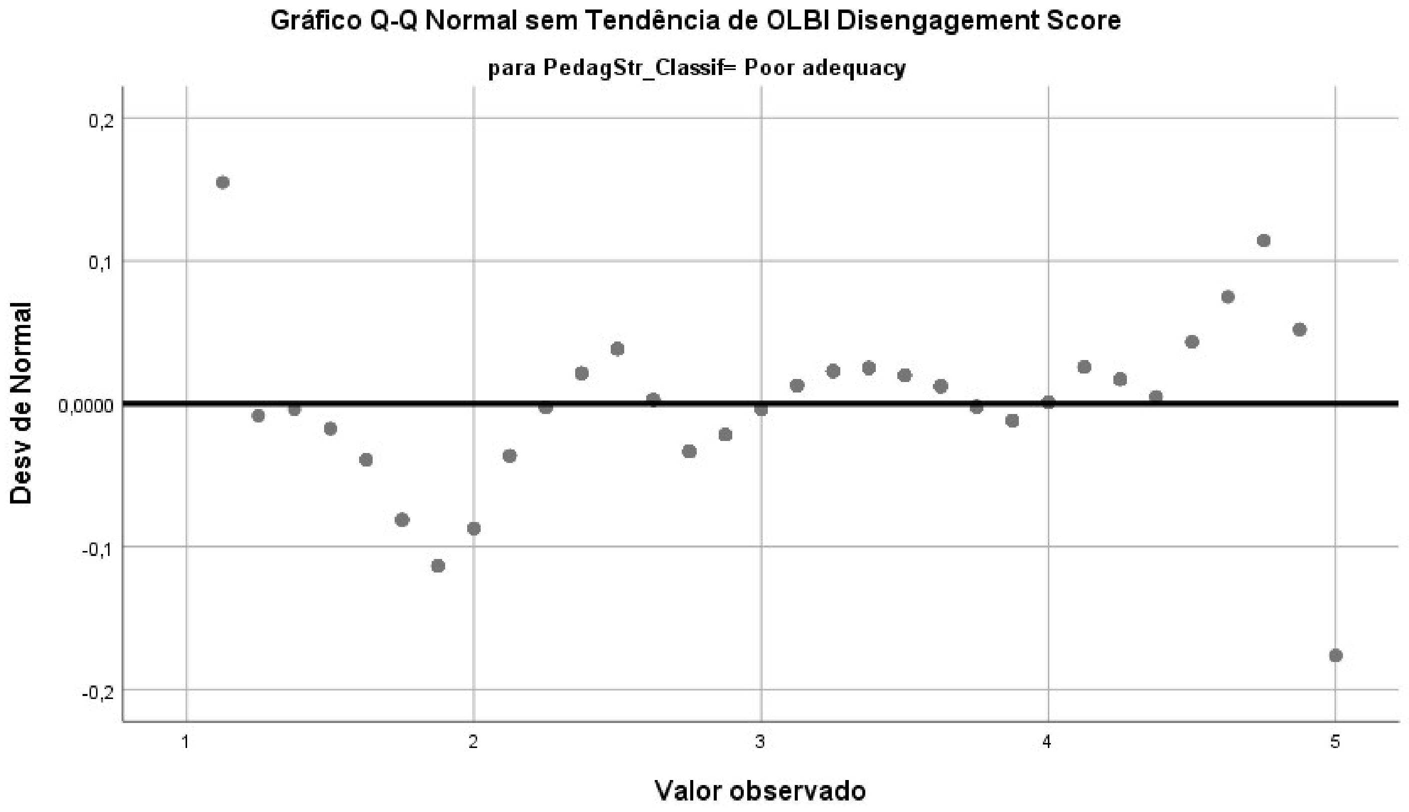

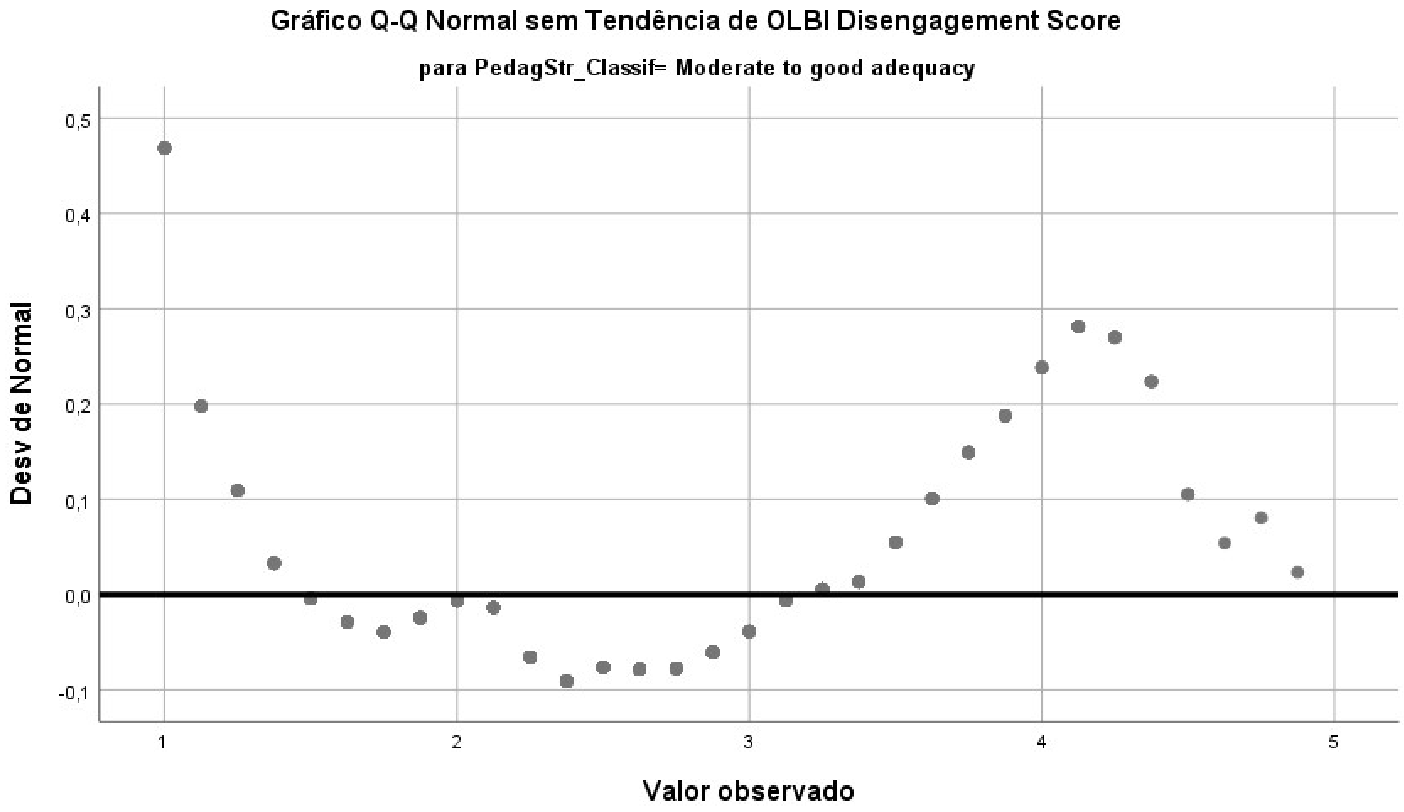

OLBI Exhaustion Score

Histogramas

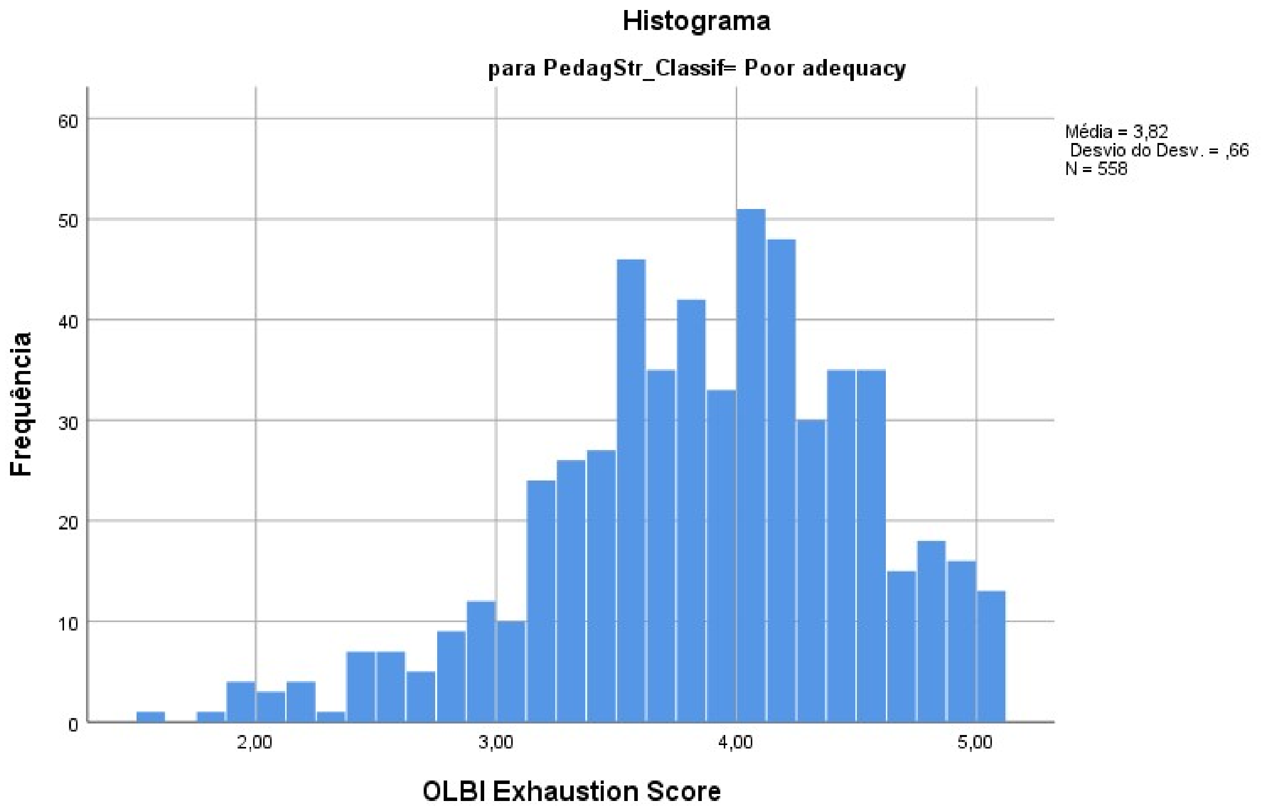

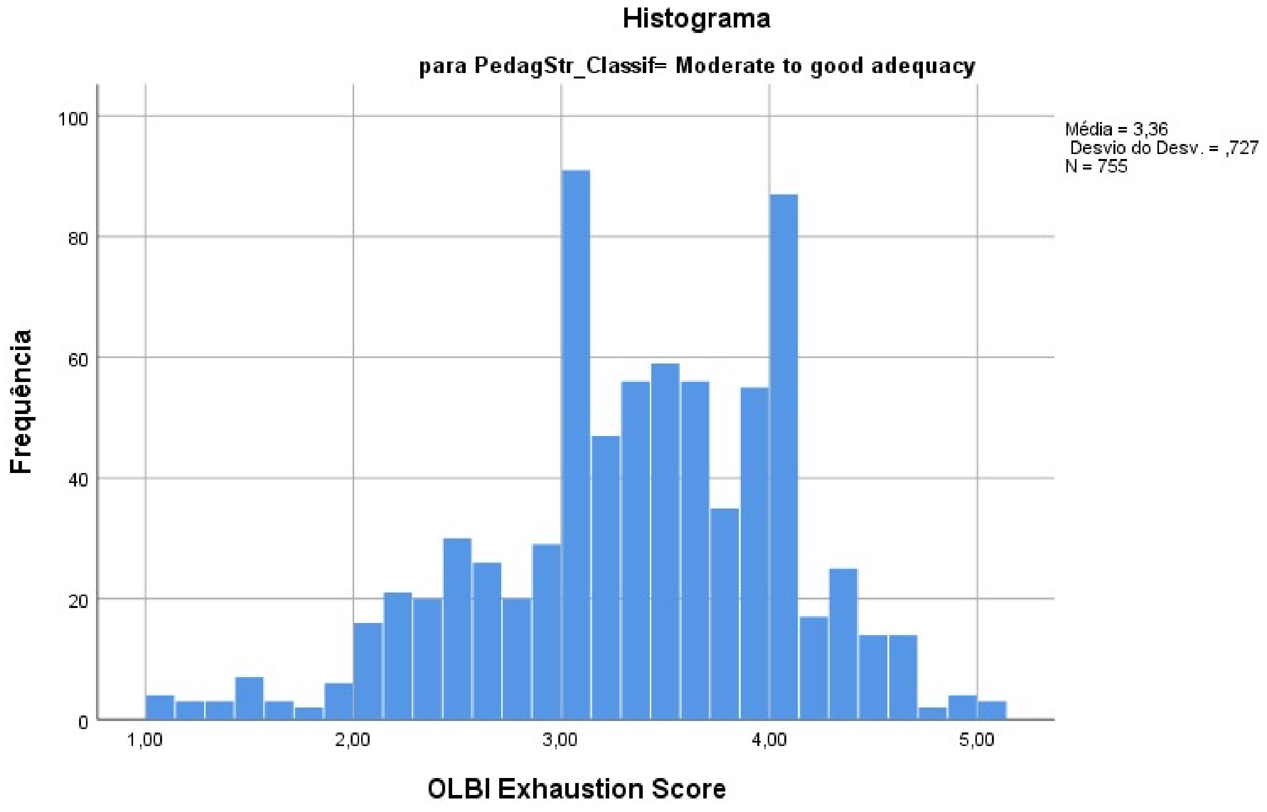

Gráfico Q-Q normais

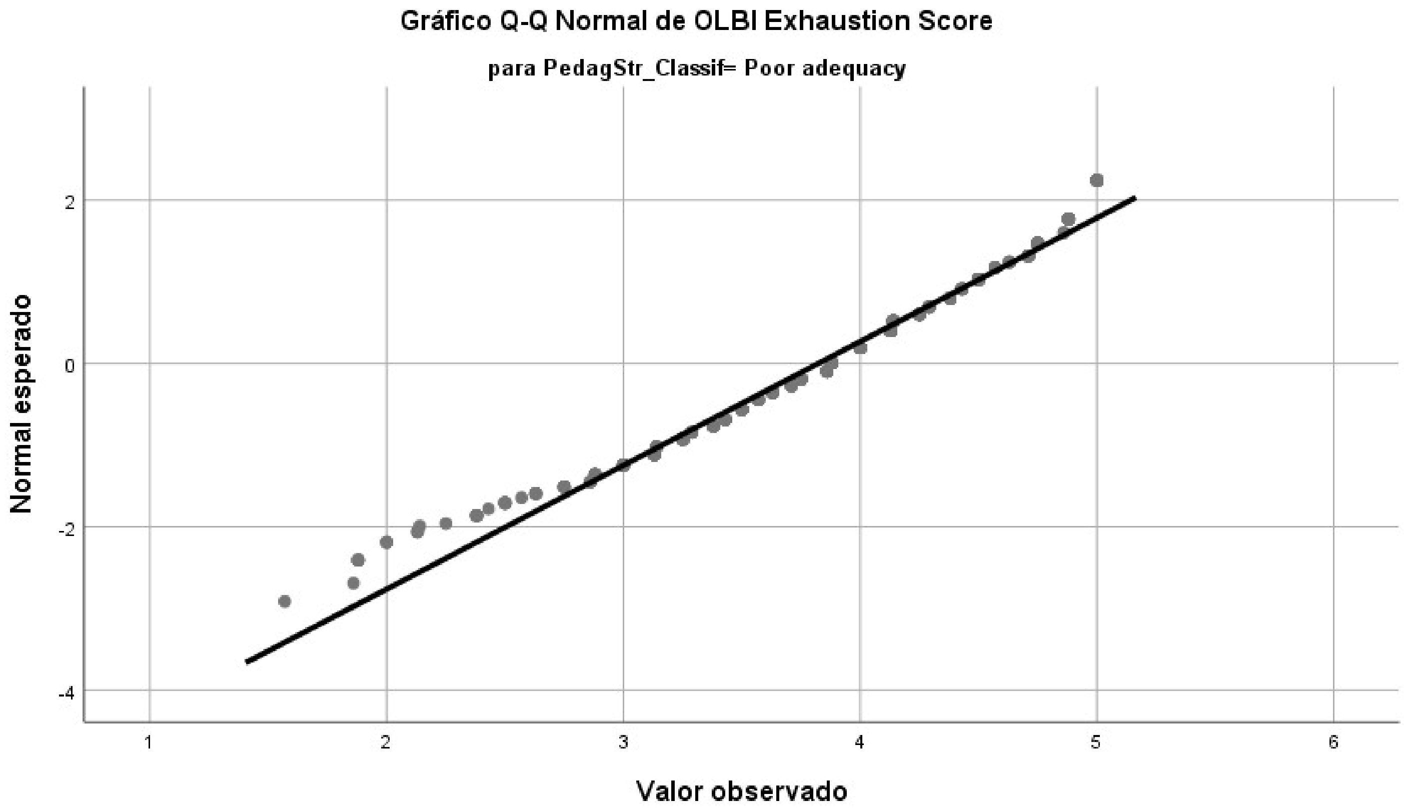

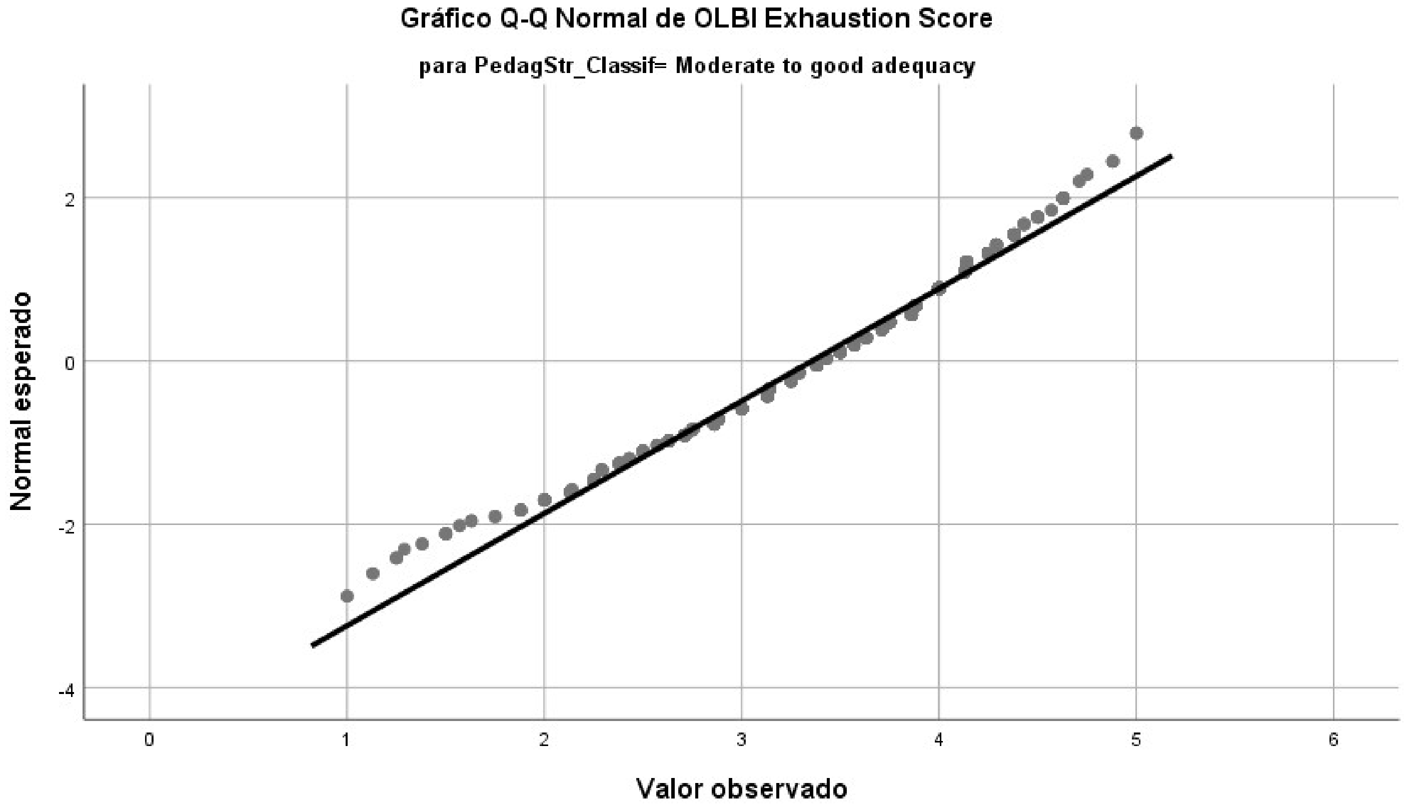

Gráfico Q-Q normais sem tendência

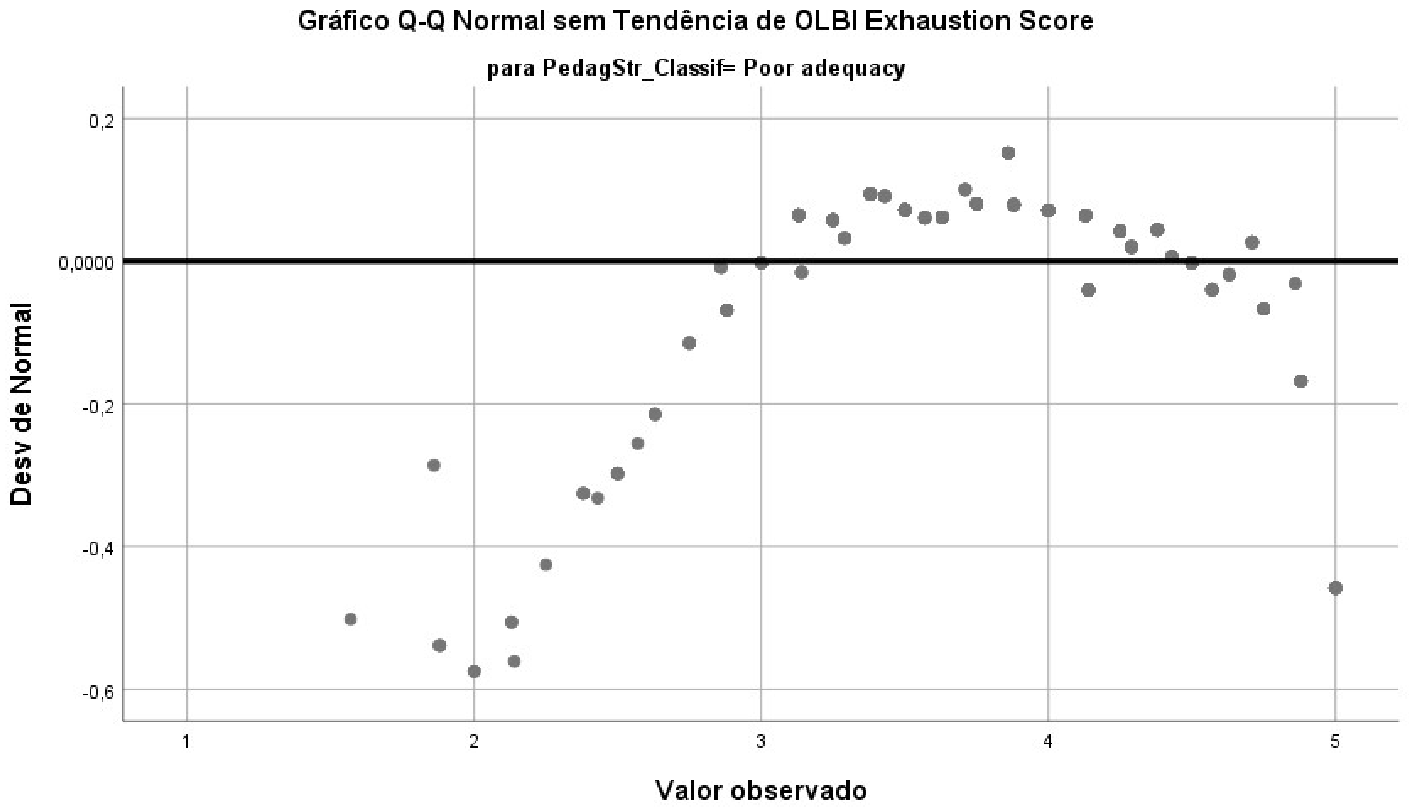

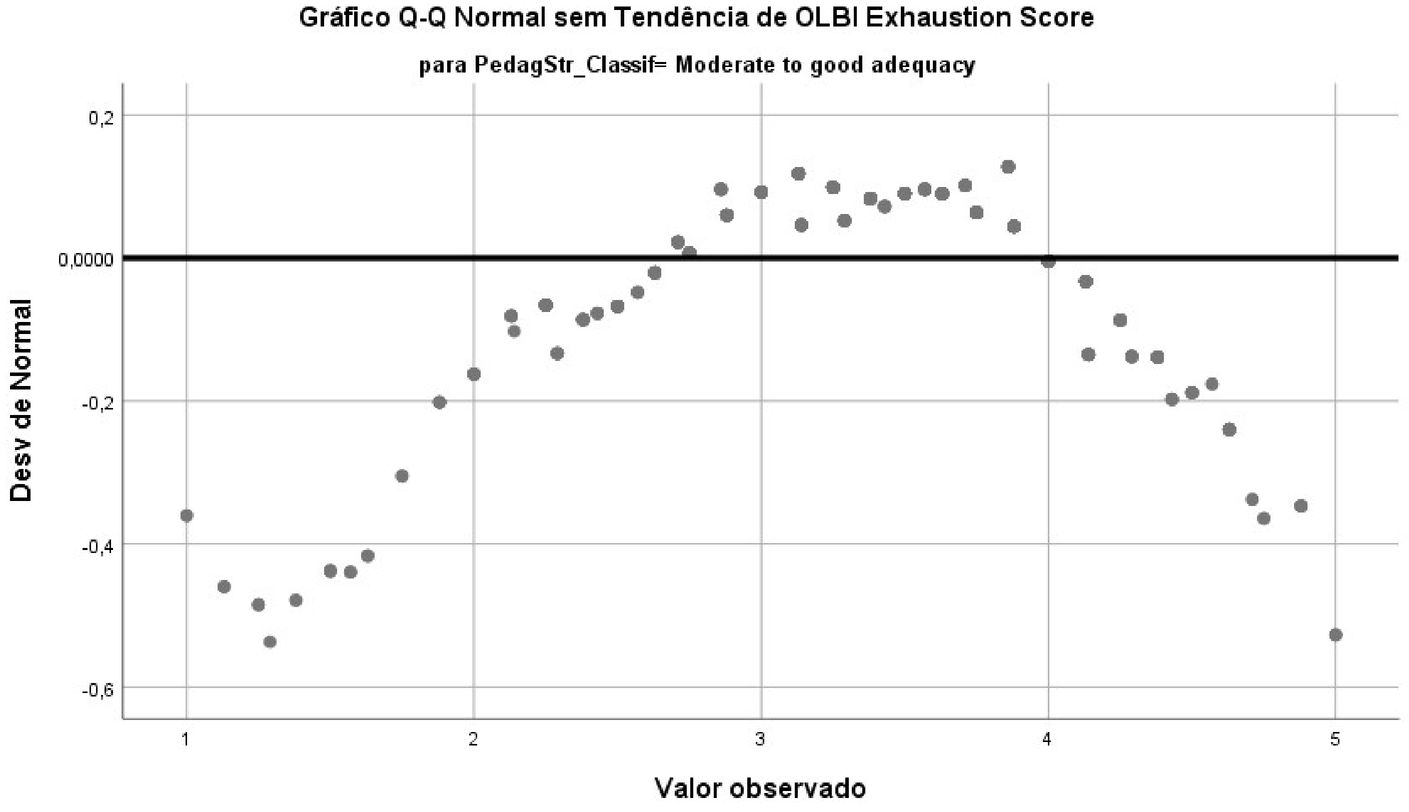

Perceived adequacy of the availability of personal protective equipment, when providing care for patients in the residency program

Resumo de processamento do caso

	Perceived adequacy of the availability of personal protective equipment, when providing care for patients in the residency program	Casos					
		Válido		Omisso		Total	
		N	Porcentagem	N	Porcentagem	N	Porcentagem
OLBI Disengagement Score	Poor adequacy	281	100,0%	0	0,0%	281	100,0%
	Good adequacy	1032	100,0%	0	0,0%	1032	100,0%
OLBI Exhaustion Score	Poor adequacy	281	100,0%	0	0,0%	281	100,0%
	Good adequacy	1032	100,0%	0	0,0%	1032	100,0%

Descritivos

	Perceived adequacy of the availability of personal protective equipment, when providing care for patients in the residency program			Estatística	Erro Erro
OLBI Disengagement Score	Poor adequacy	Média		3,0810	,05123
		95% Intervalo de Confiança para Média	Limite inferior	2,9801	
			Limite superior	3,1818	
		5% da média aparada		3,0865	
		Mediana		3,1250	
		Variância		,737	
		Erro Desvio		,85872	
		Mínimo		1,00	
		Máximo		5,00	
		Intervalo		4,00	
		Amplitude interquartil		1,25	
		Assimetria		-,078	,145
		Curtose		-,512	,290
	Good adequacy	Média		2,6904	,02536
		95% Intervalo de Confiança para Média	Limite inferior	2,6406	
			Limite superior	2,7402	
		5% da média aparada		2,6761	
		Mediana		2,6250	
		Variância		,664	
		Erro Desvio		,81479	
		Mínimo		1,00	
		Máximo		5,00	
		Intervalo		4,00	
		Amplitude interquartil		1,13	
		Assimetria		,237	,076
		Curtose		-,330	,152
OLBI Exhaustion Score	Poor adequacy	Média		3,7724	,04089
		95% Intervalo de Confiança para Média	Limite inferior	3,6919	
			Limite superior	3,8529	

		5% da média aparada		3,8000	
		Mediana		3,8800	
		Variância		,470	
		Erro Desvio		,68541	
		Mínimo		1,00	
		Máximo		5,00	
		Intervalo		4,00	
		Amplitude interquartil		,98	
		Assimetria		-,638	,145
		Curtose		,432	,290
	Good adequacy	Média		3,4953	,02300
		95% Intervalo de Confiança para Média	Limite inferior	3,4502	
			Limite superior	3,5404	
		5% da média aparada		3,5180	
		Mediana		3,5700	
		Variância		,546	
		Erro Desvio		,73877	
		Mínimo		1,00	
		Máximo		5,00	
		Intervalo		4,00	
		Amplitude interquartil		,87	
		Assimetria		-,496	,076
		Curtose		,203	,152

Percentis

		Perceived adequacy of the availability of personal protective equipment, when providing care for patients in the residency program	Percentis 5	10	25	50	75	90	95
Média Ponderada (Definição 1)	OLBI Disengagement Score	Poor adequacy	1,6250	2,0000	2,5000	3,1250	3,7500	4,2500	4,5000
		Good adequacy	1,3750	1,6250	2,1250	2,6250	3,2500	3,7500	4,1250
	OLBI Exhaustion Score	Poor adequacy	2,5130	2,8800	3,2900	3,8800	4,2700	4,5560	4,7500
		Good adequacy	2,2115	2,5000	3,1300	3,5700	4,0000	4,3800	4,6300
Teste de Tukey	OLBI Disengagement Score	Poor adequacy			2,5000	3,1250	3,7500		
		Good adequacy			2,1250	2,6250	3,2500		
	OLBI Exhaustion Score	Poor adequacy			3,2900	3,8800	4,2500		
		Good adequacy			3,1300	3,5700	4,0000		

Testes de Normalidade

	Perceived adequacy of the	Kolmogorov-Smirnov[a]	Shapiro-Wilk

	availability of personal protective equipment, when providing care for patients in the residency program	Estatística	df	Sig.	Estatística	df	Sig.
OLBI Disengagement Score	Poor adequacy	,053	281	,059	,990	281	,060
	Good adequacy	,062	1032	,000	,990	1032	,000
OLBI Exhaustion Score	Poor adequacy	,106	281	,000	,970	281	,000
	Good adequacy	,070	1032	,000	,981	1032	,000

a. Correlação de Significância de Lilliefors

OLBI Disengagement Score

Histogramas

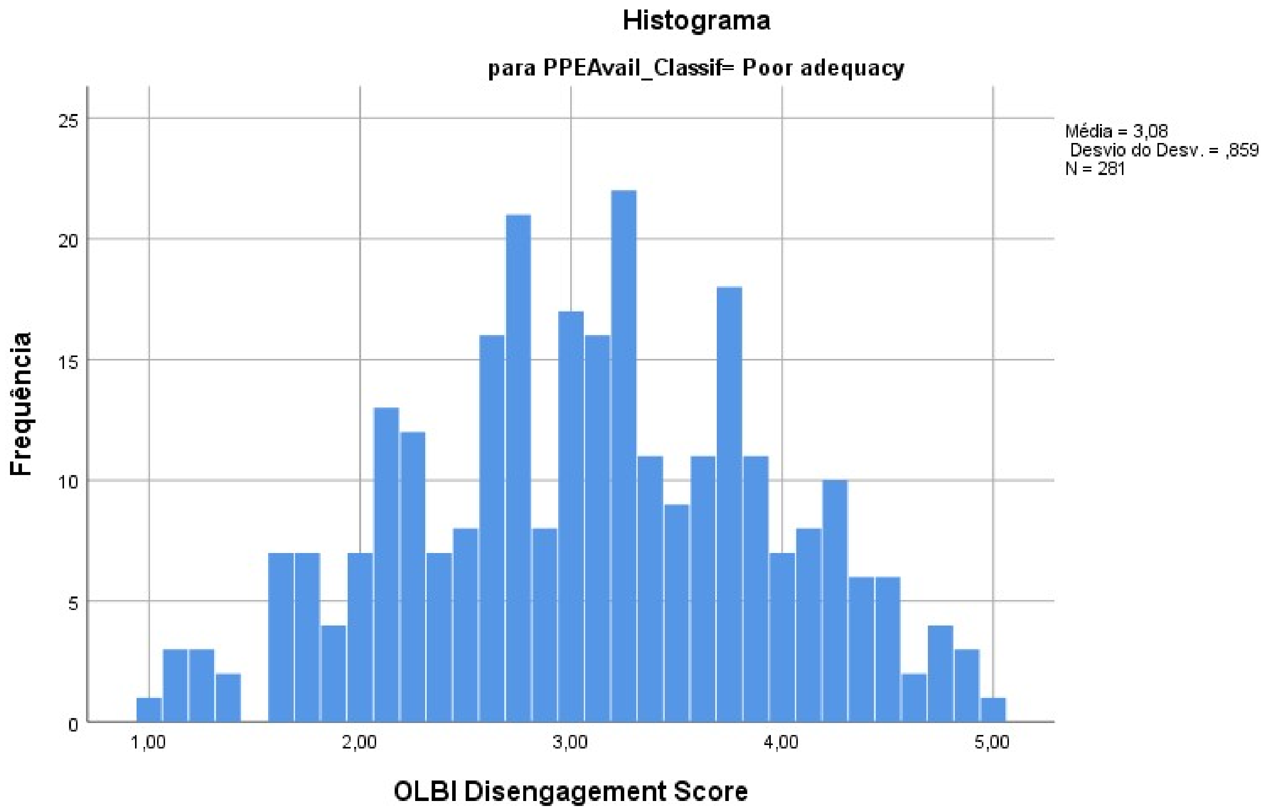

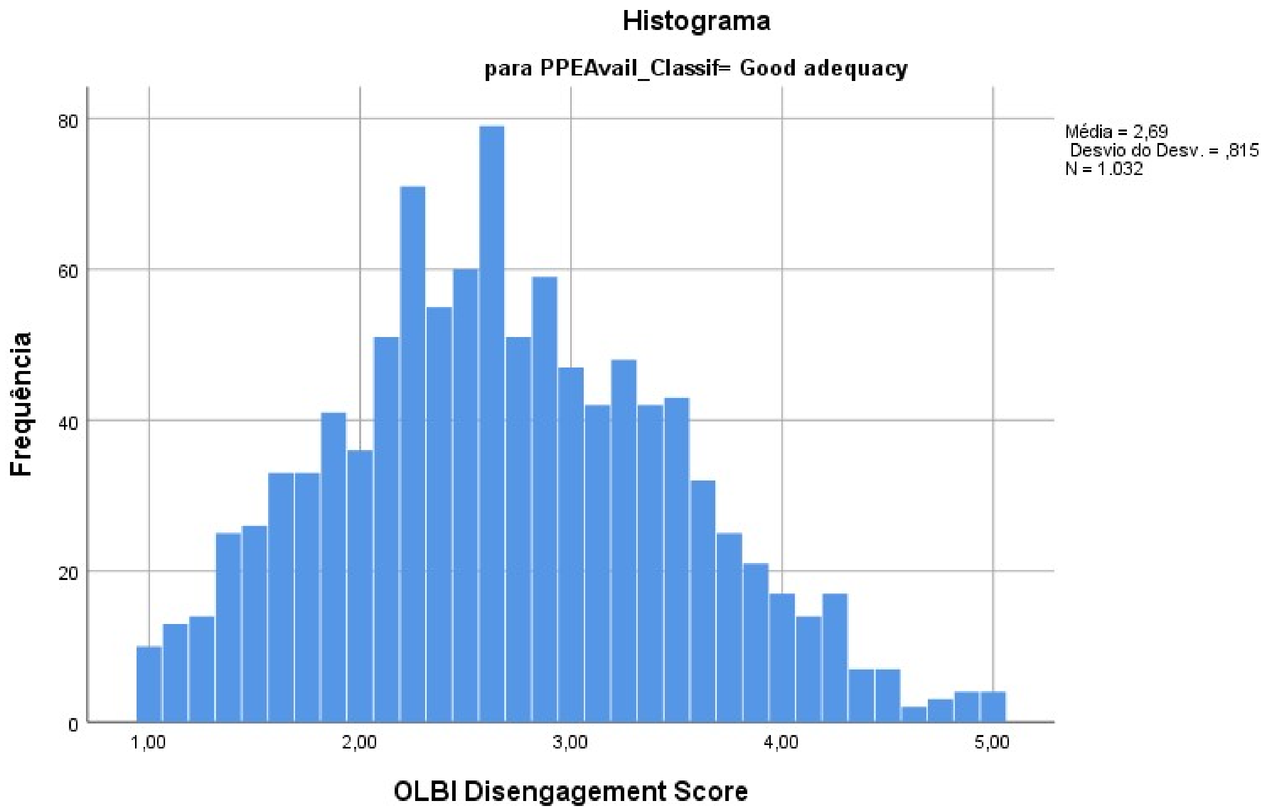

Gráfico Q-Q normais

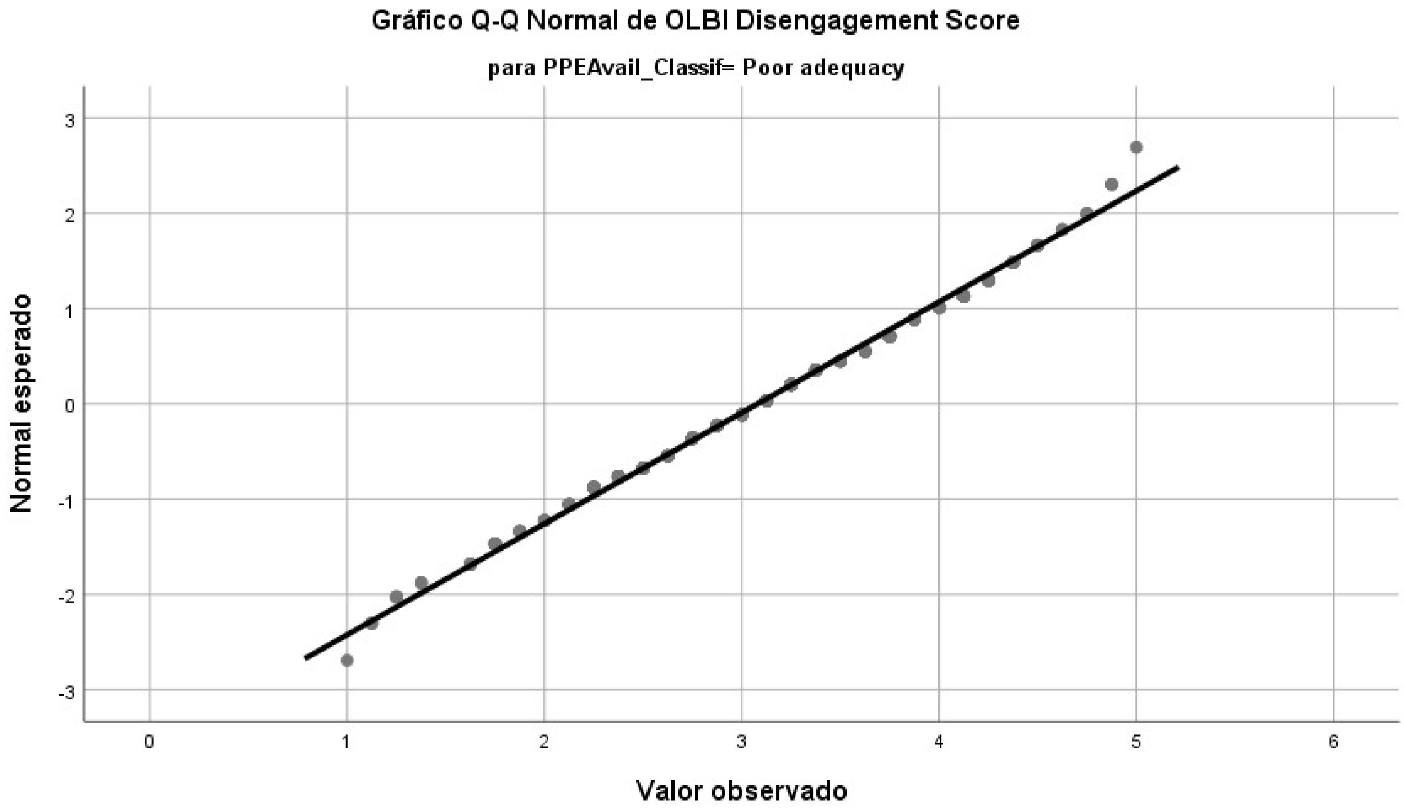

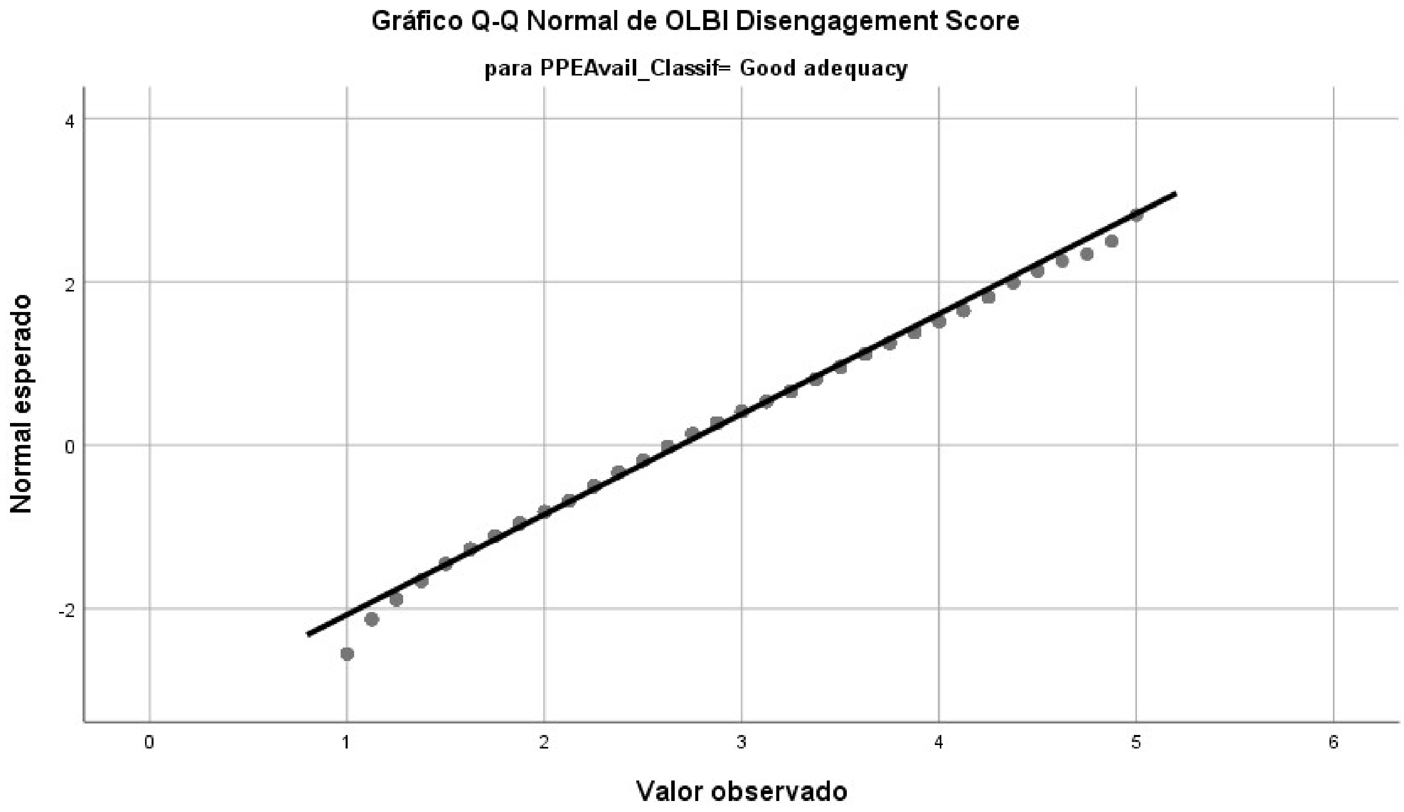

Gráfico Q-Q normais sem tendência

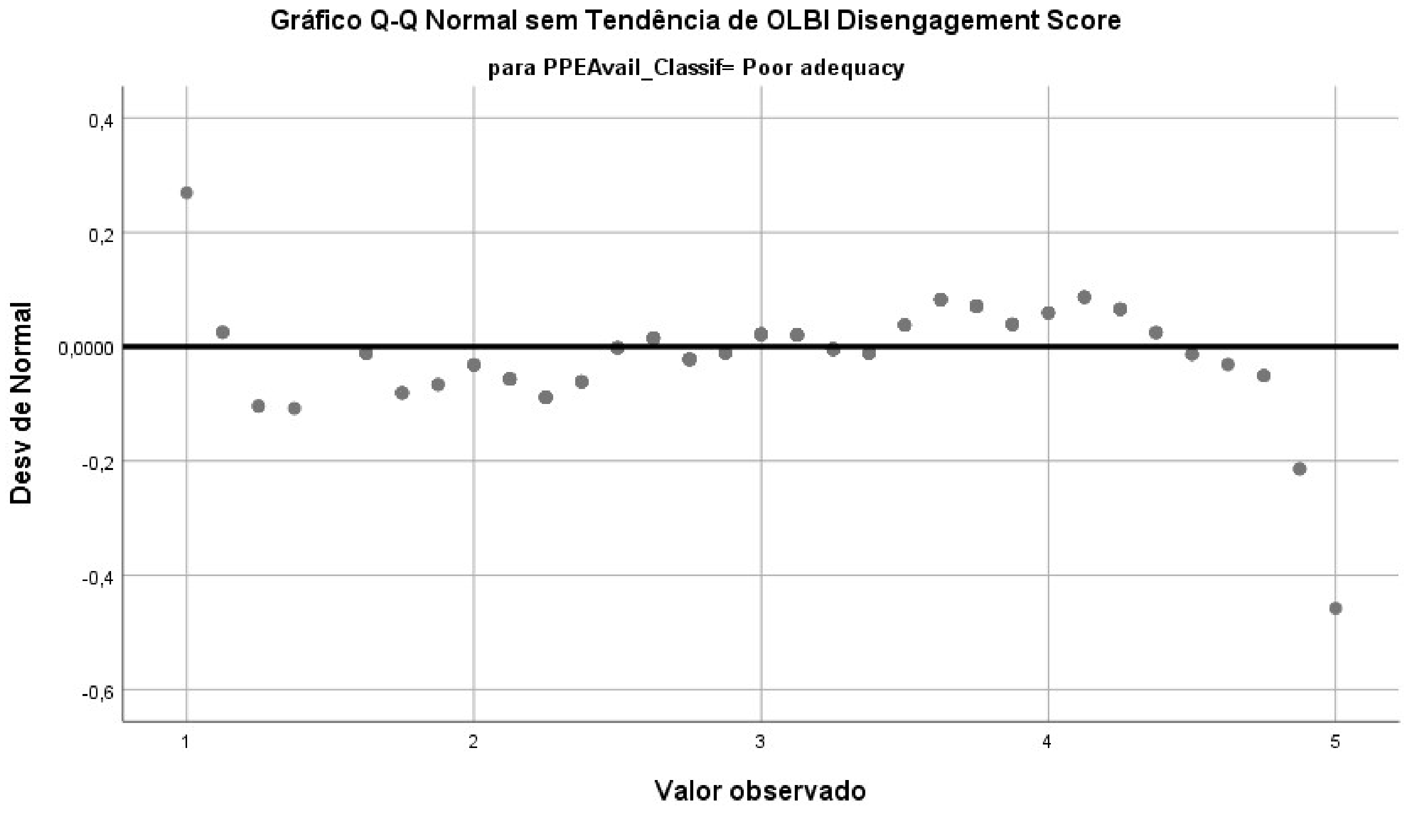

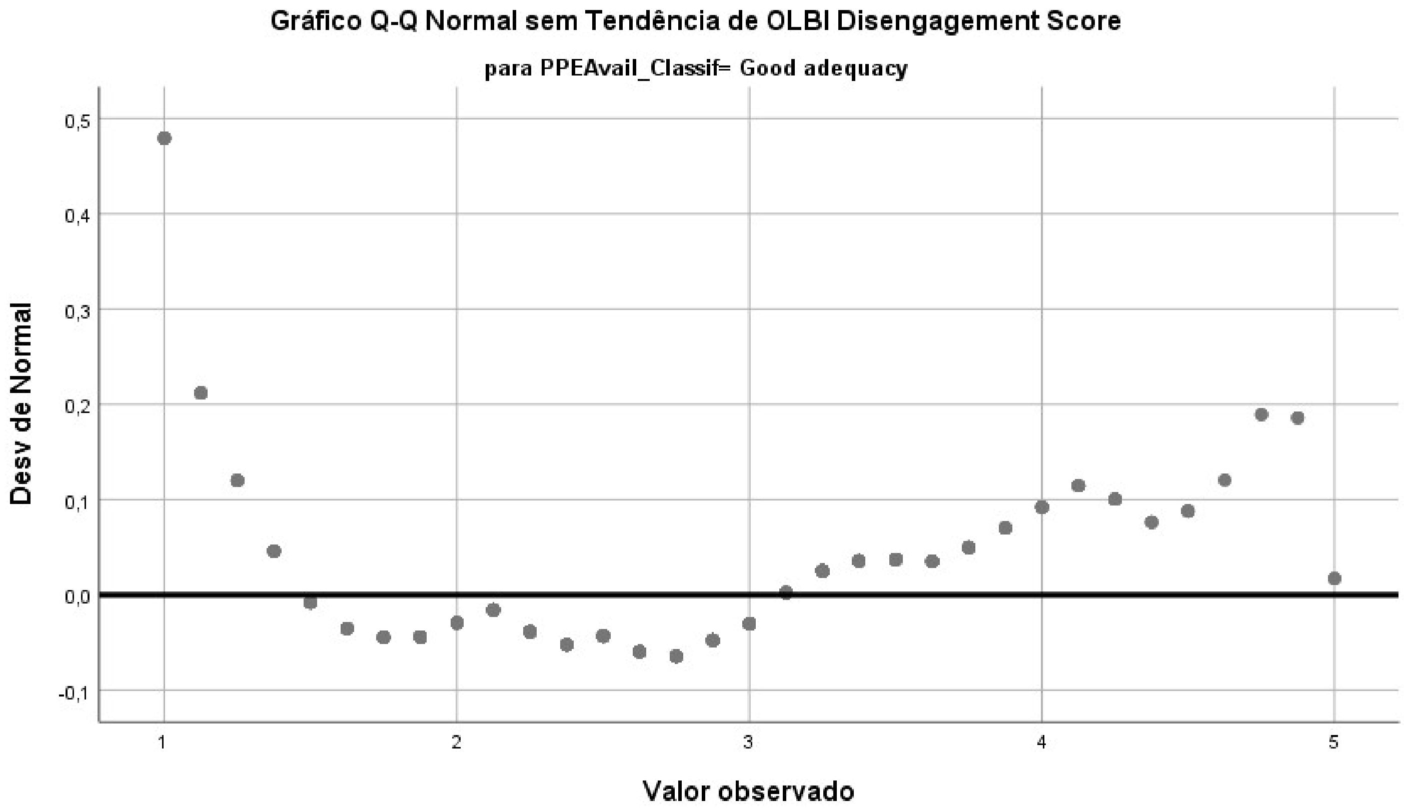

OLBI Exhaustion Score

Histogramas

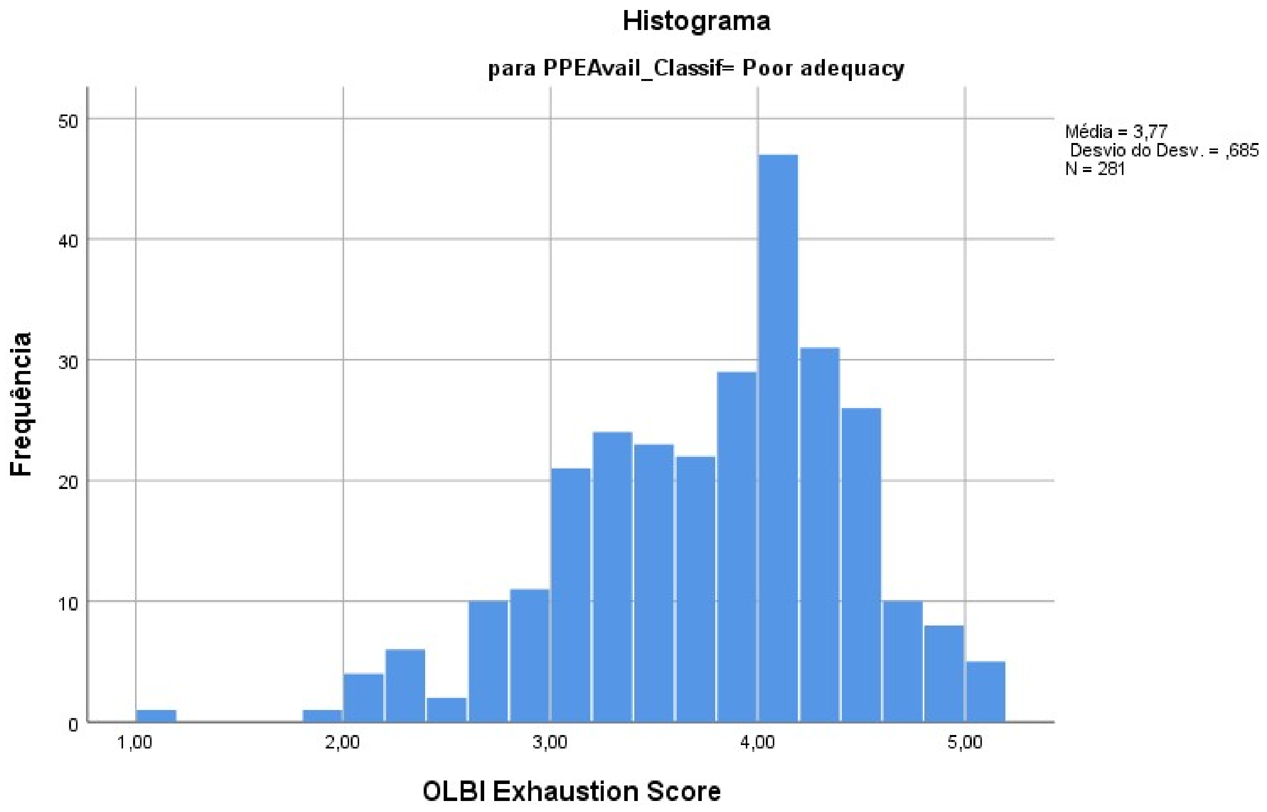

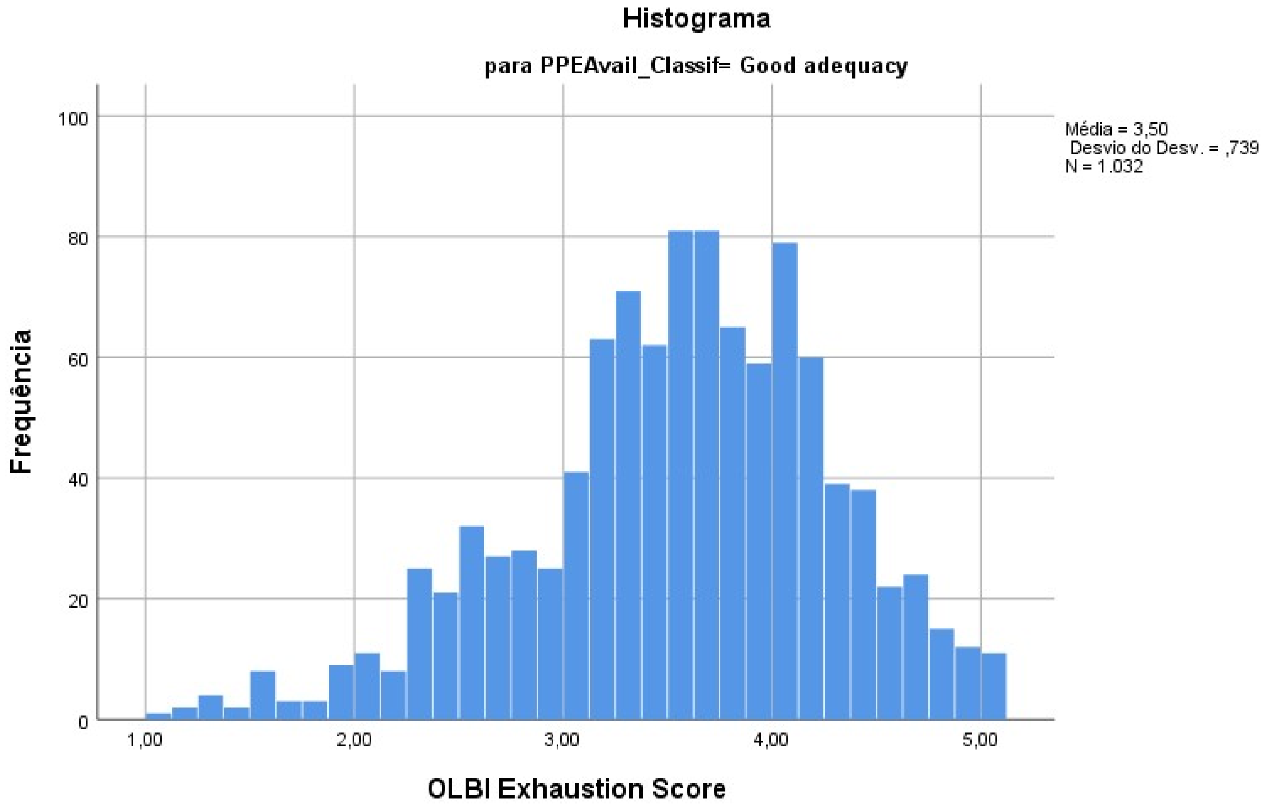

Gráfico Q-Q normais

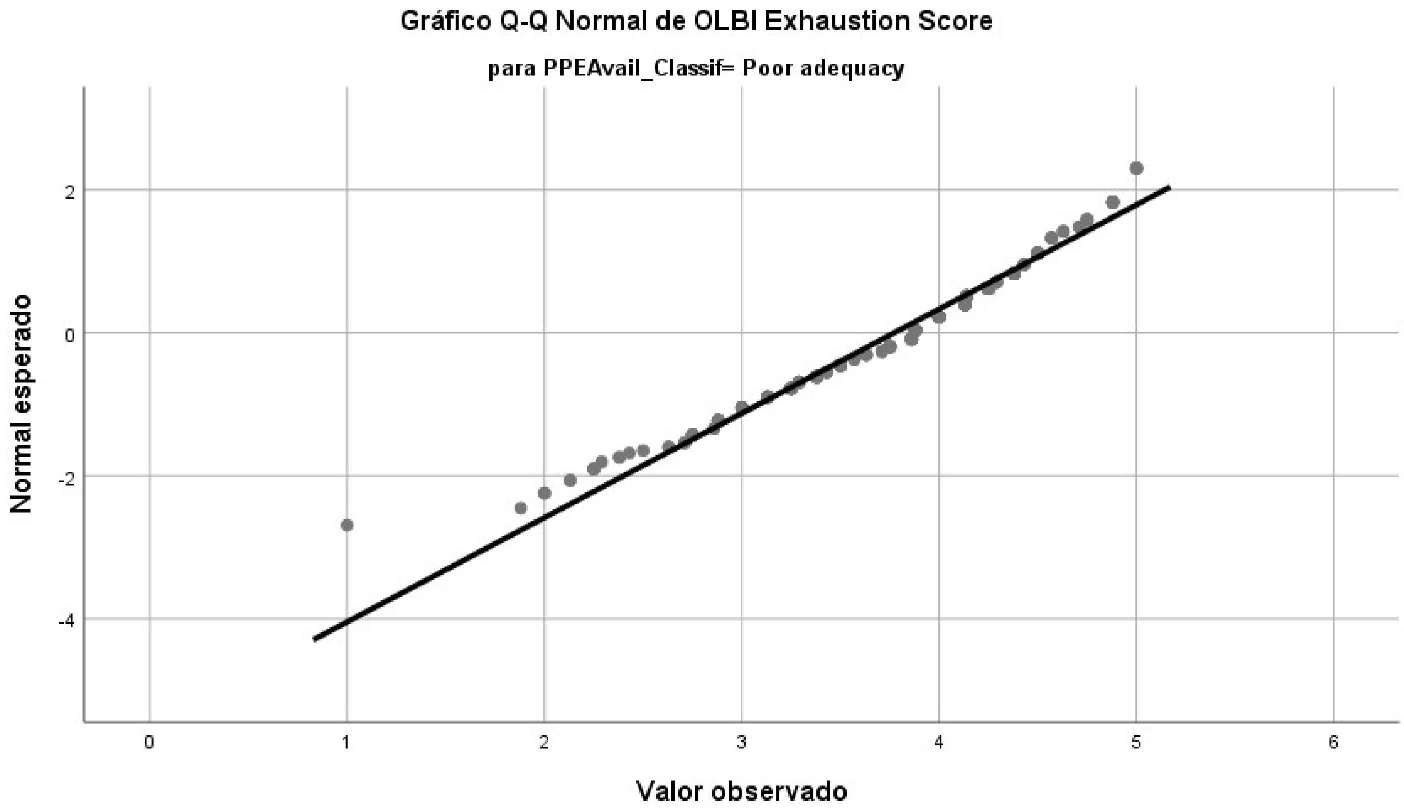

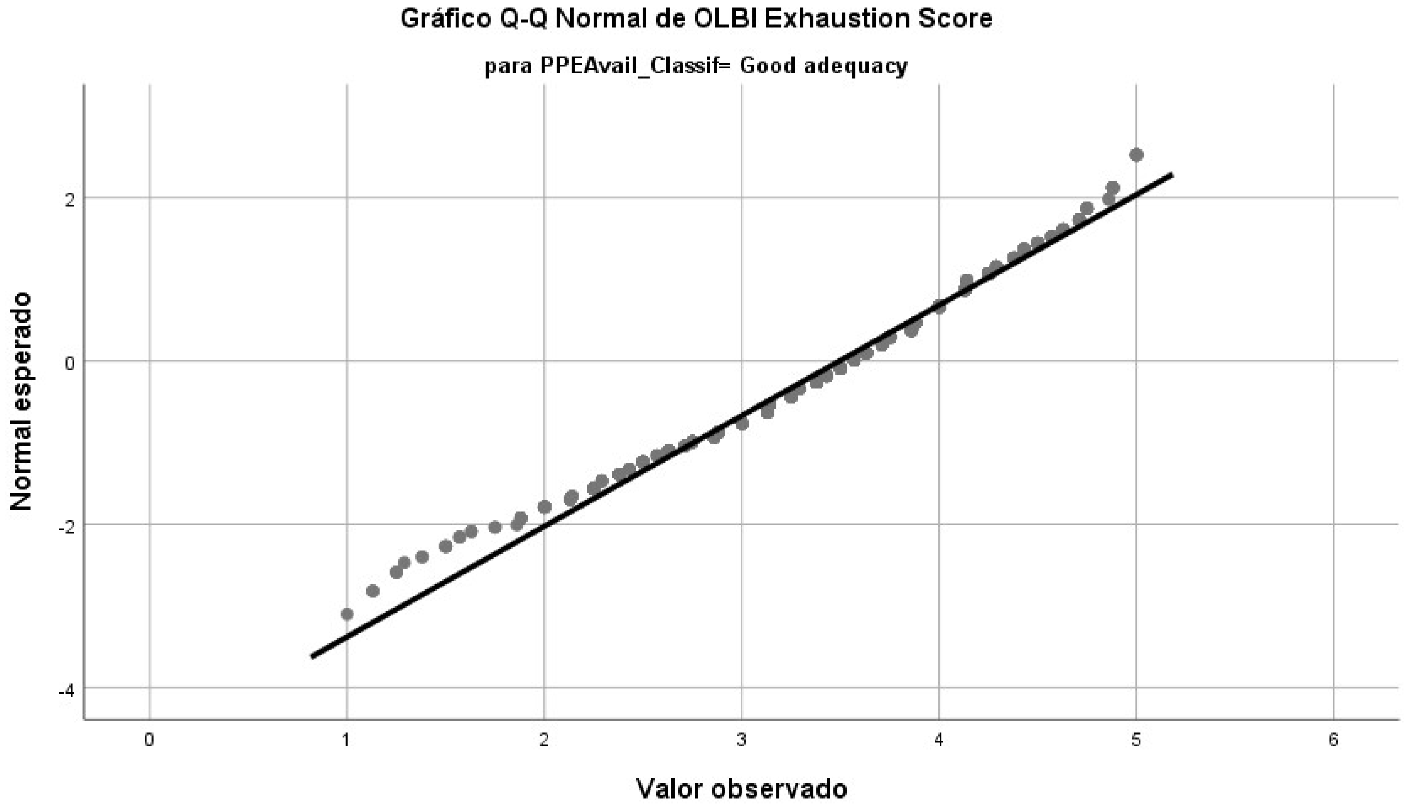

Gráfico Q-Q normais sem tendência

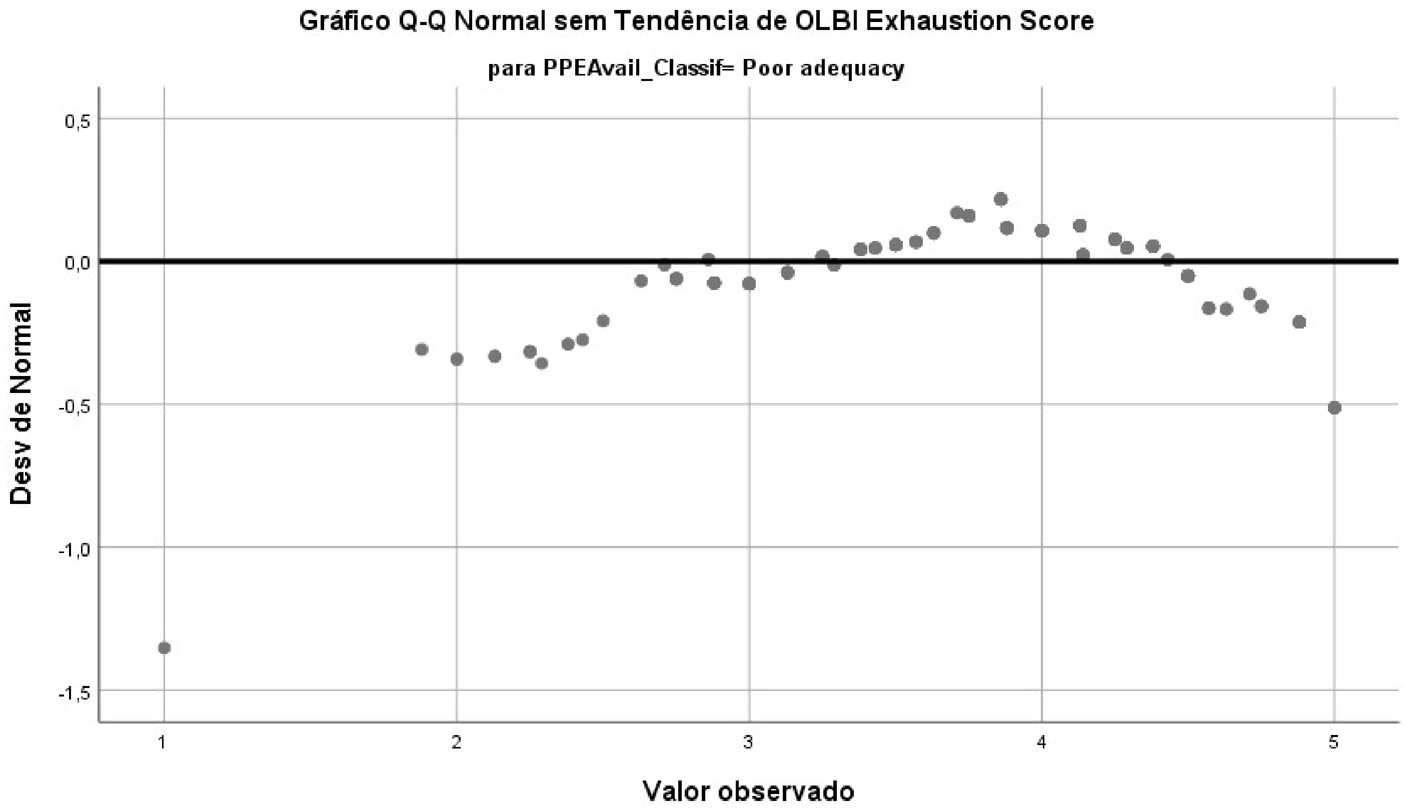

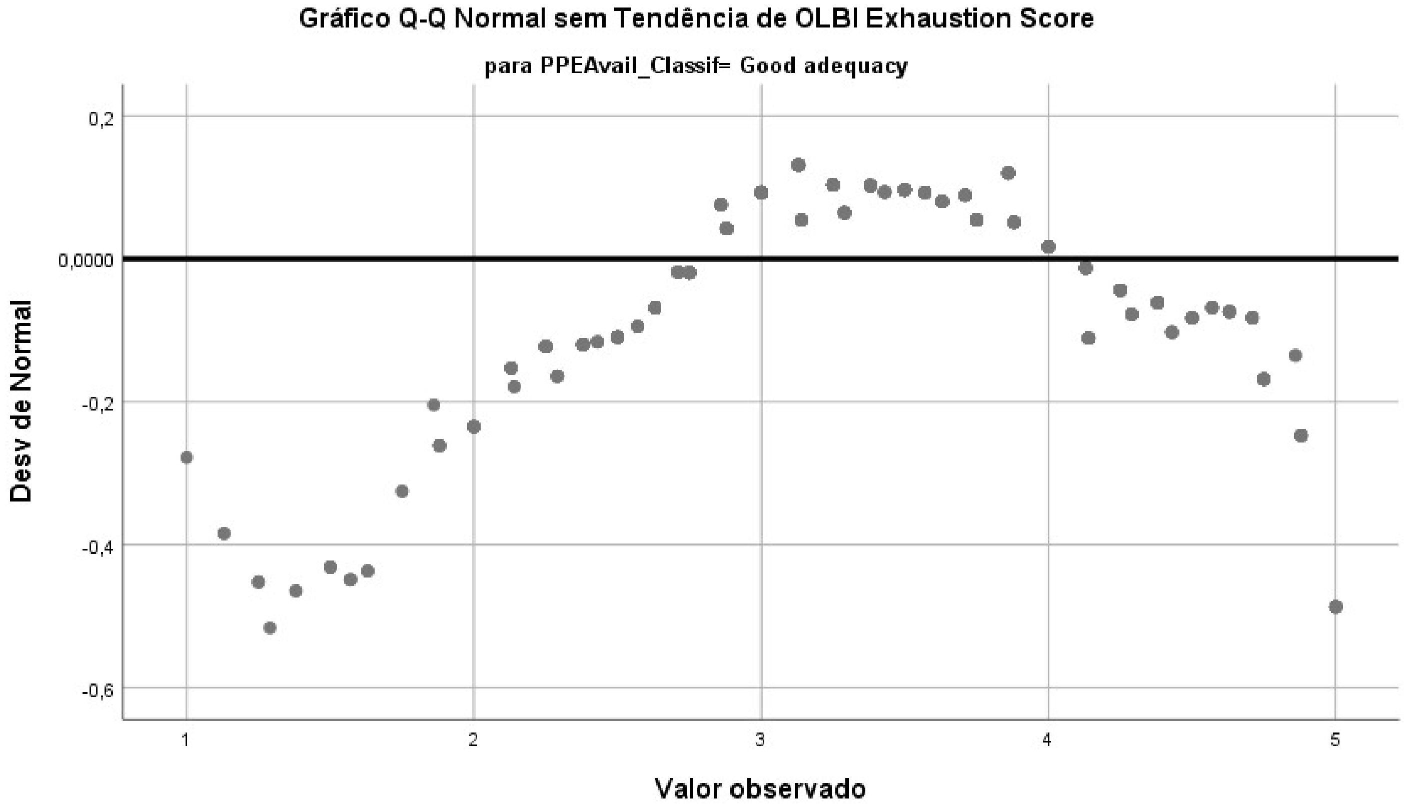

Cumulative weekly workload < or >= 60h (dichotomous)

Resumo de processamento do caso

	Cumulative weekly workload < or >= 60h (dichotomous)	Casos					
		Válido		Omisso		Total	
		N	Porcentagem	N	Porcentagem	N	Porcentagem
OLBI Disengagement Score	<=60h	541	100,0%	0	0,0%	541	100,0%
	>60h	772	100,0%	0	0,0%	772	100,0%
OLBI Exhaustion Score	<=60h	541	100,0%	0	0,0%	541	100,0%
	>60h	772	100,0%	0	0,0%	772	100,0%

Descritivos

	Cumulative weekly workload < or >= 60h (dichotomous)			Estatística	Erro Erro
OLBI Disengagement Score	<=60h	Média		2,7426	,03603
		95% Intervalo de Confiança para Média	Limite inferior	2,6718	
			Limite superior	2,8134	
		5% da média aparada		2,7225	
		Mediana		2,6250	
		Variância		,702	
		Erro Desvio		,83801	
		Mínimo		1,00	
		Máximo		5,00	
		Intervalo		4,00	
		Amplitude interquartil		1,13	
		Assimetria		,293	,105
		Curtose		-,268	,210
	>60h	Média		2,7960	,03025
		95% Intervalo de Confiança para Média	Limite inferior	2,7366	
			Limite superior	2,8554	
		5% da média aparada		2,7916	
		Mediana		2,7500	
		Variância		,706	
		Erro Desvio		,84037	
		Mínimo		1,00	
		Máximo		5,00	
		Intervalo		4,00	
		Amplitude interquartil		1,13	
		Assimetria		,112	,088
		Curtose		-,516	,176
OLBI Exhaustion Score	<=60h	Média		3,4599	,03254
		95% Intervalo de Confiança para Média	Limite inferior	3,3960	
			Limite superior	3,5238	
		5% da média aparada		3,4809	
		Mediana		3,5000	
		Variância		,573	

		Erro Desvio		,75677	
		Mínimo		1,25	
		Máximo		5,00	
		Intervalo		3,75	
		Amplitude interquartil		1,00	
		Assimetria		-,434	,105
		Curtose		-,084	,210
	>60h	Média		3,6210	,02572
		95% Intervalo de Confiança para Média	Limite inferior	3,5705	
			Limite superior	3,6715	
		5% da média aparada		3,6461	
		Mediana		3,7100	
		Variância		,511	
		Erro Desvio		,71457	
		Mínimo		1,00	
		Máximo		5,00	
		Intervalo		4,00	
		Amplitude interquartil		,88	
		Assimetria		-,583	,088
		Curtose		,516	,176

Percentis

		Cumulative weekly workload < or >= 60h (dichotomous)	Percentis 5	10	25	50	75	90	95
Média Ponderada (Definição 1)	OLBI Disengagement Score	<=60h	1,3750	1,6250	2,1250	2,6250	3,2500	3,8750	4,2500
		>60h	1,3750	1,7500	2,2500	2,7500	3,3750	4,0000	4,2500
	OLBI Exhaustion Score	<=60h	2,0000	2,3800	3,0000	3,5000	4,0000	4,3800	4,6300
		>60h	2,2900	2,6300	3,2500	3,7100	4,1300	4,5000	4,7100
Teste de Tukey	OLBI Disengagement Score	<=60h			2,1250	2,6250	3,2500		
		>60h			2,2500	2,7500	3,3750		
	OLBI Exhaustion Score	<=60h			3,0000	3,5000	4,0000		
		>60h			3,2500	3,7100	4,1300		

Testes de Normalidade

	Cumulative weekly workload < or >= 60h (dichotomous)	Kolmogorov-Smirnov[a] Estatística	df	Sig.	Shapiro-Wilk Estatística	df	Sig.
OLBI Disengagement Score	<=60h	,057	541	,000	,987	541	,000
	>60h	,055	772	,000	,991	772	,000
OLBI Exhaustion Score	<=60h	,074	541	,000	,982	541	,000
	>60h	,068	772	,000	,977	772	,000

a. Correlação de Significância de Lilliefors

OLBI Disengagement Score

Histogramas

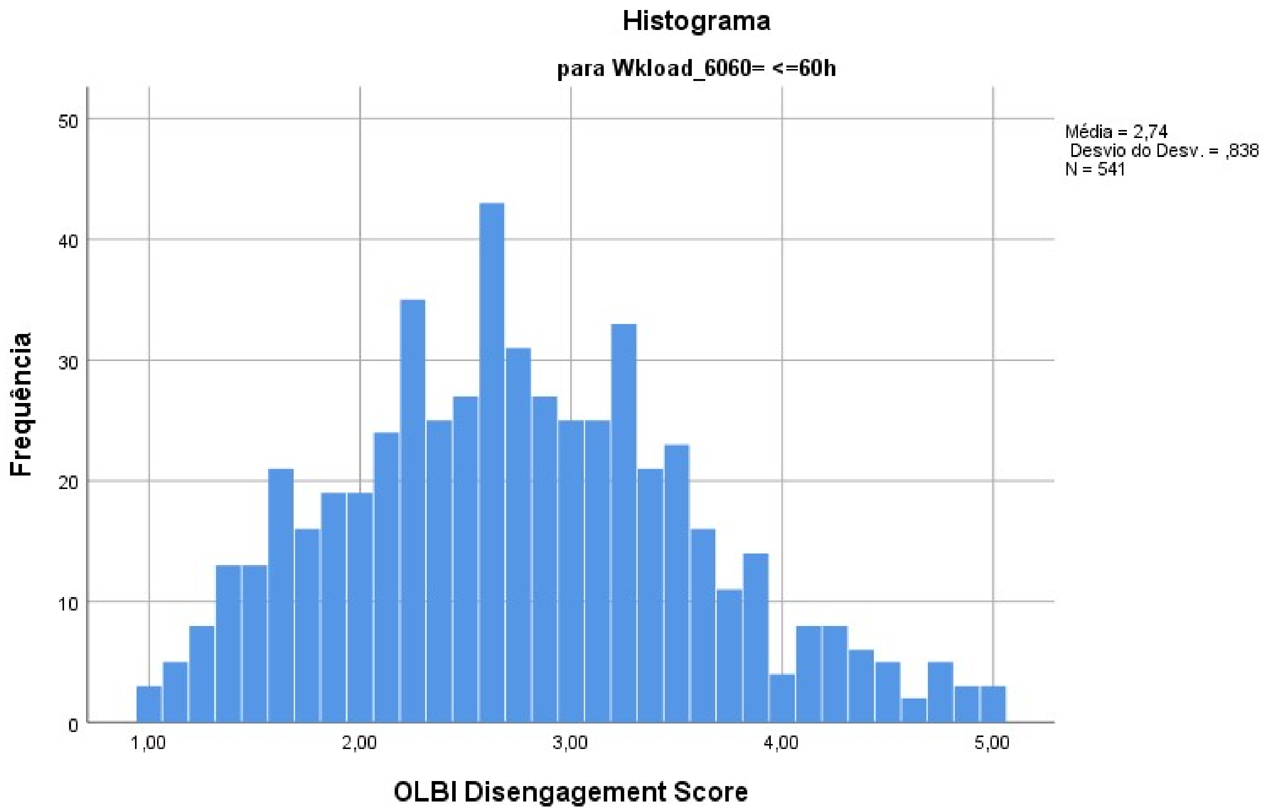

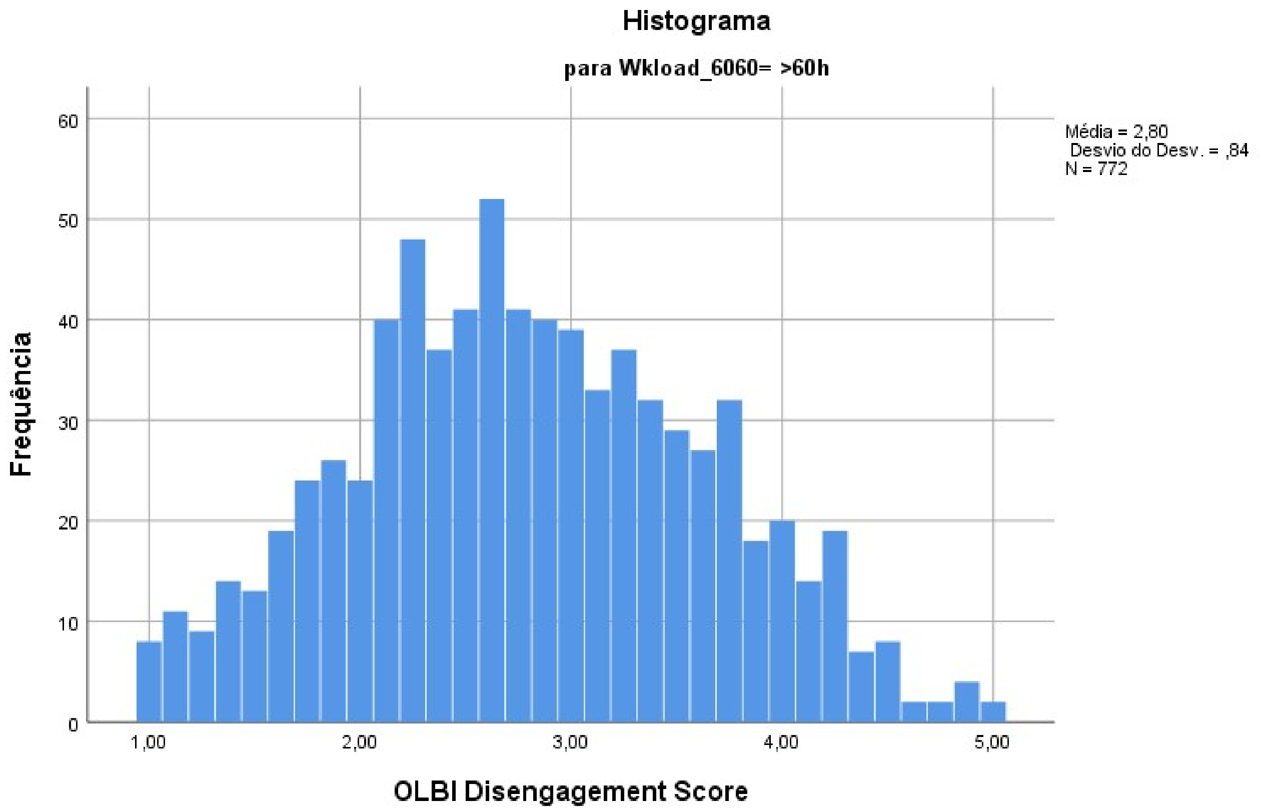

Gráfico Q-Q normais

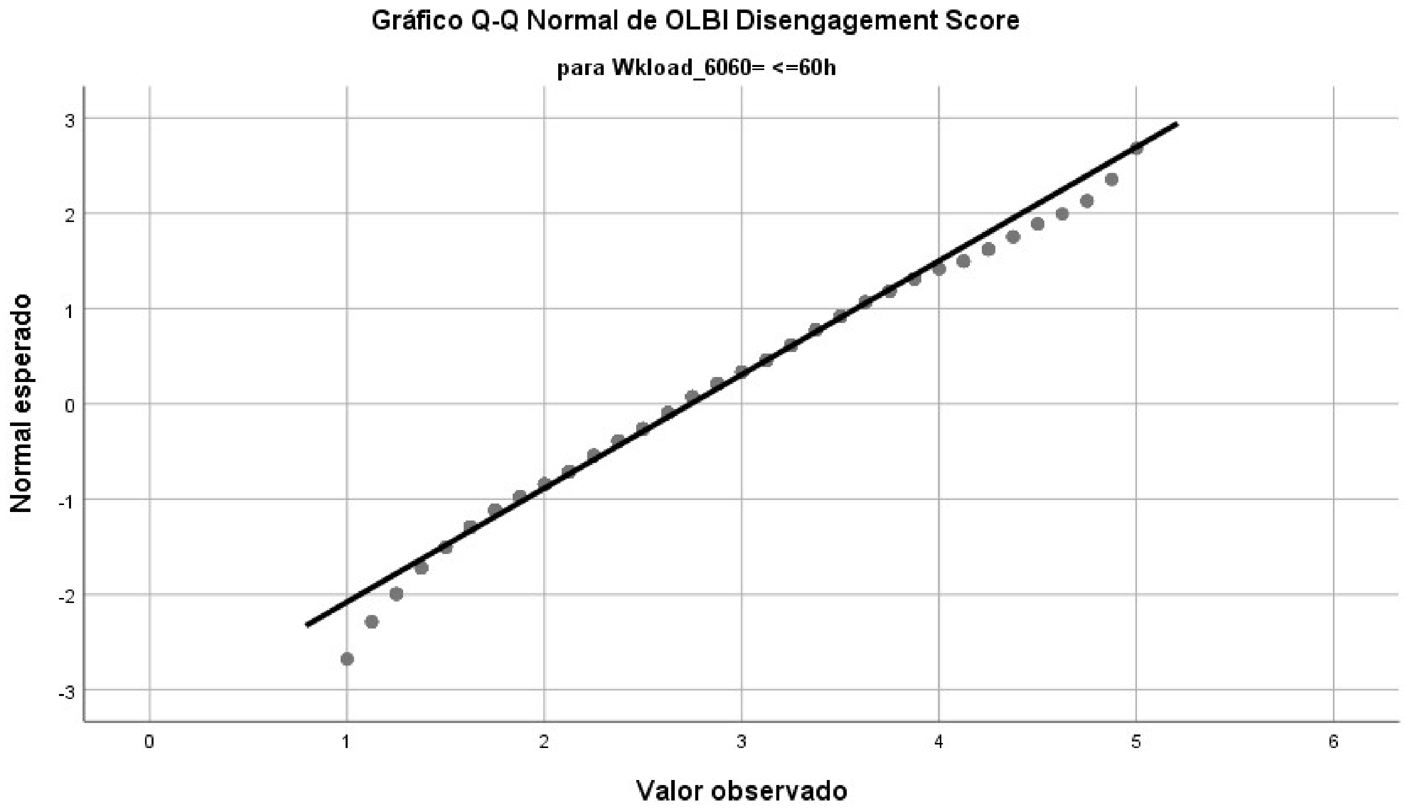

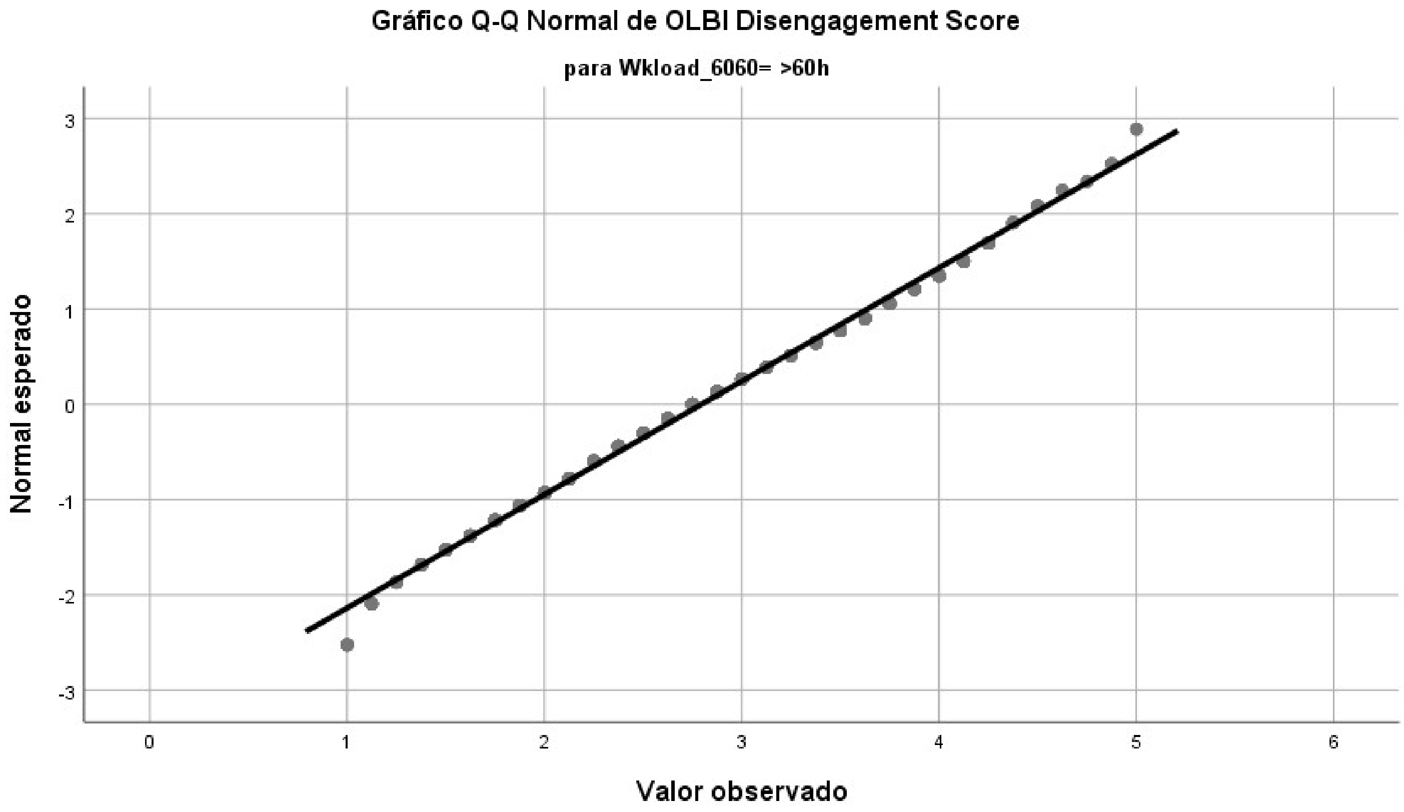

Gráfico Q-Q normais sem tendência

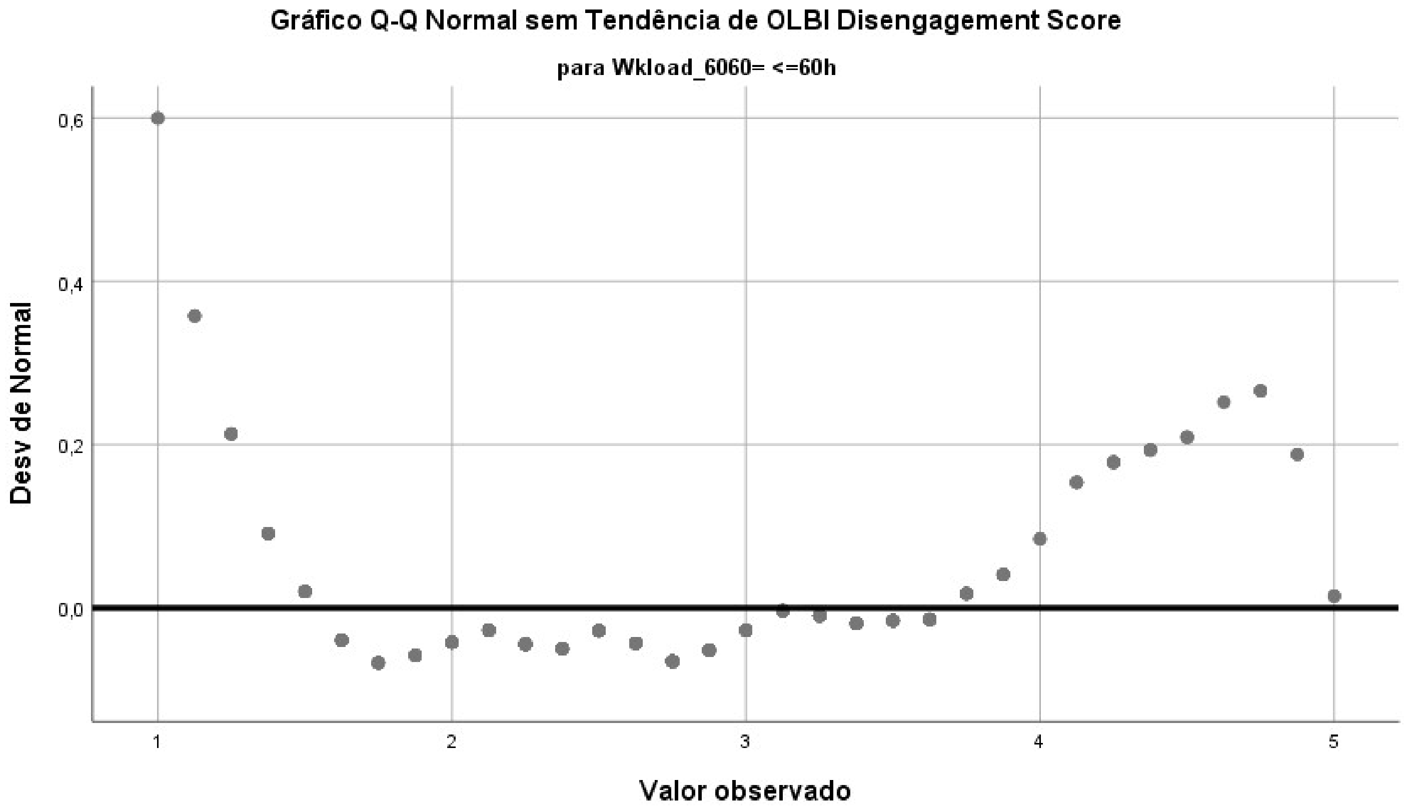

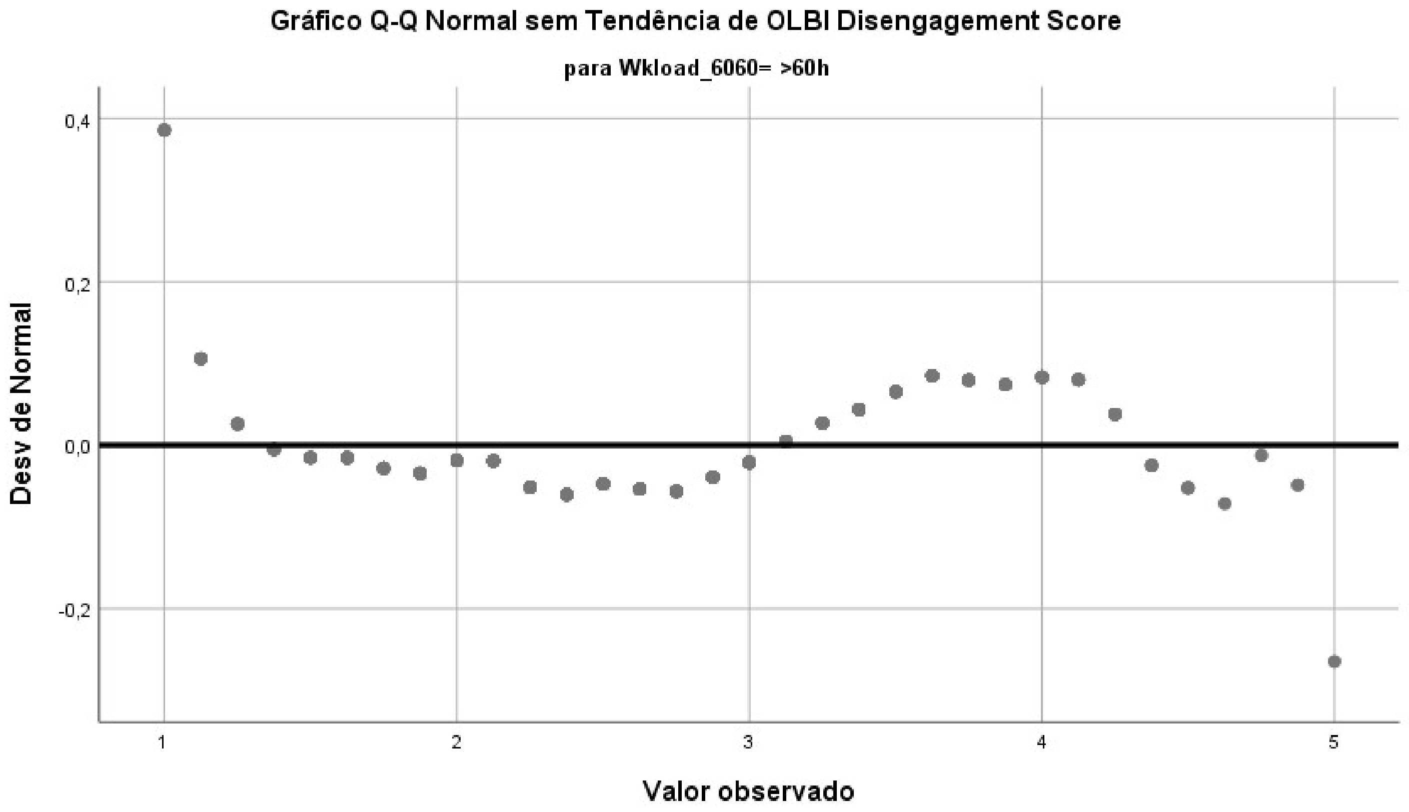

OLBI Exhaustion Score

Histogramas

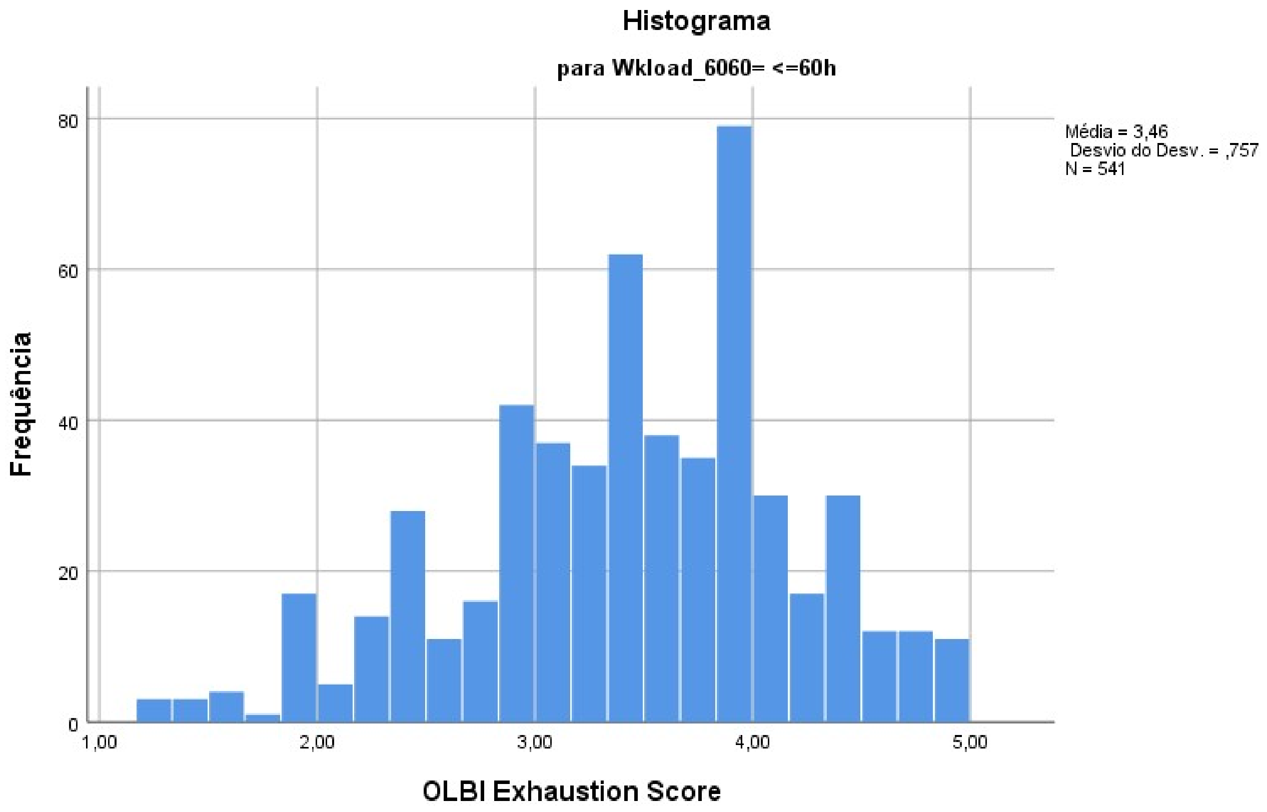

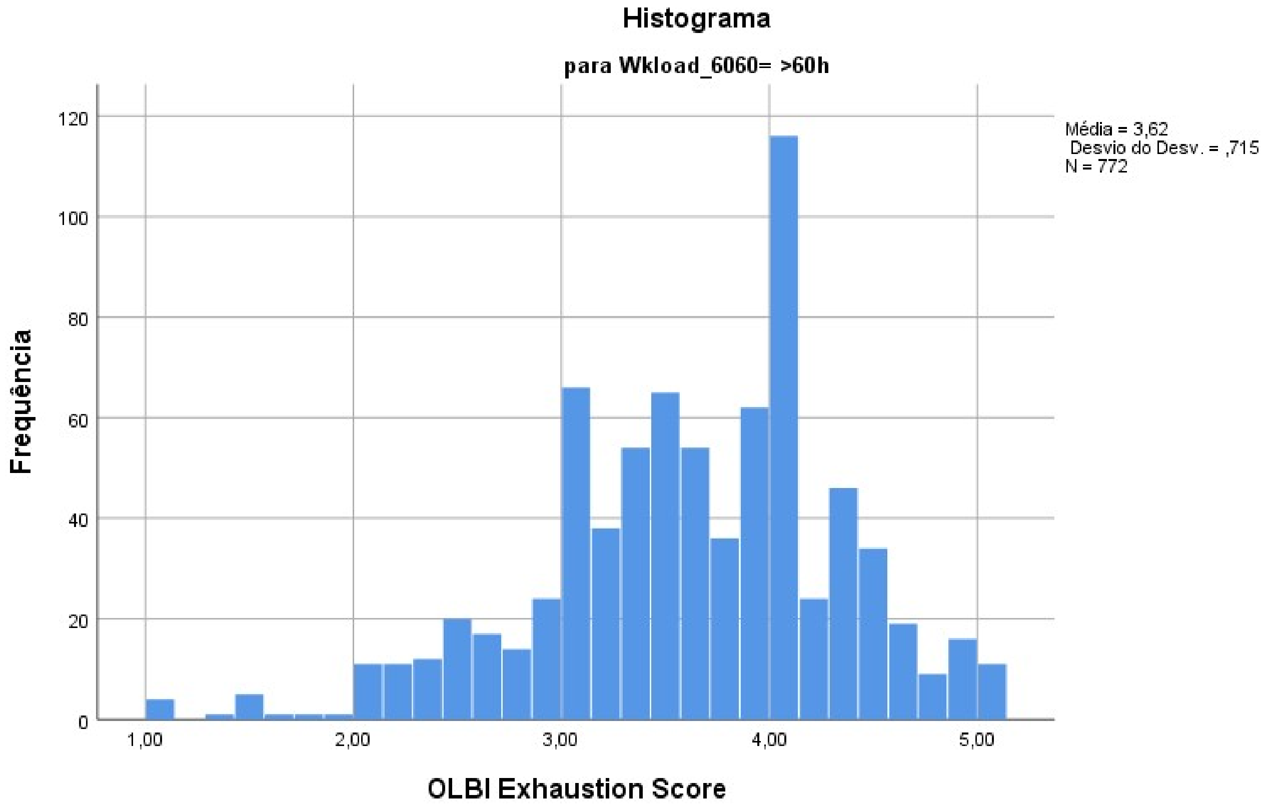

Gráfico Q-Q normais

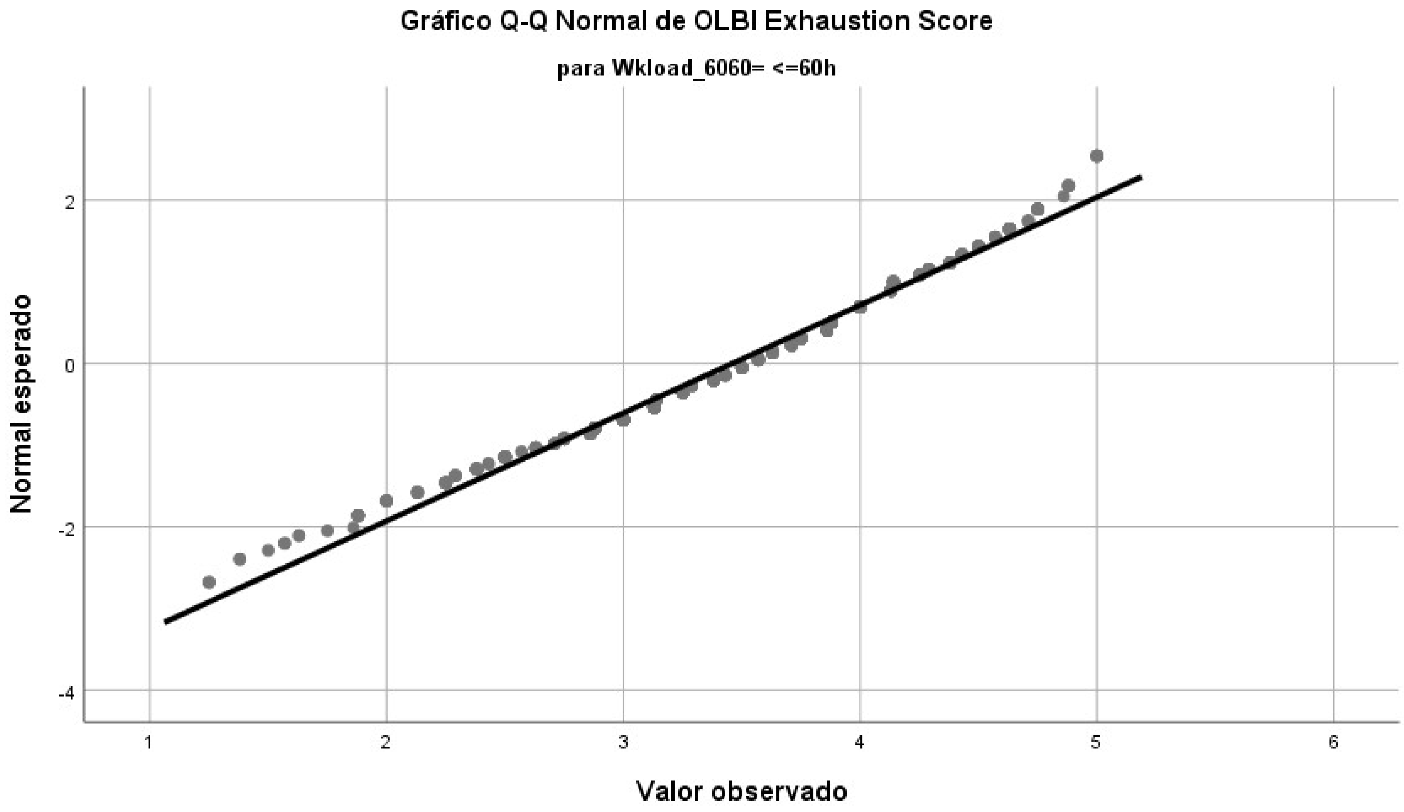

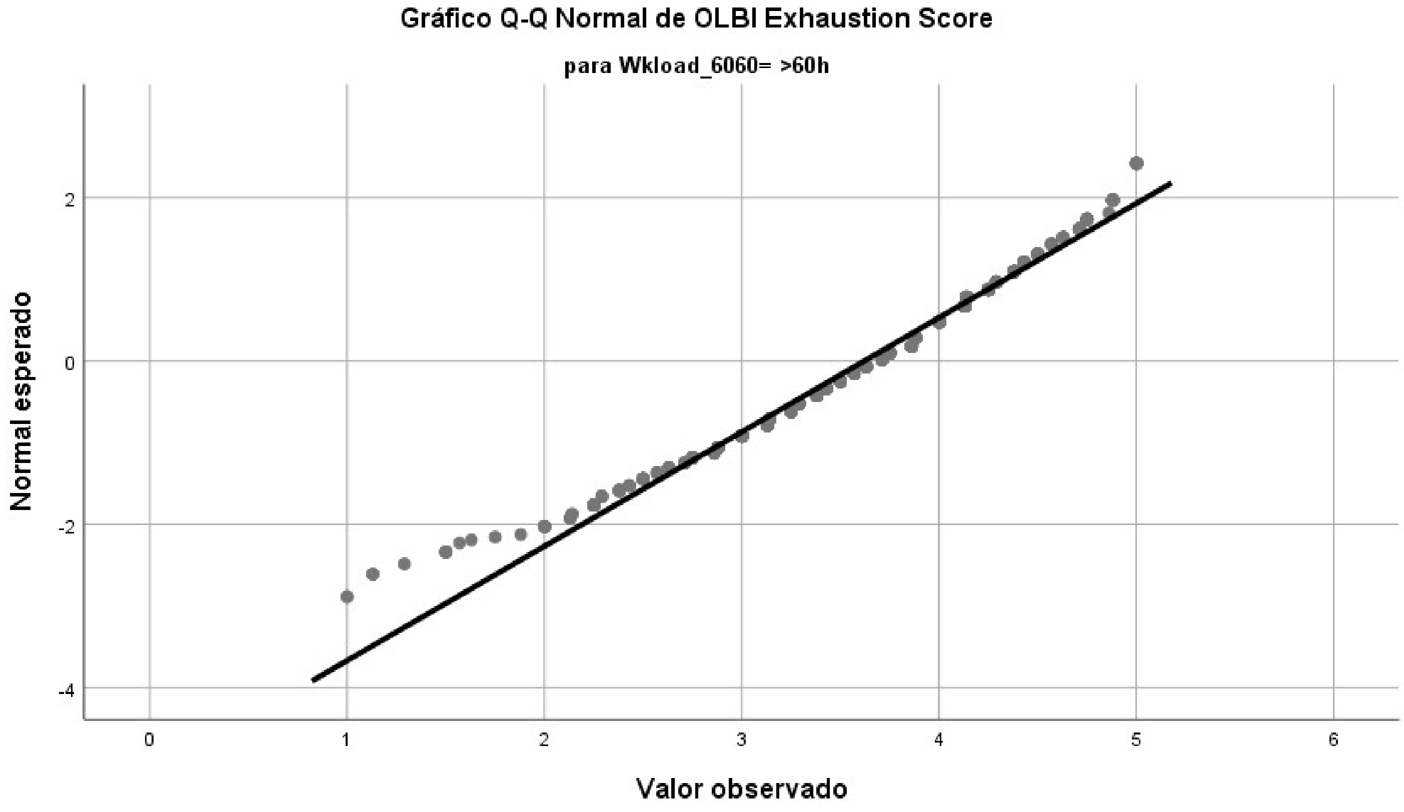

Gráfico Q-Q normais sem tendência

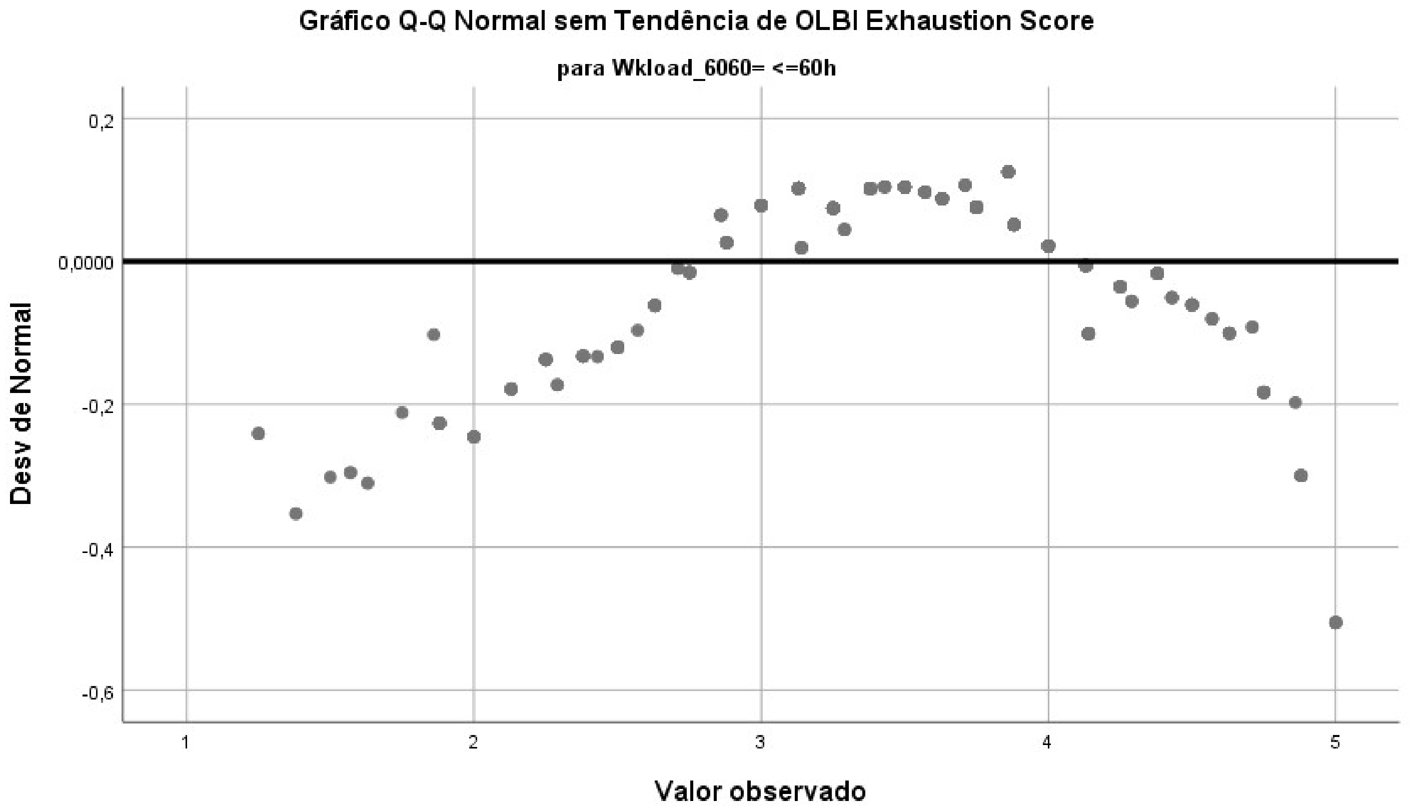

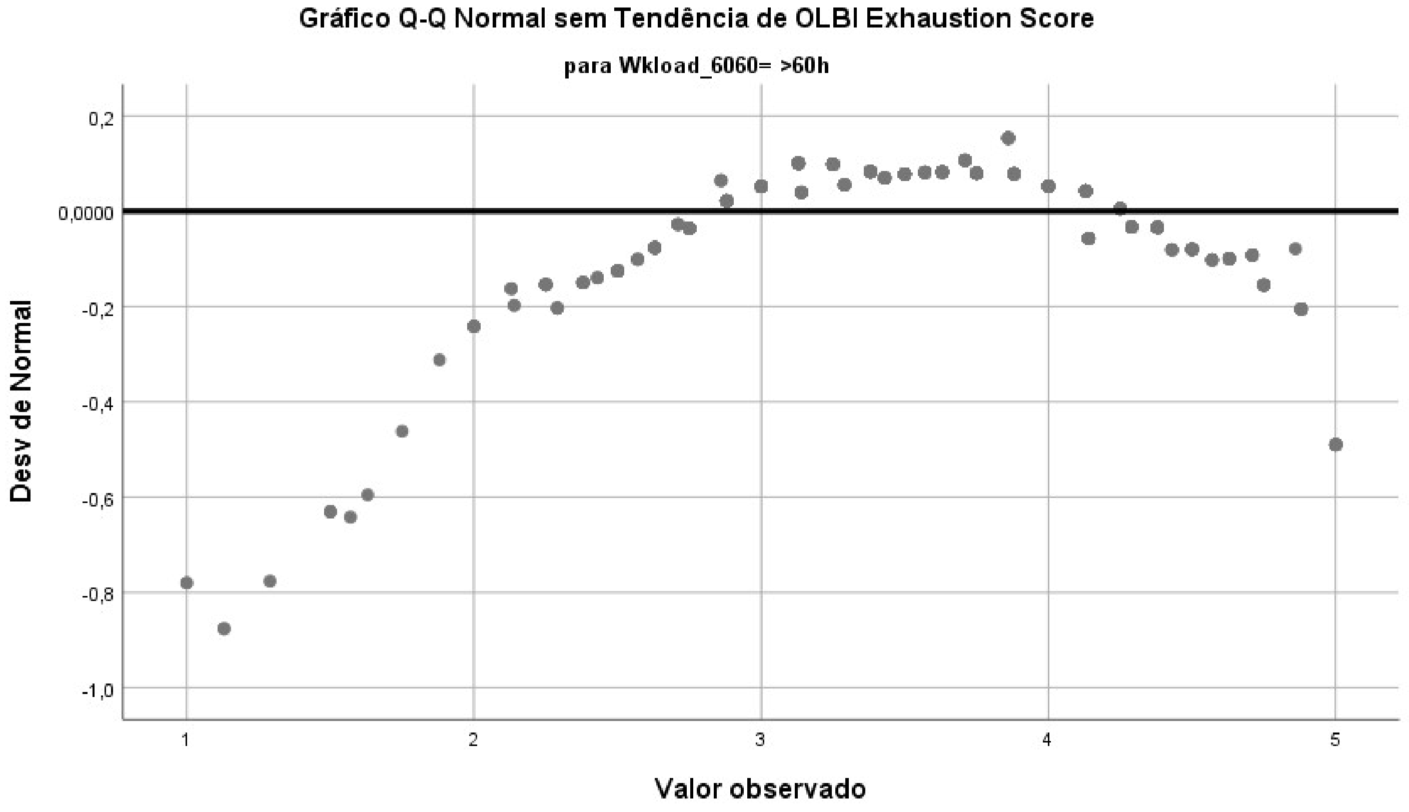


```
EXAMINE VARIABLES=OLBI_D OLBI_E BY Wkload_9090
  /PLOT HISTOGRAM NPPLOT
  /PERCENTILES(5,10,25,50,75,90,95) HAVERAGE
  /STATISTICS DESCRIPTIVES
  /CINTERVAL 95
  /MISSING PAIRWISE
  /NOTOTAL.
```

Explorar

Observações

Saída criada		21-SEP-2020 00:12:54
Comentários		
Entrada	Dados	C:\Users\User\Documents\Pesquisa\Fellow\FellowGenData_V1.sav
	Conjunto de dados ativo	ConjuntodeDados1
	Filtro	<none>
	Ponderação	<none>
	Arquivo Dividido	<none>
	N de linhas em arquivo de dados de trabalho	1313
Tratamento de valor omisso	Definição de omisso	Os valores omissos definidos pelo usuário para variáveis dependentes são tratados como omissos.
	Casos utilizados	As estatísticas são baseadas em casos sem valores omissos para a variável dependente ou fatores que estão sendo analisados.
Sintaxe		EXAMINE VARIABLES=OLBI_D OLBI_E BY Wkload_9090 /PLOT HISTOGRAM NPPLOT /PERCENTILES(5,10,25,50,75,90,95) HAVERAGE /STATISTICS DESCRIPTIVES /CINTERVAL 95 /MISSING PAIRWISE /NOTOTAL.
Recursos	Tempo do processador	00:00:01,66
	Tempo decorrido	00:00:01,65

Cumulative weekly workload < or >= 90h (dichotomous)

Resumo de processamento do caso

	Cumulative weekly workload <	Casos

	or >= 90h (dichotomous)	Válido N	Válido Porcentagem	Omisso N	Omisso Porcentagem	Total N	Total Porcentagem
OLBI Disengagement Score	<=90H	1223	100,0%	0	0,0%	1223	100,0%
	>90H	90	100,0%	0	0,0%	90	100,0%
OLBI Exhaustion Score	<=90H	1223	100,0%	0	0,0%	1223	100,0%
	>90H	90	100,0%	0	0,0%	90	100,0%

Descritivos

	Cumulative weekly workload < or >= 90h (dichotomous)			Estatística	Erro Erro
OLBI Disengagement Score	<=90H	Média		2,7535	,02380
		95% Intervalo de Confiança para Média	Limite inferior	2,7068	
			Limite superior	2,8002	
		5% da média aparada		2,7423	
		Mediana		2,7500	
		Variância		,693	
		Erro Desvio		,83229	
		Mínimo		1,00	
		Máximo		5,00	
		Intervalo		4,00	
		Amplitude interquartil		1,25	
		Assimetria		,190	,070
		Curtose		-,410	,140
	>90H	Média		3,0528	,09390
		95% Intervalo de Confiança para Média	Limite inferior	2,8662	
			Limite superior	3,2394	
		5% da média aparada		3,0525	
		Mediana		3,0000	
		Variância		,794	
		Erro Desvio		,89080	
		Mínimo		1,25	
		Máximo		4,88	
		Intervalo		3,63	
		Amplitude interquartil		1,38	
		Assimetria		,024	,254
		Curtose		-,601	,503
OLBI Exhaustion Score	<=90H	Média		3,5357	,02096
		95% Intervalo de Confiança para Média	Limite inferior	3,4946	
			Limite superior	3,5768	
		5% da média aparada		3,5582	
		Mediana		3,6300	
		Variância		,537	
		Erro Desvio		,73297	
		Mínimo		1,00	
		Máximo		5,00	

		Intervalo		4,00	
		Amplitude interquartil		,87	
		Assimetria		-,506	,070
		Curtose		,191	,140
	>90H	Média		3,8119	,07764
		95% Intervalo de Confiança para Média	Limite inferior	3,6576	
			Limite superior	3,9662	
		5% da média aparada		3,8510	
		Mediana		3,9400	
		Variância		,543	
		Erro Desvio		,73658	
		Mínimo		1,13	
		Máximo		5,00	
		Intervalo		3,87	
		Amplitude interquartil		1,00	
		Assimetria		-,947	,254
		Curtose		1,443	,503

Percentis

		Cumulative weekly workload < or >= 90h (dichotomous)	Percentis 5	10	25	50	75	90	95
Média Ponderada (Definição 1)	OLBI Disengagement Score	<=90H	1,3750	1,6250	2,1250	2,7500	3,3750	3,8750	4,2500
		>90H	1,5000	1,7625	2,3750	3,0000	3,7500	4,2500	4,5000
	OLBI Exhaustion Score	<=90H	2,2500	2,5000	3,1300	3,6300	4,0000	4,4100	4,6300
		>90H	2,6300	2,7500	3,3800	3,9400	4,3800	4,6300	4,8800
Teste de Tukey	OLBI Disengagement Score	<=90H			2,1250	2,7500	3,3750		
		>90H			2,3750	3,0000	3,7500		
	OLBI Exhaustion Score	<=90H			3,1300	3,6300	4,0000		
		>90H			3,3800	3,9400	4,3800		

Testes de Normalidade

	Cumulative weekly workload < or >= 90h (dichotomous)	Kolmogorov-Smirnov[a] Estatística	df	Sig.	Shapiro-Wilk Estatística	df	Sig.
OLBI Disengagement Score	<=90H	,058	1223	,000	,991	1223	,000
	>90H	,057	90	,200*	,985	90	,393
OLBI Exhaustion Score	<=90H	,068	1223	,000	,981	1223	,000
	>90H	,126	90	,001	,946	90	,001

*. Este é um limite inferior da significância verdadeira.

a. Correlação de Significância de Lilliefors

OLBI Disengagement Score

Histogramas

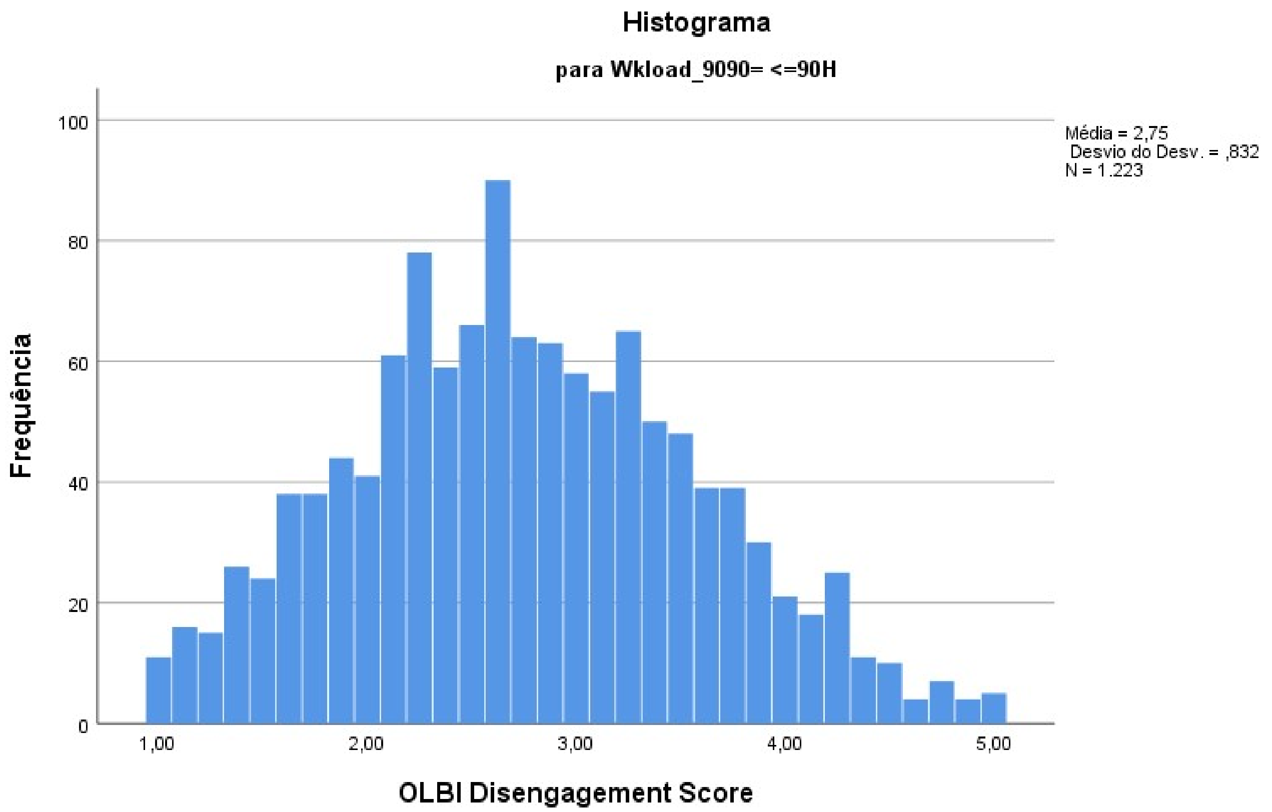

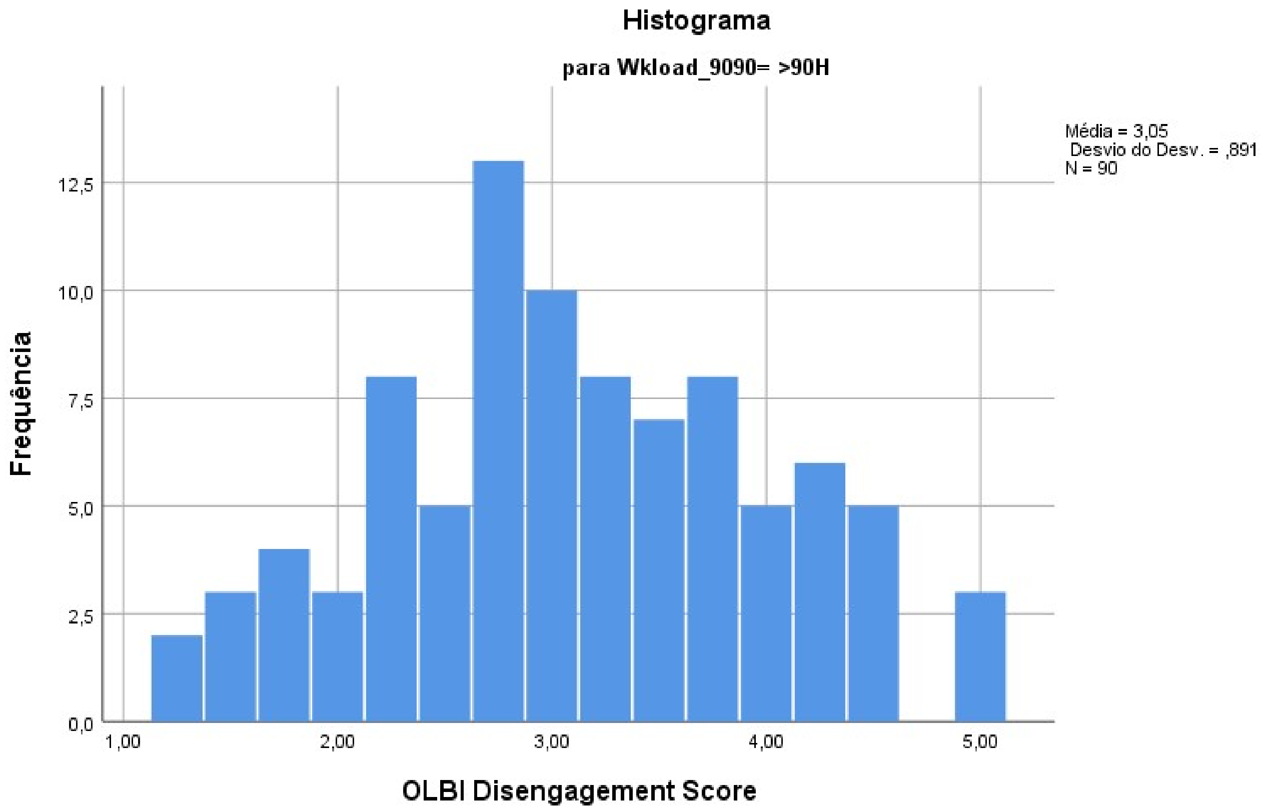

Gráfico Q-Q normais

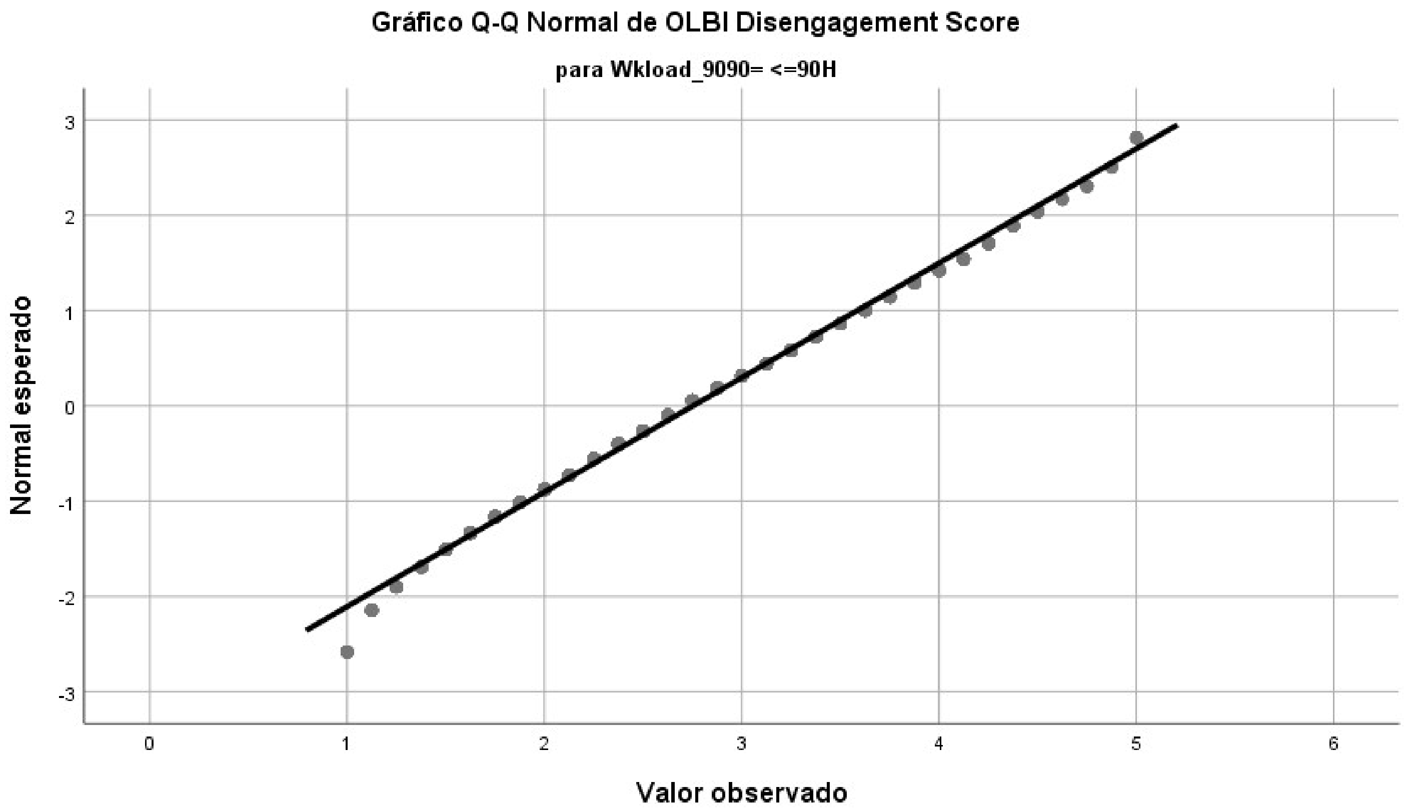

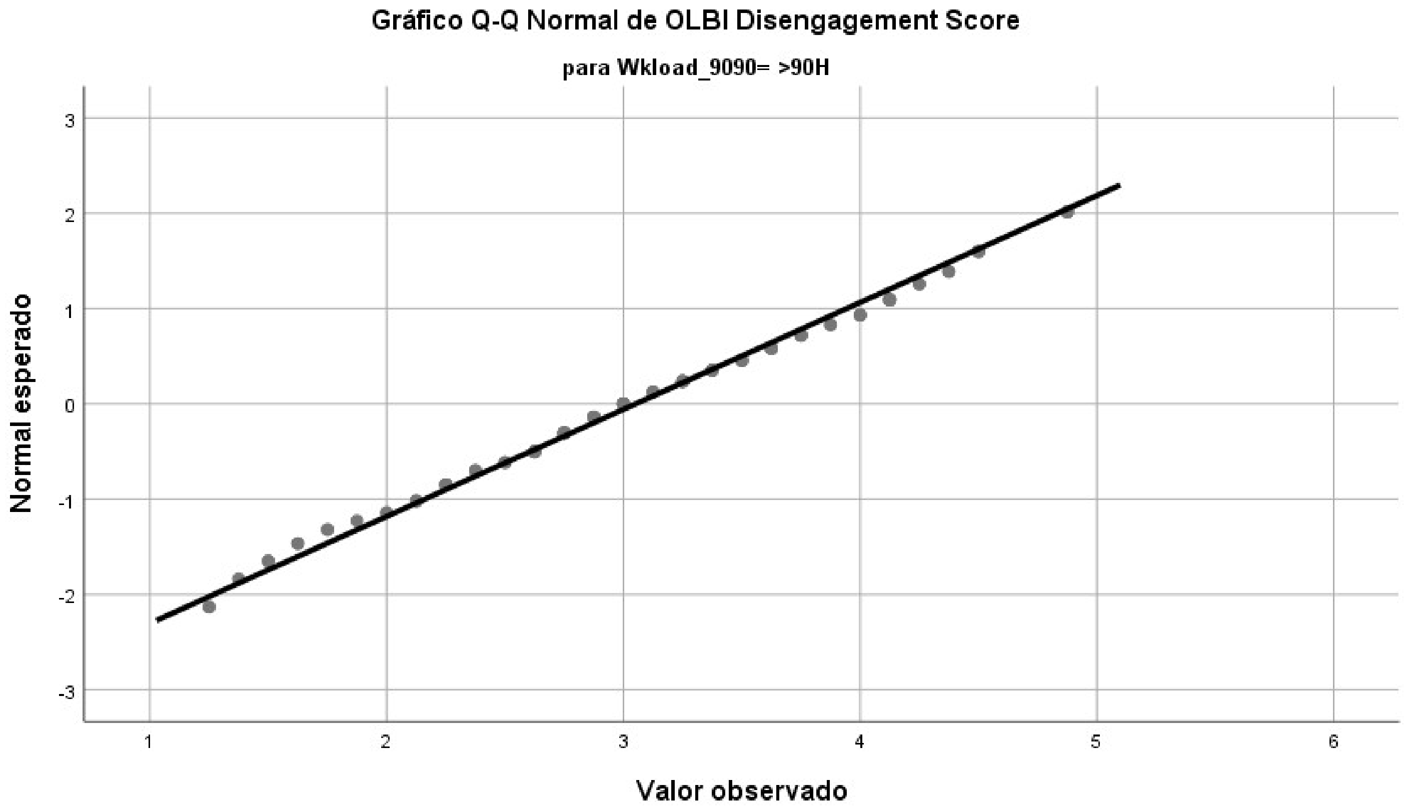

Gráfico Q-Q normais sem tendência

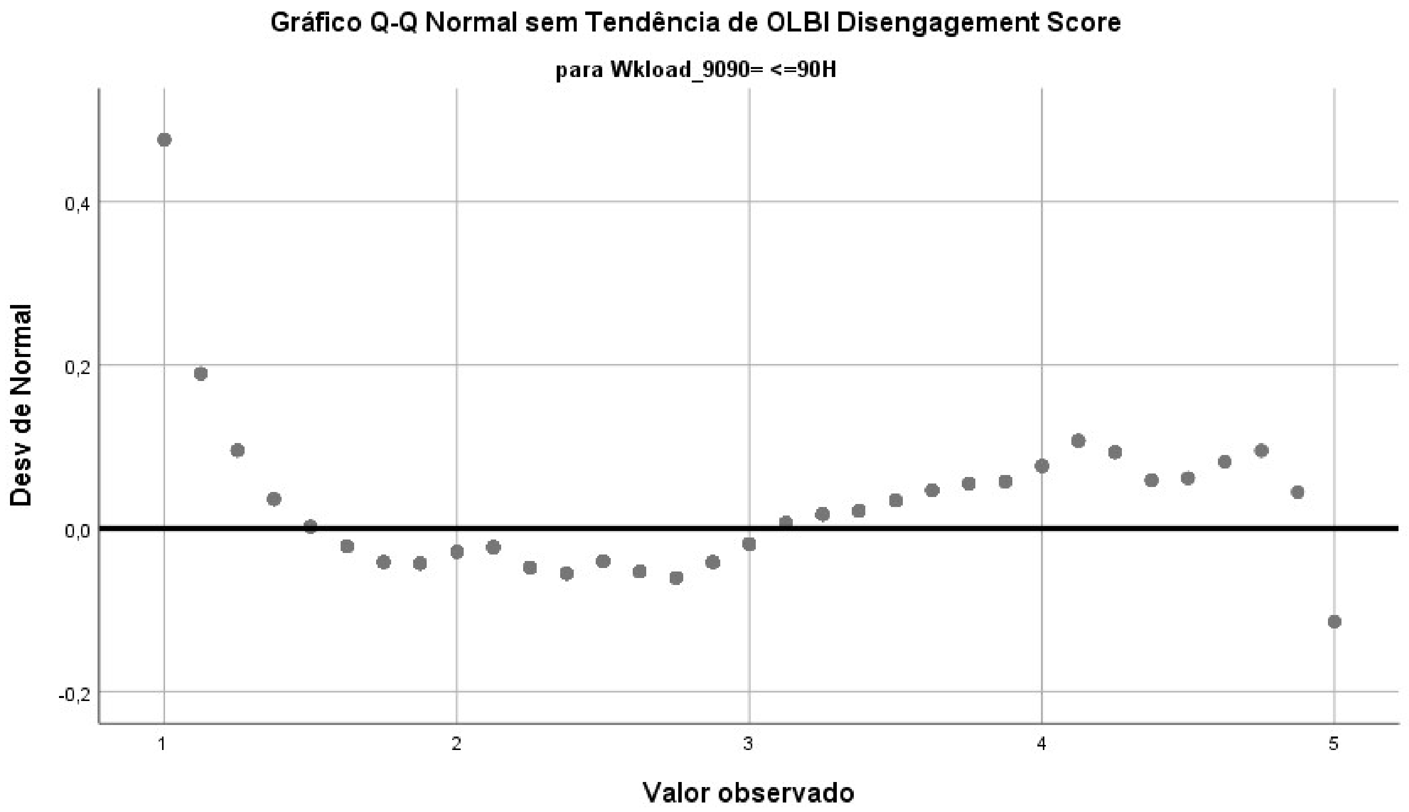

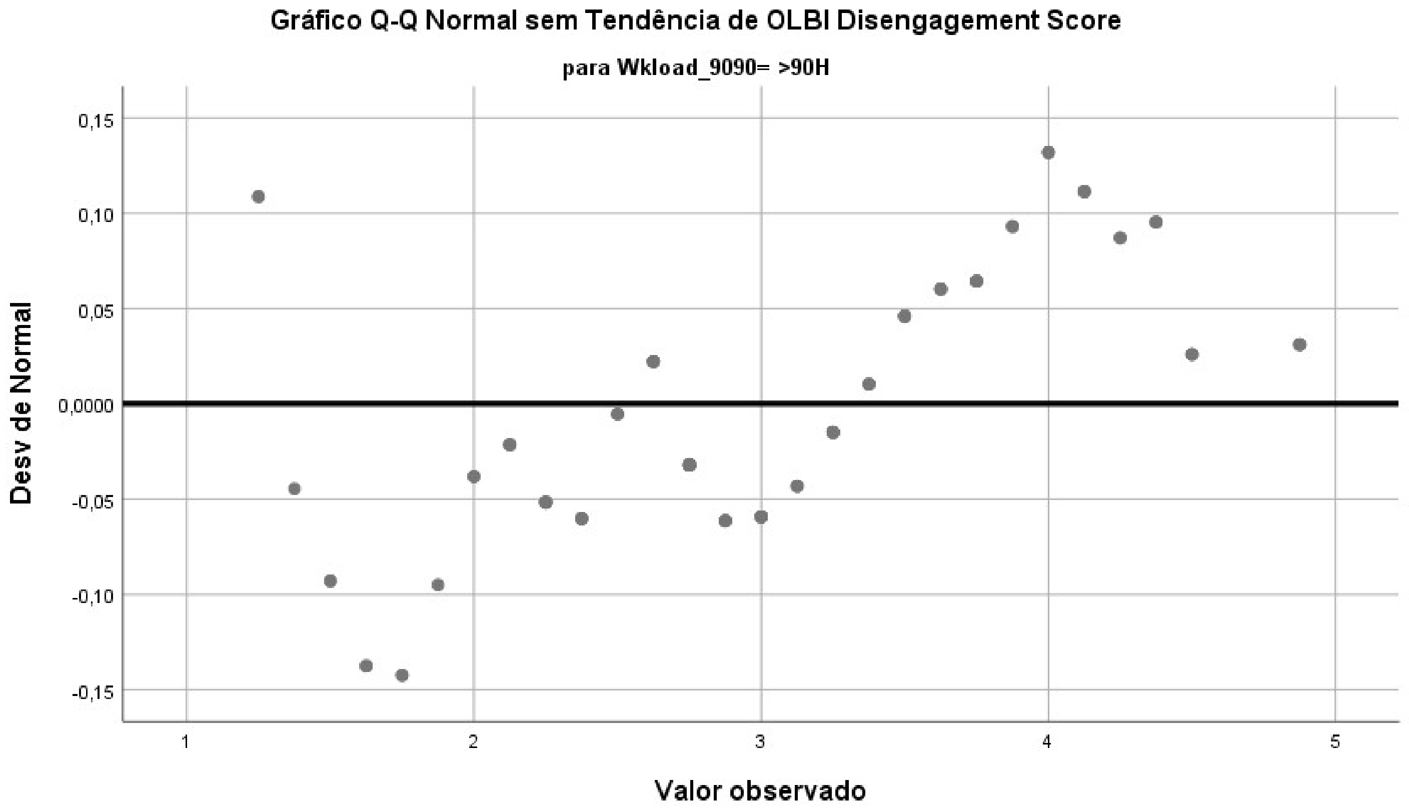

OLBI Exhaustion Score

Histogramas

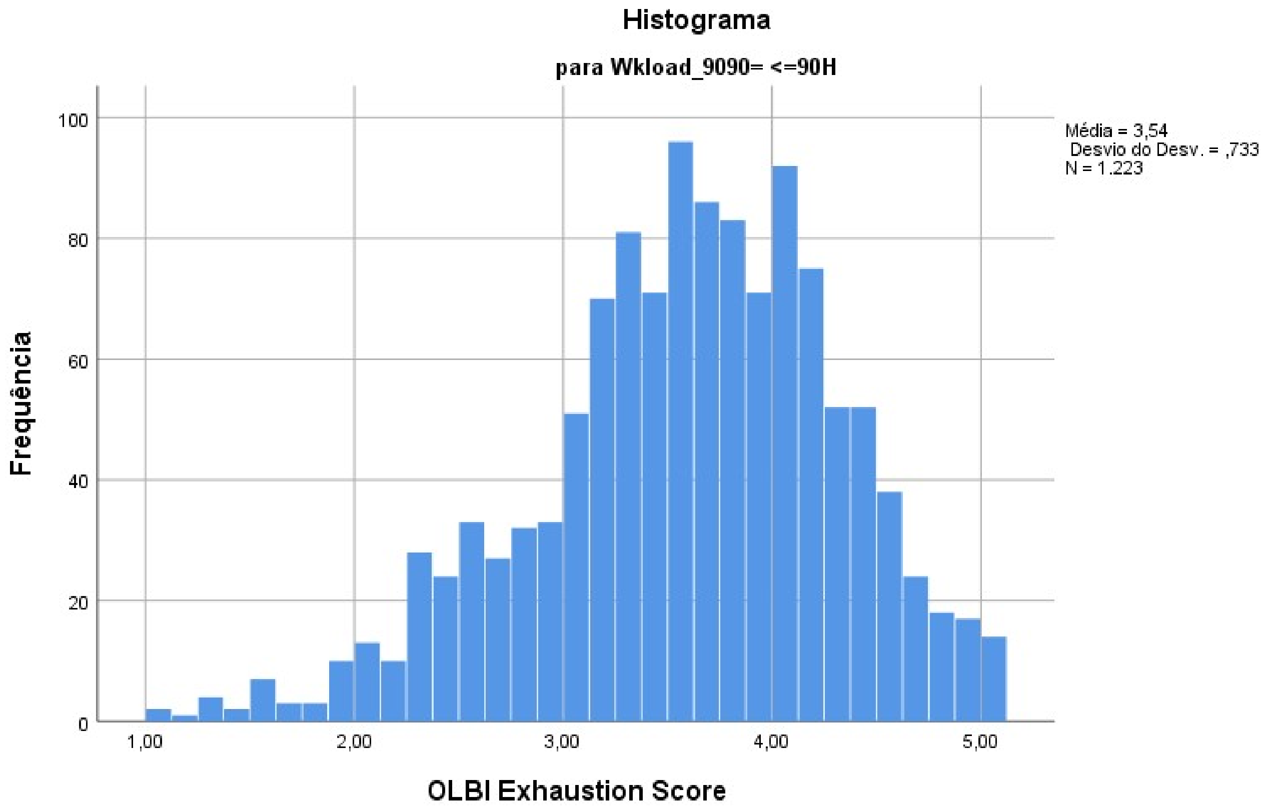

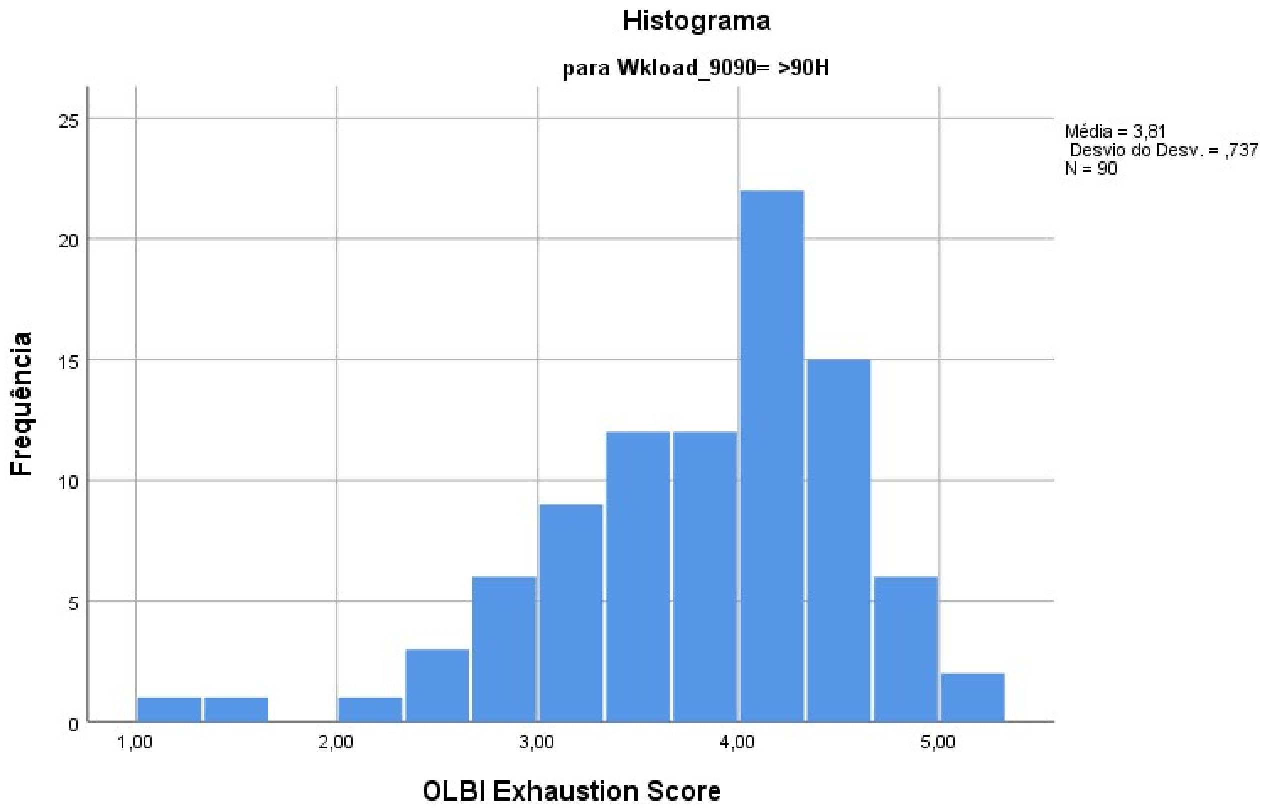

Gráfico Q-Q normais

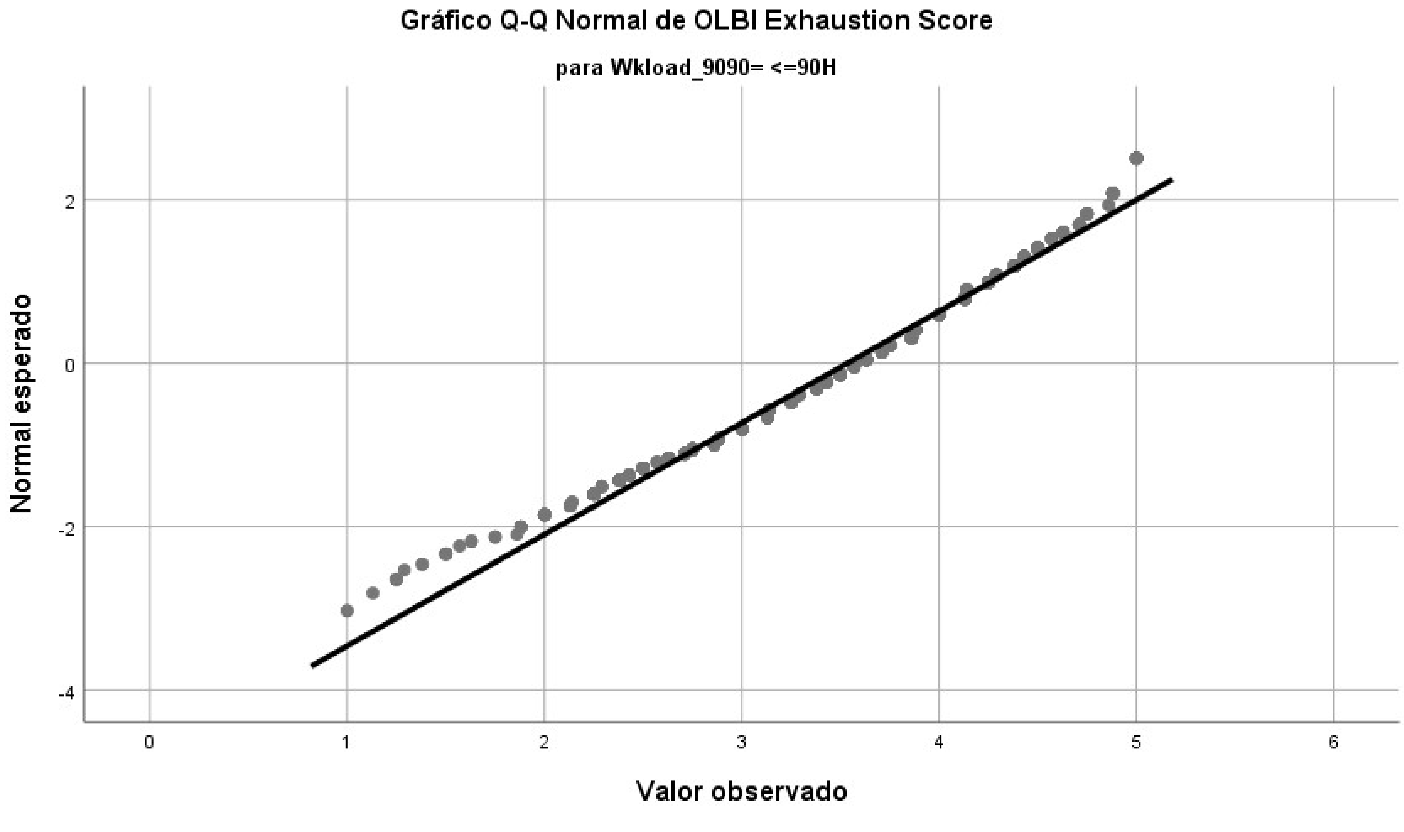

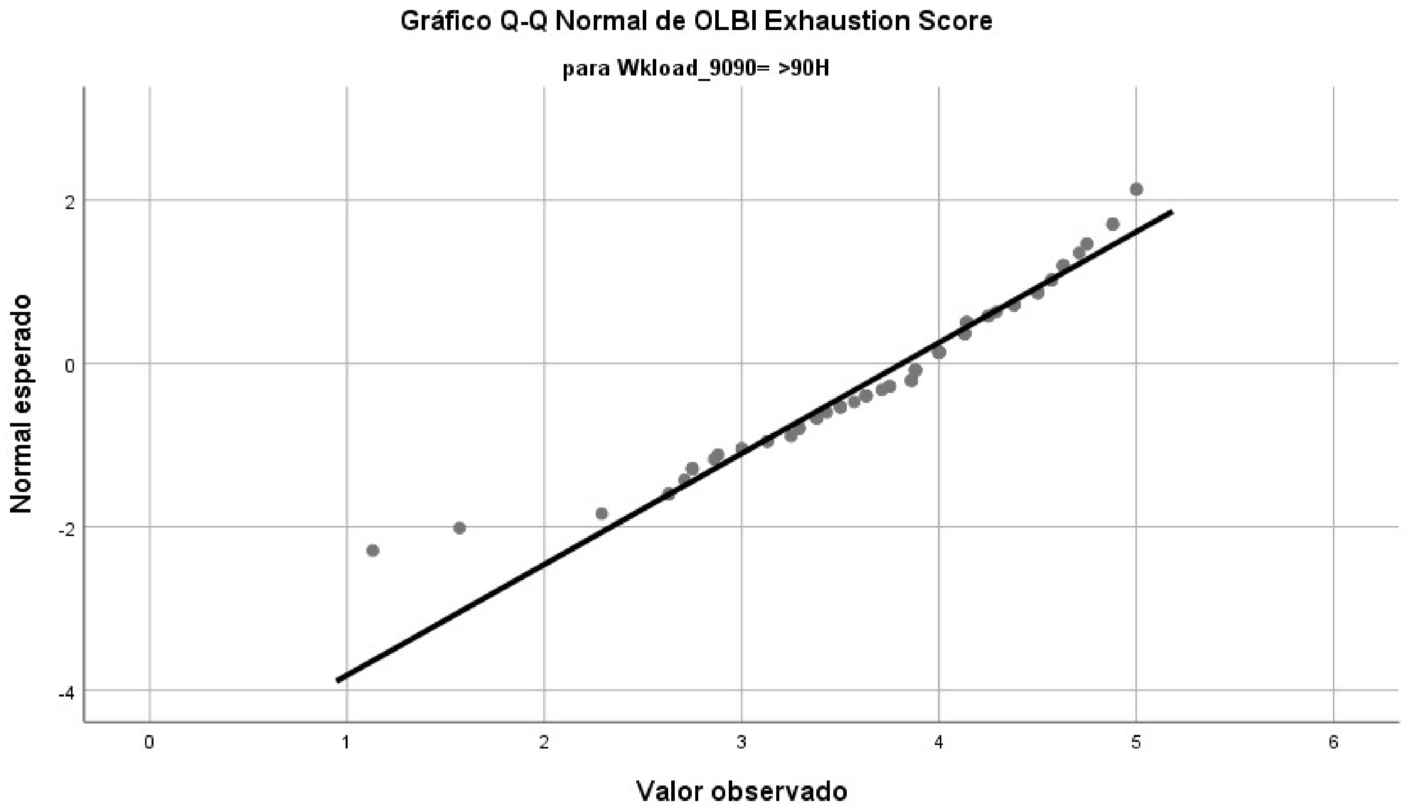

Gráfico Q-Q normais sem tendência

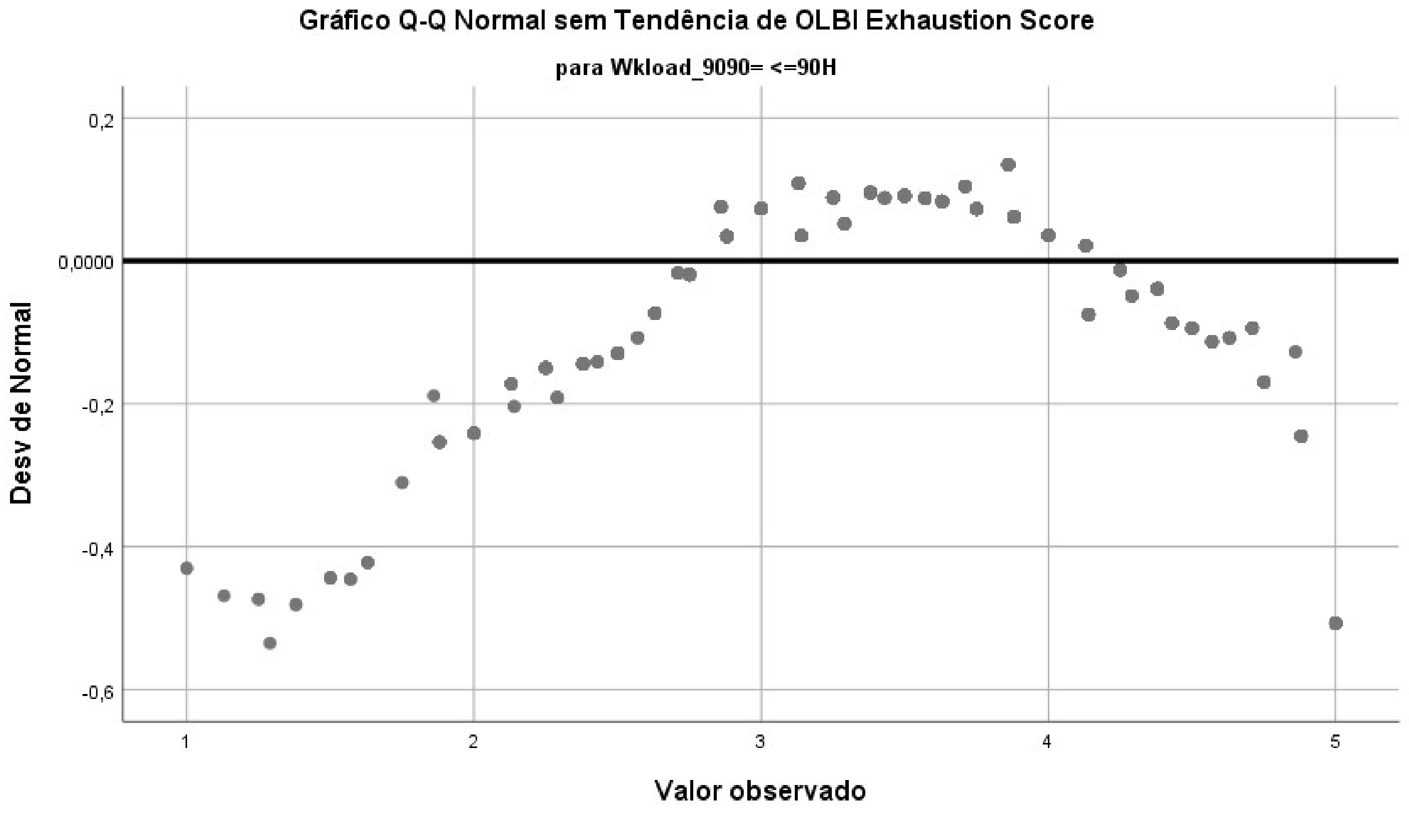

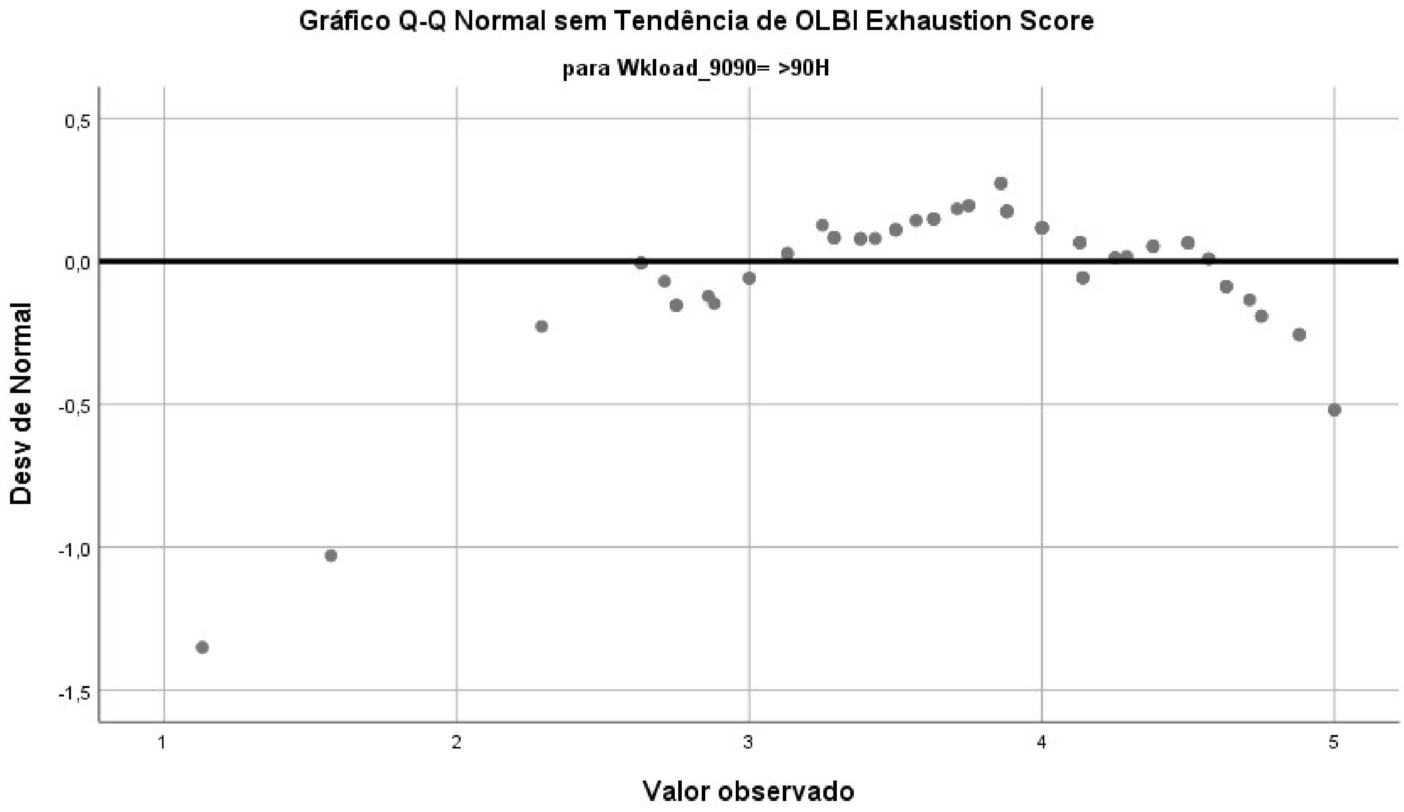


```
*Nonparametric Tests: Independent Samples.
NPTESTS
  /INDEPENDENT TEST (OLBI_D OLBI_E) GROUP (Sex)
  /MISSING SCOPE=ANALYSIS USERMISSING=EXCLUDE
  /CRITERIA ALPHA=0.05   CILEVEL=95.
```

Testes não paramétricos

Observações

Saída criada		21-SEP-2020 00:14:31
Comentários		
Entrada	Dados	C:\Users\User\Documents\Pesquisa\Fellow\FellowGenData_V1.sav
	Conjunto de dados ativo	ConjuntodeDados1
	Filtro	<none>
	Ponderação	<none>
	Arquivo Dividido	<none>
	N de linhas em arquivo de dados de trabalho	1313

Sintaxe		NPTESTS<br>/INDEPENDENT TEST (OLBI_D OLBI_E) GROUP (Sex)<br>/MISSING SCOPE=ANALYSIS USERMISSING=EXCLUDE<br>/CRITERIA ALPHA=0.05 CILEVEL=95.
Recursos	Tempo do processador	00:00:00,17
	Tempo decorrido	00:00:00,12

null : null

Resumo de Teste de Hipótese

	Hipótese nula	Teste	Sig.	Decisão
1	A distribuição de OLBI Disengagement Score é a mesma entre as categorias de Sex.	Teste U de Mann-Whitney de amostras independentes	,154	Reter a hipótese nula.
2	A distribuição de OLBI Exhaustion Score é a mesma entre as categorias de Sex.	Teste U de Mann-Whitney de amostras independentes	,000	Rejeitar a hipótese nula.

São exibidas significâncias assintóticas. O nível de significância é ,05.

Teste U de Mann-Whitney de amostras independentes

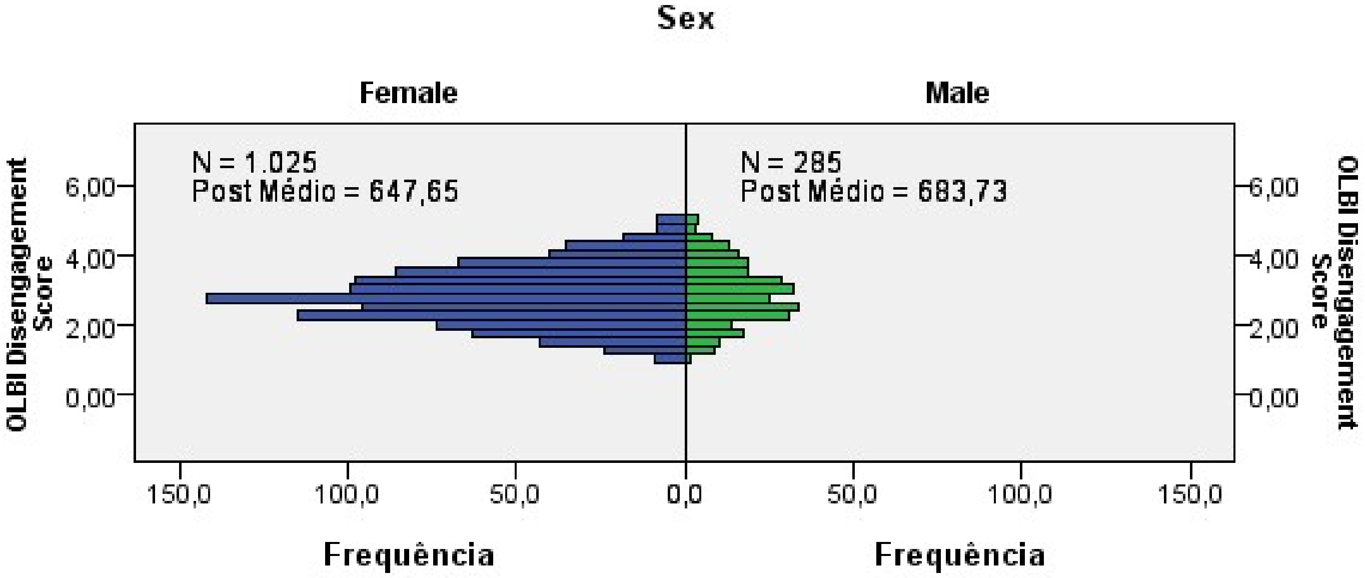

**N total**	1.310
**U de Mann-Whitney**	138.018,000
**Wilcoxon W**	663.843,000
**Estatística de teste**	138.018,000
**Erro padrão**	5.643,561
**Estatística de Teste Padronizado**	-1,425
**Sig. assintótico (teste de 2 lados)**	,154

Teste U de Mann-Whitney de amostras independentes

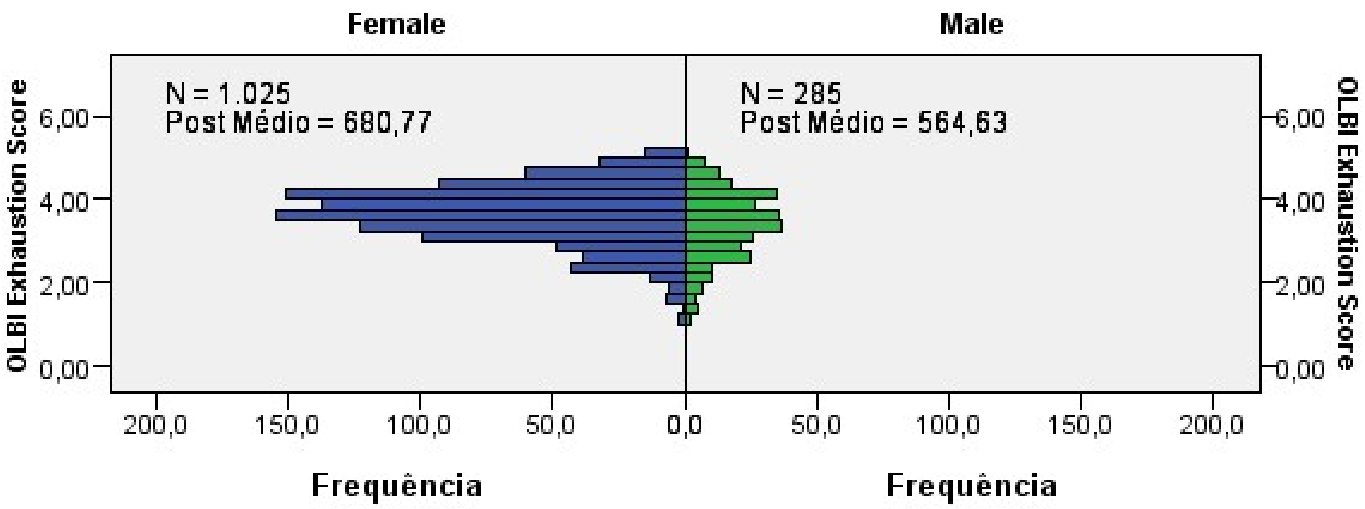

**N total**	1.310
**U de Mann-Whitney**	171.960,500
**Wilcoxon W**	697.785,500
**Estatística de teste**	171.960,500
**Erro padrão**	5.644,152
**Estatística de Teste Padronizado**	4,588
**Sig. assintótico (teste de 2 lados)**	,000

```
CROSSTABS
  /TABLES=Sex BY Wkload_5P Wkload_6060 Wkload_9090
  /FORMAT=AVALUE TABLES
  /STATISTICS=CHISQ PHI RISK
  /CELLS=COUNT ROW TOTAL
  /COUNT ROUND CELL.
```

Tabulações cruzadas

Observações

Saída criada		21-SEP-2020 00:18:27
Comentários		
Entrada	Dados	C:\Users\User\Documents\Pesquisa\Fellow\FellowGenData_V1.sav
	Conjunto de dados ativo	ConjuntodeDados1
	Filtro	<none>
	Ponderação	<none>
	Arquivo Dividido	<none>
	N de linhas em arquivo de dados de trabalho	1313
Tratamento de valor omisso	Definição de omisso	Os valores omissos definidos pelo usuário são tratados como omissos.
	Casos utilizados	As estatísticas de cada tabela são baseadas em todos os casos com dados válidos na(s) amplitude(s) especificada(s) para todas as variáveis de cada tabela.
Sintaxe		CROSSTABS<br>/TABLES=Sex BY Wkload_5P Wkload_6060 Wkload_9090<br>/FORMAT=AVALUE TABLES<br>/STATISTICS=CHISQ PHI RISK<br>/CELLS=COUNT ROW TOTAL<br>/COUNT ROUND CELL.
Recursos	Tempo do processador	00:00:00,02
	Tempo decorrido	00:00:00,02
	Dimensões solicitadas	2
	Células disponíveis	524245

```
Sex * Cumulative weekly workload (five categories)
```

Crosstab

			Cumulative weekly workload (five categories)					
			|--- 24h	>24h --- 60h	>60h --- 90h	>90h --- 120h	>120h	Total
Sex	Male	Contagem	1	99	152	28	5	285
		% em Sex	0,4%	34,7%	53,3%	9,8%	1,8%	100,0%
		% do Total	0,1%	7,6%	11,6%	2,1%	0,4%	21,8%
	Female	Contagem	10	430	528	53	4	1025
		% em Sex	1,0%	42,0%	51,5%	5,2%	0,4%	100,0%

	% do Total	0,8%	32,8%	40,3%	4,0%	0,3%	78,2%
Total	Contagem	11	529	680	81	9	1310
	% em Sex	0,8%	40,4%	51,9%	6,2%	0,7%	100,0%
	% do Total	0,8%	40,4%	51,9%	6,2%	0,7%	100,0%

Testes qui-quadrado

	Valor	gl	Significância Assintótica (Bilateral)
Qui-quadrado de Pearson	17,904[a]	4	,001
Razão de verossimilhança	16,202	4	,003
Associação Linear por Linear	13,725	1	,000
N de Casos Válidos	1310		

a. 2 células (20,0%) esperavam uma contagem menor que 5. A contagem mínima esperada é 1,96.

Medidas Simétricas

		Valor	Significância Aproximada
Nominal por Nominal	Fi	,117	,001
	V de Cramer	,117	,001
N de Casos Válidos		1310	

Estimativa de Risco

	Valor
Razão de Chances para Sex (Male / Female)	[a]

a. Não é possível calcular as estatísticas de Estimativa de Risco. Elas são computadas apenas para uma tabela 2*2 sem células vazias.

```
Sex * Cumulative weekly workload < or >= 60h (dichotomous)
```

Crosstab

			Cumulative weekly workload < or >= 60h (dichotomous)		
			<=60h	>60h	Total
Sex	Male	Contagem	100	185	285
		% em Sex	35,1%	64,9%	100,0%

		% do Total	7,6%	14,1%	21,8%
	Female	Contagem	440	585	1025
		% em Sex	42,9%	57,1%	100,0%
		% do Total	33,6%	44,7%	78,2%
Total		Contagem	540	770	1310
		% em Sex	41,2%	58,8%	100,0%
		% do Total	41,2%	58,8%	100,0%

Testes qui-quadrado

	Valor	gl	Significância Assintótica (Bilateral)	Sig exata (2 lados)	Sig exata (1 lado)
Qui-quadrado de Pearson	5,656[a]	1	,017		
Correção de continuidade[b]	5,337	1	,021		
Razão de verossimilhança	5,730	1	,017		
Teste Exato de Fisher				,017	,010
Associação Linear por Linear	5,651	1	,017		
N de Casos Válidos	1310				

a. 0 células (,0%) esperavam uma contagem menor que 5. A contagem mínima esperada é 117,48.

b. Computado apenas para uma tabela 2x2

Medidas Simétricas

		Valor	Significância Aproximada
Nominal por Nominal	Fi	-,066	,017
	V de Cramer	,066	,017
N de Casos Válidos		1310	

Estimativa de Risco

	Valor	Intervalo de confiança de 95% Inferior	Superior
Razão de Chances para Sex (Male / Female)	,719	,547	,944
Para grupo Cumulative weekly workload < or >= 60h (dichotomous) = <=60h	,817	,688	,972
Para grupo Cumulative weekly workload < or >= 60h (dichotomous) = >60h	1,137	1,029	1,258
N de Casos Válidos	1310		

Sex * Cumulative weekly workload < or >= 90h (dichotomous)

Crosstab

			Cumulative weekly workload < or >= 90h (dichotomous)		
			<=90H	>90H	Total
Sex	Male	Contagem	252	33	285
		% em Sex	88,4%	11,6%	100,0%
		% do Total	19,2%	2,5%	21,8%
	Female	Contagem	968	57	1025
		% em Sex	94,4%	5,6%	100,0%
		% do Total	73,9%	4,4%	78,2%
Total		Contagem	1220	90	1310
		% em Sex	93,1%	6,9%	100,0%
		% do Total	93,1%	6,9%	100,0%

Testes qui-quadrado

	Valor	gl	Significância Assintótica (Bilateral)	Sig exata (2 lados)	Sig exata (1 lado)
Qui-quadrado de Pearson	12,622[a]	1	,000		
Correção de continuidade[b]	11,699	1	,001		
Razão de verossimilhança	11,227	1	,001		
Teste Exato de Fisher				,001	,001
Associação Linear por Linear	12,613	1	,000		
N de Casos Válidos	1310				

a. 0 células (,0%) esperavam uma contagem menor que 5. A contagem mínima esperada é 19,58.

b. Computado apenas para uma tabela 2x2

Medidas Simétricas

		Valor	Significância Aproximada
Nominal por Nominal	Fi	-,098	,000
	V de Cramer	,098	,000
N de Casos Válidos		1310	

Estimativa de Risco

	Valor	Intervalo de confiança de 95%	
		Inferior	Superior

Razão de Chances para Sex (Male / Female)	,450	,287	,706
Para grupo Cumulative weekly workload < or >= 90h (dichotomous) = <=90H	,936	,895	,979
Para grupo Cumulative weekly workload < or >= 90h (dichotomous) = >90H	2,082	1,384	3,132
N de Casos Válidos	1310		

```
*Nonparametric Tests: Independent Samples.
NPTESTS
  /INDEPENDENT TEST (OLBI_D OLBI_E) GROUP (ComorbAny)
  /MISSING SCOPE=ANALYSIS USERMISSING=EXCLUDE
  /CRITERIA ALPHA=0.05   CILEVEL=95.
```

Testes não paramétricos

Observações

Saída criada		21-SEP-2020 00:23:28
Comentários		
Entrada	Dados	C:\Users\User\Documents\Pesquisa\Fellow\FellowGenData_V1.sav
	Conjunto de dados ativo	ConjuntodeDados1
	Filtro	<none>
	Ponderação	<none>
	Arquivo Dividido	<none>
	N de linhas em arquivo de dados de trabalho	1313
Sintaxe		NPTESTS /INDEPENDENT TEST (OLBI_D OLBI_E) GROUP (ComorbAny) /MISSING SCOPE=ANALYSIS USERMISSING=EXCLUDE /CRITERIA ALPHA=0.05 CILEVEL=95.
Recursos	Tempo do processador	00:00:00,11
	Tempo decorrido	00:00:00,12

null : null

Resumo de Teste de Hipótese

	Hipótese nula	Teste	Sig.	Decisão
1	A distribuição de OLBI Disengagement Score é a mesma entre as categorias de Any comorbidity (regardless COVID risk).	Teste U de Mann-Whitney de amostras independentes	,000	Rejeitar a hipótese nula.
2	A distribuição de OLBI Exhaustion Score é a mesma entre as categorias de Any comorbidity (regardless COVID risk).	Teste U de Mann-Whitney de amostras independentes	,000	Rejeitar a hipótese nula.

São exibidas significâncias assintóticas. O nível de significância é ,05.

Teste U de Mann-Whitney de amostras independentes

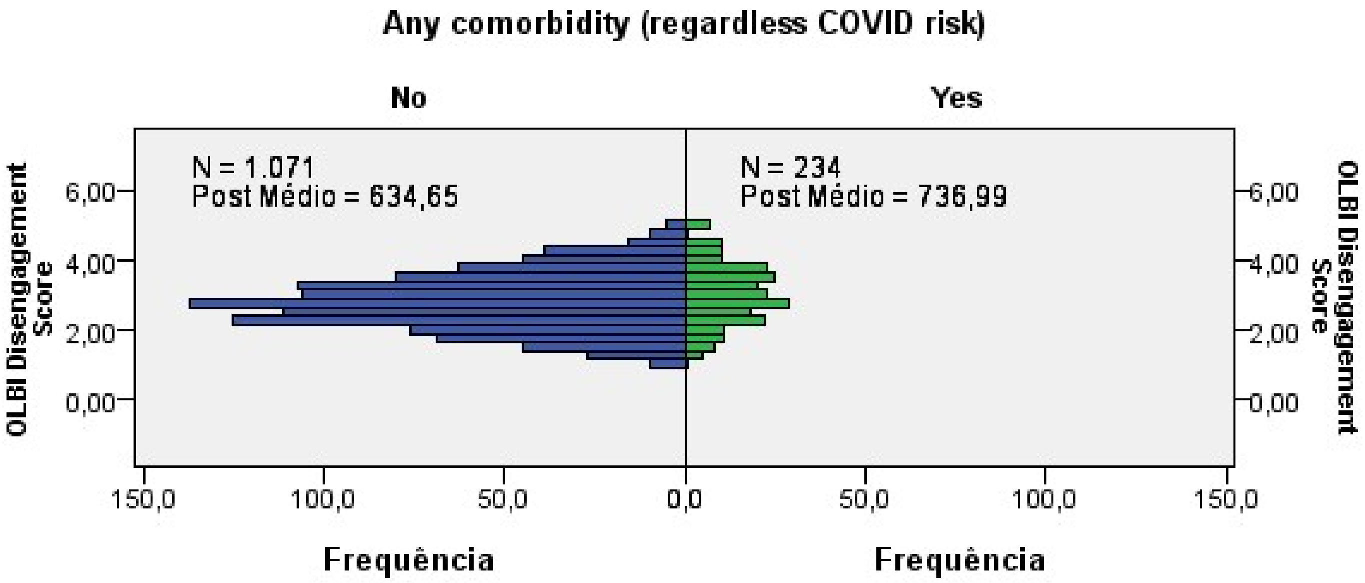

**N total**	1.305
**U de Mann-Whitney**	144.960,000
**Wilcoxon W**	172.455,000
**Estatística de teste**	144.960,000
**Erro padrão**	5.217,247
**Estatística de Teste Padronizado**	3,767
**Sig. assintótico (teste de 2 lados)**	,000

Teste U de Mann-Whitney de amostras independentes

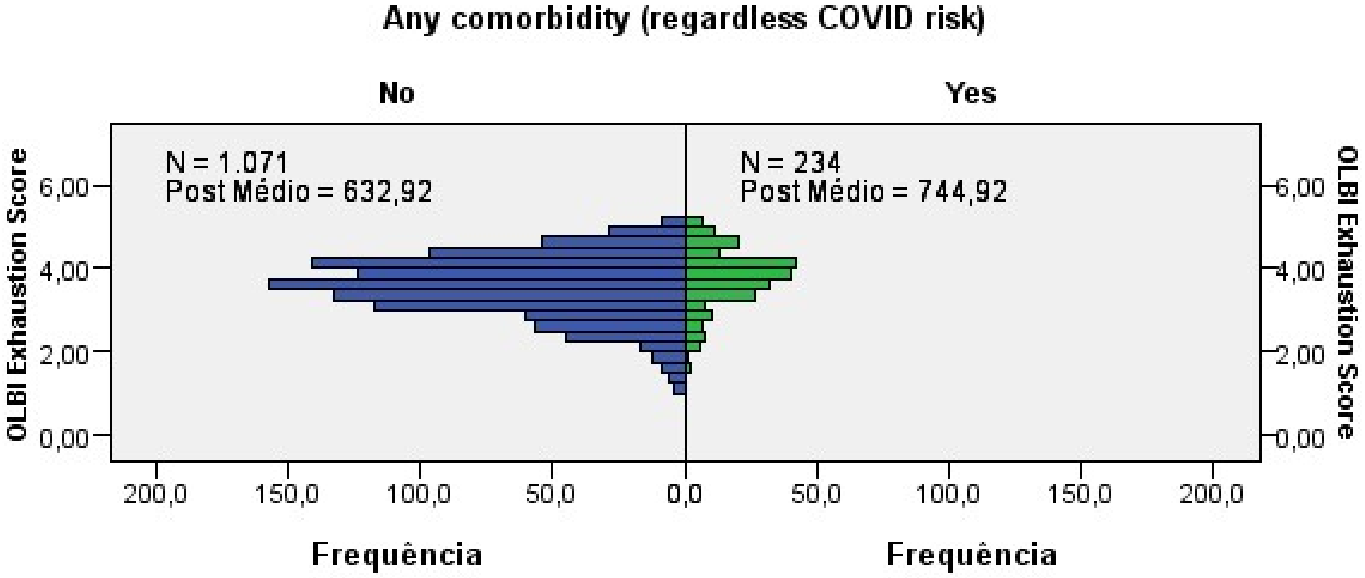

**N total**	1.305
**U de Mann-Whitney**	146.816,000
**Wilcoxon W**	174.311,000
**Estatística de teste**	146.816,000
**Erro padrão**	5.217,874
**Estatística de Teste Padronizado**	4,122
**Sig. assintótico (teste de 2 lados)**	,000

Observações

Saída criada		21-SEP-2020 00:31:57
Comentários		
Entrada	Dados	C:\Users\User\Documents\Pesquisa\Fellow\FellowGenData_V1.sav
	Conjunto de dados ativo	ConjuntodeDados1
	Filtro	<none>

	Ponderação	<none>
	Arquivo Dividido	<none>
	N de linhas em arquivo de dados de trabalho	1313
Sintaxe		GRAPH<br><br>/SCATTERPLOT(BIVAR)=BRCS_Score WITH OLBI_D<br>/MISSING=LISTWISE.
Recursos	Tempo do processador	00:00:00,22
	Tempo decorrido	00:00:00,22

```
T-TEST GROUPS=BRCS_Classif(0 1)
  /MISSING=ANALYSIS
  /VARIABLES=OLBI_D OLBI_E
  /CRITERIA=CI(.95).
```

Teste-T

Observações

Saída criada		21-SEP-2020 00:48:00
Comentários		
Entrada	Dados	C:\Users\User\Documents\Pesquisa\Fellow\FellowGenData_V1.sav
	Conjunto de dados ativo	ConjuntodeDados1
	Filtro	<none>
	Ponderação	<none>
	Arquivo Dividido	<none>
	N de linhas em arquivo de dados de trabalho	1313
Tratamento de valor omisso	Definição de omisso	Os valores omissos definidos pelo usuário são tratados como omissos.
	Casos utilizados	As estatísticas para cada análise são baseadas nos casos sem dados omissos ou fora do intervalo para qualquer variável da análise.
Sintaxe		T-TEST GROUPS=BRCS_Classif(0 1)<br>/MISSING=ANALYSIS<br>/VARIABLES=OLBI_D OLBI_E<br>/CRITERIA=CI(.95).
Recursos	Tempo do processador	00:00:00,02
	Tempo decorrido	00:00:00,01

Estatísticas de grupo

	BRCS Classification	N	Média	Erro Desvio	Erro padrão da média
OLBI Disengagement Score	Low resilience	813	2,9845	,79388	,02784
	Moderate to High	500	2,4318	,79865	,03572
OLBI Exhaustion Score	Low resilience	813	3,7136	,68374	,02398
	Moderate to High	500	3,2961	,74592	,03336

Teste de amostras independentes

		Teste de Levene para igualdade de variâncias		teste-t para Igualdade de Médias						
									95% Intervalo de Confiança da Diferença	
		Z	Sig.	t	df	Sig. (2 extremidades)	Diferença média	Erro padrão de diferença	Inferior	Superior
OLBI Disengagement Score	Variâncias iguais assumidas	,003	,956	12,222	1311	,000	,55272	,04522	,46401	,64144
	Variâncias iguais não assumidas			12,205	1051,189	,000	,55272	,04529	,46386	,64158
OLBI Exhaustion Score	Variâncias iguais assumidas	3,554	,060	10,373	1311	,000	,41743	,04024	,33848	,49637
	Variâncias iguais não assumidas			10,161	986,138	,000	,41743	,04108	,33681	,49805

```
*Nonparametric Tests: Independent Samples.
NPTESTS
  /INDEPENDENT TEST (OLBI_D OLBI_E) GROUP (Autonomy_Classif)
  /MISSING SCOPE=ANALYSIS USERMISSING=EXCLUDE
  /CRITERIA ALPHA=0.05   CILEVEL=95.
```

Testes não paramétricos

Observações

Saída criada		21-SEP-2020 00:51:00
Comentários		
Entrada	Dados	C:\Users\User\Documents\Pesquisa\Fellow\FellowGenData_V1.sav
	Conjunto de dados ativo	ConjuntodeDados1
	Filtro	<none>
	Ponderação	<none>
	Arquivo Dividido	<none>
	N de linhas em arquivo de dados de trabalho	1313

Sintaxe		NPTESTS<br>/INDEPENDENT TEST (OLBI_D OLBI_E) GROUP (Autonomy_Classif)<br>/MISSING SCOPE=ANALYSIS USERMISSING=EXCLUDE<br>/CRITERIA ALPHA=0.05 CILEVEL=95.
Recursos	Tempo do processador	00:00:00,14
	Tempo decorrido	00:00:00,12

null : null

Resumo de Teste de Hipótese

	Hipótese nula	Teste	Sig.	Decisão
1	A distribuição de OLBI Disengagement Score é a mesma entre as categorias de Classification of the percieved autonomy to self-conduct in the residency program.	Teste U de Mann-Whitney de amostras independentes	,000	Rejeitar a hipótese nula.
2	A distribuição de OLBI Exhaustion Score é a mesma entre as categorias de Classification of the percieved autonomy to self-conduct in the residency program.	Teste U de Mann-Whitney de amostras independentes	,000	Rejeitar a hipótese nula.

São exibidas significâncias assintóticas. O nível de significância é ,05.

Teste U de Mann-Whitney de amostras independentes

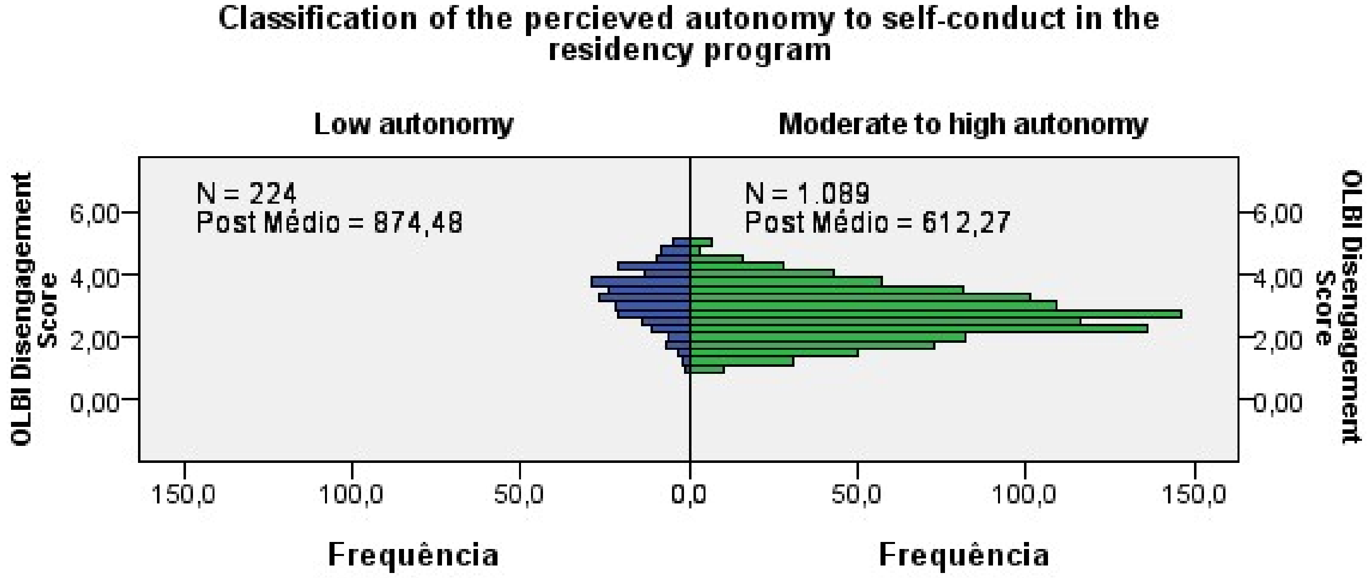

**N total**	1.313
**U de Mann-Whitney**	73.253,500
**Wilcoxon W**	666.758,500
**Estatística de teste**	73.253,500
**Erro padrão**	5.163,021
**Estatística de Teste Padronizado**	-9,435
**Sig. assintótico (teste de 2 lados)**	,000

Teste U de Mann-Whitney de amostras independentes

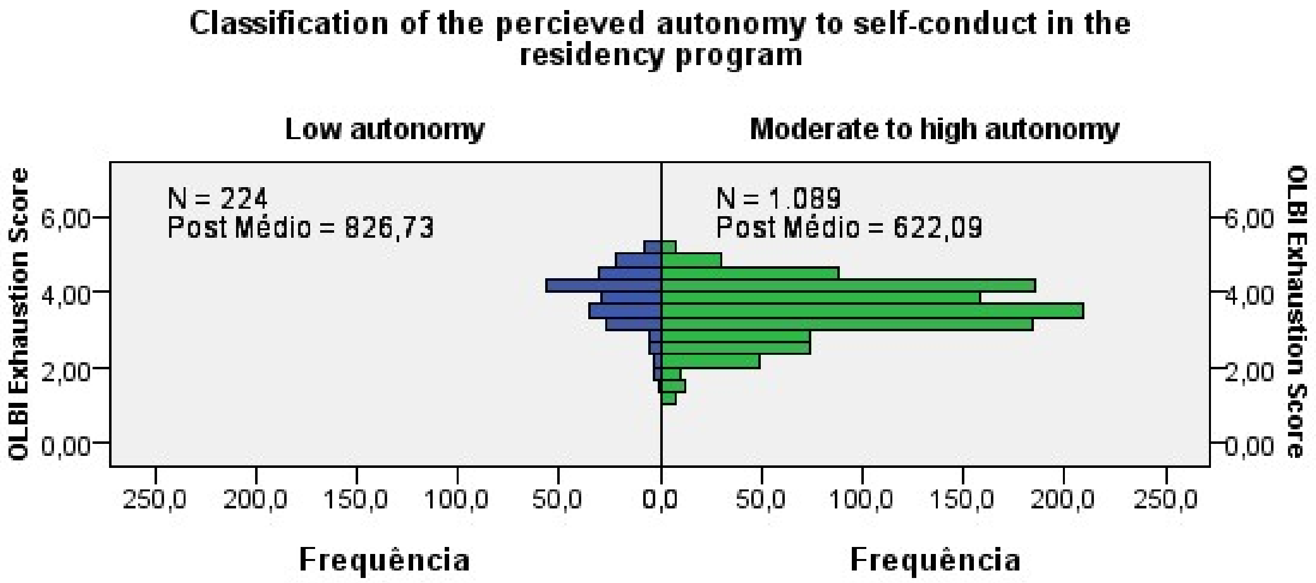

**N total**	1.313
**U de Mann-Whitney**	83.949,500
**Wilcoxon W**	677.454,500
**Estatística de teste**	83.949,500
**Erro padrão**	5.163,567
**Estatística de Teste Padronizado**	-7,363
**Sig. assintótico (teste de 2 lados)**	,000

```
T-TEST GROUPS=PedagStr_Classif(0 1)
  /MISSING=ANALYSIS
  /VARIABLES=OLBI_D OLBI_E
  /CRITERIA=CI(.95).
```

Teste-T

Observações

Saída criada		21-SEP-2020 00:52:37
Comentários		
Entrada	Dados	C:\Users\User\Documents\Pesquisa\Fellow\FellowGenData_V1.sav
	Conjunto de dados ativo	ConjuntodeDados1
	Filtro	<none>
	Ponderação	<none>
	Arquivo Dividido	<none>
	N de linhas em arquivo de dados de trabalho	1313
Tratamento de valor omisso	Definição de omisso	Os valores omissos definidos pelo usuário são tratados como omissos.
	Casos utilizados	As estatísticas para cada análise são baseadas nos casos sem dados omissos ou fora do intervalo para qualquer variável da análise.
Sintaxe		T-TEST GROUPS=PedagStr_Classif(0 1)<br>/MISSING=ANALYSIS<br>/VARIABLES=OLBI_D OLBI_E<br>/CRITERIA=CI(.95).
Recursos	Tempo do processador	00:00:00,00
	Tempo decorrido	00:00:00,02

Estatísticas de grupo

	Percieved adequacy of the pedagogic structure and availability of resouces in the residency program	N	Média	Erro Desvio	Erro padrão da média
OLBI Disengagement Score	Poor adequacy	558	3,1254	,78926	,03341
	Moderate to good adequacy	755	2,5142	,77919	,02836
OLBI Exhaustion Score	Poor adequacy	558	3,8236	,65989	,02794
	Moderate to good adequacy	755	3,3558	,72694	,02646

Teste de amostras independentes

		Teste de Levene para igualdade de variâncias		teste-t para Igualdade de Médias						
									95% Intervalo de Confiança da Diferença	
		Z	Sig.	t	df	Sig. (2 extremidades)	Diferença média	Erro padrão de diferença	Inferior	Superior
OLBI Disengagement Score	Variâncias iguais assumidas	,213	,644	13,974	1311	,000	,61121	,04374	,52540	,69702

	Variâncias iguais não assumidas			13,947	1191,689	,000	,61121	,04382	,52523	,69719
OLBI Exhaustion Score	Variâncias iguais assumidas	5,342	,021	11,982	1311	,000	,46774	,03904	,39116	,54432
	Variâncias iguais não assumidas			12,157	1257,148	,000	,46774	,03847	,39226	,54322

```
*Nonparametric Tests: Independent Samples.
NPTESTS
   /INDEPENDENT TEST (OLBI_D OLBI_E) GROUP (PPEAvail_Classif)
   /MISSING SCOPE=ANALYSIS USERMISSING=EXCLUDE
   /CRITERIA ALPHA=0.05   CILEVEL=95.
```

Testes não paramétricos

Observações

Saída criada		21-SEP-2020 00:55:54
Comentários		
Entrada	Dados	C:\Users\User\Documents\Pesquisa\Fellow\FellowGenData_V1.sav
	Conjunto de dados ativo	ConjuntodeDados1
	Filtro	<none>
	Ponderação	<none>
	Arquivo Dividido	<none>
	N de linhas em arquivo de dados de trabalho	1313
Sintaxe		NPTESTS /INDEPENDENT TEST (OLBI_D OLBI_E) GROUP (PPEAvail_Classif) /MISSING SCOPE=ANALYSIS USERMISSING=EXCLUDE /CRITERIA ALPHA=0.05 CILEVEL=95.
Recursos	Tempo do processador	00:00:00,14
	Tempo decorrido	00:00:00,12

null : null

Resumo de Teste de Hipótese

	Hipótese nula	Teste	Sig.	Decisão
1	A distribuição de OLBI Disengagement Score é a mesma entre as categorias de Perceived adequacy of the availability of personal protective equipment, when providing care for patients in the residency program.	Teste U de Mann-Whitney de amostras independentes	,000	Rejeitar a hipótese nula.
2	A distribuição de OLBI Exhaustion Score é a mesma entre as categorias de Perceived adequacy of the availability of personal protective equipment, when providing care for patients in the residency program.	Teste U de Mann-Whitney de amostras independentes	,000	Rejeitar a hipótese nula.

São exibidas significâncias assintóticas. O nível de significância é ,05.

Teste U de Mann-Whitney de amostras independentes

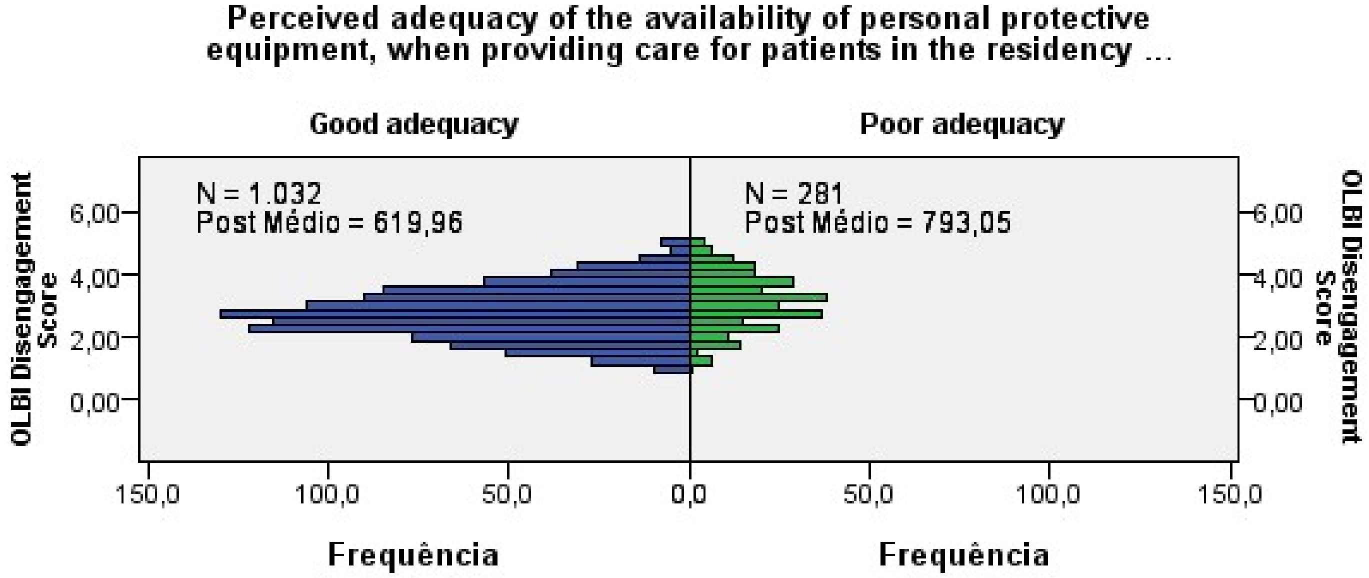

**N total**	1.313
**U de Mann-Whitney**	106.767,000
**Wilcoxon W**	639.795,000
**Estatística de teste**	106.767,000
**Erro padrão**	5.629,359
**Estatística de Teste Padronizado**	-6,791
**Sig. assintótico (teste de 2 lados)**	,000

Teste U de Mann-Whitney de amostras independentes

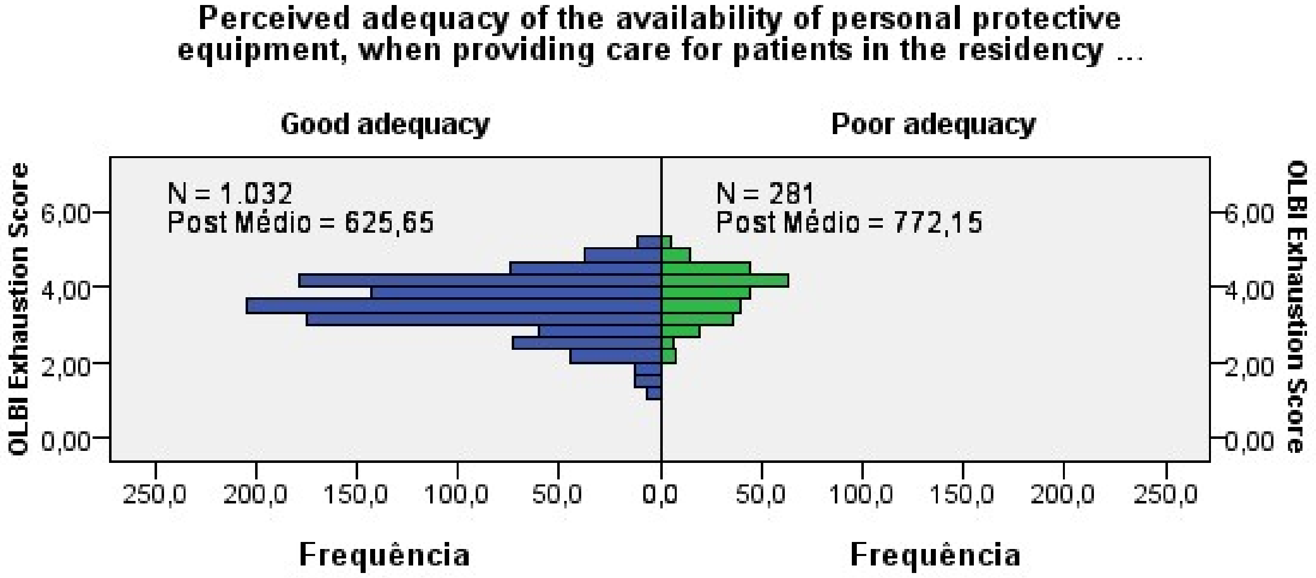

N total	1.313
U de Mann-Whitney	112.638,500
Wilcoxon W	645.666,500
Estatística de teste	112.638,500
Erro padrão	5.629,954
Estatística de Teste Padronizado	-5,747
Sig. assintótico (teste de 2 lados)	,000

```
T-TEST GROUPS=Wkload_6060(0 1)
  /MISSING=ANALYSIS
  /VARIABLES=OLBI_D OLBI_E
  /CRITERIA=CI(.95).
```

Teste-T

Observações

Saída criada		21-SEP-2020 00:58:11
Comentários		
Entrada	Dados	C:\Users\User\Documents\Pesquisa\Fellow\FellowGenData_V1.sav
	Conjunto de dados ativo	ConjuntodeDados1
	Filtro	<none>
	Ponderação	<none>
	Arquivo Dividido	<none>
	N de linhas em arquivo de dados de trabalho	1313
Tratamento de valor omisso	Definição de omisso	Os valores omissos definidos pelo usuário são tratados como omissos.
	Casos utilizados	As estatísticas para cada análise são baseadas nos casos sem dados omissos ou fora do intervalo para qualquer variável da análise.
Sintaxe		T-TEST GROUPS=Wkload_6060(0 1) /MISSING=ANALYSIS /VARIABLES=OLBI_D OLBI_E /CRITERIA=CI(.95).
Recursos	Tempo do processador	00:00:00,02
	Tempo decorrido	00:00:00,02

Estatísticas de grupo

	Cumulative weekly workload < or >= 60h (dichotomous)	N	Média	Erro Desvio	Erro padrão da média
OLBI Disengagement Score	<=60h	541	2,7426	,83801	,03603
	>60h	772	2,7960	,84037	,03025
OLBI Exhaustion Score	<=60h	541	3,4599	,75677	,03254
	>60h	772	3,6210	,71457	,02572

Teste de amostras independentes

		Teste de Levene para igualdade de variâncias		teste-t para Igualdade de Médias					95% Intervalo de Confiança da Diferença	
		Z	Sig.	t	df	Sig. (2 extremidades)	Diferença média	Erro padrão de diferença	Inferior	Superior
OLBI Disengagement Score	Variâncias iguais assumidas	,283	,595	-1,134	1311	,257	-,05338	,04706	-,14571	,03895
	Variâncias iguais não assumidas			-1,135	1164,298	,257	-,05338	,04704	-,14567	,03892

OLBI Exhaustion Score	Variâncias iguais assumidas	2,636	,105	-3,925	1311	,000	-,16113	,04106	-,24167	-,08058
	Variâncias iguais não assumidas			-3,885	1119,504	,000	-,16113	,04147	-,24250	-,07975

```
*Nonparametric Tests: Independent Samples.
NPTESTS
  /INDEPENDENT TEST (OLBI_D OLBI_E) GROUP (Wkload_9090)
  /MISSING SCOPE=ANALYSIS USERMISSING=EXCLUDE
  /CRITERIA ALPHA=0.05   CILEVEL=95.
```

Testes não paramétricos

Observações

Saída criada		21-SEP-2020 00:59:18
Comentários		
Entrada	Dados	C:\Users\User\Documents\Pesquisa\Fellow\FellowGenData_V1.sav
	Conjunto de dados ativo	ConjuntodeDados1
	Filtro	<none>
	Ponderação	<none>
	Arquivo Dividido	<none>
	N de linhas em arquivo de dados de trabalho	1313
Sintaxe		NPTESTS /INDEPENDENT TEST (OLBI_D OLBI_E) GROUP (Wkload_9090) /MISSING SCOPE=ANALYSIS USERMISSING=EXCLUDE /CRITERIA ALPHA=0.05 CILEVEL=95.
Recursos	Tempo do processador	00:00:00,13
	Tempo decorrido	00:00:00,13

null : null

Resumo de Teste de Hipótese

	Hipótese nula	Teste	Sig.	Decisão
1	A distribuição de OLBI Disengagement Score é a mesma entre as categorias de Cumulative weekly workload < or >= 90h (dichotomous).	Teste U de Mann-Whitney de amostras independentes	,002	Rejeitar a hipótese nula.
2	A distribuição de OLBI Exhaustion Score é a mesma entre as categorias de Cumulative weekly workload < or >= 90h (dichotomous).	Teste U de Mann-Whitney de amostras independentes	,000	Rejeitar a hipótese nula.

São exibidas significâncias assintóticas. O nível de significância é ,05.

Teste U de Mann-Whitney de amostras independentes

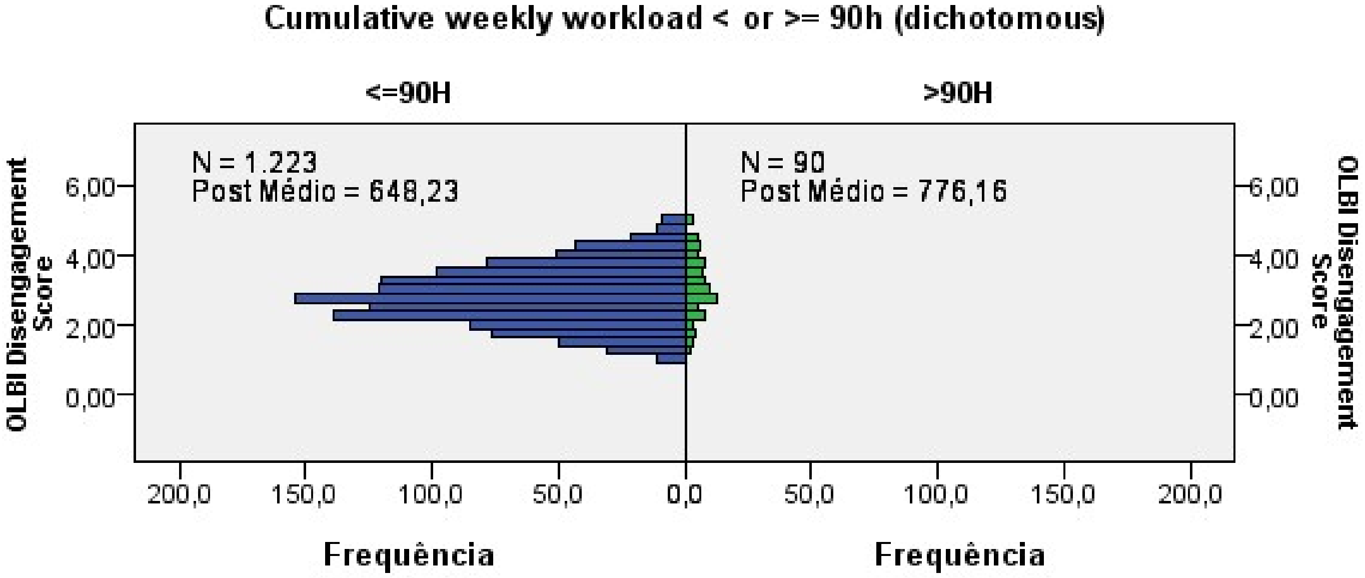

**N total**	1.313
**U de Mann-Whitney**	65.759,500
**Wilcoxon W**	69.854,500
**Estatística de teste**	65.759,500
**Erro padrão**	3.468,170
**Estatística de Teste Padronizado**	3,092
**Sig. assintótico (teste de 2 lados)**	,002

Teste U de Mann-Whitney de amostras independentes

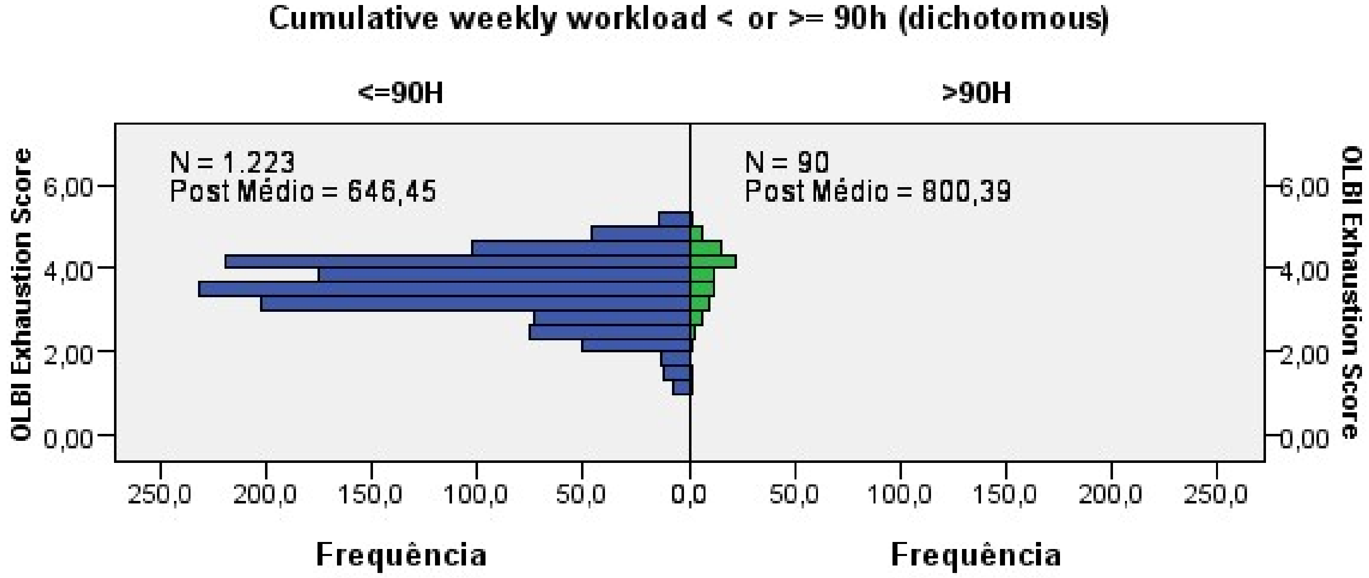

**N total**	1.313
**U de Mann-Whitney**	67.940,500
**Wilcoxon W**	72.035,500
**Estatística de teste**	67.940,500
**Erro padrão**	3.468,537
**Estatística de Teste Padronizado**	3,721
**Sig. assintótico (teste de 2 lados)**	,000
