# Supplementary material for: High prevalence of burnout syndrome among medical and nonmedical residents during the COVID-19 pandemic
Source: PLoS One. 2022 Nov 22;17(11):e0267530. doi: 10.1371/journal.pone.0267530 (PMC9681108; doi:10.1371/journal.pone.0267530)
Supplement: Attachment — Submitted filename: My Comments .docx [file pone.0267530.s003.docx]

**Manuscript Number**: PONE-D-22-10606

**Title:** High prevalence of burnout syndrome among medical and nonmedical residents during the COVID-19 pandemic

Thank you for inviting me to review this manuscript. This study addresses an important issue concerning the COVID-19 impact. Nevertheless, I do have several concerns in this particular study I would like to address.

**Comments** in the article which needs clarification, rewrites and/or additional information

**Title:**

Why authors included the burnout only in the title despite measuring other conditions as anxiety, stress, depression, Resilience and others? Even should refer to it.

Also why authors involved medical and non-medical residents despite there were no difference between them regarding burnout syndrome at the end. (The overall prevalence of burnout in our sample was 33.3%, with no significant differences between medical residents and nonmedical residents (35% vs. 31.6%, p = 0.191).

**The Suggestive title** (High prevalence of burnout and its associated factors among sample of Brazilians health residents during the COVID-19 pandemic).

**Abstract:**

Rewrite methods as: Healthcare residents were assessed for burnout (Oldenburg Burnout Inventory (OLBI)), resilience (brief resilient coping scale (BRCS), anxiety, stress and depression (depression, anxiety and stress scale (DASS-21) and Patient Health Questionnaire (PHQ-9)…………………etc.

**Introduction:**

- In early 2020, COVID-19, caused by a new coronavirus (SARS-CoV-2): write full name of SARS before writing the abbreviation (Severe acute respiratory syndrome (SARS).
- The objective of the present study was to evaluate the prevalence of burnout syndrome among health professionals in training, medical residencies (how to evaluate the prevalence, better to replace the action verb to determine) and write the goal if possible (what is the long-term outcome after determining the prevalence?

**Material and methods:**

- Mention the study design and study period (i.e. a descriptive online cross-sectional survey design was conducted from ……….to …….. (Write date beside month and year).
- Determine the type of sampling and the sampling technique (is it convenience sampling? How the authors reach to the healthcare residents all across the Brazil as mentioned in the abstract? Was it probability or non-probability sampling?
- How the sample size was calculated? Which software program was used (please write the reference) does they have list for all the public and private hospitals and health centers?
- Write in details about the study participants, selection criteria (inclusion and exclusion). Write the examples for medical and non-medical residents. Was there a pilot study?
- Concerning the questionnaire, was it structured as written? Or semi-structured?

What about the questions for the socio-demographic data and chronic diseases?

- In the data analysis and management: what about the normality test? The authors used parametric tests which mean that data was normally distributed. Also write the dependent and independent variables (please clarify and write in details)

**Results:**

- In table 1 the number of female (n=1025), male (n=285) isn’t equal to the total number (n= 1313 as mentioned in the table)
- Question (Increased risk for severe forms of COVID-19 in table 1) : what does this question mean, how the participant know whether they are at increased risk or not**?**
- Activity outside the residency program: give example for these activities as a note below the table**.**
- Where is the table for these results? (The mean (SD) age, for the overall sample, was 27.8 (4.4) years, and the mean scores for the instruments were as follows: OLBI-D, 2.8 (0.8); OLBI-E, 3.6 (0.7); OLBI Total, 3.2 (0.7); BRCS, 12.4 (3.8); DASS-21 depression, 15.3 (11.3); DASS-21 anxiety, 12.1 (10.3); DASS-21 stress, 20.3 (10.7); PHQ-9, 12.0 (6.5); perception of autonomy, 6.5 (2.1); and adequacy of the educational structure, 5.8 (2.5).
- **Table 4**: check the entire percentage % in the table. How it was calculated? for example

Number of male 94 (33%), number of female 342 (33.4%) how is it?

- Table 5: **Differences Between Genders Regarding the Various Variables Studied**. Why authors made this table despite it wasn’t from the objectives of the study to show the gender difference.
- Where the table for correlation as it is mentioned in the discussion and the abstract?

**Discussion:**

- The discussions missed data and interpretations on the regression analysis also the recommendations at the end.

I hope that the above comments and suggestions may be helpful for you

*Prof. Dr. Marwa Zalat*

*MD community, occupational and environmental medicine*
